# Enantioselective Phase-Transfer-Catalyzed Synthesis of Spirocyclic Azetidine Oxindoles

**DOI:** 10.1021/acs.orglett.4c00358

**Published:** 2024-03-06

**Authors:** Alexander J. Boddy, Aditya K. Sahay, Emma L. Rivers, Andrew J. P. White, Alan C. Spivey, James A. Bull

**Affiliations:** † Department of Chemistry, Imperial College London, Molecular Sciences Research Hub, White City Campus, Wood Lane, London W12 0BZ, U.K.; ‡ Hit Discovery, Discovery Sciences, R&D, AstraZeneca, Cambridge, U.K.

## Abstract

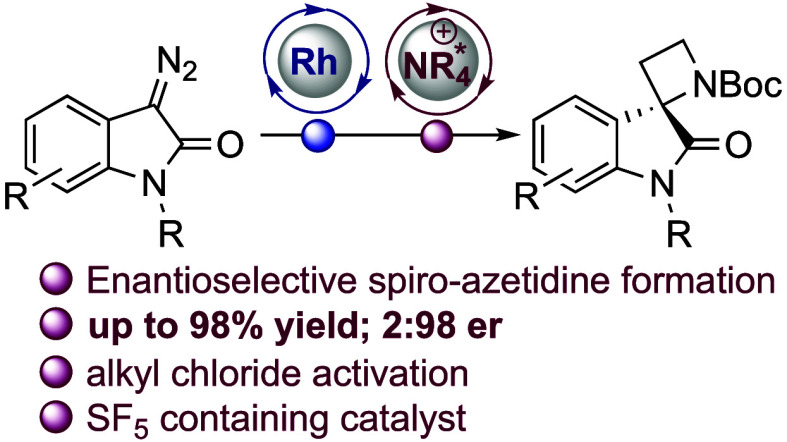

Spiro-3,2′-azetidine oxindoles combine two independently important pharmacophores in an understudied spirocyclic motif that is attractive for medicinal chemistry. Here, the enantioselective synthesis of these structures is achieved in up to 2:98 er through intramolecular C–C bond formation, involving activation of the substrate with a novel SF_5_-containing chiral cation phase-transfer (PT) catalyst. The products are readily elaborated/deprotected to afford medicinally relevant enantioenriched compounds. Control experiments suggest an interfacial PT mechanism, whereby catalytic asymmetric induction is achieved through the activation of the chloride leaving group.

Phase transfer (PT) catalysis enables the rate enhancement of reactions between substrates partitioned in immiscible phases.^1^ The use of chiral PT catalysts now constitutes a major field of asymmetric synthesis.^2^ The relatively mild conditions, sustainability, and low cost associated with this type of asymmetric organocatalysis have led to numerous applications within the pharmaceutical industry.^3^ Following seminal work by Merck,^4^ asymmetric intermolecular alkylation has become a benchmark reaction of this type,^5,6^ yet examples of intramolecular reactions remain relatively rare, likely due to the difficulty of suppressing the rate of the uncatalyzed background reaction.^7^

Spirocyclic oxindoles are important structures within bioactive natural products and active pharmaceutical ingredients (Figure 1A).^8^ Four-membered ring-containing spirocycles have attracted recent attention as they provide a rigid 3D framework from which to build bioactive agents in novel IP space.^9^ New methods to access enantioenriched azetidines are also highly desirable.^10^ Spiro-3,2′-azetidine oxindoles therefore present attractive scaffolds for medicinal chemistry but remain unexplored.^8a,11^ Isatin derivatives are privileged substrates in asymmetric PT catalysis to prepare enantioenriched oxindoles.^12^ Intramolecular C–C bond formation cyclization has rarely been investigated on these substrates, but there are some notable examples. Merck developed the synthesis of a CGRP antagonist using a novel bisquaternized cinchona alkaloid PT catalyst achieving up to 97:3 er,^13^ and Ooi developed an enantioselective amination to give spiropyrrolidine and piperidine oxindoles using a triazolium PT catalyst in up to 98.5:1.5 er (Figure 1B).^14^

**Figure 1:**
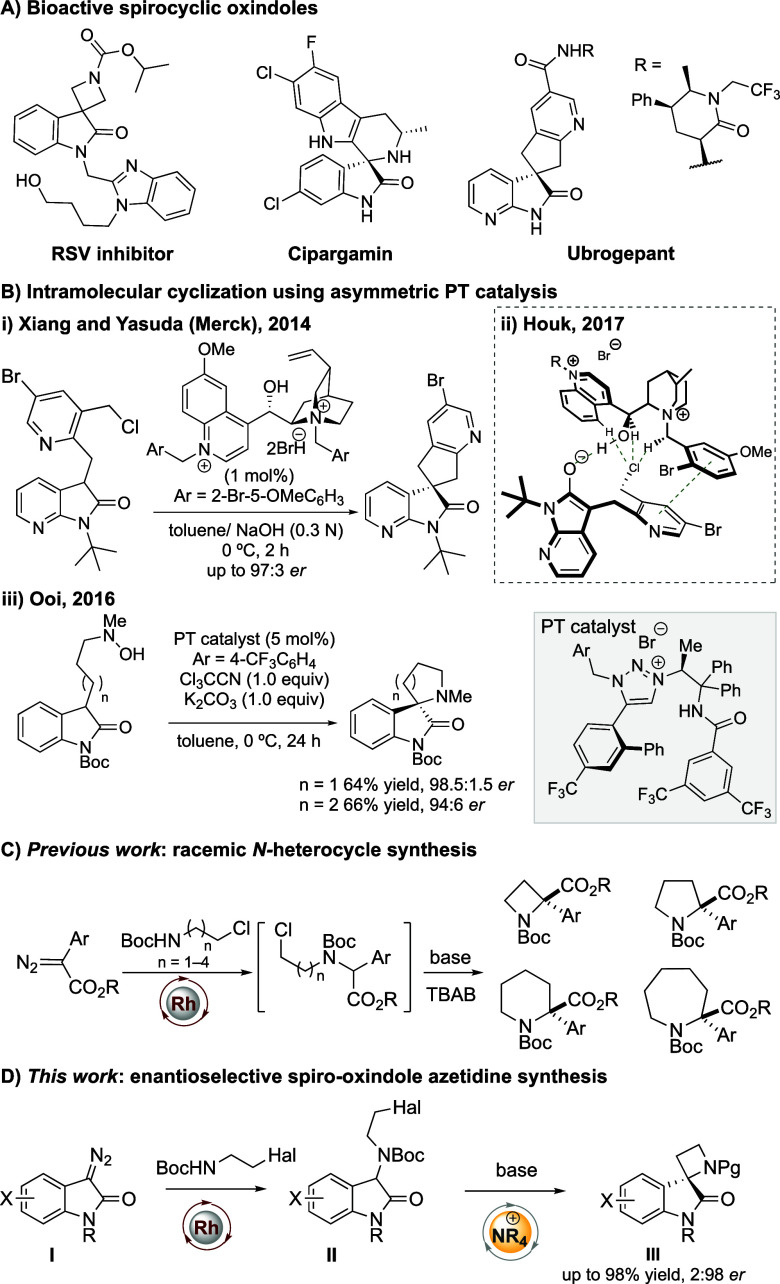
Spirocyclic oxindoles in medicinal chemistry and recent advances in intramolecular cyclization catalyzed by a chiral cation.

As part of our interest in four-membered heterocycles in medicinal chemistry, we previously reported strategies for their synthesis involving intramolecular C–C bond formation.^15,16^ In particular, we developed an N–H insertion/C–C cyclization strategy to form 4- to 7-membered saturated N-heterocycles from acyclic diazo compounds in racemic form (Figure 1C).^16^ Identifying an opportunity to access compounds in valuable and novel chemical space, we considered that a suitable PT catalyst may enhance the challenging cyclization to form 4-membered rings and provide a mechanism for asymmetric induction.

Here, we describe the synthesis of spirocyclic azetidine oxindoles from a new class of substrates, isatin-derived diazo compounds. Moreover, we render this C–C bond-forming cyclization enantioselective in the first example of such a process in 4-membered rings, using a novel and readily accessible SF_5_-containing cinchona alkaloid derived PT catalyst in high yields and with up to 2:98 er (Figure 1D).

The required chloride cyclization precursors **II** were prepared by N–H insertion reactions of 3-diazo isatin compounds **I** (Figure 1D, see SI). For the conversion of chloride **2a** to spirocyclic oxindole **3**, we first explored various classes of PT catalysts in toluene with 50% aq. NaOH as base (Table 1; for full studies see SI). This initial catalyst screen included quinine-derived ammonium salts, for which electron-deficient *N*-benzyl derivatives gave the most promising levels of enantioselectivity, e.g., 4-CF_3_ benzyl derivative **Cat1** (entry 1, 23:77 er). The corresponding *ortho*- or *meta*-CF_3_-substituted benzyl-containing catalysts and their unsubstituted or more electron-rich congeners gave poorer results (see SI). Further screening of bases revealed that their use in solid form rather than as aqueous solutions led to better outcomes (for full studies, see SI). Indeed, the use of solid KOH and CsOH caused a significant increase in enantioselectivity and yield (entries 3 and 4). A solvent screen showed that *m*-xylene gave optimal levels of er (entry 5, up to 6:94 er). With these optimized conditions, the catalyst structure was further investigated. Methylation of the OH group (**Cat2**) or hydrogenation of the alkene (**Cat3**) gave reductions in yield and er (entries 6 and 7). Introducing substituents at C2 of the quinoline (**Cat4** and **Cat5**) was not beneficial (entries 8 and 9). However, changing to the *cinchonidine*-derived catalyst **Cat6**, which lacks the quinoline C6 methoxy group, gave an improved yield and enantioselectivity (entry 10). Finally, we found that a *para*-SF_5_-substituted *N*-benzyl substituent (**Cat7**) led to optimal yield and selectivity (entry 11, 3:97 er).^17^ The enantiomeric product could be obtained by using pseudoenantiomeric **Cat8** derived from cinchonine, maintaining excellent er (entry 12, 96:4 er). In the absence of the PT catalyst the cyclization did not proceed under the final conditions (entry 13). We anticipate that the new SF_5_-containing catalysts, which can be readily prepared in one step from their respective parent cinchona alkaloids, may find broad application in asymmetric PT catalysis processes and should be considered in typical screening efforts.

**Table 1:**
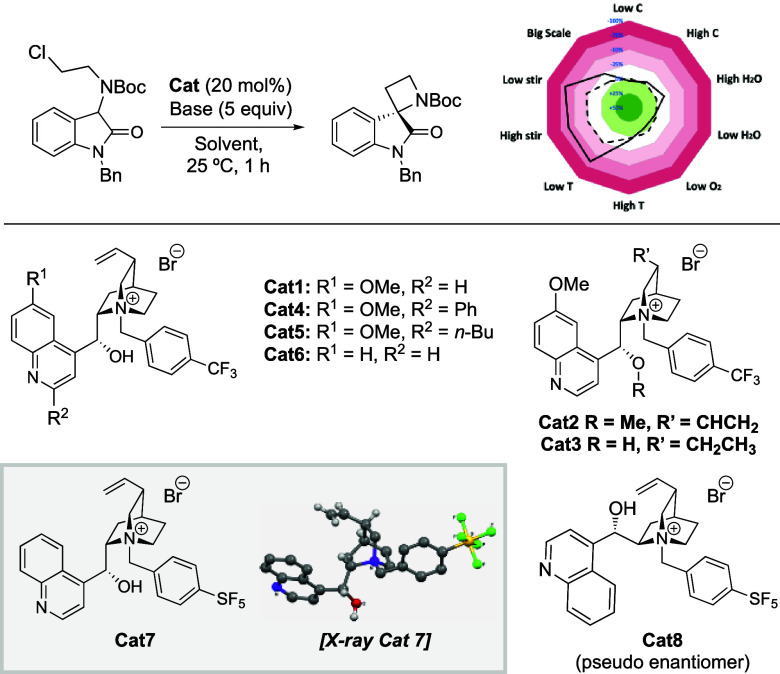

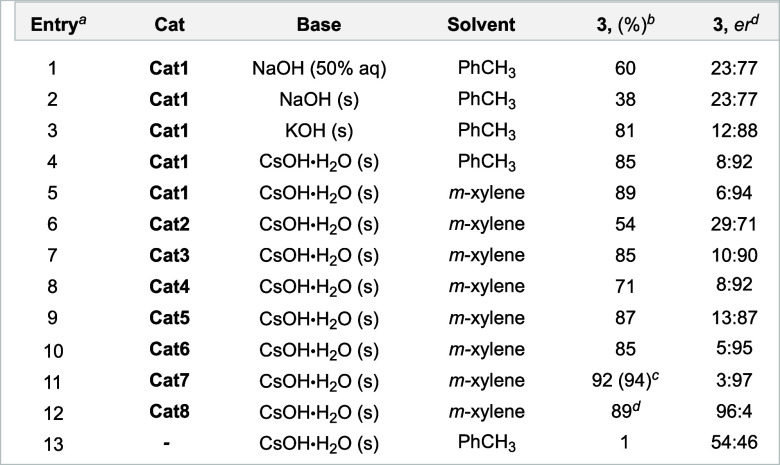
Selected Optimization of Enantioselective Cyclization

a Reactions on a 0.05 mmol scale.

b Yields are determined by in situ ^1^H NMR spectroscopy with respect to 1,3,5-trimethoxybenzene as an internal standard.

c Isolated yield on a 0.2 mmol scale.

d Enantiomeric ratio (er) determined by HPLC analysis of the crude reaction mixture with a chiral stationary phase. er reported as R:S, which for **3** corresponds to elution time in HPLC trace (see later for X-ray data and see SI for full details). For the sensitivity assessment: *T* = temperature, Bold line = yield, Dashed line = ee.

Other important aspects of the optimization were water content, stirring rate, and catalyst loading (see SI). A sensitivity assessment of the optimized reaction showed high tolerance to changes in conditions including higher H_2_O and O_2_ content, concentration, and temperature (Table 1, top right).^18^ The reaction was most sensitive to the stirring rate and low temperatures.

We then sought to explore the scope of the reaction, with respect to the structure of the oxindole (Scheme 1A). The enantioselective cyclization reaction was tolerant of a broad range of substituents on the oxindole aromatic ring (Scheme 1B).

**Scheme 1:**
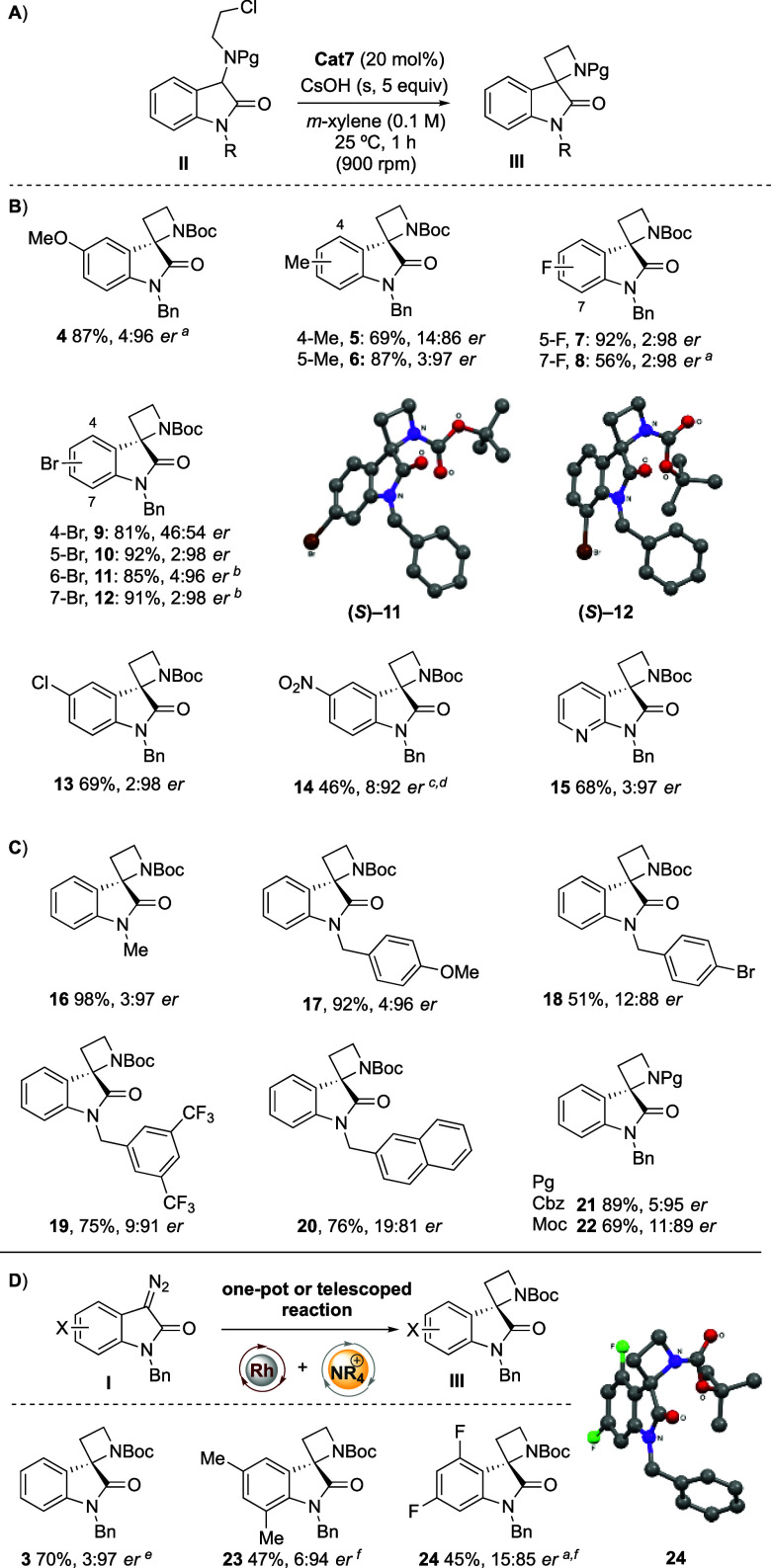
Scope of Enantioselective Cyclization Structure confirmed by X-ray. Structure and absolute configuration confirmed by X-ray (see inset images and SI). 0.1 mmol scale. 18 h reaction time. One-pot reaction. Yield reported from diazo compound. Telescoped reaction. Yield reported from diazo compound. Reactions on a 0.2 mmol scale. er determined by HPLC analysis on a chiral stationary phase, reported as R:S with S assigned as the major isomer based on X-ray data.

Electron-donating 5-methoxyoxindole derivatives (**4**) were formed in high yield, with 4:96 er. 5-Methyloxindole-azetidine **6** was also formed in high yield and 3:97 er, whereas the 4-Me derivative gave a lower er. Fluorine atoms at the 5- and 7-positions gave excellent er. Bromine substitution at each of the 4-, 5-, 6-, and 7-positions of the oxindole gave high yields, and all gave excellent er (**10**–**12**), except for 4-Br derivative **9**. Compound **9** was not formed enantioselectively, which together with the result of 4-substituted spirocyclic azetidines **5** suggests unfavorable interactions of substituents at this position with the catalyst (*vide infra*). Chloro and nitro derivatives gave high yields and enantioselectivity (**13**–**14**). 7-Azaoxindole spirocyclic azetidine **15** was obtained in moderate yield and high er (3:97). The absolute configuration of products **11** and **12** was determined from anomalous dispersion single-crystal X-ray diffraction data.

Variation of the oxindole N-substituent (Scheme 1C) showed that the benzyl group could be replaced with a methyl group (**16**), and high yield and er were maintained. *N*-PMB oxindole derivative **17** was formed in 92% yield and 4:96 er. Substitution of the benzyl group with electron-withdrawing substituents or replacement with a 2-naphthylmethyl group gave slightly lower yields and enantioselectivities (**18**–**20**). The er was well maintained on changing the Boc group to a Cbz group (**21**). Further reduction in the size of the carbamate-protecting group to a Moc group (**22**) gave a reduction in yield and er.

Enantioselective cyclization was also accomplished in one pot directly from *N*-benzyl diazo isatins **I**, circumventing the need to isolate the intermediate alkyl chlorides **II** (Scheme 1D). Thus, spirocycle **3** was prepared in 70% yield and 3:97 er directly from diazo isatin **1** (**I**, X = H) in *m*-xylene, which compares favorably with the 2-step process (**1**–**2a–3**, 66%/92%, 3:97 er, cf. Table 1, entry 11, and SI). For diazo isatins that have low solubility in *m*-xylene a telescoped approach can be taken in which the N–H insertion step is performed in CH_2_Cl_2_, and then the solvent is evaporated before addition of **Cat7**, base, and *m*-xylene for the cyclization step. This protocol gave spirocyclic azetidines **23** and **24** in moderate yields and good enantioselectivity.

A gram-scale (5 mmol) one-pot reaction was performed from diazo compound **1** to form spirocyclic oxindole azetidine **3** without a reduction in yield or enantioselectivity (Scheme 2). To demonstrate the potential for exploitation of the vectors from the spirocyclic scaffold, orthogonal deprotection of the Boc and Bn groups in spirocyclic azetidine **3** was explored: the former was removed on treatment with HCl and the latter using a modified Birch/Benkeser reduction (**25** and **26**, Scheme 2).^19^

**Scheme 2:**
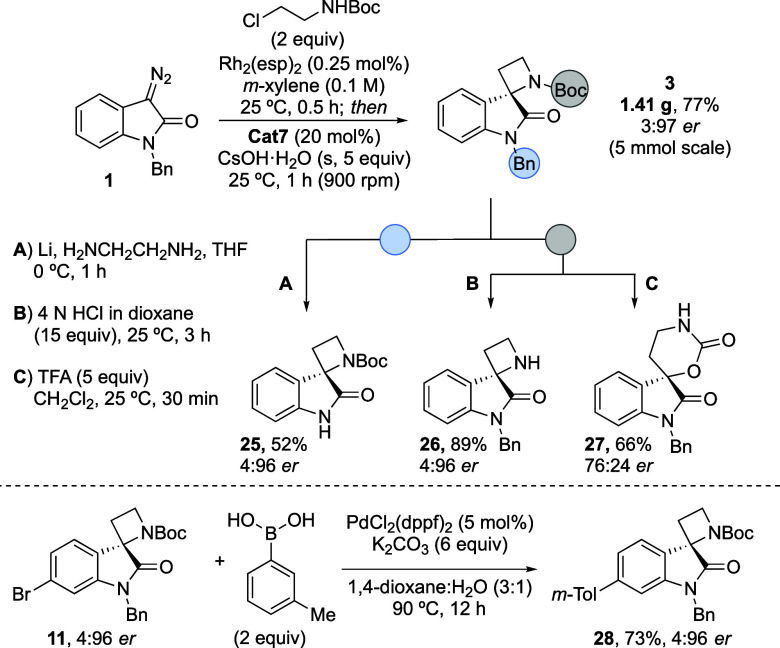
Gram-Scale Synthesis of **3** and Derivatization to Medicinally Relevant Fragments **25**–**28**

Both transformations proceeded with the retention of enantiopurity. Treatment of *N*-Boc spirocyclic azetidine **3** with TFA led to isolation of the ring-expanded spirocyclic oxazinan-2-one **27** with a slight reduction in er, likely because of partial carbocation formation.^20^ Suzuki cross coupling of 6-bromo-oxindole spirocyclic azetidine **11** was also successful, again with the retention of er.

To understand the origin of the stereoinduction, variation of the halide leaving group in cyclization precursor **II** was investigated (Scheme 3A). Unlike chloride **2a**, both the bromide and the iodide (**2b** and **2c**) show significant background rates in the absence of the catalyst, and consequently although both the PT-catalyzed reactions gave comparable yields, they gave significantly diminished levels of enantioselectivity. A more rapid background reaction was observed for 5-membered ring formation: without added catalyst racemic **30** was formed in quantitative yield within 1 h (Scheme 3B). Despite this background reaction, the catalyzed reaction still gave product **30** with an acceptable enantioselectivity (17:83 er).

**Scheme 3:**
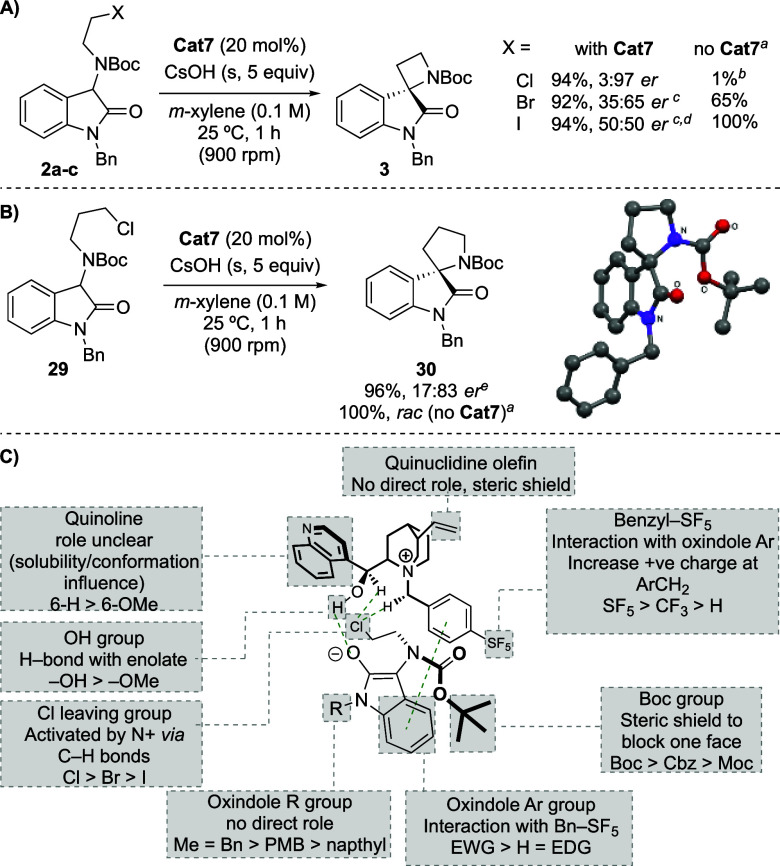
Investigations into the Mechanism of Enantioinduction Reactions on a 0.05 mmol scale, yields determined by in situ ^1^H NMR spectroscopy with respect to 1,3,5-trimethoxybenzene as an internal standard. In toluene. 0.1 mmol scale. 40 mol % cat. Structure confirmed by X-ray. Reactions on 0.2 mmol scale unless specified.

These observations suggest that deprotonation of the substrate likely does not involve the catalyst. In support of this, when the rate of 4-membered ring formation was studied with **Cat7**, substrate **2a** was almost completely consumed within 10 min, while product yield at 10 min was <10%, indicating that significant deprotonation of **2a** occurs prior to the catalyst accelerated step to form the enantioenriched product. This finding coupled with a significant yield and enantioselectivity dependence on the rate of stirring is suggestive of an interfacial PT mechanism.^21^

Despite extensive studies, notably by Denmark,^22^ to demystify the origin of stereoinduction during asymmetric PT-catalyzed reactions, models generally remain ad hoc and based on empirical observations related to a specific system. For intermolecular alkylations there have been numerous proposals.^4,5b,23,24^ In particular, Houk studied Merck’s intramolecular cyclization (Figure 1B(i))^13^ using DFT calculations.^25^ This work corroborated Pilego’s earlier proposal that an N + CH···Cl interaction is important for leaving group orientation and activation.^26^ This H-bond, along with one between the catalyst OH group and the oxindole, in concert with the oxindole *N*-*tert*-butyl group blocking access to the other face of the enolate (locked by a π–π interaction), was suggested to account for the enantioinduction in that reaction (see Figure 1B(ii)). We propose that asymmetric induction in our reactions also accrues from a chiral cation-directed cyclization in which the energy barrier to cyclization is lowered by substrate activation.^27^ By analogy with the Merck system, the *N*-Boc group on the nascent azetidine nitrogen, likely locked by either a π–π, a C–H−π, or a π–SF_5_ group interaction between the oxindole aryl ring and the catalyst’s SF_5_-substituted benzyl group, acts as a steric shield on the opposite face to the activated chloride leaving group (Scheme 3C).^27,28^

In summary, we developed a catalytic enantioselective synthesis of spirocyclic azetidine oxindoles. The novel SF_5_-containing catalyst is readily prepared in one step from a cinchona alkaloid. The scope of the reaction encompasses electron-poor and electron-rich substituents on the oxindole aryl ring. Different protecting and leaving groups have also been explored. The highly enantioenriched products can be readily prepared in a 2-step protocol from 3-diazo isatins or in one pot without isolation of the N–H insertion intermediate. The products offer the potential for elaboration to a plethora of medicinally relevant compounds. We have shown that these reactions likely proceed via a PT catalysis mechanism and that enantioinduction is achieved by chiral cation activation of the leaving group in concert with a key π-interaction between the catalyst and the substrate.

## Data Availability

The data underlying this study are available in the published article and its Supporting Information.

## Supporting Information Available

Experimental procedures, characterization data, and copies of ^1^H and ^13^C NMR spectra; catalyst preparation; full details of reaction optimization; X-ray crystallography data; and HPLC traces for enantioenriched compounds (PDF)
