# Supplementary material for: Enantioselective Phase-Transfer-Catalyzed Synthesis of Spirocyclic Azetidine Oxindoles
Source: Org Lett. 2024 Mar 6;26(10):2079–84. doi: 10.1021/acs.orglett.4c00358 (PMC10949229; doi:10.1021/acs.orglett.4c00358)
Supplement: Supplementary file 1 — ol4c00358_si_001.pdf [file ol4c00358_si_001.pdf]

SUPPORTING INFORMATION

Enantioselective Phase Transfer Catalyzed Synthesis of Spirocyclic Azetidine Oxindoles

Alexander J. Boddy,[a] Aditya K. Sahay,[a] Emma L. Rivers,[b] Andrew J. P. White,[a] Alan C. Spivey [a]* and James A. Bull [a]*

[a] Department of Chemistry, Imperial College London, Molecular Sciences Research Hub, White City Campus, Wood Lane W12 0BZ, UK
[b] Hit Discovery, Discovery Sciences, R&D, AstraZeneca, Cambridge, UK

*E-mail: j.bull@imperial.ac.uk
*E-mail: a.c.spivey@imperial.ac.uk

Table of Contents S1
General Experimental Considerations S2
Structures of Additional Compounds in SI S4
Preliminary Studies on O-H insertion reaction S6
Optimisation of N-H insertion reaction S7
Extended Optimisation of Enantioselective Cyclisation S10
Enantioselective Cyclisation – Summary of Key Findings S13
Enantioselective Cyclisation – Full Optimisation Table S15
Reaction Sensitivity S27
Effect of Stirring Rate S28
Catalyst Degradation S29
Diazo Compounds S31
Protected Amine Starting Materials S42
N-H Insertion Intermediates S46
Cyclised Products S58
Scale up and derivatisation reactions S73
Catalysts S76
NMR of Selected Compounds S98
Chiral HPLC Data S253
X-Ray Discussion S282
References S292

General Experimental Considerations

All non-aqueous reactions were carried out under an inert atmosphere (argon) with flame-dried glassware, using standard techniques. Anhydrous solvents were obtained by filtration through drying columns (CH_2Cl_2 and DMF) or used as supplied (benzene). Reactions in sealed tubes were run using Biotage microwave vials (2–5 mL) and aluminium caps with molded butyl (when using benzene) or butyl/PTFE (when using CH_2Cl_2) septa. Stirring rate (900 rpm), stir bar size (3 × 10 mm, PTFE), reaction vessel size (2–5 mL) were kept constant for the scope evaluation. For reactions that required heating, a hotplate was used with the appropriate DrySyn aluminium block.

Flash chromatography was performed using 230–400 mesh silica, with the indicated solvent system according to standard techniques. Analytical thin-layer chromatography (TLC) was performed on precoated glass-backed silica gel plates. Visualization of the developed chromatogram was performed by UV absorbance (254 nm) and stained with aqueous potassium permanganate solution, a ninhydrin solution in ethanol or a phosphomolybdic acid solution in ethanol.

Infrared spectra (ν_{max}, FTIR ATR) were recorded in reciprocal centimeters (cm^{-1}).

Nuclear magnetic resonance spectra were recorded on 400 or 500 MHz spectrometers. Chemical shifts for ^{1}H NMR spectra are recorded in parts per million from tetramethylsilane with the solvent resonance as the internal standard (chloroform: δ = 7.27 ppm, DMSO: δ = 2.50 ppm, CD_3OD: δ = 3.35 ppm, CF_3CO_2D: δ = 11.5 ppm). Data is reported as follows: chemical shift (multiplicity [s = singlet, d = doublet, t = triplet, q = quartet, p = pentet, m = multiplet and br = broad], coupling constant (in Hz), integration and assignment). ^{13}C NMR spectra were recorded with complete proton decoupling. Chemical shifts are reported in parts per million from tetramethylsilane with the solvent resonance as the internal standard ($^{13}CDCl_3$: δ = 77.0 ppm, $(^{13}CD_3)_2SO$: δ = 39.5 ppm, $^{13}CD_3OD$: δ = 49.0 ppm, $^{13}CF_3{}^{13}CO_2D$: δ = 164.2 ppm). Assignments of ^{1}H and ^{13}C spectra were based upon the analysis of δ and *J* values, as well as HSQC and HMBC experiments where appropriate.

All *tert*-butyloxycarbonyl (Boc) containing compounds appeared as a mixture of rotamers in the NMR spectra at rt. In most cases, NMR spectra at room temperature are reported. In some cases, it was necessary to carry out NMR experiments at 373 K in order to assign the signals.

Melting points are uncorrected.

The high-resolution mass spectrometry (HRMS) analyses were performed using electrospray ion source (ESI) or pneumatically assisted atmospheric pressure chemical ionization (APCI) using an atmospheric solids analysis probe (ASAP). ESI was performed using a Waters LCT Premier equipped with an ESI source operated in positive or negative ion mode. The software used was MassLynx 4.1. This software does not account for the electron and all the calibrations/references are calculated accordingly, i.e. $[M+H]^+$ is detected and the mass is calibrated to output [M+H]. APCI was performed using an Orbitrap XL or Xevo G2S using an ASAP to insert samples into the APCI source. The sample was introduced at ambient temperature and the temperature increased until the sample vaporised.

Crystals for single crystal X-ray spectroscopy were grown using slow evaporation from diethyl ether (Et_2O) for cyclized products. Crystal for **Cat7** was grown using slow evaporation from methanol (MeOH).

Reagents
Where the synthesis of a reagent is not stated, the reagent was commercially available.
Commercial reagents were used as supplied or purified by standard techniques where necessary.
Catalyst purity: Bis[rhodium(α,α,α′,α′-tetramethyl-1,3-benzenedipropionic acid)] 96% (Aldrich 622623).

Notes:

- *Although we have not experienced any problems in the handling of azides or diazo reagents, extreme care should be taken when manipulating them due to their potentially explosive nature.*[1]
- *Melting points were not recorded for diazo compounds.*
- *For the diazo compounds synthesized, the resonance for the fully substituted C=N=N carbon in the ^{13}C NMR spectrum may not be seen due to quadrupole coupling to ^{14}N. Unless the ^{13}C resonance was observed it is not reported.*

Structures of Additional Compounds in SI

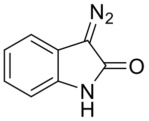
S1

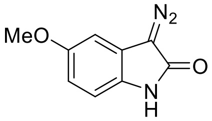
S2

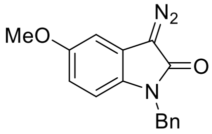
S3

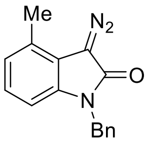
S4

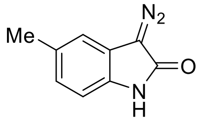
S5

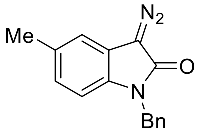
S6

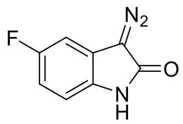
S7

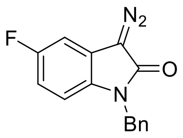
S8

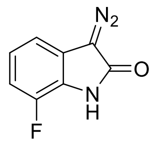
S9

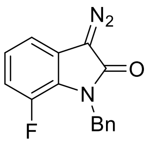
S10

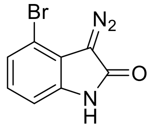
S11

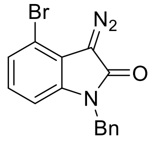
S12

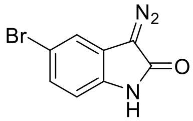
S13

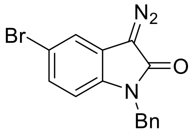
S14

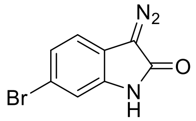
S15

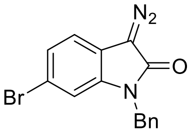
S16

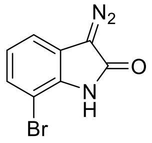
S17

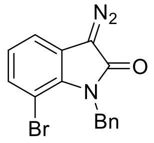
S18

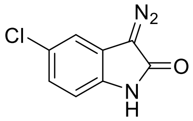
S19

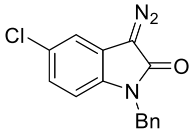
S20

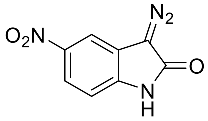
S21

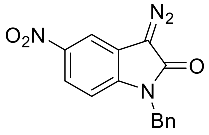
S22

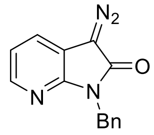
S23

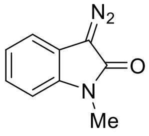
S24

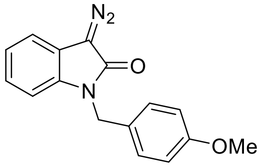
S25

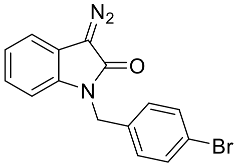
S26

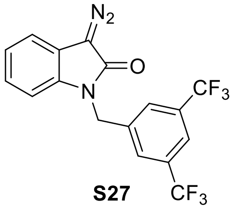
S27

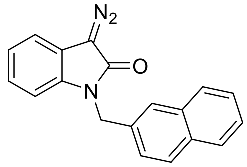
S28

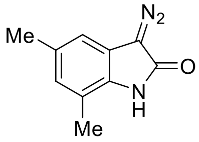
S29

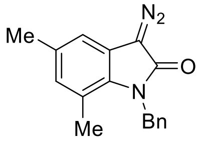
S30

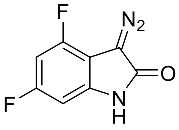
S31

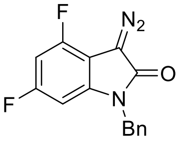
S32

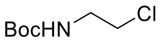
S33

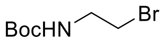
S34

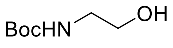
S35

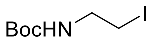
S36

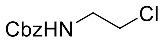
S37

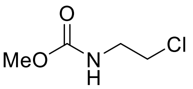
S34

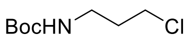
S35

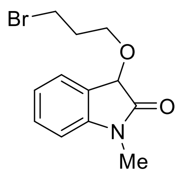
S40

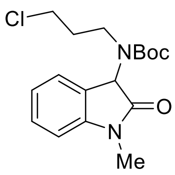
S41

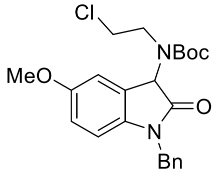
S42

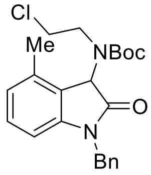
S43

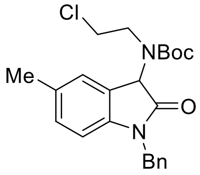
S44

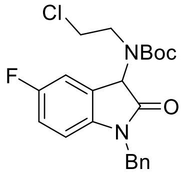
S45

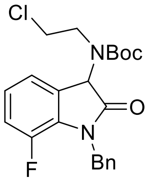
S46

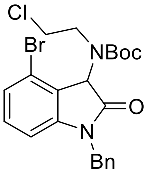
S47

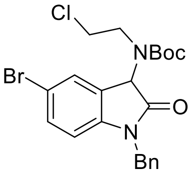
S48

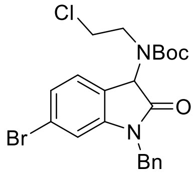
S49

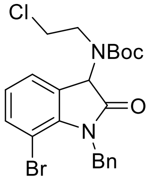
S50

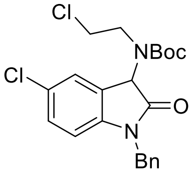
S51

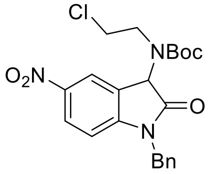
S52

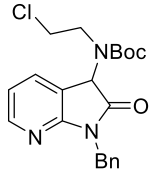
S53

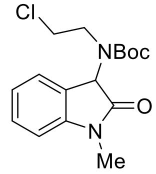
S54

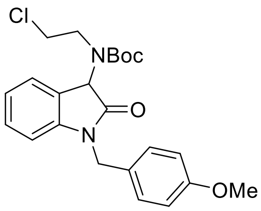
S55

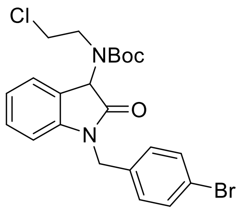
S56

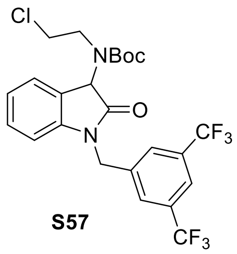
S57

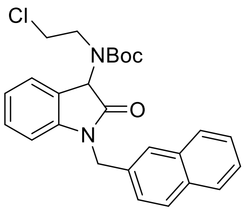
S58

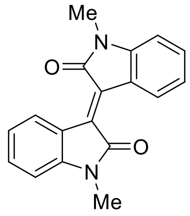
S59

Preliminary Studies on O-H Insertion Reaction

Initially, the O-H insertion reaction was studied using **S24** and -bromopropanol as a readily available cheap reagent which would serve as a suitable surrogate for *N*-Boc protected haloamine. Additionally, the O-H insertion products would be without rotameric signals, so simplifying analysis. An initial catalyst screen showed that Rh catalysts were effective for this transformation (Table **1**, Entries **1-4**) with AgOTf giving similar yield but more side-products (Table **1**, Entry **11**). Cu, Ru, Fe and Au catalysts were not successful giving little or no product. Interestingly, dimerization of diazo **S24** gave side product **S59** as a side product exclusively using Cu catalyst (Table **1**, Entry **7**).

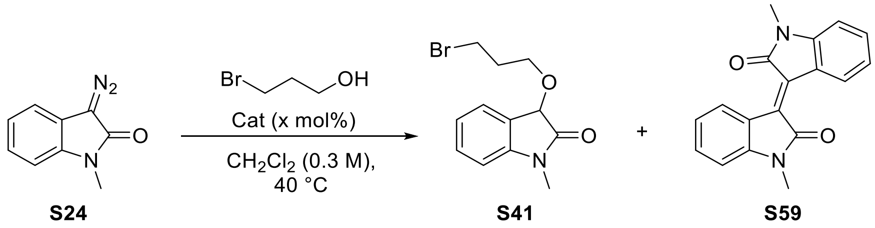

Entry	Equiv of S24	Catalyst	Time/ h	Yield (%)[a]		
				S24	S41	S59
1	0.67	$Rh_2(OAc)_4$ (0.5 mol%)	2	0	(25)	-
2	1.5	$Rh_2(OAc)_4$ (0.5 mol%)	0.3	0	27	-
3	1.5	$Rh_2(OAc)_4$ (0.5 mol%)	0.3	0	22	-
4	1.5	$Rh_2(oct)_4$ (0.5 mol%)	0.3	0	23	-
5	1.5	$[RuCp^*(MeCN)_3]PF_6$ + 1,10-phen (1 mol%)	40	0	trace	14
6	1.5	$Cu(OTf)_2$ (5 mol%)	20	0	7	44
7	1.5	$[Cu(MeCN)_4]PF_6$ (5 mol%)	20	0	0	68 (62)
8	1.5	$Fe(acac)_3$ (5 mol%)	18	45	trace	-
9	1.5	CuCl (5 mol%)	18	83	trace	6
10	1.5	PPh_3AuCl (5 mol%)	18	96	trace	8
11	1.5	AgOTf[b] (5 mol%)	18	0	21[c]	-

Table 1: Catalyst screen for O-H insertion of diazo **S24**. Isolated yields in parentheses. [a] Yields are determined by in situ ^{1}H NMR spectroscopy with respect to 1,3,5-trimethoxybenzene as internal standard. [b] Addition over 10 minutes. [c] With formation of an additional side product.

Subsequent optimisation of the O-H insertion reaction conditions including solvent, reaction time, temperature, reagent stoichiometries and diazo addition time gave up to 62% yield of the desired product **S41**:

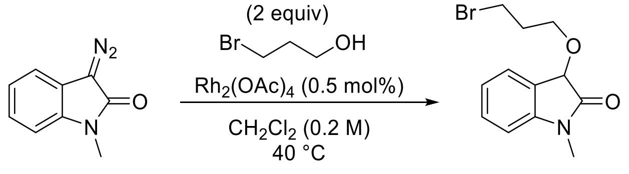

S24
(*addition over 10 min*)

S41, 62%

Optimisation of N-H Insertion Reaction

At this stage as the yield of the O-H insertion reaction was not changing significantly upon changing the variables. Instead, we switched substrate to apply the conditions to the synthesis of **S42** using protected amine **S39**. Increasing the equivalents of **S39** from 1 equiv to 2 equiv had a beneficial effect (Table **2**, Entry **2**). Increasing the catalyst loading had a negative effect (Table **2**, Entries **6-7**). It was found that $PhCH_3$ gave a similar yield to CH_2Cl_2 (Table **2**, Entry **13**).

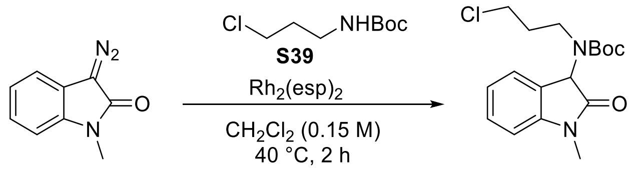

Exp EN09954-	Equiv of S39	Cat. loading (mol%)	Solvent	Conc (M)	Yield S42/ %[a]
1	1	0.5	CH_2Cl_2	0.15	54
2	2	0.5	CH_2Cl_2	0.15	71
3	3	0.5	CH_2Cl_2	0.15	72
4	5	0.5	CH_2Cl_2	0.15	74
5	1.5	0.5	CH_2Cl_2	0.15	63
6	2	1.0	CH_2Cl_2	0.15	66
7	2	2.0	CH_2Cl_2	0.15	60
8	2	0.5	CH_2Cl_2	0.05	65
9	2	0.5	CH_2Cl_2	0.1	72
10	2	0.5	CH_2Cl_2	0.2	69
11	2	0.5	CH_2Cl_2	0.3	66
12	2	0.5	CH_2Cl_2	0.5	58
13	2	0.5	$PhCH_3$	0.1	64
14	2	0.5	DCE	0.2	60

Table 2: Test of effect of equivalents, catalyst loading, concentration and solvent for the NH insertion of diazo **S24**. Reactions on 0.25 mmol scale. [a] Yields are determined by in situ ^1H NMR spectroscopy with respect to 1,3,5-trimethoxybenzene as internal standard.

Decreasing the catalyst loading had a small beneficial effect and so 0.25 mol% of catalyst could be employed instead of 0.5 mol% (Table **3**, Entry **2**). Further screening of catalysts and temperature gave a high yield in $PhCH_3$ (Table **3,** Entry **5**). Testing different catalysts showed $Rh_2(esp)_2$ was superior for this NH insertion reaction (Table **3**, Entries 9 and **12-14**).

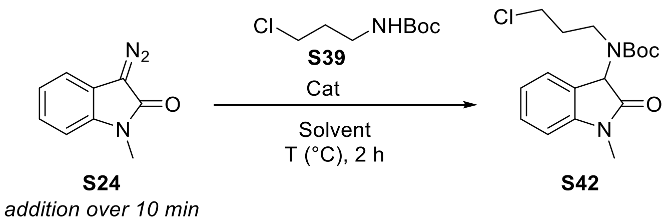

Exp	Solvent	Cat	Cat loading (mol%)	T (ºC)	Yield S42/ %[a]
1	CH_2Cl_2	$Rh_2(esp)_2$	0.5	40	72
2	CH_2Cl_2	$Rh_2(esp)_2$	**0.25**	40	75
3	CH_2Cl_2	$Rh_2(esp)_2$	0.1	40	72
4	CH_2Cl_2	$Rh_2(esp)_2$	0.25	25	73
5	**$PhCH_3$**	$Rh_2(esp)_2$	0.25	25	85
6	$PhCH_3$	$Rh_2(esp)_2$	0.25	40	75
7	$PhCH_3$	$Rh_2(esp)_2$	0.25	60	76
8	$PhCH_3$	$Rh_2(esp)_2$	0.25	80	65
9	$PhCH_3$	**$Rh_2(esp)_2$**	0.25	**25**	85
10	$PhCH_3$	$Rh_2(esp)_2$	0.25	0	61
11	CH_2Cl_2	$Rh_2(esp)_2$	0.25	0	57
12	$PhCH_3$	$Rh_2(oct)_4$	0.25	25	55
13	$PhCH_3$	$Rh_2(OAc)_4$	0.25	25	21
14	$PhCH_3$	$Rh_2(TFA)_4$	0.25	25	19

Table 3: Effect of catalyst loading, temperature and catalyst on NH insertion. Reactions on 0.25 mmol scale. [a] Yields are determined by in situ ^{1}H NMR spectroscopy with respect to 1,3,5-trimethoxybenzene as internal standard.

The effect of the slow addition was analysed and this showed that a 10 minute addition was beneficial over no addition and even longer addition times (Table **4**). These optimised reaction conditions were then applied to the synthesis of NH insertion products on >1 mmol scale with a tolerable drop in yield upon scale up (avg. yields ~60%)

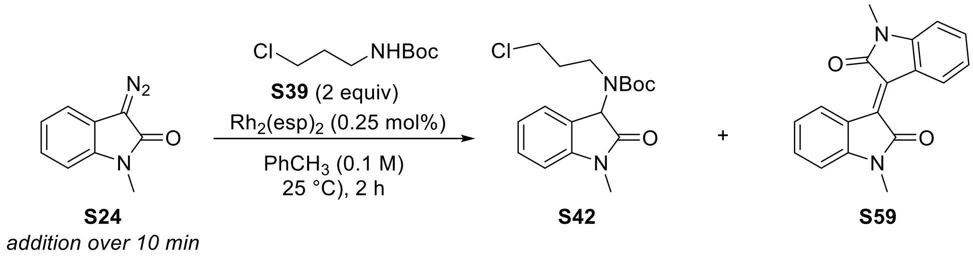

Exp	Addition time (min)	Yield 42/ %[a]	Yield S59/ %[a]	Total Yield
1	60	78	0	78
2	30	78	0	78
3	10	89	0	89
4	2	63	6	69
5	none	56	13	69

Table 4: Effect of addition time on the NH insertion of **S24**. Reactions on 0.25 mmol scale. [a] Yields are determined by in situ ^{1}H NMR spectroscopy with respect to 1,3,5-trimethoxybenzene as internal standard.

Extended Optimisation of Enantioselective Cyclisation

Catalysts used in Optimisation

CD

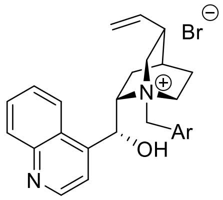

Ar

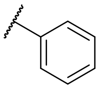

CD1

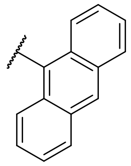

CD2

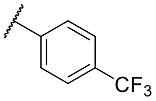

CD3 (Cat6)

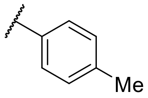

CD4

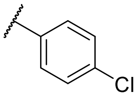

CD5

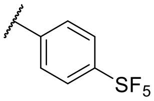

CD6 (Cat7)

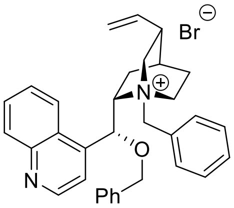

CD1A

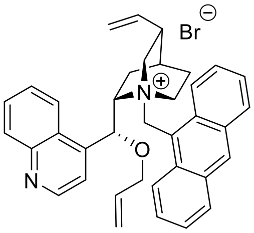

CD2A

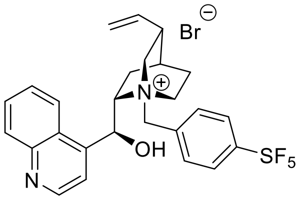

Cat8

QN

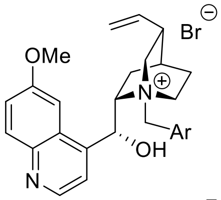

Ar

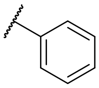

QN1

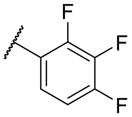

QN2

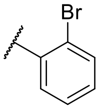

QN3

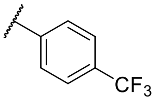

QN4 (Cat1)

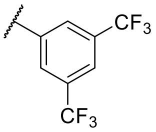

QN5

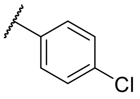

QN6

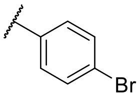

QN7

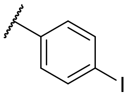

QN8

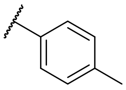

QN9

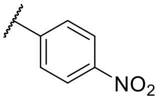

QN10

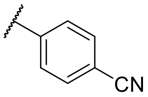

QN11

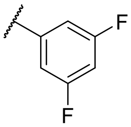

QN12

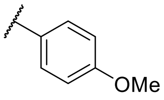

QN13

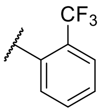

QN14

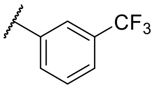

QN15

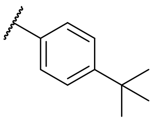

QN16

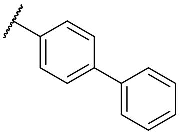

QN17

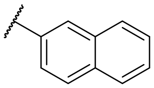

QN18

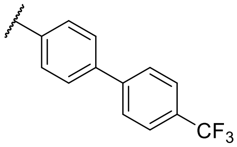

QN19

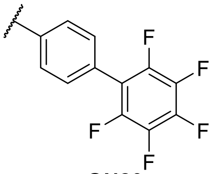

QN20

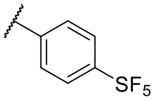

QN21

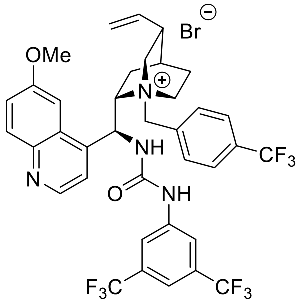

QN4A

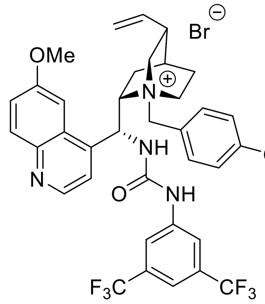

QN4A'

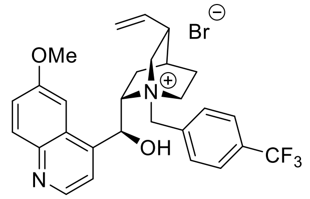

QN4'

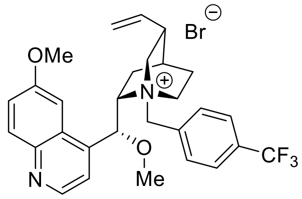

QN4B (Cat2)

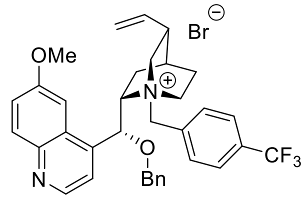

QN4C

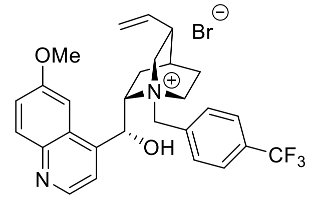

QN4D

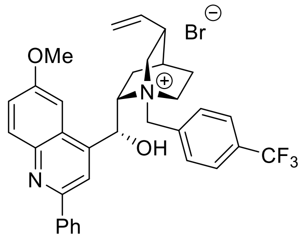

QN4E (Cat4)

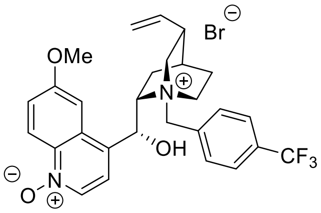

QN4F

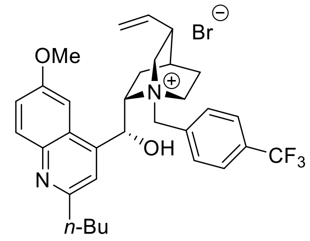

QN4G (Cat5)

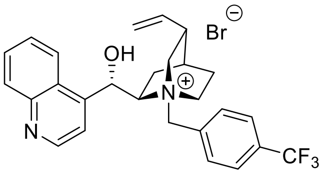

CN1

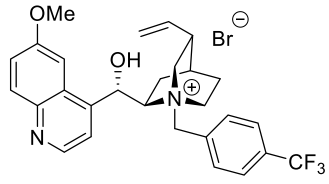

QD1

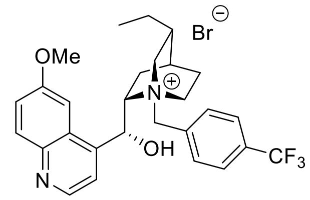

HQN1 (Cat3)

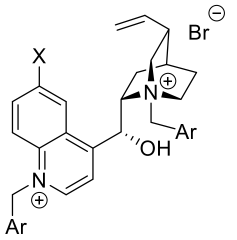

DQ

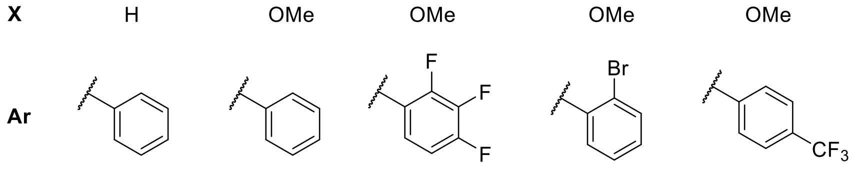

DQ1 **DQ2** **DQ3** **DQ4** **DQ5**

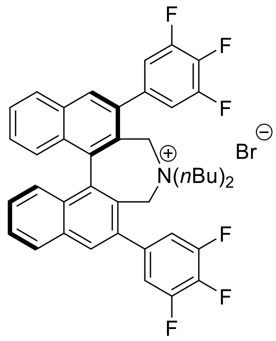

M1

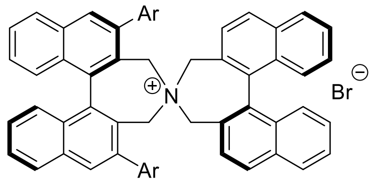

M2
Ar = 3,4,5-$F_3C_6H_2$

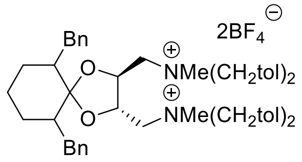

Sh1

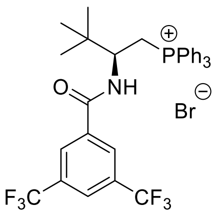

P1

Enantioselective Cyclisation - Summary of key findings

The following contains a discussion of key findings obtained during optimisation of the enantioselective cyclisation. The full optimisation table, to which the entries refer, is provided below (Table **5**).

Effect of Base

The base used had a critical effect on the outcome of the reaction. Hydroxide bases were superior in terms of yield and enantioselectivity compared to carbonate or phosphate bases (Entries **1-10 and 85-93**). When examining hydroxide bases as 50% aqueous solutions, NaOH gave the highest enantioselectivity with LiOH and KOH giving similar results. While CsOH gave a slightly higher yield but lower enantioselectivity. These trends are likely explained by the effect of water (see below). A crucial realisation of the optimisation was that use of solid hydroxide bases gave markedly different results. Solid CsOH gave very high yield and enantioselectivity while KOH gave lower yild and enantioselectivity and then NaOH gave even lower with solid LiOH giving no conversion. Other Cs bases such as Cs_2CO_3 gave much lower enantioselectivity. The amount of base was important with lower loadings giving lower enantioselectivity and higher loadings giving no benefit (Entries **100 and 121**).

Effect of Water

As discussed above a crucial realisation of the optimisation was the effect of water. The lower enantioselectivity of CsOH as a 50% aq. solution compared to KOH or NaOH was likely due to the amount of water (50% w/v) (Entries **85-91**). Using solid CsOH gave very high yield and enantioselectivity, indicating the presence of water significantly reduced both metrics. Doping the reaction with water (Entries **115-118**) showed that with small amounts the reaction gave similar yields and enantioselectivity but with more water there was a dramatic impact in rate of reaction and enantioselectivity. Of note is that water is tolerated in the reaction because the CsOH used was the monohydrate. Oven-drying the base gave significantly reduced yield with the same enantioselectivity (Entry **130**). Using the same oven-dried base but grinding the base prior to addition gave a relatively higher yield with unchanged selectivity (Entry **131**). This shows that the presence of water in the form of the monohydrate of CsOH is crucial to the reaction yield. This may be due to the effect this has on the surface area/dispersion of the base. The nature of this effect is complicated by the number of factors involved i.e. contribution to base composition/surface area, possible solvation effect of catalyst/interaction within transition state as well as influencing the dielectric constant of the reaction medium. Addition of small amounts of polar solvents (Entries **102-103 and 124-126**) to test the effect on the dielectric constant, all led to lower yield and generally lower enantioselectivity, although DMF had no effect on the enantioselectivity.

Catalyst Loading

Catalyst loading in organocatalysis is a common problem, with high catalyst loadings often required. 20 mol% of catalyst was chosen as a standard with a higher and lower catalyst loading being tested. Lower catalyst loading led to significantly reduced yield and lower enantioselectivity, likely due to greater substrate degradation due to less catalyst available for substrate activation (Entry **123**). Higher catalyst loading gave a small boost in yield but no benefit for the enantioselectivity (Entry **120**).

Solvent

Two extensive solvent screens were carried out in the optimisation (Entries **56-64** and **104-111**). These identified that non-polar aromatic solvents were superior particularly toluene or xylenes. Polar or fluorinated aromatic solvents gave lower yield and enantioselectivity. There are a number of asymmetric PTC methodologies that employ polar solvents, however, solvents such as CH_2Cl_2, Et_2O and EtOAc gave poor enantioselectivities. *m*-Xylene or *o*-xylene gave very high yield and enantioselectivity.

Substrate Protecting group (Pg)

Protecting group (Pg) was modified by changing the carbamate group to a Cbz group or methoxycarbamate (Entry **49-50**). These groups were chosen to not alter significantly from the optimised NH insertion chemistry and as they could be feasibly removed. These groups gave lower yield and enantioselectivity, likely due to the smaller group being a poorer steric shield compared to the presumably easy to remove Boc group.

Substrate R group

The R group was changed for different alkyl groups. The benzyl group consistently gave high yield and enantioselectivity compared to other groups i.e. napthyl or anthracenyl groups (Entries **52-53**). The smaller methyl group gave lower enantioselectivity (Entry **51**). Altering the electronics of the benzyl group (Entries **54-55**) had small effects on the yield and small reduction in enantioselectivity.

Leaving Group

At an early stage of the optimisation the Cl leaving group was established to give higher enantioselectivity than Br or I (Entries **46-48**). The higher yields observed with Br or I are expected due to the better nucleofuge ability and hence lower barrier to cyclisation, potentially resulting in greater background reaction. Additionally, lower enantioselectivities can be explained by a larger leaving group reducing catalyst-substrate interactions and having poorer electrostatic interaction with the quaternised nitrogen.

Catalyst Structure

Quaternary ammonium salts derived from *cinchona* alkaloids proved to give the highest enantioselectivity. A benzyl substituent with *para*-substitution was favoured (Entries **22-45**) with highest enantioselectivity observed for electron-withdrawing substituents (Entries **24-31**), though NO_2 and CN did not give as high enantioselectivity (Entries **30-31**). Commercial catalysts (Entries **15, 65-67**) gave poor yield and enantioselectivity with both NaOH (50% aq.) and CsOH (s). Protection of the hydroxyl group of the catalyst either with a Me or Bn group reduced enantioselectivity and yield (Entry **72-73**). This suggests that the OH group is important for substrate interaction i.e. by hydrogen bonding. Dixon has developed urea containing phase transfer catalysts to try to improve hydrogen bonding.[2] Using both epimers of this type of catalyst gave poor yield and enantioselectivity (Entries **70-71**). Substitution at the 2-position of the quinoline gave no benefit to yield or enantioselectivity (Entries **77**). Oxidation of the quinoline to the N-oxide improved the yield but gave poor enantioselecitivity (Entry **78**).[3] With solid CsOH as base cinchonidine catalysts including commercial catalyst **CD1** gave improved enantioselectivity (Entries **132-143**) with high enantioselectivity being achieved with **CD4** and **CD6**, with highly electron withdrawing fluorinated substituents. The pseudoenantiomeric *cinchona* alkaloids quinidine or cinchonine gave the reversed enantioselectivity, albeit, with a slight reduction in enantioselectivity.

Enantioselective Cyclisation - Full Optimisation Table

The full optimisation table for the optimisation of the enantioselective cyclisation can be found below for the following general reaction (key changes are highlighted in bold, green highlights indicates the best result in each round of optimisation). All of the yields have been calculated by ^{1}H NMR spectroscopy with respect to 1,3,5-trimethoxybenzene as an internal standard and have been checked so that they have been integrated uniformly (this may result in some changes from previous reports to correct mistakes). A narrative of this table is given below.

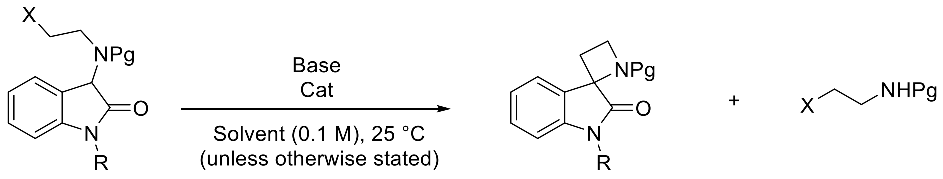

2a-e **3a-e** **HA**

Initial results used 2 equiv of KOH, either solid or 50% aq., and compared the results with and without a simple, commercial phase transfer catalyst. Results showed low (but non zero) enantioselectivity with higher yield and lower background reaction observed for the reaction with 50% KOH.

Exp	Cat	(mol%)	Base	R	Pg	X	Solvent	additive/ condition	t (h)	Conv	Yield		3 *er*
											3	HA	
1	none	-	KOH (**s**, 2 equiv)	Bn	Boc	Cl	$PhCH_3$	-	20	100	35	39	na
2	CD1	**10**	KOH (s, 2 equiv)	Bn	Boc	Cl	$PhCH_3$	-	20	100	41	29	42:58
3	none	-	KOH (**50% aq**, 2 equiv)	Bn	Boc	Cl	$PhCH_3$	-	20	32	15	17	na
4	CD1	**10**	KOH (50% aq, 2 equiv)	Bn	Boc	Cl	$PhCH_3$	-	20	100	77	23	42:57

A screen of bases (at 5 equiv of base) showed that NaOH gave the highest enantioselectivity and yield (Entry **7**). Hydroxide bases were superior to carbonate bases with respect to reaction yield (Entries **9-10**).

Exp	Cat	(mol%)	Base	R	Pg	X	Solvent	additive/ condition	t (h)	Conv	Yield		3 *er*
											3	HA	
5	CD1	10	KOH (50% aq, **5 equiv**)	Bn	Boc	Cl	$PhCH_3$	-	20	37	23	14	42:58
6	CD1	10	KOH (50% aq, 5 equiv)	Bn	Boc	Cl	$PhCH_3$	**20% H_2O**	20	41	26	15	41:59
7	CD1	10	**NaOH** (50% aq, 5 equiv)	Bn	Boc	Cl	$PhCH_3$	-	20	100	50	38	35:65
8	CD1	10	**CsOH** (50% aq, 5 equiv)	Bn	Boc	Cl	$PhCH_3$	-	20	38	22	16	40:60

9	CD1	10	**Cs_2CO_3** (50% aq, 5 equiv)	Bn	Boc	Cl	$PhCH_3$	-	20	6	2	4	40:60
10	CD1	10	**K_2CO_3** (50% aq, 5 equiv)	Bn	Boc	Cl	$PhCH_3$	-	20	14	4	9	40:60

A catalyst screen showed that quininium based catalyst **QN1** (Entry **13**) gave a similar yield and enantioselectivity and this was more reliable.

Exp	Cat	(mol%)	Base	R	Pg	X	Solvent	additive/ condition	t (h)	Conv	Yield		3 *er*
											3	HA	
11	**CD2**	10	NaOH (50% aq, 5 equiv)	Bn	Boc	Cl	$PhCH_3$	-	20	100	20	46	37:63
12	**CD2A**	10	NaOH (50% aq, 5 equiv)	Bn	Boc	Cl	$PhCH_3$	-	20	100	12	60	62:37
13	**QN1**	10	NaOH (50% aq, 5 equiv)	Bn	Boc	Cl	$PhCH_3$	-	20	100	50	49	35:65
14	**CD1A**	10	NaOH (50% aq, 5 equiv)	Bn	Boc	Cl	$PhCH_3$	-	20	100	18	54	53:47
15	**M1**	10	NaOH (50% aq, 5 equiv)	Bn	Boc	Cl	$PhCH_3$	-	20	100	10	55	52:48
16	**DQ1**	10	NaOH (50% aq, 5 equiv)	Bn	Boc	Cl	$PhCH_3$	-	20	100	32	52	36:64
17	**CD1**	10	NaOH (50% aq, 5 equiv)	Bn	Boc	Cl	$PhCH_3$	0 ºC	20	100	9	60	46:54

A screen of quininium salts showed that a 4-CF_3 benzyl substituent **QN4** gave higher enantioselectivity.

Exp	Cat	(mol%)	Base	R	Pg	X	Solvent	additive/ condition	t (h)	Conv	Yield		3 *er*
											3	HA	
18	**DQ2**	10	NaOH (50% aq, 5 equiv)	Bn	Boc	Cl	$PhCH_3$	-	20	100	33	58	49:51
19	**DQ3**	10	NaOH (50% aq, 5 equiv)	Bn	Boc	Cl	$PhCH_3$	-	20	87	15	43	54:46
20	**DQ4**	10	NaOH (50% aq, 5 equiv)	Bn	Boc	Cl	$PhCH_3$	-	20	83	17	34	51:49
21	**DQ5**	10	NaOH (50% aq, 5 equiv)	Bn	Boc	Cl	$PhCH_3$	-	20	90	29	61	37:63

Exp	Cat	(mol%)	Base	R	Pg	X	Solvent	additive/ condition	t (h)	Conv	Yield		3 *er*
											3	HA	
22	**QN1**	10	NaOH (50% aq, 5 equiv)	Bn	Boc	Cl	$PhCH_3$	-	20	100	32	60	33:67
23	**QN2**	10	NaOH (50% aq, 5 equiv)	Bn	Boc	Cl	$PhCH_3$	-	20	100	15	75	49:51
24	**QN3**	10	NaOH (50% aq, 5 equiv)	Bn	Boc	Cl	$PhCH_3$	-	20	100	19	77	56:44
24	**QN4**	10	NaOH (50% aq, 5 equiv)	Bn	Boc	Cl	$PhCH_3$	-	20	100	48	49	21:79
25	**QN5**	10	NaOH (50% aq, 5 equiv)	Bn	Boc	Cl	$PhCH_3$	-	20	100	19	45	56:44
26	**QN6**	10	NaOH (50% aq, 5 equiv)	Bn	Boc	Cl	$PhCH_3$	-	20	100	27	24	26:74
27	**QN7**	10	NaOH (50% aq, 5 equiv)	Bn	Boc	Cl	$PhCH_3$	-	20	100	50	34	27:73
28	**QN8**	10	NaOH (50% aq, 5 equiv)	Bn	Boc	Cl	$PhCH_3$	-	20	100	48	47	27:73
29	**QN9**	10	NaOH (50% aq, 5 equiv)	Bn	Boc	Cl	$PhCH_3$	-	20	100	40	54	31:69
30	**QN10**	10	NaOH (50% aq, 5 equiv)	Bn	Boc	Cl	$PhCH_3$	-	20	100	64	45	39:61
31	**QN11**	10	NaOH (50% aq, 5 equiv)	Bn	Boc	Cl	$PhCH_3$	-	20	100	59	36	37:63
32	**QN12**	10	NaOH (50% aq, 5 equiv)	Bn	Boc	Cl	$PhCH_3$	-	20	100	14	46	48:52
33	**QN13**	10	NaOH (50% aq, 5 equiv)	Bn	Boc	Cl	$PhCH_3$	-	20	100	28	55	41:59
34	**QN14**	20	NaOH (50% aq, 5 equiv)	Bn	Boc	Cl	$PhCH_3$	-	20	100	23	75	59:41
35	**QN15**	20	NaOH (50% aq, 5 equiv)	Bn	Boc	Cl	$PhCH_3$	-	20	100	9	15	42:58
36	**QN16**	20	NaOH (50% aq, 5 equiv)	Bn	Boc	Cl	$PhCH_3$	-	20	100	48	45	32:68

37	**QN17**	20	NaOH (50% aq, 5 equiv)	Bn	Boc	Cl	$PhCH_3$	-	20	100	36	35	32:68
38	**QN18**	20	NaOH (50% aq, 5 equiv)	Bn	Boc	Cl	$PhCH_3$	-	20	100	23	52	34:66
39	**None**	-	NaOH (50% aq, 5 equiv)	Bn	Boc	Cl	$PhCH_3$	-	20	100	10	41	51:49
40	QN4	**10**	NaOH (50% aq, 5 equiv)	Bn	Boc	Cl	$PhCH_3$	-	20	100	24	37	23:77
41	QN4	**20**	NaOH (50% aq, 5 equiv)	Bn	Boc	Cl	$PhCH_3$	-	20	100	48	41	23:77
42	**QD1**	20	NaOH (50% aq, 5 equiv)	Bn	Boc	Cl	$PhCH_3$	-	20	100	51	23	79:21
43	**CD3**	20	NaOH (50% aq, 5 equiv)	Bn	Boc	Cl	$PhCH_3$	-	20	100	22	10	33:67
44	**CN1**	20	NaOH (50% aq, 5 equiv)	Bn	Boc	Cl	$PhCH_3$	-	20	100	27	29	50:50
45	**QN4***	20	NaOH (50% aq, 5 equiv)	Bn	Boc	Cl	$PhCH_3$	-	20	100	62	37	23:77

Screening for structural changes in the substrate showed that Bn, Boc and Cl were the best substituents to use (Entries **46**, **54-55**).

Exp	Cat	(mol%)	Base	R	Pg	X	Solvent	additive/ condition	t (h)	Conv	Yield		3 *er*
											3	HA	
46	QN4	20	NaOH (50% aq, 5 equiv)	Bn	Boc	**Cl**	$PhCH_3$	-	20	100	60	39	23:77
47	QN4	20	NaOH (50% aq, 5 equiv)	Bn	Boc	**Br**	$PhCH_3$	-	20	100	81	0	41:59
48	QN4	20	NaOH (50% aq, 5 equiv)	Bn	Boc	**I**	$PhCH_3$	-	20	100	83	0	57:43
49	QN4	20	NaOH (50% aq, 5 equiv)	Bn	**Cbz**	Cl	$PhCH_3$	-	20	100	33	31	62:38
50	QN4	20	NaOH (50% aq, 5 equiv)	Bn	**CO_2Me**	Cl	$PhCH_3$	-	20	100	45	51	57:43
51	QN4	20	NaOH (50% aq, 5 equiv)	**Me**	Boc	Cl	$PhCH_3$	-	20	100	66	52	71:29

52	QN4	20	NaOH (50% aq, 5 equiv)	**2-Menapthyl**	Boc	Cl	$PhCH_3$	-	20	100	39	72	21:79
53	QN4	20	NaOH (50% aq, 5 equiv)	**9-Meanthryl**	Boc	Cl	$PhCH_3$	-	20	100	37	61	50:50
54	QN4	20	NaOH (50% aq, 5 equiv)	**4-CF_3Bn**	Boc	Cl	$PhCH_3$	-	20	100	56	44	25:75
55	QN4	20	NaOH (50% aq, 5 equiv)	**4-OMeBn**	Boc	Cl	$PhCH_3$	-	20	100	59	35	72:28

A solvent screen showed that non-polar aromatic solvents (toluene, xylenes) were the best with no other solvents giving an advantage in terms of yield or enantioselectivity (Entries **56 and 62**).

Exp	Cat	(mol%)	Base	R	Pg	X	Solvent	additive/ condition	t (h)	Conv	Yield		3 *er*
											3	HA	
56	QN4	20	NaOH (50% aq, 5 equiv)	Bn	Boc	Cl	**$PhCH_3$**	-	20	100	43	43	25:75
57	QN4	20	NaOH (50% aq, 5 equiv)	Bn	Boc	Cl	**$PhCF_3$**	-	20	100	29	51	38:62
58	QN4	20	NaOH (50% aq, 5 equiv)	Bn	Boc	Cl	**PhH**	-	20	100	28	23	26:74
59	QN4	20	NaOH (50% aq, 5 equiv)	Bn	Boc	Cl	**CH_2Cl_2**	-	20	100	43	44	46:54
60	QN4	20	NaOH (50% aq, 5 equiv)	Bn	Boc	Cl	**EtOAc**	-	20	100	trace	28	50:50
61	QN4	20	NaOH (50% aq, 5 equiv)	Bn	Boc	Cl	**PhOMe**	-	20	100	13	44	33:67
62	QN4	20	NaOH (50% aq, 5 equiv)	Bn	Boc	Cl	**Xylenes**	-	20	100	59	36	24:76
63	QN4	20	NaOH (50% aq, 5 equiv)	Bn	Boc	Cl	**Mesitylene**	-	20	100	*nd**	36	28:72
64	QN4	20	NaOH (50% aq, 5 equiv)	Bn	Boc	Cl	**1,2-$F_2C_6H_4$**	-	20	100	26	74	48:52

A screen of different catalysts, including some commercial catalysts (Entries **65-67**), and structural modifications to the quinine scaffold resulted in no improvements in enantioselectivity (Entries **69-78**).

Exp	Cat	(mol%)	Base	R	Pg	X	Solvent	additive/ condition	t (h)	Conv	Yield		3 *er*
											3	HA	
65	**M2**	10	NaOH (50% aq, 5 equiv)	Bn	Boc	Cl	$PhCH_3$	-	20	100	22	51	31:69
66	**Sh1**	10	NaOH (50% aq, 5 equiv)	Bn	Boc	Cl	$PhCH_3$	-	20	100	41	42	36:64
67	**P1**	10	NaOH (50% aq, 5 equiv)	Bn	Boc	Cl	$PhCH_3$	-	20	100	20	69	62:38
68	**QN4**	20	NaOH (50% aq, 5 equiv)	Bn	Boc	Cl	$PhCH_3$	-	20	100	72	28	23:77
69	**QN4'**	20	NaOH (50% aq, 5 equiv)	Bn	Boc	Cl	$PhCH_3$	-	20	100	37	33	40:60
70	**QN4A'**	20	NaOH (50% aq, 5 equiv)	Bn	Boc	Cl	$PhCH_3$	-	20	100	15	65	57:43
71	**QN4A**	20	NaOH (50% aq, 5 equiv)	Bn	Boc	Cl	$PhCH_3$	-	20	100	11	48	55:45
72	**QN4B**	20	NaOH (50% aq, 5 equiv)	Bn	Boc	Cl	$PhCH_3$	-	20	100	53	30	30:70
73	**QN4C**	20	NaOH (50% aq, 5 equiv)	Bn	Boc	Cl	$PhCH_3$	-	20	100	31	43	30:70
74	**QN19**	20	NaOH (50% aq, 5 equiv)	Bn	Boc	Cl	$PhCH_3$	-	20	100	74	26	25:75
75	**QN20**	20	NaOH (50% aq, 5 equiv)	Bn	Boc	Cl	$PhCH_3$	-	20	100	38	32	27:73
76	**QN4D**	20	NaOH (50% aq, 5 equiv)	Bn	Boc	Cl	$PhCH_3$	-	20	100	45	30	50:50
77	**QN4E**	20	NaOH (50% aq, 5 equiv)	Bn	Boc	Cl	$PhCH_3$	-	20	100	45	30	23:77
78	**QN4F**	20	NaOH (50% aq, 5 equiv)	Bn	Boc	Cl	$PhCH_3$	-	20	100	79	21	43:57

Different additives, concentrations and temperature were examined with none showing a significant benefit (Entries **79-84**).

Exp	Cat	(mol%)	Base	R	Pg	X	Solvent	additive/ condition	t (h)	Conv	Yield		**3** *er*
											**3**	**HA**	
79	QN4	20	NaOH (50% aq, 5 equiv)	Bn	Boc	Cl	$PhCH_3$	**-**	20	100	62	33	25:75
80	QN4	20	NaOH (50% aq, 5 equiv)	Bn	Boc	Cl	$PhCH_3$	**40 ºC**	20	100	99	-	30:70
81	QN4	20	NaOH (50% aq, 5 equiv)	Bn	Boc	Cl	$PhCH_3$	**0.2 M**	20	100	50	31	29:71
82	QN4	20	NaOH (50% aq, 5 equiv)	Bn	Boc	Cl	$PhCH_3$	**0.05 M**	20	100	62	21	20:80
83	QN4	20	NaOH (50% aq, 5 equiv)	Bn	Boc	Cl	$PhCH_3$	**10% $CHCl_3$**	20	100	59	26	23:77
84	QN4	20	NaOH (50% aq, 5 equiv)	Bn	Boc	Cl	$PhCH_3$	**10% MTBE**	20	100	55	40	25:75

Changing base showed decreased enantioselectivity however CsOH gave higher yield - using solid CsOH gave a large increase in enantioselectivity (Entries **86 and 93**). Using KOH or NaOH gave lower yields and enantioselectivities (Entries **85 and 90-92**).

Exp	Cat	(mol%)	Base	R	Pg	X	Solvent	additive/ condition	t (h)	Conv	Yield		**3** *er*
											**3**	**HA**	
85	QN4	20	**KOH** (50% aq, 5 equiv)	Bn	Boc	Cl	$PhCH_3$	-	20	100	65	31	31:69
86	QN4	20	**CsOH** (50% aq, 5 equiv)	Bn	Boc	Cl	$PhCH_3$	-	20	100	70	30	38:62
87	QN4	20	**LiOH** (50% aq, 5 equiv)	Bn	Boc	Cl	$PhCH_3$	-	20	100	70	30	32:68
88	QN4	20	**K_3PO_4** (50% aq, 5 equiv)	Bn	Boc	Cl	$PhCH_3$	-	20	100	30	26	37:63
89	QN4	20	**LiOH** (**s**, 5 equiv)	Bn	Boc	Cl	$PhCH_3$	-	20	0	0	0	-
90	QN4	20	**NaOH** (**s**, 5 equiv)	Bn	Boc	Cl	$PhCH_3$	-	20	100	38	42	23:77
91	QN4	20	**KOH** (**s**, 5 equiv)	Bn	Boc	Cl	$PhCH_3$	-	20	100	81	0	14:86

92	QN4	20	**Na_2CO_3** (**s**, 5 equiv)	Bn	Boc	Cl	$PhCH_3$	-	20	0	0	0	-
93	QN4	20	**CsOH** (**s**, 5 equiv)	Bn	Boc	Cl	$PhCH_3$	-	20	100	85	0	8:92

Now using CsOH (s) as base, increasing or decreasing the concentration led to reduced enantioselectivity (Entries **94-99**), lowering the amount of base led to lowerinng of enantioselectivity (Entry **100**) and lowering the temperature led to poor yield and enantioselectivity (Entry **101**).

Exp	Cat	(mol%)	Base	R	Pg	X	Solvent	additive/ condition	t (h)	Conv	Yield		3 *er*
											3	HA	
94	**none**	20	CsOH (s, 5 equiv)	Bn	Boc	Cl	$PhCH_3$	-	20	100	1	0	54:46
95	**QN4**	20	CsOH (s, 5 equiv)	Bn	Boc	Cl	$PhCH_3$	-	20	100	100	0	8:92
96	QN4	20	CsOH (s, 5 equiv)	Bn	Boc	Cl	$PhCH_3$	**0.05 M**	20	100	100	0	8:92
97	QN4	20	CsOH (s, 5 equiv)	Bn	Boc	Cl	$PhCH_3$	**0.025 M**	20	100	91	0	12:88
98	QN4	20	CsOH (s, 5 equiv)	Bn	Boc	Cl	$PhCH_3$	**0.2 M**	20	100	80	0	9:91
99	QN4	20	CsOH (s, 5 equiv)	Bn	Boc	Cl	$PhCH_3$	**0.3 M**	20	100	91	0	11:89
100	QN4	20	CsOH (s, **2.5 equiv**)	Bn	Boc	Cl	$PhCH_3$	-	20	100	82	0	10:90
101	QN4	20	CsOH (s, 5 equiv)	Bn	Boc	Cl	$PhCH_3$	**0 °C**	20	87	19	36	25:75
102	QN4	20	CsOH (s, 5 equiv)	Bn	Boc	Cl	$PhCH_3$	**10% Et_2O**	20	100	82	0	10:90
103	QN4	20	CsOH (s, 5 equiv)	Bn	Boc	Cl	$PhCH_3$	**10% $CHCl_3$**	20	100	92	0	10:90

A screen of non-polar aromatic solvents showed higher enantioselectivity with *m*-xylene and *o*-xylene (Entries **107** and **108**). Further screening of other solvents confirmed that. non-polar aromatic solvents were best, with other solvents commonly seen in phase transfer catalysed reactions performing poorly (Entries **109-111**).

Exp	Cat	(mol%)	Base	R	Pg	X	Solvent	additive/ condition	t (h)	Conv	Yield		3 *er*
											3	HA	
104	QN4	20	CsOH (s, 5 equiv)	Bn	Boc	Cl	**Xylenes**	-	20	100	78	0	7:93
105	QN4	20	CsOH (s, 5 equiv)	Bn	Boc	Cl	***p*-xylene**	-	20	100	68	0	8:92
106	QN4	20	CsOH (s, 5 equiv)	Bn	Boc	Cl	***o*-xylene**	-	20	100	81	0	6:94

107	QN4	20	CsOH (s, 5 equiv)	Bn	Boc	Cl	***m*-xylene**	-	1	100	81	0	6:94
109	QN4	20	CsOH (s, 5 equiv)	Bn	Boc	Cl	**CH_2Cl_2**	-	1	100	49	13	33:67
110	QN4	20	CsOH (s, 5 equiv)	Bn	Boc	Cl	**Et_2O**	-	1	100	74	trace	37:63
111	QN4	20	CsOH (s, 5 equiv)	Bn	Boc	Cl	**PhH**	-	1	100	65	0	12:88

Carrying. out the reaction under Ar or at higher temperature led to small drops in enantioselectivity (Entries **112-114**). Doping water to the reaction had a significant detrimental effect to the rate of the reaction and enantioselectivity (Entries **116-118**).

Exp	Cat	(mol%)	Base	R	Pg	X	Solvent	additive/ condition	t (h)	Conv	Yield		3 *er*
											3	HA	
112	QN4	20	CsOH (s, 5 equiv)	Bn	Boc	Cl	*m*-xylene	**Under Ar**	1	100	100	0	7:93
113	QN4	20	CsOH (s, 5 equiv)	Bn	Boc	Cl	*m*-xylene	**40 ºC**	1	100	92	0	8:92
114	QN4	20	CsOH (s, 5 equiv)	Bn	Boc	Cl	*m*-xylene	**30 ºC**	1	100	98	0	7:93
115	QN4	20	CsOH (s, 5 equiv)	Bn	Boc	Cl	*m*-xylene	**20 ºC**	1	100	84	0	6:94
116	QN4	20	CsOH (s, 5 equiv)	Bn	Boc	Cl	*m*-xylene	**10 μL H_2O**	1	100	86	5	8:92
117	QN4	20	CsOH (s, 5 equiv)	Bn	Boc	Cl	*m*-xylene	**50 μL H_2O**	1	83	22	26	36:64
118	QN4	20	CsOH (s, 5 equiv)	Bn	Boc	Cl	*m*-xylene	**100 μL H_2O**	1	37	13	19	37:63

Increasing catalyst loading gave no improvement in yield or selectivity whereas decreasing catalyst loading led to a significant drop in yield (Entries **119-122**).

Exp	Cat	(mol%)	Base	R	Pg	X	Solvent	additive/ condition	t (h)	Conv	Yield		3 *er*
											3	HA	
119	QN4	**20**	CsOH (s, 5 equiv)	Bn	Boc	Cl	*m*-xylene	-	1	100	91	0	5:95
120	QN4	**40**	CsOH (s, 5 equiv)	Bn	Boc	Cl	*m*-xylene	-	1	100	89	0	6:94
121	QN4	**20**	**CsOH (s, 10 equiv)**	Bn	Boc	Cl	*m*-xylene	-	1	100	79	0	5:95
123	QN4	**10**	CsOH (s, 5 equiv)	Bn	Boc	Cl	*m*-xylene	-	1	100	23	trace	10:90

Additives that could affect the dielectric constant of the medium as well as Cs salts did not improve the reaction (Entries **124-129**). Drying the CsOH hasd a marked effect on the yield of the reaction but didn't affect the selectivity (Entries **130 and 131**).

Exp	Cat	(mol%)	Base	R	Pg	X	Solvent	additive/ condition	t (h)	Conv	Yield		3 *er*
											3	HA	
124	QN4	20	CsOH (s, 5 equiv)	Bn	Boc	Cl	*m*-xylene	**10 μL HFIP**	1	66	9	10	39:61
125	QN4	20	CsOH (s, 5 equiv)	Bn	Boc	Cl	*m*-xylene	**10 μL urea**	1	100	36	4	6:94
126	QN4	20	CsOH (s, 5 equiv)	Bn	Boc	Cl	*m*-xylene	**10 μL DMF**	1	100	42	0	11:89
127	QN4	20	CsOH (s, 5 equiv)	Bn	Boc	Cl	*m*-xylene	**$AgBF_4$ (1 equiv)**	1	100	Trace	26	-
128	QN4	20	**Cs_2CO_3** (s, 5 equiv)	Bn	Boc	Cl	*m*-xylene	-	23	100	71	16	23:77
129	QN4	20	CsOH (s, 5 equiv)	Bn	Boc	Cl	*m*-xylene	**CsCl** (1 equiv)	1	100	82	0	5:95
130	QN4	20	CsOH (s, 5 equiv, **oven dried)**	Bn	Boc	Cl	*m*-xylene	-	1	100	49	trace	6:94
131	QN4	20	CsOH (s, 5 equiv, **oven dried and ground**)	Bn	Boc	Cl	*m*-xylene	-	1	100	75	0	5:95

Further catalyst screening showed that removal of the OMe group of the quinoline had a positive effect on selectivity (Entries **132-143**).

Exp	Cat	(mol%)	Base	R	Pg	X	Solvent	additive/ condition	t (h)	Conv	Yield		3 *er*
											3	HA	
132	**QN4B**	20	CsOH (s, 5 equiv)	Bn	Boc	Cl	*m*-xylene	-	1	100	54	20	29:71
133	**QN4A**	20	CsOH (s, 5 equiv)	Bn	Boc	Cl	*m*-xylene	-	1	85	Trace	10	62:38*
133	**QN4E**	20	CsOH (s, 5 equiv)	Bn	Boc	Cl	*m*-xylene	-	1	100	71	8	8:92
134	**QN20**	20	CsOH (s, 5 equiv)	Bn	Boc	Cl	*m*-xylene	-	1	100	45	8	13:87
135	**QD1**	20	CsOH (s, 5 equiv)	Bn	Boc	Cl	*m*-xylene	-	1	100	80	12	90:10
136	**HQN1**	20	CsOH (s, 5 equiv)	Bn	Boc	Cl	*m*-xylene	-	1	100	85	0	10:90

Exp	Cat	(mol%)	Base	R	Pg	X	Solvent	additive/ condition	t (h)	Conv	Yield 3	Yield HA	3 *er*
137	**QN4**	20	CsOH (s, 5 equiv)	Bn	Boc	Cl	*m*-xylene	-	1	100	92	0	7:93
138	**QN21**	20	CsOH (s, 5 equiv)	Bn	Boc	Cl	*m*-xylene	-	1	100	78	0	10:90
139	**QN10**	20	CsOH (s, 5 equiv)	Bn	Boc	Cl	*m*-xylene	-	1	100	11	0	29:71
140	**QN4G**	20	CsOH (s, 5 equiv)	Bn	Boc	Cl	*m*-xylene	-	1	100	87	0	13:87
141	**M2**	20	CsOH (s, 5 equiv)	Bn	Boc	Cl	*m*-xylene	-	1	100	15	22	32:68
142	**Sh1**	20	CsOH (s, 5 equiv)	Bn	Boc	Cl	*m*-xylene	-	1	100	67	0	36:64
143	**CD1**	20	CsOH (s, 5 equiv)	Bn	Boc	Cl	*m*-xylene	-	1	100	78	0	10:90

Furter optimisation of the benzyl substituent led to the optimal enantioselectivity (Entry **144**).

Exp	Cat	(mol%)	Base	R	Pg	X	Solvent	additive/ condition	t (h)	Conv	Yield		3 *er*
											3	HA	
144	QN4	20	CsOH (s, 5 equiv)	**Bn**	Boc	Cl	*m*-xylene	-	1	100	80	0	5:95
145	QN4	20	CsOH (s, 5 equiv)	**2-Menapthyl**	Boc	Cl	*m*-xylene	-	1	100	44	24	29:71
146	QN4	20	CsOH (s, 5 equiv)	**4-CF_3Bn**	Boc	Cl	*m*-xylene	-	1	100	86	0	12:88
147	QN4	20	CsOH (s, 5 equiv)	**4-OMeBn**	Boc	Cl	*m*-xylene	-	1	100	89	trace	93:7

A final screening of catalysts showed that cinchonidine CD6 gave the optimal enantioselectivity (Entry **155**)

Exp	Cat	(mol%)	Base	R	Pg	X	Solvent	additive/ condition	t (h)	Conv	Yield		3 *er*
											3	HA	
148	**QN4**	20	CsOH (s, 5 equiv)	Bn	Boc	Cl	*m*-xylene	-	1	100	88	0	6:94
149	**QN1**	20	CsOH (s, 5 equiv)	Bn	Boc	Cl	*m*-xylene	-	1	100	60	7	30:70
150	**CD3**	20	CsOH (s, 5 equiv)	Bn	Boc	Cl	*m*-xylene	-	1	100	97	0	4:96
151	**CN1**	20	CsOH (s, 5 equiv)	Bn	Boc	Cl	*m*-xylene	-	1	100	89	0	93:7
152	**CD4**	20	CsOH (s, 5 equiv)	Bn	Boc	Cl	*m*-xylene	-	1	100	16	0	9:91
153	**CD5**	20	CsOH (s, 5 equiv)	Bn	Boc	Cl	*m*-xylene	-	1	100	13	trace	8:92

154	**DQ1**	20	CsOH (s, 5 equiv)	Bn	Boc	Cl	*m*-xylene	-	1	100	54	8	40:60
**155**	**CD6**	**20**	**CsOH (s, 5 equiv)**	**Bn**	**Boc**	**Cl**	***m*-xylene**	**-**	**1**	**100**	**92**	**0**	**3:97**
156	**CD6**	20	CsOH (s, 5 equiv)	Bn	Boc	Cl	*m*-xylene	-	1	100	90	0	3:97

Reaction Sensitivity

The reaction sensitivity was studied using the methodology described by Glorius.[4] This is the first time that this full assessment has been done for a reaction using an asymmetric phase transfer catalyst/chiral cation and the first fully reported assessment for an enantioselective reaction.[5] We chose the following 10 experiments (excluding control reaction) to assess the reaction sensitivity when changes were made to the standard conditions: (Table **6**).

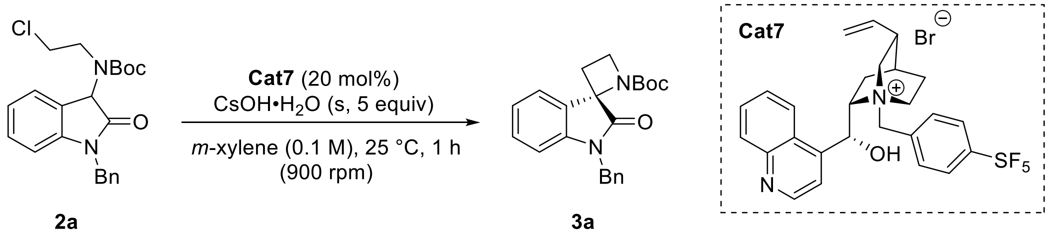

2a **3a**

Entry	Variable	Description	Yield of 3a[a]	Yield (±%)	*Ee0jx*[b]	*ee* (±%)
1	control	no deviation	94	-	94	-
2	low conc	0.09 M	98	4	94	0
3	high conc	0.11 M	90	–4	94	0
4	high H_2O	+ 5 µL H_2O	80	–14	94	0
5	low H_2O	flame-dried vial	98	4	94	0
6	low O_2	under Ar	100	6	90	–4
7	high T	35 ºC	87	–7	91	–3
8	low T	15 ºC	29	–65	68	–26
9	high stir	1150 rpm	33	–61	68	–26
10	low stir	650 rpm	27	–67	66	–28
11	big scale	1.0 mmol scale	72 (60)	–22	88	–6

Table 6: Sensitivity screen using optimised standard conditions. Reactions on 0.05 mmol scale unless otherwise stated. Reactions under air unless otherwise stated. [a] Yields calculated by ^1H NMR spectroscopy with respect to 1,3,5-trimethoxybenzene as an internal standard. [b] er determined by HPLC analysis of crude reaction mixture on a chiral stationary phase.

Concentration

Increasing or decreasing the concentration from the optimal 0.1 M concentration effected the yield and enantioselectivity weakly, generally resulting in a decreased enantioselectivity (Table **6**, Entries **2 and 3**).

Presence of Air

All reactions were conducted under an air atmosphere. When air was excluded the yield was high but the enantioselectivity was slightly impacted. Flame drying and putting under Ar had a detrimental effect on the enantioselectivity (Table **6**, Entries **4-6**).

Temperature

Increasing the temperature above 25 ºC increased the yield but reduced enantioselectivity (Table **6**, Entry **7**). Reducing the temperature is a common tactic to improve asymmetric induction *via* reduction of the rate of competing background reactions. Reduction to 15 ºC gave no improvement in yield or enantioselectivity (Table **6**, Entry **8**).). The lower temperature appears to inhibit the phase transfer catalysis and inhibits cyclisation leading to much higher rate of degradation of the substrate.

Effect of Stirring Rate

When examining the variability of the reaction, one of the key variables was the rate of stirring (Table **7**). High and low rates of stirring afforded poor yields and enantioselectivity. An optimum range of stirring was identified as 800-900 rpm with repeatable results achieved when using a stirring rate of 900 rpm.

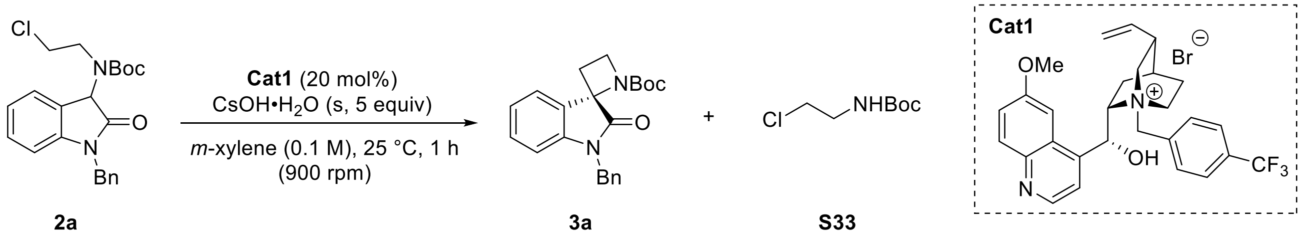

Exp	Stirring rate (rpm)	Yield (%) [a]			3a *er* [b]
		2a (SM)	S33 (BP)	3a	
1	500	0	13	30	20:80 (60)
2	500	0	7	27	15:85
3	700	0	trace	89	7:93
4	800	0	0	98	6:94
5	900	0	trace	87	5:95 (90)
6	1000	0	9	42	14:86
7	1100	0	5	23	15:85
8	1200	0	11	46	11:89
9	1200	0	8	25	12:88
10	1500	49	27	24	30:70 (40)
11	900	0	0	92	5:95 (90)

Table 7: Effect of rate of stirring on the yield and enantioselectivity of the reaction. Reaction on 0.05 mmol scale. [a] Yields are in situ ^1H NMR yields with respect to 1,3,5-trimethoxybenzene as an internal standard. [b] Enantiomeric ratio determined by chiral HPLC of the crude reaction mixture.

Catalyst Degradation

When optimising the reaction it was observed that catalyst **QN4** degraded under the reaction conditions. Scheme **1** shows the isolation of the desired spiroazetidine product **3a** and a side product which was identified as **S76**. HPLC traces of the crude reaction mixture, isolated product **3a** and catalyst degradation product **S76** are shown below (Scheme **1**).

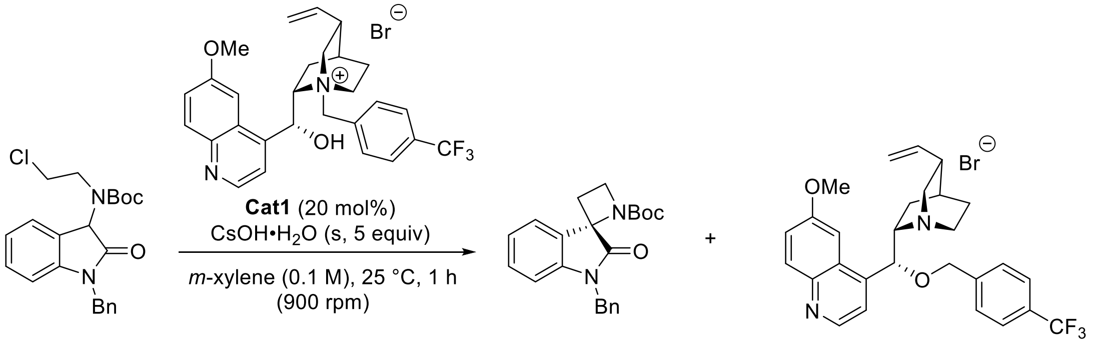

2a **3a**, 90%, 11:89 *er* **S76**, 95%

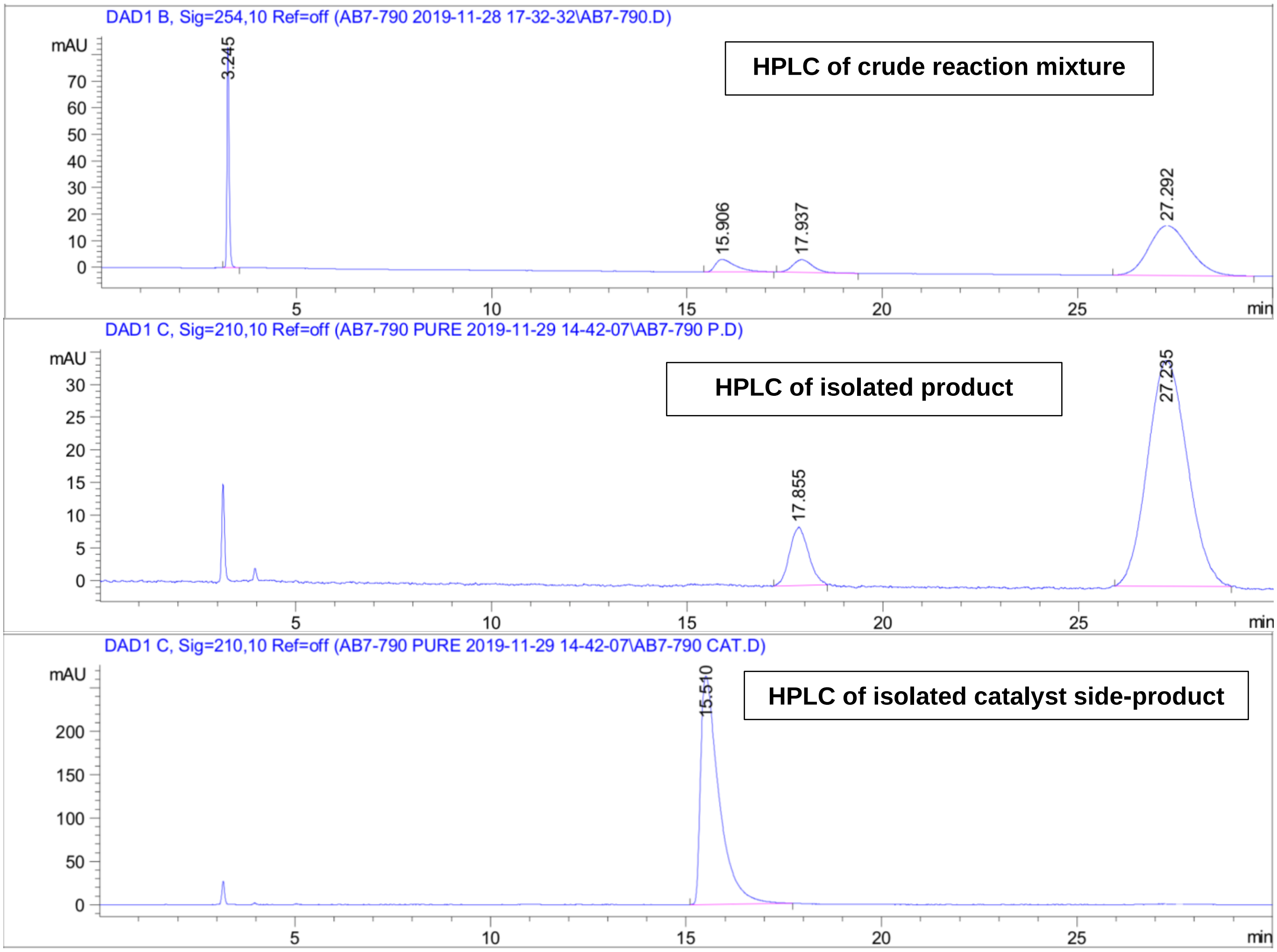

Scheme 1: HPLC traces for the enantioselective spirocyclisation reaction. Column conditions: ID–10%–30min.

Subjecting the catalyst to the reaction conditions led to quantitative conversion to **S76**

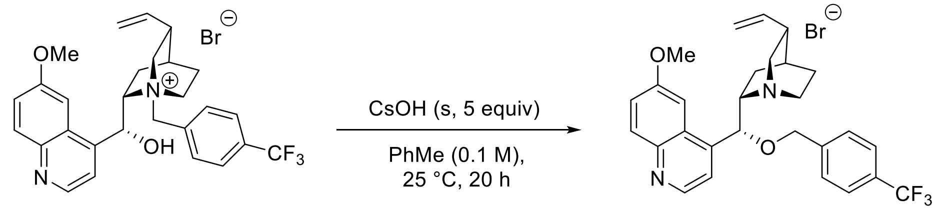

Cat1 **S76**, 96%

Scheme 2: *O*-Alkylation of catalyst **QN4** under the reaction conditions. Reaction on 0.1 mmol scale.

Testing the effect of this catalyst degradation on the enantioselectivity of the cyclisation of **2a** using **Cat1** as the catalyst showed that there was no significant effect on the yield or enantioselectivity (Scheme **3**). This suggests that the catalyst degradation does not compete with the activity of the catalyst acting as a chiral cation. In addition, it showed that **xx** does not catalyse the reaction independently as only degradation of **xx** was observed in the absence of **QN4**.

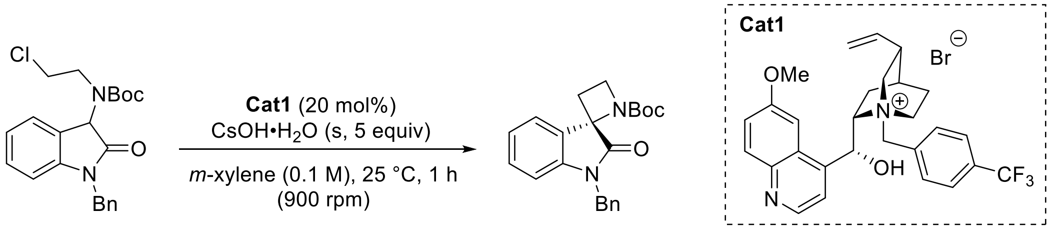

2a **3a**

Exp	Change	Yield of 3a (%) [a]	3a er [b]
1	none	97	7:93
2	no **Cat1** + 20 mol% **S76**	0	-
3	**Cat1** + 20 mol% **S76**	87	5:95

Scheme 3: Effect of the degradation of the catalyst on the yield and enantioselectivity of the cyclisation reaction. [a] Yields are in situ ^{1}H NMR yields with respect to 1,3,5-trimethoxybenzene as an internal standard. [b] Enantiomeric ratio determined by chiral HPLC of the crude reaction mixture.

Diazo Compounds

3-Diazoindolin-2-one (S1)

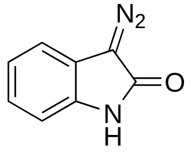

Tosylhydrazide (7.66 g, 44.0 mmol) in MeOH (40 mL) was heated to 70 ºC to solubilise the hydrazide. The mixture was cooled to rt and isatin (5.90 g, 40.0 mmol) was added. The reaction mixture was stirred at 70 ºC for 10 min, then cooled to rt. The solid was filtered, then transferred to a new reaction flask. H_2O (200 mL) was added, then NaOH (3.2 g, 80.0 mmol) was added and the reaction mixture was stirred at 70 ºC for 3 h. The aqueous reaction mixture was extracted with EtOAc (3 × 200 mL) and the combined organic layers were dried over Na_2SO_4, filtered and concentrated under reduced pressure. The crude reaction mixture was dry-loaded onto silica (CH_2Cl_2). Purification by flash chromatography (60% EtOAc/hexane to EtOAc) afforded 3-diazoindolin-2-one **S1** (5.37 g, 84%) as an orange solid. R_f 0.30 (60% EtOAc/hexane); ν_{max} (film)/cm^{-1} 3019 (br s, NH), 2080 (C=N=N out-of-phase), 1674 (C=O), 1614, 1394 (C=N=N in-phase), 1208, 1096, 988, 742; ^{1}H NMR (400 MHz, $CDCl_3$) δ 9.52–9.37 (br d, 1 H, NH), 7.19 (d, J = 7.9 Hz, 1 H, HC_{Ar}), 7.15 (dd, J = 7.7, 1.3 Hz, 1 H, HC_{Ar}), 7.08 (dt, J = 7.6, 1.1 Hz, 1 H, HC_{Ar}), 7.05–7.01 (m, 1 H, HC_{Ar}); ^{13}C NMR (101 MHz, $CDCl_3$) δ 169.5 (C=O quat), 132.0 (NC_{Ar} quat), 125.5 (C_{Ar}), 122.1 (C_{Ar}), 118.3 (C_{Ar}), 117.2 (C_{Ar} quat), 110.8 (C_{Ar}), 61.5 ($C=N_2$). The observed characterisation data (^{1}H NMR) was consistent with that previously reported in the literature.[6]

SMILES: O=C1NC2=CC=CC=C2C1=[N+]=[N-]
InChI = 1S/C8H5N3O/c9-11-7-5-3-1-2-4-6(5)10-8(7)12/h1-4H,(H,10,12)

1-Benzyl-3-diazoindolin-2-one (1)

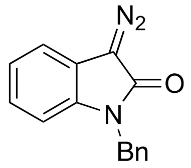

BnBr (1.30 mL) was added to a stirring solution of 3-diazoindolin-2-one **S1** (1.59 g, 10.0 mmol), K_2CO_3 (1.66 g, 12.0 mmol) and TBAI (370 mg, 1.0 mmol) in DMF (10.0 mL) at 25 ºC. The reaction mixture was stirred for 18 h, then H_2O (100 mL) was added and the aqueous reaction mixture was extracted with EtOAc (2 × 100 mL). The combined organic layers were washed with brine (2 × 100 mL), dried over Na_2SO_4, filtered, and concentrated under reduced pressure to afford 1-benzyl-3-diazoindolin-2-one **1** (2.06 g, 83%) as an orange solid. R_f 0.50 (50% Et_2O/hexane); ν_{max} (film)/cm^{-1} 2117 (C=N=N out-of-phase), 1662 (C=O), 1610, 1342 (C=N=N in-phase), 1167, 734, 697; ^{1}H NMR (400 MHz, $CDCl_3$) δ 7.34–7.29 (m, 4 H, 4 × HC_{Ar}), 7.28–7.25 (m, 1 H, HC_{Ar}), 7.23–7.20 (m, 1 H, HC_{Ar}), 7.12–7.05 (m, 2 H, 2 × HC_{Ar}), 6.85–6.82 (m, 1 H, HC_{Ar}), 5.04 (s, 2 H, NCH_2Ph); ^{13}C NMR (101 MHz, $CDCl_3$) δ 166.8 (C=O), 136.0 (NC_{Ar} quat), 133.6 (C_{Ar} quat), 128.7 (2 × C_{Ar}), 127.6 (C_{Ar}), 127.2 (2 × C_{Ar}), 125.4 (C_{Ar}), 122.1 (C_{Ar}), 118.2 (C_{Ar}), 116.7 (C_{Ar} quat), 109.5 (C_{Ar}), 44.2 (NCH_2Ph). The observed characterisation data (^{1}H, ^{13}C NMR and IR) was consistent with that previously reported in the literature.[10]

3-Diazo-5-methoxyindolin-2-one (S2)

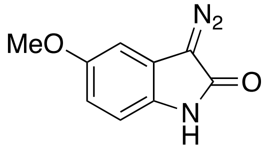

Tosylhydrazide (1.92 g, 11.0 mmol) in MeOH (10.0 mL) was heated to 70 ºC to solubilise the hydrazide. The mixture was cooled to rt and 5-methoxyisatin (1.77 g, 10.0 mmol) was added. The reaction mixture was stirred at 70 ºC for 25 min, then cooled to rt. The solid was filtered, then transferred to a new reaction flask. H_2O (100 mL) was added, then NaOH (0.8 g, 20.0 mmol) was added and the reaction mixture was stirred at 70 ºC for 3 h. The aqueous reaction mixture was extracted with EtOAc (3 × 100 mL) and the combined organic layers were dried over Na_2SO_4, filtered and concentrated under reduced pressure to afford 3-diazo-5-methoxyindolin-2-one **S2** (1.54 g, 81%) as an orange solid which was used without further purification. R_f 0.20 (60% EtOAc/hexane); ν_{max} (film)/cm^{-1} 3094 (br s, NH), 3030, 2885, 2832, 2788, 2087 (C=N=N out-of-phase), 1666 (C=O), 1595, 1480, 1413, 1379 (C=N=N in-phase), 1308, 1264, 1197, 1148, 1010, 902, 787, 727; ^{1}H NMR (400 MHz, $CDCl_3$) δ 10.46 (br s, 1 H, NH), 7.11 (d, J = 2.5 Hz, 1 H, HC_{Ar}), 6.80 (d, J = 8.5 Hz, 1 H, HC_{Ar}), 6.66 (dd, J = 8.5, 2.5 Hz, 1 H, HC_{Ar}), 3.71 (s, 3 H, OCH_3); ^{13}C NMR (101 MHz, $CDCl_3$) δ 167.9 (C=O), 154.7 (OC_{Ar} quat), 126.3 (NC_{Ar} quat), 118.0 (C_{Ar} quat), 111.4 (C_{Ar}), 110.4 (C_{Ar}), 105.3

(C_{Ar}), 60.7 (CN_2 quat), 55.5 (OCH_3). The observed characterisation data (IR, ^{1}H and ^{13}C NMR) was consistent with that previously reported in the literature.

SMILES: O=C1NC2=CC=C(OC)C=C2C1=[N+]=[N-]
InChI=1S/C9H7N3O2/c1-14-5-2-3-7-6(4-5)8(12-10)9(13)11-7/h2-4H,1H3,(H,11,13)

1-Benzyl-3-diazo-5-methoxyindolin-2-one (S3)

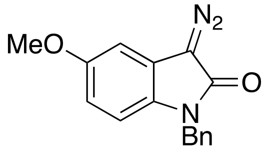

Benzyl bromide (0.95 mL, 8.0 mmol) was added to a solution of 3-diazo-5-methoxyindolin-2-one **S2** (757 mg, 4.0 mmol), K_2CO_3 (2.21 g, 16.0 mmol) and TBAI (300 mg, 0.8 mmol) in MeCN (8.0 mL) at 25 ºC. The reaction mixture was stirred for 19 h at 25 ºC. CH_2Cl_2 (100 mL) was added and the reaction mixture was extracted with sat. aq. $NaHCO_3$ (3 × 100 mL). The combined organic layers were dried over Na_2SO_4, filtered and concentrated under reduced pressure. Purification by flash chromatography (30% EtOAc/hexane; dry loaded with CH_2Cl_2) afforded 1-benzyl-3-diazo-5-methoxyindolin-2-one **S3** (956 mg, 86%) as a pale brown solid. R_f 0.29 (30% EtOAc/hexane); ν_{max} (film)/cm^{-1} 3060, 3029, 3000, 2933, 2833, 2084 (C=N=N out-of-phase), 1670 (C=O), 1591, 1479, 1436, 1389 (C=N=N in-phase), 1353, 1329, 1295, 1284, 1238, 1220, 1197, 1165, 1034, 797, 772, 731; ^{1}H NMR (400 MHz, $CDCl_3$) δ 7.35–7.22 (m, 5 H, 5 × HC_{Ph}), 6.80 (d, J = 2.3 Hz, 1 H, HC_{Ar}), 6.70 (d, J = 8.6 Hz, 1 H, HC_{Ar}), 6.64 (dd, J = 8.6, 2.3 Hz, 1 H, HC_{Ar}), 5.00 (s, 2 H, NCH_2Ph), 3.79 (s, 3 H, OCH_3); ^{13}C NMR (101 MHz, $CDCl_3$) δ 166.6 (C=O), 155.6 (OC_{Ar} quat), 136.0 (C_{Ph} quat), 128.6 (2 × C_{Ph}), 127.52 (C_{Ph}), 127.48 (C_{Ar} quat), 127.2 (2 × C_{Ph}), 117.7 (C_{Ar} quat), 111.0 (C_{Ar}), 110.0 (C_{Ar}), 104.7 (C_{Ar}), 61.2 (CN_2 quat), 55.7 (OCH_3), 44.3 (NCH_2Ph); HRMS (ESI$^+$) m/z Calculated for $C_{16}H_{14}N_3O_2$ [M+H] 280.1086; Found 280.1091.

SMILES: O=C1N(C2=CC=C(OC)C=C2C1=[N+]=[N-])CC3=CC=CC=C3
InChI=1S/C16H13N3O2/c1-21-12-7-8-14-13(9-12)15(18-17)16(20)19(14)10-11-5-3-2-4-6-11/h2-9H,10H2,1H3

1-Benzyl-4-methyl-3-diazoindolin-2-one (S4)

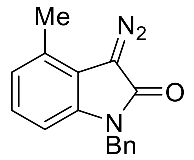

Tosylhydrazide (149 mg, 0.8 mmol) in MeOH (0.7 mL) was added to a stirred solution of 1-benzyl-4-methyl-indoline-2,3-dione (190 mg, 0.8 mmol) in MeOH (2 mL). The reaction mixture was heated to 70 ºC for 30 min. The precipitated yellow solid was collected by filtration and transferred to a new reaction flask. H_2O (10 mL) was added, then NaOH (80 mg, 2.0 mmol) was added and the reaction mixture was stirred at 70 ºC for 3 h. The reaction mixture was cooled to room temperature and extracted with EtOAc (3 × 20 mL) and the combined organic layers were dried over Na_2SO_4, filtered and concentrated under reduced pressure to afford 1-benzyl-3-diazo-4-methylindolin-2-one **S4** (172 mg, 82%) as an orange solid. R_f 0.4 (40% Et2O/hexane); ν_{max} (film)/cm^{-1} 2080 (C=N=N out of phase), 1672 (C=O amide), 1600, 1463, 1396, 1369 (C=N=N in phase), 1218, 1156, 1077, 1026, 762, 693; ^{1}H NMR (400 MHz, DMSO-d_6) δ 7.41 – 7.20 (m, 5 H, 5 × HCPh), 7.03 (t, J = 7.8 Hz, 1 H, HC_{Ar}), 6.90 (d, J = 7.9 Hz, 1 H, HC_{Ar}), 6.84 (d, J = 7.6 Hz, 1 H, HC_{Ar}), 5.01 (s, 2 H, NCH_2Ph), 2.38 (s, 3 H, $ArCH_3$); ^{13}C NMR (101 MHz, DMSO-d_6) δ 167.6 (C=O amide), 137.2 (C_{Ar} quat), 133.4 (C_{Ar}), 130.3 (C_{Ar} quat), 129.1 (2 × C_{Ph}), 127.9 (C_{Ph}), 127.7 (2 × C_{Ph}), 125.9 (C_{Ph}), 124.0 (C_{Ar}), 114.7 (C_{Ar} quat), 107.9 (C_{Ar}), 43.8 (NCH_2Ph), 17.8 ($ArCH_3$); HRMS (ESI$^+$) m/z Calculated for $C_{16}H_{14}N_3O$ $[M+H]^+$ 264.1131; Found 264.1134.

SMILES: O=C1N(C2=CC=CC(C)=C2C1=[N+]=[N-])CC3=CC=CC=C3
InChI=1S/C16H13N3O/c1-11-6-5-9-13-14(11)15(18-17)16(20)19(13)10-12-7-3-2-4-8-12/h2-9H,10H2,1H3

3-Diazo-5-methylindolin-2-one (S5)

Tosylhydrazide (575 mg, 3.3 mmol) in MeOH (3.0 mL) was heated to 70 ºC to solubilise the hydrazide. The mixture was cooled to rt and 5-methylisatin (484 mg, 3.0 mmol) was added. The reaction mixture was stirred at 70 ºC for 30 min, then cooled to rt. The solid was filtered, then transferred to a new reaction flask. H_2O (30 mL) was added, then NaOH (240 mg, 6.0 mmol) was added and the reaction mixture was stirred at 70 ºC

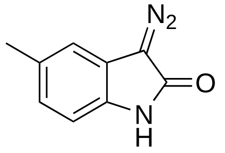

for 4 h. The aqueous reaction mixture was extracted with EtOAc (3 × 200 mL) and the combined organic layers were dried over Na_2SO_4, filtered and concentrated under reduced pressure. The crude reaction mixture was dry-loaded onto silica (CH_2Cl_2). Purification by flash chromatography (50% to 60% EtOAc/hexane) afforded 3-diazo-5-methylindolin-2-one **S5** (325 mg, 63%) as an orange solid. R_f 0.30 (50% EtOAc/hexane); ν_{max} (film)/cm^{-1} 3150, 3056, 3027, 2102 (C=N=N out-of-phase), 1677 (C=O), 1484, 1387 (C=N=N in-phase), 1208, 805, 671; ^1H NMR (400 MHz, $CDCl_3$) δ 9.16 (br s, 1 H, NH), 7.02–7.01 (m, 1 H, HC_{Ar}), 6.98–6.95 (m, 1 H, HC_{Ar}), 6.90 (d, J = 8.0 Hz, 1 H, HC_{Ar}), 2.37 (s, 3 H, CH_3); ^{13}C NMR (101 MHz, $CDCl_3$) δ 169.3 (C=O), 131.8 (C_{Ar} quat), 129.6 (C_{Ar} quat), 126.3 (C_{Ar}), 118.9 (C_{Ar}), 117.3 (C_{Ar} quat), 110.4 (C_{Ar}), 21.2 (CH_3); HRMS (FTMS + pAPCI) *m/z* Calculated for $C_9H_8N_3O^+$ $[M+H]^+$ 174.0662; Found 174.0662.

SMILES: O=C1NC2=CC=C(C)C=C2C1=[N+]=[N-]
InChI=1S/C9H7N3O/c1-5-2-3-7-6(4-5)8(12-10)9(13)11-7/h2-4H,1H3,(H,11,13)

1-Benzyl-3-diazo-5-methylindolin-2-one (S6)

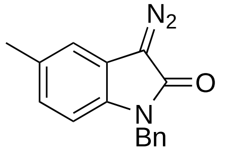

Benzyl bromide (0.13 mL, 1.1 mmol) was added to a solution of 3-diazo-5-methylindolin-2-one **S5** (173 mg, 1.0 mmol), TBAI (37 mg, 0.1 mmol) and K_2CO_3 (166 mg, 1.2 mmol) in DMF (10 mL) at 0 ºC. The reaction mixture was stirred for 19 h at 25 ºC. Sat. aq. NaCl (100 mL) was added and extracted with EtOAc (2 × 100 mL). The combined organic layers were washed with sat. aq. NaCl (3 × 100 mL), dried over Na_2SO_4, filtered and concentrated under reduced pressure. Purification by flash chromatography (25% Et_2O/pentane) afforded 1-benzyl-3-diazo-5-methylindolin-2-one **S6** (223 mg, 85%) as an orange solid. R_f 0.40 (30% Et_2O/pentane); ν_{max} (film)/cm^{-1} 2091 (C=N=N out-of-phase), 1670 (C=O), 1487, 1387 (C=N=N in-phase), 1182, 1122, 801, 719; ^1H NMR (400 MHz, $CDCl_3$) δ 7.34–7.23 (m, 5 H, 5 × HC_{Ar}), 7.04–7.03 (m, 1 H, HC_{Ar}), 6.90 (ddd, J = 8.0, 1.6, 0.7 Hz, 1 H, HC_{Ar}), 6.71 (d, J = 8.0 Hz, 1 H, HC_{Ar}), 5.01 (s, 2 H, NCH_2Ph), 2.34 (s, 3 H, CH_3); ^{13}C NMR (101 MHz, $CDCl_3$) δ 167.0 (C=O), 136.1 (C_{Ar} quat), 131.8 (C_{Ar} quat), 131.5 (C_{Ar} quat),128.7 (2 × C_{Ar}), 127.6 (C_{Ar}), 127.2 (2 × C_{Ar}), 126.0 (C_{Ar}), 118.9 (C_{Ar}), 116.8 (C_{Ar}), 116.8 (C_{Ar} quat), 109.3 (C_{Ar}), 44.3 (NCH_2Ph), 21.1 (CH_3); HRMS (ESI^+) *m/z* Calculated for $C_{16}H_{14}N_3O$ [M+H] 264.1137; Found 264.1135.

SMILES: O=C1N(C2=CC=C(C)C=C2C1=[N+]=[N-])CC3=CC=CC=C3
InChI=1S/C16H13N3O/c1-11-7-8-14-13(9-11)15(18-17)16(20)19(14)10-12-5-3-2-4-6-12/h2-9H,10H2,1H3

3-Diazo-5-fluoroindolin-2-one (S7)

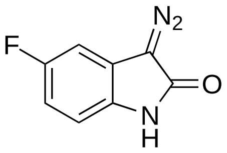

Tosylhydrazide (958 mg, 5.5 mmol) in MeOH (5.0 mL) was heated to 70 ºC to solubilise the hydrazide. The mixture was cooled to rt and 5-fluoroisatin (826 mg, 5.0 mmol) was added. The reaction mixture was stirred at 70 ºC for 10 min, then cooled to rt. The solid was filtered, then transferred to a new reaction flask. H_2O (50 mL) was added, then NaOH (0.4 g, 10.0 mmol) was added and the reaction mixture was stirred at 70 ºC for 3 h. The aqueous reaction mixture was extracted with EtOAc (3 × 100 mL) and the combined organic layers were dried over Na_2SO_4, filtered and concentrated under reduced pressure to afford 3-diazo-5-fluoroindolin-2-one **S7** (735 mg, 83%) as an orange solid which was used without further purification. R_f 0.32 (40% EtOAc/hexane); ν_{max} (film)/cm^{-1} 3284, 2087 (C=N=N out-of-phase), 1685 (C=O), 1651, 1476, 1402, 1286, 1245, 1189, 906, 846, 805; ^1H NMR (400 MHz, DMSO-d_6) δ 10.68 (br s, 1 H, NH), 7.40 (dd, J = 9.0, 2.5 Hz, 1 H, HC_{Ar}), 6.94–6.84 (m, 2 H, 2 × HC_{Ar}); ^{13}C NMR (101 MHz, DMSO-d^6) δ 167.6 (C=O quat), 157.6 (d, $J_{C–F}$ = 235.2 Hz, FC_{Ar} quat), 128.9 (C_{Ar}), 118.5 (d, $J_{C–F}$ = 11.4 Hz, C_{Ar} quat), 111.6 (d, $J_{C–F}$ = 24.1 Hz, C_{Ar}), 111.5 (d, $J_{C–F}$ = 9.0 Hz, C_{Ar}), 106.6 (d, $J_{C–F}$ = 27.8 Hz, C_{Ar} quat), 61.2 (C=N_2 quat); ^{19}F NMR (377 MHz, DMSO-d_6) δ –122.0. The observed characterisation data (IR, ^1H and ^{13}C NMR) was consistent with that previously reported in the literature.[7]

SMILES: O=C1NC2=CC=C(F)C=C2C1=[N+]=[N-]
InChI=1S/C8H4FN3O/c9-4-1-2-6-5(3-4)7(12-10)8(13)11-6/h1-3H,(H,11,13)

1-Benzyl-3-diazo-5-fluoroindolin-2-one (S8)

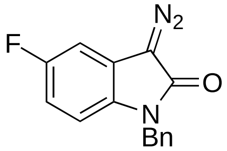

Benzyl bromide (0.32 mL, 2.7 mmol) was added to a solution of 3-diazo-5-fluoroindolin-2-one **S7** (239 mg, 1.35 mmol), K_2CO_3 (746 mg, 5.4 mmol) and TBAI (100 mg, 0.27 mmol) in MeCN (2.7 mL) at 25 ºC. The reaction mixture was stirred for 14 h at 25 ºC. CH_2Cl_2 (50 mL) was added and the reaction mixture was extracted with sat. aq. $NaHCO_3$ (3 × 50 mL). The combined organic layers were dried over Na_2SO_4, filtered and concentrated under reduced pressure. Purification by flash chromatography (30% Et_2O/pentane) afforded 1-benzyl-3-diazo-5-fluoroindolin-2-one **S8** (261 mg, 73%) as an orange solid. R_f 0.31 (30% Et_2O/pentane); ν_{max} (film)/cm^{-1} 3064, 2926, 2087 (C=N=N out-of-phase), 1674 (C=O), 1603, 1476, 1394 (C=N=N in-phase), 1357, 1297, 1159, 850, 783; ^{1}H NMR (400 MHz, $CDCl_3$) δ 7.36–7.25 (m, 5 H, 5 × HC_{Ph}), 6.96 (dd, J = 8.0, 2.4 Hz, 1 H, HC_{Ar}), 6.83–6.77 (m, 1 H, HC_{Ar}), 6.71 (dd, J = 8.6, 4.2 Hz, 1 H, HC_{Ar}), 5.02 (s, 2 H, NCH_2Ph); ^{13}C NMR (101 MHz, $CDCl_3$) δ 166.5 (C=O quat), 158.8 (d, $J_{C–F}$ = 239.8 Hz, FC_{Ar} quat), 135.7 (C_{Ar} quat), 129.6 (C_{Ph} quat), 128.8 (2 × C_{Ph}), 127.7 (C_{Ph}), 127.2 (2 × C_{Ph}), 117.9 (d, $J_{C–F}$ = 10.7 Hz, C_{Ar} quat), 112.1 (d, $J_{C–F}$ = 23.9 Hz, C_{Ar}), 110.0 (d, $J_{C–F}$ = 8.7 Hz, C_{Ar}), 105.8 (d, $J_{C–F}$ = 27.3 Hz, C_{Ar}), 61.5 (s, C=N_2 quat), 44.4 (NCH_2Ph); ^{19}F NMR (377 MHz, $CDCl_3$) δ –120.4. The observed characterisation data (IR, ^{1}H, ^{13}C and ^{19}F NMR) was consistent with that previously reported in the literature.[8]

SMILES: O=C1N(C2=CC=C(F)C=C2C1=[N+]=[N-])CC3=CC=CC=C3
InChI=1S/C15H10FN3O/c16-11-6-7-13-12(8-11)14(18-17)15(20)19(13)9-10-4-2-1-3-5-10/h1-8H,9H2

3-Diazo-7-fluoroindolin-2-one (S9)

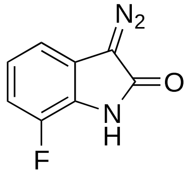

Tosylhydrazide (1.92 g, 11.0 mmol) in MeOH (10.0 mL) was heated to 70 ºC to solubilise the hydrazide. The mixture was cooled to rt and 7-fluoroisatin (1.65 g, 10.0 mmol) was added. The reaction mixture was stirred at 70 ºC for 10 min, then cooled to rt. The solid was filtered, then transferred to a new reaction flask. H_2O (100 mL) was added, then NaOH (0.8 g, 20.0 mmol) was added and the reaction mixture was stirred at 70 ºC for 5 h. The aqueous reaction mixture was extracted with EtOAc (3 × 100 mL) and the combined organic layers were dried over Na_2SO_4, filtered and concentrated under reduced pressure to afford 3-diazo-7-fluoroindolin-2-one **S9** (912 mg, 51%) as an orange solid which was used without further purification. R_f 0.37 (50% EtOAc/hexane); ν_{max} (film)/cm^{-1} 3123 (br s, NH), 3034, 2993, 2102 (C=N=N out-of-phase), 1636 (C=O), 1599, 1390 (C=N=N in-phase), 1334, 1249, 1215, 1182, 885, 869, 768, 697; ^{1}H NMR (400 MHz, DMSO-d_6) δ 11.19 (br s, 1 H, NH), 7.31–7.26 (m, 1 H, HC_{Ar}), 7.04–6.96 (m, 2 H, 2 × HC_{Ar}); ^{13}C NMR (101 MHz, DMSO-d_6) δ 167.3 (C=O quat), 146.6 (d, $J_{C–F}$ = 241.2 Hz, FC_{Ar} quat), 122.0 (d, $J_{C–F}$ = 6.3 Hz, C_{Ar}), 120.1 (d, $J_{C–F}$ = 6.1 Hz, C_{Ar} quat), 119.6 (d, $J_{C–F}$ = 15.1 Hz, C_{Ar} quat), 115.3 (d, $J_{C–F}$ = 2.9 Hz, C_{Ar}), 111.4 (d, $J_{C–F}$ = 17.3 Hz, C_{Ar}), 61.1 (C=N_2 quat); ^{19}F NMR (377 MHz, DMSO-d^6) δ –133.3; HRMS (pAPCI) m/z Calculated for $C_8H_5FN_3O^+$ $[M+H]^+$ 178.0411; Found 178.0414.

SMILES: O=C1NC2=C(F)C=CC=C2C1=[N+]=[N-]
InChI=1S/C8H4FN3O/c9-5-3-1-2-4-6(5)11-8(13)7(4)12-10/h1-3H,(H,11,13)

1-Benzyl-3-diazo-7-fluoroindolin-2-one (S10)

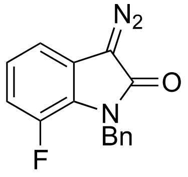

Benzyl bromide (0.71 mL, 6.0 mmol) was added to a solution of 3-diazo-7-fluoroindolin-2-one **S9** (531 mg, 3.0 mmol), K_2CO_3 (1.66 g, 12.0 mmol) and TBAI (222 mg, 0.6 mmol) in MeCN (6.0 mL) at 25 ºC. The reaction mixture was stirred for 19 h at 25 ºC. CH_2Cl_2 (100 mL) was added and the reaction mixture was extracted with sat. aq. $NaHCO_3$ (3 × 100 mL). The combined organic layers were dried over Na_2SO_4, filtered and concentrated under reduced pressure. Purification by flash chromatography (30% Et_2O/pentane) afforded 1-benzyl-3-diazo-7-fluoroindolin-2-one **S10** (557 mg, 70%) as an orange solid. R_f 0.28 (30% Et_2O/pentane); ν_{max} (film)/cm^{-1} 3064, 3034, 2091 (C=N=N out-of-phase), 1685 (C=O), 1625, 1592, 1491, 1469, 1394 (C=N=N in-

phase), 1334, 1241, 1156, 768, 716; ^{1}H NMR (400 MHz, $CDCl_3$) δ 7.39–7.24 (m, 5 H, 5 × HC_{Ph}), 7.03–6.96 (m, 2 H, 2 × HC_{Ar}), 6.90–6.84 (m, 1 H, HC_{Ar}), 5.17 (s, 2 H, NCH_2Ph); ^{13}C NMR (101 MHz, $CDCl_3$) δ 166.2 (C=O quat), 147.8 (d, $J_{C–F}$ = 244.0 Hz, FC_{Ar} quat), 137.1 (C_{Ph} quat), 128.6 (2 × C_{Ph}), 127.6 (C_{Ph}), 127.5 (2 × C_{Ph}), 122.7 (d, $J_{C–F}$ = 7.1 Hz, C_{Ar}), 120.5 (d, $J_{C–F}$ = 11.4 Hz, C_{Ar} quat), 119.5 (d, $J_{C–F}$ = 5.5 Hz, C_{Ar} quat), 114.1 (d, $J_{C–F}$ = 2.9 Hz, C_{Ar}), 112.8 (d, $J_{C–F}$ = 19.6 Hz, C_{Ar}), 46.0 (d, $J_{C–F}$ = 4.7 Hz, NCH_2Ph); ^{19}F NMR (377 MHz, $CDCl_3$) δ –133.2. The observed characterisation data (IR, ^{1}H, ^{13}C and ^{19}F NMR) was consistent with that previously reported in the literature.[9]

SMILES: O=C1N(C2=C(F)C=CC=C2C1=[N+]=[N-])CC3=CC=CC=C3
InChI=1S/C15H10FN3O/c16-12-8-4-7-11-13(18-17)15(20)19(14(11)12)9-10-5-2-1-3-6-10/h1-8H,9H2

4-Bromo-3-diazoindolin-2-one (S11)

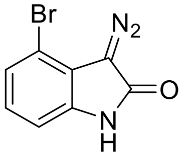

Tosylhydrazide (2.04 g, 11.0 mmol) in 1% aq. H_2SO_4 in MeOH (10.0 mL) was stirred at 25 ºC to solubilise the hydrazide. 4-bromosatin (2.26 g, 10.0 mmol) was added and the reaction mixture was stirred at 55 ºC for 1 h, then cooled to rt. The solid was filtered, then transferred to a new reaction flask. H_2O (100 mL) was added, then NaOH (0.8 g, 20.0 mmol) was added and the reaction mixture was stirred at 70 ºC for 3 h. The aqueous reaction mixture was extracted with EtOAc (3 × 100 mL) and the combined organic layers were dried over Na_2SO_4, filtered and concentrated under reduced pressure to afford 4-bromo-diazoindolin-2-one **S11** (2.09 g, 88%) as an orange solid which was used without further purification. R_f 0.51 (50% EtOAc/hexane) ^{1}H NMR (400 MHz, DMSO-d_6) δ 10.98 (s, 1 H, NH), 7.18 (d, J = 8.1 Hz, 1 H, HC_{Ar}), 7.05 (t, J = 8.0 Hz, 1 H, HC_{Ar}), 6.93 (d, J = 7.8 Hz, 1 H, HC_{Ar}). ^{13}C NMR (101 MHz, DMSO-d_6) δ 167.2 (C=O), 134.2 (NC_{Ar} quat), 126.7 (C_{Ar}), 124.4 (C_{Ar}), 116.1 (C_{Ar} quat), 113.3 (BrC_{Ar} quat), 109.4 (C_{Ar}); HRMS (FTMS + pAPCI) m/z Calculated for $C_8H_5{}^{79}BrN_3O$ 237.9611; Found 237. 9616.

SMILES: O=C1NC2=CC=CC(Br)=C2C1=[N+]=[N-]
InChI=1S/C8H4BrN3O/c9-4-2-1-3-5-6(4)7(12-10)8(13)11-5/h1-3H,(H,11,13)

1-Benzyl-4-bromo-3-diazoindolin-2-one (S12)

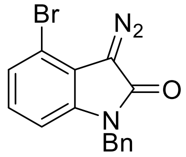

Benzyl bromide (0.47 mL, 3.9 mmol) was added to a solution of 4-bromo-3-diazoindolin-2-one **S11** (470 mg, 2.0 mmol), K_2CO_3 (1.08 g, 7.9 mmol) and TBAI (145 mg, 0.4 mmol) in MeCN-DMF (1:1, 20 mL) at 25 ºC. The reaction mixture was stirred for 18 h, then H_2O (100 mL) and CH_2Cl_2 (100 mL) was added. The aqueous layer was extracted with CH_2Cl_2 (2 × 100 mL). The combined organic layers were washed with sat. aq. $NaHCO_3$, brine (3 x 50 mL), dried over Na_2SO_4, filtered and concentrated under reduced pressure to afford 1-benzyl-4-bromo-3-diazoindolin-2-one **S12** (523 mg, 80%) as an orange solid. R_f 0.34 (40% EtOAc/hexanes); ν_{max} (film)/cm^{-1} 2090 (C=N=N out-of-phase), 1679 (C=O amide), 1602, 1577, 1446, 1395, 1367 (C=N=N in-phase), 1285, 1158, 1128, 1021, 732, 682, 580; ^{1}H NMR (400 MHz, $CDCl_3$) δ 7.36 – 7.21 (m, 5 H, 5 × HCAr), 7.12 (d, J = 8.2 Hz, 1 H, HC_{Ar}), 6.92 (t, J = 8.0 Hz, 1 H, HC_{Ar}), 6.75 (d, J = 7.9 Hz, 1 H, HC_{Ar}), 5.01 (s, 2H, NCH_2Ph); ^{13}C NMR (101 MHz, $CDCl_3$) δ 166.8 (C=O), 135.7 (C_{Ph} quat), 134.9 (NC_{Ar} quat), 128.9 (2 × C_{Ph}), 127.9 (C_{Ph}), 127.4 (2 × C_{Ph}), 126.3 (C_{Ar}), 125.5 (C_{Ar}), 116.3 (C_{Ar} quat), 114.1 (C_{Ar}), 108.5 (C_{Ar}), 62.7 (CN_2 quat), 44.6 (NCH_2Ph); HRMS (ESI$^+$) m/z Calculated for $C_{15}H_{11}{}^{79}BrN_3O$ 328.0085; Found 328.0088.

SMILES: O=C1N(C2=CC=CC(Br)=C2C1=[N+]=[N-])CC3=CC=CC=C3
InChI=1S/C15H10BrN3O/c16-11-7-4-8-12-13(11)14(18-17)15(20)19(12)9-10-5-2-1-3-6-10/h1-8H,9H2

5-Bromo-3-diazoindolin-2-one (S13)

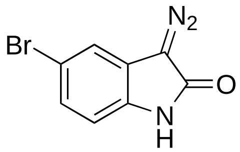

Tosylhydrazide (958 mg, 5.5 mmol) in MeOH (5.0 mL) was heated to 70 ºC to solubilise the hydrazide. The mixture was cooled to rt and 5-bromoisatin (1.13 g, 5.0 mmol) was added. The reaction mixture was stirred at 70 ºC for 10 min, then cooled to rt. The solid was filtered, then transferred to a new reaction flask. H_2O (50 mL) was added, then NaOH (0.4 g, 10.0 mmol) was added and the reaction mixture was stirred at 70 ºC for 6 h. The aqueous reaction mixture was extracted with EtOAc (3 × 100 mL) and the combined organic layers were dried over Na_2SO_4, filtered and concentrated under reduced pressure. Purification by flash chromatography (50% to 70% EtOAc/hexane) afforded 5-bromo-3-diazoindolin-2-one **S13** (782 mg, 66%) as an orange solid. R_f 0.18 (50% EtOAc/hexane); ν_{max} (film)/cm^{-1} 3265 (br s, NH), 3101, 2095 (C=N=N out-of-phase), 1700 (C=O), 1648, 1469, 1394 (C=N=N in-phase), 1282, 1245, 1193, 1059, 1014, 821; ^{1}H NMR (400 MHz, DMSO-*d*$_6$) δ 10.80 (s, 1 H, NH), 7.68 (d, *J* = 1.8 Hz, 1 H, HC_{Ar}), 7.23 (dd, *J* = 8.3, 1.9 Hz, 1 H, HC_{Ar}), 6.84 (d, *J* = 8.3 Hz, 1 H, HC_{Ar}); ^{13}C NMR (101 MHz, DMSO-*d*$_6$) δ 167.3 (C=O quat), 131.7 (C_{Ar} quat), 127.5 (C_{Ar}), 121.7 (C_{Ar}), 119.5 (C_{Ar} quat), 112.8 (C_{Ar} quat), 111.5 (C_{Ar} quat), 60.5 (C=N_2 quat); HRMS (FTMS + pAPCI) *m/z* Calculated for $C_8H_5N_3O^{79}Br^+$ [M+H]$^+$ 237.9611; Found 237.9601.

SMILES: O=C1NC2=CC=C(Br)C=C2C1=[N+]=[N-]
InChI=1S/C8H4BrN3O/c9-4-1-2-6-5(3-4)7(12-10)8(13)11-6/h1-3H,(H,11,13)

1-Benzyl-5-bromo-3-diazoindolin-2-one (S14)

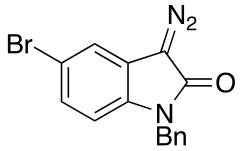

Benzyl bromide (1.19 mL, 10.0 mmol) was added to a solution of 5-bromo-3-diazoindolin-2-one **S13** (1.19 g, 5.0 mmol) and K_2CO_3 (2.76 g, 20.0 mmol) in MeCN (10 mL) at 25 ºC. The reaction mixture was stirred for 14 h at 25 ºC. CH_2Cl_2 (100 mL) was added and the reaction mixture was extracted with sat. aq. $NaHCO_3$ (3 × 100 mL). The combined organic layers were dried over Na_2SO_4, filtered and concentrated under reduced pressure. Purification by flash chromatography (10% to 20% EtOAc/hexane) afforded 1-benzyl-5-bromo-3-diazoindolin-2-one **S14** (1.11 g, 68%) as an orange solid. R_f 0.27 (20% EtOAc/hexane); ν_{max} (film)/cm^{-1} 3030, 3064, 2091 (C=N=N out-of-phase), 1677, 1607, 1476, 1439, 1387 (C=N=N in-phase), 1353, 1170, 1118, 801, 701; ^{1}H NMR (400 MHz, $CDCl_3$) δ 7.35–7.25 (m, 6 H, 5 × HC_{Ph} and HC_{Ar}), 7.19 (dd, *J* = 8.4, 1.9 Hz, 1 H, HC_{Ar}), 6.67 (d, *J* = 8.4 Hz, 1 H, HC_{Ar}), 5.01 (s, 2 H, NCH_2Ph); ^{13}C NMR (101 MHz, $CDCl_3$) δ 166.1 (C=O), 135.5 (C_{Ph} quat), 132.5 (NC_{Ar} quat), 128.8 (2 × C_{Ph}), 128.2 (C_{Ar}), 127.8 (C_{Ph}), 127.2 (2 × C_{Ph}), 120.9 (C_{Ar}), 118.7 (C_{Ar} quat), 114.7 (C_{Ar} quat), 110.8 (C_{Ar}), 44.4 (NCH_2Ph); HRMS (FTMS + pAPCI) *m/z* Calculated for $C_{15}H_{11}N_3O^{79}Br^+$ [M+H]$^+$ 328.0080; Found 328.0076.

SMILES: O=C1N(C2=CC=C(Br)C=C2C1=[N+]=[N-])CC3=CC=CC=C3
InChI=1S/C15H10BrN3O/c16-11-6-7-13-12(8-11)14(18-17)15(20)19(13)9-10-4-2-1-3-5-10/h1-8H,9H2

6-Bromo-3-diazoindolin-2-one (S15)

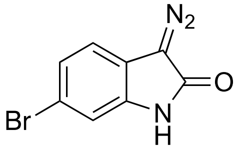

Tosylhydrazide (1.92 g, 11.0 mmol) in MeOH (10 mL) was heated to 70 ºC to solubilise the hydrazide. The mixture was cooled to rt and 6-bromoisatin (2.26 g, 10.0 mmol) was added. The reaction mixture was stirred at 70 ºC for 10 min, then cooled to rt. The solid was filtered, then transferred to a new reaction flask. H_2O (100 mL) was added, then NaOH (800 mg, 20.0 mmol) was added and the reaction mixture was stirred at 70 ºC for 16 h. The aqueous reaction mixture was extracted with EtOAc (3 × 100 mL) and the combined organic layers were dried over Na_2SO_4, filtered and concentrated under reduced pressure to afford 6-bromo-3-diazoindolin-2-one **S15** (1.87 g, 79%) as a red solid which was used without further purification. R_f 0.35 (50% EtOAc/hexane); ν_{max} (film)/cm^{-1} 3120, 2084 (C=N=N out-of-phase), 1681 (C=O), 1599, 1484, 1397 (C=N=N in-phase), 1327, 1208, 1170, 1107, 1055, 995, 910, 861, 801, 742; ^{1}H NMR (400 MHz, DMSO-*d*$_6$) δ 10.80 (br s, 1 H, NH), 7.37 (d, *J* = 8.2 Hz, 1 H, HC_{Ar}), 7.16 (dd, *J* = 8.2, 1.7 Hz, 1 H, HC_{Ar}), 7.03 (d, *J* = 1.7 Hz, 1 H,

HC_{Ar}); ^{13}C NMR (101 MHz, DMSO-d_6) δ 167.4 (C=O), 133.9 (NC_{Ar} quat), 123.7 (C_{Ar}), 120.8 (C_{Ar}), 117.2 (C_{Ar} quat), 116.5 (C_{Ar} quat), 112.5 (C_{Ar}), 60.5 (CN_2 quat); HRMS (FTMS + pAPCI) *m/z* Calculated for $C_8H_5N_3O^{79}Br^+$ $[M+H]^+$ 237.9611; Found 237.9613.

SMILES: O=C1NC2=CC(Br)=CC=C2C1=[N+]=[N-]
InChI=1S/C8H4BrN3O/c9-4-1-2-5-6(3-4)11-8(13)7(5)12-10/h1-3H,(H,11,13)

1-Benzyl-6-bromo-3-diazoindolin-2-one (S16)

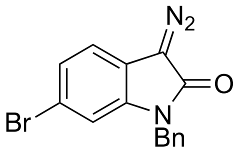

Benzyl bromide (1.19 mL, 10.0 mmol) was added to a solution of 6-bromo-3-diazoindolin-2-one **S15** (1.19 g, 5.0 mmol) and K_2CO_3 (2.76 g, 20.0 mmol) in MeCN (10 mL) at 25 ºC. The reaction mixture was stirred for 14 h at 25 ºC. CH_2Cl_2 (100 mL) was added and the reaction mixture was extracted with sat. aq. $NaHCO_3$ (3 × 100 mL). The organic layers were dried over Na_2SO_4, filtered and concentrated under reduced pressure. Purification by flash chromatography (20% to 30% Et_2O/pentane) afforded 1-benzyl-6-bromo-3-diazoindolin-2-one **S16** (1.45 g, 88%) as an orange solid. R_f 0.37 (30% Et_2O/pentane); ν_{max} (film)/cm^{-1} 3064, 3030, 2087 (C=N=N out-of-phase), 1677 (C=O), 1603, 1580, 1476, 1394 (C=N=N in-phase), 1349, 1256, 1170, 1115, 1062, 910, 861, 798, 731; ^{1}H NMR (400 MHz, $CDCl_3$) δ 7.37–7.26 (m, 5 H, 5 × HC_{Ph}), 7.18 (dd, *J* = 8.1, 1.7 Hz, 1 H, HC_{Ar}), 7.05 (d, *J* = 8.1 Hz, 1 H, HC_{Ar}), 6.97 (d, *J* = 1.7 Hz, 1 H, HC_{Ar}), 4.99 (s, 2 H, NCH_2Ph); ^{13}C NMR (101 MHz, $CDCl_3$) δ 166.4 (C=O quat), 135.4 (C_{Ph} quat), 134.6 (NC_{Ar} quat), 128.8 (2 × C_{Ph}), 127.8 (C_{Ph}), 127.2 (2 × C_{Ph}), 124.9 (C_{Ar}), 119.1 (C_{Ar}), 118.5 (C_{Ar} quat), 115.5 (C_{Ar} quat), 112.6 (C_{Ar}), 60.9 (CN_2 quat), 44.3 (NCH_2Ph); HRMS (ESI$^+$) *m/z* Calculated for $C_{15}H_{11}N_3O^{79}Br$ $[M+H]^+$ 328.0080; Found 328.0081.

SMILES: O=C1N(C2=CC(Br)=CC=C2C1=[N+]=[N-])CC3=CC=CC=C3
InChI=1S/C15H10BrN3O/c16-11-6-7-12-13(8-11)19(15(20)14(12)18-17)9-10-4-2-1-3-5-10/h1-8H,9H2

7-Bromo-3-diazoindolin-2-one (S17)

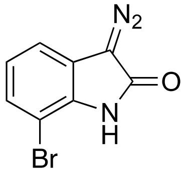

Tosylhydrazide (766 mg, 4.4 mmol) in MeOH (4.0 mL) was heated to 70 ºC to solubilise the hydrazide. The mixture was cooled to rt and 7-bromoisatin (904 mg, 4.0 mmol) was added. The reaction mixture was stirred at 70 ºC for 1 h, then cooled to rt. The solid was filtered, then transferred to a new reaction flask. H_2O (40 mL) was added, then NaOH (320 mg, 8.0 mmol) was added and the reaction mixture was stirred at 70 ºC for 16 h. The aqueous reaction mixture was extracted with EtOAc (3 × 100 mL) and the combined organic layers were dried over Na_2SO_4, filtered and concentrated under reduced pressure to afford 7-bromo-3-diazoindolin-2-one **S17** (526 mg, 55%) as an orange solid which was used without further purification. R_f 0.48 (50% EtOAc/hexane); ν_{max} (film)/cm^{-1} 3109, 3027, 2982, 2773, 2717, 2076 (C=N=N out-of-phase), 1670 (C=O), 1607, 1439, 1379 (C=N=N in-phase), 1323, 1290, 1226, 1185, 1010, 954, 760, 697; ^{1}H NMR (400 MHz, DMSO-d_6) δ 10.99 (br s, 1 H, NH), 7.45 (d, *J* = 7.6 Hz, 1 H, HC_{Ar}), 7.28 (dd, *J* = 8.1, 0.8 Hz, 1 H, HC_{Ar}), 6.96 (t, *J* = 7.8 Hz, 1 H, HC_{Ar}); ^{13}C NMR (101 MHz, DMSO-d_6) δ 167.3 (C=O), 131.5 (NC_{Ar} quat), 127.5 (C_{Ar}), 122.7 (C_{Ar}), 118.8 (C_{Ar} quat), 118.2 (C_{Ar}), 102.3 (BrC_{Ar} quat), 61.4 (CN_2 quat); HRMS (FTMS + pAPCI) *m/z* Calculated for $C_8H_5N_3O^{79}Br^+$ $[M+H]^+$ 237.9611; Found 237.9608.

SMILES: O=C1N(C2=C(Br)C=CC=C2C1=[N+]=[N-])CC3=CC=CC=C3
InChI=1S/C15H10BrN3O/c16-12-8-4-7-11-13(18-17)15(20)19(14(11)12)9-10-5-2-1-3-6-10/h1-8H,9H2

1-Benzyl-7-bromo-3-diazoindolin-2-one (S18)

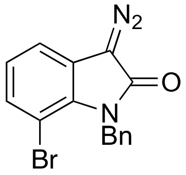

Benzyl bromide (0.33 mL, 2.8 mmol) was added to a solution of 7-bromo-3-diazoindolin-2-one **S17** (340 mg, 1.4 mmol) and K_2CO_3 (773 mg, 5.6 mmol) in MeCN (3.0 mL) at 25 ºC. The reaction mixture was stirred for 16 h at 25 ºC. CH_2Cl_2 (50 mL) was added and the reaction mixture was extracted with sat. aq. $NaHCO_3$ (3 × 50 mL). The organic layers were dried over Na_2SO_4, filtered and concentrated under reduced pressure. Purification by flash chromatography (20% to 30% Et_2O/pentane) afforded 1-benzyl-7-bromo-3-diazoindolin-2-

one **S18** (390 mg, 85%) as a dark orange solid. R_f 0.43 (30% Et_2O/pentane); ν_{max} (film)/cm^{-1} 3064, 3030, 2094 (C=N=N out-of-phase), 1685 (C=O), 1446, 1394 (C=N=N in-phase), 1334, 1156, 1126, 772, 719; ^{1}H NMR (400 MHz, $CDCl_3$) δ 7.34–7.14 (m, 7 H, 5 × HC_{Ph} and 2 × HC_{Ar}), 6.95 (t, J = 7.8 Hz, 1 H, HC_{Ar}), 5.53 (s, 2 H, NCH_2Ph); ^{13}C NMR (101 MHz, $CDCl_3$) δ 167.0 (C=O), 137.6 (C_{Ph} quat), 130.94 (NC_{Ar} quat), 130.89 (C_{Ar}), 128.5 (2 × C_{Ph}), 127.1 (C_{Ph}), 126.3 (2 × C_{Ph}), 123.2 (C_{Ar}), 119.6 (C_{Ar} quat), 117.1 (C_{Ar}), 103.3 (BrC_{Ar} quat), 45.0 (NCH_2Ph). The observed characterisation data (^{1}H NMR) was consistent with that previously reported in the literature.**Error! Bookmark not defined.**

SMILES: O=C1N(C2=C(Br)C=CC=C2C1=[N+]=[N-])CC3=CC=CC=C3
InChI=1S/C15H10BrN3O/c16-12-8-4-7-11-13(18-17)15(20)19(14(11)12)9-10-5-2-1-3-6-10/h1-8H,9H2

5-Chloro-3-diazoindolin-2-one (S19)

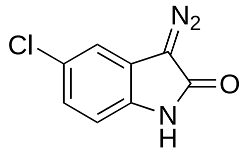

Tosylhydrazide (1.92 g, 11.0 mmol) in MeOH (10 mL) was heated to 70 ºC to solubilise the hydrazide. The mixture was cooled to rt and 5-chloroisatin (1.82 g, 10.0 mmol) was added. The reaction mixture was stirred at 70 ºC for 10 min, then cooled to rt. The solid was filtered, then transferred to a new reaction flask. H_2O (50 mL) was added, then NaOH (0.8 g, 20.0 mmol) was added and the reaction mixture was stirred at 70 ºC for 19 h. The aqueous reaction mixture was extracted with EtOAc (3 × 200 mL) and the combined organic layers were dried over Na_2SO_4, filtered and concentrated under reduced pressure to afford 5-chloro-3-diazoindolin-2-one **S19** (1.49 g, 77%) as an orange solid which was used without further purification. R_f 0.25 (50% EtOAc/hexane); ν_{max} (film)/cm^{-1} 3254 (br s, NH), 2091 (C=N=N out-of-phase), 1696 (C=O), 1648, 1472, 1241, 1394, 1282, 1189, 1070, 887, 861, 701; ^{1}H NMR (400 MHz, DMSO-d_6) δ 10.77 (s, 1 H, NH), 7.54 (d, J = 2.0 Hz, 1 H, HC_{Ar}), 7.09 (dd, J = 8.3, 2.0 Hz, 1 H, HC_{Ar}), 6.88 (d, J = 8.3 Hz, 1 H, HC_{Ar}); ^{13}C NMR (101 MHz, DMSO-d_6) δ 167.3 (C=O), 131.3 (NC_{Ar} quat), 125.2 (C_{Ar} quat), 124.7 (C_{Ar}), 119.0 (C_{Ar} quat), 118.9 (C_{Ar}), 110.9 (C_{Ar}), 60.7 ($C=N_2$ quat); HRMS (FTMS + pAPCI) m/z Calculated for $C_8H_5N_3OCl^+$ $[M+H]^+$ 194.0116; Found 194.0110.

SMILES: O=C1NC2=CC=C(Cl)C=C2C1=[N+]=[N-]
InChI=1S/C8H4ClN3O/c9-4-1-2-6-5(3-4)7(12-10)8(13)11-6/h1-3H,(H,11,13)

1-Benzyl-5-chloro-3-diazoindolin-2-one (S20)

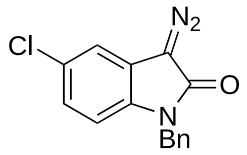

Benzyl bromide (0.48 mL, 4.0 mmol) was added to a solution of 5-chloro-3-diazoindolin-2-one **S19** (390 mg, 2.0 mmol) and K_2CO_3 (550 mg, 4.0 mmol) in MeCN (4.0 mL) at 25 ºC. The reaction mixture was stirred for 18 h at 25 ºC. CH_2Cl_2 (50 mL) was added and the reaction mixture was extracted with H_2O (3 × 50 mL). The combined organic layers were dried over Na_2SO_4, filtered and concentrated under reduced pressure. Purification by flash chromatography (20% to 30% EtOAc/hexane) afforded 1-benzyl-5-chloro-3-diazoindolin-2-one **S20** (338 mg, 60%) as an orange solid. R_f 0.31 (30% EtOAc/hexane); ν_{max} (film)/cm^{-1} 3034, 3064, 2095 (C=N=N out-of-phase), 1681 (C=O), 1610, 1476, 1446, 1387 (C=N=N in-phase), 1170, 1118; ^{1}H NMR (400 MHz, $CDCl_3$) δ 7.36–7.25 (m, 5 H, 5 × HC_{Ph}), 7.19 (d, J = 2.0 Hz, 1 H, HC_{Ar}), 7.05 (dd, J = 8.4, 2.0 Hz, 1 H, HC_{Ar}), 6.72 (d, J = 8.4 Hz, 1 H, HC_{Ar}), 5.01 (s, 2 H, NCH_2Ph); ^{13}C NMR (101 MHz, $CDCl_3$) δ 166.3 (C=O), 135.5 (C_{Ar} quat), 132.1 (C_{Ar} quat), 128.8 (2 × C_{Ph}), 127.8 (C_{Ph}), 127.6 (C_{Ar} quat), 127.2 (2 × C_{Ph}), 125.4 (C_{Ar}), 118.3 (C_{Ar} quat), 118.2 (C_{Ar}), 110.3 (C_{Ar}), 44.4 (NCH_2Ph); HRMS (FTMS + pAPCI) m/z Calculated for $C_{15}H_{11}N_3OCl^+$ $[M+H]^+$ 284.0585; Found 284.0580.

SMILES: O=C1N(CC2=CC=CC=C2)C3=CC=C(Cl)C=C3C1=[N+]=[N-]
InChI=1S/C15H10ClN3O/c16-11-6-7-13-12(8-11)14(18-17)15(20)19(13)9-10-4-2-1-3-5-10/h1-8H,9H2

3-Diazo-5-nitroindolin-2-one (S21)

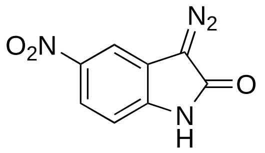

Tosylhydrazide (958 mg, 5.5 mmol) in MeOH (5.0 mL) was heated to 70 ºC to solubilise the hydrazide. The mixture was cooled to rt and 5-nitroisatin (961 mg, 5.0 mmol) was added. The reaction mixture was stirred at 70 ºC for 10 min, then cooled to rt. The solid was filtered, then transferred to a new reaction flask. H_2O (50 mL) was added, then NaOH (0.4 g, 10.0 mmol) was added and the reaction mixture was stirred at 70 ºC for 3 h. The aqueous reaction mixture was extracted with EtOAc (3 × 100 mL) and the combined organic layers were dried over Na_2SO_4, filtered and concentrated under reduced pressure. Purification by flash chromatography (50% EtOAc/hexane to 20% Et_2O/CH_2Cl_2 to 50% Et_2O/CH_2Cl_2)* to afford 3-diazo-5-nitroindolin-2-one **S21** (450 mg, 44%) as a yellow solid. R_f 0.19 (50% EtOAc/hexane); ν_{max} (film)/cm^{-1} 3105 (br s, NH), 2837, 2132 (C=N=N out-of-phase), 1700 (C=O amide), 1614, 1513 (NO_2), 1461, 1379 (C=N=N in-phase), 1342 (NO_2), 1200, 1156, 1088, 883, 820, 768, 738, 675; ^{1}H NMR (400 MHz, $CDCl_3$) δ 11.4 (br s, 1 H, NH), 8.47 (d, J = 2.3 Hz, 1 H, HC_{Ar}), 8.03 (dd, J = 8.7, 2.3 Hz, 1 H, HC_{Ar}), 7.07 (d, J = 8.7 Hz, 1 H, HC_{Ar}); ^{13}C NMR (101 MHz, $CDCl_3$) δ 167.5 (C=O), 141.7 (NO_2C_{Ar} quat), 138.1 (NC_{Ar} quat), 121.5 (C_{Ar}), 118.3 (C_{Ar} quat), 114.9 (C_{Ar}), 109.6 (C_{Ar}), 61.7 (CN_2 quat); HRMS (FTMS – p APCI) *m/z* Calculated for $C_8H_3N_4O_3^-$ $[M–H]^-$ 203.0211; Found 203.0205.

* The crude material was dry loaded with CH_2Cl_2 but did not elute with 50% EtOAc/hexane. The residual CH_2Cl_2 was difficult to remove and **S21** was used in the next step with this residual solvent.

SMILES: O=C1N([H])C2=CC=C([N+]([O-])=O)C=C2C1=[N+]=[N-]
InChI=1S/C8H4N4O3/c9-11-7-5-3-4(12(14)15)1-2-6(5)10-8(7)13/h1-3H,(H,10,13)

1-Benzyl-3-diazo-5-nitroindolin-2-one (S22)

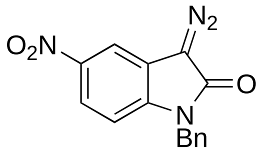

Benzyl bromide (0.48 mL, 4.0 mmol) was added to a solution of 3-diazo-5-nitroindolin-2-one **S21** (408 mg, 2.0 mmol) and K_2CO_3 (553 mg, 4.0 mmol) in MeCN (4.0 mL) at 25 ºC. The reaction mixture was stirred for 14 h at 25 ºC. CH_2Cl_2 (50 mL) was added and the reaction mixture was extracted with H_2O (3 × 50 mL). The combined organic layers were dried over Na_2SO_4, filtered and concentrated under reduced pressure. Purification by flash chromatography (dry loaded with CH_2Cl_2, eluted with 40% to 60% Et_2O/pentane to CH_2Cl_2 then CH_2Cl_2 to 10% Et_2O/CH_2Cl_2) afforded 1-benzyl-3-diazo-5-nitroindolin-2-one **S22** (372 mg, 63%) as a yellow solid. R_f 0.15 (30% Et_2O/pentane); ν_{max} (film)/cm^{-1} 3064, 2113 (C=N=N out-of-phase), 1685 (C=O), 1610, 1517, 1484, 1387 (C=N=N in-phase), 1338, 1170, 1092, 734; ^{1}H NMR (400 MHz, $CDCl_3$) δ 8.14 (d, J = 2.2 Hz, 1 H, HC_{Ar}), 8.05 (dd, J = 8.7, 2.2 Hz, 1 H, HC_{Ar}), 7.38–7.26 (m, 5 H, 5 × HC_{Ph}), 6.90 (d, J = 8.7 Hz, 1 H, HC_{Ar}), 5.09 (s, 2 H, NCH_2Ph); ^{13}C NMR (101 MHz, $CDCl_3$) δ 163.1 (C=O quat), 143.0 (O_2NC_{Ar} quat), 138.3 (NC_{Ar} quat), 134.9 (C_{Ph} quat), 129.0 (2 × C_{Ph}), 128.2 (C_{Ph}), 127.3 (2 × C_{Ph}), 122.0 (C_{Ar}), 118.0 (C_{Ar} quat), 113.6 (C_{Ar}), 108.9 (C_{Ar}), 44.7 (NCH_2Ph). The observed characterisation data (IR, ^{1}H and ^{13}C NMR) was consistent with that previously reported in the literature.[8]

SMILES:O=C1N(CC2=CC=CC=C2)C3=CC=C([N+]([O-])=O)C=C3C1=[N+]=[N-]
InChI=1S/C15H10N4O3/c16-17-14-12-8-11(19(21)22)6-7-13(12)18(15(14)20)9-10-4-2-1-3-5-10/h1-8H,9H2

1-Benzyl-3-diazo-1,3-dihydro-2*H*-pyrrolo[2,3-b]pyridin-2-one (S23)

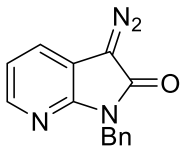

Tosylhydrazide (859 mg, 4.6 mmol) in 5% v/v H_2SO_4 in MeOH (4.2 mL) was added to a stirred solution of 1-benzyl-1*H*-pyrrolo[2,3-b]pyridine-2,3-dione (1.03 g, 4.2 mmol) in MeOH (10 mL). The reaction mixture was heated to 55 ºC for 30 min. The precipitated yellow solid was collected by filtration and transferred to a new reaction flask. H_2O (40 mL) was added, then NaOH (324 mg, 8.1 mmol) was added and the reaction mixture was stirred at 70 ºC for 3 h. The reaction mixture was cooled to room temperature and extracted with EtOAc (3 × 80 mL) and the combined organic layers were dried over Na_2SO_4, filtered and concentrated under reduced pressure to afford

1-benzyl-3-diazo-1,3-dihydro-2*H*-pyrrolo[2,3-b]pyridin-2-one **S23** (561 mg, 54%) as an red solid. R_f 0.47 (45% Et_2O/hexanes); ν_{max} (film)/cm^{-1} 2081 (C=N=N out-of-phase), 1692 (C=O amide), 1599, 1576, 1448, 1398, 1337 (C=N=N inphase), 1276, 1157, 1077, 797, 695; ^{1}H NMR (400 MHz, $CDCl_3$) δ 8.12 (m, 1 H, HC_{Ar}), 7.49 – 7.44 (m, 2 H, 2 × HC_{Ph}), 7.42 (m, 1 H, HC_{Ar}), 7.34 – 7.20 (m, 3 H, 3 × HC_{Ph}), 6.98 (m, 1 H, HC_{Ar}), 5.12 (s, 2 H, NCH_2Ph). ^{13}C NMR (101 MHz, $CDCl_3$) δ 166.1 (C=O quat), 146.9 (NC_{Ar} quat), 144.8 (C_{Ar}), 136.8 (C_{Ph}), 128.7 (2 × C_{Ph}), 128.5 (2 × C_{Ph}), 127.9 (C_{Ph}), 124.9 (CAr), 117.9 (C_{Ar}), 112.1 (C_{Ar}), 43.3(NCH_2Ph); HRMS (ESI$^+$) *m*/*z* Calculated for $C_{14}H_{11}N_4O$ [M+H] 251.0933; Found 251.0923.

SMILES: O=C1N(C2=NC=CC=C2C1=[N+]=[N-])CC3=CC=CC=C3
InChI=1S/C14H10N4O/c15-17-12-11-7-4-8-16-13(11)18(14(12)19)9-10-5-2-1-3-6-10/h1-8H,9H2

3-Diazo-1-methylindolin-2-one (S24)

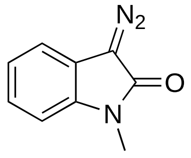

MeI (0.13 mL) was added dropwise to a solution of 3-diazoindolin-2-one **S1** (318 mg, 2.0 mmol) and K_2CO_3 (304 mg, 2.2 mmol) in DMF (4.0 mL) at 25 ºC. The reaction mixture was stirred for 26 h, then H_2O (50 mL) was added followed by EtOAc (50 mL). The layers were separated and the aqueous layer extracted with EtOAc (2 × 50 mL). The combined organic layers were dried over Na_2SO_4, filtered and concentrated under reduced pressure. Purification by flash chromatography (50% Et_2O/pentane) afforded 3-diazo-1-methylindolin-2-one **S24** as an orange solid. R_f 0.30 (50% Et_2O/pentane); ν_{max} (film)/cm^{-1} ; ^{1}H NMR (400 MHz, $CDCl_3$) δ 7.23–7.18 (m, 2 H, 2 × HC_{Ar}), 7.12–7.07 (m, 1 H, HC_{Ar}), 6.94–6.91 (m, 1 H, HC_{Ar}), 3.33 (s, 3 H, NCH_3); ^{13}C NMR (101 MHz, $CDCl_3$) δ 166.8 (C=O), 134.4 (NC_{Ar} quat), 125.4 (C_{Ar}), 122.0 (C_{Ar}), 118.2 (C_{Ar}), 116.6 (C_{Ar} quat), 108.6 (C_{Ar}), 26.8 (CH_3). The observed characterisation data (^{1}H and ^{13}C NMR) was consistent with that previously reported in the literature.[10]

SMILES: O=C1N(C)C2=CC=CC=C2C1=[N+]=[N-]
InChI = 1S/C9H7N3O/c1-12-7-5-3-2-4-6(7)8(11-10)9(12)13/h2-5H,1H3

3-Diazo-1-(4-methoxybenzyl)indolin-2-one (S25)

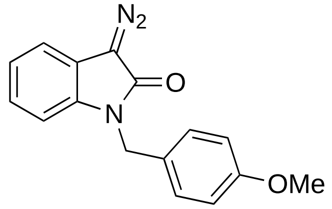

1-(Bromomethyl)-4-methoxybenzene (0.63 mL, 4.4 mmol) was added dropwise to a solution of 3-diazoindolin-2-one **S1** (637 mg, 4.0 mmol), TBAI (148 mg, 0.4 mmol) and K_2CO_3 (663 mg, 4.8 mmol) in DMF (40 mL) at 0 ºC. The reaction mixture was stirred for 15 h at 25 ºC. Sat. aq. NaCl (50 mL) was added and extracted with EtOAc (3 × 50 mL). The combined organic layers were washed with sat. aq. NaCl (2 × 50 mL), dried over Na_2SO_4, filtered and concentrated under reduced pressure. Purification by flash chromatography (30% EtOAc/hexane) afforded 3-Diazo-1-(4-methoxybenzyl)indolin-2-one **S25** (494 mg, 59%) as an orange solid. R_f 0.36 (30% EtOAc/hexane); ν_{max} (film)/cm^{-1} 3056, 2997, 2933, 2833, 2084 (C=N=N out-of-phase), 1674 (C=O), 1610, 1513, 1465, 1398, 1338, 1301, 1245, 1200, 1167, 1103, 1029, 842, 809, 738; ^{1}H NMR (400 MHz, $CDCl_3$) δ 7.26 (d, *J* = 8.7 Hz, 2 H, 2 × HC_{Ar}), 7.20–7.17 (m, 1 H, HC_{Ar}), 7.10 (td, *J* = 7.6, 1.4 Hz, 1 H, HC_{Ar}), 7.05 (td, *J* = 7.6, 1.2 Hz, 1 H, HC_{Ar}), 6.88 – 6.82 (m, 3 H, 3 × HC_{Ar}), 4.95 (s, 2 H, NCH_2Ar), 3.76 (s, 3 H, OCH_3); ^{13}C NMR (101 MHz, $CDCl_3$) δ 166.7 (C=O), 158.9 (OC_{Ar} quat), 133.5 (C_{Ar} quat), 128.6 (2 × C_{Ar}), 128.0 (C_{Ar} quat), 125.3 (C_{Ar}), 122.0 (C_{Ar}), 118.1 (C_{Ar}), 116.6 (C_{Ar} quat), 114.0 (2 × C_{Ar}), 109.4 (C_{Ar}), 60.7 (CN_2 quat), 55.1 (OCH_3), 43.6 (NCH_2Ar). The observed characterisation data (IR, ^{1}H and ^{13}C NMR) was consistent with that previously reported in the literature.[11]

SMILES: O=C1N(CC2=CC=C(OC)C=C2)C3=CC=CC=C3C1=[N+]=[N-]
InChI=1S/C16H13N3O2/c1-21-12-8-6-11(7-9-12)10-19-14-5-3-2-4-13(14)15(18-17)16(19)20/h2-9H,10H2,1H3

1-(4-Bromobenzyl)-3-diazoindolin-2-one (S26)

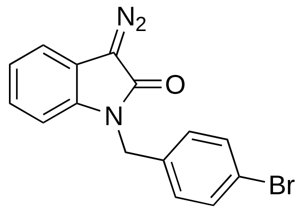

1-Bromo-4-(bromomethyl)benzene (2.00 g, 8.0 mmol) was added dropwise to a solution of 3-diazoindolin-2-one **S1** (636 mg, 4.0 mmol), TBAI (309 mg, 0.4 mmol) and K_2CO_3 (2.24 g, 16.0 mmol) in MeCN (8.0 mL) at 25 ºC. The reaction mixture was stirred for 19 h at 25 ºC. CH_2Cl_2 (50 mL) was added and the reaction mixture was extracted with sat. aq. $NaHCO_3$ (3 × 50 mL). The combined organic layers were dried over Na_2SO_4, filtered and concentrated under reduced pressure. Purification by flash chromatography (10% to 20% EtOAc/hexane) afforded 1-(4-Bromobenzyl)-3-diazoindolin-2-one **S26** (1.13 g, 86%) as an orange solid. R_f 0.30 (20% EtOAc/hexane); ν_{max} (film)/cm^{-1} 3056, 2930, 2084 (C=N=N out-of-phase), 1674 (C=O), 1607, 1484, 1435, 1398, 1338 (C=N=N in-phase), 1204, 1163, 1103, 1070, 1010, 850; ^{1}H NMR (400 MHz, $CDCl_3$) δ 7.44 (d, J = 8.4 Hz, 2 H, 2 × HC_{Ar}), 7.23–7.16 (m, 3 H, 3 × HC_{Ar}), 7.15–7.05 (m, 2 H, 2 × HC_{Ar}), 6.81–6.78 (m, 1 H, HC_{Ar}), 4.97 (s, 2 H, NCH_2Ar); ^{13}C NMR (101 MHz, $CDCl_3$) δ 166.7 (C=O), 135.0 (C_{Ar} quat), 133.3 (C_{Ar} quat), 131.8 (2 × C_{Ar}), 129.0 (2 × C_{Ar}), 125.4 (C_{Ar}), 122.3 (C_{Ar}), 121.6 (C_{Ar} quat), 118.3 (C_{Ar}), 116.7 (C_{Ar} quat), 109.3 (C_{Ar}), 60.8 (CN_2 quat), 43.6 (NCH_2Ar). The observed characterisation data (IR, ^{1}H and ^{13}C NMR) was consistent with that previously reported in the literature.[12]

SMILES: O=C1N(CC2=CC=C(Br)C=C2)C3=CC=CC=C3C1=[N+]=[N-]
InChI=1S/C15H10BrN3O/c16-11-7-5-10(6-8-11)9-19-13-4-2-1-3-12(13)14(18-17)15(19)20/h1-8H,9H2

1-(3,5-Bis(trifluoromethyl)benzyl)-3-diazoindolin-2-one (S27)

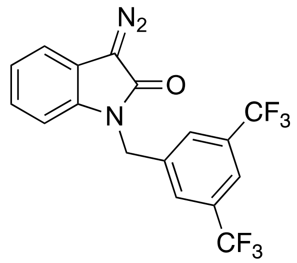

1-(Bromomethyl)-3,5-bis(trifluoromethyl)benzene (0.81 mL, 4.4 mmol) was added to a solution of 3-diazoindolin-2-one **S1** (637 mg, 4.0 mmol), TBAI (148 mg, 0.4 mmol) and K_2CO_3 (663mg, 4.8 mmol) in DMF (40 mL) at 0 ºC. The reaction mixture was stirred for 15 h at 25 ºC. Sat. aq. NaCl (100 mL) was added and extracted with EtOAc (3 × 100 mL). The combined organic layers were washed with sat. aq. NaCl (3 × 100 mL), dried over Na_2SO_4, filtered and concentrated under reduced pressure. Purification by flash chromatography (30% EtOAc/hexane) afforded 1-(3,5-bis(trifluoromethyl)benzyl)-3-diazoindolin-2-one **S27** (1.42 g, 92%) as an orange solid. R_f 0.32 (40% EtOAc/hexane); ν_{max} (film)/cm^{-1} 2084 (C=N=N out-of-phase), 1696 (C=O), 1379 (C=N=N in-phase), 1342, 1275, 1163, 1126, 1044, 1010, 962, 846, 734, 705; ^{1}H NMR (400 MHz, $CDCl_3$) δ 7.81 (s, 1 H, HC_{Ar}), 7.75 (s, 2 H, 2 × HC_{Ar}), 7.28–7.25 (m, 2 H, 2 × HC_{Ar}), 7.18–7.11 (m, 2 H, 2 × HC_{Ar}), 6.80–6.77 (m, 1 H, HC_{Ar}), 5.13 (s, 2 H, NCH_2Ar); ^{13}C NMR (101 MHz, $CDCl_3$) δ 166.7 (C=O), 138.8 (C_{Ar} quat), 132.9 (C_{Ar} quat), 132.2 (q, $J_{C\text{-}F}$ = 33.4 Hz, 2 x CF_3C_{Ar} quat), 127.4 (q, $J_{C\text{-}F}$ = 3.6 Hz, 2 x C_{Ar}), 125.6 (C_{Ar}), 123.2 (q, $J_{C\text{-}F}$ = 272.9 Hz, 2 x CF_3 quat), 122.7 (C_{Ar}), 121.9 (m, C_{Ar}), 118.6 (C_{Ar}), 116.8 (C_{Ar} quat), 108.9 (C_{Ar}), 61.1 (CN_2 quat), 43.5 (NCH_2Ar) ; ^{19}F{^{1}H} NMR (377 MHz, $CDCl_3$) δ –62.8; HRMS (FTMS + pAPC) *m/z* Calculated for $C_{17}H_{10}N_3OF_6$ $[M+H]^+$ 386.0723; Found 386.0718 .

SMILES: O=C1N(CC2=CC(C(F)(F)F)=CC(C(F)(F)F)=C2)C3=CC=CC=C3C1=[N+]=[N-]
InChI=1S/C17H9F6N3O/c18-16(19,20)10-5-9(6-11(7-10)17(21,22)23)8-26-13-4-2-1-3-12(13)14(25-24)15(26)27/h1-7H,8H2

3-Diazo-1-(naphthalen-2-ylmethyl)indolin-2-one (S28)

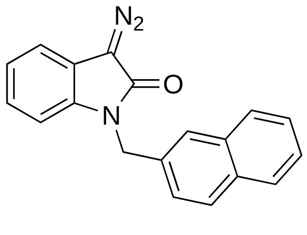

2-Bromonapthalene (243 mg, 1.1 mmol) in DMF (2 mL) was added to a solution of 3-diazoindolin-2-one **S1** (478 mg, 3.0 mmol), K_2CO_3 (829 mg, 6.0 mmol) in MeCN (6.0 mL) at 25 ºC. The reaction mixture was stirred for 16 h then CH_2Cl_2 (50 mL) was added and the organic layer was extracted with H_2O (3 × 50 mL). The combined organic layers were dried over Na_2SO_4, filtered and concentrated under reduced pressure. Purification by flash chromatography (30% to 40% Et_2O/pentane) afforded 3-diazo-1-(naphthalen-2-ylmethyl)indolin-2-one **S28** (593 mg, 66%) as an orange-red solid. R_f 0.13 (30% Et_2O/hexane); ν_{max} (film)/cm^{-1} 3054, 2085 (C=N=N out-of-phase), 1672 (C=O), 1608, 1467, 1375 (C=N=N in-phase), 1337, 1166, 1102, 740; ^{1}H NMR (400 MHz, $CDCl_3$) δ 7.84–7.75 (m, 4 H, 4 × HC_{Ar}), 7.51–7.43 (m, 3 H, 3 × HC_{Ar}), 7.24–7.19 (m, 1 H, HC_{Ar}), 7.10–7.03 (m, 2 H, 2 × HC_{Ar}), 6.90–6.85 (m, 1 H, HC_{Ar}), 5.20 (s, 2 H, NCH_2Ar); ^{13}C NMR (101 MHz, $CDCl_3$) δ 166.9 (C=O), 133.6 (NC_{Ar} quat), 133.5 (C_{Ar} quat), 133.2 (C_{Ar} quat), 132.8 (C_{Ar} quat), 128.7 (C_{Ar}), 127.72 (C_{Ar}), 127.66 (C_{Ar}), 126.3 (C_{Ar}), 126.1 (C_{Ar}), 126.0 (C_{Ar}), 125.4 (C_{Ar}), 125.2 (C_{Ar}), 122.2 (C_{Ar}), 118.2 (C_{Ar}), 116.7 (C_{Ar} quat), 109.6 (C_{Ar}), 44.5 (NCH_2Ar); HRMS (ESI$^+$) *m/z* Calculated for $C_{19}H_{14}N_3O$ [M+H] 300.1137; Found 300.1132.

SMILES: O=C1N(CC2=CC=C(C=CC=C3)C3=C2)C4=CC=CC=C4C1=[N+]=[N-]
InChI=1S/C19H13N3O/c20-21-18-16-7-3-4-8-17(16)22(19(18)23)12-13-9-10-14-5-1-2-6-15(14)11-13/h1-11H,12H2

3-Diazo-5,7-dimethylindolin-2-one (S29)

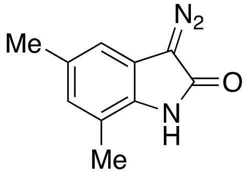

Tosylhydrazide (1.92 g, 11.0 mmol) in MeOH (10.0 mL) was heated to 70 ºC to solubilise the hydrazide. The mixture was cooled to rt and 5,7-dimethylisatin (1.75 g, 10.0 mmol) was added. The reaction mixture was stirred at 70 ºC for 20 min, then cooled to rt. The solid was filtered, then transferred to a new reaction flask. H_2O (100 mL) was added, then NaOH (0.8 g, 20.0 mmol) was added and the reaction mixture was stirred at 70 ºC for 3 h. The aqueous reaction mixture was extracted with EtOAc (3 × 100 mL) and the combined organic layers were dried over Na_2SO_4, filtered and concentrated under reduced pressure to afford 3-diazo-5,7-dimethylindolin-2-one **S29** (1.00 g, 53%) as a dark orange solid which was used without further purification. R_f 0.55 (50% EtOAc/pentane); ν_{max} (film)/cm^{-1} 3124 (NH), 3008, 2907, 2818, 2087 (C=N=N out-of-phase), 1670 (C=O), 1461, 1431, 1379 (C=N=N in-phase), 1327, 1200, 1096, 1036, 962, 895, 772, 705; ^{1}H NMR (400 MHz, DMSO-*d6*) δ 10.59 (br s, 1 H, NH), 7.03 (s, 1 H, HC_{Ar}), 6.72 (s, 1 H, HC_{Ar}), 2.23 (s, 1 H, $ArCH_3$), 2.18 (s, 1 H, $ArCH_3$); ^{13}C NMR (101 MHz, DMSO-*d6*) δ 168.3 (C=O), 130.2 (C_{Ar} quat), 129.0 (C_{Ar} quat), 127.3 (C_{Ar}), 119.1 (C_{Ar} quat), 117.1 (C_{Ar}), 116.6 (C_{Ar} quat), 60.1 (CN_2 quat), 20.7 ($ArCH_3$), 16.3 ($ArCH_3$); HRMS (ESI$^+$) *m/z* Calculated for $C_{12}H_{13}N_4O$ [M+CH_3CN+H] 229.1089; Found 229.1084.

SMILES: O=C1NC2=C(C)C=C(C)C=C2C1=[N+]=[N-]
InChI=1S/C10H9N3O/c1-5-3-6(2)8-7(4-5)9(13-11)10(14)12-8/h3-4H,1-2H3,(H,12,14)

1-Benzyl-3-diazo-5,7-dimethylindolin-2-one (S30)

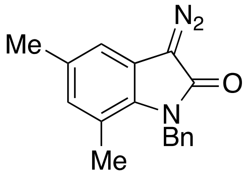

Benzyl bromide (0.95 mL, 8.0 mmol) was added to a solution of 3-diazo-5,7-dimethylindolin-2-one **S29** (749 mg, 4.0 mmol), K_2CO_3 (2.21 g, 16.0 mmol) and TBAI (300 mg, 0.8 mmol) in MeCN (8.0 mL) at 25 ºC. The reaction mixture was stirred for 19 h at 25 ºC. CH_2Cl_2 (100 mL) was added and the reaction mixture was extracted with sat. aq. $NaHCO_3$ (3 × 100 mL). The combined organic layers were dried over Na_2SO_4, filtered and concentrated under reduced pressure. Purification by flash chromatography (10% to 15% EtOAc/hexane; dry loaded with CH_2Cl_2) afforded 1-benzyl-3-diazo-5,7-dimethylindolin-2-one **S30** (899 mg, 81%) as an orange solid. R_f 0.13 (10% EtOAc/hexane); ν_{max} (film)/cm^{-1} 3062, 3029, 2919, 2864, 2085 (C=N=N out-of-phase), 1677 (C=O), 1592, 1470, 1446, 1418, 1392, 1380, 1347 (C=N=N in-phase), 1331, 1154, 11234, 842, 722, 697; ^{1}H NMR (400 MHz, $CDCl_3$) δ 7.33–7.28 (m, 2 H, 2 × HC_{Ph}), 7.26–7.21 (m, 1 H, HC_{Ph}), 7.14–7.11 (m, 2 H, 2 × HC_{Ph}), 6.91 (s, 1 H, HC_{Ar}), 6.68 (s, 1 H, HC_{Ar}), 5.29 (s, 2 H, NCH_2Ph), 2.32

(s, 3 H, $ArCH_3$), 2.27 (s, 3 H, $ArCH_3$); ^{13}C NMR (101 MHz, $CDCl_3$) δ 167.7 (C=O), 137.9 (C_{Ar} quat), 131.8 (C_{Ar} quat), 130.1 (C_{Ar}), 129.7 (C_{Ar} quat), 128.8 (2 × C_{Ph}), 127.1 (C_{Ph}), 125.6 (2 × C_{Ph}), 120.4 (C_{Ar} quat), 117.2 (C_{Ar} quat), 116.8 (C_{Ar}), 45.5 (NCH_2Ph), 28.8 ($ArCH_3$), 18.7 ($ArCH_3$). The observed characterisation data (IR, ^{1}H and ^{13}C NMR) was consistent with that previously reported in the literature.[8]

SMILES: O=C1N(CC2=CC=CC=C2)C3=C(C)C=C(C)C=C3C1=[N+]=[N-]
InChI=1S/C17H15N3O/c1-11-8-12(2)16-14(9-11)15(19-18)17(21)20(16)10-13-6-4-3-5-7-13/h3-9H,10H2,1-2H3

3-Diazo-4,6-difluoroindolin-2-one (S31)

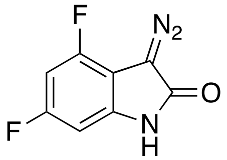

Tosylhydrazide (1.92 g, 11.0 mmol) in MeOH (10.0 mL) was heated to 70 ºC to solubilise the hydrazide. The mixture was cooled to rt and 4,6-difluoroisatin (1.83 g, 10.0 mmol) was added. The reaction mixture was stirred at 70 ºC for 10 min, then cooled to rt. The solid was filtered, then transferred to a new reaction flask. H_2O (100 mL) was added, then NaOH (0.8 g, 20.0 mmol) was added and the reaction mixture was stirred at 70 ºC for 1 h. The aqueous reaction mixture was extracted with EtOAc (3 × 100 mL) and the combined organic layers were dried over Na_2SO_4, filtered and concentrated under reduced pressure to afford 3-diazo-4,6-difluoroindolin-2-one **S31** (1.21 g, 62%) as an orange solid which was used without further purification. R_f 0.40 (60% EtOAc/hexane); ν_{max} (film)/cm^{-1} 3079 (br s, NH), 2851, 2788, 2747, 2099 (C=N=N out-of-phase), 1674 (C=O), 1636, 1607, 1513, 1454, 1402, 1342 (C=N=N in-phase), 1252, 1167, 1118, 1066, 984, 783, 734; ^{1}H NMR (400 MHz, DMSO-d_6) δ 11.10 (br s, 1 H, NH), 6.97–6.89 (m, 1 H, HC_{Ar}), 6.71–6.67 (m, 1 H, HC_{Ar}); ^{13}C NMR (101 MHz, DMSO-d_6) δ 167.4 (C=O), 160.4 (dd, J_{C-F} = 241.9, 12.4 Hz, FC_{Ar} quat), 154.8 (dd, J_{C-F} = 244.2, 14.8 Hz, FC_{Ar} quat), 135.0 (dd, J_{C-F} = 14.3, 10.7 Hz, NC_{Ar} quat), 100.0 (d, J_{C-F} = 19.5 Hz, C_{Ar} quat), 96.7 (dd, J_{C-F} = 27.6, 23.7 Hz, C_{Ar}), 95.1 (d, J_{C-F} = 27.8 Hz, C_{Ar}), 58.4 (CN_2 quat); ^{19}F{^{1}H} NMR (377 MHz, DMSO-d_6) δ –113.2, –122.4; HRMS (ESI$^-$) *m/z* Calculated for $C_8H_2N_3OF_2$ [M–H] 194.0166; Found 194.0172.

SMILES: O=C1NC2=CC(F)=CC(F)=C2C1=[N+]=[N-]
InChI=1S/C8H3F2N3O/c9-3-1-4(10)6-5(2-3)12-8(14)7(6)13-11/h1-2H,(H,12,14)

1-Benzyl-3-diazo-4,6-difluoroindolin-2-one (S32)

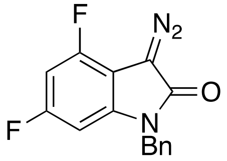

Benzyl bromide (0.95 mL, 8.0 mmol) was added to a solution of 3-diazo-4,6-difluoroindolin-2-one **S31** (780 mg, 4.0 mmol), K_2CO_3 (2.21 g, 16.0 mmol) and TBAI (300 mg, 0.8 mmol) in MeCN (8.0 mL) at 25 ºC. The reaction mixture was stirred for 19 h at 25 ºC. CH_2Cl_2 (100 mL) was added and the reaction mixture was extracted with sat. aq. $NaHCO_3$ (3 × 100 mL). The combined organic layers were dried over Na_2SO_4, filtered and concentrated under reduced pressure. Purification by flash chromatography (10% EtOAc/hexane) afforded 1-benzyl-3-diazo-4,6-difluoroindolin-2-one **S32** (907 mg, 79%) as an orange solid. R_f 0.20 (10% EtOAc/hexane); ν_{max} (film)/cm^{-1} 3086, 3066, 3031, 2925, 2093 (C=N=N out-of-phase), 1687 (C=O), 1643, 1599, 1498, 1461, 1437, 1402, 1388 (C=N=N in-phase), 1356, 1319, 1297, 1216, 1180, 1073, 1094, 1073, 1029, 955, 929, 824, 730, 696, 669; ^{1}H NMR (400 MHz, $CDCl_3$) δ 7.37–7.26 (m, 5 H, 5 × HC_{Ph}), 6.60–6.50 (m, 1 H, HC_{Ar}), 6.45–6.41 (m, 1 H, HC_{Ar}), 4.99 (s, 2 H, NCH_2Ph); ^{13}C NMR (101 MHz, $CDCl_3$) δ 166.7 (C=O), 162.2 (dd, J_{C-F} = 245.3, 12.0 Hz, FC_{Ar} quat), 155.2 (dd, J_{C-F} = 246.7, 14.2 Hz, FC_{Ar} quat), 135.5 (dd, J_{C-F} = 13.2, 10.4 Hz, NC_{Ar} quat), 135.1 (C_{Ph} quat), 128.9 (2 × C_{Ph}), 128.0 (C_{Ph}), 127.2 (2 × C_{Ph}), 99.4 (dd, J_{C-F} = 20.0, 3.0 Hz, C_{Ar} quat), 97.6 (dd, J_{C-F} = 27.5, 23.1 Hz, C_{Ar}), 94.7 (dd, J_{C-F} = 28.2, 3.5 Hz, C_{Ar}), 59.1 (CN_2 quat), 44.7 (NCH_2Ph); ^{19}F{^{1}H} NMR (377 MHz, $CDCl_3$) δ –111.96 (d, J_{F-F} = 4.5 Hz), –121.68 (d, J_{F-F} = 4.5 Hz); HRMS (ESI$^+$) *m/z* Calculated for $C_{15}H_{10}N_3OF_2$ [M+H] 286.0792; Found 286.0804.

SMILES: O=C1N(CC2=CC=CC=C2)C3=CC(F)=CC(F)=C3C1=[N+]=[N-]
InChI=1S/C15H9F2N3O/c16-10-6-11(17)13-12(7-10)20(15(21)14(13)19-18)8-9-4-2-1-3-5-9/h1-7H,8H2

Protected Amine Starting Materials

tert-Butyl (2-chloroethyl)carbamate (S33)

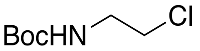

Triethylamine (4.70 mL, 33.7 mmol) was added dropwise to a stirred mixture of 2-chloroethylamine hydrochloride (3.00 g, 25.9 mmol) in CH_2Cl_2 (50 mL), and was subsequently cooled to 0 ºC. Di-*tert*-butyl dicarbonate (6.22 g, 28.5 mmol) was added portionwise carefully, then the solution was allowed to warm to 25 ºC for 18 h. H_2O (100 mL) and $CHCl_3$ (150 mL) were added, the organic and aqueous layers were separated, and the aqueous layer was extracted with further $CHCl_3$ (2 × 150 mL). The combined organic layers were then dried over Na_2SO_4, and the resulting solution was concentrated under reduced pressure. Purification by flash chromatography (15% EtOAc/pentane) afforded *tert*-butyl (2-chloroethyl)carbamate **S33** (4.60 g, 99%) as a white solid. R_f 0.79 (20% EtOAc/pentane); mp = 27–28 ºC; ν_{max} (film)/cm^{-1} 3343 (NH), 2979, 1686 (C=O), 1511, 1366, 1247, 1162, 657; ^{1}H NMR (400 MHz, $CDCl_3$) δ 5.05 (br s, 1 H, NH), 3.63–3.53 (t, *J* = 5.7 Hz, 2 H, CH_2Cl), 3.49–3.40 (q, *J* = 5.7 Hz, 2 H, NCH_2), 1.43 (s, 9 H, $C(CH_3)_3$); ^{13}C NMR (101 MHz, $CDCl_3$) δ 155.6 (C=O), 79.7 ($C(CH_3)_3$), 44.2 (CH_2Cl), 42.4 (NCH_2), 28.3 ($C(CH_3)_3$). The observed characterisation data (IR, ^{1}H and ^{13}C NMR) was consistent with that previously reported in the literature.[13]

SMILES : ClCCNC(OC(C)(C)C)=O
InChI=1S/C7H14ClNO2/c1-7(2,3)11-6(10)9-5-4-8/h4-5H2,1-3H3,(H,9,10)

tert-Butyl (2-bromoethyl)carbamate (S34)

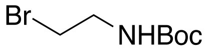

Triethylamine (2.09 mL, 15.0 mmol) was added dropwise to a stirred mixture of 2-bromoethylamine hydrobromide (2.25 g, 11.0 mmol) in CH_2Cl_2 (80 mL), and was subsequently cooled to 0 ºC. Di-*tert*-butyl dicarbonate (2.18 g, 10.0 mmol) was added portionwise, then the solution was warmed to 25 ºC for 18 g. Sat. aq. NH_4Cl (100 mL) and CH_2Cl_2 (100 mL) were added and the layers were separated. The organic layer was washed. with sat. aq. NH_4Cl (100 mL) then sat. aq. $NaHCO_3$ (2 × 100 mL) and sat. aq. brine (2 × 100 mL), dried over Na_2SO_4, filtered and concentrated under reduced pressure. Purification by flash chromatography (0 to 20% EtOAc/hexane) afforded *tert*-butyl (2-bromoethyl)carbamate **S34** (1.43 g, 64%) as a white solid with a low melting point. R_f 0.16 (10% EtOAc/hexane); ν_{max} (film)/cm^{-1} 3347 (br s, NH), 2976, 1687 (C=O), 1508, 1365, 1269, 1248, 1159; ^{1}H NMR (400 MHz, $CDCl_3$) δ 5.16 (br s, 1 H, NH), 3.50–3.35 (m, 4 H, NCH_2CH_2), 1.38 (s, 9 H, $C(CH_3)_3$); ^{13}C NMR (101 MHz, $CDCl_3$) δ 155.5 (C=O), 79.5 ($C(CH_3)_3$), 42.2 (NCH_2), 32.4 (CH_2Br), 28.2 ($C(CH_3)_3$). The observed characterisation data (IR, ^{1}H and ^{13}C NMR) was consistent with that previously reported in the literature.[14]

SMILES : BrCCNC(OC(C)(C)C)=O
InChI=1S/C7H14BrNO2/c1-7(2,3)11-6(10)9-5-4-8/h4-5H2,1-3H3,(H,9,10)

tert-Butyl (2-hydroxyethyl)carbamate (S35)

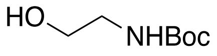

Ethanolamine (3.02 mL, 50.0 mmol) was added to a solution of di-*tert*-butyl dicarbonate (12.0 g, 55.0 mmol) in CH_2Cl_2 (100 mL) at 0 ºC. The reaction mixture was stirred at 25 ºC for 16 h then CH_2Cl_2 (100 mL) and sat. aq. $NaHCO_3$ (100 mL) were added and the layers separated. The organic layer was washed with sat. aq. $NaHCO_3$ (2 × 100 mL), dried over Na_2SO_4 and concentrated under reduced pressure to afford *tert*-butyl (2-hydroxyethyl)carbamate **S35** (6.11 g, 76%) as a colourless oil which was used without further purification. R_f 0.16 (60% EtOAc/hexane); ν_{max} (film)/cm^{-1} 3355 (br s, NH), 2976, 2934, 1685 (C=O), 1509, 1457, 1366, 1274, 1249, 1165, 1118, 1064, 1000, 971, 865, 779; ^{1}H NMR (400 MHz, $CDCl_3$) δ 5.17 (br s, 1 H, NH), 3.69–3.62 (m, 2 H, OCH_2), 3.29–3.20 (m, 3 H, NCH_2 and OH), 1.42 (s, 9 H, $C(CH_3)_3$); ^{13}C NMR (101 MHz, $CDCl_3$) δ 156.8 (C=O), 79.6 ($C(CH_3)_3$), 62.3 (OCH_2), 43.0 (NCH_2), 28.3 ($C(CH_3)_3$). The observed characterisation data (^{1}H and ^{13}C NMR) was consistent with that previously reported in the literature.[15]

SMILES : OCCNC(OC(C)(C)C)=O
InChI=1S/C7H15NO3/c1-7(2,3)11-6(10)8-4-5-9/h9H,4-5H2,1-3H3,(H,8,10)

tert-Butyl (2-iodoethyl)carbamate (S36)

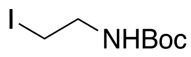

tert-Butyl (2-hydroxyethyl)carbamate **S35** (806 mg, 5.0 mmol) in CH_2Cl_2 (10 mL) was added dropwise to a stirred solution of PPh_3 (1.31 g, 5.0 mmol), I_2 (1.52 g, 6.0 mmol) and imidazole (343 mg, 5.0 mmol) in CH_2Cl_2 (40 mL) at 25 ºC. The reaction mixture was stirred at 25 ºC for 2.5 h then H_2O (50 mL) was added and the layers separated. The organic layer was washed with sat. aq. sodium thiosulfate (2 × 50 mL), sat. brine (50 mL), dried over Na_2SO_4 and concentrated under reduced pressure. Purification by flash chromatography (50% to 100% CH_2Cl_2/pentane) afforded *tert*-butyl (2-iodoethyl)carbamate **S36** (880 mg, 65%) as a pale yellow solid. R_f 0.28 (50% CH_2Cl_2/pentane); mp = 42–44 ºC [lit mp = 42–43 ºC][16]; ν_{max} (film)/cm^{-1} 3350 (br s, NH), 2977, 1677 (C=O), 1513, 1362, 1297, 1270, 1192, 1158, 1135, 1045, 1025, 933, 919, 861, 784; ^{1}H NMR (400 MHz, $CDCl_3$) δ 4.94 (br s, 1 H, NH), 3.49 (q, *J* = 6.3 Hz, 2 H, NCH_2), 3.24 (t, *J* = 6.3 Hz, 2 H, ICH_2), 1.45 (s, 9 H, $C(CH_3)_3$); ^{13}C NMR (101 MHz, $CDCl_3$) δ 155.4 (C=O), 79.8 ($\mathit{C}(CH_3)_3$), 42.9 (NCH_2), 28.3 ($C(\mathit{C}H_3)_3$), 6.0 (ICH_2). The observed characterisation data (^{1}H and ^{13}C NMR) was consistent with that previously reported in the literature.[16]

SMILES : ICCNC(OC(C)(C)C)=O
InChI=1S/C7H14INO2/c1-7(2,3)11-6(10)9-5-4-8/h4-5H2,1-3H3,(H,9,10)

Benzyl (2-chloroethyl)carbamate (S37)[17]

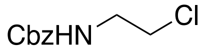

Benzyl chloroformate (3.14 mL, 22.0 mmol) was added dropwise over 5 min to a stirred solution of 2-chloroethylamine hydrochloride (2.32 g, 20.0 mmol) in aq. NaOH (10% in H_2O, 8.0 mL) at 0 °C. Further aq. NaOH (10%, 8.0 mL) was added and the temperature raised to 25 °C. After 24 h, the reaction mixture was diluted with CH_2Cl_2 (40 mL) and the aqueous and organic layers were separated. The aqueous layer was extracted further with CH_2Cl_2 (2 × 40 mL). The combined organic layers were dried over Na_2SO_4, filtered and concentrated under reduced pressure. Purification by flash chromatography (20% EtOAc/pentane) afforded benzyl (2-chloroethyl)carbamate **S37** (3.97 g, 93%) as a colourless oil. R_f 0.33 (20% EtOAc/pentane); ν_{max} (film)/cm^{-1} 3327 (br, NH), 3034, 2960, 1695 (C=O), 1517, 1242, 1140, 1063, 996, 737, 696; ^{1}H NMR (400 MHz, $CDCl_3$) δ 7.43–7.30 (m, 5 H, 5 × HC_{Ph}), 5.25–5.10 (m, 3 H, NH and OCH_2Ph), 3.69–3.49 (m, 4 H, $N(CH_2)_2$); ^{13}C NMR (101 MHz, $CDCl_3$) δ 156.2 (C=O), 136.2 (C_{Ph} quat), 128.5 (2 × C_{Ph}), 128.2 (C_{Ph}), 128.1 (2 × C_{Ph}), 67.0 (OCH_2), 44.1 (CH_2Cl), 42.8 (NCH_2). The observed characterisation data (^{1}H and ^{13}C NMR) was consistent with that previously reported in the literature.[18]

SMILES: ClCCNC(OCC1=CC=CC=C1)=O
InChI=1S/C10H12ClNO2/c11-6-7-12-10(13)14-8-9-4-2-1-3-5-9/h1-5H,6-8H2,(H,12,13)

Methyl (2-chloroethyl)carbamate (S38)

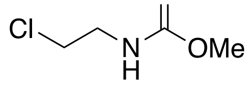

Methyl chloroformate (0.77 mL, 10.0 mmol) was added dropwise to a solution of 2-chloroethylamine hydrochloride (1.28 g, 11.0 mmol) and triethylamine (3.1 mL, 22.0 mmol) in CH_2Cl_2 (50 mL) at 0 ºC. The reaction mixture was stirred for 17 h at 25 ºC, then sat. aq. NH_4Cl (50 mL) was added and the aqueous reaction mixture was extracted with CH_2Cl_2 (3 × 50 mL). The combined organic layers were dried over Na_2SO_4, filtered and concentrated under reduced pressure. Purification by flash chromatography (30% Et_2O/pentane) afforded methyl (2-chloroethyl)carbamate **S38** (881 mg, 64%) as a colourless oil. R_f 0.14 (20% Et_2O/pentane); ν_{max} (film)/cm^{-1} 3332 (br s, NH), 2958, 1696 (C=O carbamate), 1523, 1437, 1247, 1194, 1067, 1010, 779; ^{1}H NMR (400 MHz, $CDCl_3$) δ 5.23 and 5.08 (2 × br s, 1 H, NH), 3.75–3.65 (br s, 3 H, OCH_3), 3.60 (br t, *J* = 5.6 Hz, CH_2Cl), 3.51 (br q, *J* = 5.6 Hz, NCH_2); ^{13}C NMR (101 MHz, $CDCl_3$) δ 156.9 (C=O), 52.3 (OCH_3), 44.0 (NCH_2), 42.8 (CH_2Cl). The observed characterisation data (IR and ^{1}H NMR) was consistent with that previously reported in the literature.[19]

SMILES: ClCCNC(OC)=O
InChI = 1S/C4H8ClNO2/c1-8-4(7)6-3-2-5/h2-3H2,1H3,(H,6,7)

tert-Butyl (3-chloropropyl)carbamate (S39)

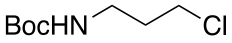

Triethylamine (8.78 mL, 63.0 mmol) was added dropwise to a stirred mixture of 3-chloropropylamine hydrochloride (3.90 g, 30.0 mmol) in CH_2Cl_2 (60 mL) and stirred at 0 °C for 30 min. Di-*tert*-butyl dicarbonate (7.20 g, 33 mmol) was added to the stirred solution at 0 °C. After 5 min, the temperature was raised to 25 °C and stirred for 21 h. H_2O (100 mL) and $CHCl_3$ (100 mL) were added, the organic and aqueous layers were separated, and the aqueous layer was extracted with further $CHCl_3$ (2 × 100 mL). The combined organic layers were then dried over Na_2SO_4, and the resulting solution was concentrated under reduced pressure. Purification by flash chromatography (20 % EtOAc/pentane) afforded *tert*-butyl (3-chloroproyl)carbamate **S39** (5.90 g, quant) as a white solid. R_f 0.42 (20% EtOAc/pentane); mp = 29–30 ºC (lit. = 32 ºC)[20]; ν_{max} (film)/cm^{-1} 3337 (br, NH), 2978, 1685 (C=O), 1518, 1366, 1251, 1164, 1119, 1074; ^{1}H NMR (400 MHz, $CDCl_3$) δ 4.68 (s, 1 H, NH), 3.59 (t, J = 6.4 Hz, 2 H, CH_2Cl), 3.28 (q, J = 6.4 Hz, 2 H, NCH_2), 1.97 (t, J = 6.4 Hz, 2 H, NCH_2CH_2), 1.44 (s, 9H, $C(CH_3)_3$); ^{13}C NMR (101 MHz, $CDCl_3$) δ 155.9 (C=O), 79.4 ($C(CH_3)_3$), 42.4 (CH_2Cl), 37.9 (NCH_2), 32.6 (NCH_2CH_2), 28.4 ($C(CH_3)_3$). The observed characterisation data (^{1}H and ^{13}C NMR) was consistent with that previously reported in the literature.[21]

NH–Insertion Intermediates

General Procedure **A**

A solution of diazo (1.0 equiv) in CH_2Cl_2 or toluene (0.16 M) was slowly added over 10 min to a stirred solution of the appropriate protected amine (2.0 equiv) in CH_2Cl_2 or toluene (0.3 M) at 25 ºC. The remaining diazo solution was washed with CH_2Cl_2 or toluene (1 M) to ensure quantitative transfer. After 30 min the reaction mixture was concentrated under reduced pressure. Purification by flash chromatography afforded the NH insertion product.

3-(3-Bromopropoxy)-1-methylindolin-2-one (S40)

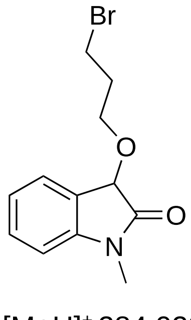

Purification by flash chromatography (0 to 10% MeCN/CH_2Cl_2) afforded 3-(3-bromopropoxy)-1-methylindolin-2-one **S40** as a yellow/orange oil*.* R_f 0.27 (50% EtOAc/heptane); ν_{max} (film)/cm^{-1} 2923, 2868, 1707 (C=O), 1612, 1469, 1351, 1111, 1054, 754 ; ^{1}H NMR (400 MHz, $CDCl_3$) δ 7.42–7.41 (m, 1 H, HC_{Ar}), 7.35 (tdd, *J* = 7.7, 1.2, 0.4 Hz, 1 H, HC_{Ar}), 7.11 (td, *J* = 7.6, 0.9 Hz, 1 H, HC_{Ar}), 6.82 (d, 7.8 Hz, 1 H, HC_{Ar}), 4.91 (s, 1 H, OCH), 4.00–3.95 (m, 1 H, OC*H*H), 3.77–3.72 (m, 1 H, OCH*H*), 3.57 (t, *J* = 6.6 Hz, 2 H, CH_2Br), 3.19 (s, 3 H, NCH_3), 2.21–2.15 (m, 2 H, OCH_2CH_2); ^{13}C NMR (101 MHz, $CDCl_3$) δ 174.4 (C=O), 144.2 (NC_{Ar} quat), 130.0 (C_{Ar}), 125.3 (C_{Ar}), 124.9 (C_{Ar} quat), 123.0 (C_{Ar}), 108.4 (C_{Ar}), 76.0 (OCH), 66.4 (OCH_2), 32.9 (OCH_2CH_2), 30.3 (NCH_3), 26.0 (CH_2Br); HRMS (ESI^+) *m/z* Calculated for $C_{12}H_{15}NO_2Br$ $[M+H]^+$ 284.0286; Found 284.0294.

SMILES: O=C1N(C)C2=CC=CC=C2C1OCCCBr
InChI=1S/C12H14BrNO2/c1-14-10-6-3-2-5-9(10)11(12(14)15)16-8-4-7-13/h2-3,5-6,11H,4,7-8H2,1H3

tert-Butyl (3-chloropropyl)(1-methyl-2-oxoindolin-3-yl)carbamate (S41)

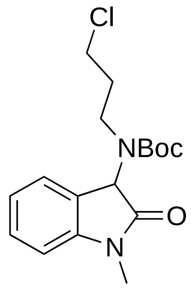

A solution of diazo **S24** (66 mg, 0.38 mmol) in CH_2Cl_2 (0.9 mL) was slowly added over 10 min to a stirred solution of protected amine **S39** (48 mg, 0.25 mmol) and $Rh_2(esp)_2$ (0.9 mg, 1.25 μmol) in CH_2Cl_2 (0.67 mL) at 40 ºC. The remaining solution of diazo **S24** was washed with CH_2Cl_2 (0.1 mL) to ensure quantitative transfer. After 2 h at 40 ºC the reaction mixture was concentrated under reduced pressure. Purification by flash chromatography (heptane to 30% EtOAc/heptane) afforded *tert*-butyl (3-chloropropyl)(1-methyl-2-oxoindolin-3-yl)carbamate **S41** (41 mg, 48%) as a brown oil. R_f 0.20 (30% EtOAc/heptane); ν_{max} (film)/cm^{-1} 2984, 2954, 2928, 1720 (C=O amide), 1689 (C=O carbamate), 1609, 1495, 1471, 1457, 1417, 1362, 1375, 1353, 1336, 1282, 1154, 1121, 1084, 766; ^{1}H NMR (400 MHz, DMSO-d_6, 373 K) δ 7.31 (tt, *J* = 7.7, 1.1 Hz, 1 H, HC_{Ar}), 7.22–7.19 (m, 1 H, HC_{Ar}), 7.05 (td, *J* = 7.6, 0.9 Hz, 1 H, HC_{Ar}), 6.96 (d, *J* = 7.8 Hz, 1 H, HC_{Ar}), 5.01 (br s, 1 H, NCH), 3.76–3.66 (m, 2 H, CH_2Cl), 3.64–3.39 (m, 2 H, NCH_2), 3.13 (s, 3 H, NCH_3), 2.13–2.02 (m, 2 H, NCH_2CH_2), 1.29–1.07 (br s, 9 H, $C(CH_3)_3$); ^{13}C NMR (101 MHz, DMSO-d_6, 373 K) δ 173.2 (C=O amide), 153.4 (C=O carbamate), 143.1 (NC_{Ar} quat), 127.9 (C_{Ar}), 126.4 (C_{Ar} quat), 122.0 (C_{Ar}), 121.4 (C_{Ar}), 107.7 (C_{Ar}), 79.2 ($C(CH_3)_3$), 59.4 (NCH), 45.6 (NCH_2), 42.0 (CH_2Cl), 31.6 (NCH_2CH_2), 27.1 ($C(CH_3)_3$), 25.4 (NCH_3); HRMS (ESI^+) *m/z* Calculated for $C_{17}H_{23}N_2O_3{}^{35}Cl$ [M+Na] 361.1295; Found 361.1295.

SMILES: O=C1N(C)C2=CC=CC=C2C1N(CCCCl)C(OC(C)(C)C)=O
InChI=1S/C17H23ClN2O3/c1-17(2,3)23-16(22)20(11-7-10-18)14-12-8-5-6-9-13(12)19(4)15(14)21/h5-6,8-9,14H,7,10-11H2,1-4H3

tert-Butyl (1-benzyl-2-oxoindolin-3-yl)(2-chloroethyl)carbamate (2a)

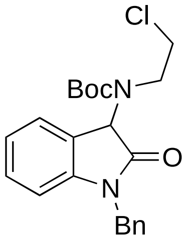

Following general procedure **A** on 1.0 mmol scale using diazo **1** and protected amine **S33** in toluene. Purification by flash chromatography (25% Et_2O/pentane) afforded *tert*-butyl (1-benzyl-2-oxoindolin-3-yl)(2-chloroethyl)carbamate **2a** (264 mg, 66%) as a pale orange solid. R_f 0.10 (25% Et_2O/hexane); mp = 112–114 ºC; ν_{max} (film)/cm^{-1} 2978, 1703 (2 × C=O), 1608, 1485, 1447, 1365, 1334, 1241, 1156, 738, 701; ^{1}H NMR (400 MHz, DMSO-d_6) δ 7.46–7.14 (m, 7 H, 7 HC_{Ar}), 7.07–6.93 (m, 2 H, 2 × HC_{Ar}), 5.26 (s, 1 H, NCH), 5.11–4.60 (m, 2 H, NCH_2Ph), 4.00–3.70 (m, 4 H, $N(CH_2)_2$), 1.38 and 0.87 (2 × s, 9 H, $C(CH_3)_3$); ^{13}C NMR (101 MHz, DMSO-d_6) δ 174.2 (C=O amide), 153.5 (C=O carbamate), 142.3 (NC_{Ar} quat), 136.5 (C_{Ar} quat), 128.5, 128.4, 128.1, 127.7, 127.5, 127.2 (6 × C_{Ar} and C_{Ar} quat), 122.6 (C_{Ar}), 122.1 (C_{Ar}), 108.8 (C_{Ar}), 79.9 ($C(CH_3)_3$), 60.2 (NCH), 50.7 (NCH_2), 42.9 (NCH_2Ph), 41.9 (CH_2Cl), 27.8 and 27.2 ($C(CH_3)_3$); HRMS (ESI$^+$) *m/z* Calculated for $C_{24}H_{28}N_3O_3NaCl$ [M+CH_3CN+Na] 464.1717; Found 464.1716.

SMILES: O=C1N(CC2=CC=CC=C2)C3=CC=CC=C3C1N(CCCl)C(OC(C)(C)C)=O
InChI=1S/C22H25ClN2O3/c1-22(2,3)28-21(27)24(14-13-23)19-17-11-7-8-12-18(17)25(20(19)26)15-16-9-5-4-6-10-16/h4-12,19H,13-15H2,1-3H3

tert-Butyl (1-benzyl-2-oxoindolin-3-yl)(2-bromoethyl)carbamate (2b)

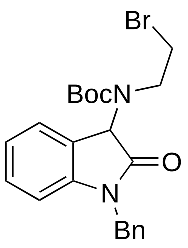

Following general procedure **A** on 1.0 mmol scale using diazo **1** and protected amine **S34** in toluene. Purification by flash chromatography (30% Et_2O/pentane) afforded *tert*-butyl (1-benzyl-2-oxoindolin-3-yl)(2-bromoethyl)carbamate **2b** (263 mg, 59%) as a white solid. R_f 0.18 (30% Et_2O/pentane); mp = 113–115 ºC; ν_{max} (film)/cm^{-1} 2974, 1696 (br s, 2 × C=O), 1613, 1467, 1454, 1364, 1149, 747; ^{1}H NMR (400 MHz, $CDCl_3$) δ 7.42–7.17 (m, 7 H, 7 × HC_{Ar}), 7.09–7.00 (m, 1 H, HC_{Ar}), 6.81–6.71 (m, 1 H, HC_{Ar}), 5.27–4.78 and 4.59–4.45 (m, 2 H, NCH_2Ph), 4.75–4.62 (m, 1 H, NCH), 4.11-3.93 (br s, 1 H, NC*H*H), 3.77–3.62 (m, 1 H, NCH*H*), 3.60–3.32 (m, 2 H, CH_2Br), 1.53 and 0.97 (2 × br s, 9 H, $C(CH_3)_3$); ^{13}C NMR (101 MHz, $CDCl_3$) δ 174.2 and 173.7 (C=O amide), 154.9 and 153.7 (C=O carbamate), 142.8 and 142.5 (NC_{Ar} quat), 135.7 and 135.4 (C_{Ph} quat), 129.3, 128.8, 128.6 (2 × C_{Ph} and C_{Ar}), 127.8, 127.7 and 127.2 (3 × C_{Ph}), 125.3 and 124.2 (C_{Ar} quat), 122.9 and 122.6 (2 × C_{Ar}), 106.5 and 108.8 (C_{Ar}), 81.5 and 81.2 ($C(CH_3)_3$), 61.8 and 59.1 (NCH), 52.5 (NCH_2), 44.1 and 44.0 (NCH_2Ph), 30.0 and 28.7 (CH_2Br), 28.2 and 27.6 ($C(CH_3)_3$); HRMS (ESI$^+$) *m/z* Calculated for $C_{24}H_{28}N_3O_3Na^{79}Br$ [M+CH_3CN+Na] 508.1212; Found 508.1207.

SMILES O=C1N(CC2=CC=CC=C2)C3=CC=CC=C3C1N(CCBr)C(OC(C)(C)C)=O
InChI=1S/C22H25BrN2O3/c1-22(2,3)28-21(27)24(14-13-23)19-17-11-7-8-12-18(17)25(20(19)26)15-16-9-5-4-6-10-16/h4-12,19H,13-15H2,1-3H3

tert-Butyl (1-benzyl-2-oxoindolin-3-yl)(2-iodoethyl)carbamate (2c)

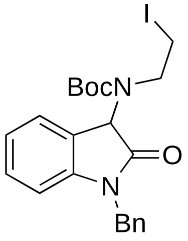

Following general procedure **A** on 0.88 mmol scale using diazo **1** and protected amine **S36** in toluene. Purificaton by flash chromatography (CH_2Cl_2 to 1% to 3% Et_2O/CH_2Cl_2 then 30% Et_2O/pentane) afforded *tert*-butyl (1-benzyl-2-oxoindolin-3-yl)(2-iodoethyl)carbamate **2c** (155 mg, 36%) as an off-white solid. R_f 0.45 (50% Et_2O/pentane); mp = 111–112 ºC; ν_{max} (film)/cm^{-1} 2974, 1696 (br s, 2 × C=O), 1613, 1487, 1467, 1453, 1407, 1364, 1281, 1148, 748, 697; ^{1}H NMR (400 MHz, $CDCl_3$) δ 7.43–7.19 (m, 7 H, 7 × HC_{Ar}), 7.10–7.01 (m, 1 H, HC_{Ar}), 6.81–6.72 (m, 1 H, HC_{Ar}), 5.74 (br s, 1 H, NCH rotamer), 5.29–4.97 (m, 1 H, NC*H*HPh), 4.90–4.75 and 4.70–4.42 (m, 1 H, NCH rotamer and NCH*H*Ph), 4.18–3.80 (2 × br s, 1 H, NC*H*H), 3.62–3.02 (m, 3 H, NCH*HCH₂*), 1.54 and 0.98 (2 × s, 9 H, $C(CH_3)_3$); ^{1}H NMR (400 MHz, DMSO-d_6) δ 7.46–7.15 (m, 7 H, 7 × HC_{Ar}), 7.07–6.92 and 6.86–6.81 (m, 2 H, 2 × HC_{Ar}), 5.26 (s, 1 H, NCH), 5.10–5.01, 4.98–4.83 and 4.68–4.60 (m, 2 H, NCH_2Ph), 3.94–3.78 (m, 1 H, NC*H*H), 3.49–3.29 (m, 3 H, NCHHCH_2), 1.39 and 0.87 (2 × br s, 9 H, $C(CH_3)_3$); ^{13}C NMR (101 MHz, $CDCl_3$) δ 174.0 and 173.7 (C=O amide), 153.6 (C=O carbamate), 142.8 and 142.6 (NC_{Ar} quat), 135.5 (C_{Ph} quat), 129.3, 128.9 (C_{Ar} and 2 × C_{Ph}), 127.8, 127.7 and 127.2 (3 × C_{Ph}), 125.3

and 124.2 (C_{Ar} quat), 123.0 and 122.6 (2 × C_{Ar}), 109.5 and 108.9 (C_{Ar}), 81.5 and 81.2 (*C*(CH_3)$_3$), 61.5 and 59.0 (NCH), 53.5 (NCH_2), 44.1 and 44.0 (NCH_2Ph), 28.3 and 27.6 (C(*C*H_3)$_3$), 1.6 (CH_2I); ^{13}C NMR (101 MHz, DMSO-*d*$_6$) δ 174.2 (C=O amide), 153.3 (C=O carbamate), 142.3 (NC_{Ar} quat), 136.5 and 136.3 (C_{Ph} quat), 128.6, 128.5, 128.1, 127.7, 127.4, 127.2 (5 × C_{Ph}, C_{Ar} and C_{Ar} quat), 122.7 (C_{Ar}), 122.2 and 122.1 (C_{Ar}), 108.8 (C_{Ar}), 80.4 and 79.9 (*C*(CH_3)$_3$), 59.9 (NCH), 51.7 and 51.6 (NCH_2), 42.9 and 42.8 (NCH_2Ph), 27.8 and 27.2 (C(*C*H_3)$_3$), 3.0 (CH_2I); HRMS (ESI$^+$) *m/z* Calculated for $C_{22}H_{26}N_2O_3{}^{127}I$ [M+H] 493.0988; Found 493.0992.

SMILES O=C1N(CC2=CC=CC=C2)C3=CC=CC=C3C1N(CCI)C(OC(C)(C)C)=O
InChI=1S/C22H25IN2O3/c1-22(2,3)28-21(27)24(14-13-23)19-17-11-7-8-12-18(17)25(20(19)26)15-16-9-5-4-6-10-16/h4-12,19H,13-15H2,1-3H3

tert-Butyl (1-benzyl-5-methoxy-2-oxoindolin-3-yl)(2-chloroethyl)carbamate (S42)

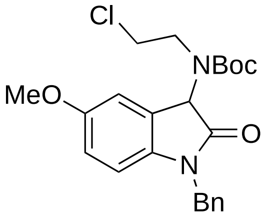

Following general procedure **A** on 1.0 mmol scale using diazo **S3** and protected amine **S33** in CH_2Cl_2. Purification by flash chromatography (20% to 25% to 30% EtOAc/pentane) afforded *tert*-butyl (1-benzyl-5-methoxy-2-oxoindolin-3-yl)(2-chloroethyl)carbamate **S42** (328 mg, 76%) as a white solid. R_f 0.13 (20% EtOAc/pentane); ν_{max} (film)/cm^{-1} 2973, 2930, 1697 (2 × C=O), 1602, 1491, 1454, 1364, 1333, 1435, 1406, 1279, 1247, 1176, 1151, 1081, 1062, 1035, 855, 807, 762, 727; ^{1}H NMR (400 MHz, $CDCl_3$) δ 7.44–7.22 (m, 5 H, 5 × HC_{Ph}), 6.97 and 6.91 (2 × br s, 1 H, HC_{Ar}), 6.87–6.70 (m, 2 H, 2 × HC_{Ar}), 5.22 (s, 1 H, NCH), 5.04 (d, *J* = 15.5 Hz, 0.5 H, NC*H*HPh), 4.90 and 4.84 (2 × d, *J* = 15.6 Hz, 1 H, NCH*H*Ph), 4.60 (d, *J* = 15.5 Hz, 0.5 H, NC*H*HPh), 3.98–3.65 (m, 7 H, N(CH_2)$_2$ and OCH_3), 1.38 and 0.89 (2 × s, 9 H, C(CH_3)$_3$); ^{13}C NMR (101 MHz, $CDCl_3$) δ 173.8 (C=O amide), 155.4 (OC_{Ar} quat), 153.5 (C=O carbamate), 136.6 and 136.3 (C_{Ar} quat), 135.8 and 135.7 (C_{Ar} quat), 129.1 (C_{Ar} quat), 128.5 and 128.4 (2 × C_{Ph}), 127.7, 127.4 and 127.2 (3 × C_{Ph}), 112.4 (C_{Ar}), 110.6 and 110.0 (C_{Ar}), 109.2 (C_{Ar}), 80.3 and 79.9 (*C*(CH_3)$_3$), 60.5 (NCH), 55.6 and 55.4 (OCH_3), 50.7 (NCH_2), 43.0 and 42.8 (NCH_2Ph), 42.0 (CH_2Cl), 27.8 and 27.3 (C(*C*H_3)$_3$); HRMS (FTMS – pAPCI) *m/z* Calculated for $C_{23}H_{26}N_2O_4{}^{35}Cl^-$ [M–H]$^-$ 429.1587; Found 429.1574.

SMILES O=C1N(CC2=CC=CC=C2)C3=CC=C(OC)C=C3C1N(CCCl)C(OC(C)(C)C)=O
InChI=1S/C23H27ClN2O4/c1-23(2,3)30-22(28)25(13-12-24)20-18-14-17(29-4)10-11-19(18)26(21(20)27)15-16-8-6-5-7-9-16/h5-11,14,20H,12-13,15H2,1-4H3

tert-Butyl (1-benzyl-4-methyl-2-oxoindolin-3-yl)(2-chloroethyl)carbamate (S43)

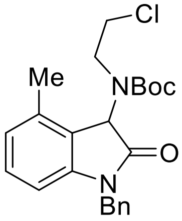

Following general procedure, **A** on 0.9 mmol scale using diazo **S4** and protected amine **S33** in CH_2Cl_2. Purification by flash chromatography (30% Et_2O/pentane) afforded *tert*-butyl (1-benzyl-5-methyl-2-oxoindolin-3-yl)(2-chloroethyl)carbamate **S43** (196 mg, 51%) as a brown solid. R_f 0.31 (20% Et_2O/hexane); mp = 99–118 ºC; νmax (film)/cm^{-1} 2973, 2924, 1713 (C=O amide), 1693 (C=O carbamate), 1601, 1466, 1442, 1351 1327, 1280, 1238, 1148, 777, 703; ^{1}H NMR (400 MHz, DMSO-*d*$_6$) δ 7.44 – 7.23 (m, 5 H, 5 × HC_{Ar}), 7.12 (d, *J* = 8.7 Hz, 1 H, HC_{Ar}), 6.81 (d, *J* = 7.3 Hz, 2 H, 2 × HC_{Ar}), 5.27 (s, 1 H, NCH), 5.05 – 4.62 (m, 2 H, NCH_2Ph), 4.18 – 3.70 (m, 2 H, NC*H*$_2$CH$_2$), 3.65 – 3.37 (m, 2H, NCH$_2$C*H*$_2$), 2.17 (d, *J* = 12.5 Hz, 3 H, ArCH_3), 1.39 and 0.84 (2 × s, 9 H, C(CH_3)$_3$); 13^C NMR (101 MHz, DMSO-*d*$_6$) δ 174.1 (C=O amide), 153.6 (C=O carbamate), 142.3 (NC_{Ar} quat), 136.6 (C_{Ar} quat), 133.2 (C_{Ar} quat), 128.7, 128.5, 128.0, 127.8, 127.5 and 127.2 (6 × C_{Ar}), 125.0 and 124.7 (C_{Ar} quat), 123.9 (C_{Ar}), 106.7 (C_{Ar}), 79.9 (*C*(CH_3)$_3$), 60.4 (NCH), 50.6 (NCH_2), 43.0 (NCH_2Ph), 40.8 and 40.6 (CH_2Cl), 27.8 and 27.1 (C(*C*H_3)$_3$), 17.6 (ArCH_3); HRMS (ESI$^+$) *m/z* Calculated for $C_{23}H_{28}{}^{35}ClN_2O_3$ [M+H]$^+$ 415.1783; Found 415.1785

SMILES O=C1N(CC2=CC=CC=C2)C3=CC=CC(C)=C3C1N(CCCl)C(OC(C)(C)C)=O
InChI=1S/C23H27ClN2O3/c1-16-9-8-12-18-19(16)20(25(14-13-24)22(28)29-23(2,3)4)21(27)26(18)15-17-10-6-5-7-11-17/h5-12,20H,13-15H2,1-4H3

tert-Butyl (1-benzyl-5-methyl-2-oxoindolin-3-yl)(2-chloroethyl)carbamate (S44)

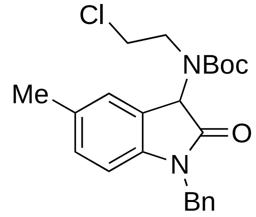

Following general procedure **A** on 0.9 mmol scale using diazo **S6** and protected amine **S33** in CH_2Cl_2. Purification by flash chromatography (30% Et_2O/pentane) afforded *tert*-butyl (1-benzyl-5-methyl-2-oxoindolin-3-yl)(2-chloroethyl)carbamate **S44** (196 mg, 51%) as a brown solid. R_f 0.19 (30% Et_2O/pentane); mp = 147–148 ºC; ν_{max} (film)/cm^{-1} 2973, 2924, 2869, 1702 (2 × C=O), 1495, 1415, 1365, 1334, 1244, 1154, 1132, 825, 741, 703; ^{1}H NMR (400 MHz, DMSO-d_6) δ 7.44–7.22 (m, 5 H, 5 × HC_{Ar}), 7.15–6.96 (m, 2 H, 2 × HC_{Ar}), 6.85–6.66 (m, 1 H, HC_{Ar}), 5.20 (s, 1 H, NCH), 5.04 and 4.91 (2 × d, *J* = 15.4 Hz, 1 H, NC*H*HAr), 4.83 and 4.60 (2 × d, *J* = 15.4 Hz, 1 H, NCH*H*Ar), 3.97–3.64 (m, 4 H, NCH_2CH_2), 2.27–2.22 (s, 3 H, $ArCH_3$), 1.39 and 0.88 (s, 9 H, $C(CH_3)_3$); ^{13}C NMR (101 MHz, DMSO-d_6) δ 174.0 (C=O amide), 153.5 (C=O carbamate), 139.9 (C_{Ar} quat), 136.5 (C_{Ar}), 131.1 (C_{Ar} quat), 128.5, 128.1, 127.6, 127.4 and 127.2 (4 × C_{Ar} and 2 × C_{Ar} quat), 123.3 (C_{Ar}), 108.8 and 108.5 (C_{Ar}), 80.3 and 79.8 ($C(CH_3)_3$), 60.3 (NCH), 50.7 (NCH_2), 42.9 and 42.8 (NCH_2Ar), 41.8 (CH_2Cl), 27.8 and 27.2 ($C(CH_3)_3$), 20.6 ($ArCH_3$); HRMS (ESI$^+$) *m/z* Calculated for $C_{23}H_{28}N_2O_3Cl$ [M+H] 415.1788; Found 415.1800.

SMILES: O=C1N(CC2=CC=CC=C2)C3=CC=C(C)C=C3C1N(C(OC(C)(C)C)=O)CCCl
InChI=1S/C23H27ClN2O3/c1-16-10-11-19-18(14-16)20(25(13-12-24)22(28)29-23(2,3)4)21(27)26(19)15-17-8-6-5-7-9-17/h5-11,14,20H,12-13,15H2,1-4H3

tert-Butyl (1-benzyl-5-fluoro-2-oxoindolin-3-yl)(2-chloroethyl)carbamate (S45)

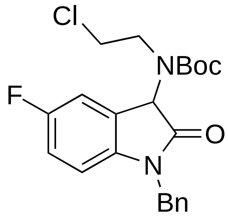

Following general procedure **A** on 1.0 mmol scale using diazo **S8** and protected amine **S33** in CH_2Cl_2. Purification by flash chromatography (30% Et_2O/pentane) afforded *tert*-butyl (1-benzyl-5-fluoro-2-oxoindolin-3-yl)(2-chloroethyl)carbamate **S45** (275 mg, 66%) as a off-white solid. R_f 0.26 (30% Et_2O/pentane); mp = 87-88 ºC; ν_{max} (film)/cm^{-1} 2974, 2930, 1700 (br s, 2 × C=O), 1487, 1454, 1409, 1364, 1334, 1290, 1249, 1156, 809, 772, 693; ^{1}H NMR (400 MHz, DMSO-d_6) δ 7.44–7.14 (m, 6 H, 6 × HC_{Ar}), 7.13–7.00 (m, 1 H, HC_{Ar}), 6.99–6.94 and 6.82–6.77 (2 × m, 1 H, HC_{Ar}), 5.31 (s, 1 H, NCH), 5.05 and 4.92 (2 × d, *J* =16.0, 15.5 Hz, 1 H, NC*H*HiPh), 4.87 and 4.65 (2 × d, *J* = 16.0, 15.5 Hz, 1 H, NCH*H*Ph), 3.96–3.69 (m, 4 H, NCH_2CH_2), 1.37 and 0.90 (2 × s, 9 H, $C(CH_3)_3$); ^{13}C NMR (101 MHz, DMSO-d_6) δ 174.1 and 173.6 (C=O amide), 158.5 (d, J_{C-F} = 237.2 Hz, FC_{Ar} quat), 153.3 (C=O carbamate), 138.6 and 138.4 (C_{Ar} quat), 136.3 and 136.1 (C_{Ph} quat), 129.7 and 129.7 (C_{Ar} quat), 128.6 and 128.4 (2 × C_{Ph}), 127.8, 127.5, 127.3 and 127.2 (3 × C_{Ph}), 114.3 and 114.1 (C_{Ar}), 110.8 and 110.6 (C_{Ar}), 109.6 and 109.6 (C_{Ar}), 80.5 and 80.1 ($C(CH_3)_3$), 60.4 (NCH), 50.6 (NCH_2), 43.0 and 42.9 (NCH_2Ph), 42.3 (CH_2Cl), 27.8 and 27.2 ($C(CH_3)_3$); ^{19}F{^{1}H} NMR (377 MHz, DMSO-d_6) δ –121.2; HRMS (ESI$^+$) *m/z* Calculated for $C_{22}H_{25}N_2O_3F^{35}Cl$ $[M+H]^+$ 419.1538; Found 419.1533.

SMILES O=C1N(CC2=CC=CC=C2)C3=CC=C(F)C=C3C1N(CCCl)C(OC(C)(C)C)=O
InChI=1S/C22H24ClFN2O3/c1-22(2,3)29-21(28)25(12-11-23)19-17-13-16(24)9-10-18(17)26(20(19)27)14-15-7-5-4-6-8-15/h4-10,13,19H,11-12,14H2,1-3H3

tert-Butyl (1-benzyl-7-fluoro-2-oxoindolin-3-yl)(2-chloroethyl)carbamate (S46)

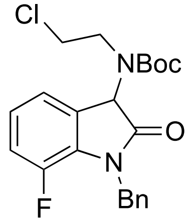

Following general procedure **A** on 1.0 mmol scale using diazo **S10** and protected amine **S33** in CH_2Cl_2. Purification by flash chromatography (30% Et_2O/pentane) afforded *tert*-butyl (1-benzyl-7-fluoro-2-oxoindolin-3-yl)(2-chloroethyl)carbamate **S46** (197 mg, 47%) as a pale brown gum. R_f 0.30 (30% Et_2O/pentane); ν_{max} (film)/cm^{-1} 2974, 2933, 2868, 1726 (C=O amide), 1702 (C=O carbamate), 1631, 1474, 1455, 1366, 1338, 1292, 1182, 1152, 1126, 1082, 1068, 766, 721, 697; ^{1}H NMR (400 MHz, $CDCl_3$) δ 7.38–7.24 (m, 5 H, 5 × HC_{Ar}), 7.22–7.12 (m, 2 H, 2 × HC_{Ar}), 7.10–6.98 (m, 1 H, HC_{Ar}), 5.39 (s, 1 H, NCH), 5.13 and 5.02 (2 × d, *J* = 16.0, 15.7 Hz, 1 H, NC*H*HPh), 4.93 and 4.76 (2 × d, *J* = 16.0, 15.7 Hz, NCH*H*Ph), 3.98–3.71 (m, 4 H, NCH_2CH_2), 1.37 and 0.92 (2 × s, 9 H, $C(CH_3)_3$); ^{13}C NMR (101 MHz, $CDCl_3$) δ 174.1 and 173.7 (C=O amide), 153.3 (C=O carbamate), 146.7 (d, J_{C-F} = 242 Hz, FC_{Ar} quat), 137.1 (C_{Ar} quat), 131.0 (C_{Ar} quat), 128.5, 128.3,

127.5 and 127.2 (4 × C_{Ar} and C_{Ar} quat), 126.6 (C_{Ar}), 123.34 and 123.28 (C_{Ar}), 119.1 (C_{Ar}), 116.2 (d, J_{C-F} = 19.6 Hz, C_{Ar}), 80.6 and 80.2 ($C(CH_3)_3$), 60.2 (NCH), 50.7 (NCH_2), 44.8 and 44.7 (NCH_2Ph), 42.4 and 42.0 (CH_2Cl), 27.8 and 27.2 ($C(CH_3)_3$); $^{19}F\{^1H\}$ NMR (377 MHz, DMSO-d_6) δ –135.5, –135.8 (br s); HRMS (ESI$^+$) *m/z* Calculated for $C_{22}H_{25}N_2O_3F^{35}Cl$ [M+H] 419.1538; Found 419.1532.

SMILES O=C1N(CC2=CC=CC=C2)C3=C(F)C=CC=C3C1N(CCCl)C(OC(C)(C)C)=O
InChI=1S/C22H24ClFN2O3/c1-22(2,3)29-21(28)25(13-12-23)19-16-10-7-11-17(24)18(16)26(20(19)27)14-15-8-5-4-6-9-15/h4-11,19H,12-14H2,1-3H3

tert-Butyl (1-benzyl-5-fluoro-2-oxoindolin-3-yl)(2-chloroethyl)carbamate (S47)

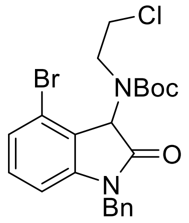

Following general procedure **A** on 0.5 mmol scale using diazo **S12** and protected amine **S33** in CH_2Cl_2 for 24 h. Purification by flash chromatography (40% Et_2O/hexane) afforded *tert*-Butyl (1-benzyl-5-fluoro-2-oxoindolin-3-yl)(2-chloroethyl)carbamate **S47** (77 mg, 35%) as a white solid. R_f 0.47 (50% Et_2O/hexane); mp = 118–127 ºC; ν_{max} (film)/cm^{-1} 2974, 2925, 1726 (C=O amide), 1697 (C=O carbamate), 1605, 1452, 1408, 1365, 1328, 1286, 1243, 1155, 768, 737, 699; ^1H NMR (400 MHz, DMSO-d_6) δ 7.49 – 6.96 (m, 8 H, 8 × HC_{Ar}), 5.40 (s, 1 H, NCH), 5.04 – 4.68 (m, 2 H, NCH_2Ph), 4.21 – 3.95 (m, 1 H, NCH_2CH_2), 3.80 (m, 2 H, NCH_2CH_2), 3.67 – 3.42 (m, 1 H, NCH_2CH_2), 1.35 and 0.85 (2 × br s, 9 H, $C(CH_3)_3$); ^{13}C NMR (101 MHz, DMSO-d_6) δ 173.4 (C=O) amide, 153.1 (C=O carbamate), 144.1 (C_{Ar} quat), 136.1 (C_{Ar} quat), 130.1 (C_{Ar}), 128.6, 128.4, 127.9, 127.6, 127.2, 126.2, 125.4 and 125.3 (8 × C_{Ar} quat), 117.0 (C_{Ar}), 108.3 (C_{Ar}), 80.4 and 80.1 ($C(CH_3)_3$), 61.9 and 61.3 (NCH), 51.9 and 51.2 (NCH_2), 43.2 and 42.9 (NCH_2Ph), 41.4 and 40.8 (CH_2Cl), 27.8 and 27.2 ($C(CH_3)_3$); HRMS (ESI$^+$) *m/z* Calculated for $C_{22}H_{25}BrClN_2O_3$ $[M+H]^+$ 479.0737; Found 479.0746.

SMILES O=C1N(CC2=CC=CC=C2)C3=CC=CC(Br)=C3C1N(CCCl)C(OC(C)(C)C)=O
InChI=1S/C22H24BrClN2O3/c1-22(2,3)29-21(28)25(13-12-24)19-18-16(23)10-7-11-17(18)26(20(19)27)14-15-8-5-4-6-9-15/h4-11,19H,12-14H2,1-3H3

tert-Butyl (1-benzyl-5-bromo-2-oxoindolin-3-yl)(2-chloroethyl)carbamate (S48)

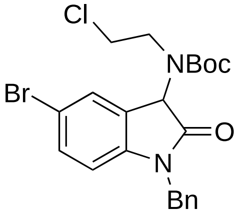

Following general procedure **A** on 1.0 mmol scale using diazo **S14** and protected amine **S33** in CH_2Cl_2. Purification by flash chromatography (20% to 30% Et_2O/pentane) afforded *tert*-butyl (1-benzyl-5-bromo-2-oxoindolin-3-yl)(2-chloroethyl)carbamate **S48** (377 mg, 79%) as a white solid. R_f 0.29 (30% Et_2O/pentane); mp = 57–59 ºC; ν_{max} (film)/cm^{-1} 2974, 2930, 1698 (br s, 2 × C=O), 1608, 1481, 1455, 1406, 1366, 1331, 1291, 1248, 1154, 1113, 1081, 807, 698; ^1H NMR (400 MHz, $CDCl_3$) δ 7.52–7.23 (m, 7 H, 7 × HC_{Ar}), 6.95 and 6.77 (2 × d, *J* = 8.2 Hz, 1 H, HC_{Ar}), 5.38–5.21 (m, 1 H, NCH), 5.05 and 4.94 (2 × d, *J* = 15.9 Hz, 1 H, NC*H*HPh), 4.86 and 4.66 (2 × d, *J* = 15.9 Hz, 1 H, NCH*H*Ph), 3.98–3.66 (m, 4 H, NCH_2CH_2), 1.37 and 0.90 (2 × br s, 9 H, $C(CH_3)_3$); ^{13}C NMR (101 MHz, $CDCl_3$) δ 173.8 and 173.3 (C=O amide), 153.3 (C=O carbamate), 141.7 and 141.5 (NC_{Ar} quat), 136.1 and 135.9 (C_{Ph} quat), 130.7 (C_{Ar}), 130.2 (C_{Ar} quat), 128.6 and 128.4 (2 × C_{Ph}), 127.7 and 127.5 (2 × C_{Ph}), 127.3 and 127.2 (C_{Ph}), 125.8 and 125.6 (C_{Ar}), 114.0 (BrC_{Ar} quat), 110.9 and 110.8 (C_{Ar}), 80.6 and 80.2 ($C(CH_3)_3$), 60.0 (NCH), 50.7 (NCH_2), 43.0 and 42.8 (NCH_2Ph), 42.4 and 42.0 (CH_2Cl), 27.8 and 27.2 ($C(CH_3)_3$); HRMS (FTMS – pAPCI) *m/z* Calculated for $C_{22}H_{23}N_2O_3{}^{35}Cl^{79}Br^-$ $[M–H]^-$ 477.0586; Found 477.0567.

SMILES O=C1N(CC2=CC=CC=C2)C3=CC=C(Br)C=C3C1N(CCCl)C(OC(C)(C)C)=O
InChI=1S/C22H24BrClN2O3/c1-22(2,3)29-21(28)25(12-11-24)19-17-13-16(23)9-10-18(17)26(20(19)27)14-15-7-5-4-6-8-15/h4-10,13,19H,11-12,14H2,1-3H3

tert-Butyl (1-benzyl-6-bromo-2-oxoindolin-3-yl)(2-chloroethyl)carbamate (S49)

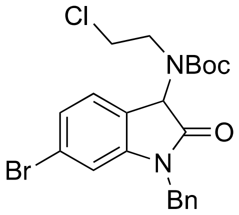

Following general procedure **A** on 1.0 mmol scale using diazo **S16** and protected amine **S33** in CH_2Cl_2. Purification by flash chromatography (25% Et_2O/pentane) afforded *tert*-butyl (1-benzyl-6-bromo-2-oxoindolin-3-yl)(2-chloroethyl)carbamate **S49** (377 mg, 79%) as a white solid. R_f 0.33 (30% Et_2O/pentane); mp = 57–60 ºC; ν_{max} (film)/cm^{-1} 2973, 2929, 1720 (C=O amide), 1701 (C=O carbamate), 1606, 1589, 1484, 1454, 1439, 1426, 1405, 1366, 1333, 1290, 1249, 1153, 1105, 927, 875, 800, 741, 724, 697, 670; ^{1}H NMR (400 MHz, DMSO-*d$_6$*) δ 7.48–7.03 (m, 8 H, 8 × HC_{Ar}), 5.26 (s, 1 H, NCH), 5.04 and 4.95 (2 × d, *J* = 15.6 and 16.0 Hz, 1 H, NC*H*HPh), 4.89 and 4.73 (2 × d, *J* = 16.0 and 15.6 Hz, 1 H, NCH*H*Ph), 3.99–3.65 (m, 4 H, NCH_2CH_2), 1.36 and 0.91 (2 × s, 9 H, $C(CH_3)_3$); ^{13}C NMR (101 MHz, DMSO-*d$_6$*) δ 174.2 and 173.8 (C=O amide), 153.3 (C=O carbamate), 144.1 and 143.9 (NC_{Ar} quat), 136.2 and 135.9 (C_{Ph} quat), 128.6 and 128.4 (2 × C_{Ph}), 127.7, 127.5, 127.3, 127.2 and 127.1 (3 × C_{Ph} and C_{Ar} quat), 124.7 and 124.4 (2 × C_{Ar}), 120.7 (BrC_{Ar} quat), 111.9 and 111.7 (C_{Ar}), 80.5 and 80.1 (*C*$(CH_3)_3$), 59.8 (NCH), 50.7 (NCH_2), 42.9 and 42.7 (NCH_2Ph), 42.3 and 42.0 (CH_2Cl), 27.8 and 27.2 ($C(CH_3)_3$); HRMS (FTMS – pAPCI) *m/z* Calculated for $C_{22}H_{23}N_2O_3{}^{35}Cl^{79}Br^-$ [M–H]$^-$ 477.0586; Found 477.0604.

SMILES O=C1N(CC2=CC=CC=C2)C3=CC(Br)=CC=C3C1N(CCCl)C(OC(C)(C)C)=O
InChI=1S/C22H24BrClN2O3/c1-22(2,3)29-21(28)25(12-11-24)19-17-10-9-16(23)13-18(17)26(20(19)27)14-15-7-5-4-6-8-15/h4-10,13,19H,11-12,14H2,1-3H3

tert-Butyl (1-benzyl-7-bromo-2-oxoindolin-3-yl)(2-chloroethyl)carbamate (S50)

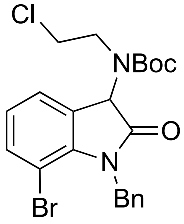

Following general procedure **A** on 0.85 mmol scale using diazo **S18** and protected amine **S33** in CH_2Cl_2. Purification by flash chromatography (20% to 30% Et_2O/pentane) afforded *tert*-butyl (1-benzyl-7-bromo-2-oxoindolin-3-yl)(2-chloroethyl)carbamate **S50** (269 mg, 66%) as a white solid. R_f 0.45 (30% Et_2O/pentane); mp = 128–130 ºC; ν_{max} (film)/cm^{-1} 2974, 2928, 1728 (C=O amide), 1694 (C=O carbamate), 1608, 1450, 1418, 1357, 1327, 1251, 1200, 1175, 1149, 1115, 1019, 1075, 963, 917, 850, 882, 768, 749, 729, 659; ^{1}H NMR (400 MHz, DMSO-*d$_6$*) δ 7.45–7.20 (m, 7 H, 7 × HC_{Ar}), 7.04–6.94 (m, 1 H, HC_{Ar}), 5.44 (s, 1 H, NCH), 5.31 (d, *J* = 16.7 Hz, NC*H*HPh), 5.26 (d, *J* = 16.7 Hz, NCH*H*Ph), 3.97–3.73 (m, 4 H, NCH_2CH_2), 1.37 and 1.00 (2 × s, 9 H, $C(CH_3)_3$); ^{13}C NMR (101 MHz, DMSO-*d$_6$*) δ 175.2 (C=O amide), 153.2 (C=O carbamate), 139.54 and 139.45 (NC_{Ar} quat), 137.7 and 137.6 (C_{Ph} quat), 133.7 (C_{Ar}), 131.4 (C_{Ar} quat), 128.4 and 128.2 (2 × C_{Ph}), 127.0 and 126.7 (C_{Ph}), 126.3 and 126.1 (2 × C_{Ph}), 124.1 (C_{Ar}), 122.2 (C_{Ar}), 101.4 (BrC_{Ar} quat), 80.7 and 80.4 (*C*$(CH_3)_3$), 59.6 (NCH), 50.7 (NCH_2), 44.0 (NCH_2Ph), 41.9 (CH_2Cl), 27.8 and 27.3 ($C(CH_3)_3$); HRMS (FTMS – pAPCI) *m/z* Calculated for $C_{22}H_{23}N_2O_3{}^{35}Cl^{79}Br^-$ [M–H]$^-$ 477.0586; Found 477.0589.

SMILES O=C1N(CC2=CC=CC=C2)C3=C(Br)C=CC=C3C1N(CCCl)C(OC(C)(C)C)=O
InChI=1S/C22H24BrClN2O3/c1-22(2,3)29-21(28)25(13-12-24)19-16-10-7-11-17(23)18(16)26(20(19)27)14-15-8-5-4-6-9-15/h4-11,19H,12-14H2,1-3H3

tert-Butyl (1-benzyl-5-chloro-2-oxoindolin-3-yl)(2-chloroethyl)carbamate (S51)

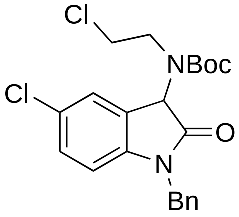

Following general procedure **A** on 1.0 mmol scale using diazo **S20** and protected amine **S33** in CH_2Cl_2. Purification by flash chromatography (CH_2Cl_2 to 2% Et_2O/CH_2Cl_2 to 4% Et_2O/CH_2Cl_2) afforded *tert*-butyl (1-benzyl-5-chloro-2-oxoindolin-3-yl)(2-chloroethyl)carbamate **S51** (224 mg, 51%) as a pale yellow solid. R_f 0.52 (2% Et_2O/CH_2Cl_2); mp = 60–63 ºC; ν_{max} (film)/cm^{-1} 2971, 2926, 1689 (br s, 2 × C=O), 1610, 1480, 1428, 1364, 1327, 1245, 1152, 1078, 809, 738, 697; ^{1}H NMR (400 MHz, DMSO-*d$_6$*) δ 7.45–7.10 (m, 7 H, 7 × HC_{Ar}), 7.02–6.83 (m, 1 H, HC_{Ar}), 5.33 (br s, 1 H, NCH), 5.10–4.84 and 4.77–4.61 (m, 2 H, NCH_2Ph), 3.98–3.67 (m, 4 H, NCH_2CH_2), 1.37 and 0.90 (2 × s, 9 H, $C(CH_3)_3$); ^{13}C NMR (101 MHz, DMSO-*d$_6$*) δ 173.9 and 173.5 (C=O amide), 153.3 (C=O carbamate), 141.3 and 141.1 (NC_{Ar} quat), 136.2 and 135.9 (C_{Ph}

quat), 129.9 (C_{Ar} quat), 128.6, 128.4, 127.9, 127.7, 127.6, 127.3, 127.2, 126.8, 126.4 (6 × C_{Ar} and C_{Ar} quat), 122.9 (C_{Ar}), 110.2 (C_{Ar}), 80.6 and 80.2 (*C*(CH_3)$_3$), 60.1 (NCH), 50.7 (NCH_2), 43.0 and 42.8 (NCH_2Ph), 42.4 and 42.0 (CH_2Cl), 27.8 and 27.2 (C(CH_3)$_3$); HRMS (ESI$^+$) *m/z* Calculated for $C_{22}H_{25}N_2O_3{}^{35}Cl_2$ [M+H] 435.1242; Found 435.1247.

SMILES O=C1N(CC2=CC=CC=C2)C3=CC=C(Cl)C=C3C1N(CCCl)C(OC(C)(C)C)=O
InChI=1S/C22H24Cl2N2O3/c1-22(2,3)29-21(28)25(12-11-23)19-17-13-16(24)9-10-18(17)26(20(19)27)14-15-7-5-4-6-8-15/h4-10,13,19H,11-12,14H2,1-3H3

tert-Butyl (1-benzyl-5-nitro-2-oxoindolin-3-yl)(2-chloroethyl)carbamate (S52)

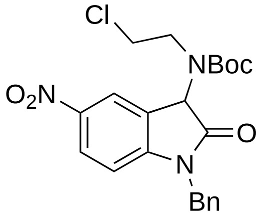

Following general procedure **A** on 0.95 mmol scale using diazo **S22** and protected amine **S33** in CH_2Cl_2. Purification by flash chromatography (40% Et_2O/pentane) afforded *tert*-butyl (1-benzyl-5-nitro-2-oxoindolin-3-yl)(2-chloroethyl)carbamate **S52** (254 mg, 60%) as a white solid. R_f 0.34 (50% Et_2O/pentane); mp = 67–69 ºC; ν_{max} (film)/cm^{-1} 2973, 2926, 1735 (C=O amide), 1698 (C=O carbamate), 1611, 1517, 1444, 1406, 1388, 1366, 1331, 1248, 1152, 1124, 1071, 884, 823, 750; ^{1}H NMR (400 MHz, DMSO-*d$_6$*) δ 8.28–8.15 (m, 2 H, 2 × HC_{Ar}), 7.47–7.23 and 7.07–7.03 (m, 6 H, HC_{Ar} and 5 × HC_{Ph}), 5.48 and 5.37 (2 × s, 1 H, NCH), 5.17–4.94 and 4.87–4.79 (m, 2 H, NCH_2Ph), 4.04–3.73 (m, 4 H, NCH_2CH_2), 1.36 and 0.87 (2 × s, 9 H, C(CH_3)$_3$); ^{13}C NMR (101 MHz, DMSO-*d$_6$*) δ 174.8 and 174.5 (C=O amide), 153.4 and 153.1 (C=O carbamate), 148.4 and 148.2 (NC_{Ar} quat), 142.6 (NO_2C_{Ar} quat), 135.6 and 135.5 (C_{Ph} quat), 128.9, 128.7, 128.5, 127.80, 127.75, 127.5, 127.2 (5 × C_{Ph} and C_{Ar} quat), 125.5 (C_{Ar}), 118.3 and 118.2 (C_{Ar}), 109.0 (C_{Ar}), 80.9 and 80.5 (*C*(CH_3)$_3$), 60.0 and 59.8 (NCH), 50.7 (NCH_2), 43.3 and 43.1 (NCH_2Ph), 42.6 and 42.2 (CH_2Cl), 27.8 and 27.2 (C(CH_3)$_3$); HRMS (ESI$^+$) *m/z* Calculated for $C_{22}H_{25}N_3O_5{}^{35}Cl$ [M+H] 446.1483; Found 446.1477.

SMILES O=C1N(CC2=CC=CC=C2)C3=CC=C([N+]([O-])=O)C=C3C1N(CCCl)C(OC(C)(C)C)=O
InChI=1S/C22H24ClN3O5/c1-22(2,3)31-21(28)24(12-11-23)19-17-13-16(26(29)30)9-10-18(17)25(20(19)27)14-15-7-5-4-6-8-15/h4-10,13,19H,11-12,14H2,1-3H3

tert-butyl (1-benzyl-2-oxo-2,3-dihydro-1*H*-pyrrolo[2,3-b]pyridin-3-yl)(2-chloroethyl)carbamate (S53)

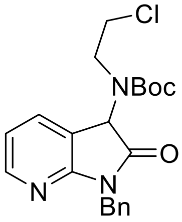

Following general procedure **A** on 0.7 mmol scale using diazo **S23** and protected amine **S33** in CH_2Cl_2. Purification by flash chromatography (35% Et_2O/hexane) afforded *tert*-butyl (1-benzyl-2-oxo-2,3-dihydro-1*H*-pyrrolo[2,3-b]pyridin-3-yl)(2-chloroethyl)carbamate **S53** (122 mg, 44%) as an off-white gum. R_f 0.44 (55% Et2O/hexane); ν_{max} (film)/cm^{-1} 2974, 2930, 1724 (C=O amide), 1700 (C=O carbamate), 1443, 1404, 1338, 1364, 1350, 1169, 756, 697; ^{1}H NMR (400 MHz, DMSO-*d$_6$*) δ 8.14 (dd, *J* = 27.6, 5.2 Hz, 1 H, HC_{Ar}), 7.67 (dd, *J* = 14.1, 7.1 Hz, 1H, HC_{Ar}), 7.50 – 7.20 (m, 5 H, 5 × HC_{Ar}), 7.07 (m, 1 H, HC_{Ar}), 5.36 (s, 1 H, NCH), 4.90 (m, 2 H, NCH_2Ph), 4.08 – 3.57 (m, 4 H, NCH_2CH_2), 1.35 and 0.87 (2 x br s, 9 H, C(CH_3)$_3$); ^{13}C NMR (101 MHz, DMSO-*d$_6$*) δ 174.2 and 173.8 (C=O amide), 155.6 and 153.2 (C=O carbamate), 146.7 (NCN quat), 136.7 (C_{Ar}), 130.5 (C_{Ar} quat), 128.4, 128.3, 128.0, 127.4, 127.2, 127.1 and 122.6 (7 × C_{Ar}), 118.4 (C_{Ar}), 80.7 and 80.2 (*C*(CH3)3), 59.5 (NCH), 50.7 (NCH_2), 42.5 (NCH_2Ph), 42.0 (CH_2Cl), 27.8 and 27.2 (C(CH_3)$_3$); HRMS (ESI$^+$) *m/z* Calculated for $C_{21}H_{25}ClN_3O_3$ [M+H]$^+$ 402.1584; Found 402.1579.

SMILES O=C1N(CC2=CC=CC=C2)C3=NC=CC=C3C1N(CCCl)C(OC(C)(C)C)=O
InChI=1S/C21H24ClN3O3/c1-21(2,3)28-20(27)24(13-11-22)17-16-10-7-12-23-18(16)25(19(17)26)14-15-8-5-4-6-9-15/h4-10,12,17H,11,13-14H2,1-3H3

tert-Butyl (2-chloroethyl)(1-methyl-2-oxoindolin-3-yl)carbamate (S54)

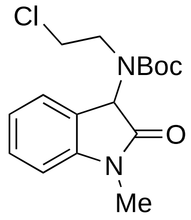

Following general procedure **A** on 1.0 mmol scale using diazo **S24** and protected amine **S33** in toluene. Purification by flash chromatography (50% Et_2O/pentane) afforded *tert*-butyl (2-chloroethyl)(1-methyl-2-oxoindolin-3-yl)carbamate **S54** (210 mg, 65%) as a white solid. R_f 0.19 (50% Et_2O/pentane); mp = 123–124 ºC; ν_{max} (film)/cm^{-1} 2982, 1716 (C=O amide), 1697 (C=O carbamate), 1609, 1456, 1441, 1370, 1245, 1156, 1120, 1088, 759, 731; ^{1}H NMR (400 MHz, DMSO-*d*$_6$) δ 7.35–7.23 (m, 2 H, 2 × HC_{Ar}), 7.09–6.95 (m, 2 H, 2 × HC_{Ar}), 5.06 (br s, 1 H, NCH), 3.67–3.94 (m, 4 H, $N(CH_2)_2$), 3.11 (s, 3 H, NCH_3), 1.35 and 0.92 (2 × s, 9 H, $C(CH_3)_3$); ^{13}C NMR (101 MHz, DMSO-*d*$_6$) δ 174.0 (C=O amide), 153.5 (C=O carbamate), 143.3 (NC_{Ar} quat), 128.3 (C_{Ar}), 127.6 (C_{Ar} quat), 122.5 (C_{Ar}), 122.0 (C_{Ar}), 108.2 (C_{Ar}), 79.7 ($\mathit{C}(CH_3)_3$), 60.2 (NCH), 50.7 (NCH_2), 41.9 (CH_2Cl), 27.8 and 27.3 ($C(\mathit{C}H_3)_3$), 26.0 (NCH_3); HRMS (ESI$^+$) *m/z* Calculated for $C_{16}H_{22}N_2O_3Cl$ [M+H] 325.1319; Found 325.1324.

SMILES: O=C1N(C)C2=CC=CC=C2C1N(CCCl)C(OC(C)(C)C)=O
InChI=1S/C16H21ClN2O3/c1-16(2,3)22-15(21)19(10-9-17)13-11-7-5-6-8-12(11)18(4)14(13)20/h5-8,13H,9-10H2,1-4H3

tert-Butyl (2-chloroethyl)(1-(4-methoxybenzyl)-2-oxoindolin-3-yl)carbamate (S55)

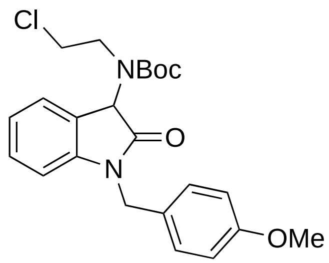

Following general procedure **A** on 1.0 mmol scale using diazo **S25** and protected amine **S33** in toluene. Purification by flash chromatography (30% to 40% Et_2O/pentane) afforded *tert*-butyl (2-chloroethyl)(1-(4-methoxybenzyl)-2-oxoindolin-3-yl)carbamate **S55** (138 mg, 64%) as a pale yellow solid. R_f 0.11 (25% Et_2O/pentane); mp = 107–110 ºC; ν_{max} (film)/cm^{-1} 2971, 2933, 1718 (C=O amide), 1692 (C=O carbamate), 1610, 1439, 1364, 1338, 1245, 1156, 1126, 753, 727; ^{1}H NMR (400 MHz, DMSO-*d*$_6$) δ 7.40–7.15 (m, 4 H, 4 × HC_{Ar}), 7.06–6.95 (m, 2 H, 2 × HC_{Ar}), 6.92–6.82 (m, 2 H, 2 × HC_{Ar}), 5.21 (s, 1 H, NCH), 4.99 and 4.85 (2 × d, *J* = 15.1 Hz, 1 H, NC*H*HAr), 4.80 and 4.57 (2 × d, *J* = 15.1 Hz, 1 H, NCH*H*Ar), 3.99–3.66 (m, 7 H, N$\mathit{CH_2CH_2}$ and OCH_3), 1.39 and 0.86 (2 × s, 9 H, $C(CH_3)_3$); ^{13}C NMR (101 MHz, DMSO-*d*$_6$) δ 174.1 (C=O amide), 159.6 and 158.5 (OC_{Ar} quat), 153.5 (C=O carbamate), 142.4 and 142.3 (NC_{Ar} quat), 129.2, 128.6, 128.4, 128.1 and 127.7 (4 × C_{Ar} and C_{Ar} quat), 122.5 (C_{Ar} quat), 122.0 (C_{Ar}), 113.9 and 113.8 (2 × C_{Ar}), 109.1 and 108.8 (C_{Ar}), 80.4 and 79.8 ($\mathit{C}(CH_3)_3$), 60.2 (NCH), 55.0 (OCH_3), 50.7 (NCH_2), 42.4 and 42.3 (NCH_2Ar), 41.9 (CH_2Cl), 27.8 and 27.2 ($C(\mathit{C}H_3)_3$); HRMS (ESI$^+$) *m/z* Calculated for $C_{23}H_{27}N_2O_4Na^{35}Cl$ [M+Na] 453.1557; Found 453.1551.

SMILES O=C1N(CC2=CC=C(OC)C=C2)C3=CC=CC=C3C1N(CCCl)C(OC(C)(C)C)=O
InChI=1S/C23H27ClN2O4/c1-23(2,3)30-22(28)25(14-13-24)20-18-7-5-6-8-19(18)26(21(20)27)15-16-9-11-17(29-4)12-10-16/h5-12,20H,13-15H2,1-4H3

tert-Butyl (1-(4-bromobenzyl)-2-oxoindolin-3-yl)(2-chloroethyl)carbamate (S56)

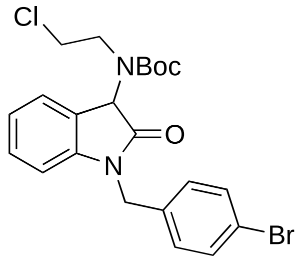

Following general procedure **A** on 1.0 mmol scale using diazo **S26** and protected amine **S33** in toluene. Purification by flash chromatography (30% Et_2O/pentane) afforded *tert*-butyl (1-(4-bromobenzyl)-2-oxoindolin-3-yl)(2-chloroethyl)carbamate **S56** (325 mg, 68%) as a white solid. R_f 0.18 (30% Et_2O/pentane); mp = 58–60 ºC; ν_{max} (film)/cm^{-1} 2975, 2932, 1720 (C=O amide), 1703 (C=O carbamate), 1613, 1487, 1467, 1407, 1365, 1293, 1249, 1156, 1012, 750, 731; ^{1}H NMR (400 MHz, DMSO-*d*$_6$) δ 7.55 and 7.49 (2 × d, *J* = 8.2 Hz, 2 H, 2 × HC_{Ar}), 7.42–7.16 (m, 4 H, 4 × HC_{Ar}), 7.08–6.94 and 6.85–6.80 (2 × m, 2 H, 2 × HC_{Ar}), 5.25 (s, 1 H, NCH), 5.04 and 4.92 (d, *J* = 15.9 Hz, 1 H, NC*H*H), 4.85 and 4.63 (d, *J* = 15.7 Hz, 1 H, NCH*H*), 4.00–3.69 (m, 4 H, NCH_2CH_2), 1.37 and 0.87 (2 × br s, 9 H, $C(CH_3)_3$); ^{13}C NMR (101 MHz, DMSO-*d*$_6$) δ 174.3 and 173.7 (C=O amide), 153.5 and 153.4 (C=O carbamate), 142.2 and 142.1 (NC_{Ar} quat), 136.0 and 135.8 (C_{Ar} quat), 131.4 and 131.3 (2 × C_{Ar}), 130.0 and 129.6 (2 × C_{Ar} and C_{Ar} quat), 128.3 and 128.2 (C_{Ar}), 127.7 and 127.6 (C_{Ar}), 122.7 and 122.2 (C_{Ar}), 120.6

(BrC_{Ar} quat), 108.9 and 108.7 (C_{Ar}), 80.4 and 79.9 (*C*(CH_3)$_3$), 60.2 (NCH), 50.7 (NCH_2), 42.3, 42.2 and 42.0 (NCH_2Ar and CH_2Cl), 27.8 and 27.2 (C(*C*H_3)$_3$); HRMS (FTMS – pAPCI) *m/z* Calculated for $C_{22}H_{23}N_2O_3{}^{35}Cl^{79}Br^-$ $[M–H]^-$ 477.0586; Found 477.0587.

SMILES: O=C1N(CC2=CC=C(Br)C=C2)C3=CC=CC=C3C1N(CCCl)C(OC(C)(C)C)=O
InChI=1S/C22H24BrClN2O3/c1-22(2,3)29-21(28)25(13-12-24)19-17-6-4-5-7-18(17)26(20(19)27)14-15-8-10-16(23)11-9-15/h4-11,19H,12-14H2,1-3H3

tert-Butyl (1-(3,5-bis(trifluoromethyl)benzyl)-2-oxoindolin-3-yl)(2-chloroethyl)carbamate (S57)

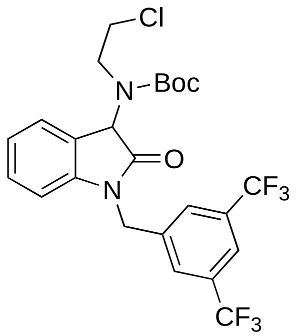

Following general procedure **A** on 0.5 mmol scale using diazo **S27** and protected amine **S33** in toluene. Purification by flash chromatography (30% Et_2O/pentane) afforded *tert*-butyl (1-(3,5-bis(trifluoromethyl)benzyl)-2-oxoindolin-3-yl)(2-chloroethyl)carbamate **S57** (160 mg, 60%) as a pale orange solid. R_f 0.17 (30% Et_2O/pentane); mp = 67–70 ºC; ν_{max} (film)/cm^{-1} 2986, 2933, 1729 (C=O amide), 1702 (C=O carbamate), 1465, 1414, 1341, 1277, 1157, 1126, 1010, 907, 882, 747, 737, 713; ^{1}H NMR (400 MHz, DMSO-*d*$_6$) δ 8.16 (s, 2 H, 2 × HC_{Ar}), 8.05 and 7.97 (2 × s, 1 H, HC_{Ar}), 7.37–7.32 (m, 1 H, HC_{Ar}), 7.28 and 7.18 (2 × t, *J* = 7.6 Hz, 1 H, HC_{Ar}), 7.15–6.98 and 6.86–6.77 (m, 2 H, 2 × HC_{Ar}), 5.36–5.10 (m, 2 H, NCH and NC*H*HAr), 5.01–4.90 (m, 1 H, NCH*H*Ar), 4.01–3.69 (m, 4 H, NCH_2CH_2), 1.34 and 0.82 (2 × s, 9 H, C(CH_3)$_3$); ^{13}C NMR (101 MHz, DMSO-*d*$_6$) δ 174.9 and 174.0 (C=O amide), 153.6 and 153.4 (C=O carbamate), 142.1 and 142.0 (C_{Ar} quat), 140.3 and 140.2 (C_{Ar}), 130.7 and 130.5 (2 × q, $J_{C–F}$ = 33 Hz, 2 × *C*$_{Ar}CF_3$ quat), 129.0, 128.5, 128.4, 128.0 and 126.8 (3 × C_{Ar} and C_{Ar} quat), 123.4 (q, $J_{C–F}$ = 273 Hz, 2 × CF_3), 123.3 and 123.0 (C_{Ar}), 122.6 (C_{Ar}), 121.6 and 121.2 (C_{Ar}), 108.9 (C_{Ar}), 80.6 and 80.0 (*C*(CH_3)$_3$), 60.7 and 60.4 (NCH), 51.5 and 51.0 (NCH_2), 42.5, 42.2 and 42.0 (NCH_2Ar and CH_2Cl), 27.8 and 27.3 (C(*C*H_3)$_3$); ^{19}F{^{1}H} NMR (377 MHz, DMSO-*d*^{6}) δ –61.26 and –61.34; HRMS (FTMS – pAPCI) *m/z* Calculated for $C_{24}H_{22}N_2O_3F_6Cl^-$ $[M+H]^-$ 535.1218; Found 535.1228.

SMILES: O=C1N(CC2=CC(C(F)(F)F)=CC(C(F)(F)F)=C2)C3=CC=CC=C3C1N(C(OC(C)(C)C)=O)CCCl
InChI=1S/C24H23ClF6N2O3/c1-22(2,3)36-21(35)32(9-8-25)19-17-6-4-5-7-18(17)33(20(19)34)13-14-10-15(23(26,27)28)12-16(11-14)24(29,30)31/h4-7,10-12,19H,8-9,13H2,1-3H3

tert-Butyl (2-chloroethyl)(1-(naphthalen-2-ylmethyl)-2-oxoindolin-3-yl)carbamate (S58)

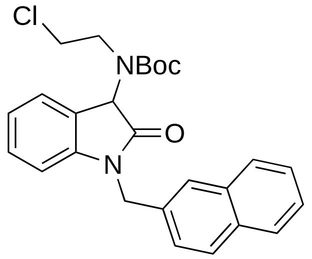

Following general procedure **A** on 0.5 mmol scale using diazo **S28** and protected amine **S33** in toluene. Purification by flash chromatography (30% Et_2O/pentane) afforded *tert*-butyl (2-chloroethyl)(1-(naphthalen-2-ylmethyl)-2-oxoindolin-3-yl)carbamate **S58** (95 mg, 42%) as a white solid. R_f 0.20 (50% Et_2O/hexane); mp = 74–76 ºC; ν_{max} (film)/cm^{-1} 3055, 2974, 2929, 1718 (C=O amide), 1701 (C=O carbamate), 1614, 1467, 1364, 1339, 1247, 1154, 747; ^{1}H NMR (400 MHz, $CDCl_3$) δ 7.97–7.83 (m, 4 H, 4 × HC_{Ar}), 7.58–7.44 (m, 3 H, 3 × HC_{Ar}), 7.35–7.28 (m, 1 H, HC_{Ar}), 7.25–7.12 (m, 1 H, HC_{Ar}), 7.06–6.97 (m, 2 H, 2 × HC_{Ar}), 5.30 (s, 1 H, NCH), 5.25 (d, *J* = 15.5 Hz, 0.5 H, NC*H*HPh), 5.14 (d, *J* = 16.2 Hz, 0.5 H, NCH*H*Ph), 5.01 (d, *J* = 16.2 Hz, 0.5 H, NCH*H*Ph), 4.79 (d, *J* = 15.5 Hz, 0.5 H, NC*H*HPh), 4.01–3.73 (m, 4 H, N(CH_2)$_2$), 1.41 and 0.88 (2 × s, 9 H, C(CH_3)$_3$); ^{13}C NMR (101 MHz, $CDCl_3$) δ 174.4 (C=O amide), 153.5 (C=O carbamate), 142.3 (NC_{Ar} quat), 134.0 (C_{Ar} quat), 132.8 (C_{Ar} quat), 132.3 (C_{Ar} quat), 128.3 (C_{Ar}), 128.2 (C_{Ar}), 127.6 (2 × C_{Ar}), 126.4 (C_{Ar}), 126.1 (C_{Ar}), 126.0 (C_{Ar}), 125.9 (C_{Ar}), 125.6 (C_{Ar}), 122.7 (C_{Ar}), 122.2 (C_{Ar}), 108.8 (C_{Ar}), 79.9 (*C*(CH_3)$_3$), 60.3 (NCH), 50.8 (NCH_2), 43.2 (NCH_2Ar), 42.0 (CH_2Cl), 27.9 and 27.2 (C(*C*H_3)$_3$); HRMS (ESI$^+$) *m/z* Calculated for $C_{26}H_{28}N_2O_3Cl$ [M+H] 451.1788; Found 451.1795.

SMILES: O=C1N(CC2=CC=C(C=CC=C3)C3=C2)C4=CC=CC=C4C1N(CCCl)C(OC(C)(C)C)=O
InChI=1S/C26H27ClN2O3/c1-26(2,3)32-25(31)28(15-14-27)23-21-10-6-7-11-22(21)29(24(23)30)17-18-12-13-19-8-4-5-9-20(19)16-18/h4-13,16,23H,14-15,17H2,1-3H3

Benzyl (1-benzyl-2-oxoindolin-3-yl)(2-chloroethyl)carbamate (2d)

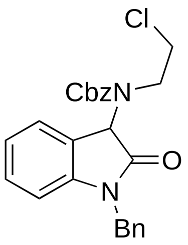

Following general procedure **A** on 1.0 mmol scale using diazo **1** and protected amine **S37** in toluene. Purification by flash chromatography (CH_2Cl_2 to 2% EtOAc/CH_2Cl_2) afforded benzyl (1-benzyl-2-oxoindolin-3-yl)(2-chloroethyl)carbamate **2d** (253 mg, 58%) as a brown gummy solid. R_f 0.33 (2% EtOAc/CH_2Cl_2); mp = 110–112 ºC; ν_{max} (film)/cm^{-1} 3026, 2922, 1701 (2 × C=O), 1655, 1439, 1420, 1284, 1156, 1178, 1005, 907, 746, 662; ^{1}H NMR (400 MHz, DMSO-*d*$_6$) δ 7.42–7.10 (m, 12 H, 12 × HC_{Ar}), 7.06–6.97 (m, 1 H, HC_{Ar}), 6.87–6.79 and 6.63–6.55 (m, 1 H, HC_{Ar}), 5.36 (br s, 1 H, NCH), 5.13 (s, 1 H, OC*H*HPh), 4.97–4.75 and 4.62–4.40 (m, 3 H, OCH*H*Ph and NCH_2Ph), 4.08–3.70 (m, 4 H, NCH_2CH_2); ^{13}C NMR (101 MHz, DMSO-*d*$_6$) δ 173.7 and 173.4 (C=O amide), 154.5 and 154.2 (C=O carbamate), 142.5 and 142.2 (NC_{Ar} quat), 136.3, 136.2 and 135.6 (2 × C_{Ph} quat), 128.5, 128.4, 128.1, 128.0 (4 × C_{Ph} and C_{Ar} quat), 127.6, 127.4, 127.3, 127.2, 126.9 (6 × C_{Ph} and C_{Ar}), 123.2 and 123.0 (C_{Ar}), 122.4 and 122.2 (C_{Ar}), 109.1 (C_{Ar}), 67.0 and 66.7 (OCH_2Ph), 60.4 and 60.1 (NCH), 51.5 and 50.5 (NCH_2), 42.9 (NCH_2Ph), 42.0 and 41.8 (CH_2Cl); HRMS (ESI$^+$) *m/z* Calculated for $C_{25}H_{23}N_2O_3Na^{35}Cl$ [M+Na] 457.1295; Found 457.1306.

SMILES O=C1N(CC2=CC=CC=C2)C3=CC=CC=C3C1N(CCCl)C(OCC4=CC=CC=C4)=O
InChI=1S/C25H23ClN2O3/c26-15-16-27(25(30)31-18-20-11-5-2-6-12-20)23-21-13-7-8-14-22(21)28(24(23)29)17-19-9-3-1-4-10-19/h1-14,23H,15-18H2

Methyl (1-benzyl-2-oxoindolin-3-yl)(2-chloroethyl)carbamate (2e)

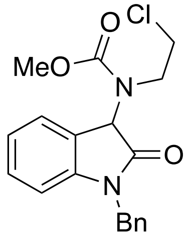

Following general procedure **A** on 1.0 mmol scale using diazo **1** and protected amine **S38** in toluene. Purification by flash chromatography (CH_2Cl_2 to 2% Et_2O/CH_2Cl_2) afforded methyl (1-benzyl-2-oxoindolin-3-yl)(2-chloroethyl)carbamate **2e** (106 mg, 30%) as a brown gummy solid. R_f 0.27 (90% CH_2Cl_2/pentane); ν_{max} (film)/cm^{-1} 3060, 2956, 1707 (br s, 2 × C=O), 1614, 1469, 1361, 1174, 753; ^{1}H NMR (400 MHz, $CDCl_3$) δ 7.37–7.19 (m, 7 H, 7 × HC_{Ar}), 7.09–7.02 (m, 1 H, HC_{Ar}), 6.81–6.62 (m, 1 H, HC_{Ar}), 5.09–4.74 (m, 3 H, NCH_2Ph and NCH), 3.86–3.44 (m, 7 H, NCH_2CH_2Cl and OCH_3); ^{13}C NMR (101 MHz, $CDCl_3$) δ 173.5 (C=O amide), 156.3 (C=O carbamate), 142.9 and 142.5 (NC_{Ar} quat), 135.4 (C_{Ph} quat), 129.4, 128.8 127.8, 127.7, 127.5, 127.2 (5 × C_{Ph}, C_{Ar} and $C_{Ar.}$ quat), 124.9 and 124.0 (C_{Ar}), 123.0 (C_{Ar}), 109.5 (C_{Ar}), 60.5 (NCH), 53.5 and 53.2 (OCH_3 and NCH_2), 44.0 (NCH_2Ph), 41.1 (CH_2Cl); HRMS (ESI$^+$) *m/z* Calculated for $C_{19}H_{20}N_2O_3{}^{35}Cl$ [M+H] 359.1162; Found 359.1154.

SMILES O=C1N(CC2=CC=CC=C2)C3=CC=CC=C3C1N(C(OC)=O)CCCl
InChI=1S/C19H19ClN2O3/c1-25-19(24)21(12-11-20)17-15-9-5-6-10-16(15)22(18(17)23)13-14-7-3-2-4-8-14/h2-10,17H,11-13H2,1H3

***tert*-Butyl (1-benzyl-2-oxoindolin-3-yl)(3-chloropropyl)carbamate (29)**

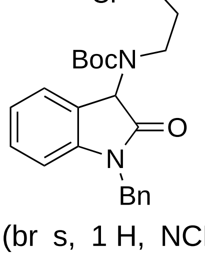

Following general procedure **A** on 2.0 mmol scale using diazo **1** and protected amine **S39** in toluene. Purification by flash chromatography (CH_2Cl_2 to 3% Et_2O/CH_2Cl_2) afforded *tert*-butyl (1-benzyl-2-oxoindolin-3-yl)(3-chloropropyl)carbamate **29** (270 mg, 65%) as a brown solid. R_f 0.40 (3% Et_2O/CH_2Cl_2); mp = 104–105 ºC; ν_{max} (film)/cm^{-1} 2980, 2947, 2931, 1718 (C=O amide), 1699 (C=O carbamate), 1612, 1417, 1362, 1347, 1250, 1149, 746, 700; ^{1}H NMR (400 MHz, DMSO-*d*^{6}, 373 K) δ 7.43–7.39 (m, 2 H, 2 × HC_{Ar}), 7.35–7.30 (m, 2 H, 2 × HC_{Ar}), 7.29–7.20 (m, 3 H, 3 × HC_{Ar}), 7.05–7.01 (m, 1 H, HC_{Ar}), 6.88 (d, *J* = 8.1 Hz, 1 H, HC_{Ar}), 5.21 (br s, 1 H, NCH), 5.00 (d, *J* = 15.8 Hz, 1 H, PhC*H*H), 4.76 (d, *J* = 15.8 Hz, 1 H, PhCH*H*), 3.75–3.40 (NC*H*$_2$CH$_2$C*H*$_2$Cl), 2.16–2.02 (m, 2 H, NCH$_2$C*H*$_2$), 1.19 (br s, 9 H, $C(CH_3)_3$); ^{13}C NMR (101 MHz, $CDCl_3$) δ 173.4 (C=O amide), 153.5 (C=O carbamate), 142.2 (NC_{Ar} quat), 135.8 (C_{Ar} quat), 127.9 (3 × C_{Ar}), 127.8 (C_{Ar}), 126.9 (2 × C_{Ar}), 126.7 (C_{Ar}), 122.3 (C_{Ar} quat), 121.6 (C_{Ar}), 108.4 (C_{Ar}), 79.4 (*C*($CH_3)_3$), 59.5 (NCH), 45.6

(CH_2Cl), 42.8 ($PhCH_2$), 42.1 (NCH_2), 31.6 (NCH_2CH_2), 27.2 ($C(CH_3)_3$); HRMS (ESI$^+$) *m/z* Calculated for $C_{23}H_{28}N_2O_3Cl$ [M+H] 415.1791; Found 415.1788.

SMILES: O=C1N(CC2=CC=CC=C2)C3=CC=CC=C3C1N(CCCCl)C(OC(C)(C)C)=O
InChI=1S/C23H27ClN2O3/c1-23(2,3)29-22(28)25(15-9-14-24)20-18-12-7-8-13-19(18)26(21(20)27)16-17-10-5-4-6-11-17/h4-8,10-13,20H,9,14-16H2,1-3H3

(*E*)-1,1'-Dimethyl-[3,3'-biindolinylidene]-2,2'-dione (S59)

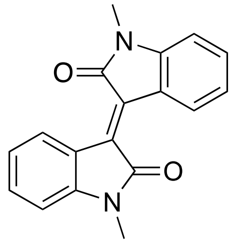

A solution of diazo **S24** (66 mg, 0.38 mmol) in CH_2Cl_2 (0.5 mL) was slowly added over 10 min to a stirred solution of 3-bromopropan-1-ol (35 mg, 0.25 mmol) and $[Cu(MeCN)_4]PF_6$ (4.7 mg, 0.01 mmol) in CH_2Cl_2 (0.2 mL) at 40 ºC. The remaining solution of diazo **S24** was washed with CH_2Cl_2 (0.1 mL) to ensure quantitative transfer. The reaction mixture was stirred for 18 h at 40 ºC and then concentrated under reduced pressure. Purification by flash chromatography (50% CH_2Cl_2/heptane to CH_2Cl_2) afforded (*E*)-1,1'-dimethyl-[3,3'-biindolinylidene]-2,2'-dione **S24** (34 mg, 62%) as a purple solid. R_f 0.22 (CH_2Cl_2); mp = 275 ºC [lit = 268 ºC][22]; ν_{max} (film)/cm^{-1} 1677 (C=O), 1603, 1469, 1370, 1334, 1076, 772; ^{1}H NMR (400 MHz, $CDCl_3$) δ 9.24–9.22 (m, 2 H, 2 × HC_{Ar}), 7.39 (td, *J* = 7.7, 1.2 Hz, 2 H, 2 × HC_{Ar}), 7.08 (td, *J* = 8.0, 1.2 Hz, 2 H, 2 × HC_{Ar}), 6.80 (dd, *J* = 7.8, 0.5 Hz, 2 H, 2 × HC_{Ar}), 3.30 (s, 6 H, 2 × NCH_3); ^{13}C NMR (101 MHz, $CDCl_3$) δ 168.0 (2 × C=O), 145.2 (2 × NC_{Ar} quat), 133.4 (2 × C_{Ar} quat), 132.4 (2 × C_{Ar}), 129.9 (2 × C_{Ar}), 122.4 (2 × C_{Ar}), 121.6 (*C*=*C*), 107.6 (2 × C_{Ar}), 26.1 (2 × NCH_3). The observed characterisation data (^{1}H and ^{13}C NMR) was consistent with that previously reported in the literature.[23]

SMILES: CN(C/1=O)C2=CC=CC=C2C1=C(C(C=CC=C3)=C3N4C)/C4=O
InChI=1S/C18H14N2O2/c1-19-13-9-5-3-7-11(13)15(17(19)21)16-12-8-4-6-10-14(12)20(2)18(16)22/h3-10H,1-2H3/b16-15+

Cyclised Products

General Procedure **B** (One-Pot)

A solution of diazo (1.0 equiv) in CH_2Cl_2 or toluene (0.16 M) was slowly added over 10 min to a stirred solution of the appropriate protected amine (2.0 equiv) and $Rh_2(esp)_2$ (0.25 mol%) in CH_2Cl_2 or toluene (0.3 M) at 25 ºC. The remaining diazo solution was washed with CH_2Cl_2 or toluene (1 M) to ensure quantitative transfer. After 30 min, CsOH·H_2O (2 equiv) and tetrabutylammonium bromide (10 mol%) were added and the reaction mixture was stirred for the stated time (0.5–3 h) at 25 ºC. The reaction mixture was filtered through celite, washing with Et_2O (25 mL) and concentrated under reduced pressure followed by purification by flash chromatography.

General Procedure **C** (From cyclisation precursor)

From the stated cyclisation precursor in toluene or CH_2Cl_2 (0.1 M), CsOH·H_2O (2 equiv) and tetrabutylammonium bromide (10 mol%) were added and the reaction mixture was stirred for the stated time (0.5–3 h) at 25 ºC. The reaction mixture was filtered through celite, washing with Et_2O (25 mL) and concentrated under reduced pressure followed by purification by flash chromatography.

General Procedure **D** (Enantioselective)

CsOH·H_2O (5 equiv) was added and to the stated cyclisation precursor and stated catalyst (20 mol%) in *m*-xylene (0.1 M) and the reaction stirred (900 rpm) at 25 ºC for 1 h. The reaction mixture was filtered through celite, washing with Et_2O (25 mL) and concentrated under reduced pressure followed by purification by flash chromatography.

General Procedure **E** (Telescoped Enantioselective)

A solution of diazo (1.0 equiv) in CH_2Cl_2 (0.16 M) was slowly added over 10 min to a stirred solution of the appropriate protected amine (2.0 equiv) and $Rh_2(esp)_2$ (0.25 mol%) in CH_2Cl_2 (0.3 M) at 25 ºC. The remaining diazo solution was washed with CH_2Cl_2 (1 M) to ensure quantitative transfer. After 30 min, the solvent was removed under reduced pressure. Then **Cat7** (20 mol%) and *m*-xylene (0.1 M) were added. CsOH·H_2O (5 equiv) was added and the reaction mixture was stirred (900 rpm) at 25 ºC for 1 h. The reaction mixture was filtered through celite, washing with Et_2O (25 mL) and concentrated under reduced pressure followed by purification by flash chromatography.

(±)-*tert*-Butyl 1'-benzyl-2'-oxospiro[azetidine-2,3'-indoline]-1-carboxylate (3)

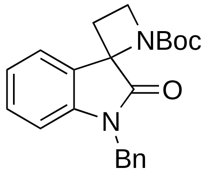

Following general procedure **B** on 0.5 mmol scale using diazo **1** and protected amine **S33** in toluene. Purification by flash chromatography (40% Et_2O/pentane) afforded *tert*-butyl 1'-benzyl-2'-oxospiro[azetidine-2,3'-indoline]-1-carboxylate **3** (116 mg, 64%) as a brown solid. R_f 0.14 (40% Et_2O/pentane); mp = 41–43 ºC; ν_{max} (film)/cm^{-1} 2973, 1718 (C=O amide), 1701 (C=O carbamate), 1614, 1467, 1363, 1346, 1177, 1146, 1099, 966, 748, 695; ^{1}H NMR (400 MHz, $CDCl_3$) δ 7.59–7.55 (m, 1 H, HC_{Ar}), 7.42–7.19 (m, 5 H, 5 × HC_{Ar}), 7.11–7.03 (m, 1 H, HC_{Ar}), 6.96 and 6.78 (2 × d, *J* = 7.8 Hz, 1 H, HC_{Ar}), 5.00–4.74 (m, 2 H, NCH_2Ph), 4.16–3.98 (m, 2 H, NCH_2), 2.58–2.44 (NCH_2CH_2), 1.36 and 0.94 (2 × s, 9 H, $C(CH_3)_3$); ^{13}C NMR (101 MHz, $CDCl_3$) δ 175.3 and 175.2 (C=O amide), 154.8 and 154.4 (C=O carbamate), 143.0 and 142.8 (NC_{Ar} quat), 136.7 and 136.5 (C_{Ar} quat), 130.0 (C_{Ar}), 129.12, 129.06, 129.0 (3 × C_{Ar}), 128.2, 128.0, 127.8, 127.6 (2 × C_{Ar} and C_{Ar} quat), 124.4 and 124.1 (C_{Ar}), 123.2 (C_{Ar}), 109.6 and 109.3 (C_{Ar}), 80.2 and 79.5 ($C(CH_3)_3$), 68.8 and 68.3 (NC quat), 47.5 and 46.1 (NCH_2), 43.4 and 43.2 (NCH_2Ph), 28.5 and 27.9 ($C(CH_3)_3$), 27.5 and 27.3 (NCH_2CH_2); HRMS (ESI$^+$) *m/z* Calculated for $C_{22}H_{24}N_2O_3Na$ [M+Na] 387.1685; Found 387.1676.

SMILES: O=C1N(CC2=CC=CC=C2)C3=CC=CC=C3C14N(CC4)C(OC(C)(C)C)=O
InChI=1S/C22H24N2O3/c1-21(2,3)27-20(26)24-14-13-22(24)17-11-7-8-12-18(17)23(19(22)25)15-16-9-5-4-6-10-16/h4-12H,13-15H2,1-3H3

(–)–*tert*-Butyl 1'-benzyl-2'-oxospiro[azetidine-2,3'-indoline]-1-carboxylate ((*S*)–3)

Following general procedure **D** on 0.2 mmol scale using cyclisation precursor **2a** and **Cat7** with purification by flash chromatography (40% Et_2O/pentane) afforded *tert*-butyl 1'-benzyl-5'-fluoro-2'-oxospiro[azetidine-2,3'-indoline]-1-carboxylate **(*S*)–3** (68 mg, 94%) as a pale yellow solid. The characterisation data observed was consistent with the above. The *er* of the product was 3:97 [Chiralpak ID column, 90:10 *n*-hexane:*i*-PrOH, flow rate: 1 mL min^{-1}, 35 ºC, UV detection wavelength: 254 nm, *rac* **3** retention times 18.03 and 27.55 min]; $[\alpha]_D^{22} = -55$ (*c* = 0.62, CH_2Cl_2).

(+)–*tert*-Butyl 1'-benzyl-2'-oxospiro[azetidine-2,3'-indoline]-1-carboxylate ((*R*)–3)

Following general procedure **D** on 0.2 mmol scale using cyclisation precursor **2a** and **Cat8** with purification by flash chromatography (50% Et_2O/pentane) afforded *tert*-butyl 1'-benzyl-5'-fluoro-2'-oxospiro[azetidine-2,3'-indoline]-1-carboxylate **(*R*)–3** (65 mg, 89%) as a pale yellow solid. The characterisation data observed was consistent with the above. The *er* of the product was 96:4 [Chiralpak ID column, 90:10 *n*-hexane:*i*-PrOH, flow rate: 1 mL min^{-1}, 35 ºC, UV detection wavelength: 254 nm, *rac* **3** retention times 18.03 and 27.55 min]; $[\alpha]_D^{22}$ = +38º (*c* = 1.83, Et_2O).

(±)–*tert*-Butyl 1'-benzyl-5'-methoxy-2'-oxospiro[azetidine-2,3'-indoline]-1-carboxylate (4)

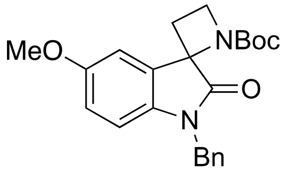

Following general procedure **B** on 0.25 mmol scale using diazo **S3** and protected amine **S33** in CH_2Cl_2. Purification by flash chromatography (40% EtOAc/hexane) afforded *tert*-butyl 1'-benzyl-5'-methoxy-2'-oxospiro[azetidine-2,3'-indoline]-1-carboxylate **4** (100 mg, quant) as an off-white solid. R_f 0.19 (30% EtOAc/hexane); mp = 166–167 ºC; ν_{max} (film)/cm^{-1} 2973, 2896, 1718 (C=O amide), 1702 (C=O carbamate), 1604, 1495, 1477, 1435, 1389, 1366, 1178, 1154, 1036; ^{1}H NMR (400 MHz, $CDCl_3$) δ 7.38–7.22 (m, 5 H, 5 × HC_{Ar}), 7.09 and 7.07 (2 × d, *J* = 2.5 Hz, 1 H, HC_{Ar}), 6.74 and 6.68 (2 × dd, *J* = 8.5, 2.5 Hz, 1 H, HC_{Ar}), 6.62 and 6.51 (2 × d, *J* = 8.5 Hz, 1 H, HC_{Ar}), 5.02 and 4.97 (2 × d, *J* = 15.5 Hz, 1 H, NC*H*HPh), 4.86 and 4.67 (2 × d, *J* = 15.5 Hz, 1 H, NCH*H*Ph), 4.37–4.24 (m, 1 H, NC*H*H), 4.15–3.98 (m, 1 H, NCH*H*), 3.79 and 3.77 (2 × s, 3 H, OCH_3), 2.76–2.68 and 2.64–2.56 (2 × m, 1 H, NCH_2CHH), 2.53–2.44 (m, 1 H, NCH_2CHH), 1.45 and 1.08 (2 × s, 9 H, $C(CH_3)_3$); ^{13}C NMR (101 MHz, $CDCl_3$) δ 175.5 and 175.1 (C=O amide), 156.3 (OC_{Ar} quat), 155.3 and 154.5 (C=O carbamate), 136.1 and 136.0 (C_{Ar} quat), 135.7 and 135.6 (C_{Ar} quat), 130.4 and 129.8 (C_{Ar} quat), 128.73 and 128.69 (2 × C_{Ph}), 127.70 and 127.67 (2 × C_{Ph}),

127.4 and 127.2 (C_{Ph}), 114.1 and 114.0 (C_{Ar}), 111.0 and 110.5 (C_{Ar}), 109.8 and 109.3 (C_{Ar}), 80.5 and 80.1 ($C(CH_3)_3$), 69.0 and 68.8 (NC quat), 55.9 and 55.8 (OCH_3), 47.4 and 45.9 (NCH_2), 44.1 and 43.9 (NCH_2Ph), 28.4 and 27.9 ($C(CH_3)_3$), 27.7 (NCH_2CH_2); HRMS (FTMS – pAPCI) *m/z* Calculated for $C_{23}H_{25}N_2O_4^-$ $[M–H]^-$ 393.1820; Found 393.1813.

SMILES: O=C1N(CC2=CC=CC=C2)C3=CC=C(OC)C=C3C14N(CC4)C(OC(C)(C)C)=O
InChI=1S/C23H26N2O4/c1-22(2,3)29-21(27)25-13-12-23(25)18-14-17(28-4)10-11-19(18)24(20(23)26)15-16-8-6-5-7-9-16/h5-11,14H,12-13,15H2,1-4H3

(–)–*tert*-Butyl 1'-benzyl-5'-methoxy-2'-oxospiro[azetidine-2,3'-indoline]-1-carboxylate ((*S*)–4)

Following general procedure **D** on 0.2 mmol scale using cyclisation precursor **S42** and **Cat7** with purification by flash chromatography (30% EtOAc/pentane) afforded *tert*-Butyl 1'-benzyl-5'-methoxy-2'-oxospiro[azetidine-2,3'-indoline]-1-carboxylate **(*S*)–4** (69 mg, 87%) as a white solid. The characterisation data observed was consistent with the above. The *er* of the product was 4:96 [Chiralpak IA column, 80:20 *n*-hexane:*i*-PrOH, flow rate: 1 mL min^{-1}, 35 ºC, UV detection wavelength: 254 nm, *rac* **4** retention times 8.40 and 13.11 min]; $[\alpha]_D^{22} = –80$ (*c* = 0.45, Et_2O).

(±)-*tert*-butyl 1'-benzyl-4'-methyl-2'-oxospiro[azetidine-2,3'-indoline]-1-carboxylate (5)

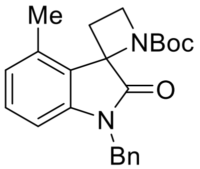

Following general procedure **C** on 0.2 mmol scale using cyclisation precursor **S43**. Purification by column chromatography (40-60% Et_2O/hexane) afforded ***t**ert*-butyl 1'-benzyl-4'-methyl-2'-oxospiro[azetidine-2,3'-indoline]-1-carboxylate **5** (45.2 mg, 72%) as an off-white solid. R_f 0.37 (40% Et_2O/hexane); mp = 65–77 ºC; ν_{max} (film)/cm^{-1} 2970, 2893, 2362, 1718 (C=O amide), 1695 (C=O carbamate), 1603, 1459, 1361, 1226, 1145, 772, 730, 697; ^1H NMR (400 MHz, $CDCl_3$) δ 7.40 – 7.21 (m, 5 H, 5 x HC_{Ar}), 7.17 – 7.02 (m, 1 H, HC_{Ar}), 6.84 (dd, *J* = 14.9, 7.8 Hz, 1 H, HC_{Ar}), 6.54 (dd, *J* = 44.3, 7.8 Hz, 1 H, HC_{Ar}), 5.05 – 4.68 (m, 2 H, NCH_2Ph), 4.42 – 4.28 (m, 1 H, $NCHHCH_2$), 4.18 – 4.02 (m, 1 H, $NCHHCH_2$), 2.75 – 2.64 (m, 2 H, NCH_2CH_2), 2.59 – 2.46 (m, 3 H, $ArCH_3$), 1.47 and 1.07 (2 x br s, 9 H, $C(CH_3)_3$); ^{13}C NMR (101 MHz, $CDCl_3$) δ 176.0 and 175.7 (C=O amide), 155.2 and 154.4 (C=O carbamate), 143.1 and 142.8 (C_{Ar} quat), 135.9 and 135.8 (C_{Ph} quat), 135.6 and 135.5 (MeC_{Ar} quat), 129.5 and 129.4 (C_{Ar} quat), 128.9 and 128.8 (2 × C_{Ar}), 127.8 (2 × C_{Ar}), 127.5 and 127.3 (C_{Ar}), 125.6 and 125.4 (C_{Ar}), 125.2 and 125.1 (C_{Ar}), 107.3 and 106.7 (C_{Ar}), 80.5 and 80.0 ($C(CH_3)_3$), 69.2 and 68.9 (NC quat), 47.0 and 45.5 (NCH_2CH_2), 44.1 and 44.0 (NCH_2Ph), 28.5 and 28.0 ($C(CH_3)_3$), 25.1 and 25.1 (NCH_2CH_2), 17.1 and 17.0 ($ArCH_3$); HRMS (ESI^+) *m/z* Calculated for $C_{23}H_{26}N_2O_3$ $[M+H]^+$ 378.1943; Found 378.1967.

SMILES: O=C1N(CC2=CC=CC=C2)C3=CC=CC(C)=C3C14N(CC4)C(OC(C)(C)C)=O
InChI=1S/C23H26N2O3/c1-16-9-8-12-18-19(16)23(13-14-25(23)21(27)28-22(2,3)4)20(26)24(18)15-17-10-6-5-7-11-17/h5-12H,13-15H2,1-4H3

(–)–*tert*-butyl 1'-benzyl-4'-methyl-2'-oxospiro[azetidine-2,3'-indoline]-1-carboxylate ((*S*)–5)

Prepared according to general procedure **D** on 0.07 mmol scale using cyclisation precursor **S43** and **Cat7** with purification by column chromatography (40-60% Et_2O/hexanes) afforded *tert*-butyl 1'-benzyl-4'-methyl-2'-oxospiro[azetidine-2,3'-indoline]-1-carboxylate **(*S*)–5** (17.4 mg, 69%) as a white solid. The characterisation data was consisted with the above. The *er* of the product was 84:16 [Chiralpak IA column, 90:10 n-hexane:i-PrOH, flow rate: 1 mL min^{-1} , 30 ºC, UV detection wavelength: 254 nm, *rac* **5** retention times 9.51 and 11.21 min]; $[\alpha]_D^{22} = –18$ (*c* = 0.35, Et_2O).

(±)-*tert*-Butyl 1'-benzyl-5'-methyl-2'-oxospiro[azetidine-2,3'-indoline]-1-carboxylate (6)

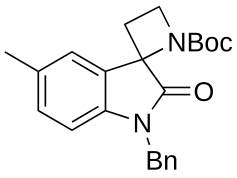

Following general procedure **B** on 0.25 mmol scale using diazo **S6** and protected amine **S33** in toluene. Purification by flash chromatography (40% Et_2O/pentane) afforded *tert*-butyl 1'-benzyl-5'-methyl-2'-oxospiro[azetidine-2,3'-indoline]-1-carboxylate **6** (55 mg, 58%) as a maroon solid. R_f 0.20 (40% Et_2O/pentane); mp = 65–66 ºC; ν_{max} (film)/cm^{-1} 3027, 2967, 2919, 2894, 1718 (C=O amide), 1701 (C=O carbamate), 1602, 1496, 1455, 1477, 1388, 1363, 1340, 1299, 1176, 1146, 969, 807, 775, 761, 731, 714, 697; ^{1}H NMR (400 MHz, DMSO-d_6) δ 7.42–7.19 (m, 6 H, 6 × HC_{Ar}), 7.07 and 7.02 (2 × ddd, J = 7.9, 1.6, 0.6 Hz, 1 H, HC_{Ar}), 6.83 and 6.65 (2 × d, J = 8.0 Hz, 1 H, HC_{Ar}), 4.95 (d, J = 15.6 Hz, 1 H, NC*H*HPh), 4.79 and 4.73 (2 × d, J = 15.9 Hz, 1 H, NCH*H*Ph), 4.15–3.97 (m, 2 H, NCH_2), 2.58–2.42 (m, 2 H, NCH_2CH_2), 2.28 and 2.26 (2 × s, 3 H, $ArCH_3$), 1.36 and 0.96 (2 × s, 9 H, $C(CH_3)_3$); ^{13}C NMR (101 MHz, DMSO-d_6) δ 174.8 and 174.6 (C=O amide), 154.3 and 154.0 (C=O carbamate), 140.1 and 140.0 (C_{Ar} quat), 136.3 and 136.0 (C_{Ar} quat), 131.8 (C_{Ar} quat), 129.6, 128.7, 128.5 and 128.4 (3 × C_{Ar} and C_{Ar} quat), 127.6 and 127.4 (2 × C_{Ar}), 127.2 and 127.1 (C_{Ar}), 124.5 and 124.3 (C_{Ar}), 108.9 and 108.6 (C_{Ar}), 79.7 and 79.0 ($C(CH_3)_3$), 68.4 and 67.9 (NC quat), 47.0 and 45.6 (NCH_2), 42.9 and 42.7 (NCH_2Ph), 28.0 and 27.5 ($C(CH_3)_3$), 27.0 and 26.8 (NCH_2CH_2), 20.1 ($ArCH_3$); HRMS (ESI$^+$) *m/z* Calculated for $C_{23}H_{26}N_2O_3Na$ [M+Na] 401.1841; Found 401.1848.

SMILES: O=C1N(CC2=CC=CC=C2)C3=CC=C(C)C=C3C14N(CC4)C(OC(C)(C)C)=O
InChI=1S/C23H26N2O3/c1-16-10-11-19-18(14-16)23(12-13-25(23)21(27)28-22(2,3)4)20(26)24(19)15-17-8-6-5-7-9-17/h5-11,14H,12-13,15H2,1-4H3

(–)–*tert*-Butyl 1'-benzyl-5'-methyl-2'-oxospiro[azetidine-2,3'-indoline]-1-carboxylate ((*S*)–6)

Following general procedure **D** on 0.2 mmol scale using cyclisation precursor **S44** and **Cat7** with purification by flash chromatography (40% Et_2O/pentane) afforded *tert*-butyl 1'-benzyl-5'-methyl-2'-oxospiro[azetidine-2,3'-indoline]-1-carboxylate **(*S*)–7** (66 mg, 87%) as a pale yellow solid. The characterisation data observed was consistent with the above. The *er* of the product was 3:97 [Chiralpak ID column, 90:10 *n*-hexane:*i*-PrOH, flow rate: 1 mL min^{-1}, 35 ºC, UV detection wavelength: 254 nm, *rac* **6** retention times 15.79 and 24.07 min]; $[\alpha]_D^{22} = -25$ (c = 0.55, Et_2O).

(±)–*tert*-Butyl 1'-benzyl-5'-fluoro-2'-oxospiro[azetidine-2,3'-indoline]-1-carboxylate (7)

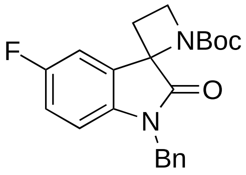

Following general procedure **C** on 0.1 mmol scale using cyclisation precursor **S45**. Purification by flash chromatography (CH_2Cl_2 to 2% Et_2O/CH_2Cl_2 to 4% Et_2O/CH_2Cl_2) afforded *tert*-butyl 1'-benzyl-5'-fluoro-2'-oxospiro[azetidine-2,3'-indoline]-1-carboxylate **7** (18 mg, 66%) as a white solid. R_f 0.10 (CH_2Cl_2); mp = 142–145 ºC; ν_{max} (film)/cm^{-1} 2974, 2930, 1722 (2 × C=O), 1491, 1457, 1364, 1156; ^{1}H NMR (400 MHz, $CDCl_3$) δ 7.37–7.19 (m, 6 H, 6 × HC_{Ar}), 6.92 and 6.85 (2 × td, J = 8.8, 2.5 Hz, 1 H, HC_{Ar}), 6.64 and 6.53 (2 × dd, J = 8.6, 4.0 Hz, 1 H, HC_{Ar}), 5.04 and 4.98 (2 × d, J = 15.8 and 15.5 Hz, 1 H, NC*H*HPh), 4.87 and 4.68 (2 × d, J = 15.8 and 15.5 Hz, 1 H, NCH*H*Ph), 4.38–4.26 (m, 1 H, NC*H*H), 4.12–3.99 (m, 1 H, NCH*H*), 2.76–2.57 (m, 1 H, NCH_2C*H*H), 2.54–2.45 (m, 1 H, NCH_2CH*H*), 1.45 and 1.09 (2 × s, 9 H, $C(CH_3)_3$); ^{13}C NMR (101 MHz, $CDCl_3$) δ 175.6 and 175.2 (C=O amide), 159.51 and 159.42 (2 × d, J_{C-F} = 241.7 Hz, FC_{Ar} quat), 155.3 and 154.3 (C=O carbamate), 138.6 and 138.4 (C_{Ar} quat), 135.3 and 135.2 (C_{Ar} quat), 130.7 and 130.1 (2 × d, J_{C-F} = 7.6 Hz, C_{Ar} quat), 128.8 (2 × C_{Ar}), 127.9, 127.6, 127.5 and 127.1 (3 × C_{Ar}), 115.8 and 115.7 (2 × d, J_{C-F} = 23.4 Hz, C_{Ar}), 111.8 and 111.6 (2 × d, J_{C-F} = 25.1 Hz, C_{Ar}), 110.0 and 109.4 (2 × d, J_{C-F} = 8.0 Hz, C_{Ar}), 80.8 and 80.3 ($C(CH_3)_3$), 68.7 and 68.4 (NC quat), 47.4 and 45.9 (NCH_2), 44.1 and 44.0 (NCH_2Ph), 28.3 and 27.9 ($C(CH_3)_3$), 27.62 and 26.55 (NCH_2CH_2); ^{19}F NMR (377 MHz, $CDCl_3$) δ –119.9 and –120.2; HRMS (ESI$^+$) *m/z* Calculated for $C_{22}H_{27}N_3O_3F$ [M+NH$_4$] 400.2036; Found 400.2030.

SMILES: O=C1N(CC2=CC=CC=C2)C3=CC=C(F)C=C3C14N(CC4)C(OC(C)(C)C)=O

InChI=1S/C22H23FN2O3/c1-21(2,3)28-20(27)25-12-11-22(25)17-13-16(23)9-10-18(17)24(19(22)26)14-15-7-5-4-6-8-15/h4-10,13H,11-12,14H2,1-3H3

(–)–*tert*-Butyl 1'-benzyl-5'-fluoro-2'-oxospiro[azetidine-2,3'-indoline]-1-carboxylate ((*S*)–7)

Following general procedure **D** on 0.2 mmol scale using cyclisation precursor **S45** and **Cat7** with purification by flash chromatography (40% Et_2O/pentane) afforded *tert*-butyl 1'-benzyl-5'-fluoro-2'-oxospiro[azetidine-2,3'-indoline]-1-carboxylate **(*S*)–7** (71 mg, 92%) as a white solid. The characterisation data observed was consistent with the above. The *er* of the product was 2:98 [Chiralpak ID column, 90:10 *n*-hexane:*i*-PrOH, flow rate: 1 mL min^{-1}, 35 ºC, UV detection wavelength: 254 nm, *rac* **7** retention times 14.06 and 34.18 min]; $[\alpha]_D^{22}$ = –37 (*c* = 0.38, Et_2O).

(±)–*tert*-Butyl 1'-benzyl-7'-fluoro-2'-oxospiro[azetidine-2,3'-indoline]-1-carboxylate (8)

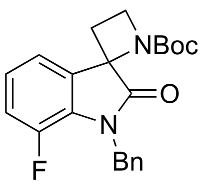

Following general procedure **B** on 0.25 mmol scale using diazo **S10** and protected amine **S33** and in CH_2Cl_2. Purification by flash chromatography (40% Et_2O/pentane then 2% Et_2O/CH_2Cl_2) afforded *tert*-butyl 1'-benzyl-7'-fluoro-2'-oxospiro[azetidine-2,3'-indoline]-1-carboxylate **8** (50 mg, 52%) as a white solid. R_f 0.16 (30% Et_2O/pentane); mp = 151–153 ºC; ν_{max} (film)/cm^{-1} 2974, 2933, 2896, 1727 (C=O carbamate), 1702 (C=O amide), 1633, 1487, 1472, 1365, 1345, 1245, 1170, 1153, 782, 727, 699; ^{1}H NMR (400 MHz, $CDCl_3$) δ 7.45–7.38 (m, 2 H, 2 × HC_{Ar}), 7.35–7.22 (m, 4 H, 4 × HC_{Ar}), 7.07–6.91 (m, 2 H, 2 × HC_{Ar}), 5.13–5.06 and 4.96–4.91 (2 × m, 2 H, NCH_2Ph), 4.36–4.24 (m, 1 H, NC*H*H), 4.12–3.98 (m, 1 H, NCH*H*), 2.75–2.68 and 2.63–2.55 (m, 1 H, NCH_2C*H*H), 2.52–2.43 (m, 1 H, NCH_2CH*H*), 1.45 and 1.04 (2 × s, 9 H, $(CH_3)_3$); ^{13}C NMR (101 MHz, $CDCl_3$) δ 175.6 and 175.1 (C=O amide), 155.2 and 154.2 (C=O carbamate), 148.4 and 146.0 (2 × d, $J_{C–F}$ = 244.3 Hz, FC_{Ar} quat), 136.8 (C_{Ph} quat), 135.1 and 131.6 (2 × d, $J_{C–F}$ = 2.7 Hz, C_{Ar} quat), 129.2 (d, $J_{C–F}$ = 8.5 Hz, C_{Ar} quat), 128.6, 128.5, 128.1, 127.7 and 127.4 (5 × C_{Ph}), 123.7 and 123.6 (2 × d, $J_{C–F}$ = 6.4 Hz, C_{Ar}), 119.6 and 119.4 (2 × d, $J_{C–F}$ = 2.6 Hz, C_{Ar}), 117.8 and 117.7 (2 × d, $J_{C–F}$ = 19.8 Hz, C_{Ar}), 80.8 and 80.3 (*C*$(CH_3)_3$), 68.6 and 68.4 (NC quat), 47.3 and 45.9 (NCH_2), 45.6 and 45.5 (NCH_2Ph), 28.3, 28.0, 27.9 and 27.8 (C(*C*$H_3)_3$ and NCH_2*C*H_2); ^{19}F{^{1}H} NMR (377 MHz, $CDCl_3$) δ –134.1, –134.2; HRMS (ESI$^+$) *m/z* Calculated for $C_{22}H_{27}N_3$-O_3F [M+NH$_4$] 400.2036; Found 400.2042.

SMILES: O=C1N(CC2=CC=CC=C2)C3=C(F)C=CC=C3C14N(CC4)C(OC(C)(C)C)=O
InChI=1S/C22H23FN2O3/c1-21(2,3)28-20(27)25-13-12-22(25)16-10-7-11-17(23)18(16)24(19(22)26)14-15-8-5-4-6-9-15/h4-11H,12-14H2,1-3H3

(–)–*tert*-Butyl 1'-benzyl-7'-fluoro-2'-oxospiro[azetidine-2,3'-indoline]-1-carboxylate ((*S*)–8)

Following general procedure **D** on 0.2 mmol scale using cyclisation precursor **S46** and **Cat7** with purification by flash chromatography (40% Et_2O/pentane) afforded *tert*-butyl 1'-benzyl-7'-fluoro-2'-oxospiro[azetidine-2,3'-indoline]-1-carboxylate **(*S*)–8** (41 mg, 56%) as a white solid. The characterisation data observed was consistent with the above. The *er* of the product was 2:98 [Chiralpak ID column, 90:10 *n*-hexane:*i*-PrOH, flow rate: 1 mL min^{-1}, 35 ºC, UV detection wavelength: 254 nm, *rac* **8** retention times 12.32 and 18.44 min]; $[\alpha]_D^{22}$ = –32 (*c* = 1.95, Et_2O).

(±)-*tert*-Butyl 1'-benzyl-4'-bromo-2'-oxospiro[azetidine-2,3'-indoline]-1-carboxylate (9)

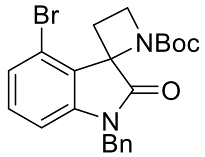

Prepared according to general procedure **C** on 0.2 mmol scale using cyclisation precursor **S47**. Purification by column chromatography (30-40% Et_2O/hexane) afforded *tert*-butyl 1'-benzyl-4'-bromo-2'-oxospiro[azetidine-2,3'-indoline]-1-carboxylate **9** (67 mg, 72%) as a white gum. R_f 0.28 (50% Et_2O/hexane); ν_{max} (film)/cm^{-1} 2970, 2893, 1725 (C=O amide), 1694 (C=O carbamate), 1603, 1582, 1451, 1364, 1146, 1020, 962, 770, 727; ^{1}H NMR (400 MHz, $CDCl_3$) δ 7.40 – 6.97 (m, 7 H, 2 × HC_{Ar} + 5 × HC_{Ph}), 6.62 (m, 1 H, HC_{Ar}), 4.89 (m, 2 H, NCH_2Ph), 4.34 – 4.12

(m, 2 H, NCH_2CH$_2$), 2.85 (m, 1 H, NCH$_2$C*H*H), 2.57 (m, 1 H, NCH$_2$CH*H*), 1.44 and 1.03 (2 × br s, 9 H, C(CH$_3$)$_3$); ^{13}C NMR (101 MHz, CDCl$_3$) δ 175.1 and 174.9 (C=O amide), 154.4 and 153.5 (C=O carbamate), 144.9 and 144.7 (NC$_{Ar}$ quat) 135.3 and 135.2 (C$_{Ph}$ quat), 131.0 and 130.9 (C$_{Ar}$ quat), 129.2 and 129.0 (C$_{Ph}$), 128.9 and 128.7 (C$_{Ph}$), 128.4 and 128.0 (C$_{Ar}$), 127.7 and 127.5 (C$_{Ar}$), 127.2 and 127.0 (C$_{Ph}$), 126.8 and 126.4 (C$_{Ph}$), 125.8 (C$_{Ph}$), 119.6 and 119.5 (BrC$_{Ar}$), 108.6 and 108.0 (C$_{Ar}$), 80.5 and 80.0 (*C*(CH$_3$)$_3$), 69.4 and 69.0 (NC quat), 47.2 and 45.8 (N*C*H$_2$CH$_2$), 44.2 and 44.1 (NCH$_2$Ph), 28.5 and 28.0 (C(*C*H$_3$)$_3$), 24.3 and 24.1 (NCH$_2$*C*H$_2$); HRMS (ESI$^+$) *m/z* Calculated for $C_{22}H_{24}{}^{79}BrN_2O_3$ [M+H] 443.0970; Found 443.0967.

SMILES: O=C1N(CC2=CC=CC=C2)C3=CC=CC(Br)=C3C14N(CC4)C(OC(C)(C)C)=O
InChI=1S/C22H23BrN2O3/c1-21(2,3)28-20(27)25-13-12-22(25)18-16(23)10-7-11-17(18)24(19(22)26)14-15-8-5-4-6-9-15/h4-11H,12-14H2,1-3H3

(–)–*tert*-Butyl 1'-benzyl-4'-bromo-2'-oxospiro[azetidine-2,3'-indoline]-1-carboxylate ((*S*)–9)

Following general procedure **D** on 0.2 mmol scale using cyclisation precursor **S47** and **Cat7** with purification using by flash chromatography (30-40% Et$_2$O/hexane) afforded *tert*-butyl 1'-benzyl-4'-bromo-2'-oxospiro[azetidine-2,3'-indoline]-1-carboxylate **(*S*)–9** (75 mg, 81%) as a white solid. The characterisation data was consisted with the above. The *er* of the product was 46:54 [Chiralpak IA column, 90:10 n-hexane:*i*-PrOH, flow rate: 1 mL min^{-1} , 30 ºC, UV detection wavelength: 254 nm, *rac* **9** retention times 11.02 and 15..25 min]; $[\alpha]_D^{22}$ = +3 (*c* = 0.45, CH$_2$Cl$_2$).

(±)–*tert*-Butyl 1'-benzyl-5'-bromo-2'-oxospiro[azetidine-2,3'-indoline]-1-carboxylate (10)

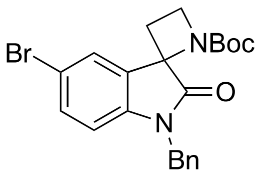

Following general procedure **B** on 0.25 mmol scale using diazo **S14** and protected amine **S33** in CH$_2$Cl$_2$. Purification by flash chromatography (30% to 40% Et$_2$O/pentane) afforded *tert*-butyl 1'-benzyl-5'-bromo-2'-oxospiro[azetidine-2,3'-indoline]-1-carboxylate **10** (76 mg, 68%) as a white solid. *R*$_f$ 0.19 (30% Et$_2$O/pentane); mp = 64–65 ºC; ν_{max} (film)/cm^{-1} 2974, 2897, 1726 (C=O amide), 1702 (C=O carbamate), 1610, 1476, 1429, 1366, 1344, 1176, 1152, 734; ^{1}H NMR (400 MHz, CDCl$_3$) δ 7.59 and 7.58 (2 × d, *J* = 1.9 Hz, 1 H, HC$_{Ar}$), 7.40–7.16 (m, 6 H, 6 × HC$_{Ar}$), 6.60 and 6.49 (2 × d, *J* = 8.3 Hz, 1 H, HC$_{Ar}$), 5.04 and 4.98 (2 × d, *J* = 15.7 Hz, 1 H, NC*H*HPh), 4.86 and 4.67 (2 × d, *J* = 15.7 Hz, 1 H, NCH*H*Ph), 4.36–4.24 (m, 1 H, NC*H*H), 4.13–3.98 (m, 1 H, NCH*H*), 2.76–2.68 and 2.64–2.56 (m, 1 H, NCH$_2$C*H*H), 2.55–2.45 (m, 1 H NCH$_2$CH*H*), 1.45 and 1.09 (2 × br s, 9 H, C(CH$_3$)$_3$); ^{13}C NMR (101 MHz, CDCl$_3$) δ 175.3 and 174.8 (C=O amide), 155.2 and 154.3 (C=O carbamate), 141.8 and 141.7 (NC$_{Ar}$ quat), 135.2 and 135.0 (C$_{Ph}$ quat), 132.4 and 132.3 (C$_{Ar}$), 131.1 and 130.6 (C$_{Ar}$ quat), 128.9 and 128.8 (2 × C$_{Ph}$), 127.9, 127.6, 127.13, 127.07 , 126.8 (3 × C$_{Ph}$ and C$_{Ar}$), 115.7 and 115.5 (C$_{Ar}$ quat), 110.9 and 110.3 (C$_{Ar}$), 80.8 and 80.4 (*C*(CH$_3$)$_3$), 68.4 and 68.1 (NC quat), 47.4 and 45.9 (NCH$_2$), 44.1 and 43.9 (NCH$_2$Ph), 28.3 and 27.9 (C(*C*H$_3$)$_3$), 27.6 and 27.5 (NCH$_2$*C*H$_2$); HRMS (FTMS + pAPCI) *m/z* Calculated for $C_{22}H_{23}N_2O_3{}^{79}Br^{\cdot+}$ $[M]^{\cdot+}$ 442.0887; Found 442.0902.

SMILES: O=C1N(CC2=CC=CC=C2)C3=CC=C(Br)C=C3C14N(CC4)C(OC(C)(C)C)=O
InChI=1S/C22H23BrN2O3/c1-21(2,3)28-20(27)25-12-11-22(25)17-13-16(23)9-10-18(17)24(19(22)26)14-15-7-5-4-6-8-15/h4-10,13H,11-12,14H2,1-3H3

(–)–*tert*-Butyl 1'-benzyl-5'-bromo-2'-oxospiro[azetidine-2,3'-indoline]-1-carboxylate ((*S*)–10)

Following general procedure **D** on 0.2 mmol scale using cyclisation precursor **S48** and **Cat7** with purification by flash chromatography (40% Et$_2$O/pentane) afforded *tert*-butyl 1'-benzyl-5'-bromo-2'-oxospiro[azetidine-2,3'-indoline]-1-carboxylate **(*S*)–10** (89 mg, 92%) as a white solid. The characterisation data observed was consistent with the above. The *er* of the product was 2:98 [Chiralpak IC column, 90:10 *n*-hexane:*i*-PrOH, flow

rate: 1 mL min^{-1}, 35 ºC, UV detection wavelength: 254 nm, *rac* **10** retention times 14.60 and 18.19 min]; $[\alpha]_D^{22}$ = –79 (*c* = 0.63, CH_2Cl_2).

(±)–*tert*-Butyl 1'-benzyl-6'-bromo-2'-oxospiro[azetidine-2,3'-indoline]-1-carboxylate (11)

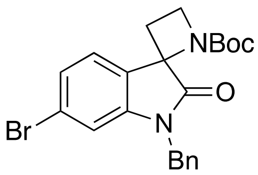

Following general procedure **B** on 0.25 mmol scale using diazo **S16** and protected amine **S33** in CH_2Cl_2. Purification by flash chromatography (40% Et_2O/pentane) afforded *tert*-butyl 1'-benzyl-6'-bromo-2'-oxospiro[azetidine-2,3'-indoline]-1-carboxylate **11** (79 mg, 71%) as a pale brown solid. R_f 0.17 (30% Et_2O/pentane); mp = 140–141 ºC; ν_{max} (film)/cm^{-1} 2974, 2931, 2896, 1727 (C=O amide), 1702 (C=O carbamate), 1608, 1487, 1428, 1389, 1366, 1338, 1178, 1154, 1114, 731; ^{1}H NMR (400 MHz, $CDCl_3$) δ 7.37–7.26 (m, 6 H, 6 × HC_{Ar}), 7.23 and 7.19 (2 × dd, *J* = 7.9, 1.6 Hz, 1 H, HC_{Ar}), 6.88 and 6.77 (2 × d, *J* = 1.6 Hz, 1 H, HC_{Ar}), 5.02, 4.96, 4.85 and 4.66 (4 × d, *J* = 15.9 or 15.5 Hz, 2 H, NCH_2Ph), 4.36–4.24 (m, 1 H, NC*H*H), 4.11–3.98 (m, 1 H, NCH*H*), 2.74–2.66 and 2.63–2.56 (m, 1 H, NCH_2C*H*H), 2.52–2.44 (m, 1 H, NCH_2CH*H*), 1.44 and 1.08 (2 × s, 9 H, $C(CH_3)_3$); ^{13}C NMR (101 MHz, $CDCl_3$) δ 175.7 and 175.2 (C=O amide), 154.3 (C=O carbamate), 144.1 and 144.0 (NC_{Ar} quat), 135.1 and 135.0 (C_{Ph} quat), 128.9 and 128.9 (2 × C_{Ph}), 128.1, 128.0, 128.0 and 127.1 (3 × C_{Ph} and C_{Ar} quat), 125.9 and 125.8 (C_{Ar}), 125.0 (C_{Ar}), 123.3 and 123.1 (BrC_{Ar} quat), 112.7 and 112.1 (C_{Ar}), 80.8 and 80.4 (*C*$(CH_3)_3$), 68.2 and 68.0 (NC quat), 47.4 and 45.9 (NCH_2), 44.1 and 44.0 (NCH_2Ph), 28.3 and 28.0 (C(*C*$H_3)_3$), 27.6 and 27.4 (NCH_2*C*H_2); HRMS (FTMS + pAPCI) *m/z* Calculated for $C_{22}H_{23}N_2O_3{}^{79}Br^{\cdot+}$ $[M]^{\cdot+}$ 442.0887; Found 442.0879.

SMILES: O=C1N(CC2=CC=CC=C2)C3=CC(Br)=CC=C3C14N(CC4)C(OC(C)(C)C)=O
InChI=1S/C22H23BrN2O3/c1-21(2,3)28-20(27)25-12-11-22(25)17-10-9-16(23)13-18(17)24(19(22)26)14-15-7-5-4-6-8-15/h4-10,13H,11-12,14H2,1-3H3

(–)–*tert*-Butyl 1'-benzyl-6'-bromo-2'-oxospiro[azetidine-2,3'-indoline]-1-carboxylate ((*S*)–11)

Following general procedure **D** on 0.2 mmol scale using cyclisation precursor **S49** and **Cat7** with purification by flash chromatography (40% Et_2O/pentane) afforded *tert*-butyl 1'-benzyl-6'-bromo-2'-oxospiro[azetidine-2,3'-indoline]-1-carboxylate **(*S*)–11** (76 mg, 85%) as a white solid. The characterisation data observed was consistent with the above. The *er* of the product was 4:96 [Chiralpak ID column, 90:10 *n*-hexane:*i*-PrOH, flow rate: 1 mL min^{-1}, 35 ºC, UV detection wavelength: 254 nm, *rac* **11** retention times 11.36 and 14.89 min]; $[\alpha]_D^{22}$ = –35 (*c* = 0.8, Et_2O).

(±)–*tert*-Butyl 1'-benzyl-7'-bromo-2'-oxospiro[azetidine-2,3'-indoline]-1-carboxylate (12)

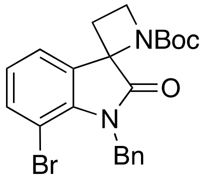

Following general procedure **C** on 0.1 mmol scale using cyclisation precursor **S50** and in toluene. Purification by flash chromatography (40% Et_2O/pentane) afforded *tert*-butyl 1'-benzyl-7'-bromo-2'-oxospiro[azetidine-2,3'-indoline]-1-carboxylate **12** (11 mg, 25%) as a white solid. R_f 0.30 (40% Et_2O/pentane); mp = 67–68 ºC; ν_{max} (film)/cm^{-1} 2973, 2933, 1731 (C=O amide), 1703 (C=O carbamate), 1612, 1450, 1390, 1366, 1346, 1161, 1123, 731; ^{1}H NMR (400 MHz, $CDCl_3$) δ 7.47–7.38 (m, 2 H, 2 × HC_{Ar}), 7.35–7.21 (m, 5 H, 5 × HC_{Ph}), 7.01–6.92 (m, 1 H, HC_{Ar}), 5.50–5.30 (m, 2 H, NCH_2Ph), 4.37–4.24 (m, 1 H, NC*H*H), 4.13–3.99 (m, 1 H, NCH*H*), 2.76–2.69 and 2.64–2.56 (m, 1 H, NCH_2C*H*H), 2.55–2.44 (m, 1 H, NCH_2CH*H*), 1.44 and 1.15 (2 × s, 9 H, $C(CH_3)_3$); ^{13}C NMR (101 MHz, $CDCl_3$) δ 176.6 and 176.2 (C=O amide), 155.2 and 154.2 (C=O carbamate), 140.3 (NC_{Ar} quat), 137.3 and 137.2 (C_{Ph} quat), 135.5 and 135.4 (C_{Ar}), 132.5 and 132.0 (C_{Ar} quat), 128.49 and 128.5 (2 × C_{Ph}), 127.2 and 126.9 (C_{Ph}), 126.7 and 126.4 (2 × C_{Ph}), 124.32 and 124.27 (C_{Ar}), 122.9 and 122.7 (C_{Ar}), 102.7 and 102.3 (BrC_{Ar} quat), 80.8 and 80.5 (*C*$(CH_3)_3$), 68.0 and 67.8 (NC quat), 47.3 and 45.9 (NCH_2), 44.7 and 44.6 (NCH_2Ph), 28.3, 28.1, 28.0 and 27.8 (C(*C*$H_3)_3$ and NCH_2*C*H_2); HRMS (FTMS + pAPCI) *m/z* Calculated for $C_{22}H_{23}N_2O_3{}^{79}Br^{\cdot+}$ $[M]^{\cdot+}$ 442.0887; Found 442.0874.

SMILES: O=C1N(CC2=CC=CC=C2)C3=C(Br)C=CC=C3C14N(CC4)C(OC(C)(C)C)=O

InChI=1S/C22H23BrN2O3/c1-21(2,3)28-20(27)25-13-12-22(25)16-10-7-11-17(23)18(16)24(19(22)26)14-15-8-5-4-6-9-15/h4-11H,12-14H2,1-3H3

(+)–*tert*-Butyl 1'-benzyl-7'-bromo-2'-oxospiro[azetidine-2,3'-indoline]-1-carboxylate ((*S*)–12)

Following general procedure **D** on 0.1 mmol scale using cyclisation precursor **S50** and **Cat7** with purification by flash chromatography (40% Et_2O/pentane) afforded *tert*-butyl 1'-benzyl-7'-bromo-2'-oxospiro[azetidine-2,3'-indoline]-1-carboxylate **(*S*)–12** (40 mg, 91%) as a white solid. The characterisation data observed was consistent with the above. The *er* of the product was 2:98 [Chiralpak ID column, 90:10 *n*-hexane:*i*-PrOH, flow rate: 1 mL min^{-1}, 35 ºC, UV detection wavelength: 254 nm, *rac* **12** retention times 14.33 and 24.52 min]; $[\alpha]_D^{22} = +8$ (c = 0.5, Et_2O).

(±)–*tert*-Butyl 1'-benzyl-5'-chloro-2'-oxospiro[azetidine-2,3'-indoline]-1-carboxylate (13)

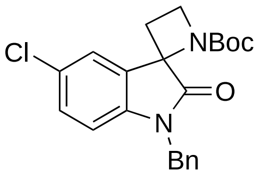

Following general procedure **C** on 0.1 mmol scale using cyclisation precursor **S51**. Purification by flash chromatography (30% Et_2O/pentane) afforded *tert*-butyl 1'-benzyl-5'-chloro-2'-oxospiro[azetidine-2,3'-indoline]-1-carboxylate **13** (22 mg, 47%) as an off-white solid. R_f 0.20 (30% Et_2O/pentane); mp = 62–63 ºC; ν_{max} (film)/cm^{-1} 2974, 2930, 2896, 1700 (br s, 2 × C=O), 1614, 1484, 1364, 1297, 1260, 1148, 1077, 969, 813, 738, 701; ^{1}H NMR (400 MHz, DMSO-d_6) δ 7.74 and 7.72 (2 × d, J = 2.2 Hz, 1 H, HC_{Ar}), 7.40–7.22 (m, 6 H, 6 × HC_{Ar}), 6.99 and 6.80 (2 × d, J = 8.4 Hz, 1 H, HC_{Ar}), 5.04–4.76 (m, 2 H, NCH_2Ph), 4.14–3.96 (m, 2 H, NCH_2), 2.60–2.43 (m, 2 H, NCH_2CH_2), 1.37 and 0.96 (2 × s, 9 H, $C(CH_3)_3$); ^{13}C NMR (101 MHz, DMSO-d_6) δ 174.6 and 174.4 (C=O amide), 154.3 and 153.6 (C=O carbamate), 141.4 and 141.2 (C_{Ar} quat), 135.9 and 135.6 (C_{Ph} quat), 130.6 and 130.4 (C_{Ar} quat), 129.3 (br s, C_{Ar}), 128.6 and 128.5 (2 × C_{Ar}), 127.7 and 127.6 (2 × C_{Ar}), 127.3, 127.1 and 126.9 (C_{Ar} quat andC_{Ar}), 124.3 and 124.1 (C_{Ar}), 110.6 and 110.4 (C_{Ar}), 79.9 and 79.2 ($C(CH_3)_3$), 68.1 and 67.6 (NC quat), 47.1 and 45.7 (NCH_2), 43.0 and 42.8 (NCH_2Ph), 27.9 and 27.4 ($C(CH_3)_3$), 26.7 and 26.6 (NCH_2CH_2); HRMS (ESI$^+$) *m/z* Calculated for $C_{22}H_{23}N_2O_3NaCl$ [M+Na] 421.1295; Found 421.1284.

SMILES: O=C1N(CC2=CC=CC=C2)C3=CC=C(Cl)C=C3C14N(CC4)C(OC(C)(C)C)=O
InChI=1S/C22H23ClN2O3/c1-21(2,3)28-20(27)25-12-11-22(25)17-13-16(23)9-10-18(17)24(19(22)26)14-15-7-5-4-6-8-15/h4-10,13H,11-12,14H2,1-3H3

(–)–*tert*-Butyl 1'-benzyl-5'-chloro-2'-oxospiro[azetidine-2,3'-indoline]-1-carboxylate ((*S*)–13)

Following general procedure **D** on 0.2 mmol scale using cyclisation precursor **S51** and **Cat7** with purification by flash chromatography (40% Et_2O/pentane) afforded *tert*-butyl 1'-benzyl-5'-chloro-2'-oxospiro[azetidine-2,3'-indoline]-1-carboxylate **(*S*)–13** (55 mg, 69%) as a paleyellow solid. The characterisation data observed was consistent with the above. The *er* of the product was 2:98 [Chiralpak IE column, 90:10 *n*-hexane:*i*-PrOH, flow rate: 1 mL min^{-1}, 35 ºC, UV detection wavelength: 254 nm, *rac* **13** retention times 16.27 and 20.00 min]; $[\alpha]_D^{22} = -95$ (c = 0.65, Et_2O).

(±)–*tert*-Butyl 1'-benzyl-5'-nitro-2'-oxospiro[azetidine-2,3'-indoline]-1-carboxylate (14)

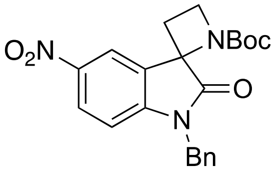

Following general procedure **C** using cyclisation precursor **S52** on 0.1 mmol scale. Purification by flash chromatography (CH_2Cl_2 to 1% to 2% Et_2O/CH_2Cl_2) afforded *tert*-butyl 1'-benzyl-5'-nitro-2'-oxospiro[azetidine-2,3'-indoline]-1-carboxylate **14** (6 mg, 15%) as a white solid. R_f 0.16 (CH_2Cl_2); mp = 75–77 ºC; ν_{max} (film)/cm^{-1} 2976, 1737 (C=O amide), 1702 (C=O carbamate), 1616, 1521, 1489, 1388, 1367, 1335 (N-O stretch), 1176, 1156; ^{1}H NMR (400 MHz, $CDCl_3$) δ 8.38 and 8.37 (2 × d, J = 2.2 Hz, 1 H, HC_{Ar}), 8.22 and 8.14 (2 × dd, J = 8.6, 2.2 Hz, 1 H, HC_{Ar}), 7.39–7.28 (m, 5 H, 5 × HC_{Ph}), 6.84 and 6.70 (2 × d, J = 8.6 Hz, 1 H, HC_{Ar}), 5.12 (d, J = 15.5 Hz, 0.5 H, NC*H*HPh), 5.07 (d, J = 16.0 Hz, 0.5 H, NCH*H*Ph), 4.92 (d, J = 16.0 Hz, 0.5 H, NCH*H*Ph), 4.73 (d, J = 15.5 Hz, 0.5 H, NC*H*HPh), 4.41–4.31 (m, 1 H, NC*H*H), 4.18–

4.05 (m, 1 H, NCH*H*), 2.78–2.55 (m, 2 H, NCH_2CH_2), 1.45 and 1.07 (2 × s, 9 H, $C(CH_3)_3$); ^{13}C NMR (101 MHz, $CDCl_3$) δ 176.1 (C=O amide), 155.2 (C=O carbamate), 148.3 (NC_{Ar} quat), 143.8 and 143.7 (NO_2C_{Ar} quat), 134.5 and 134.3 (C_{Ph} quat), 130.0 and 129.4 (C_{Ar} quat), 129.1 and 129.0 (2 × C_{Ph}), 128.3 and 128.0 (C_{Ph}), 127.7 and 127.2 (2 × C_{Ph}), 126.7 and 126.6 (C_{Ar}), 119.8 and 119.5 (C_{Ar}), 109.1 and 108.4 (C_{Ar}), 81.3 and 80.8 ($C(CH_3)_3$), 67.9 and 67.5 (NC quat), 47.4 and 46.0 (NCH_2), 44.4 and 44.3 (NCH_2Ph), 28.3 and 27.9 ($C(CH_3)_3$), 27.6 and 27.3 (NCH_2CH_2); HRMS (FTMS – pAPCI) *m/z* Calculated for $C_{22}H_{23}N_3O_5Cl^-$ $[M+Cl]^-$ 444.1332; Found 444.1331.

SMILES: O=C1N(CC2=CC=CC=C2)C3=CC=C([N+]([O-])=O)C=C3C14N(CC4)C(OC(C)(C)C)=O
InChI=1S/C22H23N3O5/c1-21(2,3)30-20(27)24-12-11-22(24)17-13-16(25(28)29)9-10-18(17)23(19(22)26)14-15-7-5-4-6-8-15/h4-10,13H,11-12,14H2,1-3H3

(–)–*tert*-Butyl 1'-benzyl-5'-nitro-2'-oxospiro[azetidine-2,3'-indoline]-1-carboxylate ((*S*)–14)

Following general procedure **D** on 0.1 mmol scale using cyclisation precursor **S52** and **Cat7** (40 mol%) with 18 h reaction time and purification by flash chromatography (50% Et_2O/pentane) afforded *tert*-Butyl 1'-benzyl-5'-nitro-2'-oxospiro[azetidine-2,3'-indoline]-1-carboxylate **(*S*)–14** (19 mg, 46%) as a white solid. The characterisation data observed was consistent with the above. The *er* of the product was 8:92 [Chiralpak IF column, 90:10 *n*-hexane:*i*-PrOH, flow rate: 1 mL min^{-1}, 35 ºC, UV detection wavelength: 254 nm, *rac* **14** retention times 14.65 and 16.43 min]; $[\alpha]_D^{22}$ = –90 (*c* = 0.20, Et_2O).

(±)-*tert*-Butyl 1'-benzyl-2'-oxo-1',2'-dihydrospiro[azetidine-2,3'-pyrrolo[2,3-b]pyridine]-1-carboxylate (15)

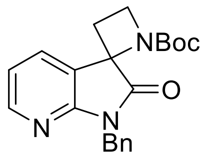

Following general procedure **C** on 0.1 scale mmol using cyclisation precursor **S53** in CH_2Cl_2. Purification by flash chromatography (50-70% Et_2O/hexane) afforded *tert*-butyl 1'-benzyl-2'-oxo-1',2'-dihydrospiro[azetidine-2,3'-pyrrolo[2,3-b]pyridine]-1-carboxylate (24.4 mg, 64%) as a white gum. R_f 0.21 (50% Et_2O/hexane); ν_{max} (film)/cm^{-1} 2966, 2923, 2891, 1722 (C=O amide), 1693 (C=O carbamate), 1591, 1447, 1363, 1176, 1144, 1098, 958, 803, 726; ^{1}H NMR (400 MHz, $CDCl_3$) δ 8.28 – 8.12 (m, 1 H, HC_{Ar}), 7.73 – 7.64 (m, 1 H, HC_{Ar}), 7.56 – 7.48 (m, 2 × HC_{Ar}), 7.38 – 7.20 (m, 3 H, 3 × HC_{Ph}), 7.06 – 6.92 (m, 1 H, HC_{Ar}), 5.13 – 4.89 (m, 2 H, NCH_2Ph), 4.37 – 4.22 (m, 1 H, NC*H*HCH2), 4.07 – 4.03 (m, 1 H, NCH*H*CH_2), 2.68 – 2.65 (m, 1 H, NCH_2C*H*H), 2.60 – 2.43 (m, 1 H, NCH_2CH*H*), 1.45 and 1.00 (2 × br s, 9 H, $C(CH_3)_3$); ^{13}C NMR (101 MHz, $CDCl_3$) δ 175.0 (C=O amide), 156.6 (C=O carbamate), 154.4 (C_{Ar} quat), 148.6 and 148.5 (C_{Ar}), 136.5 (C_{Ar} quat), 131.2 and 131.0 (C_{Ar}), 129.2 (C_{Ar} quat), 128.7 and 128.6 (C_{Ar}), 128.1, 127.8, 127.4 and 123.6 (4 × C_{Ar}), 118.7 and 118.6 (C_{Ar}), 81.0 and 80.5 ($C(CH_3)_3$), 68.1 and 67.9 (NC quat), 47.6 and 46.1 (NCH_2), 42.8 (NCH_2Ph), 28.4 and 27.9 ($C(CH_3)_3$), 27.1 and 27.0 (NCH_2CH_2). HRMS (ESI^+) *m/z* Calculated for $C_{21}H_{24}N_3O_3$ [M+H] 366.1818; Found 366.1822.

SMIELS O=C1N(CC2=CC=CC=C2)C3=NC=CC=C3C14N(CC4)C(OC(C)(C)C)=O
InChI=1S/C21H23N3O3/c1-20(2,3)27-19(26)24-13-11-21(24)16-10-7-12-22-17(16)23(18(21)25)14-15-8-5-4-6-9-15/h4-10,12H,11,13-14H2,1-3H3

(–)–*tert*-Butyl 1'-benzyl-2'-oxo-1',2'-dihydrospiro[azetidine-2,3'-pyrrolo[2,3-b]pyridine]-1-carboxylate ((*S*)–15)

Prepared according to general procedure **D** on 0.1 mmol scale using cyclisation precursor **S53** and **Cat7** with purification by flash chromatography (50-70% Et_2O/hexane) afforded tert-butyl 1'-benzyl-2'-oxo-1',2'-dihydrospiro[azetidine-2,3'- pyrrolo[2,3-b]pyridine]-1-carboxylate **(*S*)–15** (19 mg, 68%) as a white solid. The characterisation data was consisted with the above. The *er* of the product was 3:97 [Chiralpak ID column, 90:10 n-hexane:i-PrOH, flow rate: 1 mL min^{-1} , 30 ºC, UV detection wavelength: 254 nm, *rac* **15** retention times 14.16 and 18.95 min]; $[\alpha]_D^{22}$ = –26 (*c* = 0.50, Et_2O).

(±)–*tert*-Butyl 1'-methyl-2'-oxospiro[azetidine-2,3'-indoline]-1-carboxylate (16)

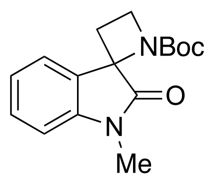

Following general procedure **B** using diazo **S24** and protected amine **S33** on 0.5 mmol scale in CH_2Cl_2. Purification by flash chromatography (0-50% EtOAc/heptane) afforded *tert*-butyl 1'-methyl-2'-oxospiro[azetidine-2,3'-indoline]-1-carboxylate **16** (73 mg, 51%) as a colourless gum. R_f 0.30 (50% EtOAc/heptane); ν_{max} (film)/cm^{-1} 2892, 2974, 1700 (br s, 2 × C=O), 1614, 1495, 1364, 1249, 1126, 1014, 969, 749, 693; ^{1}H NMR (400 MHz, DMSO-d_6) δ 7.53 (d, *J* = 7.3 Hz, 1 H, HC_{Ar}), 7.34 (td, *J* = 7.7, 1.1 Hz, 1 H, HC_{Ar}), 7.10 (t, *J* = 7.5 Hz, 1 H, HC_{Ar}), 6.96 (d, *J* = 7.8 Hz, 1 Hz, HC_{Ar}), 4.08–3.97 (m, 2 H, NCH_2), 3.13 (s, 3 H, NCH_3), 2.52–2.41 (m, NCH_2CH_2), 1.44–0.97 (br d, 9 H, $C(CH_3)_3$); ^{13}C NMR (101 MHz, DMSO-d_6) δ 174.0 (C=O amide), 153.4 (C=O carbamate), 143.2 (NC_{Ar} quat), 129.0 (C_{Ar}), 128.3 (C_{Ar} quat), 122.8 (C_{Ar}), 121.9 (C_{Ar}), 107.6 (C_{Ar}), 78.4 ($C(CH_3)_3$), 45.1 (NCH_2), 27.2 ($C(CH_3)_3$), 26.1 (NCH_2CH_2), 25.3 (NCH_3); HRMS (ESI$^+$) *m/z* Calculated for $C_{18}H_{23}N_3O_3Na$ [M+CH_3CN+Na] 352.1637; Found 352.1625.

SMILES O=C1N(C)C2=CC=CC=C2C13N(CC3)C(OC(C)(C)C)=O
InChI=1S/C16H20N2O3/c1-15(2,3)21-14(20)18-10-9-16(18)11-7-5-6-8-12(11)17(4)13(16)19/h5-8H,9-10H2,1-4H3

(–)–*tert*-Butyl 1'-methyl-2'-oxospiro[azetidine-2,3'-indoline]-1-carboxylate ((*S*)–16)

Following general procedure **D** on 0.2 mmol scale using cyclisation precursor **S54** and **Cat7** with purification by flash chromatography (10% Et_2O/CH_2Cl_2) afforded *tert*-butyl 1'-methyl-2'-oxospiro[azetidine-2,3'-indoline]-1-carboxylate **(*S*)–16** (57 mg, 98%) as a colourless gum. The characterisation data observed was consistent with the above. The *er* of the product was 97:3 [Chiralpak IA column, 95:5 *n*-hexane:*i*-PrOH, flow rate: 1 mL min^{-1}, 35 ºC, UV detection wavelength: 254 nm, *rac* **16** retention times 11.51 and 12.95 min]; $[\alpha]_D^{22} = -50$ (*c* = 2.4, Et_2O).

(±)–*tert*-Butyl 1'-(4-methoxybenzyl)-2'-oxospiro[azetidine-2,3'-indoline]-1-carboxylate (17)

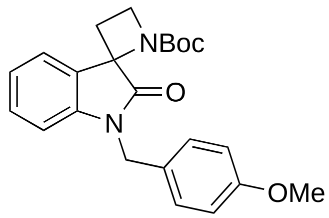

Following general procedure **B** using diazo **S25** and protected amine **S33** on 0.25 mmol scale in toluene. Purification by flash chromatography (50% Et_2O/pentane) afforded *tert*-butyl 1'-(4-methoxybenzyl)-2'-oxospiro[azetidine-2,3'-indoline]-1-carboxylate **17** (52 mg, 53%) as a pale yellow solid. R_f 0.22 (50% Et_2O/pentane); mp = 55–58 ºC; ν_{max} (film)/cm^{-1} 2971, 2896, 1700 (br s, 2 × C=O), 1614, 1465, 1349, 1245, 1148, 1029, 969, 835, 746; ^{1}H NMR (400 MHz, DMSO-d_6) δ 7.58–7.54 (m, 1H, HC_{Ar}), 7.37–7.20 (m, 3 H, HC_{Ar}), 7.08 and 7.06 (dt, *J* = 7.4, 0.7 Hz, 1 H, HC_{Ar}), 6.99 and 6.80 (d, *J* = 7.8 Hz, 1 H, HC_{Ar}), 6.89 and 6.86 (2 × d, *J* = 8.7 Hz, 2 H, 2 × HC_{Ar}), 4.89 (d, *J* = 15.3 Hz, 1 H, NC*H*HAr), 4.76 and 4.70 (2 × d, *J* = 15.3 Hz, 1 H, NCH*H*Ar), 4.16–3.98 (m, 2 H, NCH_2), 3.71 (s, 3 H, OCH_3), 2.57–2.43 (m, 2 H, NCH_2CH_2), 1.37 and 0.94 (2 × s, 9 H, $C(CH_3)_3$); ^{13}C NMR (101 MHz, DMSO-d_6) δ 174.7 and 174.6 (C=O amide), 158.6 and 158.5 (OC_{Ar} quat), 154.3 and 153.9 (C=O carbamate), 142.5 and 142.3 (C_{Ar} quat), 129.5, 129.4, 129.1, 128.7, 128.6, 128.4, 128.2 and 127.8 (3 × C_{Ar} and 2 × C_{Ar} quat), 123.8 and 123.6 (C_{Ar}), 122.6 (C_{Ar}), 113.9 and 113.8 (2 × C_{Ar}), 109.2 and 108.9 (C_{Ar}), 79.7 and 79.0 ($C(CH_3)_3$), 68.3 and 67.8 (NC quat), 55.03 and 54.99 (OCH_3), 47.0 and 46.0 (NCH_2), 42.3 and 42.1 (NCH_2Ar), 28.0 and 27.4 ($C(CH_3)_3$), 27.0 and 26.7 (NCH_2CH_2); HRMS (ESI$^+$) *m/z* Calculated for $C_{23}H_{26}N_2O_4Na$ [M+Na] 417.1790; Found 417.1790.

SMILES: O=C1N(CC2=CC=C(OC)C=C2)C3=CC=CC=C3C14N(CC4)C(OC(C)(C)C)=O
InChI=1S/C23H26N2O4/c1-22(2,3)29-21(27)25-14-13-23(25)18-7-5-6-8-19(18)24(20(23)26)15-16-9-11-17(28-4)12-10-16/h5-12H,13-15H2,1-4H3

(–)–*tert*-Butyl 1'-(4-methoxybenzyl)-2'-oxospiro[azetidine-2,3'-indoline]-1-carboxylate ((*S*)–17)

Following general procedure **D** on 0.2 mmol scale using cyclisation precursor **S55** and **Cat7** with purification by flash chromatography (50% Et_2O/pentane) afforded *tert*-butyl 1'-(4-methoxybenzyl)-2'-oxospiro[azetidine-2,3'-indoline]-1-carboxylate **(*S*)–17** (71 mg, 92%) as a white solid. The characterisation data observed was consistent with the above. The *er* of the product was 96:4 [Chiralpak IA column, 90:10 *n*-hexane:*i*-PrOH, flow rate: 1 mL min^{-1}, 35 ºC, UV detection wavelength: 254 nm, *rac* **17** retention times 12.00 and 14.68 min]; $[\alpha]_D^{22}$ = –36 (*c* = 1.85, Et_2O).

(±)–*tert*-Butyl 1'-(4-bromobenzyl)-2'-oxospiro[azetidine-2,3'-indoline]-1-carboxylate (18)

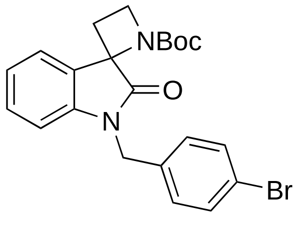

Following general procedure **B** on 0.25 mmol scale using diazo **S26** and protected amine **S33** in toluene. Purification by flash chromatography (40% Et_2O/pentane) afforded *tert*-butyl 1'-(4-bromobenzyl)-2'-oxospiro[azetidine-2,3'-indoline]-1-carboxylate **18** (70 mg, 63%) as a pale brown solid. R_f 0.24 (40% Et_2O/pentane); mp = 135–136 ºC; ν_{max} (film)/cm^{-1} 2974, 2931, 2894, 1723 (C=O amide), 1701 (C=O carbamate), 1616, 1488, 1467, 1364, 1178, 1152, 750; ^1H NMR (400 MHz, $CDCl_3$) δ 7.50–7.42 (m, 3 H, 3 × HC_{Ar}), 7.30–7.14 (m, 3 H, 3 × HC_{Ar}), 7.12–7.04 (m, 1 H, HC_{Ar}), 6.69 and 6.58 (2 × d, *J* = 7.8 Hz, 1 H, HC_{Ar}), 5.04 and 4.97 (2 × d, *J* = 15.9 Hz, 1 H, NC*H*HAr), 4.73 and 4.67 (2 × d, *J* = 15.9 Hz, 1 H, NCH*H*Ar), 4.36–4.23 (m, 1 H, NC*H*H), 4.15–4.08 and 4.06–4.00 (m, 1 H, NCH*H*), 2.75–2.67 and 2.63–2.56 (m, 1 H, NCH_2C*H*H), 2.55–2.46 (m, 1 H, NCH_2CH*H*), 1.44 and 1.05 (2 × br s, 9 H, $C(CH_3)_3$); ^{13}C NMR (101 MHz, $CDCl_3$) δ 175.8 and 175.3 (C=O amide), 154.4 (C=O carbamate), 142.5 and 142.3 (NC_{Ar} quat), 134.7 and 134.5 (C_{Ar} quat), 131.9 and 131.8 (2 × C_{Ar}), 129.64, 129.58, 129.4, 129.1, 129.0, 128.6 (3 × C_{Ar} and C_{Ar} quat), 123.9 and 123.6 (C_{Ar}), 123.1 (C_{Ar}), 121.7 and 121.4 (C_{Ar} quat), 109.1 and 108.6 (C_{Ar}), 80.6 and 80.1 (*C*$(CH_3)_3$), 68.6 and 68.4 (NC quat), 47.4 and 45.9 (NCH_2), 43.4 and 43.2 (NCH_2Ar), 28.3 and 27.9 (C(CH_3)$_3$), 27.6 and 27.5 (NCH_2CH_2); HRMS (FTMS + pAPCI) *m/z* Calculated for $C_{22}H_{23}N_2O_3{}^{79}Br^{+\cdot}$ $[M]^{+\cdot}$ 442.0887; Found 442.0873.

SMILES: O=C1N(CC2=CC=C(Br)C=C2)C3=CC=CC=C3C14N(CC4)C(OC(C)(C)C)=O
InChI=1S/C22H23BrN2O3/c1-21(2,3)28-20(27)25-13-12-22(25)17-6-4-5-7-18(17)24(19(22)26)14-15-8-10-16(23)11-9-15/h4-11H,12-14H2,1-3H3

(–)-*tert*-Butyl 1'-(4-bromobenzyl)-2'-oxospiro[azetidine-2,3'-indoline]-1-carboxylate ((*S*)–18)

Following general procedure **D** on 0.2 mmol scale using cyclisation precursor **S56** and **Cat7** with purification by flash chromatography (40% Et_2O/pentane) afforded *tert*-butyl 1'-(4-bromobenzyl)-2'-oxospiro[azetidine-2,3'-indoline]-1-carboxylate **(*S*)–18** (45 mg, 51%) as a pale brown solid. The characterisation data observed was consistent with the above. The *er* of the product was 12:88 [Chiralpak ID column, 90:10 *n*-hexane:*i*-PrOH, flow rate: 1 mL min^{-1}, 35 ºC, UV detection wavelength: 254 nm, *rac* **18** retention times 14.96 and 21.63 min]; $[\alpha]_D^{22}$ = –24 (*c* = 0.25, CH_2Cl_2).

(±)–*tert*-Butyl 1'-(3,5-bis(trifluoromethyl)benzyl)-2'-oxospiro[azetidine-2,3'-indoline]-1-carboxylate (19)

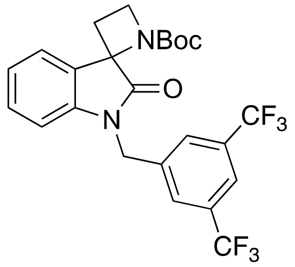

Following general procedure **B** on 0.25 mmol scale using diazo **S27** and protected amine **S33** in toluene. Purification by flash chromatography (40% Et_2O/pentane then 2% Et_2O/CH_2Cl_2) afforded *tert*-butyl 1'-(3,5-bis(trifluoromethyl)benzyl)-2'-oxospiro[azetidine-2,3'-indoline]-1-carboxylate **19** (56 mg, 45%) as a pale orange solid.
R_f 0.14 (30% Et_2O/pentane); ν_{max} (film)/cm^{-1} 2974, 1692 (2 × C=O), 1618, 1366, 1282, 1174, 1126, 973, 906, 753, 708; ^1H NMR (400 MHz, DMSO-*d_6*) δ 8.19 and 8.16 (2 × s, 2 H, 2 × HC_{Ar}), 8.08 and 7.99 (2 × s, 1 H, HC_{Ar}), 7.62 and 7.59 (2 × dd, 1 H, *J* = 7.4, 0.8 Hz, 1 H, HC_{Ar}), 7.35 (td, *J* = 7.7, 1.3 Hz, 0.4 Hz, HC_{Ar}), 7.28–7.21 (m, 1 H, HC_{Ar}), 7.13 and 7.10 (2 × td, *J*

= 7.6, 0.9 Hz, 1 H, HC_{Ar}), 6.86 (d, *J* = 7.8 Hz, 0.6 Hz, HC_{Ar}), 5.34, 5.08 and 4.91 (2 × d and s, 2 H, NCH_2Ar), 4.17–3.96 (m, 2 H, NCH_2), 2.59–2.44 (m, 2 H, NCH_2CH_2), 1.36 and 0.80 (2 × s, 9 H, $C(CH_3)_3$); ^{13}C NMR (101 MHz, DMSO-d_6) δ 175.0 (C=O amide), 154.7 and 153.6 (C=O carbamate), 142.2 and 142.0 (C_{Ar} quat), 140.0 and 139.8 (C_{Ar} quat), 130.5 and 130.4 (2 × q, J_{C-F} = 33 Hz, 2 × $C_{Ar}CF_3$ quat), 129.8, 129.2, 128.7, 128.4 and 128.2 (3 × C_{Ar}), 124.3 and 123.8 (C_{Ar}), 123.3 (q, J_{C-F} = 273 Hz, 2 × CF_3), 123.1 (2 × C_{Ar}), 121.1 (C_{Ar} quat), 108.8 and 108.7 (C_{Ar}), 80.0 and 78.8 ($C(CH_3)_3$), 68.2 and 67.7 (NC quat), 47.2 and 45.6 (NCH_2), 42.3 and 41.8 (NCH_2Ar), 27.8 and 27.1 ($C(CH_3)_3$), 26.9 and 26.4 (NCH_2CH_2); $^{19}F\{^1H\}$ NMR (377 MHz, DMSO-d_6) δ –61.2 and –61.3; HRMS (FTMS + pAPCI) *m/z* Calculated for $C_{24}H_{22}N_2O_3F_6^{\cdot+}$ $[M+H]^{\cdot+}$ 500.1529; Found 500.1517.

SMILES: O=C1N(CC2=CC(C(F)(F)F)=CC(C(F)(F)F)=C2)C3=CC=CC=C3C14N(CC4)C(OC(C)(C)C)=O
InChI=1S/C24H22F6N2O3/c1-21(2,3)35-20(34)32-9-8-22(32)17-6-4-5-7-18(17)31(19(22)33)13-14-10-15(23(25,26)27)12-16(11-14)24(28,29)30/h4-7,10-12H,8-9,13H2,1-3H3

(–)–*tert*-Butyl 1'-(3,5-bis(trifluoromethyl)benzyl)-2'-oxospiro[azetidine-2,3'-indoline]-1-carboxylate ((*S*)–19)

Following general procedure **D** on 0.2 mmol scale using cyclisation precursor **S57** and **Cat7** with purification by flash chromatography (30% Et_2O/pentane) afforded *tert*-Butyl 1'-(3,5-bis(trifluoromethyl)benzyl)-2'-oxospiro[azetidine-2,3'-indoline]-1-carboxylate **(*S*)–19** (75 mg, 75%) as a white solid. The characterisation data observed was consistent with the above. The *er* of the product was 9:91 [Chiralpak IC column, 98.5:1.5 *n*-hexane:*i*-PrOH, flow rate: 1 mL min^{-1}, 35 ºC, UV detection wavelength: 254 nm, *rac* **19** retention times 14.12 and 23.59 min]; $[\alpha]_D^{22}$ = –75 (*c* = 1.52, Et_2O).

(±)–*tert*-Butyl 1'-(naphthalen-2-ylmethyl)-2'-oxospiro[azetidine-2,3'-indoline]-1-carboxylate (20)

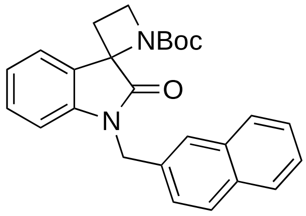

Following general procedure **B** on 0.1 mmol scale using diazo **S28.** and protected amine **S33** in CH_2Cl_2. Purification by flash chromatography (40% EtOAc/hexane) afforded *tert*-butyl 1'-(naphthalen-2-ylmethyl)-2'-oxospiro[azetidine-2,3'-indoline]-1-carboxylate **20** (25 mg, 61%) as a pale brown solid. R_f 0.17 (30% EtOAc/hexane); mp = 71–73 ºC; ν_{max} (film)/cm^{-1} 2975, 2892, 1722 (C=O amide), 1692 (C=O carbamate), 1612, 137, 1355, 1178, 1142, 745; ^1H NMR (400 MHz, $CDCl_3$) δ 7.98–7.77 (m, 4 H, 4 × HC_{Ar}), 7.61–7.45 (m, 4 H, 4 × HC_{Ar}), 7.26 and 7.20 (2 × t, *J* = 7.8 Hz, 1 H, HC_{Ar}), 7.11–7.00 (m, 1.6 H, 2 × HC_{Ar}), 6.82 (d, *J* = 7.8 Hz, 0.4 Hz, HC_{Ar}), 5.19 and 5.14 (2 × d, *J* = 16.0 Hz, 1 H, NC*H*HAr), 4.95 and 4.94 (2 × d, *J* = 16.0 Hz, 1 H, NCH*H*Ar), 4.19–3.99 (m, 2 H, NCH_2), 2.66–2.47 (m, 2 H, NCH_2CH_2), 1.40 and 0.92 (2 × s, 9 H, $C(CH_3)_3$); ^{13}C NMR (101 MHz, $CDCl_3$) δ 175.0 and 174.9 (C=O amide), 154.5 and 153.9 (C=O carbamate), 142.6 and 142.4 (NC_{Ar} quat), 133.8 and 133.4 (C_{Ar} quat), 132.9 (C_{Ar} quat), 132.3 and 132.2 (C_{Ar} quat), 129.6 (C_{Ar}), 128.7 and 128.5 (C_{Ar} quat), 128.3 and 128.1 (C_{Ar}), 127.59, 127.55 and 127.4 (2 × C_{Ar}), 126.4, 126.2, 126.0, 125.8, 125.5 and 125.4 (4 × C_{Ar}), 124.0 and 123.7 (C_{Ar}), 122.7 (C_{Ar}), 109.1 and 108.9 (C_{Ar}), 79.8 and 79.0 ($C(CH_3)_3$), 68.3 and 67.8 (NC quat), 47.1 and 45.7 (NCH_2), 43.2 and 42.8 (NCH_2Ar), 28.0 and 27.4 ($C(CH_3)_3$), 26.8 (NCH_2CH_2); HRMS (ESI^+) *m/z* Calculated for $C_{26}H_{26}N_2O_3Na$ [M+Na] 437.1841; Found 437.1827.

SMILES: O=C1N(CC2=CC=C(C=CC=C3)C3=C2)C4=CC=CC=C4C15N(CC5)C(OC(C)(C)C)=O
InChI=1S/C26H26N2O3/c1-25(2,3)31-24(30)28-15-14-26(28)21-10-6-7-11-22(21)27(23(26)29)17-18-12-13-19-8-4-5-9-20(19)16-18/h4-13,16H,14-15,17H2,1-3H3

(–)–*tert*-Butyl 1'-(naphthalen-2-ylmethyl)-2'-oxospiro[azetidine-2,3'-indoline]-1-carboxylate ((*S*)–20)

Following general procedure **D** on 0.1 mmol scale using cyclisation precursor **S58** and **Cat7** with purification by flash chromatography (30% Et_2O/pentane) afforded *tert*-Butyl 1'-(3,5-bis(trifluoromethyl)benzyl)-2'-oxospiro[azetidine-2,3'-indoline]-1-carboxylate **(*S*)–20** (31 mg, 76%) as a white solid. The characterisation data observed was consistent with the above. The *er* of the product was 19:81 [Chiralpak ID column, 90:10

n-hexane:*i*-PrOH, flow rate: 1 mL min^{-1}, 35 ºC, UV detection wavelength: 254 nm, *rac* **20** retention times 20.34 and 28.31 min]; $[\alpha]_D^{22}$ = –49 (*c* = 0.85, Et_2O).

(±)–Benzyl 1'-benzyl-2'-oxospiro[azetidine-2,3'-indoline]-1-carboxylate (21)

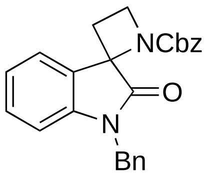

Following general procedure **B** on 0.5 mmol scale using diazo **1** and protected amine **S37** in toluene. Purification by flash chromatography (50% to 70% Et_2O/hexane) afforded benzyl 1'-benzyl-2'-oxospiro[azetidine-2,3'-indoline]-1-carboxylate **21** (113 mg, 52%) as a pale brown solid. R_f 0.30 (50% EtOAc/hexane); mp = 45–48 ºC; ν_{max} (film)/cm^{-1} 3029, 2961, 2894, 1702 (br s, 2 × C=O), 1612, 1466, 1454, 1402, 1342, 1177, 1130, 1098, 747, 729, 693; ^{1}H NMR (400 MHz, DMSO-*d*$_6$) δ 7.67–7.60 (m, 1 H, HC_{Ar}), 7.42–7.21 (m, 7 H, 7 × HC_{Ar}), 7.19–7.05 (m, 4 H, 4 × HC_{Ar}), 6.86 and 6.78 (2 × d, *J* = 7.8 Hz, 1 H, HC_{Ar}), 6.75–6.71 (m, 1 H, HC_{Ar}), 5.08–4.92 (m, 2 H, OCH_2Ph), 4.85–4.70 (m, 2 H, NCH_2Ph), 4.24–4.08 (m, 2 H, NCH_2), 2.66–2.52 (m, 2 H, NCH_2CH_2); ^{13}C NMR (101 MHz, DMSO-*d*$_6$) δ 174.5 and 174.4 (C=O amide), 154.4 and 154.1 (C=O carbamate), 142.3 (C_{Ar} quat), 136.4, 136.2 and 135.9 (2 × C_{Ar} quat), 129.9 and 129.7 (C_{Ar} quat), 128.6, 128.47, 128.45, 128.3, 128.1, 128.0, 127.7, 127.4, 127.3, 127.03, 126.95 (10 × C_{Ar}), 126.2 (C_{Ar}), 124.1 and 124.0 (C_{Ar}), 123.0 and 122.8 (C_{Ar}), 109.3 (C_{Ar}), 68.3 and 67.9 (NC quat), 66.1 and 65.5 (OCH_2Ph), 47.0 and 45.9 (NCH_2Ph), 42.8 (NCH_2), 27.6 and 27.4 (NCH_2CH_2); HRMS (ESI$^+$) *m/z* Calculated for $C_{25}H_{23}N_2O_3$ [M+H] 399.1709; Found 399.1720.

SMILES O=C1N(CC2=CC=CC=C2)C3=CC=CC=C3C14N(CC4)C(OCC5=CC=CC=C5)=O
InChI=1S/C25H22N2O3/c28-23-25(15-16-27(25)24(29)30-18-20-11-5-2-6-12-20)21-13-7-8-14-22(21)26(23)17-19-9-3-1-4-10-19/h1-14H,15-18H2

(–)–Benzyl 1'-benzyl-2'-oxospiro[azetidine-2,3'-indoline]-1-carboxylate ((*S*)–21)

Following general procedure **D** on 0.2 mmol scale using cyclisation precursor **2d** and **Cat7** with purification by flash chromatography (30% to 40% EtOAc/hexane) afforded benzyl 1'-benzyl-2'-oxospiro[azetidine-2,3'-indoline]-1-carboxylate **(*S*)–21** (71 mg, 89%) as a white gummy solid. The characterisation data observed was consistent with the above. The *er* of the product was 95:5 [Chiralpak IA column, 80:20 *n*-hexane:*i*-PrOH, flow rate: 1 mL min^{-1}, 35 ºC, UV detection wavelength: 254 nm, *rac* **21** retention times 16.03 and 15.14 min]; $[\alpha]_D^{22}$ = –49 (*c* = 0.84, Et_2O).

(±)–Methyl 1'-benzyl-2'-oxospiro[azetidine-2,3'-indoline]-1-carboxylate (22)

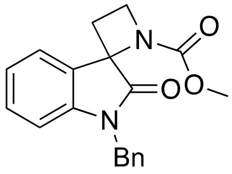

Following general procedure **B** on 0.5 mmol scale using diazo **1** and protected amine **S38** in toluene. Purification by flash chromatography (20% to 30% EtOAc/pentane) afforded methyl 1'-benzyl-2'-oxospiro[azetidine-2,3'-indoline]-1-carboxylate **22** (63 mg, 39%) as a pink solid. R_f 0.29 (40% EtOAc/pentane); mp = 102–104 ºC; ν_{max} (film)/cm^{-1} 3030, 2960, 2896, 1700 (br s, 2 × C=O), 1614, 1450, 1349, 1182, 753, 697; ^{1}H NMR (400 MHz, $CDCl_3$) δ 7.51–7.47 (m, 1 H, HC_{Ar}), 7.39–7.15 (m, 6 H, 6 × HC_{Ar}), 7.12–7.04 (m, 1 H, HC_{Ar}), 6.70 and 6.62 (2 × d, *J* = 7.8 Hz, 1 H, HC_{Ar}), 5.19, 5.03, 4.88 and 4.64 (4 × d, *J* = 15.9 Hz, NCH_2Ph), 4.42–4.31 (m, 1 H, NC*H*H), 4.18–4.10 (m, 1 H, NCH*H*), 3.68 and 3.43 (2 × s, 3 H, OCH_3), 2.78–2.67 (m, 1 H, NCH_2C*H*H), 2.61–2.51 (m, 1 H, NCH_2CH*H*); ^{13}C NMR (101 MHz, $CDCl_3$) δ 175.3 and 175.0 (C=O amide), 155.9 and 155.7 (C=O carbamate), 142.7 and 142.4 (C_{Ar} quat), 135.5 and 135.3 (C_{Ar} quat), 129.8 (C_{Ar}), 128.8 and 128.7 (2 × C_{Ar}), 127.7, 127.4, 127.2 and 127.0 (4 × C_{Ar}), 123.6 (C_{Ar}), 123.1 and 123.1 (C_{Ar} quat), 109.5 and 109.2 (C_{Ar}), 68.7 and 68.3 (NC quat), 52.5 and 52.3 (OCH_3), 47.2 and 46.2 (NCH_2Ph), 43.9 and 43.7 (NCH_2), 28.3 and 27.9 (NCH_2CH_2); HRMS (ESI$^+$) *m/z* Calculated for $C_{19}H_{19}N_2O_3$ [M+H] 323.1396; Found 323.1383.

SMILES O=C1N(CC2=CC=CC=C2)C3=CC=CC=C3C14N(C(OC)=O)CC4
InChI=1S/C19H18N2O3/c1-24-18(23)21-12-11-19(21)15-9-5-6-10-16(15)20(17(19)22)13-14-7-3-2-4-8-14/h2-10H,11-13H2,1H3

(–)–Methyl 1'-benzyl-2'-oxospiro[azetidine-2,3'-indoline]-1-carboxylate ((*S*)–22)

Following general procedure **D** on 0.1 mmol scale using cyclisation precursor **2e** and **Cat7** with purification by flash chromatography (40% EtOAc/hexane) afforded methyl 1'-benzyl-2'-oxospiro[azetidine-2,3'-indoline]-1-carboxylate **(*S*)–22** (22 mg, 69%) as a white solid. The characterisation data observed was consistent with the above. The *er* of the product was 89:11 [Chiralpak IA column, 80:20 *n*-hexane:*i*-PrOH, flow rate: 1 mL min^{-1}, 35 ºC, UV detection wavelength: 254 nm, *rac* **351** retention times 8.79 and 9.89 min]; $[\alpha]_D^{22}$ = –65 (*c* = 1.05, CH_2Cl_2).

(±)–*tert*-Butyl 1'-benzyl-5',7'-dimethyl-2'-oxospiro[azetidine-2,3'-indoline]-1-carboxylate (23)

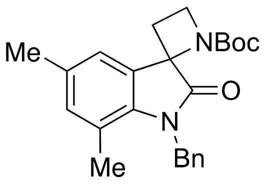

Following general procedure **B** 0.25 mmol scale using diazo **S30** and protected amine **S33** in CH_2Cl_2. Purification by flash chromatography (40% Et_2O/pentane) afforded *tert*-butyl 1'-benzyl-5',7'-dimethyl-2'-oxospiro[azetidine-2,3'-indoline]-1-carboxylate **23** (84 mg, 86%) as a white solid. R_f 0.28 (50% Et_2O/pentane); mp = 165–167 ºC; ν_{max} (film)/cm^{-1} 2895, 2973, 1718 (C=O amide), 1701 (C=O carbamate), 1604, 1480, 1454, 1389, 1365, 1347, 1252, 1169, 1153, 977, 859, 731; ^1H NMR (400 MHz, $CDCl_3$) δ 7.34–7.15 (m, 6 H, 6 × HC_{Ar}), 6.79 and 6.74 (2 × s, 1 H, HC_{Ar}), 5.28 and 5.27 (2 × d, *J* = 16.7 Hz, 1 H, NC*H*H), 5.08 and 4.98 (2 × d, *J* = 16.7 Hz, 1 H, NCH*H*), 4.36–4.21 (m, 1 H, NC*H*H), 4.16–4.09 and 4.07–3.98 (2 × m, 1 H, NCH*H*), 2.79–2.71 and 2.63–2.56 (2 × m, 1 H, NCH_2C*H*H), 2.54–2.43 (m, 1 H, NCH_2CH*H*), 1.45 and 1.16 (2 × s, 9 H, $C(CH_3)_3$); ^{13}C NMR (101 MHz, $CDCl_3$) δ 176.7 and 176.3 (C=O amide), 155.2 and 154.6 (C=O carbamate), 138.3 and 138.1 (C_{Ar} quat), 137.5 (C_{Ar} quat), 134.1 and 133.7 (C_{Ar}), 132.6 (C_{Ar} quat), 130.1 and 129.5 (C_{Ar} quat), 128.7 (2 × C_{Ph}), 127.2 and 126.9 (C_{Ph}), 125.9 (2 × C_{Ph}), 122.4 and 122.1 (C_{Ar}), 119.6 and 119.3 (C_{Ar} quat), 80.4 and 80.0 (*C*$(CH_3)_3$), 68.4 and 68.1 (NC quat), 47.3 and 45.9 (NCH_2), 45.1 (NCH_2Ph), 28.4, 28.1, 28.0 and 27.8 ($C(CH_3)_3$ and NCH_2CH_2), 20.72 and 20.67 ($ArCH_3$), 18.5 ($ArCH_3$); HRMS (FTMS + pAPCI) *m/z* Calculated for $C_{19}H_{21}N_2O^+$ $[M–(C_5H_9O_2)+2H]^+$ 293.1648; Found 293.1641.

SMILES: O=C1N(CC2=CC=CC=C2)C3=C(C)C=C(C)C=C3C14N(CC4)C(OC(C)(C)C)=O
InChI=1S/C24H28N2O3/c1-16-13-17(2)20-19(14-16)24(11-12-26(24)22(28)29-23(3,4)5)21(27)25(20)15-18-9-7-6-8-10-18/h6-10,13-14H,11-12,15H2,1-5H3

(–)–*tert*-Butyl 1'-benzyl-5',7'-dimethyl-2'-oxospiro[azetidine-2,3'-indoline]-1-carboxylate ((*S*)–23)

Following general procedure **E** on 0.2 mmol scale using diazo **S30**, protected amine **S33** and **Cat7** with purification by flash chromatography (40% to 50% Et_2O/pentane) afforded *tert*-butyl 1'-benzyl-5',7'-dimethyl-2'-oxospiro[azetidine-2,3'-indoline]-1-carboxylate **(*S*)–23** (37 mg, 47%) as a white solid. The characterisation data observed was consistent with the above. The *er* of the product was 6:94 [Chiralpak IE column, 90:10 *n*-hexane:*i*-PrOH, flow rate: 1 mL min^{-1}, 35 ºC, UV detection wavelength: 254 nm, *rac* **23** retention times 26.88 and 33.08 min]; $[\alpha]_D^{22}$ = –40 (*c* = 0.2, Et_2O).

(±)–*tert*-Butyl 1'-benzyl-4',6'-difluoro-2'-oxospiro[azetidine-2,3'-indoline]-1-carboxylate (24)

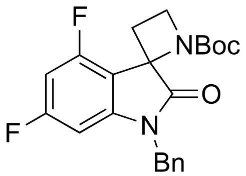

Following general procedure **B** on 0.25 mmol scale using diazo **S32** and protected amine **S33** on in CH_2Cl_2. Purification by flash chromatography (30% Et_2O/pentane) afforded *tert*-butyl 1'-benzyl-4',6'-difluoro-2'-oxospiro[azetidine-2,3'-indoline]-1-carboxylate **345** (89 mg, 89%) as a white solid. R_f 0.30 (30% Et_2O/pentane); mp = 152–154 ºC; ν_{max} (film)/cm^{-1} 2974, 2932, 2899, 1735 (C=O amide), 1701 (C=O carbamate), 1638, 1611, 1502, 1459, 1366, 1342, 1262, 1133, 1103, 967, 829, 700; ^1H NMR (400 MHz, $CDCl_3$) δ 7.37–7.25 (m, 5 H, 5 × HC_{Ph}), 6.54–6.42 (m, 1 H, HC_{Ar}), 6.35–6.31 and 6.23–6.19 (2 × m, 1 H, HC_{Ar}), 4.96 and 4.95 (2 × d, *J* = 15.5 Hz, 1 H, NC*H*H), 4.84 and 4.71 (2 × d, *J* = 15.5 Hz, NCH*H*Ph), 4.28–4.07 (m, 2 H, NCH_2), 2.78–2.60 (m, 2 H, NCH_2CH_2), 1.44 and 1.09 (2 × s, 9 H, $C(CH_3)_3$); ^{13}C NMR (101 MHz, $CDCl_3$) δ 175.2 and 174.9 (C=O amide), 165.5 and 163.0 (FC_{Ar} quat), 158.0 and 156.2 (FC_{Ar} quat), 154.8 and 153.9 (C=O carbamate), 145.6 (C_{Ar} quat), 134.8 and 134.7 (C_{Ph} quat), 129.0 and 128.9 (2 × C_{Ph}), 128.1 and 127.8

(C_{Ph}), 127.7 and 127.2 (2 × C_{Ph}), 98.4, 98.3, 98.1, 97.9 (C_{Ar}), 94.8 and 94.2 (2 × dd, $J_{C–F}$ = 27.3, 3.0 Hz, C_{Ar}), 80.7 and 80.2 ($C(CH_3)_3$), 66.9 and 66.6 (NC quat), 47.1 and. 45.5 (NCH_2), 44.5 and 44.4 (NCH_2Ph), 28.3 and 27.9 ($C(CH_3)_3$), 25.7 and 25.4 (NCH_2CH_2); $^{19}F\{^1H\}$ NMR (377 MHz, $CDCl_3$) δ –105.7 and –106.1 (2 × d, $J_{F–F}$ = 8.1 Hz), –116.5 and –117.4 (2 × d, $J_{F–F}$ = 8.1 Hz); HRMS (FTMS – pAPCI) *m/z* Calculated for $C_{22}H_{21}F_2N_2O_3^-$ $[M–H]^-$ 399.1515; Found 399.1510.

(–)–*tert*-Butyl 1'-benzyl-4',6'-difluoro-2'-oxospiro[azetidine-2,3'-indoline]-1-carboxylate ((*S*)–24)

Following general procedure **E** on 0.2 mmol scale using diazo **S32,** protected amine **S33** and **Cat7** with purification by flash chromatography (30% Et_2O/pentane) afforded *tert*-butyl 1'-benzyl-4',6'-difluoro-2'-oxospiro[azetidine-2,3'-indoline]-1-carboxylate **(*S*)–24** (36 mg, 45%) as a white solid. The characterisation data observed was consistent with the above. The *er* of the product was 15:85 [Chiralpak ID column, 90:10 *n*-hexane:*i*-PrOH, flow rate: 1 mL min^{-1}, 35 ºC, UV detection wavelength: 254 nm, *rac* **24** retention times 9.92 and 11.34 min]; $[\alpha]_D^{22}$ = –28 (*c* = 0.85, Et_2O).

(±)–*tert*-Butyl 1-benzyl-2-oxospiro[indoline-3,2'-pyrrolidine]-1'-carboxylate (30)

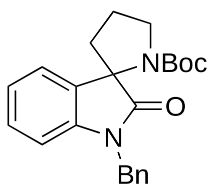

Following general procedure **B** on 0.25 mmol scale using diazo **1** and protected amine **S39** in toluene. Purification by flash chromatography (50% Et_2O/hexane) afforded *tert*-butyl 1-benzyl-2-oxospiro[indoline-3,2'-pyrrolidine]-1'-carboxylate **30** (59 mg, 62%) as a brown solid. R_f 0.26 (50% Et_2O/hexane); mp = 145–146 ºC; ν_{max} (film)/cm^{-1} 2975, 2889, 1726 (C=O amide), 1689 (C=O carbamate), 1610, 1489, 1467, 1382, 1362, 1179, 1157, 1114, 762, 697; ^1H NMR (400 MHz, DMSO-d_6, 373 K) δ 7.41–7.36 (m, 2 H, 2 × HC_{Ar}), 7.35–7.30 (m, 2 H, 2 × HC_{Ar}), 7.29–7.24 (m, 2 H, 2 × HC_{Ar}), 7.23–7.17 (m, 1 H, HC_{Ar}), 7.01 (t, *J* = 7.4 Hz, 1 H, HC_{Ar}), 6.86 (br s, 1 H, HC_{Ar}), 5.02 (br s, 1 H, NC*H*HPh), 4.69 (br s, 1 H, NCH*H*Ph), 3.77–3.62 (m, 2 H, NCH_2), 2.32–2.02 (m, 4 H, $NCH_2(CH_2)_2$), 1.00 (br s, 9 H, $C(CH_3)_3$); ^{13}C NMR (101 MHz, DMSO-d_6, 373 K) δ 176.4 (C=O amide), 151.7 (C=O carbamate), 141.6 (NC_{Ar} quat), 135.9 (C_{Ar} quat), 127.9 (2 × C_{Ar}), 127.6 (C_{Ar}), 131.9 (C_{Ar} quat), 126.8 (2 × C_{Ar}), 126.7 (C_{Ar}), 121.8 (C_{Ar}), 121.3 (C_{Ar}), 108.2 (C_{Ar}), 78.6 ($C(CH_3)_3$), 66.0 (NC quat), 47.5 (NCH_2), 42.7 (NCH_2Ph), 27.1 ($C(CH_3)_3$), 22.0 ($N(CH_2)_2CH_2$), peak due to NCH_2CH_2 obscured by DMSO residual solvent signal confirmed by HSQC; HRMS (ESI^+) *m/z* Calculated for $C_{23}H_{26}N_2O_3Na$ [M+Na] 401.1841; Found 401.1850.

SMILES O=C1N(CC2=CC=CC=C2)C3=CC=CC=C3C14N(CCC4)C(OC(C)(C)C)=O
InChI=1S/C23H26N2O3/c1-22(2,3)28-21(27)25-15-9-14-23(25)18-12-7-8-13-19(18)24(20(23)26)16-17-10-5-4-6-11-17/h4-8,10-13H,9,14-16H2,1-3H3

(–)–*tert*-Butyl 1-benzyl-2-oxospiro[indoline-3,2'-pyrrolidine]-1'-carboxylate ((*S*)–30)

Following general procedure **D** on 0.2 mmol scale using cyclisation precursor **29** and **Cat7** with purification by flash chromatography (40% Et_2O/pentane) afforded *tert*-butyl 1-benzyl-2-oxospiro[indoline-3,2'-pyrrolidine]-1'-carboxylate **(*S*)–30** (73 mg, 96%) as a pale orange solid solid. The characterisation data observed was consistent with the above. The *er* of the product was 17:83 [Chiralpak ID column, 80:20 *n*-hexane:*i*-PrOH, flow rate: 1 mL min^{-1}, 35 ºC, UV detection wavelength: 254 nm, *rac* **30** retention times 10.54 and 15.55 min]; $[\alpha]_D^{22}$ = –8 (*c* = 1.85, Et_2O).

Scale up and derivatisation reactions

One-pot synthesis of (–)–*tert*-butyl 1'-benzyl-2'-oxospiro[azetidine-2,3'-indoline]-1-carboxylate **(*S*)–3** on gram-scale

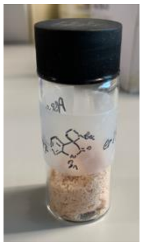

A solution of diazo **1** (1.25 g, 5.0 mmol) in toluene (30 mL) was slowly added over 10 min to a stirred solution of protected amine **S33** (1.80 g, 10.0 mmol) and $Rh_2(esp)_2$ (9.5 mg, 12.5 μmol) in toluene (18 mL) at 25 ºC. The remaining diazo was quantitatively added using toluene (2.0 mL). After 30 min, cat. **394** (592 mg, 1.0 mmol) and $CsOH{\cdot}H_2O$ (4.20 g, 25 mmol) were added and the reaction mixture was stirred for 1 h at 25 ºC. The reaction mixture was filtered through celite, washing with Et_2O (200 mL) and concentrated under reduced pressure. Purification by flash chromatography (40% Et_2O/pentane) afforded *tert*-Butyl 1'-benzyl-2'-oxospiro[azetidine-2,3'-indoline]-1-carboxylate **(*S*)–3** (1.41 g, 77%) as a pale brown solid. The observed characterisation data was consistent with those reported above. The *er* of the product was 3:97 [Chiralpak ID column, 90:10 *n*-hexane:*i*-PrOH, flow rate: 1 mL min^{-1}, 35 ºC, UV detection wavelength: 254 nm, *rac* **3** retention times 18.03 and 27.55 min].

(±)-*tert*-Butyl 2'-oxospiro[azetidine-2,3'-indoline]-1-carboxylate (25)

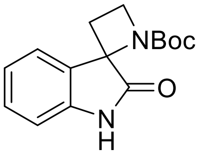

Lithium (3.5 mg, 0.55 mmol) was added to a stirred solution of *tert*-butyl 1'-benzyl-2'-oxospiro[azetidine-2,3'-indoline]-1-carboxylate (20 mg, 0.05 mmol), ethylenediamine (65 µL, 1.1 mmol) in THF (0.15 mL) at 0 ºC. The reaction was stirred at room temperature for 1 h. H_2O (10 mL) was added and the aqueous layer extracted with EtOAc (3 x 20 mL). The combined organic layers were dried over Na_2SO_4, filtered and concentrated under reduced pressure. Purification by column chromatography (70% Et_2O/hexane) affored *tert*-butyl 2'-oxospiro[azetidine-2,3'-indoline]-1-carboxylate (6.8 mg, 45%) as a colourless oil.). R_f 0.13 (60% Et_2O/hexane); ^1H NMR (400 MHz, $CDCl_3$) δ 8.51 (d, *J* = 98.3 Hz, 1 H, NH), 7.47 (d, *J* = 7.4 Hz, 1 H, HC_{Ar}), 7.30- 7.22 (m, 1 H, HC_{Ar}), 7.11 – 7.08 (m, 1 H, HC_{Ar}), 6.90 – 6.85 (m, 1 H, HC_{Ar}), 4.30 – 4.23 (m, 1 H, NC*H*HCH₂), 4.14 – 4.05 (m, 1 H, NCH*H*CH₂), 2.72 – 2.67 (m, 1 H, NCH₂C*H*H), 2.55 – 2.43 (m, 1 H, NCH₂CH*H*), 1.44 and 1.17 (2 × s, 9 H, $C(CH_3)_3$); ^{13}C NMR (101 MHz, $CDCl_3$) δ 177.8 and 177.6 (C=O amide), 155.4 and 154.8 (C=O carbamate), 141.0 (NC_{Ar} quat), 129.9, 129.8, 129.7 and 129.1 (C_{Ar} and C_{Ar} quat), 124.1 and 124.0 (C_{Ar}), 123.1 (C_{Ar}), 110.4 and 109.9 (C_{Ar}), 80.8 and 80.4 (*C*(CH₃)₃), 69.3 and 68.8 (NC quat), 47.5 and 46.1 (N*C*H₂CH₂), 28.5 and 28.0 (C(*C*H₃)₃), 27.5 and 27.4 (NCH₂*C*H₂); HRMS (ESI⁺) *m*/*z* Calculated for $C_{15}H_{18}N_2O_3$ [M+H] 275.1396; Found 275.1392.

SMILES O=C1N([H])C2=CC=CC=C2C13N(CC3)C(OC(C)(C)C)=O
InChI=1S/C15H18N2O3/c1-14(2,3)20-13(19)17-9-8-15(17)10-6-4-5-7-11(10)16-12(15)18/h4-7H,8-9H2,1-3H3,(H,16,18)

(–)–*tert*-Butyl 2'-oxospiro[azetidine-2,3'-indoline]-1-carboxylate ((*S*)–25)

Lithium (2.7 mg, 0.38 mmol) was added to a stirred solution of *tert*-butyl 1'-benzyl-2'-oxospiro[azetidine-2,3'-indoline]-1-carboxylate (15.1 mg, 0.04 mmol), ethylenediamine (50 µL, 0.77 mmol) in THF (0.11 mL) at 0 ºC. The reaction was stirred at room temperature for 1 h. H_2O (10 mL) was added, and the aqueous layer extracted with EtOAc (3 x 20 mL). The combined organic layers were dried over Na_2SO_4, filtered and concentrated under reduced pressure. Purification by column chromatography (70% Et_2O/hexane) affored *tert*-butyl 2'-oxospiro[azetidine-2,3'-indoline]-1-carboxylate (5.4 mg, 52%) as a colourless oil. The characterisation data was consistent with the above. The *er* of the product was 3:97 [Chiralpak IE column, 90:10 n-hexane:*i*-PrOH, flow rate: 1 mL min–1 , 30 ºC, UV detection wavelength: 254 nm, *rac* **25** retention times 24.94 and 30.03 min]; $[\alpha]_D^{22}$ = –5 (*c* = 0.15, CH_2Cl_2).

(±)-1'-Benzylspiro[azetidine-2,3'-indolin]-2'-one (26)

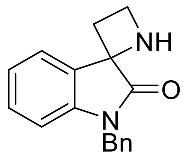

4 N HCl in 1,4-dioxane (2.8 mL, 10.0 mmol) was added to *tert*-butyl 1'-benzyl-2'-oxospiro[azetidine-2,3'-indoline]-1-carboxylate **3** (200 mg, 0.5 mmol). The solution was stirred at 25 ºC for 24 h. 1 M HCl (20 mL) was added, and the aqueous mixture extracted with EtOAc (2 × 20 mL). The aqueous layer was basified with sat. aq. $NaHCO_3$ (40 mL) and extracted with EtOAc (3 × 25 mL). The combined organic layers were dried over Na_2SO_4, filtered and concentrated under reduced pressure. Purification by column chromatography (2-5% MeOH/CH_2Cl_2) afforded 1'-benzylspiro[azetidine-2,3'-indolin]-2'-one **26** (115 mg, 79%) as a white solid. R_f 0.27 (3% MeOH/CH_2Cl_2);); mp = 55–56 ºC; ν_{max} (film)/cm^{-1} 2959, 2857, 1700 (C=O amide), 1616, 1591, 1488, 1461, 1301, 1176, 1104, 984, 852, 752; 1H NMR (400 MHz, DMSO-*d*$_6$) δ 7.73 (br s, 1 H, NH), 7.49 (d, *J* = 7.4 Hz, 1 H, HC_{Ar}), 7.39 – 7.23 (m, 6 H, 5 × HC_{Ph} + 1 × HC_{Ar}), 7.11 (t, *J* = 7.5 Hz, 1 H, HC_{Ar}), 6.99 (d, *J* = 7.8 Hz, 1 H, HC_{Ar}), 4.91 (d, *J* = 2.5 Hz, 2 H, NCH_2Ph), 3.82 – 3.63 (m, 1 H, NC*H*HCH_2), 3.34 – 3,29 (m, 1 H, NCH*H*CH_2), 2.33 – 2.26 (m, 1 H, NCH_2C*H*H), 2.17 – 2.11 (m, 1 H, NCH_2CH*H*); ^{13}C NMR (101 MHz, DMSO-*d*$_6$) δ 173.8 (C=O amide), 151.8 (C_{Ar} quat), 142.3 (C_{Ar} quat), 136.3 (C_{Ph} quat), 131.0 (C_{Ar}), 129.2 (C_{Ph}), 128.0 (C_{Ph}), 127.7 (C_{Ph}), 127.6 (2 × C_{Ph}), 124.8 (C_{Ar}), 123.7 (C_{Ar}), 110.4 (C_{Ar}), 79.2 (NC quat), 43.2 (NCH_2Ph), 35.4 (N*C*H_2CH_2), 26.9 and 26.0 (NCH_2*C*H_2); HRMS (ESI$^+$) *m*/*z* Calculated for $C_{17}H_{17}N_2O$ $[M+H]^+$ 265.1263; Found 265.1257.

SMILES O=C1N(CC2=CC=CC=C2)C3=CC=CC=C3C14NCC4
InChI=1S/C17H16N2O/c20-16-17(10-11-18-17)14-8-4-5-9-15(14)19(16)12-13-6-2-1-3-7-13/h1-9,18H,10-12H2

(–)–1'-Benzylspiro[azetidine-2,3'-indolin]-2'-one ((*S*)–26)

4 N HCl in 1,4-dioxane (0.7 mL, 2.5 mmol) was added to *tert*-butyl 1'-benzyl-2'-oxospiro[azetidine-2,3'-indoline]-1-carboxylate **(*S*)–3a** (50 mg, 0.13 mmol). The solution was stirred at 25 ºC for 24 h. 1 M HCl (5 mL) was added, and the aqueous mixture extracted with EtOAc (2 x 10 mL). The aqueous layer was basified with sat. aq. $NaHCO_3$ (20 mL) and extracted with EtOAc (3 x 10 mL). The combined organic layers were dried over Na_2SO_4, filtered and concentrated under reduced pressure. Purification by column chromatography (2-5% MeOH/CH2Cl2) afforded 1'-benzylspiro[azetidine-2,3'-indolin]-2'-one **(*S*)–26** (33 mg, 89%) as a white solid. The characterisation data was consistent with the above. The *er* of the product was 3:97 [Chiralpak IE column, 90:10 n-hexane:*i*-PrOH, flow rate: 1 mL min–1 , 30 ºC, UV detection wavelength: 254 nm, *rac* **26** retention times 28.84 and 32.72 min]; $[\alpha]_D^{22} = –12$ (*c* = 0.45, CH_2Cl_2).

(±)-1-Benzylspiro[indoline-3,6'-[1,3]oxazinane]-2,2'-dione (27)

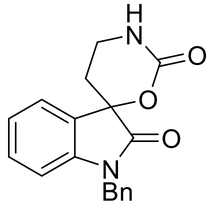

Trifluoroacetic acid (61 µL, 0.81 mmol) was added to a stirred solution of *tert*-butyl 1'-benzyl-2'- oxospiro[azetidine-2,3'-indoline]-1-carboxylate **3** (58.7 mg, 0.16 mmol) in CH_2Cl_2 (0.16 mL). The reaction mixture was stirred at 25 ºC for 30 min. The black solution was concentrated under reduced pressure and purified by column chromatography (2% MeOH/CH_2Cl_2) to afford 1-benzylspiro[indoline-3,6'- [1,3]oxazinane]-2,2'-dione **27** (38 mg, 76%) as a white solid. R_f 0.23 (2% MeOH/CH_2Cl_2); mp = 105–106; ºC νmax (film)/cm^{-1} 3239 (N-H), 2920, 2851, 1701 (C=O carbamate), 1666 (C=O amide), 1610, 1467, 1372, 1319, 1202, 1172, 1089, 749, 723, 694; ^1H NMR (400 MHz, DMSO-*d*$_6$) δ 7.73 (s, 1 H, NH), 7.49 (d, *J* = 7.4 Hz, 1 H, HC_{Ar}), 7.39 – 7.23 (m, 6 H, 6 × HC_{Ar}), 7.11 (t, *J* = 7.5 Hz, 1 H, HC_{Ar}), 6.99 (d, *J* = 7.8 Hz, 1 H, HC_{Ar}), 4.91 (d, *J* = 2.8 Hz, 2 H, NCH_2Ph), 3.72 – 3.65 (m, 1 H, NHC*H*HCH_2), 3.36 – 3.29 (m, 1 H, NHCH*H*CH_2), 2.36 – 2.24 (m, 1 H, $NHCH_2$C*H*H), 2.17 – 2.10 (m, 1 H, $NHCH_2$CH*H*); ^{13}C NMR (101 MHz, DMSO-*d*$_6$) δ 173.4 (C=O amide), 151.4 (C=O carbamate), 141.9 (C_{Ar} quat), 135.8 (C_{Ar} quat), 130.6 (C_{Ar} quat), 128.8, 127.6, 127.2, 124.4 and 123.2 (5 × C_{Ar}), 109.9 (C_{Ar}), 78.7 (OC quat), 42.7 (NCH_2Ph), 34.9 (NH*C*H_2CH_2), 26.4 ($NHCH_2$*C*H_2); HRMS (ESI$^+$) *m*/*z* Calculated for $C_{18}H_{17}N_2O_3$ [M+H] 309.1239; Found 309.1244.

SMILES O=C1N(CC2=CC=CC=C2)C3=CC=CC=C3C14OC(NCC4)=O
InChI=1S/C18H16N2O3/c21-16-18(10-11-19-17(22)23-18)14-8-4-5-9-15(14)20(16)12-13-6-2-1-3-7-13/h1-9H,10-12H2,(H,19,22)

(–)–1-Benzylspiro[indoline-3,6'-[1,3]oxazinane]-2,2'-dione ((*S*)–27)

Trifluoroacetic acid (41 μL, 0.54 mmol) was added to a stirred solution of tert-butyl 1'-benzyl-2'-oxospiro[azetidine-2,3'-indoline]-1-carboxylate **(*S*)–3a** (39.4 mg, 0.11 mmol) in CH2Cl2 (0.11 mL). The reaction mixture was stirred at 25 ºC for 30 min. The black solution was concentrated under reduced pressure and purified by column chromatography (2% MeOH/CH2Cl2) to afford 1-benzylspiro[indoline-3,6'-[1,3]oxazinane]-2,2'-dione **(*S*)–27** (21 mg, 66%) as a white solid. The characterisation data was consistent with the above. The er of the product was 25:75 [Chiralpak IF column, 90:10 n-hexane:i-PrOH, flow rate: 1 mL min–1 , 30 ºC, UV detection wavelength: 254 nm, *rac* **27** retention times 11.14 and 13.68]; $[\alpha]_D^{22}$ = –4 (*c* = 0.5, CH_2Cl_2).

(±)-*tert*-Butyl 1'-benzyl-2'-oxo-6'-(m-tolyl)spiro[azetidine-2,3'-indoline]-1-carboxylate (28)

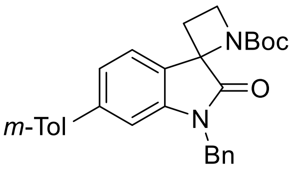

A solution of *tert*-butyl 1'-benzyl-6'-bromo-2'-oxospiro[azetidine-2,3'-indoline]-1-carboxylate **11** (27.1 mg, 0.06 mmol), *m*-tolylboronic acid (16.6 mg, 0.12 mmol), $PdCl_2(dppf)_2$ (2.2 mg, 2.2 μmol) and K_2CO_3 (50 mg, 0.37 mmol) in 1,4 dioxane (0.45 mL) and H_2O (90 μL) was stirred at 90 ºC for 18 h. The reaction mixture was cooled to rt and filtered through Celite washing with EtOAc (10 mL). The filtrate was concentrated under reduced pressure and purified by column chromatography (30% Et_2O/hexane) to give *tert*-butyl 1'-benzyl-2'-oxo-6'-(m-tolyl)spiro[azetidine-2,3'-indoline]-1-carboxylate **28** (22.4 mg, 80%) as a white solid. R_f 0.34 (30% Et_2O/hexane); mp = 157–158 ºC; νmax (film)/cm^{-1} 2966, 2922, 1723 (C=O amide), 1698 (C=O carbamate), 1618, 1479, 1361, 1145, 965, 781, 697; ^{1}H NMR (400 MHz, $CDCl_3$) δ 7.51 (s, 1 H, HC_{Ar}), 7.45 – 7.39 (m, 2 H, 2 × HC_{Ar}), 7.37 – 7.13 (m, 8 H, 8 × HC_{Ar}), 6.95 (d, *J* = 1.5 Hz, 1 H, HC_{Ar}), 5.17 – 4.72 (m, 2 H, NCH_2Ar), 4.42 – 4.25 (m, 1 H, NC*H*HCH_2), 4.20 – 4.03 (m, 1 H, NCH*H*CH_2), 2.82 – 2.60 (m, 1 H, NCH_2C*H*H), 2.57 – 2.55 (m, 1 H, NCH_2CH*H*), 2.42 (d, *J* = 15.4 Hz, 3 H, $ArCH_3$), 1.48 and 1.08 (2 × br s, 9 H, $C(CH_3)_3$); ^{13}C NMR (101 MHz, $CDCl_3$) δ 176.0 and 175.7 (C=O amide), 155.6 and 154.7 (C=O carbamate), 143.5 and 143.3 (C_{Ar} quat), 141.2 and 140.9 (C_{Ar} quat), 138.7 (C_{Ar} quat), 135.8 (C_{Ar} quat), 135.7 (C_{Ar} quat), 129.0 and 128.9 (2 × C_{Ar}), 128.8 and 128.7 (C_{Ar}), 128.4 (C_{Ar}), 128.2 (C_{Ar}), 128.0 and 127.9 (C_{Ar}), 127.6 (C_{Ar}), 127.4 (C_{Ar}), 124.5 and 124.3 (C_{Ar} quat), 124.0 and 123.8 (C_{Ar}), 122.3 and 122.1 (C_{Ar}), 108.5 (C_{Ar}), 107.8 (C_{Ar}), 80.8 and 80.3 (*C*$(CH_3)_3$), 68.7 (NC quat), 47.5 and 46.0 (NCH_2), 44.2 and 44.1 (NCH_2Ar) 28.5 (NCH_2*C*H_2), 28.0 and 27.8 (C(*C*$H_3)_3$), 22.8 ($ArCH_3$); HRMS (ESI$^+$) *m/z* Calculated for $C_{29}H_{31}N_2O_3$ [M+H] 455.2335; Found 455.2321.

SMILES O=C1N(CC2=CC=CC=C2)C3=CC(C4=CC(C)=CC=C4)=CC=C3C15N(CC5)C(OC(C)(C)C)=O
InChI=1S/C29H30N2O3/c1-20-9-8-12-22(17-20)23-13-14-24-25(18-23)30(19-21-10-6-5-7-11-21)26(32)29(24)15-16-31(29)27(33)34-28(2,3)4/h5-14,17-18H,15-16,19H2,1-4H3

(–)–*tert*-Butyl 1'-benzyl-2'-oxo-6'-(m-tolyl)spiro[azetidine-2,3'-indoline]-1-carboxylate ((*S*)–29)

A solution of *tert*-butyl 1'-benzyl-6'-bromo-2'-oxospiro[azetidine-2,3'-indoline]-1-carboxylate **(*S*)-11** (18.3 mg, 0.04 mmol), *m*-tolylboronic acid (11.2 mg, 0.08 mmol), $PdCl_2(dppf)_2$ (1.5 mg, 1.5 μmol) and K_2CO_3 (34 mg, 0.25 mmol) in 1,4 dioxane (0.3 mL) and H_2O (60 μL) was stirred at 90 ºC for 18 h. The reaction mixture was cooled to rt and filtered through Celite washing with EtOAc (10 mL). The filtrate was concentrated under reduced pressure and purified by column chromatography (30% Et_2O/hexane) to give (-)-*tert*-butyl 1'-benzyl-2'-oxo-6'-(m-tolyl)spiro[azetidine-2,3'-indoline]-1-carboxylate **(*S*)-29** (13.8 mg, 73%) as a white solid. The characterisation data was consistent with the above. The er of the product was 2:98 [Chiralpak ID column, 90:10 n-hexane:*i*-PrOH, flow rate: 1 mL min–1 , 30 ºC, UV detection wavelength: 254 nm, *rac* **29** retention times 15.46 and 22.28] $[\alpha]_D^{22}$ = –28 (*c* = 0.25, Et_2O).

Catalysts

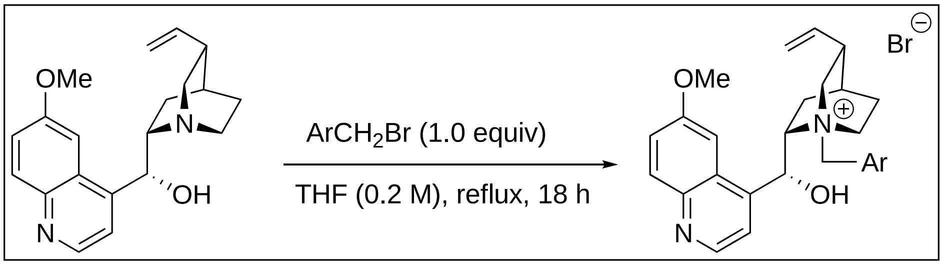

A solution of quinine (162 mg, 0.5 mmol) and alkyl halide (1 equiv) in THF (0.2 M) was heated to 70 ºC overnight. The reaction was cooled to rt and Et_2O was added. The reaction was stirred for approx. 0.5 h then the precipitate was filtered to afford the alkylated product.

(1*S*,2*S*,4*S*,5*R*)-2-((*R*)-Hydroxy(6-methoxyquinolin-4-yl)methyl)-1-(4-(trifluoromethyl)benzyl)-5-vinylquinuclidin-1-ium bromide (QN4) (Cat1)

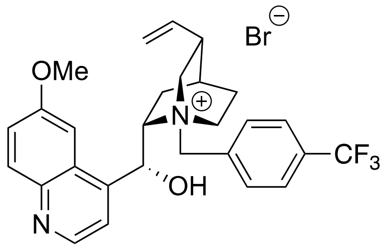

A solution of quinine (324 mg, 1.0 mmol) and 4-(trifluromethyl)benzyl bromide (239 mg, 1.0 mmol) in THF (5.0 mL) was heated to 70 ºC for 22 h. The reaction was cooled to rt and Et_2O was added. The reaction was stirred for approx. 0.5 h then the precipitate was filtered. The collected solid was dried to afford (1*S*,2*S*,4*S*,5*R*)-2-((*R*)-hydroxy(6-methoxyquinolin-4-yl)methyl)-1-(4-(trifluoromethyl)benzyl)-5-vinylquinuclidin-1-ium bromide **QN4** (505 mg, 90%) as a white solid. $[\alpha]_D^{17}$ = –180 (*c* = 0.1, MeOH) [lit = –153 (c = 0.18, MeOH)][24]; R_f 0.38 (5% MeOH/CH_2Cl_2); mp = >225 ºC [lit = 185–187 ºC][24]; ν_{max} (film)/cm^{-1} 3171 (OH), 1619, 1508, 1429, 1327, 1224, 1163, 1122, 1030, 1022, 856, 826; ^{1}H NMR (400 MHz, DMSO-*d*$_6$) δ 8.82 (d, *J* = 4.5 Hz, 1 H, HC_{Ar}), 8.03 (d, *J* = 9.2 Hz, 1 H, HC_{Ar}), 7.99–7.90 (m, 4 H, 4 × HC_{Ar}), 7.76 (d, *J* = 4.5 Hz, 1 H, HC_{Ar}), 7.51 (dd, *J* = 9.2, 2.6 Hz, 1 H, HC_{Ar}), 7.38 (d, *J* = 2.6 Hz, 1 H, HC_{Ar}), 6.72 (d, *J* = 3.7 Hz, 1 H, OH), 6.59–6.55 (m, 1 H, N^+CH), 5.74 (ddd, *J* = 17.3, 10.4, 6.9 Hz, 1 H, C*H*=CH_2), 5.49 (d, *J* = 12.4 Hz, 1 H, N^+C*H*HAr), 5.14–5.07 (m, 1 H, CH=C*H*H), 5.03–4.98 (m, 1 H, CH=CH*H*), 4.79 (d, *J* = 12.3 Hz, 1 H, N^+CH*H*Ar), 4.28–4.23 (m, 1 H, N^+C*H*H), 4.00 (s, 3 H, OCH_3), 3.89–3.81 (m, 1 H, C*H*OH), 3.70 (dd, *J* = 12.4, 5.7 Hz, 1 H, N^+C*H*H), 3.39–3.30 (m, 1 H, N^+CH*H*, confirmed by HSQC), 3.29–3.19 (m, 1 H, N^+CH*H*), 2.72–2.62 (m, 1 H, CH_2=CHC*H*), 2.29–2.20 (m, 1 H, NCH_2C*H*H), 2.19–2.08 (m, 1 H, NCHC*H*H), 2.03–1.97 (m, 1 H, $N(CH_2)_2$C*H*), 1.87–1.76 (m, 1H, NCHCH*H*), 1.50–1.40 (m, 1 H, NCH_2CH*H*); ^{1}H NMR (400 MHz, $CDCl_3$) δ 8.67 (d, *J* = 4.5 Hz, 1 H, HC_{Ar}), 7.98 (d, *J* = 8.1 Hz, 2 H, 2 × HC_{Ar}), 7.94 (d, *J* = 9.0 Hz, 1 H, HC_{Ar}), 7.70 (d, *J* = 4.5 Hz, 1 H, HC_{Ar}), 7.56 (d, *J* = 8.1 Hz, 2 H, 2 × HC_{Ar}), 7.29–7.23 (m, 2 H, 2 × HC_{Ar}), 6.62 (d, *J* = 6.6 Hz, 1 H, N^+CH), 6.38 (d, *J* = 6.7 Hz, 1 H, OH), 6.09 (d, *J* = 12.0 Hz, 1 H, N^+CH*H*Ar), 5.55 (ddd, *J* = 17.2, 10.4, 6.7 Hz, 1 H, C*H*=CH_2), 5.18–5.07 (m, 2 H, N^+CH*H*Ar and CH=C*H*H), 4.96–4.82 (m, 2 H CH=CH*H* and N^+C*H*H), 4.06–3.88 (m, 4 H, OCH_3 and C*H*OH), 3.81–3.71 (m, 1 H, N^+C*H*H), 3.40–3.30 (m, 1 H, N^+CH*H*), 3.03 (td, *J* = 11.5, 6.1 Hz, 1 H, N^+CH*H*), 2.54 (d, *J* = 7.3 Hz, 1 H, CH_2=CHC*H*), 2.32–2.16 (m, 2 H, NCH_2C*H*H and NCHC*H*H), 2.04–1.96 (m, 1 H, $N(CH_2)_2$C*H*), 1.77–1.67 (m, 1 H, NCHCH*H*), 1.54–1.44 (m, 1 H, NCH_2CH*H*); ^{13}C NMR (101 MHz, DMSO-*d*$_6$) δ 157.4 (OC_{Ar} quat), 147.4 (C_{Ar}), 143.8 (C_{Ar} quat), 143.7 (C_{Ar} quat), 138.0 (CH_2=*C*H), 134.5 (2 × C_{Ar}), 132.6 (C_{Ar} quat), 131.5 (C_{Ar}), 130.4 (q, J_{C-F} = 32 Hz, $C_{Ar}CF_3$ quat), 125.8 (2 × C_{Ar}), 125.3 (C_{Ar} quat), 124.0 (q, *J* = 272 Hz, CF_3), 121.4 (C_{Ar}), 120.3 (C_{Ar}) 116.6 (*C*H_2=CH), 102.1 (C_{Ar}), 68.6 (CHOH), 63.6 (N^+CH), 62.3 (N^+CH_2Ar), 59.2 (N^+CH_2), 56.0 (OCH_3), 50.8 (N^+CH_2), 37.0 (CH_2=CH*C*H), 25.9 ($N(CH_2)_2$*C*H), 24.2 (NCH*C*H_2), 20.3 (NCH_2*C*H_2); ^{13}C NMR (101 MHz, $CDCl_3$) δ 158.0 (OC_{Ar} quat), 147.3 (C_{Ar}), 144.1 (C_{Ar} quat), 142.8 (C_{Ar} quat), 135.9 (*C*H=CH_2), 134.4 (2 × C_{Ar}), 132.3 (q, J_{C-F} = 33 Hz, CF_3C_{Ar} quat), 131.7 (C_{Ar} quat), 131.2 (C_{Ar} quat), 125.9 (C_{Ar}), 125.7 (2 × C_{Ar}), 123.4 (q, J_{C-F} = 273 Hz, CF_3), 121.2 (C_{Ar}), 120.5 (C_{Ar}), 117.8 (CH=*C*H_2), 101.7 (C_{Ar}), 69.1 (CHOH), 63.9 (N^+CH), 61.6 (N^+CH_2Ar), 60.2 (N^+CH_2), 56.3 (OCH_3), 51.4 (N^+CH_2), 37.9 (*C*HCH=CH_2), 26.6 ($N(CH_2)_2$*C*H), 24.8 (NCH_2*C*H_2), 21.6 (NCH_2*C*H_2); ^{19}F NMR (377 MHz, DMSO-*d*$_6$) δ –61.3; ^{19}F{^{1}H} NMR (377 MHz, $CDCl_3$) δ –63.1. The observed characterisation data (^{1}H in DMSO-*d*$_6$) was consistent with that previously reported in the literature.[25]

SMILES:
C=C[C@H]1C[N@@+]2(CC3=CC=C(C(F)(F)F)C=C3)[C@H]([C@@H](C4=C(C=C(OC)C=C5)C5=NC=C4)O)C[C@@H]1CC2.[Br-]
InChI=1S/C28H30F3N2O2.BrH/c1-3-19-17-33(16-18-4-6-21(7-5-18)28(29,30)31)13-11-20(19)14-26(33)27(34)23-10-12-32-25-9-8-22(35-2)15-24(23)25;/h3-10,12,15,19-20,26-27,34H,1,11,13-14,16-17H2,2H3;1H/q+1;/p-1/t19-,20-,26-,27+,33+;/m0./s1

(1*S*,2*S*,4*S*,5*R*)-2-((*R*)-Methoxy(6-methoxyquinolin-4-yl)methyl)-5-vinylquinuclidine (S60)

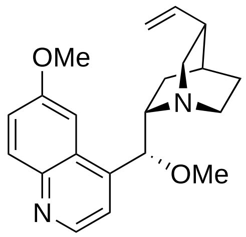

NaH (60% dispersion in mineral oil, 1.0 g, 25.0 mmol) was added portionwise to a solution of quinine (3.24 g, 10.0 mmol) in DMF (33 mL) at 0 ºC and stirred for 1 h at 0 ºC. MeI (0.62 mL, 10.0 mmol) was added dropwise at 0 ºC and the reaction mixture was stirred for 24 h at 25 ºC. Sat. aq. brine (50 mL) was added and the aqueous reaction mixture was exracted with EtOAc (3 × 50 mL). The combined organic layers were dried over Na_2SO_4, filtered and concentrated under reduced pressure. Purification by flash chromatography afforded (1*S*,2*S*,4*S*,5*R*)-2-((*R*)-methoxy(6-methoxyquinolin-4-yl)methyl)-5-vinylquinuclidine **S60** (1.63 g, 48%) as a yellow gum. $[\alpha]_D^{17}$ = –144º (*c* = 1.17, $CHCl_3$); R_f 0.18 (10% MeOH/CH_2Cl_2); ν_{max} (film)/cm^{-1} 2933, 2866, 1618, 1506, 1472, 1226, 1111, 1059, 1025, 913, 857, 716; ^{1}H NMR (400 MHz, $CDCl_3$) δ 8.77 (d, *J* = 4.5 Hz, 1 H, HC_{Ar}), 8.05 (d, *J* = 9.5 Hz, 1 H, HC_{Ar}), 7.45–7.35 (m, 3 H, 3 × HC_{Ar}), 5.70 (ddd, *J* = 17.5, 10.3, 7.6 Hz, 1 H, C*H*=CH_2), 5.14 (br s, 1 H, C*H*OCH_3), 5.01–4.86 (m, 2 H, CH=C*H_2*), 3.98 (s, 3 H, ArOCH_3), 3.51 (s, 1 H, NC*H*H), 3.34 (s, 3 H, OCH_3), 3.22–3.09 (m, 2 H, NC*H*H and NCH), 2.82–2.65 (m, 2 H, 2 × NCH*H*), 2.34 (s, 1 H, C*H*CH=CH_2), 1.85 (s, 3 H, NCHC*H*H, NCH_2C*H*H and N$(CH_2)_2$C*H*), 1.63–1.47 (m, 2 H, NCHCH*H* and NCH_2C*H*H); ^{13}C NMR (101 MHz, $CDCl_3$) δ 158.0 (OC_{Ar} quat), 147.3 (C_{Ar}), 144.5 (C_{Ar} quat), 143.9 (C_{Ar} quat), 141.1 (CH_2=CH), 131.7 (C_{Ar}), 127.3 (C_{Ar} quat), 121.8 (C_{Ar}), 118.5 (*C*H_2=CH), 114.7 (C_{Ar}), 101.1 (C_{Ar}), 82.0 (*C*HOCH_3), 59.8 (NCH), 57.0 (OCH_3), 56.6 (NCH_2), 56.0 (ArOCH_3), 43.2 (NCH_2), 39.5 (*C*HCH=CH_2), 27.7 (N$(CH_2)_2$*C*H), 27.1 ($NCHCH_2$), 21.4 (NCH_2CH_2). The observed characterisation data (IR, ^{1}H and ^{13}C NMR) was consistent with that previously reported in the literature.[26]

SMILES: C=C[C@H]1C[N@]2[C@H]([C@@H](C3=C(C=C(OC)C=C4)C4=NC=C3)OC)C[C@@H]1CC2
InChI=1S/C21H26N2O2/c1-4-14-13-23-10-8-15(14)11-20(23)21(25-3)17-7-9-22-19-6-5-16(24-2)12-18(17)19/h4-7,9,12,14-15,20-21H,1,8,10-11,13H2,2-3H3/t14-,15-,20-,21?/m0/s1

(1*S*,2*S*,4*S*,5*R*)-2-((*R*)-Methoxy(6-methoxyquinolin-4-yl)methyl)-1-(4-(trifluoromethyl)benzyl)-5-vinylquinuclidin-1-ium bromide (QN4B) (Cat2)

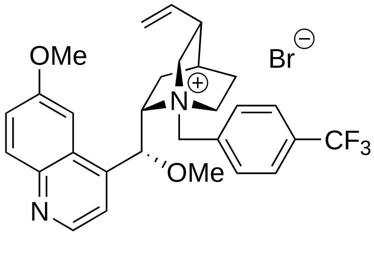

A solution of (1*S*,2*S*,4*S*,5*R*)-2-((*R*)-Methoxy(6-methoxyquinolin-4-yl)methyl)-5-vinylquinuclidine **S62** (338 mg, 1.0 mmol) and 4-(triflurometyl)benzyl bromide (239 mg, 1.0 mmol) in THF (5.0 mL) was heated to 70 ºC for 18 h. The reaction was cooled to rt and Et_2O was added. The reaction was stirred for approx. 0.5 h then the precipitate was filtered. The collected solid was purified by flash chromatography (10% MeOH/CH_2Cl_2) afforded (1*S*,2*S*,4*S*,5*R*)-2-((*R*)-methoxy(6-methoxyquinolin-4-yl)methyl)-1-(4-(trifluoromethyl)benzyl)-5-vinylquinuclidin-1-ium bromide **QN4B** (287 mg, 50%) as a pale yellow solid. $[\alpha]_D^{17}$ = –216º (*c* = 0.5, CH_3OH); R_f 0.11 (10% MeOH/CH_2Cl_2); ν_{max} (film)/cm^{-1} 2952, 1621, 1506, 1323, 1238, 1170, 1126, 1070, 1021, 910, 857, 723; ^{1}H NMR (400 MHz, CD_3OD) δ 8.85 (d, *J* = 4.5 Hz, 1 H, HC_{Ar}), 8.09 (d, *J* = 9.3 Hz, 1 H, HC_{Ar}), 8.02 (d, *J* = 8.1 Hz, 2 H, 2 × HC_{Ar}), 7.94 (d, *J* = 8.1 Hz, 2 H, 2 × HC_{Ar}), 7.83 (d, *J* = 4.5 Hz, 1 H, HC_{Ar}), 7.58 (dd, *J* = 9.3, 2.6 Hz, 1 H, HC_{Ar}), 7.49 (d, *J* = 2.4 Hz, 1 H, HC_{Ar}), 6.39 (s, 1 H, N^+CH), 5.77 (ddd, *J* = 17.3, 10.4, 7.0 Hz, 1 H, CH_2=C*H*), 5.53 (d, *J* = 12.3 Hz, 1 H, N^+C*H*HAr), 5.18 (dt, *J* = 17.2, 1.1 Hz, 1 H, C*H*H=CH), 5.08 (dt, *J* = 10.5, 1.1 Hz, 1 H, CH*H*=CH), 4.92 (d, *J* = 12.3 Hz, 1 H, N^+CH*H*Ar), 4.30–4.20 (m, 1 H, N^+C*H*H), 4.11 (s, 3 H, ArOCH_3), 4.02–3.95 (m, 1 H, C*H*OCH_3), 3.74–3.64 (m, 4 H, OCH_3 and N^+C*H*H), 3.58–3.42 (m, 2 H, 2 × N^+CH*H*), 2.80–2.71 (m, 1 H, CH_2=CHC*H*), 2.56–2.48 (m, 1 H, NCHC*H*H),

2.40–2.29 (m, 1 H, NCH_2CHH), 2.16–2.11 (m, 1 H, $N(CH_2)_2CH$), 2.02–1.91 (m, 1 H, NCH_2CHH), 1.73 – 1.63 (m, 1 H, NCHCH*H*); ^{13}C NMR (101 MHz, CD_3OD) δ 160.3 (OC_{Ar} quat), 148.2 (C_{Ar}), 145.3 (C_{Ar} quat), 141.5 (C_{Ar} quat), 138.4 (CH_2=*C*H), 135.8 (2 × C_{Ar}), 133.6 (q, $J_{C–F}$ = 33 Hz, $C_{Ar}CF_3$ quat), 132.9 (C_{Ar} quat), 132.0 (C_{Ar}), 128.3 (C_{Ar} quat), 127.2 (q, $J_{C–F}$ = 4 Hz, 2 × C_{Ar}), 125.3 (q, $J_{C–F}$ = 272 Hz, CF_3), 123.6 (C_{Ar}), 121.6 (C_{Ar}), 117.8 (*C*H_2=CH), 102.5 (C_{Ar}), 75.6 (N^+CH), 70.7 (*C*$HOCH_3$), 65.2 (NCH_2), 61.8 (N^+CH_2), 57.3 (OCH_3), 56.7 ($ArOCH_3$), 53.1 (N^+CH_2), 39.1 (CH_2=CH*C*H), 28.1 ($N(CH_2)_2CH$), 25.8 (NCH_2CH_2), 22.2 ($NCHCH_2$); ^{19}F NMR (377 MHz, CD_3OD) δ –64.5; HRMS (ESI$^+$) *m/z* Calculated for $C_{29}H_{32}N_2O_2F_3^+$ $[M+H–Br]^+$ 497.2410; Found 497.2395.

SMILES:
C=C[C@H]1C[N@@+]2(CC3=CC=C(C(F)(F)F)C=C3)[C@H]([C@@H](C4=C(C=C(OC)C=C5)C5=NC=C4)OC)C[C@@H]1CC2.[Br-]
InChI=1S/C29H32F3N2O2.BrH/c1-4-20-18-34(17-19-5-7-22(8-6-19)29(30,31)32)14-12-21(20)15-27(34)28(36-3)24-11-13-33-26-10-9-23(35-2)16-25(24)26;/h4-11,13,16,20-21,27-28H,1,12,14-15,17-18H2,2-3H3;1H/q+1;/p-1/t20-,21-,27-,28?,34+;/m0./s1

(1*S*,2*S*,4*S*,5*R*)-2-((*R*)-(Benzyloxy)(6-methoxyquinolin-4-yl)methyl)-5-vinylquinuclidine (S61)

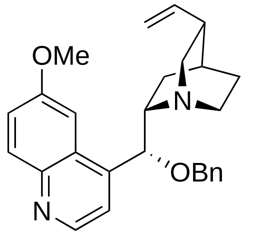

NaH (60% dispersion in mineral oil, 1.20 g, 30.0 mmol) was added portionwise to quinine (3.24 g, 10.0 mmol) in DMF (33.3 mL) at 25 ºC and stirred for 2 h. BnCl (1.27 mL, 11.0 mL) was added dropwise over 10 min and then stirred at 25 ºC for 17 h. Sat. aq. NaCl (50 mL) was added slowly, then H_2O (100 mL) was added followed by EtOAc (100 mL) and the layers were separated. The aqueous layer was extracted with EtOAc (2 × 50 mL). The combined organic layers were washed with H_2O (3 × 50 mL), dried over Na_2SO_4, filtered and concentrated under reduced pressure. Purification by flash chromatography (5% to 10% MeOH/CH_2Cl_2) afforded (1*S*,2*S*,4*S*,5*R*)-2-((*R*)-(benzyloxy)(6-methoxyquinolin-4-yl)methyl)-5-vinylquinuclidine **S61** (3.62 g, 87%) as a pale yellow gum. $[\alpha]_D^{14}$ = –57º (*c* = 1, CH_2Cl_2); R_f 0.60 (10% MeOH/CH_2Cl_2); ν_{max} (film)/cm^{-1} 2937, 2863, 1618, 1506, 1454, 1238, 1103, 1029, 857, 734, 697; ^{1}H NMR (400 MHz, $CDCl_3$) δ 8.77 (d, *J* = 4.4 Hz, 1 H, HC_{Ar}), 8.07 (d, *J* = 9.3 Hz, 1 H, HC_{Ar}), 7.49 (d, *J* = 4.5 Hz, 1 H, HC_{Ar}), 7.44 – 7.29 (m, 8 H, 2 × HC_{Ar} and 5 × HC_{Ph}), 5.74 (ddd, *J* = 17.6, 10.3, 7.7 Hz, 1 H, CH_2=C*H*), 5.27 (br s, 1 H, C*H*OBn), 5.01–4.87 (m, 2 H, C*H*$_2$=CH), 4.45 (q, *J* = 11.4 Hz, 2 H, OC*H*$_2$Ph), 3.93 (s, 3 H, OCH_3), 3.50–3.34 (m, 1 H, NC*H*H), 3.22–3.05 (m, 2 H, NC*H*H and NCH), 2.77–2.59 (m, 2 H, 2 × NCH*H*), 2.33–2.23 (s, 1 H, CH_2=CHC*H*), 1.90–1.46 (m, 5 H, NCHC*H*$_2$, NCH_2CH_2 and $N(CH_2)_2CH$); ^{13}C NMR (101 MHz, $CDCl_3$) δ 157.8 (OC_{Ar} quat), 147.6 (C_{Ar}), 144.7 (C_{Ar} quat), 144.5 (C_{Ar} quat), 141.8 (CH_2=*C*H), 137.8 (C_{Ar}), 131.8 (C_{Ar}), 128.4 (2 × C_{Ar}), 127.7 (C_{Ar}), 127.6 (2 × C_{Ar}), 127.4 (C_{Ar} quat), 121.8 (C_{Ar}), 119.0 (C_{Ar}), 114.3 (*C*H_2=CH), 101.3 (C_{Ar}), 80.9 (CHOBn), 71.1 (OCH_2Ph), 60.3 (NCH), 57.0 (NCH_2), 55.7 (OCH_3), 43.2 (NCH_2), 39.9 (CH_2=CH*C*H), 27.8 ($N(CH_2)_2CH$), 27.7 (NCH_2CH_2), 22.6 ($NCHCH_2$); HRMS (ESI$^+$) *m/z* Calculated for $C_{27}H_{31}N_2O_2$ [M+H] 415.2386; Found 415.2386. The observed characterisation data (^{1}H and ^{13}C NMR) was consistent with that previously reported in the literature.[27]

SMILES:
C=C[C@H]1C[N@]2[C@H]([C@@H](C3=C(C=C(OC)C=C4)C4=NC=C3)OCC5=CC=CC=C5)C[C@@H]1C
C2
InChI=1S/C27H30N2O2/c1-3-20-17-29-14-12-21(20)15-26(29)27(31-18-19-7-5-4-6-8-19)23-11-13-28-25-10-9-22(30-2)16-24(23)25/h3-11,13,16,20-21,26-27H,1,12,14-15,17-18H2,2H3/t20-,21-,26-,27?/m0/s1

(1*S*,2*S*,4*S*,5*R*)-2-((*R*)-(Benzyloxy)(6-methoxyquinolin-4-yl)methyl)-1-(4-(trifluoromethyl)benzyl)-5-vinylquinuclidin-1-ium bromide (QN4C)

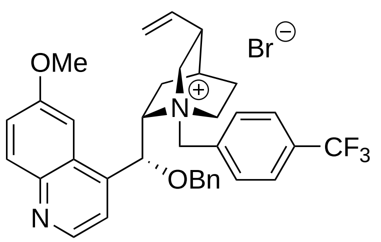

A solution of (1*S*,2*S*,4*S*,5*R*)-2-((*R*)-(benzyloxy)(6-methoxyquinolin-4-yl)methyl)-5-vinylquinuclidine **S61** (207 mg, 0.5 mmol) and 4-(trifluromethyl)benzyl bromide (120 mg, 1.0 mmol) in THF (2.5 mL) was heated to 70 ºC for 18 h. The reaction was cooled to rt and Et_2O was added. The reaction was stirred for approx. 0.5 h then the precipitate was filtered. The collected solid was purified by flash chromatography (5% MeOH/CH_2Cl_2) afforded (1*S*,2*S*,4*S*,5*R*)-2-((*R*)-(benzyloxy)(6-methoxyquinolin-4-yl)methyl)-1-(4-(trifluoromethyl)benzyl)-5-vinylquinuclidin-1-ium bromide **QN4C** (189 mg, 58%) as a pale white solid. $[\alpha]_D^{17}$ = –120º (*c* = 0.5, $CHCl_3$); R_f 0.35 (10% MeOH/CH_2Cl_2); mp = 172–173 ºC; ν_{max} (film)/cm^{-1} 2945, 1621 (C=C), 1506 (C=C), 1454, 1323, 1238, 1168, 1118, 1066, 1021, 992, 910, 828, 727; ^1H NMR (400 MHz, $CDCl_3$) δ 8.81 (d, *J* = 4.5 Hz, 1 H, HC_{Ar}), 8.01 (d, *J* = 9.2 Hz, 1 H, HC_{Ar}), 7.84 (br s, 2 H, 2 × HC_{Ar}), 7.62 (d, *J* = 8.1 Hz, 2 H, 2 × HC_{Ar}), 7.44–7.31 (m, 7 H, 7 × HC_{Ar}), 6.32 (s, 1 H, CHOBn), 5.87 (s, 1 H, C*H*=CH_2), 5.67–5.52 (m, 1 H, CH=C*H*H), 5.15 (d, *J* = 17.2 Hz, 1 H, CH=CH*H*), 4.92–4.30 (m, 5 H, N^+CH_2Ar, OCH_2Ph and N^+C*H*H), 4.29– 4.04 (m, 3 H, N^+CH, N^+C*H*H), 3.10 (s, 2 H, N^+CH*H*), 2.54–2.45 (s, 1 H, N^+CH*H*), 2.44–2.34 (m, 1 H, $N^+(CH_2)_2$C*H*), 2.18 (d, *J* = 11.9 Hz, 1H), 2.03 (d, *J* = 3.2 Hz, 1H), 1.77 (s, 1H), 1.61 (t, *J* = 12.2 Hz, 1H) ; ^{13}C NMR (101 MHz, $CDCl_3$) δ 158.8 (OC_{Ar} quat), 146.7 (br s, C_{Ar} quat), 144.9 (C_{Ar} quat), 138.2 (C_{Ar} quat), 135.8 (2 × C_{Ar}), 135.7 (*C*H=CH_2), 134.5 (2 × C_{Ar}), 132.3 (q, $J_{C–F}$ = 33 Hz, $C_{Ar}CF_3$ quat), 131.7 (C_{Ar}), 131.3 (C_{Ar} quat), 129.3 (C_{Ar}), 129.09 and 129.06 (3 × C_{Ar}), 127.1 (C_{Ar} quat), 125.8 (q, $J_{C–F}$ = 4 Hz, 2 × C_{Ar}), 123.5 (q, *J* = 273 Hz, CF_3), 123.1 (br s, C_{Ar}), 120.3 (br s, C_{Ar} quat), 118.2 (CH_2=CH), 101.1 (br s, C_{Ar}), 70.8 (OCH_2Bn), 67.2 (CHOBn), 61.0 (N^+CH_2), 59.3 (N^+CH), 56.6 (OCH_3), 51.5 (N^+CH_2), 37.7 (CH_2=CH*C*H), 26.9 ($N(CH_2)_2$*C*H), 25.0 (N^+CH_2*C*H_2), 21.8 (N^+CH*C*H_2); ^{19}F NMR (377 MHz, $CDCl_3$) δ –63.0; HRMS (ESI$^+$) *m/z* Calculated for $C_{35}H_{36}N_2O_2F_3$ [M+H–Br] 573.2729; Found 573.2723.

SMILES:
C=C[C@H]1C[N@@+]2(CC3=CC=C(C(F)(F)F)C=C3)[C@H]([C@@H](C4=C(C=C(OC)C=C5)C5=NC=C4)OCC6=CC=CC=C6)C[C@@H]1CC2.[Br-]
InChI=1S/C35H36F3N2O2.BrH/c1-3-26-22-40(21-24-9-11-28(12-10-24)35(36,37)38)18-16-27(26)19-33(40)34(42-23-25-7-5-4-6-8-25)30-15-17-39-32-14-13-29(41-2)20-31(30)32;/h3-15,17,20,26-27,33-34H,1,16,18-19,21-23H2,2H3;1H/q+1;/p-1/t26-,27-,33-,34?,40+;/m0./s1

4-((*R*)-Hydroxy((1*S*,2*S*,4*S*,5*R*)-5-vinylquinuclidin-2-yl)methyl)quinolin-6-ol (S62)[28]

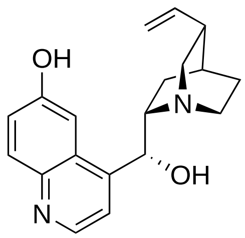

BBr_3 (1.0 M in CH_2Cl_2, 20.0 mL, 20 mmol) was added dropwise to a solution of quinine (1.62 g, 5.0 mmol) in CH_2Cl_2 (170 mL) at –78 ºC. The reaction mixture was allowed to warm to rt slowly over 1 h and then refluxed at 40 ºC for 1 h. The reaction mixture was cooled to 0 ºC then 1 M NaOH (50 mL) was added slowly and the layers were separated. The aqueous layer was extracted with CH_2Cl_2 (2 × 50 mL). The aqueous layer was acidified by addition of 1 M HCl to a pH of approx. 8.5. The resulting solution was extracted with CH_2Cl_2 (5 × 50 mL). The combined organic layers were dried over Na_2SO_4, filtered and concentrated under reduced pressure to afford 4-((*R*)-hydroxy((1*S*,2*S*,4*S*,5*R*)-5-vinylquinuclidin-2-yl)methyl)quinolin-6-ol **S62** (801 mg, 52%) as a yellow solid. $[\alpha]_D^{17}$ = –172º (*c* = 1.0, CH_3OH) [lit = –175.5º (*c* = 1.2, EtOH)][29]; R_f 0.22 (50% MeOH/CH_2Cl_2); mp = 198 ºC (decomposition observed) [lit = 198 ºC][30]; ν_{max} (film)/cm^{-1} 3075 (br s, OH), 2937, 2870, 1617 (C=C), 1510 (C=C), 1469, 1409, 1327, 1241, 1096, 936, 857, 828, 760; ^1H NMR (400 MHz, $CDCl_3$) δ 8.63 (d, *J* = 4.6 Hz, 1 H, HC_{Ar}), 7.94 (d, *J* = 9.1 Hz, 1 H, HC_{Ar}), 7.66 (d, *J* = 4.6 Hz, 1 H, HC_{Ar}), 7.37 (dd, *J* = 9.0, 2.5 Hz, 1 H, HC_{Ar}), 7.33 (d, *J* = 2.5 Hz, 1 H, HC_{Ar}), 5.79 (ddd, *J* = 17.4, 10.4, 7.5 Hz, 1 H, CH_2=C*H*), 5.57 (d, *J* = 3.2 Hz, 1 H, C*H*OH), 5.01 (dt, *J* = 17.1, 1.5 Hz, 1 H, C*H*H=CH),4.95 (dt, *J* = 10.4, 1.4 Hz, 1 H, CH*H*=CH), 3.80–3.70 (m, 1 H, NC*H*H), 3.20–3.11 (m, 2 H, NC*H*H and NCH), 2.82–2.68 (m, 2 H, 2 × NCH*H*), 2.40 (br s, 1 H, CH_2=CHC*H*), 1.97–1.81 (m, 3 H, NCHC*H*H, NCH_2C*H*H and $N(CH_2)_2$C*H*), 1.69–1.57 (m, 1 H, NCH_2CH*H*), 1.53–1.44 (m, 1 H, NCHCH*H*); ^{13}C NMR

(101 MHz, $CDCl_3$) δ 157.9 (OC_{Ar} quat), 149.7 (C_{Ar} quat), 147.4 (C_{Ar}), 144.0 (C_{Ar} quat), 142.5 (*C*H=CH_2), 131.4 (C_{Ar}), 128.4 (C_{Ar} quat), 123.3 (C_{Ar}), 119.8 (C_{Ar}), 115.0 (CH=*C*H_2), 105.1 (C_{Ar}), 72.1 (CHOH), 61.0 (NCH), 57.5 (NCH_2), 44.2 (NCH_2), 40.8 (CH_2=CH*C*H), 29.2 (N$(CH_2)_2$*CH*), 28.0 (NCH_2*C*H_2), 21.7 (NCH*C*H_2). The observed characterisation data (IR, ^{1}H and ^{13}C NMR) was consistent with that previously reported in the literature.[31]

SMILES: C=C[C@H]1C[N@]2[C@H]([C@@H](C3=C(C=C(O)C=C4)C4=NC=C3)O)C[C@@H]1CC2
InChI=1S/C19H22N2O2/c1-2-12-11-21-8-6-13(12)9-18(21)19(23)15-5-7-20-17-4-3-14(22)10-16(15)17/h2-5,7,10,12-13,18-19,22-23H,1,6,8-9,11H2/t12-,13-,18-,19+/m0/s1

(1*S*,2*S*,4*S*,5*R*)-2-((*R*)-Hydroxy(6-hydroxyquinolin-4-yl)methyl)-1-(4-(trifluoromethyl)benzyl)-5-vinylquinuclidin-1-ium bromide (QN4D)

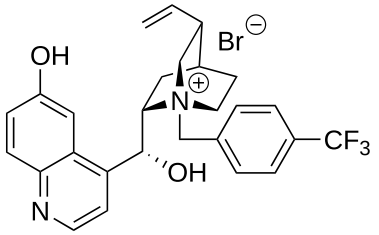

A solution of 4-((*R*)-Hydroxy((1*S*,2*S*,4*S*,5*R*)-5-vinylquinuclidin-2-yl)methyl)quinolin-6-ol **S62** (155 mg, 0.5 mmol) and 4-(trifluromethyl)benzyl bromide (120 mg, 1.0 mmol) in THF (2.5 mL) was heated to 70 ºC for 20 h. The reaction was cooled to rt and Et_2O was added. The reaction was stirred for approx. 0.5 h then the precipitate was filtered. The collected solid was dried under reduced pressure to afford (1*S*,2*S*,4*S*,5*R*)-2-((*R*)-hydroxy(6-hydroxyquinolin-4-yl)methyl)-1-(4-(trifluoromethyl)benzyl)-5-vinylquinuclidin-1-ium bromide **QN4D** (224 mg, 81%) as a pale purple solid. $[\alpha]_D^{17}$ = –72º (*c* = 0.5, CH_3OH) [lit = –178º (*c* = 1, MeOH)][32]; R_f 0.30 (5% MeOH/CH_2Cl_2); mp = 250–254 ºC (decomposition observed) [lit = 254–256 ºC (decomposition)][32]; ν_{max} (film)/cm^{-1} 3217 (OH), 3138, 2960, 1621 (C=C), 1469, 1323, 1260, 1219, 1163, 1092, 1066, 1014, 924, 861, 794, 697; ^{1}H NMR (400 MHz, DMSO-*d*^{6}) δ 10.14 (s, 1 H, OH), 8.74 (d, *J* = 4.5 Hz, 1 H, HC_{Ar}), 8.05–7.93 (m, 5 H, 5 × HC_{Ar}), 7.68 (d, *J* = 4.4 Hz, 1 H, HC_{Ar}), 7.52 (d, *J* = 2.5 Hz, 1 H, HC_{Ar}), 7.37 (dd, *J* = 9.1, 2.5 Hz, 1 H, HC_{Ar}), 6.59 (d, *J* = 4.3 Hz, 1 H, OH), 6.39 (d, *J* = 3.8 Hz, 1 H, N^+CH), 5.71 (ddd, *J* = 17.2, 10.5, 6.5 Hz, 1 H, CH_2=C*H*), 5.38 (d, *J* = 12.2 Hz, 1 H, N^+C*H*HAr), 5.19 (dt, *J* = 17.3, 1.2 Hz, 1 H, C*H*H=CH), 5.08 (d, *J* = 12.2 Hz, 1 H, N^+CH*H*Ar), 4.99 (dt, *J* = 10.6, 1.2 Hz, 1 H, CH*H*=CH), 4.27 (t, *J* = 10.9 Hz, 1 H, N^+C*H*H), 3.94 (t, *J* = 8.6 Hz, 1 H, C*H*OH), 3.85–3.77 (m, 1 H, N^+C*H*H), 3.39–3.20 (m, 1 H, N^+CH*H confirmed by HSQC*), 3.27–3.18 (m, 1 H, N^+CH*H*), 2.71–2.63 (br s, 1 H, CH_2=CHC*H*), 2.22–2.04 (m, 2 H, N^+CH_2C*H*H and N^+CHC*H*H), 2.03–1.97 (m, 1 H, N$(CH_2)_2$C*H*), 1.85–1.73 (m, 1 H, NCH_2CH*H*), 1.42–1.32 (m, 1 H, NCHCH*H*); ^{13}C NMR (101 MHz, DMSO-*d*^{6}) δ 155.9 (OC_{Ar} quat), 146.6 (C_{Ar} quat), 143.0 (C_{Ar} quat), 142.8 (CH_2=*C*H), 137.9 (C_{Ar}), 134.7 (2 × C_{Ar}), 132.6 (C_{Ar} quat), 131.5 (C_{Ar} quat), 130.3 (q, $J_{C\text{–}F}$ = 32 Hz, $C_{Ar}CF_3$ quat), 125.7 (q, *J* = 4 Hz, 2 × HC_{Ar}), 140.0 (q, $J_{C\text{–}F}$ = 273 Hz, CF_3), 121.7 (C_{Ar}), 119.9 (C_{Ar}), 116.5 (*C*H_2=CH), 104.3 (C_{Ar}), 68.0 (CHOH), 63.9 (N^+CH), 61.9 (N^+CH_2Ar), 59.0 (N^+CH_2), 50.6 (N^+CH_2), 36.9 (CH_2=CH*C*H), 25.8 (N$(CH_2)_2$*C*H), 24.1 (NCH_2*C*H_2), 20.5 (NCH*C*H_2); ^{19}F NMR (377 MHz, DMSO-*d*^{6}) δ –61.3. The observed characterisation data (IR, ^{1}H and ^{13}C NMR) was consistent with that previously reported in the literature.[32]

SMILES:
C=C[C@H]1C[N@@+]2(CC3=CC=C(C(F)(F)F)C=C3)[C@H]([C@@H](C4=C(C=C(O)C=C5)C5=NC=C4)O)C[C@@H]1CC2.[Br-]
InChI=1S/C27H27F3N2O2.BrH/c1-2-18-16-32(15-17-3-5-20(6-4-17)27(28,29)30)12-10-19(18)13-25(32)26(34)22-9-11-31-24-8-7-21(33)14-23(22)24;/h2-9,11,14,18-19,25-26,34H,1,10,12-13,15-16H2;1H/t18-,19-,25-,26+,32+;/m0./s1

(*R*)-(6-Methoxyquinolin-4-yl)((1*S*,2*S*,4*S*,5*R*)-5-vinylquinuclidin-2-yl)methyl methanesulfonate (S63)[33]

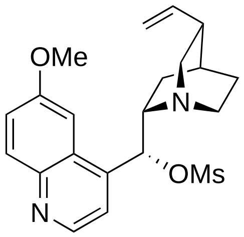

Mesyl chloride (1.54 mL, 20.0 mL) was added dropwise to a solution of quinine (3.24 g, 10.0 mmol) and NEt_3 (3.20 mL, 23.0 mmol) in THF (40 mL) at 0 ºC. The reaction mixture was stirred for 24 h then sat. aq. $NaHCO_3$ (100 mL) was added. The aqueous reaction mixture was extracted with CH_2Cl_2 (3 × 50 mL). The combined organic layers were dried over Na_2SO_4, filtered and concentrated under reduced pressure. Purification by flash chromatography (10% MeOH/EtOAc) afforded (*R*)-(6-methoxyquinolin-4-yl)((1*S*,2*S*,4*S*,5*R*)-5-vinylquinuclidin-2-yl)methyl methanesulfonate **S63** (2.62 g, 65%) as a pale yellow solid. $[\alpha]_D^{17}$ = –66º (*c* = 1, $CHCl_3$) [lit = –71.9º (*c* = 0.92, CH_2Cl_2)][34]; R_f 0.30 (10% MeOH/EtOAc); mp = 120–123 ºC [lit = 114–116.5 ºC][34]; ν_{max} (film)/cm^{-1} 2944, 2911, 2870, 1621, 1510, 1476, 1357, 1260, 1223, 1170, 1021, 984, 924, 869, 831, 678; ^{1}H NMR (400 MHz, $CDCl_3$) δ 8.79 (d, *J* = 4.3 Hz, 1 H, HC_{Ar}), 8.05 (d, *J* = 9.2 Hz, 1 H, HC_{Ar}), 7.50–7.34 (m, 3 H, 3 × HC_{Ar}), 6.18 (s, 1 H, HCOMs), 5.82 (ddd, *J* = 17.3, 9.8, 7.4 Hz, 1 H, CH_2=C*H*), 5.05–4.97 (m, 2 H, C*H_2*=CH), 3.96 (s, 3 H, OCH_3), 3.41 (br s, 1 H, NC*H*H), 3.17–3.06 (m, 1 H, NC*H*H), 3.03–2.92 (m, 1 H, NCH), 2.71–2.52 (m, 5 H, SO_2CH_3 and 2 × NCH*H*), 2.28 (br s, 1 H, CH_2=CHC*H*), 2.12–1.98 (s, 1 H, NCHC*H*H), 1.91 (br s, 1 H, N(CH_2)C*H*), 1.82–1.50 (m, 3 H, NCH_2C*H_2* and NCHCH*H*); ^{13}C NMR (101 MHz, $CDCl_3$) δ 158.3 (OC_{Ar} quat), 147.4 (C_{Ar}), 144.9 (C_{Ar} quat), 141.4 (C_{Ar}), 132.1 (C_{Ar}), 126.4 (*C*H_2=CH), 122.2 (C_{Ar} quat), 119.3 (br s, C_{Ar}), 114.6 (*C*H_2=CH), 100.7 (br s, C_{Ar} quat), 59.7 (br s, NCH), 56.4 (NCH_2), 55.7 (OCH_3), 42.2 (br s, NCH_2), 39.4 (*C*HCH=CH_2), 39.1 (SO_2CH_3), 27.5 (N(CH_2)$_2$*C*H), 27.2 (NCH*C*H_2), 24.9 (NCH_2CH_2). The observed characterisation data (IR, ^{1}H and ^{13}C NMR) was consistent with that previously reported in the literature.[34]

SMILES:
C=C[C@H]1C[N@]2[C@H]([C@@H](C3=C(C=C(OC)C=C4)C4=NC=C3)OS(C)(=O)=O)C[C@@H]1CC2
InChI=1S/C21H26N2O4S/c1-4-14-13-23-10-8-15(14)11-20(23)21(27-28(3,24)25)17-7-9-22-19-6-5-16(26-2)12-18(17)19/h4-7,9,12,14-15,20-21H,1,8,10-11,13H2,2-3H3/t14-,15-,20-,21?/m0/s1

(*S*)-(6-Methoxyquinolin-4-yl)((1*S*,2*S*,4*S*,5*R*)-5-vinylquinuclidin-2-yl)methanol (S64)

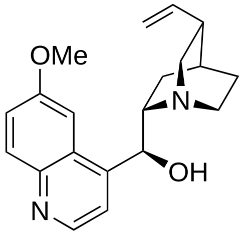

Tartaric acid (3.75 g, 25.0 mmol) was added to a solution of (*R*)-(6-Methoxyquinolin-4-yl)((1*S*,2*S*,4*S*,5*R*)-5-vinylquinuclidin-2-yl)methyl methanesulfonate (2.01 g, 5.0 mmol) in H_2O (50 mL) and heated at 100 ºC for 19 h. The reaction mixture was cooled to rt, then solid $NaHCO_3$ was added until no significant effervescence was observed. The aqueous reaction mixture was extracted with CH_2Cl_2 (3 × 100 mL). The combined organic layers were washed with sat. aq. $NaHCO_3$ (100 mL), dried over Na_2SO_4, filtered and concentrated under reduced pressure to afford (*S*)-(6-methoxyquinolin-4-yl)((1*S*,2*S*,4*S*,5*R*)-5-vinylquinuclidin-2-yl)methanol **S64** (1.41 g, 87%) as a brown solid. $[\alpha]_D^{17}$ = +30 (*c* = 1.35, $CHCl_3$) [lit = +43.3 (*c* = 1.0, EtOH)][35]; R_f 0.06 (20% MeOH/CH_2Cl_2); mp = 67–70 ºC [lit = 175 ºC][36]; ν_{max} (film)/cm^{-1} 3243 (br s, OH), 2930, 2863, 1618, 1506, 1472, 1357, 1320, 1238, 1081, 1025, 988, 913, 854, 716; ^{1}H NMR (400 MHz, $CDCl_3$) δ 8.75 (d, *J* = 4.5 Hz, 1 H, HC_{Ar}), 8.04 (d, *J* = 9.2 Hz, 1 H, HC_{Ar}), 7.66 (d, *J* = 2.8 Hz, 1 H, HC_{Ar}), 7.43–7.37 (m, 2 H, 2 × HC_{Ar}), 5.76 (ddd, *J* = 17.1, 10.3, 7.5 Hz, 1 H, CH_2=C*H*), 5.06–4.95 (m, 3 H, C*H_2*=CH and C*H*OH), 3.96 (s, 3 H, OCH_3), 3.33–3.08 (m, 3 H, 2 × NCH*H* and NCH), 2.86–2.77 (m, 2 H, 2 × NCH*H*), 2.39–2.30 (m, 1 H, CH_2=CHC*H*), 1.77–1.72 (m, 1 H, N(CH_2)$_2$C*H*), 1.66–1.60 (m, 2 H, NCHC*H*H and NCH_2C*H*H), 1.53–1.44 (m, 1 H, NCHCH*H*), 0.98 (ddt, *J* = 13.5, 7.8, 1.8 Hz, 1 H, NCH_2CH*H*); ^{13}C NMR (101 MHz, $CDCl_3$) δ 157.5 (OC_{Ar} quat), 147.6 (C_{Ar}), 144.9 (C_{Ar} quat), 144.3 (C_{Ar} quat), 141.4 (CH_2=*C*H), 131.7 (C_{Ar}), 128.2 (C_{Ar} quat), 121.3 (C_{Ar}), 120.1 (C_{Ar}), 114.7 (*C*H_2=CH), 102.6 (C_{Ar}), 71.4 (*C*HOH), 61.6 (NCH), 55.9 (NCH_2), 55.5 (OCH_3), 40.8 (NCH_2), 39.9 (CH_2=CHC*H*), 28.0 (N(CH_2)$_2$CH), 27.3 (NCH*C*H_2), 25.2 (NCH_2*C*H_2). The observed characterisation data (^{1}H and ^{13}C NMR) was consistent with that previously reported in the literature.[37]

SMILES: C=C[C@H]1C[N@]2[C@H]([C@H](C3=C(C=C(OC)C=C4)C4=NC=C3)O)C[C@@H]1CC2
InChI=1S/C20H24N2O2/c1-3-13-12-22-9-7-14(13)10-19(22)20(23)16-6-8-21-18-5-4-15(24-2)11-17(16)18/h3-6,8,11,13-14,19-20,23H,1,7,9-10,12H2,2H3/t13-,14-,19-,20-/m0/s1

(1*S*,2*S*,4*S*,5*R*)-2-((*S*)-Hydroxy(6-methoxyquinolin-4-yl)methyl)-1-(4-(trifluoromethyl)benzyl)-5-vinylquinuclidin-1-ium bromide (QN4')

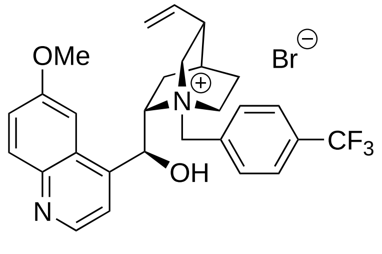

A solution of (*S*)-(6-methoxyquinolin-4-yl)((1*S*,2*S*,4*S*,5*R*)-5-vinylquinuclidin-2-yl)methanol **S64** (162 mg, 0.5 mmol) and 4-(trifluromethyl)benzyl bromide (120 mg, 0.5 mmol) in THF (2.5 mL) was heated to 70 ºC for 20 h. The reaction was cooled to rt and Et_2O was added. The reaction was stirred for approx. 0.5 h then the precipitate was filtered. The collected solid was dried to afford (1*S*,2*S*,4*S*,5*R*)-2-((*S*)-hydroxy(6-methoxyquinolin-4-yl)methyl)-1-(4-(trifluoromethyl)benzyl)-5-vinylquinuclidin-1-ium bromide **QN4'** (220 mg, 78%) as a pink solid. $[\alpha]_D^{17}$ = +72º (*c* = 1, CH_2Cl_2); R_f 0.38 (20% MeOH/CH_2Cl_2); mp = 160–165 ºC (decomposition observed); ν_{max} (film)/cm^{-1} 3191 (br s, OH, 3034, 2956, 1621, 1510, 1327, 1223, 1170, 1129, 1070, 921, 854, 731; ^{1}H NMR (400 MHz, $CDCl_3$) δ 8.60 (d, *J* = 4.5 Hz, 1 H, HC_{Ar}), 7.98 (d, *J* = 8.0 Hz, 2 H, 2 × HC_{Ar}), 7.91 (d, *J* = 9.2 Hz, 1 H, HC_{Ar}), 7.76 (d, *J* = 2.5 Hz, 1 H, HC_{Ar}), 7.57 (d, *J* = 8.0 Hz, 2 H, 2 × HC_{Ar}), 7.44 (d, *J* = 4.5 Hz, 1 H, HC_{Ar}), 7.31 (m, *J* = 9.2, 2.6 Hz, 1 H, HC_{Ar}), 6.73 (br s, 1 H, OH), 6.41 (d, *J* = 9.8 Hz, 1 H, N^+CH), 5.65–5.54 (m, 2 H, $N^+C\mathit{H}HAr$ and $CH_2{=}C\mathit{H}$), 5.47 (d, *J* = 12.8 Hz, 1 H, $N^+CH\mathit{H}Ar$), 5.14–5.06 (m, 2 H, $C\mathit{H_2}{=}CH$), 4.99 (t, *J* = 11.9 Hz, 1 H, $N^+C\mathit{H}H$), 4.41 (dt, *J* = 8.2, 2.8 Hz, 1 H, $C\mathit{H}OH$), 4.03 (s, 3 H, OCH_3), 3.97–3.72 (m, 2 H, $N^+C\mathit{H}H$ and $N^+CH\mathit{H}$), 3.29– 3.19 (m, 1 H, $N^+CH\mathit{H}$), 2.71 (q, *J* = 8.0 Hz, 1 H, $CH_2{=}CHC\mathit{H}$), 2.18–2.06 (m, 1 H, $N^+CHC\mathit{H}H$), 2.05–1.94 (m, 1 H, $N^+CH_2C\mathit{H}H$), 1.83 (br s, 1 H, $N^+(CH_2)_2C\mathit{H}$), 1.71–1.60 (m, 1 H, $N^+CH_2CH\mathit{H}$), 1.06–0.97 (m, 1 H, $N^+CHCH\mathit{H}$); ^{13}C NMR (101 MHz, $CDCl_3$) δ 158.7 (OC_{Ar} quat), 147.4 (C_{Ar}), 144.6 (C_{Ar} quat), 144.0 (C_{Ar} quat), 135.1 ($CH_2{=}CH$), 134.2 (2 × C_{Ar}), 132.3 (q, J_{C-F} = 33 Hz, $C_{Ar}CF_3$ quat), 131.8 (C_{Ar} quat), 131.6 (C_{Ar}), 127.2 (C_{Ar}), 125.8 (q, J_{C-F} = 3 Hz, 2 × C_{Ar}), 123.4 (q, J_{C-F} = 273 Hz, CF_3), 122.6 (C_{Ar}), 119.4 (C_{Ar} quat), 118.4 ($\mathit{C}H_2{=}CH$), 101.6 (C_{Ar}), 68.4 (N^+CH), 67.2 (CHOH), 64.9 (N^+CH_2Ar), 60.7 (N^+CH_2), 56.8 (OCH_3), 51.0 (N^+CH_2), 37.3 ($CH_2{=}CH\mathit{C}H$), 26.5 ($N^+(CH_2)_2\mathit{C}H$), 24.99 ($N^+CH_2\mathit{C}H_2$), 24.96 ($N^+CH\mathit{C}H_2$); ^{19}F{^{1}H} NMR (377 MHz, $CDCl_3$) δ –63.0; HRMS (FTMS + pAPCI) *m/z* Calculated for $C_{28}H_{30}N_2O_2F_3^+$ $[M–Br]^+$ 483.2254; Found 483.2244.

SMILES:
C=C[C@H]1C[N@@+]2(CC3=CC=C(C(F)(F)F)C=C3)[C@H]([C@H](C4=C(C=C(OC)C=C5)C5=NC=C4)O)C[C@@H]1CC2.[Br-]
InChI=1S/C28H30F3N2O2.BrH/c1-3-19-17-33(16-18-4-6-21(7-5-18)28(29,30)31)13-11-20(19)14-26(33)27(34)23-10-12-32-25-9-8-22(35-2)15-24(23)25;/h3-10,12,15,19-20,26-27,34H,1,11,13-14,16-17H2,2H3;1H/q+1;/p-1/t19-,20-,26-,27-,33+;/m0./s1

(*R*)-(6-Methoxy-2-phenylquinolin-4-yl)((1*S*,2*S*,4*S*,5*R*)-5-vinylquinuclidin-2-yl)methanol (S65)[38]

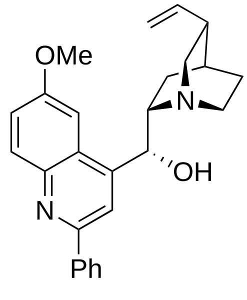

A solution of quinine (487 mg, 1.5 mmol) in MTBE (75 mL) was cooled to –10 ºC. PhLi (1.79 M in dibutyl ether, 2.56 mL, 4.58 mmol) was added over 5 min and was stirred for a further 25 min then slowly raised to 25 ºC then stirred for 4 h at 25 ºC. Acetic acid (1.0 mL) was added to the reaction at 0 ºC, then H_2O (2.5 mL) and EtOAc (2.5 mL) were added. I_2 (0.6 g) was added at 25 ºC, then sat. aq. $Na_2S_2O_3$ (10 mL) was added, followed by aq. NH_3 (28%, 5 mL) and the reaction mixture was stirred vigorously for 15 min. The layers were separated and the organic layer was washed with sat. aq. NaCl (10 mL). The combined aqueous layers were washed with CH_2Cl_2 (3 × 25 mL). The combined organic layers were dried over Na_2SO_4, filtred and concentrated under reduced pressure. Purification by flash chromatography (8:1:0.5:0.5 to 7:1:1.5:0.5 hexane:EtOAc:MeOH:NEt_3) afforded (*R*)-(6-methoxy-2-phenylquinolin-4-yl)((1*S*,2*S*,4*S*,5*R*)-5-vinylquinuclidin-2-yl)methanol **S65** (189 mg, 31%) as a pale yellow solid. $[\alpha]_D^{17}$ = –24º (*c* = 0.25, $CHCl_3$) [lit = –43.9º (*c* = 1.0, EtOH)][39]; R_f 0.15 (7:1:1.5:0.5 hexane:EtOAc:MeOH:NEt_3); mp = 137–138 ºC [lit = 145–147 ºC][39]; ν_{max} (film)/cm^{-1} 3700–2300 (br s, OH), 2933, 2863, 1618 (C=C), 1498, 1450, 1349, 1226, 1029, 906, 831, 731, 693; ^{1}H NMR (400 MHz, $CDCl_3$) δ 8.01 (d, *J* = 9.2 Hz, 1 H, HC_{Ar}), 7.92–7.88 (m, 2 H, 2 × HC_{Ar}), 7.80 (s, 1 H, HC_{Ar}), 7.39–7.32 (m, 3 H, 3 × HC_{Ar}), 7.26 (dd, *J* = 9.2, 2.6 Hz, 1 H, HC_{Ar}), 7.07 (d, *J* = 2.6 Hz, 1 H, HC_{Ar}), 5.67 (ddd, *J* = 17.6, 10.3, 7.7 Hz, 1 H,

CH_2=C*H*), 5.40 (d, *J* = 3.1 Hz, 1 H, C*H*OH), 4.95 – 4.82 (m, 2 H, C*H*$_2$=CH), 3.81 (s, 3 H, OCH_3), 3.49–3.38 (m, 1 H, NC*H*H), 3.06–2.97 (m, 2 H, NCH and NCH*H*), 2.62–2.51 (m, 2 H, 2 × NCH*H*), 2.26–2.18 (m, 1 H, CH_2=CHC*H*), 1.81–1.66 (m, 3 H, NCHC*H*H, NCH_2C*H*H and N(CH_2)$_2$C*H*), 1.48–1.36 (m, 2 H, NCHCH*H* and NCH_2CH*H*); ^{13}C NMR (101 MHz, $CDCl_3$) δ 157.4 (OC_{Ar} quat), 154.3 (C_{Ar} quat), 148.5 (C_{Ar} quat), 144.0 (C_{Ar} quat), 141.7 (C_{Ar}), 139.3 (CH_2=*C*H), 131.4 (C_{Ar}), 128.8 (C_{Ar}), 128.5 (2 × C_{Ar}), 127.1 (2 × C_{Ar}), 125.3 (C_{Ar} quat), 121.5 (C_{Ar}), 116.3 (*C*H_2=CH), 114.2 (C_{Ar}), 101.3 (C_{Ar}), 71.9 (*C*HOH), 59.8 (NCH), 56.9 (NCH_2), 55.5 (OCH_3), 43.1 (NCH_2), 39.8 (CH_2=CH*C*H), 27.8 (N(CH_2)$_2$*C*H), 27.4 (NCH_2*C*H_2), 21.1 (NCH*C*H_2). The observed characterisation data (^{1}H and ^{13}C NMR) was consistent with that previously reported in the literature.[40]

SMILES:
C=C[C@H]1C[N@]2[C@H]([C@@H](C3=C(C=C(OC)C=C4)C4=NC(C5=CC=CC=C5)=C3)O)C[C@@H]1C
C2
InChI=1S/C26H28N2O2/c1-3-17-16-28-12-11-19(17)13-25(28)26(29)22-15-24(18-7-5-4-6-8-18)27-23-10-9-20(30-2)14-21(22)23/h3-10,14-15,17,19,25-26,29H,1,11-13,16H2,2H3/t17-,19-,25-,26+/m0/s1

(1*S*,2*S*,4*S*,5*R*)-2-((*R*)-Hydroxy(6-methoxy-2-phenylquinolin-4-yl)methyl)-1-(4-(trifluoromethyl)benzyl)-5-vinylquinuclidin-1-ium bromide (QN4E) (Cat4)

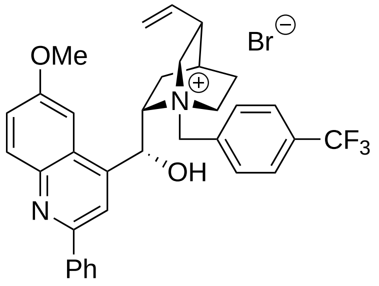

A mixture of (*R*)-(6-methoxy-2-phenylquinolin-4-yl)((1*S*,2*S*,4*S*,5*R*)-5-vinylquinuclidin-2-yl)methanol **S65** (80 mg, 0.16 mmol) and 4-(trifluromethyl)benzyl bromide (39 mg, 0.16 mmol) in toluene (1.0 mL) was heated to 75 ºC for 23 h. The reaction was cooled and concentrated under reduced pressure. Purification by flash chromatography (5% MeOH/CH_2Cl_2) afforded (1*S*,2*S*,4*S*,5*R*)-2-((*R*)-hydroxy(6-methoxy-2-phenylquinolin-4-yl)methyl)-1-(4-(trifluoromethyl)benzyl)-5-vinylquinuclidin-1-ium bromide **QN4E** (66 mg, 65%) as a white solid. $[\alpha]_D^{17}$ = –120º (*c* = 0.25, CH_3OH); R_f 0.42 (10% MeOH/CH_2Cl_2); ν_{max} (film)/cm^{-1} 3202 (br s, OH), 2982, 2948, 1621 (C=C), 1498, 1349, 1327, 1230, 1170, 1129, 1070, 1021, 831; ^{1}H NMR (400 MHz, $CDCl_3$) δ 8.22 (s, 1 H, HC_{Ar}), 8.13 (d, *J* = 7.4 Hz, 2 H, 2 × HC_{Ar}), 8.00–7.94 (m, 3 H, 3 × HC_{Ar}), 7.55 (d, *J* = 8.0 Hz, 2 H, 2 × HC_{Ar}), 7.46 (t, *J* = 7.5 Hz, 2 H, 2 × HC_{Ar}), 7.39 (t, *J* = 7.2 Hz, 1 H, HC_{Ar}), 7.27–7.25 (m, 1 H, HC_{Ar}), 7.22 (dd, *J* = 9.2, 2.4 Hz, 1 H, HC_{Ar}), 6.77–6.70 (m, 1 H,), 6.61 (s, 1 H, N^+CH), 6.02 (d, *J* = 12.0 Hz, 1 H, N^+C*H*HAr), 5.51 (ddd, *J* = 17.1, 10.5, 6.7 Hz, 1 H, CH_2=C*H*), 5.29 (d, *J* = 12.0 Hz, 1 H, N^+CH*H*Ar), 5.10 (d, *J* = 17.1 Hz, 1 H, C*H*H=CH), 4.93–4.80 (m, 2 H, CH*H*=CH and N^+C*H*H), 4.03 (t, *J* = 8.7 Hz, 1 H, N^+C*H*H), 3.89 (s, 3 H, OCH_3), 3.82–3.74 (m, 1 H, C*H*OH), 3.29 (t, *J* = 11.7 Hz, 1 H, N^+CH*H*), 3.01 (td, *J* = 11.3, 5.7 Hz, 1 H, N^+CH*H*), 2.54–2.45 (m, 1 H, CH_2=CHC*H*), 2.30–2.14 (m, 2 H, N^+CHC*H*H and N^+CH_2C*H*H), 2.00–1.87 (s, 1 H, N(CH_2)$_2$C*H*), 1.68 (t, *J* = 11.3 Hz, 1 H, N^+CH_2CH*H*), 1.47–1.37 (m, 1 H, N^+CHCH*H*); ^{13}C NMR (101 MHz, $CDCl_3$) δ 157.8 (OC_{Ar} quat), 154.3 (C_{Ar} quat), 144.3 (C_{Ar} quat), 143.3 (C_{Ar} quat), 139.2 (CH_2=*C*H), 136.0 (C_{Ar}), 134.4 (2 × C_{Ar}), 132.4 (q, $J_{C\text{–}F}$ = 4 Hz, *C*$_{Ar}CF_3$ quat), 132.1 (C_{Ar}), 131.1 (C_{Ar} quat), 129.0 (C_{Ar}), 128.7 (2 × C_{Ar}), 127.3 (2 × C_{Ar}), 125.9 (q, $J_{C\text{–}F}$ = 3 Hz, 2 × HC_{Ar}), 124.8 (C_{Ar}), 123.4 (q (obscured by other peaks), $J_{C\text{–}F}$ = 273 Hz, CF_3), 120.7 (C_{Ar}), 118.2 (C_{Ar}), 118.0 (*C*H_2=*C*H), 102.1 (C_{Ar}), 69.3 (CHOH), 65.0 (N^+CH), 62.0 (N^+CH_2Ar), 60.7 (N^+CH_2), 56.2 (OCH_3), 51.3 (N^+CH_2), 37.9 (CH_2=CH*C*H), 26.6 (N^+(CH_2)$_2$*C*H), 24.9 (N^+CH_2*C*H_2), 21.9 (N^+CH*C*H); ^{19}F NMR (377 MHz, $CDCl_3$) δ –63.0. HRMS (ESI$^+$) *m/z* Calculated for $C_{34}H_{34}N_2O_2F_3$ [M+H–Br] 559.2572; Found 559.2574.

SMILES:
C=C[C@H]1C[N@@+]2(CC3=CC=C(C(F)(F)F)C=C3)[C@H]([C@@H](C4=C(C=C(OC)C=C5)C5=NC(C6=C
C=CC=C6)=C4)O)C[C@@H]1CC2.[Br-]
InChI=1S/C34H34F3N2O2.BrH/c1-3-23-21-39(20-22-9-11-26(12-10-22)34(35,36)37)16-15-25(23)17-32(39)33(40)29-19-31(24-7-5-4-6-8-24)38-30-14-13-27(41-2)18-28(29)30;/h3-14,18-19,23,25,32-33,40H,1,15-17,20-21H2,2H3;1H/q+1;/p-1/t23-,25-,32-,33+,39+;/m0./s1

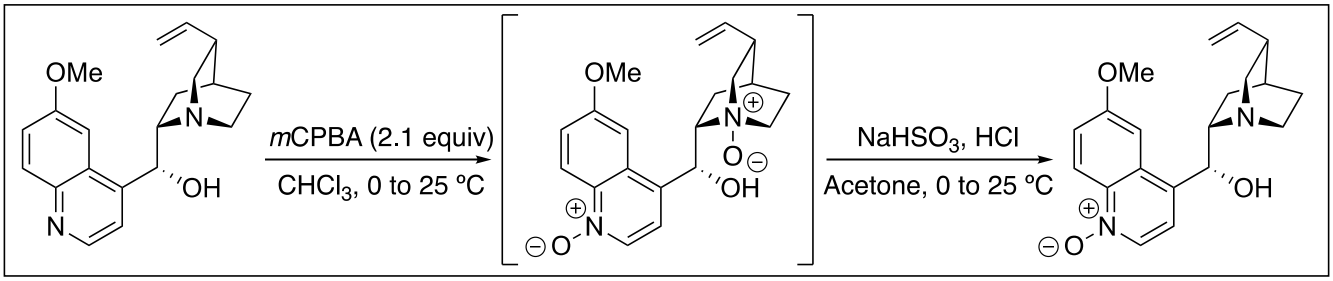

4-((*R*)-Hydroxy((1*S*,2*S*,4*S*,5*R*)-5-vinylquinuclidin-2-yl)methyl)-6-methoxyquinoline 1-oxide (S66)[41]

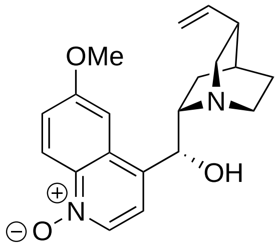

*m*CPBA (1.81 g, 10.5 mmol) was added to a solution of quinine (1.62 g, 5.0 mmol) in $CHCl_3$ (20 mL) at 0 ºC. Then the reaction mixture was stirred at 25 ºC for 3 h. The reaction was quenched with aq. NaOH (10%) until pH~10 and the aqueous reaction mixture was extracted with $CHCl_3$/MeOH (10:1, 10 × 10 mL). The combined organic layers were dried over Na_2SO_4, filtered and concentrated under reduced pressure to afford impure (1*R*,2*S*,4*S*,5*R*)-2-((*R*)-hydroxy(6-methoxy-1-oxidoquinolin-4-yl)methyl)-5-vinylquinuclidine 1-oxide **S70** (1.94 g) which was used without further purification. 1 M HCl (10 mL) was added to $NaHSO_3$ (1.37 g, 13.2 mmol) and stirred for 1 h at 0 ºC. A solution of **S70** (1.84 g, 3.2 mmol) in acetone (16 mL) was added over 10 min at 0 ºC. The reaction mixture was stirred at 25 ºC for 18 h, then the solvent was removed unde reduced pressure. NH_4OH (1 mL) was added then the aqueous reaction mixture was extracted with $CHCl_3$ (5 × 20 mL). The combined organic layers were washed with sat. aq. NaCl (50 mL), dried over Na_2SO_4, filtered and concentrated under reduced pressure. Purification by flash chromatography (5% MeOH/CH_2Cl_2 + 1% NEt_3) afforded product contaminated with HN^+Et_3. To this impure product H_2O (20 mL) and $CHCl_3$ (20 mL) was added and the layers separated. The organic layer was washed with H_2O (2 × 20 mL). The combined aqueous layers were washed with $CHCl_3$ (3 × 20 mL), dried over Na_2SO_4, filtered and concentrated under reduced pressure to afford 4-((*R*)-hydroxy((1*S*,2*S*,4*S*,5*R*)-5-vinylquinuclidin-2-yl)methyl)-6-methoxyquinoline 1-oxide **S66** (668 mg, 61% based on amount of **xx** added and assuming 100% purity of **S66**). $[\alpha]_D^{17}$ = –116º (*c* = 0.5, $CHCl_3$); R_f 0.29 (5% MeOH/CH_2Cl_2 + 1% NEt_3); mp = 75–77 ºC [lit = 103–107 ºC][42]; ν_{max} (film)/cm^{-1} 3079 (br s, OH), 2933, 2863, 1618 (C=C), 1573 (C=C), 1469, 1431, 1290, 1252, 1197, 1167, 1100, 1066, 1025, 910, 831, 731; ^{1}H NMR (400 MHz, $CDCl_3$) δ 8.36 (d, *J* = 9.5 Hz, 1 H, HC_{Ar}), 7.84 (d, *J* = 6.3 Hz, 1 H, HC_{Ar}), 7.16–7.09 (m, 2 H, 2 × HC_{Ar}), 6.88 (d, *J* = 2.3 Hz, 1 H, HC_{Ar}), 6.25 (s, 1 H, OH), 5.71 (ddd, *J* = 17.4, 10.3, 7.6 Hz, 1 H, CH_2=C*H*), 5.15 (d, *J* = 4.7 Hz, 1 H, C*H*OH), 4.95–4.85 (m, 2 H, CH_2=CH), 3.84 (s, 3 H, OCH_3), 3.48–3.38 (m, 1 H, NC*H*H), 3.10–2.96 (m, 1 H, NCH), 2.90–2.81 (m, 1 H, NC*H*H), 2.63–2.48 (m, 2 H, 2 × NCH*H*), 2.27–2.18 (m, 1 H, CH_2=CHC*H*), 1.80–1.69 (m, 3 H, NCH_2C*H*H, NCHC*H*H and N$(CH_2)_2$C*H*), 1.60–1.41 (m, 2 H, NCHCH*H* and NCH_2CH*H*); ^{13}C NMR (101 MHz, $CDCl_3$) δ 158.8 (OC_{Ar} quat), 142.3 (C_{Ar} quat), 141.8 (CH_2=*C*H), 135.1 (C_{Ar} quat), 133.6 (C_{Ar}), 128.4 (C_{Ar} quat), 122.5 (C_{Ar}), 120.9 (C_{Ar}), 118.4 (C_{Ar}), 114.2 (*C*H_2=CH), 101.7 (C_{Ar}), 70.9 (*C*HOH), 60.2 (NCH), 56.8 (NCH_2), 55.7 (OCH_3), 42.9 (NCH_2), 39.9 (CH_2=CH*C*H), 27.7 (N$(CH_2)_2$*C*H), 27.5 (NCH_2*C*H_2), 22.1 (NCH*C*H_2). The observed characterisation data (^{1}H and ^{13}C NMR) was consistent with that previously reported in the literature.[42]

SMILES:C=C[C@H]1C[N@]2[C@H]([C@@H](C3=C(C=C(OC)C=C4)C4=[N+]([O-])C=C3)O)C[C@@H]1CC2
InChI=1S/C20H24N2O3/c1-3-13-12-21-8-6-14(13)10-19(21)20(23)16-7-9-22(24)18-5-4-15(25-2)11-17(16)18/h3-5,7,9,11,13-14,19-20,23H,1,6,8,10,12H2,2H3/t13-,14-,19-,20+/m0/s1

4-((*R*)-Hydroxy((1*S*,2*S*,4*S*,5*R*)-1-(4-(trifluoromethyl)benzyl)-5-vinylquinuclidin-1-ium-2-yl)methyl)-6-methoxyquinoline 1-oxide bromide (QN4F)[41]

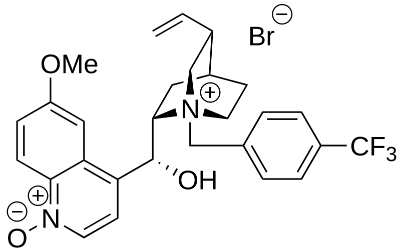

A mixture of (1*R*,2*S*,4*S*,5*R*)-2-((*R*)-hydroxy(6-methoxy-1-oxidoquinolin-4-yl)methyl)-5-vinylquinuclidine 1-oxide **S66** (85 mg, 0.25 mmol) and 4-(trifluromethyl)benzyl bromide (72 mg, 0.30 mmol) in MeCN:MeOH (4:1, 0.625 mL) was heated to 30 ºC for 23 h. The reaction was cooled and concentrated under reduced pressure. Purification by flash chromatography (5% to 10% MeOH/CH_2Cl_2) afforded 4-((*R*)-hydroxy((1*S*,2*S*,4*S*,5*R*)-1-(4-(trifluoromethyl)benzyl)-5-vinylquinuclidin-1-ium-2-yl)methyl)-6-methoxyquinoline 1-oxide bromide **QN4F** (121 mg, 83%) as a pale yellow solid. $[\alpha]_D^{14}$ = –140º (*c* = 0.1, CH_3OH); R_f 0.29 (10% MeOH/CH_2Cl_2); mp = 200–202 ºC (decomposition observed); ν_{max} (film)/cm^{-1} 3355 (OH), 3217, 2948, 1618 (C=C), 1465, 1431, 1323, 1252, 1215, 1163, 1118, 1066, 1021, 831, 701; ^{1}H NMR (400 MHz, DMSO-*d*^{6}) δ 8.56 (d, *J* = 9.5 Hz, 1 H, HC_{Ar}), 8.50 (d, *J* = 6.4 Hz, 1 H, HC_{Ar}), 7.98–7.92 (m, 4 H, 4 × HC_{Ar}), 7.65 (d, *J* = 6.4 Hz, 1 H, HC_{Ar}), 7.52 (dd, *J* = 9.5, 2.4 Hz, 1 H, HC_{Ar}), 7.44 (d, *J* = 2.5 Hz, 1 H, HC_{Ar}), 6.90 (d, *J* = 4.6 Hz, 1 H, OH), 6.50 (d, *J* = 4.4 Hz, 1 H, N$^+$CH), 5.77 (ddd, *J* = 17.4, 10.4, 7.1 Hz, 1 H, CH_2=C*H*), 5.53 (d, *J* = 12.3 Hz, 1 H, N$^+$C*H*HAr), 5.15–5.09 (m, 1 H, C*H*H=CH), 5.01 (d, *J* = 10.5 Hz, 1 H, CH*H*=CH), 4.83 (d, *J* = 12.3 Hz, 1 H, N$^+$CH*H*Ar), 4.31 (t, *J* = 11.2 Hz, 1 H, N$^+$C*H*H), 4.04 (s, 3 H, OCH_3), 3.90–3.82 (m, 1 H, C*H*OH), 3.77–3.69 (m, 1 H, N$^+$C*H*H), 3.39–3.31 (m, 1 H, N$^+$CH*H*), 3.21 (td, *J* = 11.2, 6.1 Hz, 1 H, N$^+$CH*H*), 2.72–2.63 (m, 1 H, CH_2=CHC*H*), 2.23–2.05 (m, 2 H, N$^+CH_2$C*H*H and N$^+$CHC*H*H), 2.03–1.97 (m, 1 H, N$^+(CH_2)_2$C*H*), 1.87–1.75 (m, 1 H, NCH_2CH*H*), 1.64–1.53 (m, 1 H, NCHCH*H*); ^{13}C NMR (101 MHz, DMSO-*d*^{6}) δ 159.0 (OC_{Ar} quat), 138.0 (CH_2=*C*H), 136.0 (C_{Ar} quat), 134.5 (2 × C_{Ar}), 132.7 (C_{Ar}), 132.62 (C_{Ar}), 132.58 (C_{Ar} quat), 130.3 (q, $J_{C–F}$ = 33 Hz, $C_{Ar}CF_3$ quat), 128.0 (C_{Ar}), 125.8 (q, *J* = 4 Hz, 2 × C_{Ar}), 124.0 (q, $J_{C–F}$ = 273 Hz, CF_3), 121.5 (C_{Ar}), 121.4 (C_{Ar} quat), 116.6 (*C*H_2=CH), 103.6 (C_{Ar}), 68.5 (CHOH), 63.4 (N$^+$CH), 62.2 (N$^+CH_2$Ar), 59.3 (N$^+CH_2$), 55.9 (OCH_3), 50.7 (N$^+CH_2$), 37.1 (CH_2=CHCH), 26.0 (N$^+(CH_2)_2$CH), 24.2 (N$^+CH_2CH_2$), 20.4 (N$^+$CH*C*H_2); ^{19}F{^{1}H NMR} NMR (377 MHz, DMSO-*d*^{6}) δ –61.3; HRMS (FTMS + pAPCI) *m/z* Calculated for $C_{28}H_{30}N_2O_3F_3^+$ $[M+H]^+$ 499.2203; Found 499.2183.

SMILES:
C=C[C@H]1C[N@@+]2(CC3=CC=C(C(F)(F)F)C=C3)[C@H]([C@@H](C4=C(C=C(OC)C=C5)C5=[N+]([O-])C=C4)O)C[C@@H]1CC2.[Br-]
InChI=1S/C28H30F3N2O3.BrH/c1-3-19-17-33(16-18-4-6-21(7-5-18)28(29,30)31)13-11-20(19)14-26(33)27(34)23-10-12-32(35)25-9-8-22(36-2)15-24(23)25;/h3-10,12,15,19-20,26-27,34H,1,11,13-14,16-17H2,2H3;1H/q+1;/p-1/t19-,20-,26-,27+,33+;/m0./s1

(1*S*,2*S*,4*S*,5*R*)-5-Ethyl-2-((*R*)-hydroxy(6-methoxyquinolin-4-yl)methyl)-1-(4-(trifluoromethyl)benzyl)quinuclidin-1-ium bromide (HQN1) (Cat3)

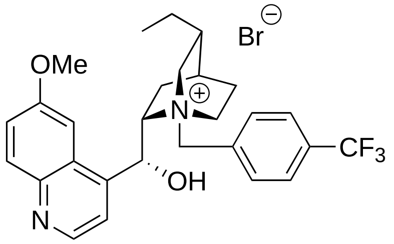

A mixture of dihydroquinine (163 mg, 0.5 mmol) and 4-(trifluromethyl)benzyl bromide (120 mg, 0.50 mmol) in THF (2.5 mL) was heated to 70 ºC for 15.5 h. The reaction was cooled and Et_2O was added. The reaction was stirred for approx. 0.5 h then the precipitate was filtered. The collected solid was dried to afford (1*S*,2*S*,4*S*,5*R*)-5-ethyl-2-((*R*)-hydroxy(6-methoxyquinolin-4-yl)methyl)-1-(4-(trifluoromethyl)benzyl)quinuclidin-1-ium bromide **HQN1** (224 mg, 79%) as a white solid. $[\alpha]_D^{17}$ = –108º (*c* = 1.06, CH_2Cl_2); R_f 0.20 (10% MeOH/CH_2Cl_2); mp = 236–238 ºC (decomposition observed); ν_{max} (film)/cm^{-1} 3213 (OH), 2963, 1621, 1510, 1327, 1241, 1170, 1129, 1070, 1021, 857, 828; ^{1}H NMR (400 MHz, $CDCl_3$) δ 8.69 (d, *J* = 4.5 Hz, 1 H, HC_{Ar}), 8.01–7.94 (m, 3 H, 3 × HC_{Ar}), 7.69 (d, *J* = 4.5 Hz, 1 H, HC_{Ar}), 7.58 (d, *J* = 8.1 Hz, 2 H, HC_{Ar}), 7.29–7.25 (m, 2 H, 2 × HC_{Ar}), 6.62 (d, *J* = 6.6 Hz, 1 H, N$^+$CH), 6.35 (d, *J* = 6.7 Hz, 1 H, OH), 6.08 (d, *J* = 12.0 Hz, 1 H, N$^+$C*H*HAr), 4.91 (d, *J* = 12.0 Hz, 1 H, N$^+$C*H*HAr), 4.87–4.78 (m, 1 H, N$^+$C*H*H), 3.99–3.88 (m, 4 H, OCH_3 and C*H*OH), 3.51–3.44 (m, 1 H, N$^+$C*H*H), 3.32 (dd, *J* = 12.4, 10.7 Hz, 1 H, N$^+$CH*H*), 2.97 (td, *J* = 11.4, 6.4 Hz, 1 H, N$^+$CH*H*), 2.31–2.18 (m, 2 H, N$^+CH_2$C*H*H and N$^+$CHC*H*H), 1.99–1.91 (m, 1 H, N$^+(CH_2)_2$C*H*), 1.74–1.58 (m, 2 H, N$^+CH_2$C*H* and N$^+CH_2$CH*H*), 1.47–1.38 (m, 1 H, N$^+$CHCH*H*), 1.29–1.08 (m, 2 H, CH_3C*H_2*), 0.66 (t, *J* =

7.3 Hz, 3 H, CH_3CH$_2$); ^{13}C NMR (101 MHz, $CDCl_3$) δ 158.0 (OC$_{Ar}$ quat), 147.4 (C$_{Ar}$), 144.1 (C$_{Ar}$ quat), 142.9 (C$_{Ar}$ quat), 134.4 (2 × C$_{Ar}$), 132.4 (q, $J_{C–F}$ = 33 Hz, *C*$_{Ar}$CF$_3$ quat), 131.9 (C$_{Ar}$ quat), 131.1 (C$_{Ar}$ quat), 125.9 (3 × C$_{Ar}$), 123.5 (q, $J_{C–F}$ = 273 Hz, CF$_3$), 120.8 (C$_{Ar}$), 120.6 (C$_{Ar}$), 102.1 (C$_{Ar}$), 69.5 (CHOH), 64.0 (N$^+$CH), 62.9 (N$^+$CH$_2$Ar), 62.1 (N$^+$CH$_2$), 56.3 (OCH$_3$), 51.4 (N$^+$CH$_2$), 36.0 (N$^+$CH$_2$C*H*), 26.0 (CH$_3$*C*H$_2$), 25.2 (N$^+$CH$_2$*C*H$_2$), 23.9 (N$^+$(CH$_2$)$_2$*C*H), 21.3 (N$^+$CH*C*H$_2$), 11.2 (*C*H$_3$CH$_2$); ^{19}F{^{1}H} NMR (377 MHz, $CDCl_3$) δ –63.0; HRMS (FTMS + pAPCI) *m/z* Calculated for $C_{28}H_{32}N_2O_2F_3^+$ [M–Br]$^+$ 485.2410; Found 485.2391.

SMILES:
CC[C@H]1C[N@@+]2(CC3=CC=C(C(F)(F)F)C=C3)[C@H]([C@@H](C4=C(C=C(OC)C=C5)C5=NC=C4)O)C[C@@H]1CC2.[Br-]
InChI=1S/C28H32F3N2O2.BrH/c1-3-19-17-33(16-18-4-6-21(7-5-18)28(29,30)31)13-11-20(19)14-26(33)27(34)23-10-12-32-25-9-8-22(35-2)15-24(23)25;/h4-10,12,15,19-20,26-27,34H,3,11,13-14,16-17H2,1-2H3;1H/q+1;/p-1/t19-,20-,26-,27+,33+;/m0./s1

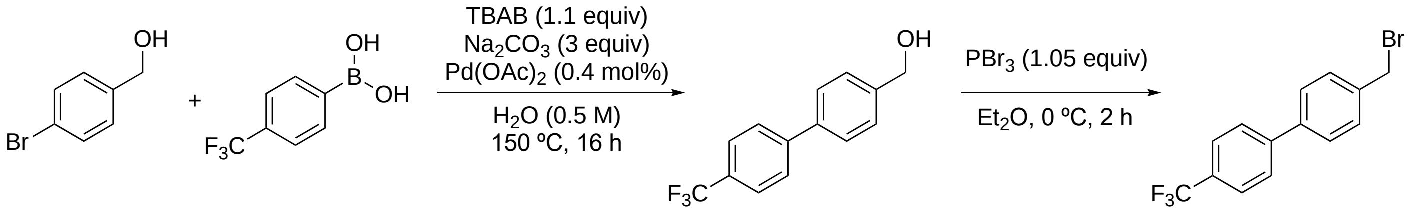

(4'-(Trifluoromethyl)-[1,1'-biphenyl]-4-yl)methanol (S67)[43]

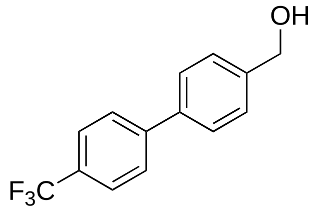

4-Bromobenzyl alcohol (187 mg, 1.0 mmol), (4-(trifluoromethyl)phenyl)boronic acid (209 mg, 1.1 mmol), TBAB (322 mg, 1.0 mmol), Na_2CO_3 (318 mg, 3.0 mmol) and $Pd(OAc)_2$ (0.9 mg, 4.0 μmol) were added to a vial. The vial was evacuated and backfilled with N_2 (3 ×) then H_2O (2.0 mL) was added and the reaction was stirred at 150 ºC for 16 h. The vial was cooled to rt then EtOAc (20 mL) and H_2O (20 mL) was added and the layers were separated. The aqueous layer was extracted with EtOAc (3 × 20 mL). The combined organic layers were dried over Na_2SO_4, filtered and concentrated under reduced pressure. Purification by flash chromatography (20% EtOAc/hexane) afforded (4'-(trifluoromethyl)-[1,1'-biphenyl]-4-yl)methanol **S67** (209 mg, 83%) as a white solid. R_f 0.28 (20% EtOAc/hexane); mp = 148–150 ºC; ν_{max} (film)/cm^{-1} 3401 (br s, OH), 1399, 1335, 1170, 1128, 1076, 1016, 811; ^{1}H NMR (400 MHz, $CDCl_3$) δ 7.70 (s, 4 H, 4 × HC$_{Ar}$), 7.62 (d, *J* = 8.3 Hz, 2 H, 2 × HC$_{Ar}$), 7.49 (d, *J* = 8.3 Hz, 2 H, 2 × HC$_{Ar}$), 4.78 (s, 2 H, CH$_2$Br); ^{13}C NMR (101 MHz, $CDCl_3$) δ 144.3 (C$_{Ar}$ quat), 140.9 (C$_{Ar}$ quat), 139.1 (C$_{Ar}$ quat), 129.4 (q, $J_{C–F}$ = 32 Hz, *C*$_{Ar}$CF$_3$ quat), 127.6 (2 × C$_{Ar}$), 127.4 (2 × C$_{Ar}$), 127.3 (2 × C$_{Ar}$), 125.7 (q, $J_{C–F}$ = 4 Hz, 2 × C$_{Ar}$), 124.3 (q, $J_{C–F}$ = 272 Hz, CF$_3$), 64.9 (OCH$_2$); ^{19}F{^{1}H} NMR (377 MHz, $CDCl_3$) δ –62.4. The observed characterisation data (^{1}H and ^{13}C NMR) was consistent with that previously reported in the literature.[43]

SMILES: OCC1=CC=C(C2=CC=C(C(F)(F)F)C=C2)C=C1
InChI=1S/C14H11F3O/c15-14(16,17)13-7-5-12(6-8-13)11-3-1-10(9-18)2-4-11/h1-8,18H,9H2

4-(Bromomethyl)-4'-(trifluoromethyl)-1,1'-biphenyl (S68)[44]

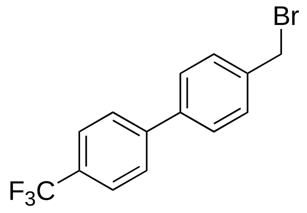

Phosphorus tribromide (59 μL, 0.63 mmol) was added dropwise to a solution of (4'-(trifluoromethyl)-[1,1'-biphenyl]-4-yl)methanol **S67** (151 mg, 0.6 mmol) in Et_2O (3.0 mL) at 0 ºC. The reaction mixture was stirred for 2 h at 0 ºC then H_2O (20 mL) was added. The aqueous reaction mixture was extracted with EtOAc (3 × 20 mL). The combined organic layers were washed with sat. aq. NaCl (30 mL), dried over Na_2SO_4, filtered and concentrated under reduced pressure. Purification by flash chromatography (10% EtOAc/hexane) afforded 4-(bromomethyl)-4'-(trifluoromethyl)-

1,1'-biphenyl **S68** (91 mg, 48%) as a white solid. R_f 0.73 (40% EtOAc/hexane); mp = 77–79 ºC; ν_{max} (film)/cm^{-1} 1616, 1398, 1329, 1167, 1124, 1113, 1073, 832, 820; ^{1}H NMR (400 MHz, $CDCl_3$) δ 7.73–7.67 (m, 4 H, 4 × HC_{Ar}), 7.59 (d, *J* = 8.3 Hz, 2 H, 2 × HC_{Ar}), 7.51 (d, *J* = 8.3 Hz, 2 H, 2 × HC_{Ar}), 4.56 (s, 2 H, CH_2Br); ^{13}C NMR (101 MHz, $CDCl_3$) δ 143.9 (C_{Ar} quat), 139.8 (C_{Ar} quat), 137.8 (C_{Ar} quat), 129.7 (2 × C_{Ar}), 129.6 (q, $J_{C–F}$ = 33 Hz, $C_{Ar}CF_3$ quat), 127.7 (2 × C_{Ar}), 127.4 (2 × C_{Ar}), 125.8 (q, $J_{C–F}$ = 4 Hz, 2 × C_{Ar}), 124.2 (q, $J_{C–F}$ = 272 Hz, CF_3), 33.0 (CH_2Br); ^{19}F{^{1}H} NMR (377 MHz, $CDCl_3$) δ –62.5. The observed characterisation data (^{1}H NMR) was consistent with that previously reported in the literature.[45]

SMILES: BrCC1=CC=C(C2=CC=C(C(F)(F)F)C=C2)C=C1
InChI=1S/C14H10BrF3/c15-9-10-1-3-11(4-2-10)12-5-7-13(8-6-12)14(16,17)18/h1-8H,9H2

(1*S*,2*S*,4*S*,5*R*)-2-((*R*)-Hydroxy(6-methoxyquinolin-4-yl)methyl)-1-((4'-(trifluoromethyl)-[1,1'-biphenyl]-4-yl)methyl)-5-vinylquinuclidin-1-ium (QN19)

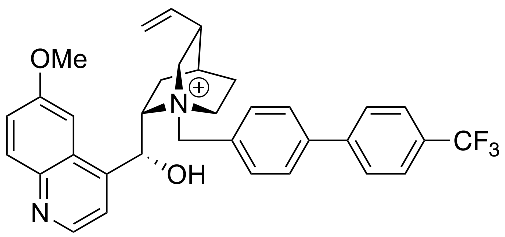

A mixture of quinine (32 mg, 0.1 mmol) and 4-(bromomethyl)-4'-(trifluoromethyl)-1,1'-biphenyl **S68** (32 mg, 0.1 mmol) in THF (0.5 mL) was heated to 70 ºC for 36 h. The reaction was cooled and Et_2O (2.5 mL) was added. The reaction was stirred for approx. 0.5 h then the precipitate was filtered. Purification by flash chromatography (10% MeOH/CH_2Cl_2) afforded (1*S*,2*S*,4*S*,5*R*)-2-((*R*)-hydroxy(6-methoxyquinolin-4-yl)methyl)-1-((4'-(trifluoromethyl)-[1,1'-biphenyl]-4-yl)methyl)-5-vinylquinuclidin-1-ium **QN19** (30 mg, 47%) as a pale brown solid. $[\alpha]_D^{17}$ = –180º (*c* = 0.1, $CHCl_3$); R_f 0.51 (20% MeOH/CH_2Cl_2); mp = 188–191 ºC; ν_{max} (film)/cm^{-1} 3198 (br s, OH), 2948, 1618, 1510, 1327, 1241, 1167, 1126, 828, 731; ^{1}H NMR (400 MHz, $CDCl_3$) δ 8.71 (d, *J* = 4.5 Hz, 1 H, HC_{Ar}), 7.97 (d, *J* = 9.2 Hz, 1 H, HC_{Ar}), 7.87 (d, *J* = 8.2 Hz, 2 H, 2 × HC_{Ar}), 7.76 (d, *J* = 4.5 Hz, 1 H, HC_{Ar}), 7.68 (d, *J* = 8.2 Hz, 2 H, 2 × HC_{Ar}), 7.57 (d, *J* = 8.1 Hz, 2 H, HC_{Ar}), 7.52 (d, *J* = 8.3 Hz, 2 H, 2 × HC_{Ar}), 7.32 (d, *J* = 2.6 Hz, 1 H, HC_{Ar}), 7.27 (dd, *J* = 9.2, 2.6 Hz, 1 H, HC_{Ar}), 6.83 (d, *J* = 5.7 Hz, 1 H, OH), 6.70–6.65 (s, 1 H, N^+CH), 6.15 (d, *J* = 12.1 Hz, 1 H, N^+C*H*HAr), 5.60 (ddd, *J* = 17.2, 10.4, 6.9 Hz, 1 H, CH_2=C*H*), 5.15 (d, *J* = 17.1 Hz, 1 H, C*H*H=CH), 5.07–4.93 (m, 3 H, N^+CH*H*Ar, CH*H*=CH and N^+C*H*H), 3.94–3.88 (s, 4 H, OCH_3 and C*H*OH), 3.57–3.43 (m, 2 H, N^+C*H*$_2$), 3.16 (dt, *J* = 11.4, 6.0 Hz, 1 H, N^+CH*H*), 2.64–2.56 (m, 1 H, CH_2=CHC*H*), 2.36– 2.18 (m, 2 H, N^+CHC*H*H and N^+CH_2C*H*H), 2.06–2.00 (m, 1 H, N^+(CH-$_2$)$_2$C*H*), 1.80–1.70 (m, 1 H, N^+CH_2CH*H*), 1.53–1.43 (m, 1 H, N^+CHCH*H*); ^{13}C NMR (101 MHz, $CDCl_3$) δ 158.0 (OC_{Ar} quat), 147.5 (C_{Ar}), 144.2 (C_{Ar} quat), 143.0 (C_{Ar} quat), 142.9 (C_{Ar} quat), 141.6 (C_{Ar} quat), 136.2 (CH_2=*C*H), 134.5 (2 × C_{Ar}), 132.0 (C_{Ar} quat), 130.1 (q, $J_{C–F}$ = 33 Hz, $C_{Ar}CF_3$ quat), 127.8 (2 × C_{Ar}), 127.3 (2 × C_{Ar}), 126.8 (C_{Ar} quat), 126.0 (C_{Ar}), 125.9 (q, $J_{C–F}$ = 4 Hz, 2 × C_{Ar}), 124.0 (q, $J_{C–F}$ = 272 Hz, CF_3), 120.6 (C_{Ar}), 120.5 (C_{Ar}), 118.1 (*C*H_2=CH), 102.2 (C_{Ar}), 69.5 (CHOH), 64.5 (N^+CH), 63.0 (N^+CH_2Ar), 61.1 (N^+CH_2), 56.3 (OCH_3), 51.1 (N^+CH_2), 38.1 (CH_2=CH*C*H), 26.7 ($N^+(CH_2)_2$*C*H), 24.9 (N^+CH_2*C*H_2), 21.8 (N^+CH*C*H_2); ^{19}F{^{1}H} NMR (377 MHz, $CDCl_3$) δ –62.6; HRMS (FTMS + pAPCI) *m/z* Calculated for $C_{34}H_{34}N_2O_2F_3^+$ $[M–Br]^+$ 559.2567; Found 559.2572

SMILES:
C=C[C@H]1C[N@@+]2(CC3=CC=C(C4=CC=C(C(F)(F)F)C=C4)C=C3)[C@H]([C@@H](C5=C(C=C(OC)C=C6)C6=NC=C5)O)C[C@@H]1CC2.[Br-]
InChI=1S/C34H34F3N2O2.BrH/c1-3-23-21-39(20-22-4-6-24(7-5-22)25-8-10-27(11-9-25)34(35,36)37)17-15-26(23)18-32(39)33(40)29-14-16-38-31-13-12-28(41-2)19-30(29)31;/h3-14,16,19,23,26,32-33,40H,1,15,17-18,20-21H2,2H3;1H/q+1;/p-1/t23-,26-,32-,33+,39+;/m0./s1

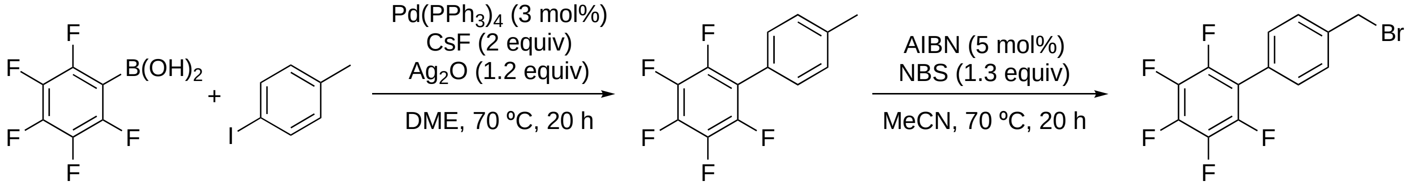

2,3,4,5,6-Pentafluoro-4'-methyl-1,1'-biphenyl (S69)[46]

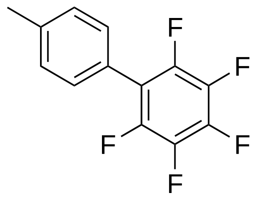

A mixture of 2,3,4,5,6-pentafluorobenzeneboronic acid (117 mg, 0.55 mmol), 4-iodotoluene (109 mg, 0.5 mmol), $Pd(PPh_3)_4$ (17 mg, 0.015 mmol), CsF (152 mg, 1.0 mmol), Ag_2O (139 mg, 0.6 mmol) in DME (1.5 mL) was degassed with Ar then heated at 70 ºC for 19 h. The reaction mixture was filtered through celite washing with EtOAc (20 mL) and concentrated under reduced pressure. Purification by flash chromatography (pentane) afforded 2,3,4,5,6-pentafluoro-4'-methyl-1,1'-biphenyl **S69** (118 mg, 91%) as a white solid. R_f 0.56 (hexane); mp = 120–121 ºC [lit = 119–120 ºC][47]; ν_{max} (film)/cm^{-1} 2926, 1484, 977, 772; ^{1}H NMR (400 MHz, $CDCl_3$) δ 7.32 (s, 4 H, 4 × HC_{Ar}), 2.43 (s, 3 H, $ArCH_3$); ^{13}C NMR (101 MHz, $CDCl_3$) δ 145.3 and 142.9 (2 × m, 2 × FC_{Ar}), 141.4 (m, FC_{Ar}), 139.4 (C_{Ar} quat), 139.0 and 136.5 (2 × m, 2 × FC_{Ar}), 130.0 (2 × C_{Ar}), 129.4 (2 × C_{Ar}), 123.3 (C_{Ar} quat), 115.9 (m, C_{Ar} quat), 21.3 ($ArCH_3$); ^{19}F NMR (377 MHz, $CDCl_3$) δ ^{19}F NMR –143.4 (dd, J = 23.0, 8.1 Hz, 2 × FC_{Ar}), –156.1 (t, J = 21.0 Hz, 2 × FC_{Ar}), –162.4 (td, J = 22.5, 8.1 Hz, FC_{Ar}). The observed characterisation data (IR, ^{1}H and ^{13}C NMR) was consistent with that previously reported in the literature.[46]

SMILES: CC1=CC=C(C2=C(F)C(F)=C(F)C(F)=C2F)C=C1
InChI=1S/C13H7F5/c1-6-2-4-7(5-3-6)8-9(14)11(16)13(18)12(17)10(8)15/h2-5H,1H3

4'-(Bromomethyl)-2,3,4,5,6-pentafluoro-1,1'-biphenyl (S70)

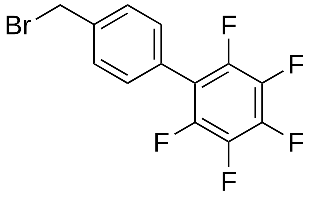

AIBN (0.8 mg, 5 µmol) was added to a solution of 2,3,4,5,6-pentafluoro-4'-methyl-1,1'-biphenyl **S69** (26 mg, 0.1 mmol) and NBS (23 mg, 0.13 mmol) in MeCN and heated to 70 ºC for 21 h. The reaction mixture was cooled to rt and filtered through celite, washing with pentane (20 mL) and concentrated under reduced pressure. Purification by flash chromatography (hexane) afforded 4'-(bromomethyl)-2,3,4,5,6-pentafluoro-1,1'-biphenyl **S70** (19 mg, 56%) as a white solid. R_f 0.42 (pentane); mp = 105–108 ºC; ν_{max} (film)/cm^{-1} 1491, 984; ^{1}H NMR (400 MHz, $CDCl_3$) δ 7.54 (d, J = 8.4 Hz, 2 H, 2 × HC_{Ar}), 7.44–7.40 (m, 2 H, 2 × HC_{Ar}), 4.55 (s, 2 H, CH_2Br); ^{13}C NMR (101 MHz, $CDCl_3$) δ 145.4 and 142.9 (2 × m, 2 × FC_{Ar}), 141.8 (m, FC_{Ar}), 139.1 and 136.6 (2 × m, 2 × FC_{Ar}), 138.9 (C_{Ar} quat), 130.6 (2 × C_{Ar}), 129.4 (2 × C_{Ar}), 126.4 (C_{Ar} quat), 115.2 (C_{Ar} quat), 32.5 (CH_2Br); ^{19}F{^{1}H} NMR (377 MHz, $CDCl_3$) δ –143.1 (dd, J = 22.9, 8.0 Hz, 2 × FC_{Ar}), –155.0 (t, J = 21.1 Hz, FC_{Ar}), –161.9 (td, J = 21.1, 7.9 Hz, 2 × FC_{Ar}); HRMS (FTMS + pAPCI) *m/z* Calculated for $C_{13}H_7{}^{79}BrF_5$ $[M+H]^+$ 336.9646; Found 336.9641.

SMILES: FC1=C(C2=CC=C(CBr)C=C2)C(F)=C(F)C(F)=C1F
InChI=1S/C13H6BrF5/c14-5-6-1-3-7(4-2-6)8-9(15)11(17)13(19)12(18)10(8)16/h1-4H,5H2

(1*S*,2*S*,4*S*,5*R*)-2-((*R*)-Hydroxy(6-methoxyquinolin-4-yl)methyl)-1-((2',3',4',5',6'-pentafluoro-[1,1'-biphenyl]-4-yl)methyl)-5-vinylquinuclidin-1-ium bromide (QN20)

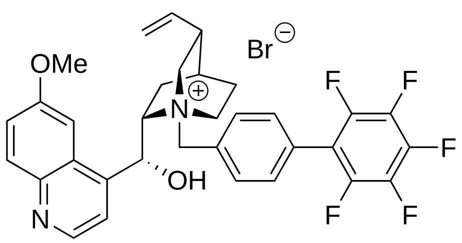

A mixture of quinine (48 mg, 0.15 mmol) and 4'-(bromomethyl)-2,3,4,5,6-pentafluoro-1,1'-biphenyl **S70** (50 mg, 0.15 mmol) in THF (0.75 mL) was heated to 70 ºC for 22 h. The reaction was cooled and Et_2O (2.5 mL) was added. The reaction was stirred for approx. 0.5 h then the precipitate was filtered. The collected solid was dried to afford (1*S*,2*S*,4*S*,5*R*)-2-((*R*)-hydroxy(6-methoxyquinolin-4-yl)methyl)-1-((2',3',4',5',6'-pentafluoro-[1,1'-biphenyl]-4-yl)methyl)-5-vinylquinuclidin-1-ium bromide **QN20** (72 mg, 73%) as a pale brown solid. $[\alpha]_D^{17} = -96º$ (*c* = 0.25, CH_2Cl_2); R_f 0.09 (10% MeOH/CH_2Cl_2); mp = 180–185 ºC (decomposition observed); ν_{max} (film)/cm^{-1} 3213 (br s, OH), 2982, 2948, 1621, 1510, 992; ^{1}H NMR (400 MHz, $CDCl_3$) δ 8.68 (d, *J* = 4.5 Hz, 1 H, HC_{Ar}), 7.98–7.93 (m, 3 H, 3 × HC_{Ar}), 7.71 (d, *J* = 4.5 Hz, 1 H, HC_{Ar}), 7.39 (d, *J* = 8.1 Hz, 2 H, 2 × HC_{Ar}), 7.32 (d, *J* = 2.5 Hz, 1 H, HC_{Ar}), 7.27 (dd, *J* = 9.2, 2.5 Hz, 2 H, 2 × HC_{Ar}), 6.69 (d, *J* = 6.2 Hz, 1 H, N^+CH), 6.42 (d, *J* = 6.8 Hz, 1 H, OH), 6.20 (d, *J* = 12.0 Hz, 1 H, N^+C*H*HAr), 5.59 (ddd, *J* = 17.2, 10.4, 6.9 Hz, 1 H, CH_2=C*H*), 5.13 (d, *J* = 16.9 Hz, 1 H, C*H*H=CH), 5.00–4.90 (t, *J* = 12.0 Hz, 3 H, N^+CH*H*Ar, N^+C*H*H and CH*H*=CH), 3.99–3.93 (m, 4 H, C*H*OH and OCH_3), 3.65 (ddd, *J* = 12.9, 5.8, 2.5 Hz, 1 H, N^+C*H*H), 3.48 (dd, *J* = 12.6, 10.7, 1 H, N^+CH*H*), 3.11 (td, *J* = 11.4, 6.4 Hz, 1 H, N^+CH*H*), 2.64–2.55 (m, 1 H, CH_2=CHC*H*), 2.37–2.23 (m, 2 H, N^+CHC*H*H and N^+CH_2C*H*H), 2.06–2.00 (m, 1 H, $N(CH_2)_2$C*H*), 1.82–1.72 (m, 1 H, NCH_2CH*H*), 1.58–1.48 (m, 1 H, NCHCH*H*); ^{13}C NMR (101 MHz, $CDCl_3$) δ 158.0 (OC_{Ar} quat), 147.2 (C_{Ar}), 145.2 and 142.8 (2 × m, 2 × FC_{Ar}), 144.1 (C_{Ar} quat), 143.0 (C_{Ar} quat), 139.1 (m, 2 × FC_{Ar}), 136.6 (m, FC_{Ar}), 136.1 (CH_2=*C*H), 134.3 (2 × C_{Ar}), 131.7 (C_{Ar}), 130.7 (2 × C_{Ar}), 128.6 (C_{Ar} quat), 128.3 (C_{Ar} quat), 125.9 (C_{Ar} quat), 121.1 (C_{Ar}), 120.5 (C_{Ar}), 117.9 (*C*H_2=CH), 114.5 (m, C_{Ar} quat), 101.8 (C_{Ar}), 69.5 (CHOH), 63.9 (N^+CH), 62.3 (N^+CH_2Ar), 60.4 (N^+CH_2), 56.3 (OCH_3), 51.2 (N^+CH_2), 37.9 (CH_2=CH*C*H), 26.8 ($N(CH_2)_2$*C*H), 24.8 (NCH_2*C*H_2), 21.5 (NCH*C*H_2); ^{19}F{^{1}H} NMR (377 MHz, $CDCl_3$) δ –143.1 (dd, *J* = 22.8, 8.2 Hz, 2 × FC_{Ar}), –153.8 (t, *J* = 20.9 Hz, FC_{Ar}), –161.3 (td, *J* = 22.0, 7.9 Hz, 2 × FC_{Ar}); HRMS (FTMS + pAPCI) *m/z* Calculated for $C_{33}H_{30}N_2O_2F_5^+$ $[M–Br]^+$ 581.2222; Found 581.2214.

SMILES:
C=C[C@H]1C[N@@+]2(CC3=CC=C(C4=C(F)C(F)=C(F)C(F)=C4F)C=C3)[C@H]([C@@H](C5=C(C=C(OC)C=C6)C6=NC=C5)O)C[C@@H]1CC2.[Br-]
InChI=1S/C33H30F5N2O2.BrH/c1-3-19-17-40(16-18-4-6-20(7-5-18)27-28(34)30(36)32(38)31(37)29(27)35)13-11-21(19)14-26(40)33(41)23-10-12-39-25-9-8-22(42-2)15-24(23)25;/h3-10,12,15,19,21,26,33,41H,1,11,13-14,16-17H2,2H3;1H/q+1;/p-1/t19-,21-,26-,33+,40+;/m0./s1

(1*S*,2*S*,4*S*,5*R*)-2-((*R*)-(6-Methoxyquinolin-4-yl)((4-(trifluoromethyl)benzyl)oxy)methyl)-5-vinylquinuclidine (S71)

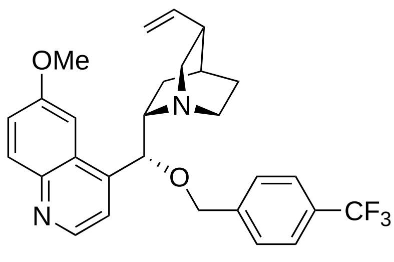

Cesium hydroxide monohydrate (84 mg, 0.5 mmol) was added to a solution of 1*S*,2*S*,4*S*,5*R*)-2-((*R*)-hydroxy(6-methoxyquinolin-4-yl)methyl)-1-(4-(trifluoromethyl)benzyl)-5-vinylquinuclidin-1-ium bromide **QN4** (56 mg, 0.1 mmol) in toluene (1.0 mL) and the mixture was stirred at 25 ºC for 17 h. The reaction mixture was filtered through celite, washing with Et_2O (25 mL) and concentrated under reduced pressure to afford (1*S*,2*S*,4*S*,5*R*)-2-((*R*)-(6-methoxyquinolin-4-yl)((4-(trifluoromethyl)benzyl)oxy)methyl)-5-vinylquinuclidine **S71** (46 mg, 96%) as a colourless gummy solid. $[\alpha]_D^{17} = -29º$ (*c* = 1.18, $CHCl_3$); R_f 0.50 (Et_2O); ν_{max} (film)/cm^{-1} 2934, 2795, 2762, 1620, 1508, 1324, 1238, 1161, 1121, 1105, 1066, 1019, 854; ^{1}H NMR (400 MHz, $CDCl_3$) δ 8.75 (d, *J* = 4.4 Hz, 1 H, HC_{Ar}), 8.06 (d, *J* = 9.2 Hz, 1 H, HC_{Ar}), 7.56 (d, *J* = 8.1 Hz, 2 H, 2 × HC_{Ar}), 7.46–7.39 (m, 3 H, 3 × HC_{Ar}), 7.27 (dd, *J* = 15.9, 3.5 Hz, 2 H, 2 × HC_{Ar}), 6.32–6.19 (m, 1 H, CH_2=C*H*), 5.14 (dd, *J* = 10.3, 1.9 Hz, 1 H, C*H*H=CH), 5.10–5.03 (m, 1 H, CH*H*=CH), 4.14 (d, *J* = 1.7 Hz, 1 H, C*H*OCH_2Ar), 3.97 (s, 3 H, OCH_3), 3.56 (d, *J* = 14.0 Hz, 1 H, OC*H*HAr), 3.48 (d, *J* = 14.0 Hz, 1 H, OCH*H*Ar), 3.04–2.97 (m, 1 H, NC*H*H), 2.86 (d, *J* = 10.7 Hz, 1 H, NCH), 2.75 (d, *J* = 10.0 Hz, 1 H, NC*H*H), 2.46 (dd, *J* = 8.7, 3.1 Hz, 1 H, CH_2=CHC*H*), 2.34–2.24 (m, 1 H, NCH*H*), 2.15 (td, *J* = 10.5, 3.7 Hz,

1 H, NCH*H*), 1.99–1.87 (m, 2 H, NCHC*H*H and NCH_2C*H*H), 1.78–1.64 (m, 3 H, N(CH_2)C*H*, NCHCH*H* and NCH_2CH*H*); ^{13}C NMR (101 MHz, $CDCl_3$) δ 157.9 (OC_{Ar} quat), 148.0 (C_{Ar}), 143.9 (C_{Ar} quat), 143.2 (C_{Ar} quat), 141.8 (C_{Ar}), 138.1 (CH_2=*C*H), 131.8 (C_{Ar}), 129.1 (q, $J_{C–F}$ = 32 Hz, $C_{Ar}CF_3$ quat), 128.8 (2 × C_{Ar}), 127.4 (C_{Ar} quat), 124.3 (q, $J_{C–F}$ = 273 Hz, CF_3) 125.1 (q, $J_{C–F}$ = 4 Hz, 2 × C_{Ar}), 121.6 (C_{Ar}), 116.8 (C_{Ar}), 116.5 (*C*H_2=CH), 101.0 (C_{Ar}), 62.6 (OCH_2Ar), 61.3 (NCH), 58.9 (NCH_2), 55.6 (OCH_3), 55.4 (*C*HOCH_2Ar), 53.7 (NCH_2), 43.4 (NCH_2) 36.9 (CH_2=CH*C*H), 35.9 (N(CH_2)$_2$*C*H), 28.7 (NCH*C*H_2 and NCH_2*C*H_2); ^{19}F{^{1}H} NMR (377 MHz, $CDCl_3$) δ –62.3; HRMS (ESI$^+$) *m/z* Calculated for $C_{28}H_{30}N_2O_2F_3$ [M+H] 483.2259; Found 483.2245.

SMILES:
C=C[C@H]1C[N@]2[C@H]([C@@H](C3=C(C=C(OC)C=C4)C4=NC=C3)OCC5=CC=C(C(F)(F)F)C=C5)C[C@@H]1CC2
InChI=1S/C28H29F3N2O2/c1-3-19-16-33-13-11-20(19)14-26(33)27(35-17-18-4-6-21(7-5-18)28(29,30)31)23-10-12-32-25-9-8-22(34-2)15-24(23)25/h3-10,12,15,19-20,26-27H,1,11,13-14,16-17H2,2H3/t19-,20-,26-,27+/m0/s1

(4-(Bromomethyl)phenyl)pentafluoro-λ^6-sulfane (S72)

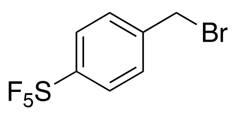

S72 could be synthesised using a method by Spokoyny,[48] however, in our hands only ~80% purity could be achieved, which could to be taken to the next step. After initial tests **S72** was purchased from Fluorochem UK. Method: A solution of AIBN (6.6 mg, 0.04 mmol), *N*-bromo succinimide (89 mg, 0.5 mmol) and pentafluoro(*p*-tolyl)-λ^6-sulfane (77 μL, 0.5 mmol) in 1,2-DCE (2.5 mL) was degassed with Ar in a sealed tube. The solution was stirred at 80 ºC for 27 h. The reaction mixture was cooled to rt, concentrated under reduced pressure then filtered through celite with Et_2O (10 mL) and concentrated under reduced pressure again. Purification by flash chromatography (pentane) afforded (4-(bromomethyl)phenyl)pentafluoro-λ^6-sulfane **S77** (100 mg, 67%) as a white solid in ~80% purity which was used in the next step.

(1*S*,2*S*,4*S*,5*R*)-2-((*R*)-Hydroxy(6-methoxyquinolin-4-yl)methyl)-1-(4-(pentafluoro-λ^6-sulfaneyl)benzyl)-5-vinylquinuclidin-1-ium bromide (QN21)

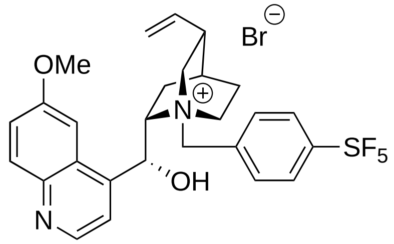

A solution of quinine (65 mg, 0.20 mmol) and (4-(bromomethyl)phenyl)pentafluoro-λ^6-sulfane **S72** (59 mg, 0.20 mmol) in THF (1.0 mL) was heated to 70 ºC for 15 h. The reaction was cooled to rt and Et_2O was added. The reaction was stirred for approx. 0.5 h then the precipitate was filtered. The collected solid was dried to afford (1*S*,2*S*,4*S*,5*R*)-2-((*R*)-hydroxy(6-methoxyquinolin-4-yl)methyl)-1-(4-(pentafluoro-λ^6-sulfaneyl)benzyl)-5-vinylquinuclidin-1-ium bromide **QN21** (93 mg, 75%) as a white solid. $[\alpha]_D^{22}$ = –120 (*c* = 0.25, CH_2Cl_2); R_f (10% MeOH/CH_2Cl_2); mp = 190–192 ºC; ν_{max} (film)/cm^{-1} 3216 (br s, OH), 2962, 1621, 1510, 1472, 1241, 846; ^{1}H NMR (400 MHz, $CDCl_3$) δ 8.69 (d, *J* = 4.5 Hz, HC_{Ar}), 8.02 (d, *J* = 8.4 Hz, 2 × HC_{Ar}), 7.93 (d, *J* = 9.0 Hz, 1 H, HC_{Ar}), 7.91–7.66 (m, 3 H, 3 × HC_{Ar}), 7.27–7.22 (m, 2 H, 2 × HC_{Ar}), 6.65–6.60 (m, 1 H, N$^+$CH), 6.31 (br d, *J* = 5.5 Hz, OH), 6.06 (d, *J* = 11.9 Hz, N$^+$C*H*HAr), 5.52 (ddd, *J* = 17.2, 10.5, 6.8 Hz, C*H*=CH_2), 5.14–5.03 (m, 2 H, CH=C*H*H and N$^+$CH*H*Ar), 4.87–4.77 (m, 2 H, CH=CH*H* and N$^+$C*H*H), 4.06–3.90 (m, 5 H, C*H*OH, OCH_3 and N$^+$C*H*H), 3.29 (dd, *J* = 11.1, 1.5 Hz, N$^+$CH*H*), 3.02–2.92 (m, 1 H, N$^+$CH*H*), 2.56–2.47 (m, 1 H, NCH_2C*H*), 2.33–2.13 (m, 2 H, NCH_2C*H*H and NCHC*H*H), 2.02–1.97 (m, 1 H, N(CH_2)$_2$C*H*), 1.78–1.69 (m, 1 H, NCHCH*H*), 1.55–1.45 (m, 1 H, NCH_2CH*H*); ^{13}C NMR (101 MHz, $CDCl_3$) δ 158.1 (OC_{Ar} quat), 155.1 (p, $J_{C–F}$ = 18.0 Hz, SF_5C_{Ar} quat), 147.2 (C_{Ar}), 143.9 (C_{Ar} quat), 143.0 (C_{Ar} quat), 135.9 (*C*H=CH_2), 134.5 (2 × C_{Ar}), 131.7 (C_{Ar} quat), 131.1 (C_{Ar} quat), 126.6 (br s, 2 × C_{Ar}), 125.9 (C_{Ar}), 121.2 (C_{Ar}), 120.5 (C_{Ar}), 118.0 (CH=*C*H_2), 101.9 (C_{Ar}), 69.6 (*C*HOH), 63.9 (N$^+$CH), 61.4 (N$^+$$CH_2$Ar), 60.5 (N$^+$$CH_2$), 56.4 ($OCH_3$), 51.4 (N$^+$$CH_2$), 37.8 (*C*HCH=$CH_2$), 26.7 (N($CH_2$)$_2$*C*H), 24.8 (NCH*C*$H_2$), 21.5 ($NCH_2$*C*$H_2$); ^{19}F{^{1}H} NMR (377 MHz, $CDCl_3$) δ 83.0 (q, $J_{F–F}$ = 151 Hz, F_{ax}), 62.5 (d, $J_{F–F}$ = 150 Hz, 4 × F_{eq}); HRMS (ESI$^+$) *m/z* Calculated for $C_{27}H_{30}N_2O_2SF_5$ [M–Br] 541.1948; Found 541.1941.

SMILES:
C=C[C@H]1C[N@@+]2(CC3=CC=C(S(F)(F)(F)(F)F)C=C3)[C@H]([C@@H](C4=C(C=C(OC)C=C5)C5=NC=C4)O)C[C@@H]1CC2.[Br-]
InChI = 1S/C27H30F5N2O2S.BrH/c1-3-19-17-34(16-18-4-7-22(8-5-18)37(28,29,30,31)32)13-11-20(19)14-26(34)27(35)23-10-12-33-25-9-6-21(36-2)15-24(23)25;/h3-10,12,15,19-20,26-27,35H,1,11,13-14,16-17H2,2H3;1H/q+1;/p-1/t19-,20-,26-,27+,34+;/m0./s1

(1*S*,2*S*,4*S*,5*R*)-2-((*R*)-Hydroxy(6-methoxyquinolin-4-yl)methyl)-1-(4-nitrobenzyl)-5-vinylquinuclidin-1-ium bromide (QN10)

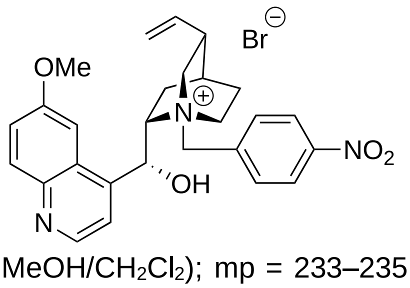

A solution of quinine (162 mg, 0.5 mmol) and 1-(bromomethyl)-4-nitrobenzene (108 mg, 0.5 mmol) in THF (2.5 mL) was heated to 70 ºC for 16 h. The reaction was cooled to rt and Et_2O was added. The reaction was stirred for approx. 0.5 h then the precipitate was filtered. The collected solid was dried to afford (1*S*,2*S*,4*S*,5*R*)-2-((*R*)-hydroxy(6-methoxyquinolin-4-yl)methyl)-1-(4-nitrobenzyl)-5-vinylquinuclidin-1-ium bromide **QN10** (93 mg, 75%) as an off-white solid. $[\alpha]_D^{21}$ = –156 (*c* = 0.5, CH_2Cl_2); R_f 0.23 (10% MeOH/CH_2Cl_2); mp = 233–235 ºC (decomposition observed); ν_{max} (film)/cm^{-1} 3176 (br s, OH), 2982, 1621, 1521, 1472, 1431, 1346, 1226, 1029, 921, 857, 824, 701; ^{1}H NMR (400 MHz, $CDCl_3$) δ 8.70 (d, *J* = 4.5 Hz, 1 H, HC_{Ar}), 8.11–8.05 (m, 4 H, 4 × HC_{Ar}), 7.94–7.91 (m, 1 H, HC_{Ar}), 7.73 (d, *J* = 4.5 Hz, 1 H, HC_{Ar}), 7.26–7.22 (m, 2 H, HC_{Ar}), 6.59 (d, *J* = 6.4 Hz, 1 H, N^+CH), 6.36 (d, *J* = 6.6 Hz, 1 H, OH), 6.13 (d, *J* = 12.0 Hz, 1 H, N^+C*H*HAr), 5.56 (ddd, *J* = 17.1, 10.5, 6.6 Hz, 1 H, CH_2=C*H*), 5.32 (d, *J* = 12.0 Hz, 1 H, N^+CH*H*Ar), 5.20–5.13 (m, 1 H, C*H*H=CH), 4.95 (d, *J* = 10.2 Hz, 1 H, CH*H*=CH), 4.93–4.82 (m, 1 H, N^+C*H*H), 4.04 (t, *J* = 8.9 Hz, 1 H, C*H*OH), 3.95–4.85 (m, 4 H, OCH_3 and N^+C*H*H), 3.36 (dd, *J* = 11.1, 1.40 Hz, 1 H, N^+CH*H*), 3.04 (td, *J* = 11.5, 6.0 Hz, 1 H, N^+CH*H*), 2.62–2.54 (m, 1 H, CH_2=CHC*H*), 2.32–2.16 (m, 2 H, NCHC*H*H and NCH_2C*H*H), 2.05–2.00 (m, 1 H, $N(CH_2)_2$C*H*), 1.81–1.70 (m, 1 H, NCHCH*H*), 1.53–1.43 (m, 1 H, NCH_2CH*H*); ^{13}C NMR (101 MHz, $CDCl_3$) δ 158.1 (OC_{Ar} quat), 148.7 (C_{Ar} quat), 147.1 (C_{Ar}), 143.8 (C_{Ar} quat), 142.9 (C_{Ar} quat), 135.9 (*C*H=CH_2), 135.2 (2 × C_{Ar}), 134.2 (C_{Ar} quat), 131.7 (C_{Ar} quat), 125.9 (C_{Ar}), 123.7 (2 × C_{Ar}), 120.8 (C_{Ar}), 120.6 (C_{Ar}), 118.1 (CH=*C*H_2), 102.2 (C_{Ar}), 69.3 (CHOH), 64.4 (N^+CH), 61.2 (N^+CH_2Ar), 60.7 (N^+CH_2), 56.4 (OCH_3), 51.6 (N^+CH_2), 37.8 (*C*HCH=CH_2), 26.5 ($N(CH_2)_2$*C*H), 24.9 (NCH*C*H_2), 21.8 (NCH_2*C*H_2); HRMS (ESI$^+$) *m/z* Calculated for $C_{27}H_{30}N_3O_4^+$ [M–Br$^-$] 460.2231; Found 460.2221.

SMILES:C=C[C@H]1C[N@@+]2(CC3=CC=C([N+]([O-
])=O)C=C3)[C@H]([C@H](O)C4=C(C=C(OC)C=C5)C5=NC=C4)C[C@@H]1CC2.[Br-]
InChI=1S/C27H29N3O4.BrH/c1-3-19-17-30(16-18-4-6-21(7-5-18)29(32)33)13-11-20(19)14-26(30)27(31)23-10-12-28-25-9-8-22(34-2)15-24(23)25;/h3-9,12,15,19-20,26-27,31H,1,11,13-14,16-17H2,2H3;1H/q+1;/p-1/t19?,20?,26?,27-;/m1./s1

(*R*)-(2-Butyl-6-methoxyquinolin-4-yl)((1*S*,2*S*,4*S*,5*R*)-5-vinylquinuclidin-2-yl)methanol (S73)[49]

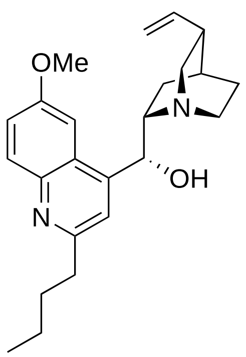

n-BuLi (1.54 M in hexanes, 3.90 mL, 6.0 mmol) was added to quinine (649 mg, 2.0 mmol) suspended in MTBE (10 mL) at –10 ºC. After 20 min at –10 ºC, the reaction mixture was stirred at 25 ºC for 1 h. AcOH (0.9 mL) was added, followed by H_2O (10 mL) and EtOAc (10 mL). I_2 (<0.5 g) was added to the stirred solution until a dark colour persisted. Then sat. aq. $Na_2S_2O_3$ (10 mL) was added followed by aq. NH_3 (~20%, 5 mL). The layers were separated and the organic layer was washed with CH_2Cl_2 (2 × 30 mL). The combined organic layers were dried over Na_2SO_4, filtered and concentrated under reduced pressure. Purification by flash chromatography (10:1:1 toluene:MeOH:NEt_3) afforded (*R*)-(2-butyl-6-methoxyquinolin-4-yl)((1*S*,2*S*,4*S*,5*R*)-5-vinylquinuclidin-2-yl)methanol **S73** (381 mg, 50%) as a white solid. $[\alpha]_D^{17}$ = –60º (*c* = 0.5, CH_2Cl_2); R_f 0.31 (10:1:1 toluene:MeOH:NEt_3); mp = 57–61 ºC; ν_{max} (film)/cm^{-1} 3400–2500 (br s, OH), 2922, 2855, 1618, 1561, 1502, 1450, 1349, 1259, 1230, 1163, 1096, 1029, 992, 939, 906, 828, 667; ^{1}H NMR (400 MHz, $CDCl_3$) δ 7.88 (d, *J* = 9.2 Hz, 1 H, HC_{Ar}), 7.39 (s, 1 H, HC_{Ar}), 7.24 (dd, *J* = 9.2, 2.7 Hz, 1 H, HC_{Ar}), 7.14 (d, *J* =

2.7 Hz, 1 H, HC_{Ar}), 5.70 (ddd, *J* = 17.9, 10.3, 7.8 Hz, 1 H, C*H*=CH_2), 5.49 (d, *J* = 3.3 Hz, 1 H, CHOH), 4.97–4.87 (m, 2 H, CH=C*H₂*), 3.86 (s, 3 H, OCH_3), 3.57–3.47 (m, 1 H, NC*H*H), 3.09–3.01 (m, 2 H, NCH and NC*H*H), 2.74 (dd, *J* = 8.9, 6.8 Hz, 2 H, $ArCH_2$), 2.69–2.59 (m, 2 H, 2 × NCH*H*), 2.28–2.20 (m, 1 H, C*H*CH=CH_2), 1.81–1.67 (m, 3 H, $N(CH_2)_2$C*H*, NCHC*H*H and NCH_2C*H*H), 1.66–1.56 (m, 2 H, $ArCH_2$CH_2), 1.52–1.38 (m, 2 H, NCHCH*H* and NCH_2CH*H*), 1.27 (h, *J* = 7.3 Hz, 2 H, $Ar(CH_2)_2$CH_2), 0.84 (t, *J* = 7.3 Hz, 3 H, $Ar(CH_2)_3$CH_2); ^{13}C NMR (101 MHz, $CDCl_3$) δ 160.0 ($CH_2$$C_{Ar}$ quat), 157.0 (OC_{Ar} quat), 148.0 (C_{Ar} quat), 143.7 (C_{Ar} quat), 141.9 (*C*H=CH_2), 130.6 (C_{Ar}), 124.8 (C_{Ar} quat), 120.9 (C_{Ar}), 118.5 (C_{Ar}), 114.3 (CH=*C*H_2), 101.4 (C_{Ar}), 71.9 (*C*HOH), 59.9 (NCH), 57.1 (NCH_2), 55.6 (OCH_3), 43.3 (NCH_2), 40.0 (*C*HCH=CH_2), 38.7 ($ArCH_2$), 32.1 ($ArCH_2$*C*H_2), 27.9 ($N(CH_2)_2CH$), 27.6 (NCH*C*H_2), 22.6 ($Ar(CH_2)_2CH_2$), 21.3 (NCH_2*C*H_2), 13.9 ($Ar(CH_2)_3CH_2$). The observed characterisation data (^{1}H and ^{13}C NMR) was consistent with that previously reported in the literature.[49]

SMILES:
C=C[C@H]1C[N@]2[C@H]([C@@H](C3=C(C=C(OC)C=C4)C4=NC(CCCC)=C3)O)C[C@@H]1CC2
InChI = 1S/C24H32N2O2/c1-4-6-7-18-13-21(20-14-19(28-3)8-9-22(20)25-18)24(27)23-12-17-10-11-26(23)15-16(17)5-2/h5,8-9,13-14,16-17,23-24,27H,2,4,6-7,10-12,15H2,1,3H3/t16-,17-,23-,24+/m0/s1

(1*S*,2*S*,4*S*,5*R*)-2-((*R*)-(2-Butyl-6-methoxyquinolin-4-yl)(hydroxy)methyl)-1-(4-(trifluoromethyl)benzyl)-5-vinylquinuclidin-1-ium bromide (QN4G) (Cat5)

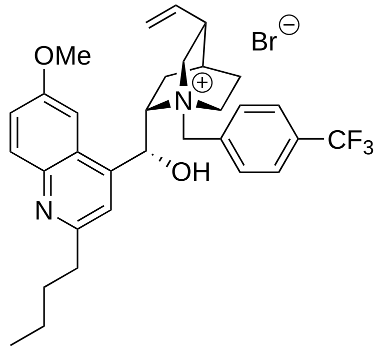

A solution of **S73** (95 mg, 0.25 mmol) and 4-(trifluromethyl)benzyl bromide (60 mg, 0.25 mmol) in THF (1.25 mL) was heated to 70 ºC for 23 h. The reaction was cooled to rt and Et_2O was added. The reaction was stirred for approx. 0.5 h then the precipitate was filtered. The collected solid was dried to afford (1*S*,2*S*,4*S*,5*R*)-2-((*R*)-(2-butyl-6-methoxyquinolin-4-yl)(hydroxy)methyl)-1-(4-(trifluoromethyl)benzyl)-5-vinylquinuclidin-1-ium bromide **QN4G** (65 mg, 42%) as an off-white crystalline solid. $[\alpha]_D^{17}$ = –112º (*c* = 0.25, $CHCl_3$); mp = 57–61 ºC; R_f 0.44 (10% MeOH/CH_2Cl_2); ν_{max} (film)/cm^{-1} 3213 (br s, OH), 2956, 1621, 1506, 1323, 1230, 1170, 1129, 1066, 1021, 857, 828, 731; ^{1}H NMR (400 MHz, $CDCl_3$) δ 8.02 (d, *J* = 8.0 Hz, 2 H, 2 × HC_{Ar}), 7.95 (d, *J* = 9.2 Hz, 1 H, HC_{Ar}), 7.69 (s, 1 H, HC_{Ar}), 7.61 (d, *J* = 8.0 Hz, 2 H, 2 × HC_{Ar}), 7.28–7.24 (m, 1 H, HC_{Ar}), 7.17 (d, *J* = 2.6 Hz, 1 H, HC_{Ar}), 6.65 (d, *J* = 6.6 Hz, 1 H, N^+CH), 6.38 (d, *J* = 6.8 Hz, 1 H, OH), 6.27 (d, *J* = 12.0 Hz, 1 H, N^+C*H*HAr), 5.55 (ddd, *J* = 17.2, 10.4, 6.9 Hz, 1 H, C*H*=CH_2), 5.12 (d, *J* = 17.6, 0.7 Hz, 1 H, CH=C*H*H), 5.02–4.92 (m, 3 H, N^+CH*H*Ar, CH=CH*H* and N^+C*H*H), 3.93–3.84 (m, 4 H, OCH_3 and OCH), 3.61–3.53 (m, 1 H, N^+C*H*H), 3.46–3.38 (m, 1 H, N^+CH*H*), 3.06 (td, *J* = 11.4, 6.2 Hz, 1 H, N^+CH*H*), 2.90 (dd, *J* = 8.8, 7.0 Hz, 2 H, $ArCH_2$), 2.62–2.53 (m, 1 H, C*H*CH=CH_2), 2.39–2.29 (m, 1 H, NCHC*H*H), 2.28–2.19 (m, 1 H, NCH_2C*H*H), 2.06–2.01 (m, 1 H, $N(CH_2)_2$C*H*), 1.81–1.71 (m, 3 H, $ArCH_2$C*H₂* and NCHCH*H*), 1.51–1.37 (m, 3 H, $Ar(CH_2)_2$C*H₂* and NCH_2CH*H*), 0.96 (t, *J* = 7.4 Hz, 3 H, $Ar(CH_2)_3$C*H₃*); ^{13}C NMR (101 MHz, $CDCl_3$) δ 160.3 (CH_2*C*$_{Ar}$ quat), 157.4 (OC_{Ar} quat), 143.9 (C_{Ar} quat), 142.8 (C_{Ar} quat), 136.0 (*C*H=CH_2), 134.4 (C_{Ar}), 132.6 (q, J_{C-F} = 32.9 Hz, *C*$_{Ar}$$CF_3$ quat), 131.4 (C_{Ar}), 130.9 (C_{Ar} quat), 126.0 (q, J_{C-F} = 3.1 Hz, 2 × C_{Ar}), 124.1 (C_{Ar}), 123.4 (q, J_{C-F} = 272.6 Hz, CF_3 quat), 120.9 (C_{Ar}), 120.1 (C_{Ar}), 118.2 (CH=*C*H_2), 102.2 (C_{Ar}), 69.8 (CHOH), 64.0 (N^+CH), 62.3 (N^+CH_2Ar), 61.1 (N^+CH_2), 56.2 (OCH_3), 51.4 (N^+CH_2), 38.8 ($ArCH_2$), 38.0 (*C*HCH=CH_2), 32.2 ($ArCH_2$*C*H_2), 26.6 ($N(CH_2)_2$C*H*), 24.8 (NCH*C*H_2), 22.7 ($Ar(CH_2)_2$*C*H_2), 21.6 (NCH_2*C*H_2), 14.0 ($Ar(CH_2)_3$*C*H_3); ^{19}F NMR (377 MHz, $CDCl_3$) δ –63.1; HRMS (ESI$^+$) *m/z* Calculated for $C_{32}H_{38}N_2O_2F_3$ [M–Br] 539.2885; Found 539.2881.

SMILES:
C=C[C@H]1C[N@@+]2(CC3=CC=C(C(F)(F)F)C=C3)[C@H]([C@@H](C4=C(C=C(OC)C=C5)C5=NC(CCCC)=C4)O)C[C@@H]1CC2.[Br-]
InChI = 1S/C32H38F3N2O2.BrH/c1-4-6-7-25-17-28(27-18-26(39-3)12-13-29(27)36-25)31(38)30-16-23-14-15-37(30,20-22(23)5-2)19-21-8-10-24(11-9-21)32(33,34)35;/h5,8-13,17-18,22-23,30-31,38H,2,4,6-7,14-16,19-20H2,1,3H3;1H/q+1;/p-1/t22-,23-,30-,31+,37+;/m0./s1

(1*S*,2*S*,4*S*,5*R*)-1-Benzyl-2-((*R*)-hydroxy(6-methoxyquinolin-4-yl)methyl)-5-vinylquinuclidin-1-ium bromide (QN1)

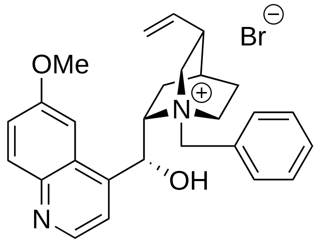

A solution of quinine (162 mg, 0.5 mmol) and benzyl bromide (60 μL, 0.5 mmol) in THF (2.5 mL) was heated to 70 ºC for 18 h. The reaction was cooled to rt and Et_2O was added. The reaction was stirred for approx. 0.5 h then the precipitate was filtered. The collected solid was dried to afford (1*S*,2*S*,4*S*,5*R*)-1-benzyl-2-((*R*)-hydroxy(6-methoxyquinolin-4-yl)methyl)-5-vinylquinuclidin-1-ium bromide **QN1** (226 mg, 91%) as a brown solid. $[\alpha]_D^{22}$ = –173 (*c* = 0.3, MeOH) [lit = –205 (*c* = 0.51, MeOH)][50]; R_f 0.15 (10% MeOH/CH_2Cl_2); mp = 165–168 ºC [lit = 168–170 ºC][51]; ν_{max} (film)/cm^{-1} 3187 (br s, OH), 2945, 1618, 1510, 1457, 1361, 1323, 1226, 1029, 913, 828, 764, 708; ^{1}H NMR (400 MHz, $CDCl_3$) δ 8.63 (d, *J* = 4.6 Hz, 1 H, HC_{Ar}), 7.91 (d, *J* = 9.2 Hz, 1 H, HC_{Ar}), 7.76 (d, *J* = 7.1 Hz, 2 H, 2 × HC_{Ar}), 7.65 (d, *J* = 4.6 Hz, 1 H, HC_{Ar}), 7.41 – 7.20 (m, 5 H, 5 × HC_{Ar}), 6.63 (d, *J* = 4.1 Hz, 1 H, N^+CH), 6.42 (d, *J* = 6.1 Hz, 1 H, OH), 5.89 (d, *J* = 12.0 Hz, 1 H, $N^+C\mathit{H}H$), 5.58 (ddd, *J* = 17.3, 10.4, 6.9 Hz, 1 H, $C\mathit{H}=CH_2$), 5.07 (d, *J* = 17.1 Hz, 1 H, $CH=C\mathit{H}H$), 4.92 (d, *J* = 10.4 Hz, 1 H, $CH=CH\mathit{H}$), 4.87–4.72 (m, 2 H, $N^+CH\mathit{H}Ar$ and $N^+C\mathit{H}H$), 4.00–3.92 (m, 4 H, $C\mathit{H}OH$ and OCH_3), 3.74–3.66 (m, 1 H, $N^+C\mathit{H}H$), 3.44–3.37 (m, 1 H, $N^+CH\mathit{H}$), 3.09–3.00 (m, 1 H, $N^+CH\mathit{H}$), 2.55 (q, *J* = 7.0 Hz, 1 H, $C\mathit{H}CH=CH_2$), 2.33–2.17 (m, 2 H, $NCHC\mathit{H}H$ and $NCH_2C\mathit{H}H$), 1.98–1.93 (m, 1 H, $N(CH_2)_2C\mathit{H}$), 1.75–1.65 (m, 1 H, $NCH_2CH\mathit{H}$), 1.57–1.47 (m, 1 H, $NCHCH\mathit{H}$); ^{13}C NMR (101 MHz, $CDCl_3$) δ 158.0 (OC_{Ar} quat), 147.0 (C_{Ar}), 143.63 (C_{Ar} quat), 143.59 (C_{Ar} quat), 136.3 ($\mathit{C}H=CH_2$), 133.8 (2 × C_{Ar}), 131.3 (C_{Ar}), 130.4 (C_{Ar}), 129.1 (2 × C_{Ar}), 126.9 (C_{Ar} quat), 126.0 (C_{Ar} quat), 121.3 (C_{Ar}), 120.4 (C_{Ar}), 117.8 ($CH=\mathit{C}H_2$), 102.0 (C_{Ar}), 69.3 (CHOH), 63.9 (N^+CH), 63.4 (N^+CH_2Ar), 60.4 (N^+CH_2), 56.3 (OCH_3), 51.1 (N^+CH_2), 38.0 ($\mathit{C}HCH=CH_2$), 26.8 ($N(CH_2)_2\mathit{C}H$), 24.7 ($NCH\mathit{C}H_2$), 21.4 ($NCH_2\mathit{C}H_2$). The observed characterisation data (^{1}H and ^{13}C NMR) was consistent with that previously reported in the literature.[51]

SMILES:
C=C[C@H]1C[N@@+]2(CC3=CC=CC=C3)[C@H]([C@@H](C4=C(C=C(OC)C=C5)C5=NC=C4)O)C[C@@H]1CC2.[Br-]
InChI=1S/C27H31N2O2.BrH/c1-3-20-18-29(17-19-7-5-4-6-8-19)14-12-21(20)15-26(29)27(30)23-11-13-28-25-10-9-22(31-2)16-24(23)25;/h3-11,13,16,20-21,26-27,30H,1,12,14-15,17-18H2,2H3;1H/q+1;/p-1/t20-,21-,26-,27+,29+;/m0./s1

(1*S*,2*S*,4*S*,5*R*)-2-((*R*)-Hydroxy(quinolin-4-yl)methyl)-1-(4-(trifluoromethyl)benzyl)-5-vinylquinuclidin-1-ium bromide (CD3) (Cat6)

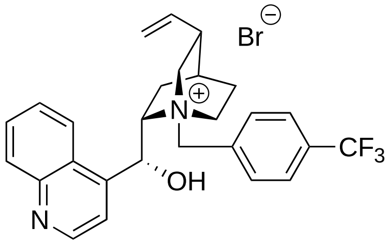

A solution of cinchonidine (147 mg, 0.5 mmol) and 4-(trifluromethyl)benzyl bromide (120 mg, 0.5 mmol) in THF (2.5 mL) was heated to 70 ºC for 23 h. The reaction was cooled to rt and Et_2O was added. The reaction was stirred for approx. 0.5 h then the precipitate was filtered. The collected solid was dried to afford (1*S*,2*S*,4*S*,5*R*)-2-((*R*)-hydroxy(quinolin-4-yl)methyl)-1-(4-(trifluoromethyl)benzyl)-5-vinylquinuclidin-1-ium bromide **CD3** (240 mg, 90%) as a white solid. $[\alpha]_D^{17}$ = –130 (*c* = 0.2, MeOH) [lit = –159 (*c* = 0.1, MeOH)][52]; mp = 235–237 ºC (decomposition observed) [lit = 222–223][52]; R_f 0.30 (10% MeOH/CH_2Cl_2); ν_{max} (film)/cm^{-1} 3183 (br s, OH), 2982, 2952, 1323, 1167, 1118, 1066, 921, 857, 775, 731; ^{1}H NMR (400 MHz, $CDCl_3$) δ 8.80 (d, *J* = 4.5 Hz, 1 H, HC_{Ar}), 8.16–8.11 (m, 1 H, HC_{Ar}), 7.88 (d, *J* = 7.8 Hz, 2 H, 2 × HC_{Ar}), 7.79 (d, *J* = 4.5 Hz, 1 H, HC_{Ar}), 7.58–7.53 (m, 1 H, HC_{Ar}), 7.42 (d, *J* = 7.8 Hz, 2 H, 2 × HC_{Ar}), 7.07–7.00 (m, 2 H, 2 × HC_{Ar}), 6.55–6.47 (m, 2 H, N^+CH and OH), 6.29 (d, *J* = 11.9 Hz, 1 H, $N^+C\mathit{H}HAr$), 5.59 (d, *J* = 11.9 Hz, 1 H, $N^+CH\mathit{H}Ar$), 5.43–5.28 (m, 2 H, $C\mathit{H}=C\mathit{H}H$), 4.94–4.90 (m, 1 H, $CH=CH\mathit{H}$), 4.71–4.62 (m, 1 H, $N^+C\mathit{H}H$), 4.24–4.16 (m, 1 H, $N^+C\mathit{H}H$), 3.99–3.92 (m, 1 H, $C\mathit{H}OH$), 3.06–2.90 (m, 2 H, 2 × $N^+CH\mathit{H}$), 2.52–2.44 (m, 1 H, $C\mathit{H}CH=CH_2$), 2.13–2.03 (m, 1 H, $NCHC\mathit{H}H$), 1.96–1.91 (m, 1 H, $N(CH_2)_2C\mathit{H}$), 1.90–1.81 (m, 1 H, $NCH_2C\mathit{H}H$), 1.66–1.55 (m, 1 H, $NCHC\mathit{H}H$), 1.05–0.95 (m, 1 H, $NCH_2CH\mathit{H}$); ^{13}C NMR (101 MHz, $CDCl_3$) δ 149.2 (C_{Ar}), 146.8 (C_{Ar} quat), 144.1 (C_{Ar} quat), 135.6 ($\mathit{C}H=CH_2$), 134.4 (2 × C_{Ar}), 132.1 (q, J_{C-F} = 32.7 Hz, $CF_3\mathit{C}_{Ar}$ quat), 131.0 (C_{Ar} quat), 129.4 (C_{Ar}), 128.3 (C_{Ar}), 127.2 (C_{Ar}), 125.3 (q, J_{C-F} = 3.4 Hz, 2 × C_{Ar}), 123.6 (q,

$J_{C–F}$ = 272.4 Hz, CF_3 quat), 123.2 (C_{Ar} quat), 122.6 (C_{Ar}), 119.6 (C_{Ar}), 117.9 (CH=CH_2), 67.1 (CHOH), 65.2 (N^+CH), 60.8 (N^+CH_2Ar), 60.0 (N^+CH_2), 50.5 (N^+CH_2), 37.7 (*C*HCH=CH_2), 26.2 ($N(CH_2)_2$*C*H), 25.0 (NCH*C*H_2), 22.5 (NCH_2*C*H_2); $^{19}F\{^1H\}$ NMR (377 MHz, $CDCl_3$) δ –63.1; HRMS (ESI^+) *m/z* Calculated for $C_{27}H_{28}N_2OF_3$ [M–Br] 453.2154; Found 453.2157. Compound previously characterised in DMSO-*d*6.[52]

SMILES:
C=C[C@H]1C[N@@+]2(CC3=CC=C(C(F)(F)F)C=C3)[C@H]([C@H](O)C4=C(C=CC=C5)C5=NC=C4)C[C@@H]1CC2.[Br-]
InChI=1S/C27H28F3N2O.BrH/c1-2-19-17-32(16-18-7-9-21(10-8-18)27(28,29)30)14-12-20(19)15-25(32)26(33)23-11-13-31-24-6-4-3-5-22(23)24;/h2-11,13,19-20,25-26,33H,1,12,14-17H2;1H/p-1/t19-,20-,25-,26+;/m0./s1

(1*S*,2*S*,4*S*,5*R*)-2-((*S*)-Hydroxy(quinolin-4-yl)methyl)-1-(4-(trifluoromethyl)benzyl)-5-vinylquinuclidin-1-ium bromide (CN1)

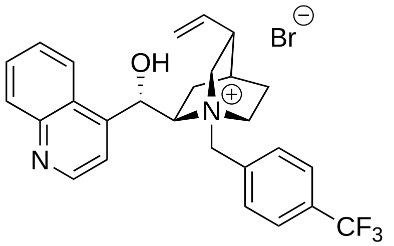

A solution of cinchonine (147 mg, 0.5 mmol) and 4-(trifluromethyl)benzyl bromide (120 mg, 0.5 mmol) in THF (2.5 mL) was heated to 70 ºC for 23 h. The reaction was cooled to rt and Et_2O was added. The reaction was stirred for approx. 0.5 h then the precipitate was filtered. The collected solid was dried to afford (1*S*,2*R*,4*S*,5*R*)-2-((*S*)-hydroxy(quinolin-4-yl)methyl)-1-(4-(trifluoromethyl)benzyl)-5-vinylquinuclidin-1-ium bromide **CN1** (227 mg, 85%) as a white solid. $[\alpha]_D^{21}$ = +150 (*c* = 0.16, MeOH) [lit = +135 (*c* = 0.50, MeOH)][51] R_f 0.23 (10% MeOH/CH_2Cl_2); mp = 240–245 ºC (decomposition observed) [lit = 244–246 ºC (with decomp.)][51] ν_{max} (film)/cm^{-1} 3183 (br s, OH), 2960, 2878, 1327, 1126, 865, 779; ^1H NMR (400 MHz, $CDCl_3$) δ 8.83 (d, *J* = 4.5 Hz, 1 H, HC_{Ar}), 8.25 (d, *J* = 8.1 Hz, 1 H, HC_{Ar}), 7.87–7.79 (m, 3 H, 3 × HC_{Ar}), 7.51 (dd, *J* = 8.3, 1.2 Hz, 1 H, HC_{Ar}), 7.39 (d, *J* = 8.1 Hz, 2 H, 2 × HC_{Ar}), 7.01–6.89 (m, 2 H, 2 × HC_{Ar}), 6.57 (d, *J* = 5.6 Hz, 1 H, N^+CH), 6.53–6.36 (m, 2 H, OH and N^+C*H*HAr), 5.82 (ddd, *J* = 17.4, 10.4, 7.2 Hz, 1 H, C*H*=CH_2), 5.43–5.31 (m, 1 H, N^+CH*H*Ar), 5.26–5.15 (m, 2 H, CH=C*H*2), 4.53–4.44 (m, 1 H, N^+C*H*H), 4.24–4.05 (m, 2 H, N^+C*H*H and C*H*OH), 3.16 (t, *J* = 11.4 Hz, 1 H, N^+CH*H*), 2.69–2.59 (m, 1 H, N^+CH*H*), 2.29 (q, *J* = 8.8 Hz, 1 H, C*H*CH=CH_2), 2.14–2.05 (m, 1 H, NCHC*H*H), 1.89–1.66 (m, 3 H, $N(CH_2)_2C$*H*, NCH_2C*H*H and NCHCH*H*), 0.77–0.68 (m, 1 H, NCH_2CH*H*); ^1H NMR (400 MHz, DMSO-d_6) δ 8.99 (d, *J* = 4.5 Hz, 1 H, HC_{Ar}), 8.44–8.38 (m, 1 H, HC_{Ar}), 8.12–8.03 (m, 3 H, 3 × HC_{Ar}), 7.96 (d, *J* = 8.2 Hz, 2 H, 2 × HC_{Ar}), 7.88–7.81 (m, 2 H, 2 × HC_{Ar}), 7.78–7.72 (m, 1 H, HC_{Ar}), 6.84 (dd, *J* = 14.8, 4.2 Hz, 1 H, OH), 6.55–6.50 (m, 1 H, N^+CH), 6.00 (ddd, *J* = 17.3, 10.7, 7.0 Hz, 1 H, C*H*=CH_2), 5.31 (d, *J* = 12.3 Hz, 1 H, N^+C*H*HAr), 5.26–5.19 (m, 2 H, CH=C*H*2), 5.12 (d, *J* = 12.3 Hz, 1 H, N^+CH*H*Ar), 4.31–4.23 (m, 1 H, N^+C*H*H), 4.13–4.03 (m, 1 H, N^+C*H*H), 3.97 (t, *J* = 9.2 Hz, 1 H, C*H*OH), 3.48 (t, *J* = 11.4 Hz, 1 H, N^+CH*H*), 3.05–2.91 (m, 1 H, N^+CH*H*), 2.65 (q, *J* = 8.4 Hz, 1 H, *C*HCH=CH_2), 2.34–2.22 (m, 1 H, NCHC*H*H), 1.89–1.85 (m, 1 H, $N(CH_2)_2C$*H*), 1.79–1.71 (m, 2 H, NCH_2C*H*H and NCHC*H*H), 1.10–1.00 (m, 1 H, NCHCH*H*); ^{13}C NMR (101 MHz, DMSO-d_6) δ 150.1 (C_{Ar}), 147.6 (C_{Ar} quat), 144.9 (C_{Ar} quat), 137.1 (*C*H=CH_2), 134.7 (2 × C_{Ar}), 132.6 (C_{Ar} quat), 130.3 (q, $J_{C–F}$ = 31.9 Hz, CF_3C_{Ar} quat), 129.7 (C_{Ar}), 129.4 (C_{Ar}), 127.2 (C_{Ar}), 125.7 (q, $J_{C–F}$ = 3.1 Hz, 2 × C_{Ar}), 124.3 (C_{Ar}), 124.01 (q, $J_{C–F}$ = 272.5 Hz, *C*F_3 quat), 123.98 (C_{Ar} quat), 120.1 (C_{Ar}), 117.0 (CH=*C*H_2), 67.3 (*C*HOH), 64.6 (N^+CH), 61.2 (N^+*C*H_2Ar), 56.0 (N^+CH_2), 53.9 (N^+CH_2), 36.6 (*C*HCH=CH_2), 26.2 ($N(CH_2)$*C*H), 22.9 (NCH*C*H_2), 20.7 (NCH_2*C*H_2); $^{19}F\{^1H\}$ NMR (377 MHz, DMSO-d_6) δ –61.3The observed characterisation data (IR, ^1H and ^{13}C NMR in DMSO-*d*6) was consistent with that previously reported in the literature.[51]

(1*S*,2*S*,4*S*,5*R*)-2-((*R*)-Hydroxy(quinolin-4-yl)methyl)-1-(4-methylbenzyl)-5-vinylquinuclidin-1-ium bromide (CD4)

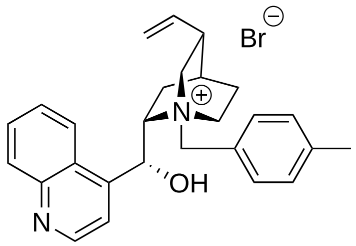

A solution of cinchonidine (147 mg, 0.5 mmol) and 1-(bromomethyl)-4-methylbenzene (92 mg, 0.5 mmol) in THF (2.5 mL) was heated to 70 ºC for 15 h. The reaction was cooled to rt and Et_2O was added. The reaction was stirred for approx. 0.5 h then the precipitate was filtered. The collected solid was dried to afford (1*S*,2*S*,4*S*,5*R*)-2-((*R*)-hydroxy(quinolin-4-yl)methyl)-1-(4-methylbenzyl)-5-vinylquinuclidin-1-ium bromide **CD4** (203 mg, 85%) as a white solid. $[\alpha]_D^{21}$ = –144º (*c* = 0.5, CH_2Cl_2); R_f 0.23 (10% MeOH/CH_2Cl_2); mp = 240–245 ºC (decomposition observed); ν_{max} (film)/cm^{-1} 3183 (br s, OH), 2948, 1510, 1461, 924, 775, 731; ^{1}H NMR (400 MHz, $CDCl_3$) δ 8.79 (d, *J* = 4.5 Hz, 1 H, HC_{Ar}), 8.18–8.13 (m, 1 H, HC_{Ar}), 7.81 (d, *J* = 4.5 Hz, 1 H, HC_{Ar}), 7.64–7.59 (m, 1 H, HC_{Ar}), 7.52 (d, *J* = 7.8 Hz, 2 H, 2 × HC_{Ar}), 7.15–7.08 (m, 2 H, 2 × HC_{Ar}), 6.91 (d, *J* = 7.8 Hz, 2 H, 2 × HC_{Ar}), 6.59 (d, *J* = 6.0 Hz, 1 H, OH), 6.49–6.45 (m, 1 H, N^+CH), 5.94 (d, *J* = 11.9 Hz, 1 H, N^+C*H*HAr), 5.48–5.34 (m, 2 H, N^+CH*H*Ar and C*H*=CH_2), 5.31–5.25 (m, 1 H, CH=C*H*H), 4.89 (dd, *J* = 10.3, 1.2 Hz, 1 H, CH=CH*H*), 4.63–3.52 (m, 1 H, N^+C*H*H), 4.13 (br t, *J* = 8.8 Hz, 1 H, C*H*OH), 3.86 (br dt, *J* = 12.8, 2.8 Hz, 1 H, N^+C*H*H), 3.14–3.04 (m, 2 H, 2 × N^+CH*H*), 2.48–2.39 (m, 1 H, C*H*CH=CH_2), 2.28 (s, 3 H, $ArCH_3$), 2.09–1.99 (m, 1 H, NCHC*H*H), 1.92–1.77 (m, 2 H, $N(CH_2)_2C$*H* and NCH_2C*H*H), 1.61–1.49 (m, 1 H, NCHCH*H*), 1.04–0.95 (m, 1 H, NCH_2CH*H*); ^{13}C NMR (101 MHz, $CDCl_3$) δ 149.2 (C_{Ar}), 146.9 (C_{Ar} quat), 144.7 (C_{Ar} quat), 139.8 (C_{Ar} quat), 136.0 (*C*H=CH_2), 133.9 (2 × C_{Ar}), 129.4 (C_{Ar}), 129.2 (2 × C_{Ar}), 128.4 (C_{Ar}), 127.3 (C_{Ar}), 123.8 (C_{Ar} quat), 123.5 (C_{Ar} quat), 122.9 (C_{Ar}), 119.8 (C_{Ar}), 117.7 (CH=*C*H_2), 66.8 (CHOH), 65.1 (N^+CH), 62.0 (N^+CH_2Ar), 59.9 (N^+CH_2), 50.1 (N^+CH_2), 37.8 (*C*HCH=CH_2), 26.4 ($N(CH_2)_2CH$), 25.1 (NCH*C*H_2), 22.4 (NCH_2*C*H_2), 21.3 ($ArCH_3$); HRMS (ESI$^+$) *m/z* Calculated for $C_{27}H_{31}N_2O$ [M–Br] 399.2436; Found 399.2443.

SMILES:
C=C[C@H]1C[N@@+]2(CC3=CC=C(C)C=C3)[C@H]([C@@H](C4=C(C=CC=C5)C5=NC=C4)O)C[C@@H]1CC2.[Br-]
InChI=1S/C27H31N2O.BrH/c1-3-21-18-29(17-20-10-8-19(2)9-11-20)15-13-22(21)16-26(29)27(30)24-12-14-28-25-7-5-4-6-23(24)25;/h3-12,14,21-22,26-27,30H,1,13,15-18H2,2H3;1H/q+1;/p-1/t21-,22-,26-,27+,29+;/m0./s1

(1*S*,2*S*,4*S*,5*R*)-1-(4-Chlorobenzyl)-2-((*R*)-hydroxy(quinolin-4-yl)methyl)-5-vinylquinuclidin-1-ium bromide (CD5)

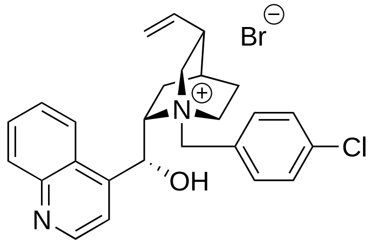

A solution of cinchonidine (147 mg, 0.5 mmol) and 1-(chloromethyl)-4-methylbenzene (103 mg, 0.5 mmol) in THF (2.5 mL) was heated to 70 ºC for 15 h. The reaction was cooled to rt and Et_2O was added. The reaction was stirred for approx. 0.5 h then the precipitate was filtered. The collected solid was dried to afford (1*S*,2*S*,4*S*,5*R*)-1-(4-chlorobenzyl)-2-((*R*)-hydroxy(quinolin-4-yl)methyl)-5-vinylquinuclidin-1-ium bromide **CD5** (236 mg, 94%) as a white solid. $[\alpha]_D^{22}$ = –167º (*c* = 0.3, MeOH); R_f 0.38 (10% MeOH/CH_2Cl_2); mp = 240–247 ºC (decomposition observed); ν_{max} (film)/cm^{-1} 3124 (br s, OH), 2982, 2948, 1592, 1491, 1457, 1092, 1066, 921, 731; ^{1}H NMR (400 MHz, $CDCl_3$) δ 8.80 (d, *J* = 4.5 Hz, 1 H, HC_{Ar}), 8.19–8.13 (m, 1 H, HC_{Ar}), 7.82 (d, *J* = 4.5 Hz, 1 H, HC_{Ar}), 7.65–7.54 (m, 3 H, 3 × HC_{Ar}), 7.16 (d, *J* = 8.5 Hz, 2 H, 2 × HC_{Ar}), 7.06–6.99 (m, 2 H, 2 × HC_{Ar}), 6.58 (d, *J* = 5.9 Hz, 1 H, OH), 6.48–6.43 (m, 1 H, N^+CH), 6.19 (d, *J* = 11.9 Hz, 1 H, N^+C*H*HAr), 5.48 (d, *J* = 11.9 Hz, 1 H, N^+CH*H*Ar), 5.43–5.29 (m, 2 H, C*H*=C*H*H), 4.93–4.88 (m, 1 H, CH=CH*H*), 4.65–4.55 (m, 1 H, N^+C*H*H), 4.18 (br t, *J* = 9.3 Hz, 1 H, C*H*OH), 3.99–3.91 (m, 1 H, N^+C*H*H), 3.09–2.95 (m, 2 H, 2 × N^+CH*H*), 2.51–2.43 (m, 1 H, C*H*CH=CH_2), 2.12–2.00 (m, 1 H, NCHC*H*H), 1.92 (br s, 1 H, $N(CH_2)_2C$*H*), 1.87–1.77 (m, 1 H, NCH_2C*H*H), 1.65–1.53 (m, 1 H, NCHCH*H*), 1.04–0.92 (m, 1 H, NCH_2CH*H*); ^{13}C NMR (101 MHz, $CDCl_3$) δ 149.0 (C_{Ar}), 146.5 (C_{Ar} quat), 144.7 (C_{Ar} quat), 136.8 (C_{Ar} quat), 135.7 (*C*H=CH_2), 135.3 (2 × C_{Ar}), 129.3 (C_{Ar}), 128.9 (2 × C_{Ar}), 128.4 (C_{Ar}), 127.3 (C_{Ar}), 125.3 (C_{Ar} quat), 123.3 (C_{Ar} quat), 122.7 (C_{Ar}), 119.6 (C_{Ar}), 117.9 (CH=*C*H_2), 66.8 (CHOH), 65.2 (N^+CH), 60.9 (N^+CH_2Ar), 59.7 (N^+CH_2), 50.1 (N^+CH_2), 37.7 (*C*HCH=CH_2), 26.3

(N(CH_2)$_2$*C*H), 25.1 (NCH*C*H_2), 22.5 (NCH_2*C*H_2); HRMS (ESI$^+$) *m/z* Calculated for $C_{26}H_{28}N_2O^{35}Cl$ [M–Br] 419.1890; Found 419.1901.

SMILES:
C=C[C@H]1C[N@@+]2(CC3=CC=C(Cl)C=C3)[C@H]([C@@H](C4=C(C=CC=C5)C5=NC=C4)O)C[C@@H]1CC2.[Br-]
InChI=1S/C26H28ClN2O.BrH/c1-2-19-17-29(16-18-7-9-21(27)10-8-18)14-12-20(19)15-25(29)26(30)23-11-13-28-24-6-4-3-5-22(23)24;/h2-11,13,19-20,25-26,30H,1,12,14-17H2;1H/q+1;/p-1/t19-,20-,25-,26+,29+;/m0./s1

(1*S*,2*S*,4*S*,5*R*)-2-((*R*)-Hydroxy(quinolin-4-yl)methyl)-1-(4-(pentafluoro-λ^6-sulfaneyl)benzyl)-5-vinylquinuclidin-1-ium bromide CD6 (Cat7)

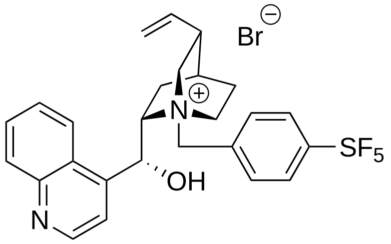

A solution of cinchonidine (736 mg, 2.5 mmol) and (4-(bromomethyl)phenyl)pentafluoro-λ^6-sulfane **S72** (743 mg, 2.5 mmol) in THF (12.5 mL) was heated to 70 ºC for 14 h. The reaction was cooled to rt and Et_2O was added. The reaction was stirred for approx. 0.5 h then the precipitate was filtered. The collected solid was dried to afford (1*S*,2*S*,4*S*,5*R*)-2-((*R*)-hydroxy(quinolin-4-yl)methyl)-1-(4-(pentafluoro-λ^6-sulfaneyl)benzyl)-5-vinylquinuclidin-1-ium bromide **CD6** (1.38 g, 93%) as a white solid. $[\alpha]_D^{22}$ = –144 (*c* = 0.5, CH_2Cl_2); R_f 0.30 (10% MeOH/CH_2Cl_2); mp = 220–223 ºC (decomposition observed); ν_{max} (film)/cm^{-1} 3183 (br s, OH), 2986, 1510, 921, 839, 731; ^{1}H NMR (400 MHz, $CDCl_3$) δ 8.81 (d, *J* = 4.5 Hz, 1 H, HC_{Ar}), 8.19–8.13 (m, 1 H, HC_{Ar}), 7.97 (d, *J* = 8.3 Hz, 2 H, 2 × HC_{Ar}), 7.79 (d, *J* = 4.5 Hz, 1 H, HC_{Ar}), 7.69–7.64 (m, 1 H, HC_{Ar}), 7.61 (d, *J* = 8.5 Hz, 2 H, 2 × HC_{Ar}), 7.18–7.12 (m, 2 H, 2 × HC_{Ar}), 6.55 (d, *J* = 5.9 Hz, 1 H, OH), 6.50–6.46 (m, 1 H, N$^+$CH), 6.18 (d, *J* = 12.0 Hz, 1 H, N$^+$C*H*HAr), 5.62 (d, *J* = 12.0 Hz, 1 H, N$^+$CH*H*Ar), 5.45–5.35 (m, 1 H, C*H*=CH_2), 5.28 (dd, *J* = 17.3, 0.6 Hz, 1 H, CH=C*H*H), 4.93 (dd, *J* = 10.3, 1.3 Hz, 1 H, CH=CH*H*), 4.72–4.62 (m, 1 H, N$^+$C*H*H), 4.16 (br t, *J* = 9.2 Hz, 1 H, C*H*OH), 3.96–3.88 (m, 1 H, N$^+$C*H*H), 3.10–2.93 (m, 2 H, 2 × N$^+$CH*H*), 2.55–2.48 (m, 1 H, C*H*CH=CH_2), 2.15–2.06 (m, 1 H, NCHC*H*H), 1.98–1.92 (m, 1 H, N(CH_2)$_2$C*H*), 1.91–1.83 (m, 1 H, NCH_2C*H*H), 1.70–1.59 (m, 1 H, NCHCH*H*), 1.08–0.98 (m, 1 H, NCH_2CH*H*); ^{13}C NMR (101 MHz, $CDCl_3$) δ 155.0 (p, $J_{C–F}$ = 17.7 Hz, SF_5C_{Ar} quat), 149.3 (C_{Ar}), 146.9 (C_{Ar} quat), 144.4 (C_{Ar} quat), 135.6 (*C*H=CH_2), 134.5 (2 × C_{Ar}), 131.1 (C_{Ar} quat), 129.6 (C_{Ar}), 128.7 (C_{Ar}), 127.4 (C_{Ar}), 126.3 (br s, 2 × C_{Ar}), 123.5 (C_{Ar} quat), 122.8 (C_{Ar}), 119.8 (C_{Ar}), 118.1 (CH=*C*H_2), 67.7 (CHOH), 65.1 (N$^+$CH), 60.8 (N$^+$$CH_2$Ar), 60.3 (N$^+$$CH_2$), 50.9 (N$^+$$CH_2$), 37.8 (*C*HCH=$CH_2$), 26.2 (N($CH_2$)$_2$*C*H), 25.0 (NCH*C*$H_2$), 22.4 (N$CH_2$*C*$H_2$); ^{19}F{^{1}H} NMR (377 MHz, $CDCl_3$) δ 82.6 (p, $J_{F–F}$ = 150 Hz, F_{ax}), 62.5 (d, $J_{F–F}$ = 150 Hz, 4 × F_{eq}); HRMS (ESI$^+$) *m/z* Calculated for $C_{26}H_{28}N_2OSF_5$ [M–Br] 511.1843; Found 511.1847.

SMILES:
C=C[C@H]1C[N@@+]2(CC3=CC=C(S(F)(F)(F)(F)F)C=C3)[C@H]([C@@H](C4=C(C=CC=C5)C5=NC=C4)O)C[C@@H]1CC2.[Br-]
InChI=1S/C26H28F5N2OS.BrH/c1-2-19-17-33(16-18-7-9-21(10-8-18)35(27,28,29,30)31)14-12-20(19)15-25(33)26(34)23-11-13-32-24-6-4-3-5-22(23)24;/h2-11,13,19-20,25-26,34H,1,12,14-17H2;1H/q+1;/p-1/t19-,20-,25-,26+,33+;/m0./s1

(1*S*,2*S*,4*S*,5*R*)-2-((*R*)-((4-(Pentafluoro-λ^6-sulfaneyl)benzyl)oxy)(quinolin-4-yl)methyl)-5-vinylquinuclidine (S74)

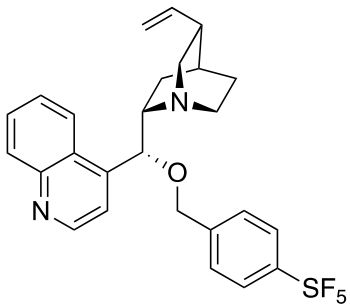

Isolated as a side product from a cyclisation reaction following general procedure **D** using cyclisation precursor **2a**. Purification by flash chromatography (40% to 50% Et_2O/pentane afforded (1*S*,2*S*,4*S*,5*R*)-2-((*R*)-((4-(pentafluoro-λ^6-sulfaneyl)benzyl)oxy)(quinolin-4-yl)methyl)-5-vinylquinuclidine **S74** (12 mg, 60%) as an amorphous white solid. R_f 0.13 (40% Et_2O/pentane); mp = 46–49 ºC; ν_{max} (film)/cm^{-1} 2934, 2794, 2763, 1594, 1510, 1096, 842, 798; ^1H NMR (400 MHz, $CDCl_3$) δ 8.90 (d, *J* = 4.5 Hz, HC_{Ar}), 8.17 (d, *J* = 8.4 Hz, HC_{Ar}), 8.05 (dd, *J* = 8.3, 0.6 Hz, HC_{Ar}), 7.78 (ddd, *J* = 8.3, 7.0, 1.4 Hz, 1 H, HC_{Ar}), 7.69 (d, *J* = 8.7 Hz, 2 H, 2 × HC_{Ar}), 7.62 (ddd, *J* = 8.3, 7.0, 1.2 Hz, 1 H, HC_{Ar}), 7.42 (d, *J* = 8.3 Hz, 2 H, 2 × HC_{Ar}), 7.34 (d, *J* = 4.4 Hz, 1 H, HC_{Ar}), 6.25 (dt, *J* = 17.6, 9.6 Hz, 1 H, CH_2=C*H*), 5.15 (dd, *J* = 10.3, 1.9 Hz, 1 H, C*H*H=CH), 5.07 (dd, *J* = 17.2, 1.7 Hz, 1 H, CH*H*=CH), 4.24 (d, *J* = 1.8 Hz, OC*H*), 3.55 (d, *J* = 14.1 Hz, 1 H, OC*H*HAr), 3.47 (d, *J* = 14.1 Hz, 1 H, OCH*H*Ar), 3.00–2.96 (m, 1 H, NCH), 2.89–2.82 (m, 1 H, NC*H*H), 2.78–2.72 (m, 1 H, NC*H*H), 2.49–2.43 (m, 1 H, CH_2=CHC*H*), 2.32 (dd, *J* = 11.0, 2.6 Hz, 1 H, NCH*H*), 2.20–2.12 (m, 1 H, NCH*H*), 2.02–1.86 (m, 2 H, NCHC*H*H and N$(CH_2)_2$C*H*), 1.74–1.65 (m, 2 H, NCH_2CH_2), 1.60–1.53 (m, 1 H, NCHCH*H*); ^{13}C NMR (101 MHz, $CDCl_3$) δ 150.6 (C_{Ar}), 147.8 (C_{Ar}), 143.5 (C_{Ar}), 143.2 (C_{Ar} quat), 138.0 (CH_2=*C*H), 130.4 (C_{Ar}), 129.4 (C_{Ar}), 128.6 (2 × C_{Ar}), 126.8 (C_{Ar}), 126.4 (C_{Ar} quat), 125.8 (t, $J_{C\text{–}F}$ = 4.5 Hz, 2 × C_{Ar}), 125.5 (C_{Ar} quat), 122.6 (C_{Ar}), 116.6 (*C*H_2=CH), 116.3 (C_{Ar}), 62.2 (OCH_2Ar), 61.8 (NCH), 59.0 (NCH_2), 55.2 (OCH), 53.7 (NCH_2), 43.5 (CH_2=CH*C*H), 36.8 (N$(CH_2)_2$*C*H), 35.9 (NCH*C*H_2), 28.6 (NCH_2CH_2), (The signal due to SF_5C_{Ar} quat was not observed); ^{19}F NMR (377 MHz, $CDCl_3$) δ 85.2 (p, $J_{F\text{–}F}$ = 153 Hz, F_{ax}), 63.1 (d, $J_{F\text{–}F}$ = 150 Hz, F_{eq}); HRMS (FTMS + pAPCI) *m/z* Calculated for $C_{26}H_{28}N_2OSF_5^+$ $[M+H]^+$ 511.1837; Found 511.1838.

SMILES:
C=C[C@H]1C[N@]2[C@H]([C@@H](C3=C(C=CC=C4)C4=NC=C3)OCC5=CC=C(S(F)(F)(F)(F)F)C=C5)C[C@@H]1CC2
InChI=1S/C26H27F5N2OS/c1-2-19-16-33-14-12-20(19)15-25(33)26(23-11-13-32-24-6-4-3-5-22(23)24)34-17-18-7-9-21(10-8-18)35(27,28,29,30)31/h2-11,13,19-20,25-26H,1,12,14-17H2/t19-,20-,25-,26+/m0/s1

NMR Spectra of Selected Compounds

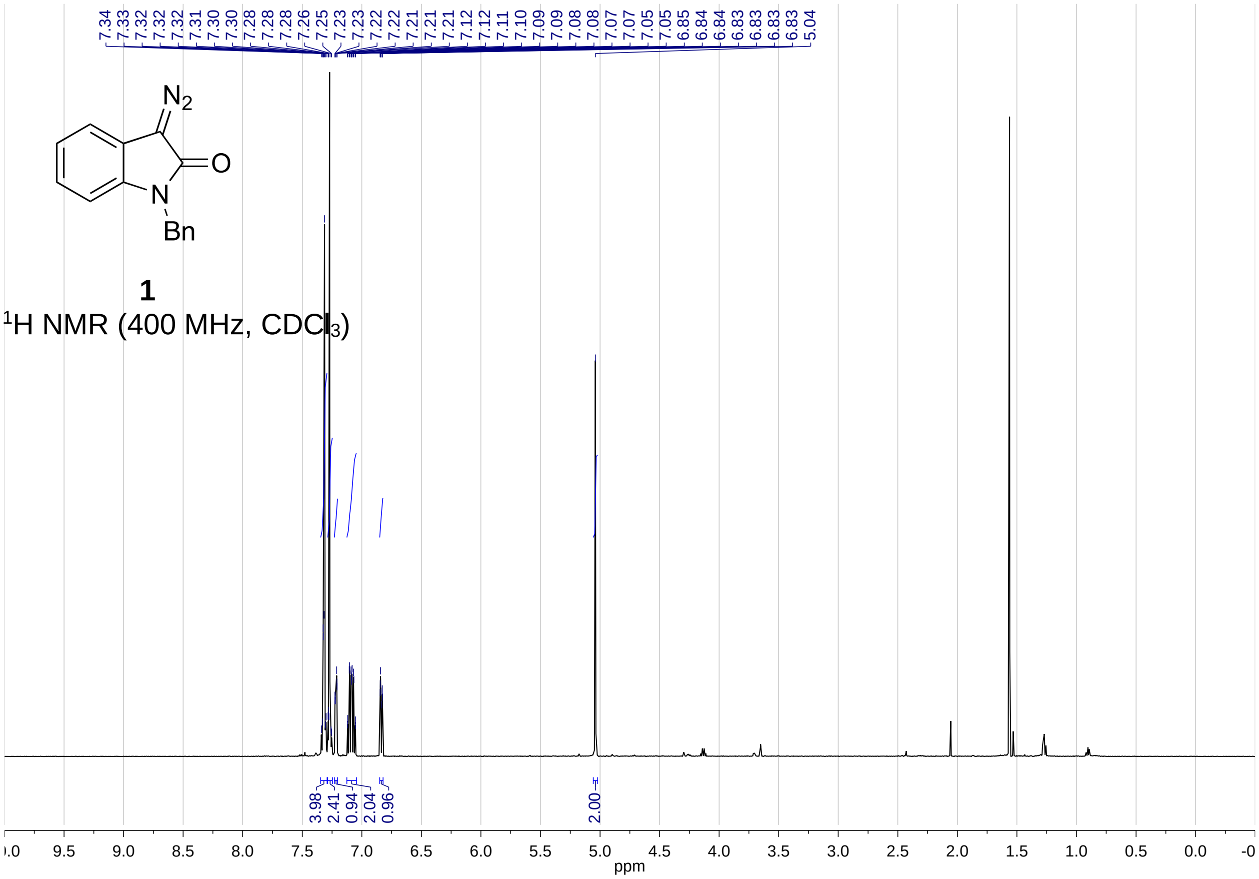

1

^{1}H NMR (400 MHz, $CDCl_3$)

1

^{13}C NMR (101 MHz, $CDCl_3$)

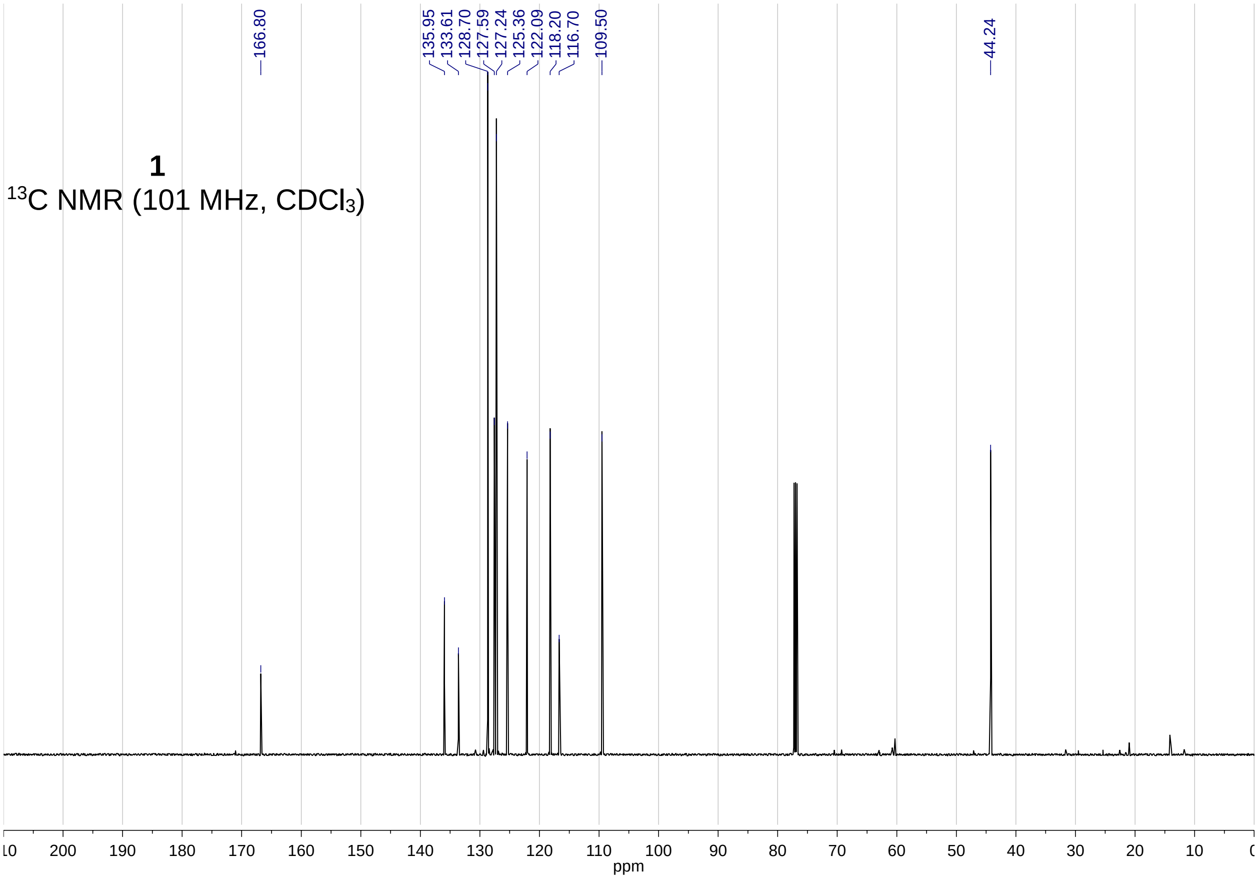

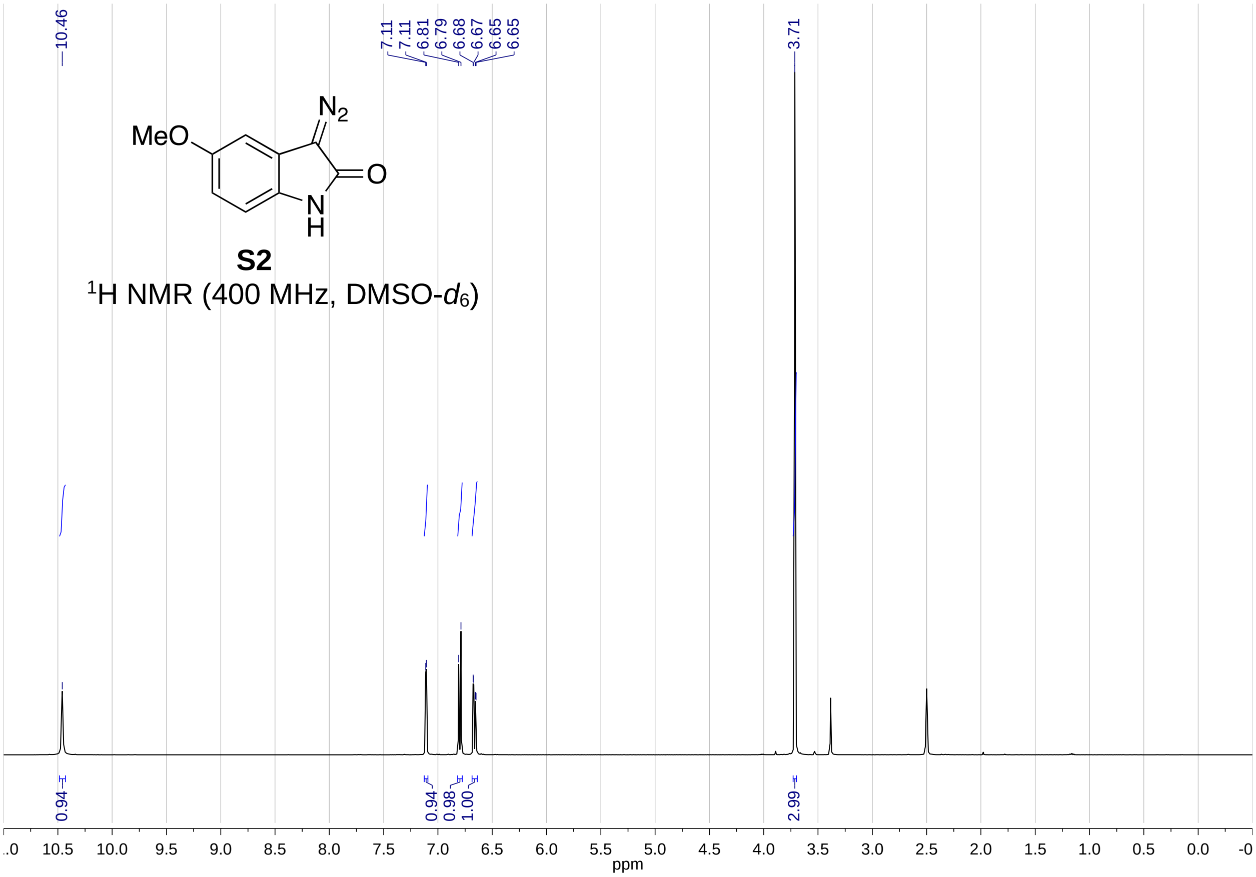
10.46
7.11
7.11
6.81
6.79
6.68
6.67
6.65
6.65
3.71
MeO
N2
O
N
H
S2
1H NMR (400 MHz, DMSO-d6)
0.94
0.94
0.98
1.00
2.99
ppm

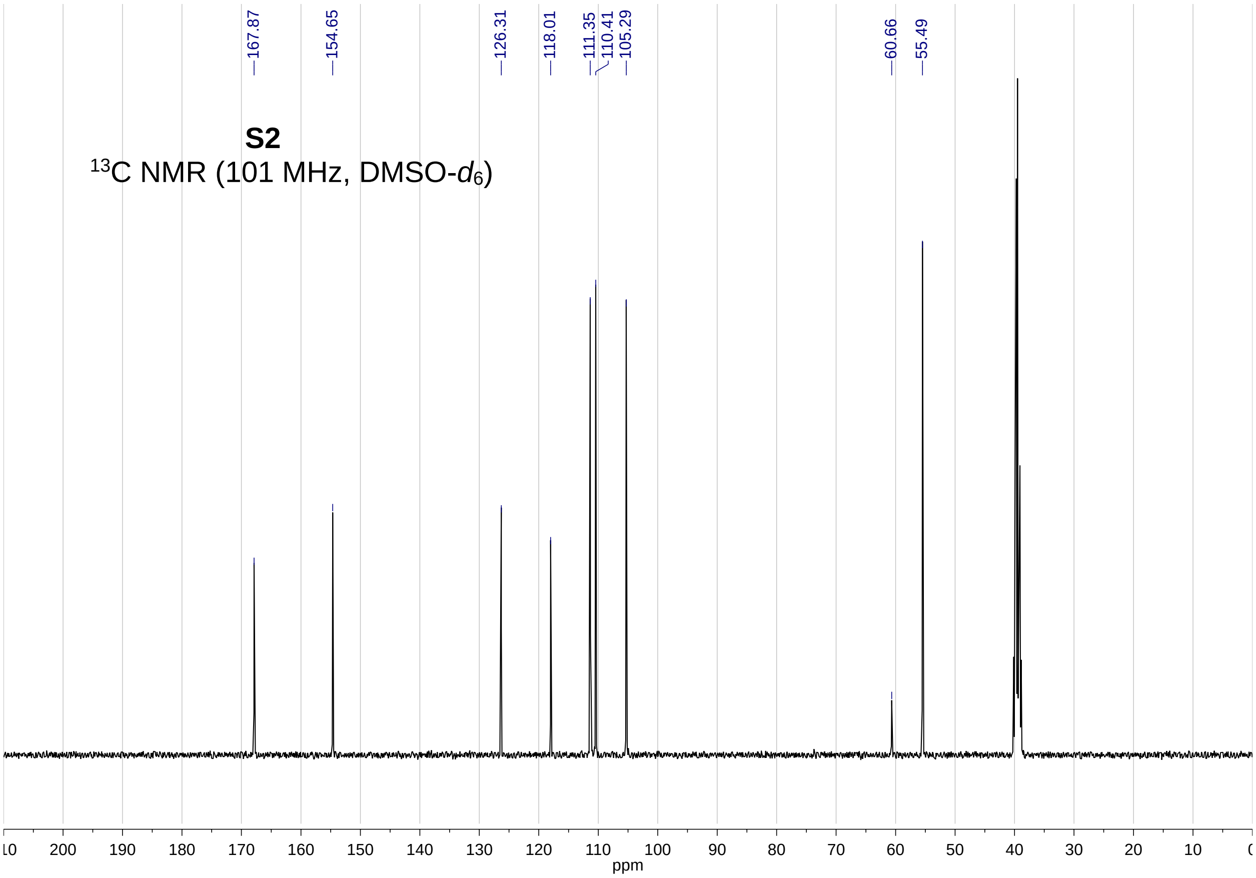
167.87
154.65
126.31
118.01
111.35
110.41
105.29
60.66
55.49
S2
13C NMR (101 MHz, DMSO-d6)
ppm

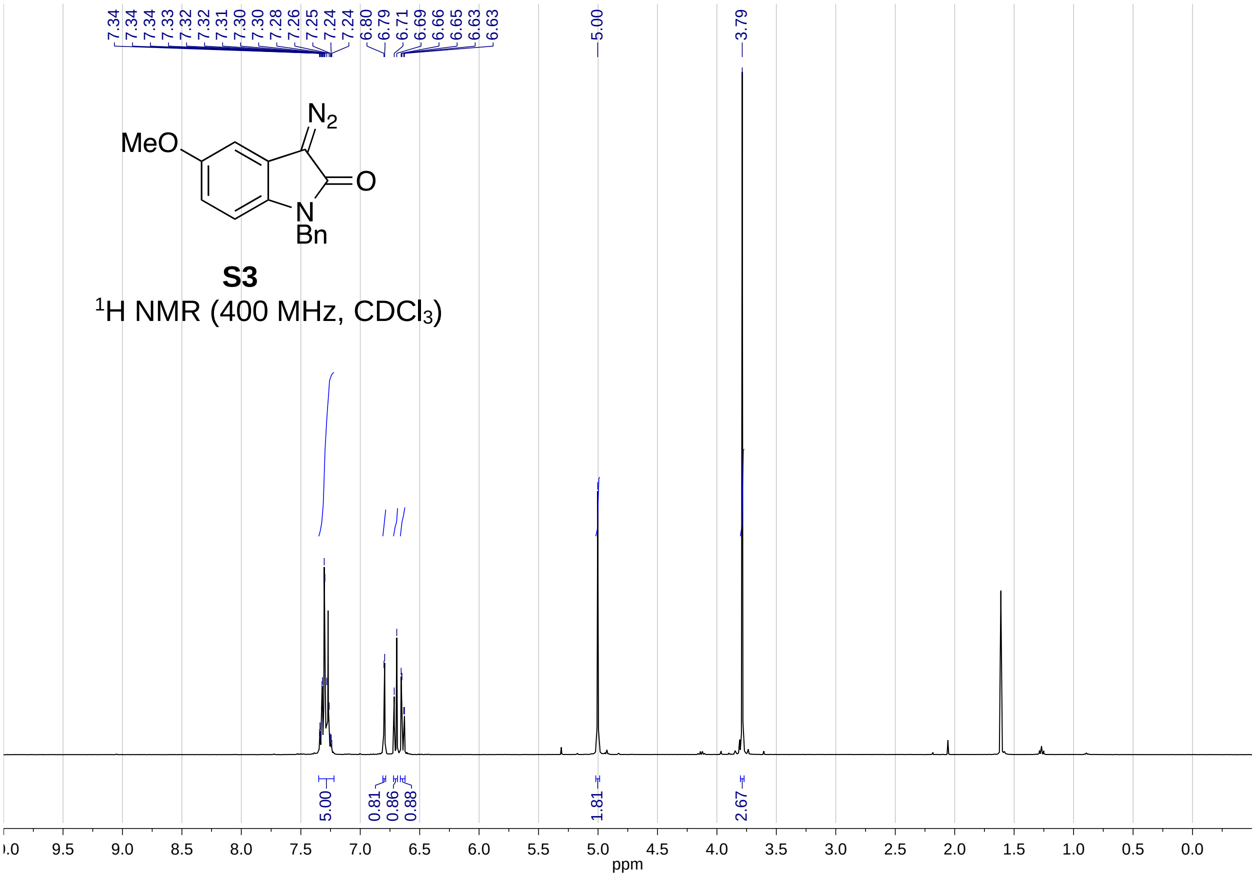
7.34
7.34
7.34
7.33
7.32
7.32
7.31
7.30
7.30
7.28
7.26
7.25
7.24
7.24
6.80
6.79
6.71
6.69
6.66
6.65
6.63
6.63
5.00
3.79
MeO
N2
O
N
Bn
S3
1H NMR (400 MHz, CDCl3)
5.00
0.81
0.86
0.88
1.81
2.67
ppm

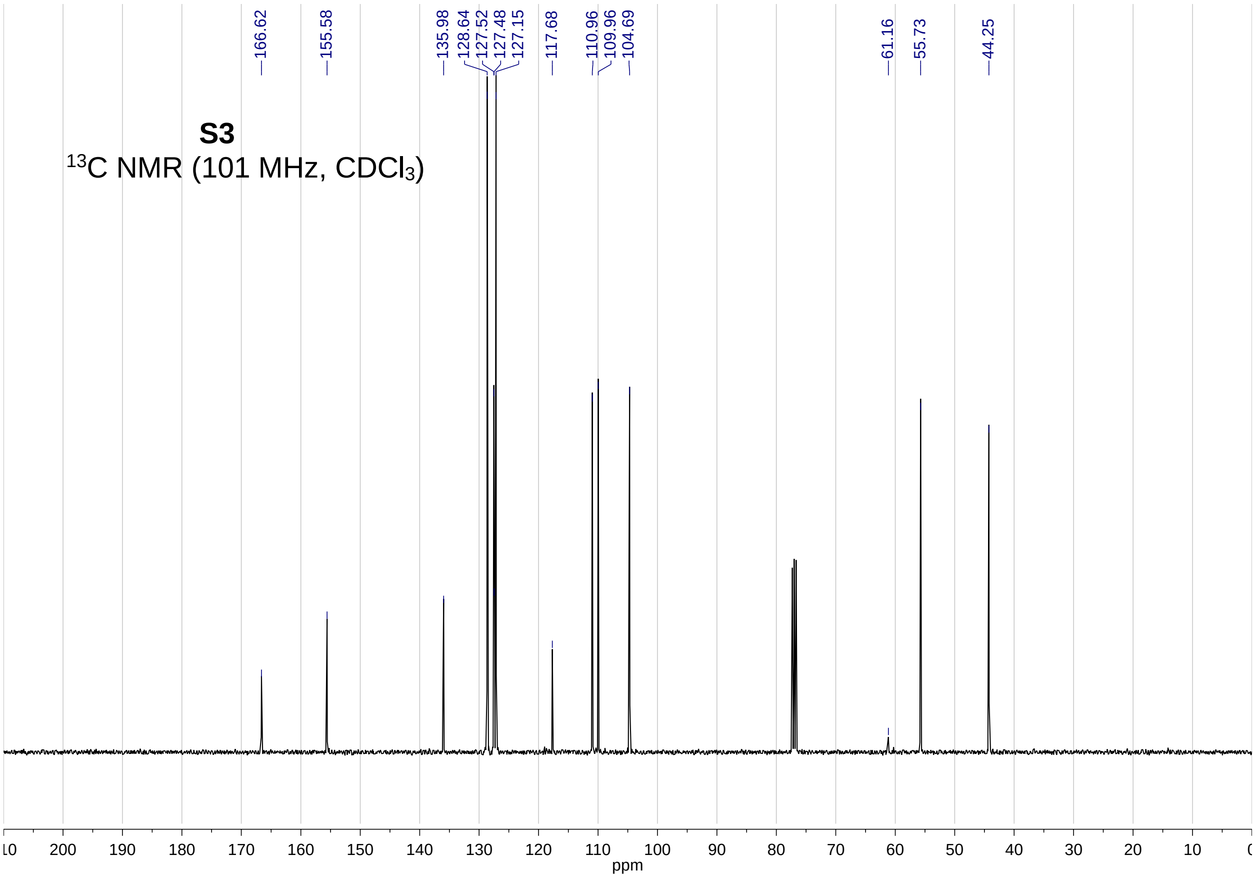
166.62
155.58
135.98
128.64
127.52
127.48
127.15
117.68
110.96
109.96
104.69
61.16
55.73
44.25
S3
13C NMR (101 MHz, CDCl3)
ppm

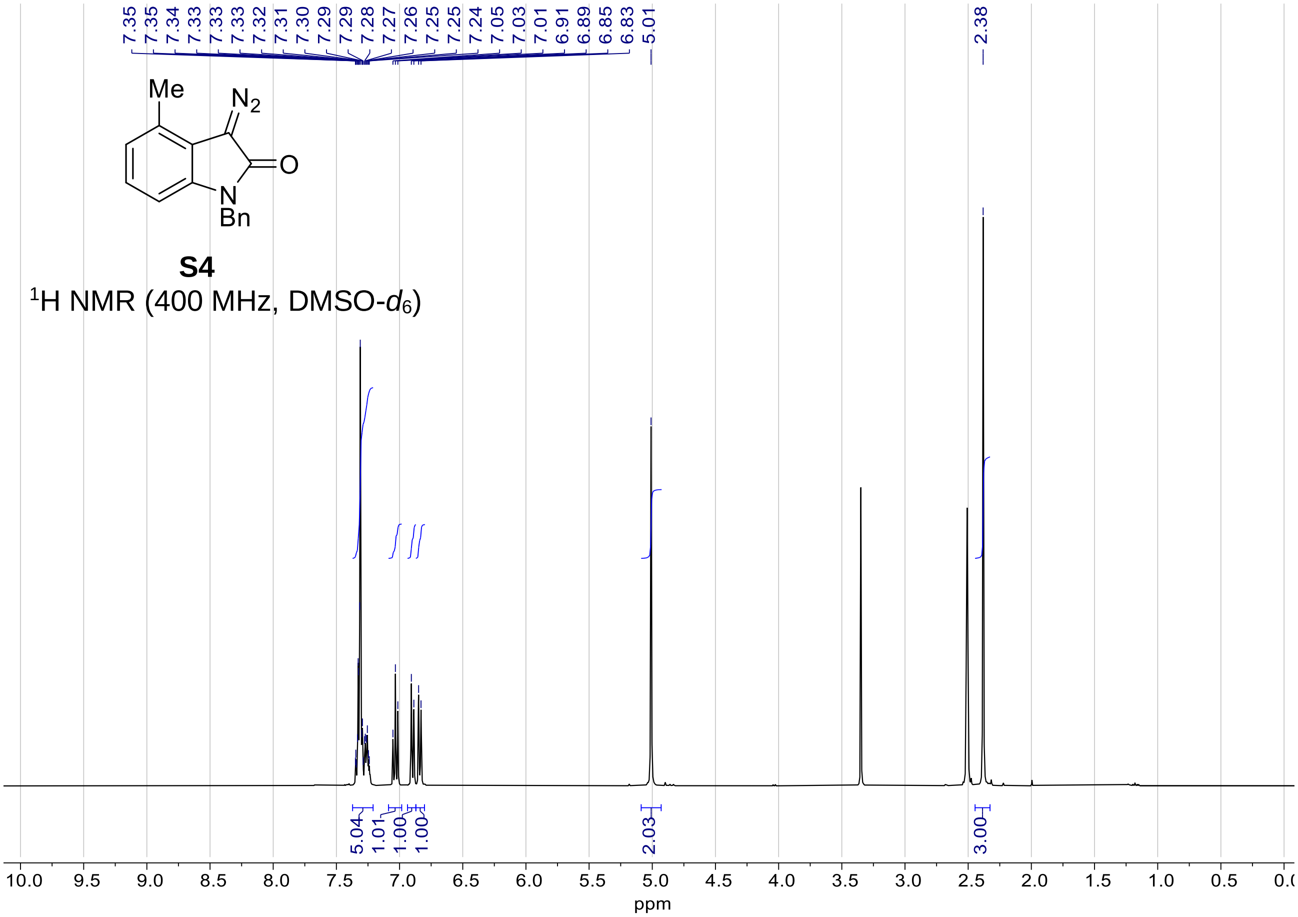
7.35
7.35
7.34
7.33
7.33
7.33
7.32
7.31
7.30
7.29
7.29
7.28
7.27
7.26
7.25
7.25
7.24
7.05
7.03
7.01
6.91
6.89
6.85
6.83
5.01
2.38
Me
N2
O
N
Bn
S4
1H NMR (400 MHz, DMSO-d6)
5.04
1.01
1.00
1.00
2.03
3.00
ppm

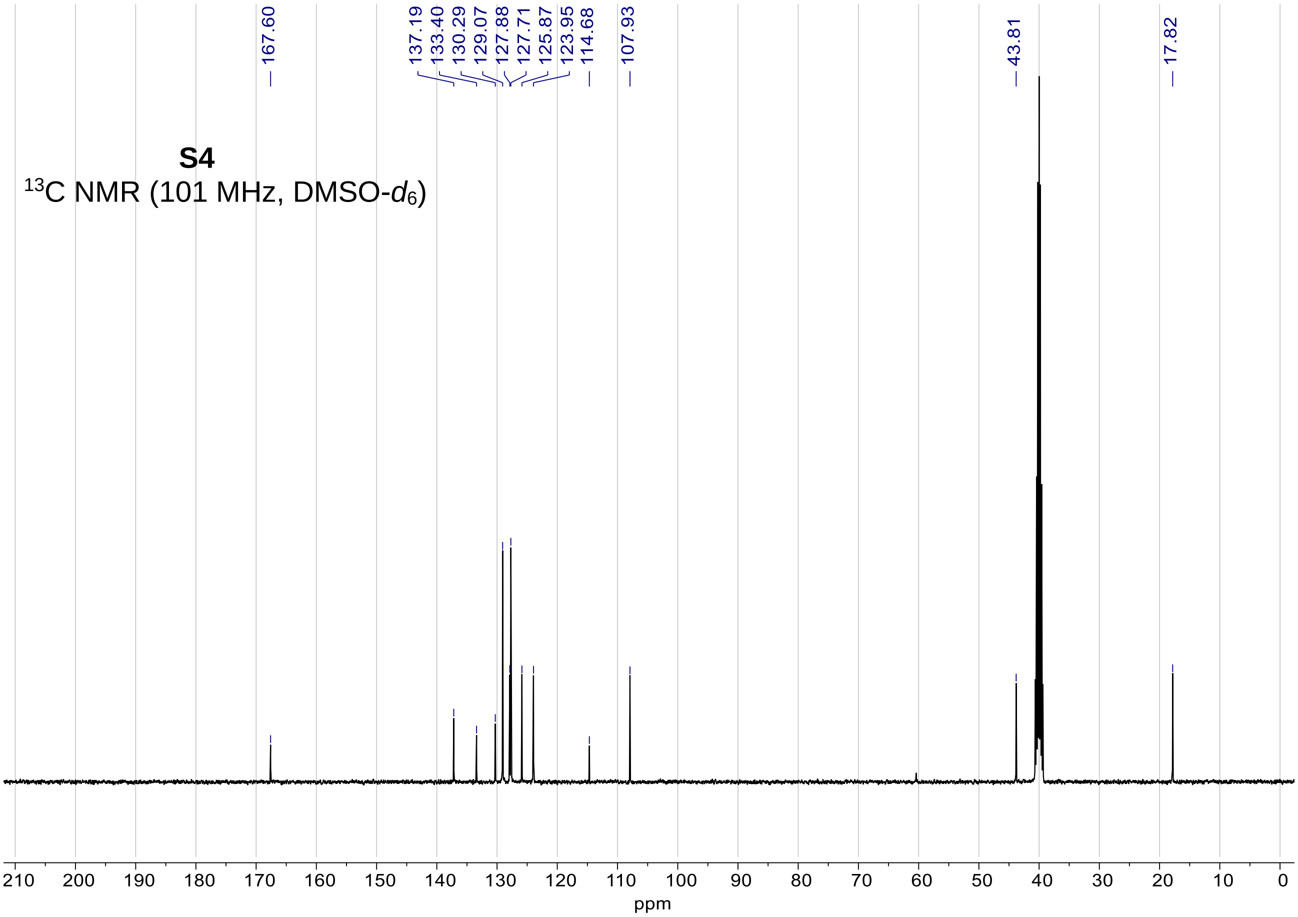
167.60
137.19
133.40
130.29
129.07
127.88
127.71
125.87
123.95
114.68
107.93
43.81
17.82
S4
13C NMR (101 MHz, DMSO-d6)
ppm

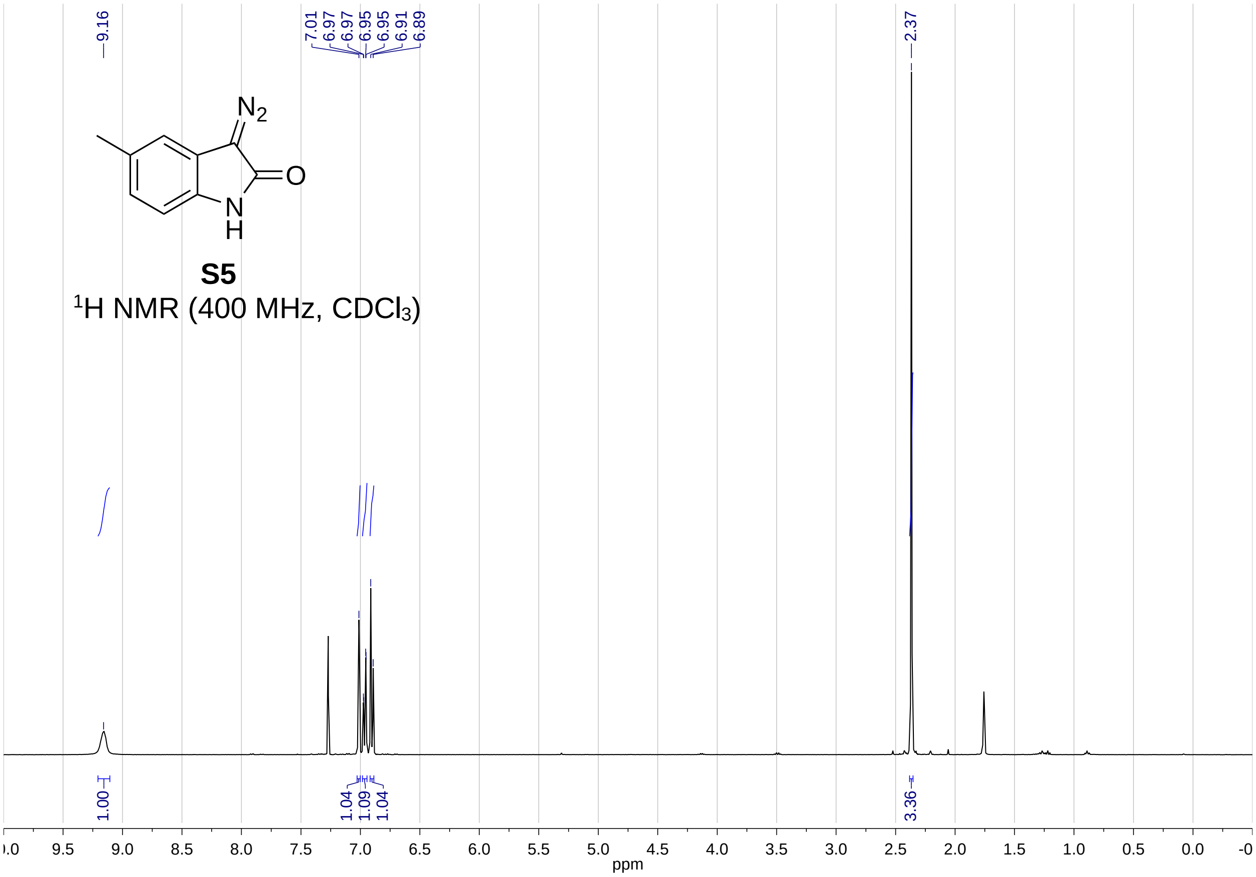

S5

^{1}H NMR (400 MHz, $CDCl_3$)

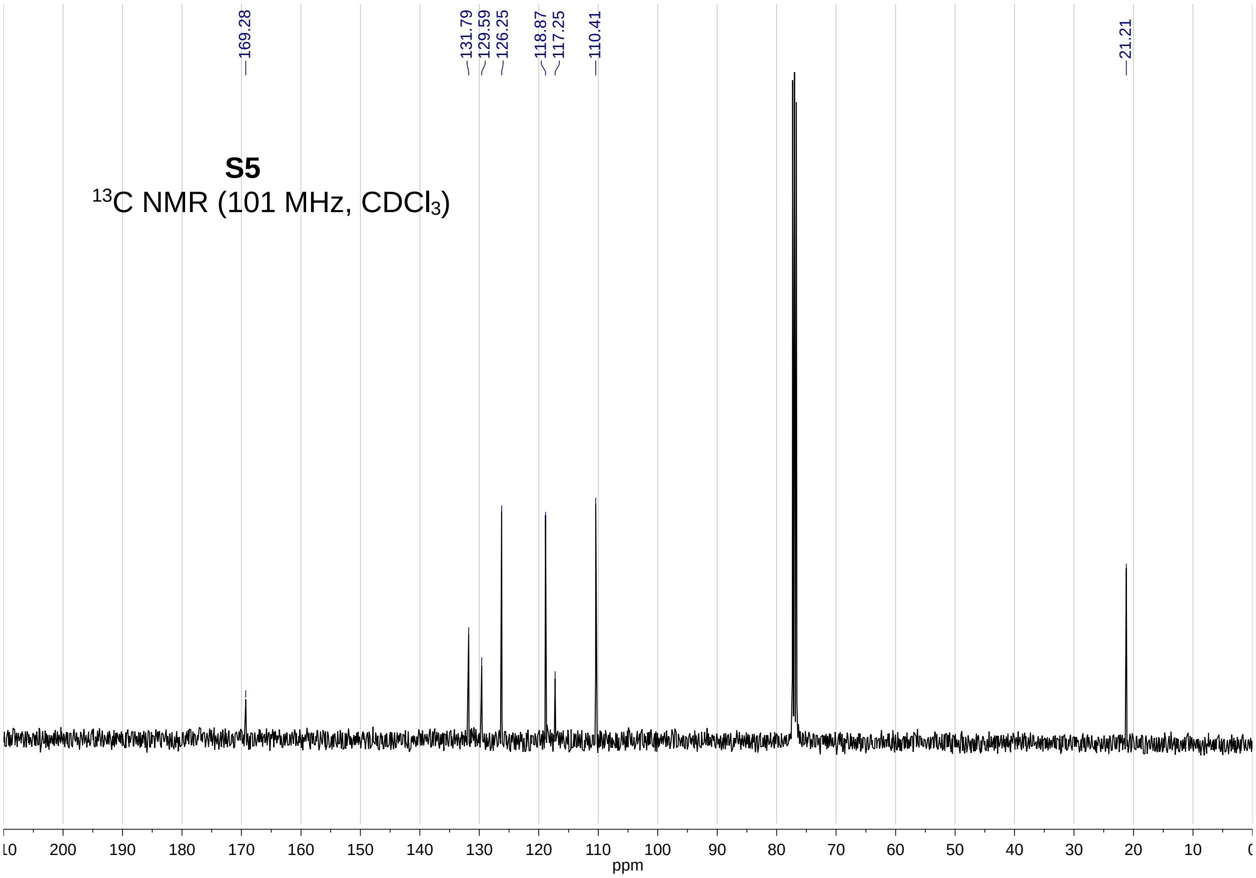

S5

^{13}C NMR (101 MHz, $CDCl_3$)

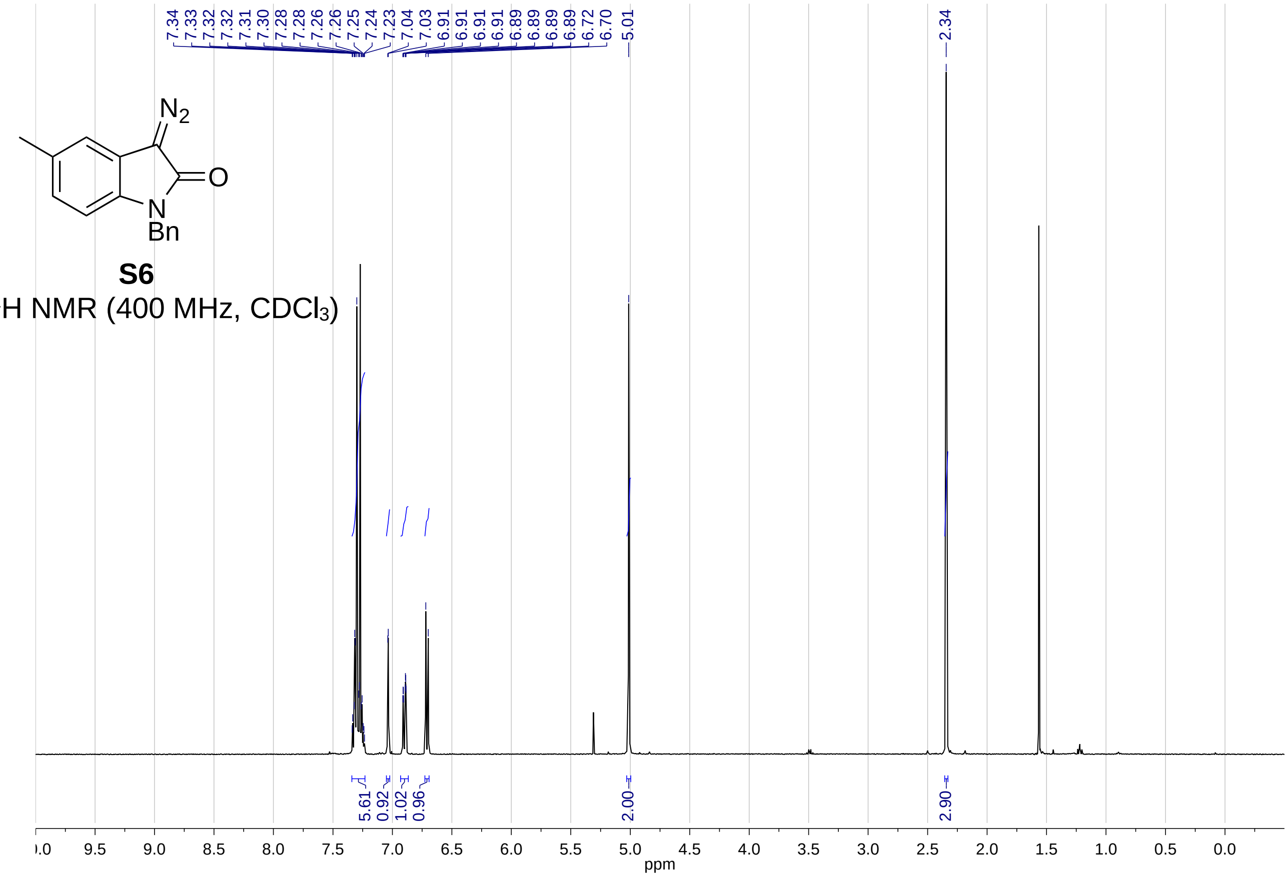

S6

^{1}H NMR (400 MHz, $CDCl_3$)

S6

^{13}C NMR (101 MHz, $CDCl_3$)

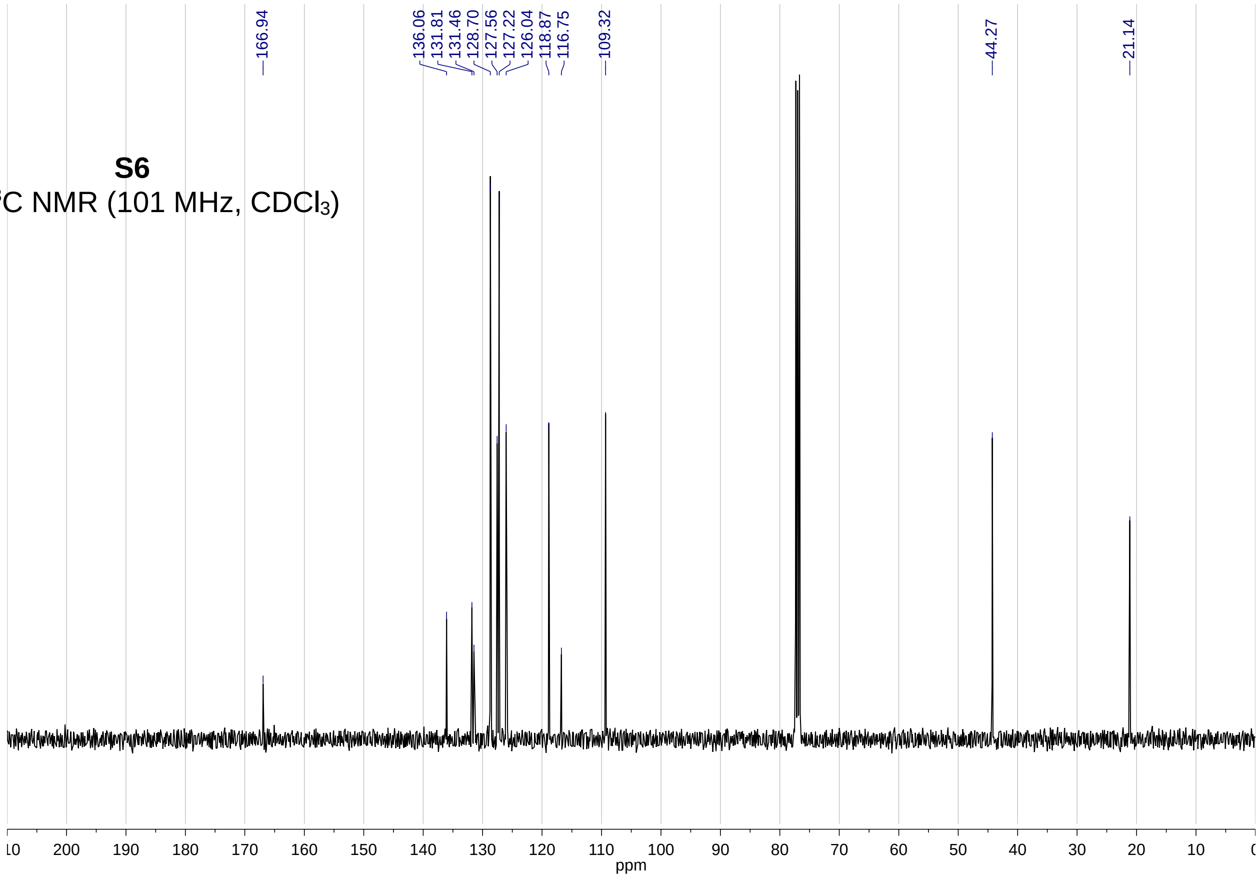

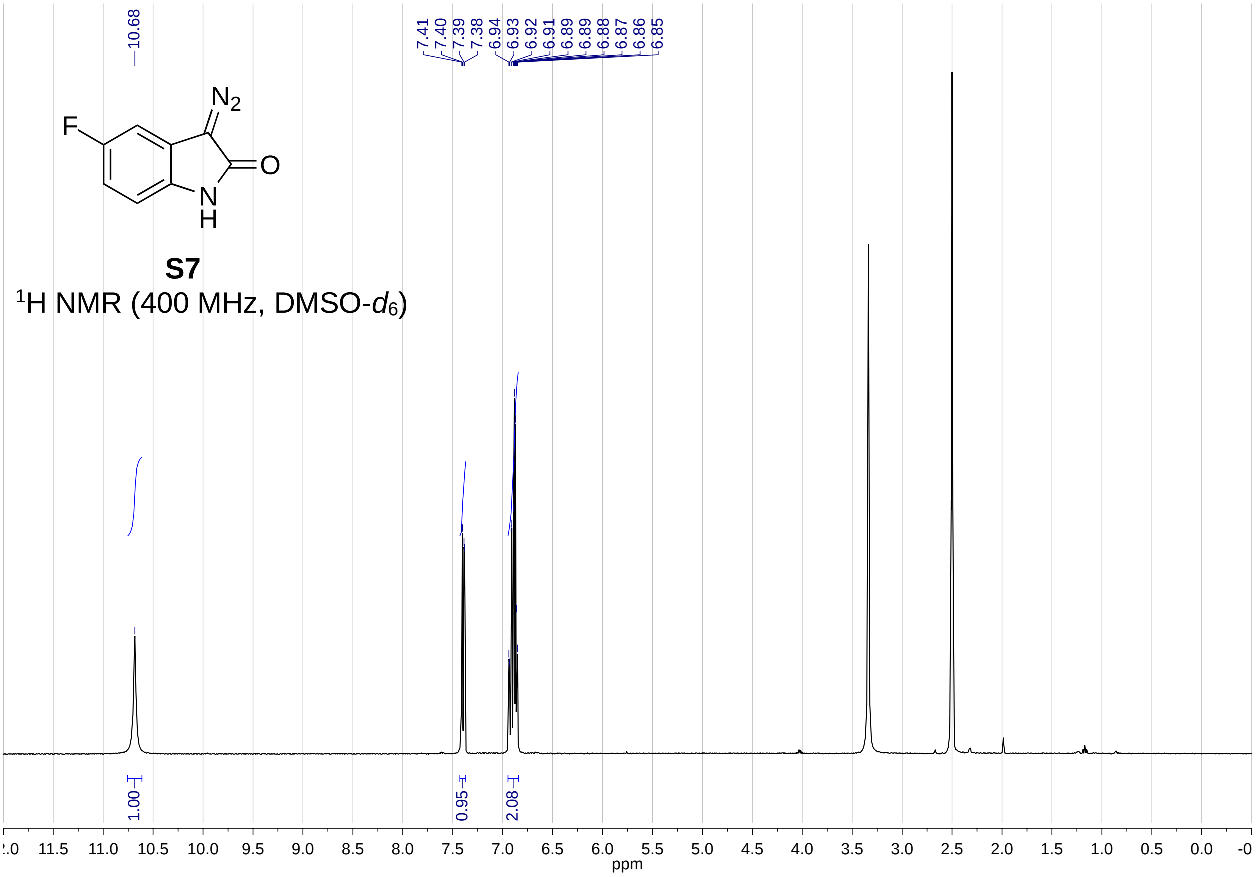
S7
1H NMR (400 MHz, DMSO-d6)
10.68
7.41
7.40
7.39
7.38
6.94
6.93
6.92
6.91
6.89
6.89
6.88
6.87
6.86
6.85
1.00
0.95
2.08
ppm

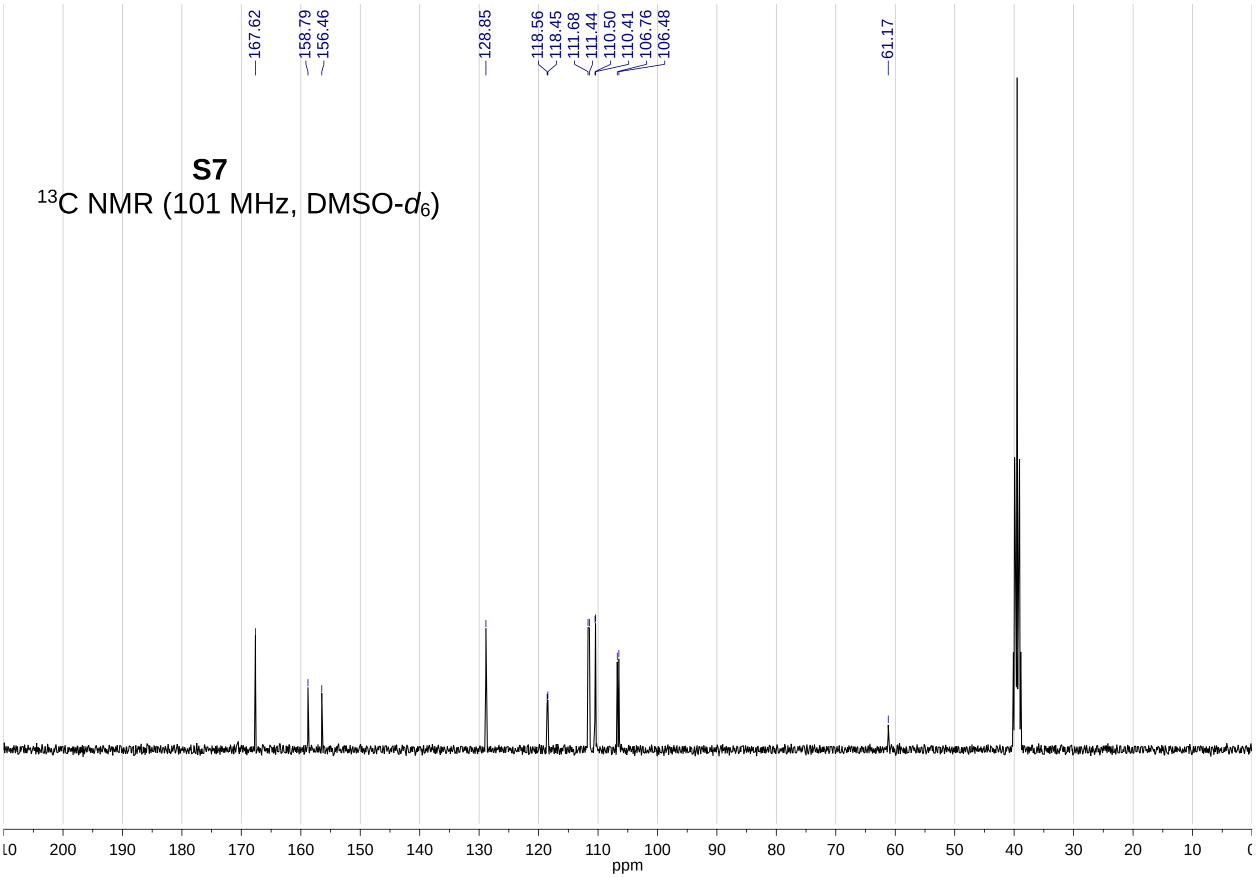
S7
13C NMR (101 MHz, DMSO-d6)
167.62
158.79
156.46
128.85
118.56
118.45
111.68
111.44
110.50
110.41
106.76
106.48
61.17
ppm

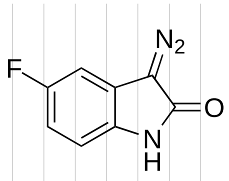

S7

^{19}F NMR (377 MHz, DMSO-d_6)

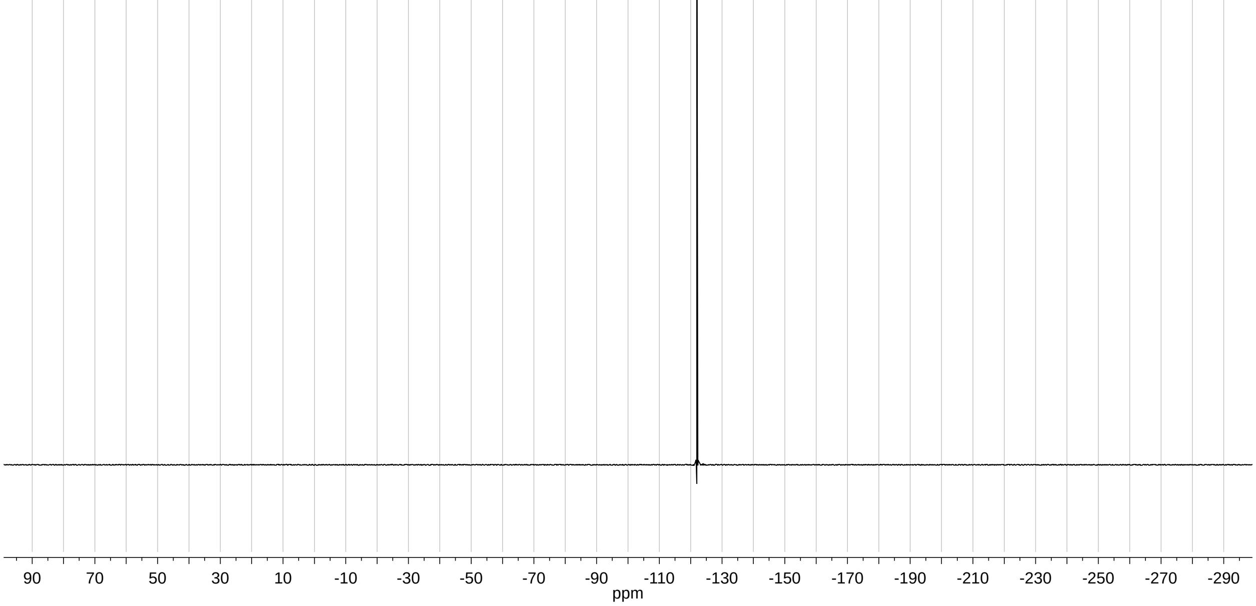

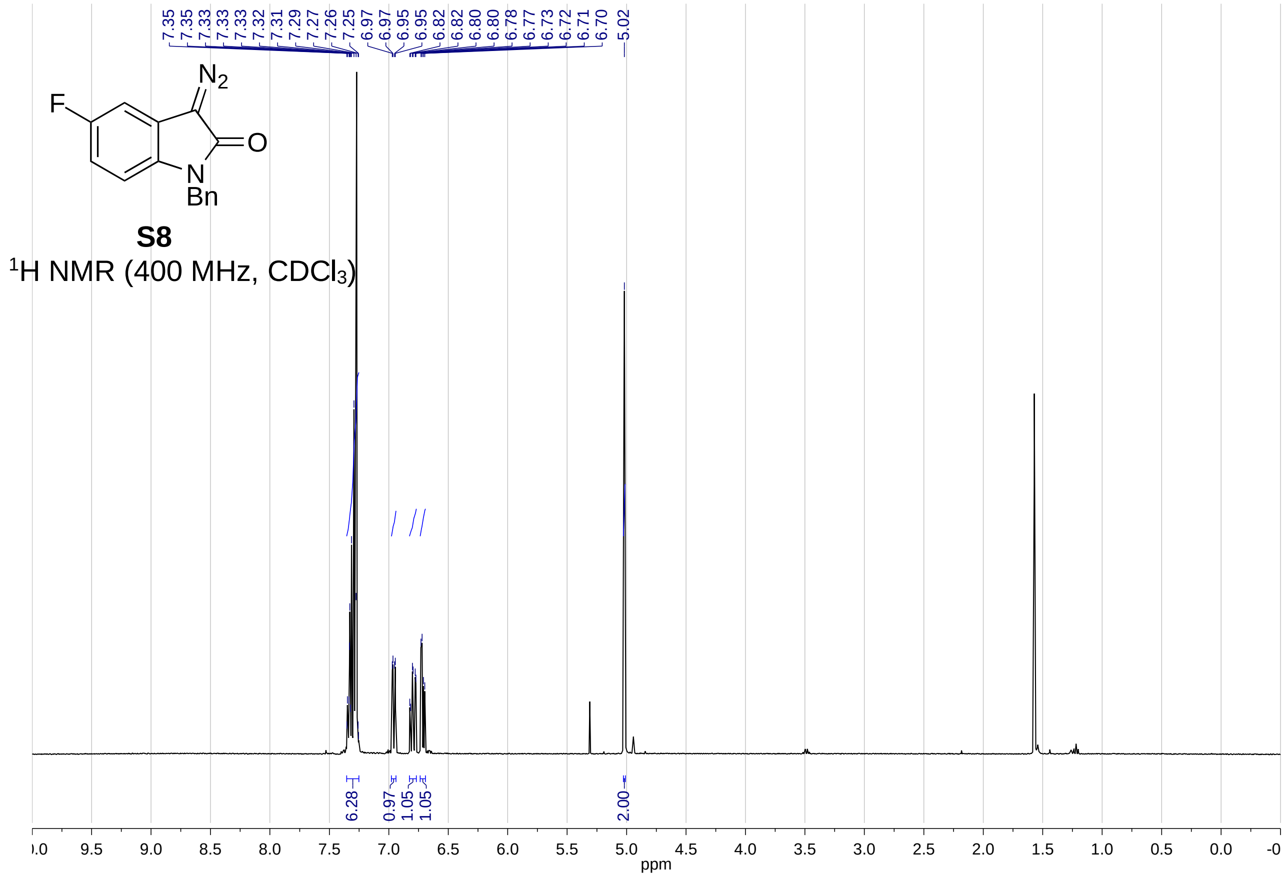
7.35
7.35
7.33
7.33
7.33
7.32
7.31
7.29
7.27
7.26
7.25
6.97
6.95
6.95
6.82
6.82
6.80
6.80
6.78
6.77
6.73
6.72
6.71
6.70
5.02
F
N2
O
N
Bn
S8
1H NMR (400 MHz, CDCl3)
6.28
0.97
1.05
1.05
2.00
ppm

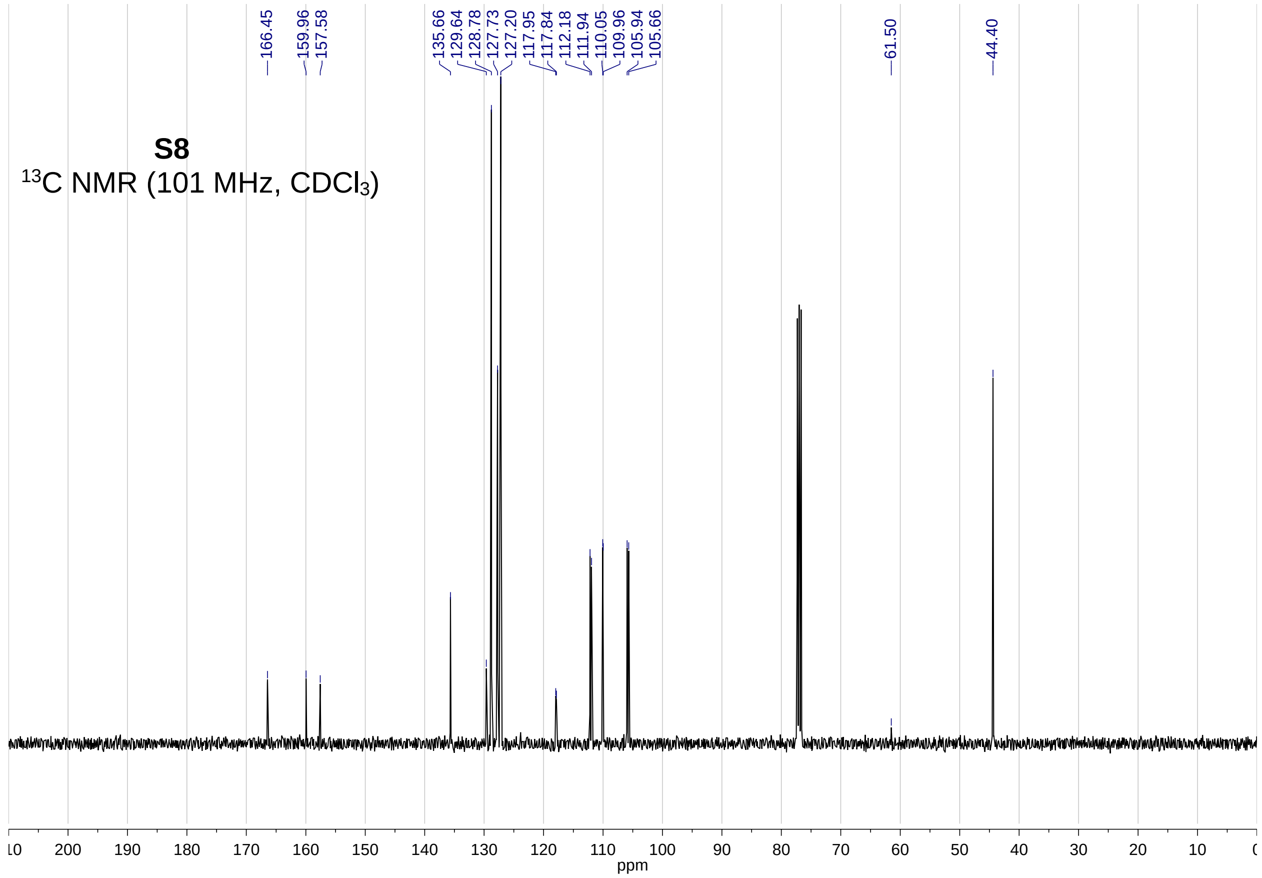
166.45
159.96
157.58
135.66
129.64
128.78
127.73
127.20
117.95
117.84
112.18
111.94
110.05
109.96
105.94
105.66
61.50
44.40
S8
13C NMR (101 MHz, CDCl3)
ppm

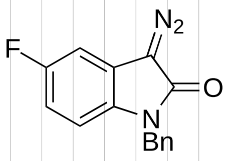

S8

^{19}F NMR (377 MHz, $CDCl_3$)

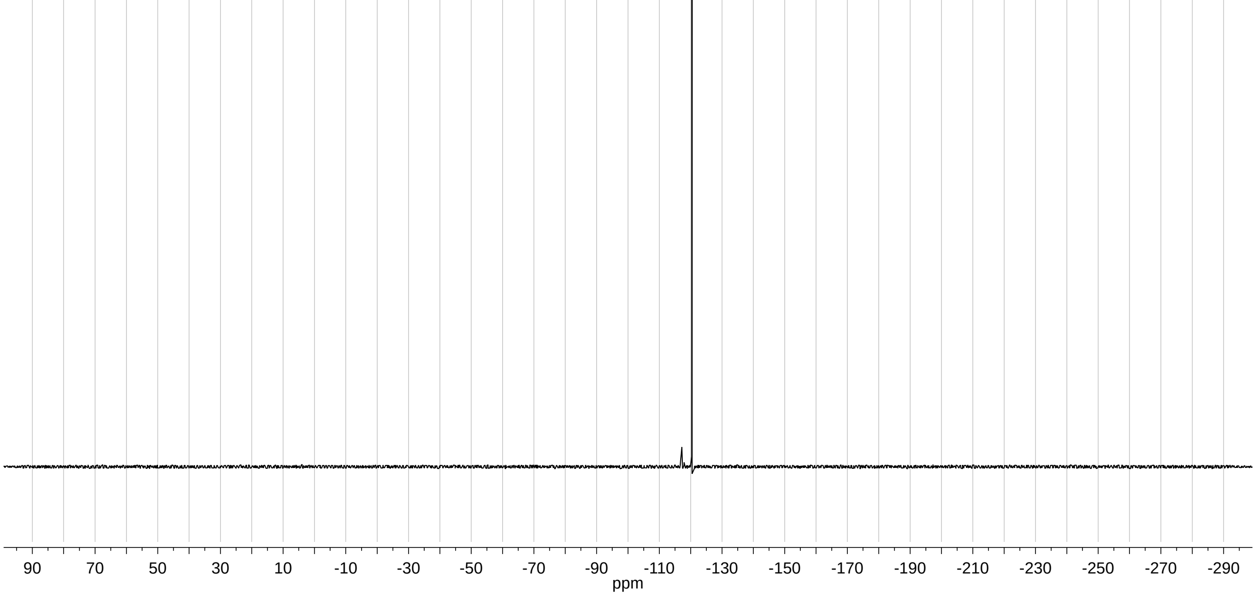

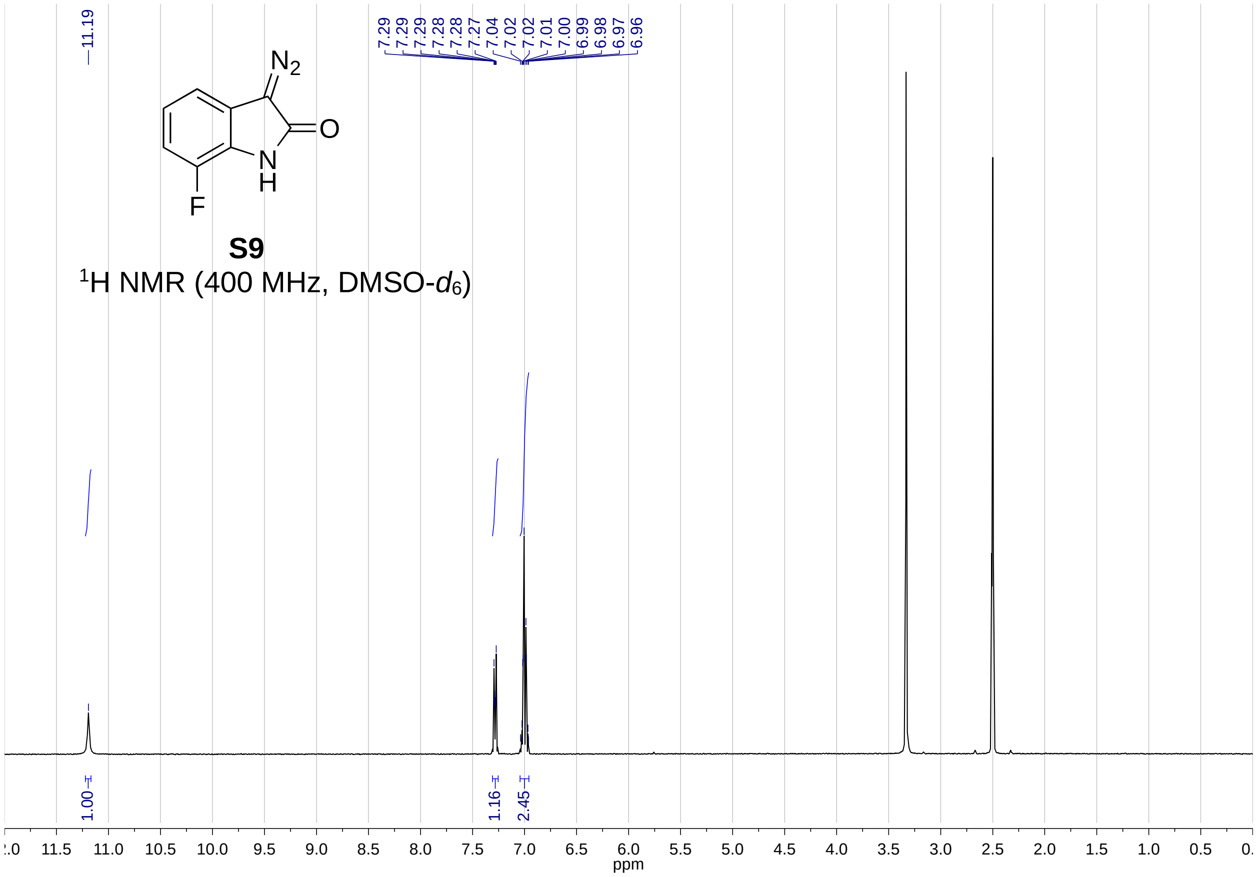
11.19
7.29
7.29
7.29
7.28
7.28
7.27
7.04
7.02
7.02
7.01
7.00
6.99
6.98
6.97
6.96
N2
O
N
H
F
S9
1H NMR (400 MHz, DMSO-d6)
1.00
1.16
2.45
ppm

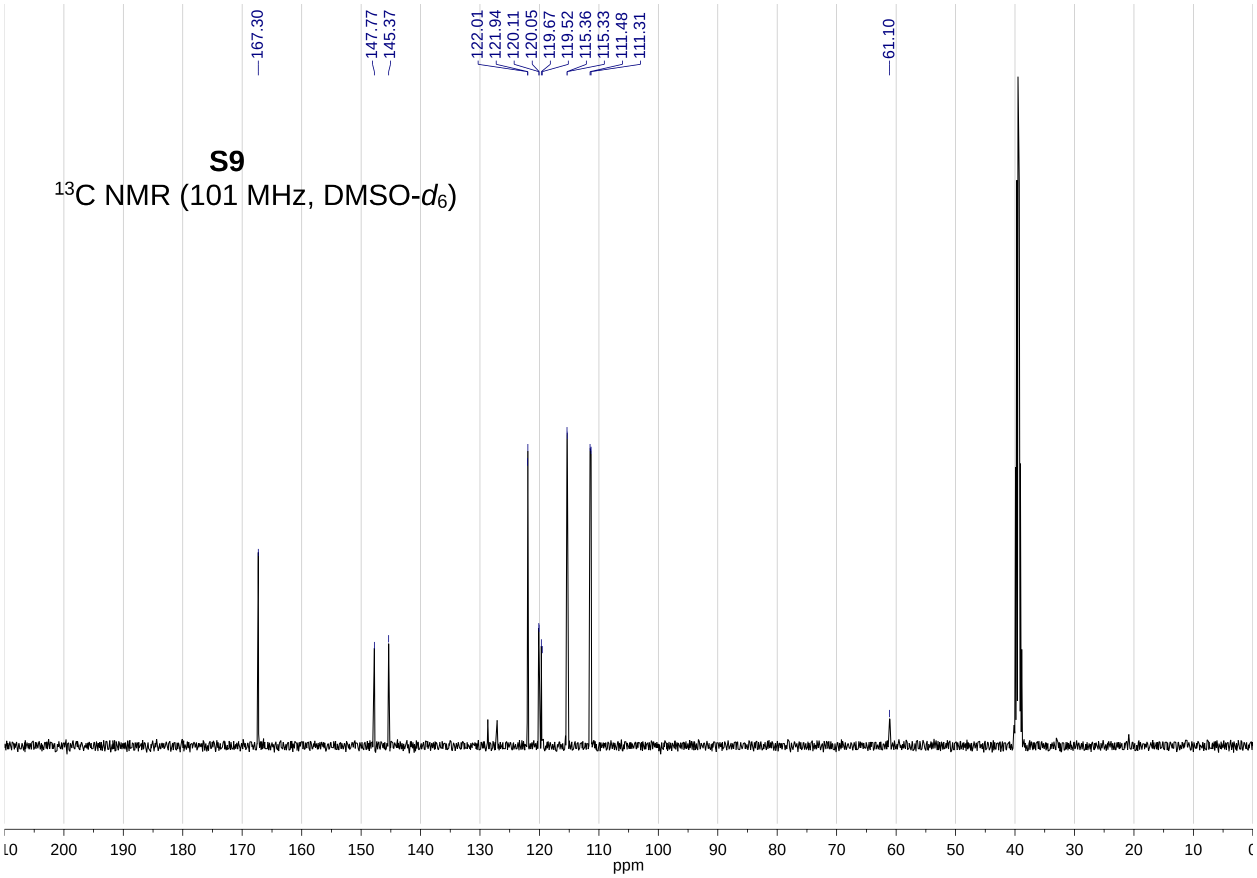
167.30
147.77
145.37
122.01
121.94
120.11
120.05
119.67
119.52
115.36
115.33
111.48
111.31
61.10
S9
13C NMR (101 MHz, DMSO-d6)
ppm

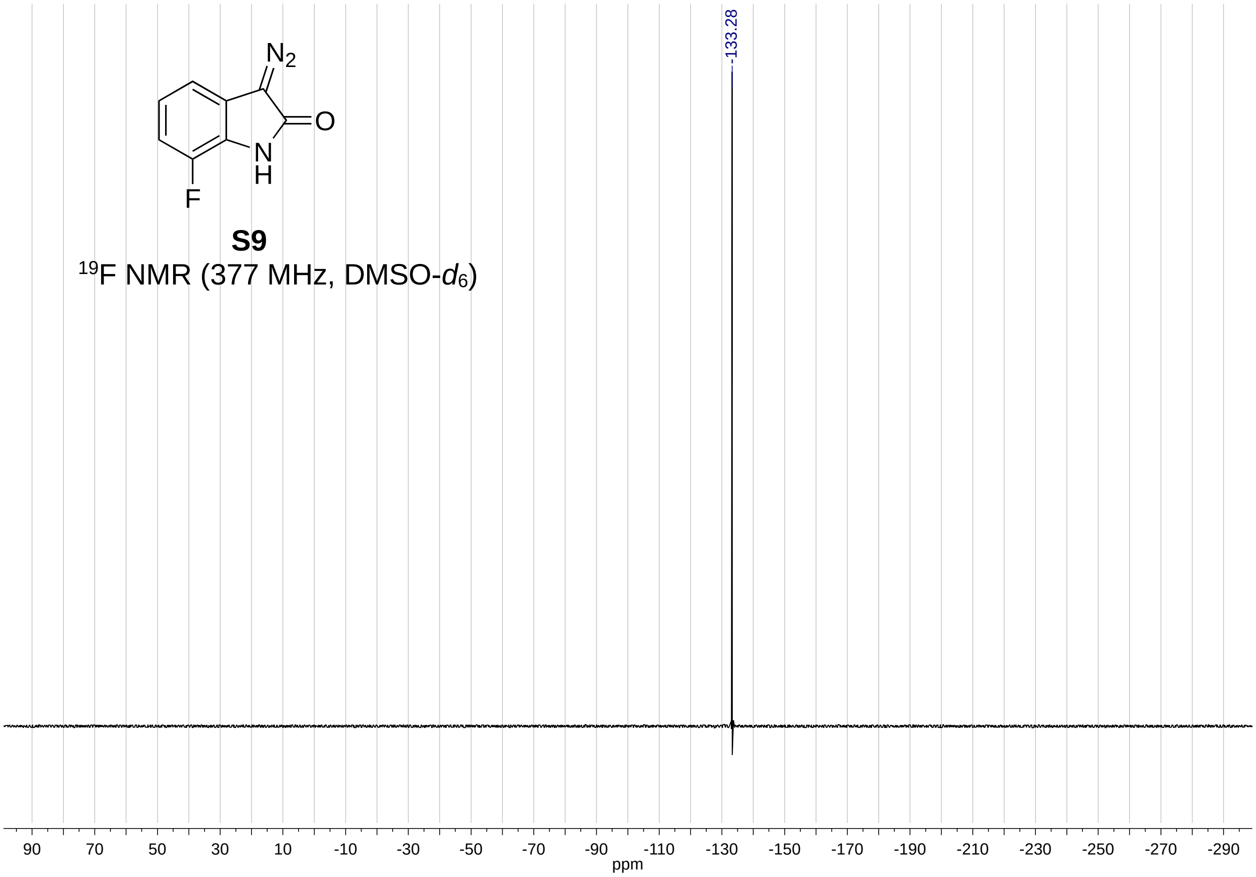

S9

^{19}F NMR (377 MHz, DMSO-d_6)

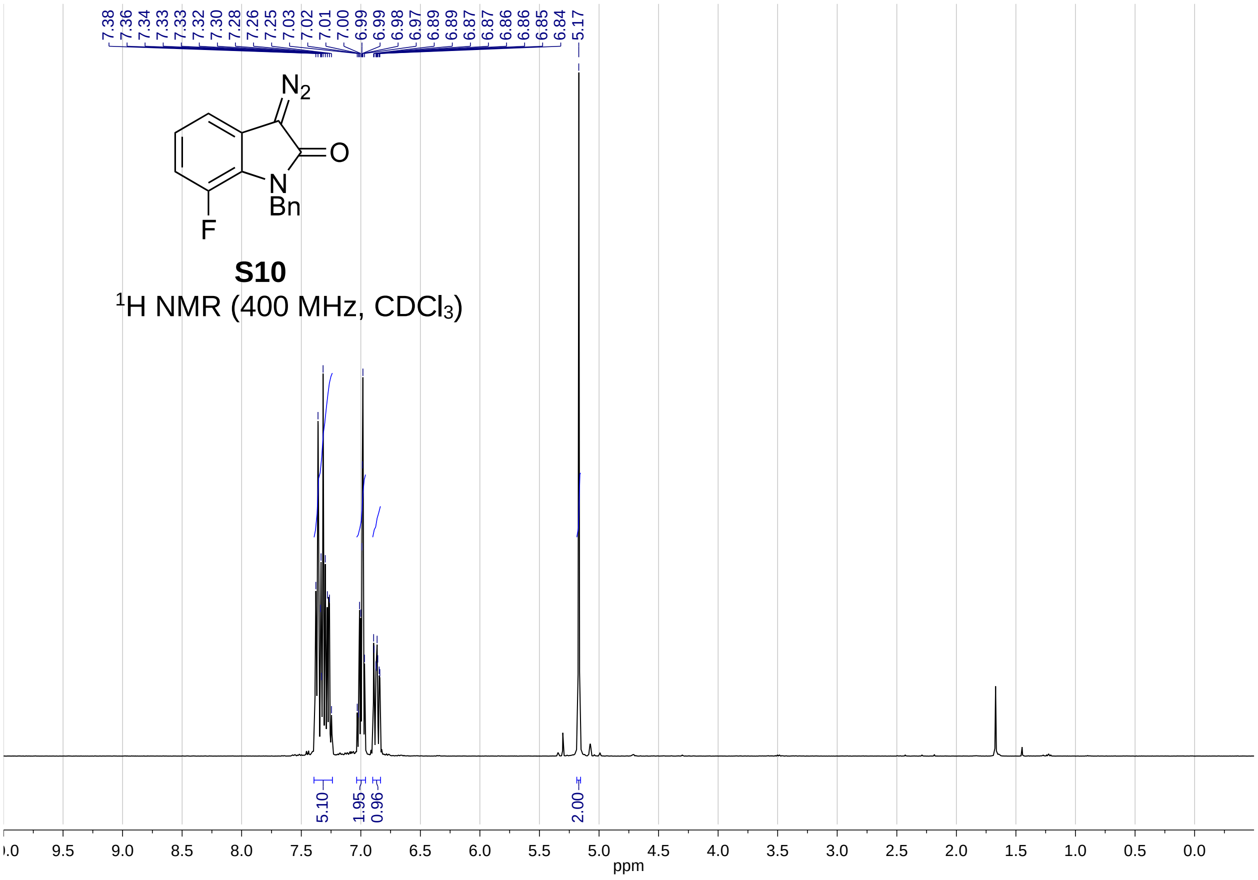
7.38
7.36
7.34
7.33
7.33
7.32
7.30
7.28
7.26
7.25
7.03
7.02
7.01
7.00
6.99
6.99
6.98
6.97
6.89
6.89
6.87
6.87
6.86
6.86
6.85
6.84
5.17
N2
O
N
Bn
F
S10
¹H NMR (400 MHz, CDCl3)
5.10
1.95
0.96
2.00
ppm

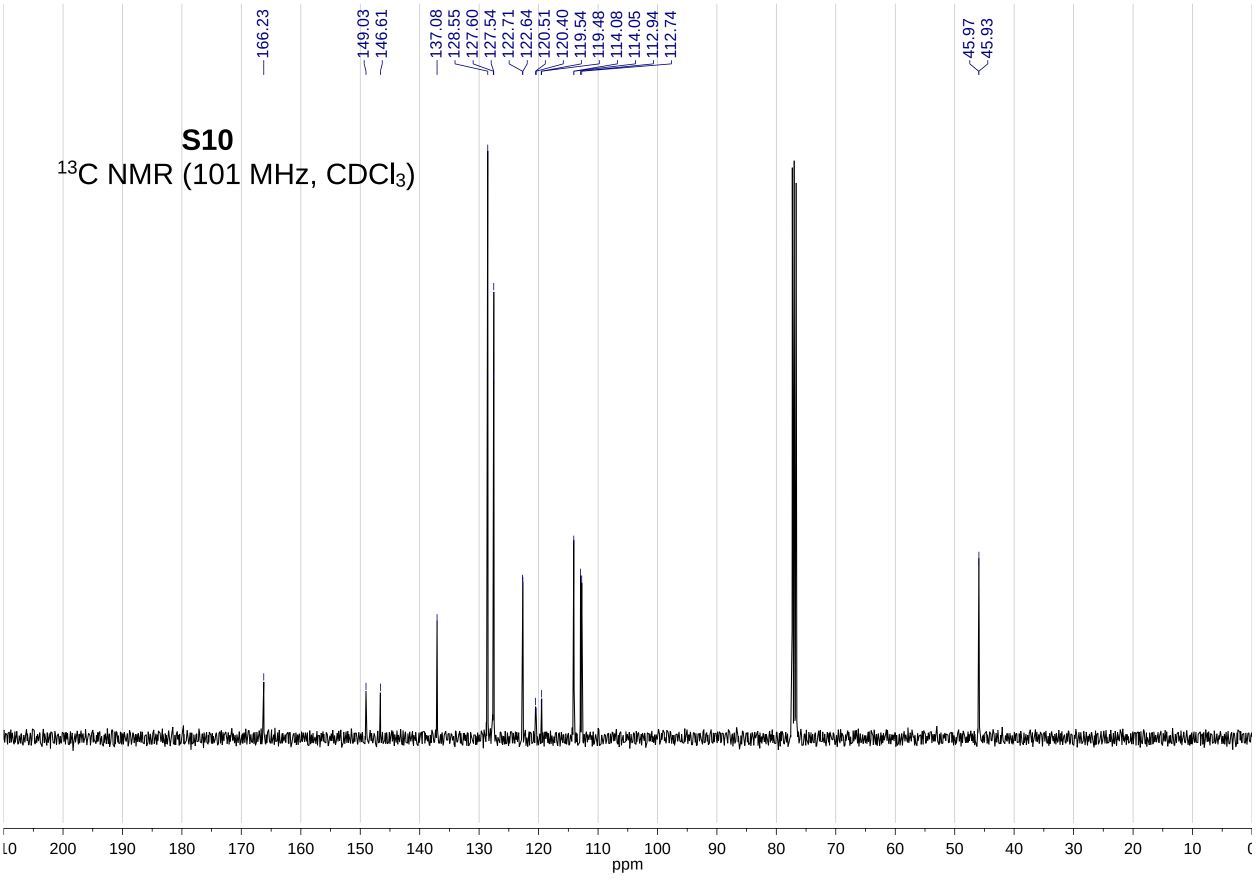
166.23
149.03
146.61
137.08
128.55
127.60
127.54
122.71
122.64
120.51
120.40
119.54
119.48
114.08
114.05
112.94
112.74
45.97
45.93
S10
¹³C NMR (101 MHz, CDCl3)
ppm

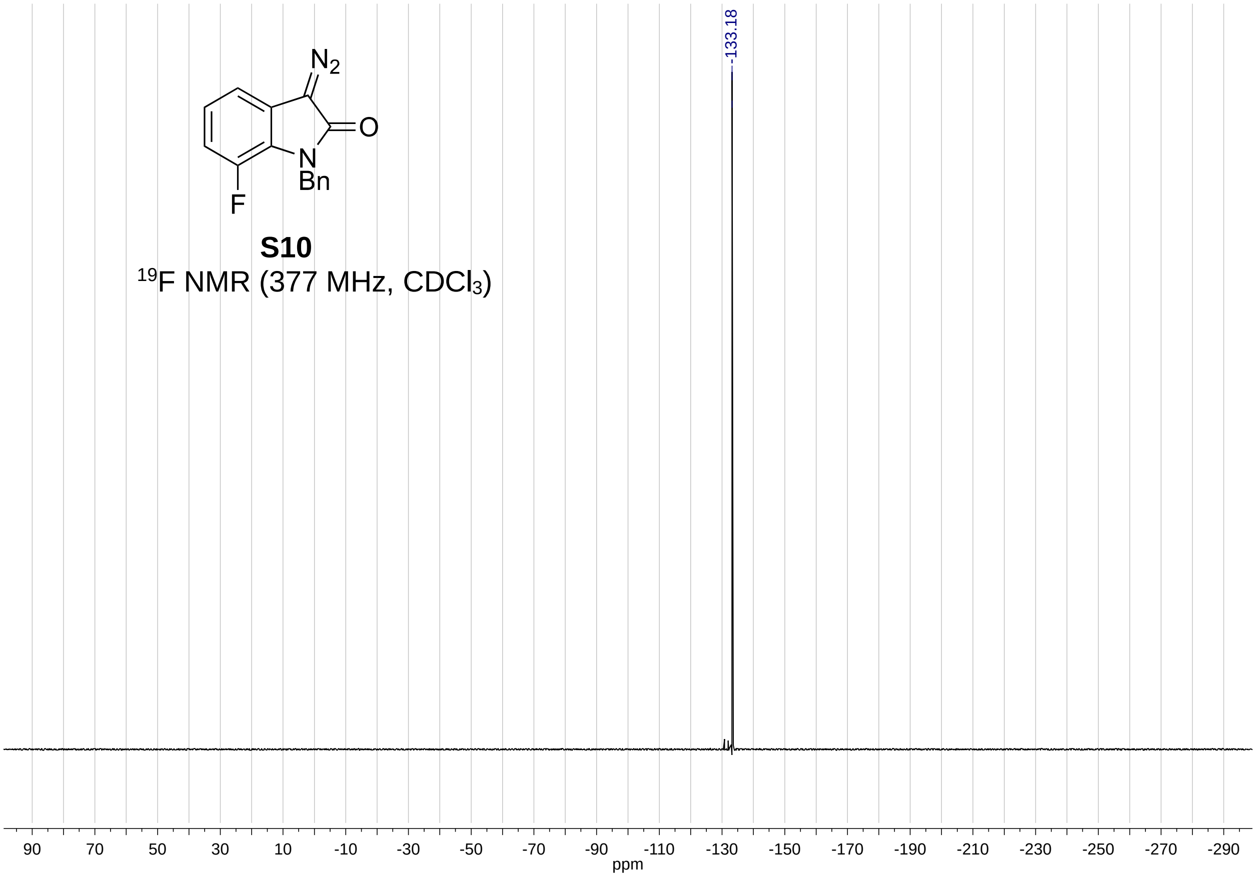

S10

^{19}F NMR (377 MHz, $CDCl_3$)

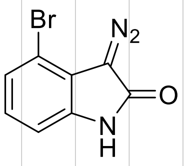

S11

^{1}H NMR (400 MHz, DMSO-d_6)

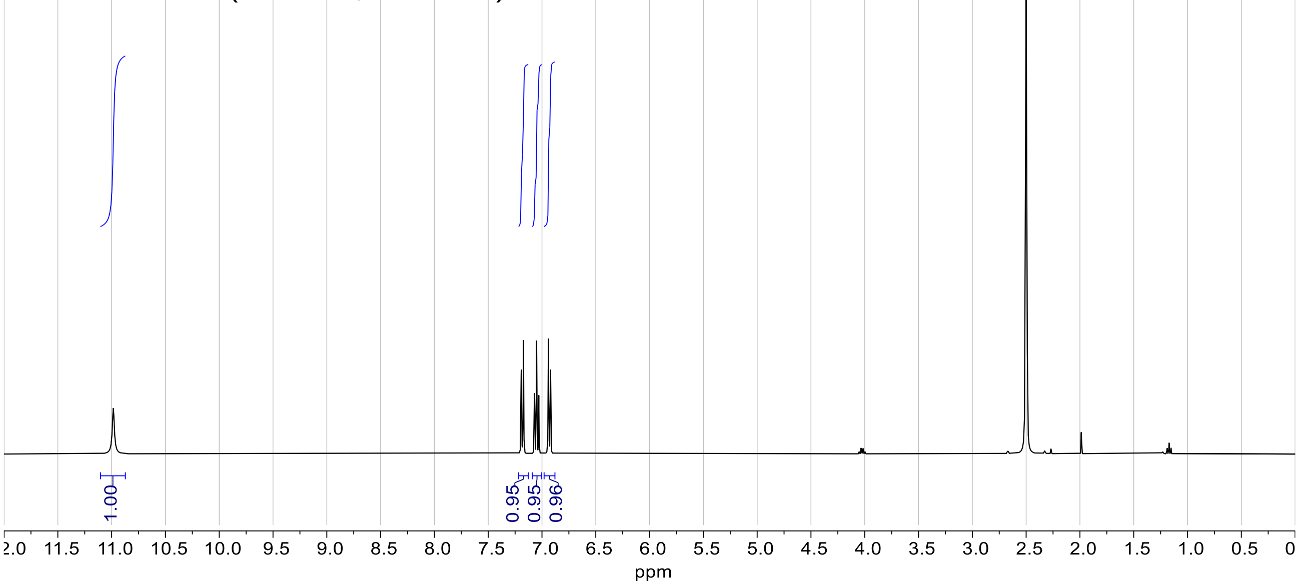

S11

^{13}C NMR (101 MHz, DMSO-d_6)

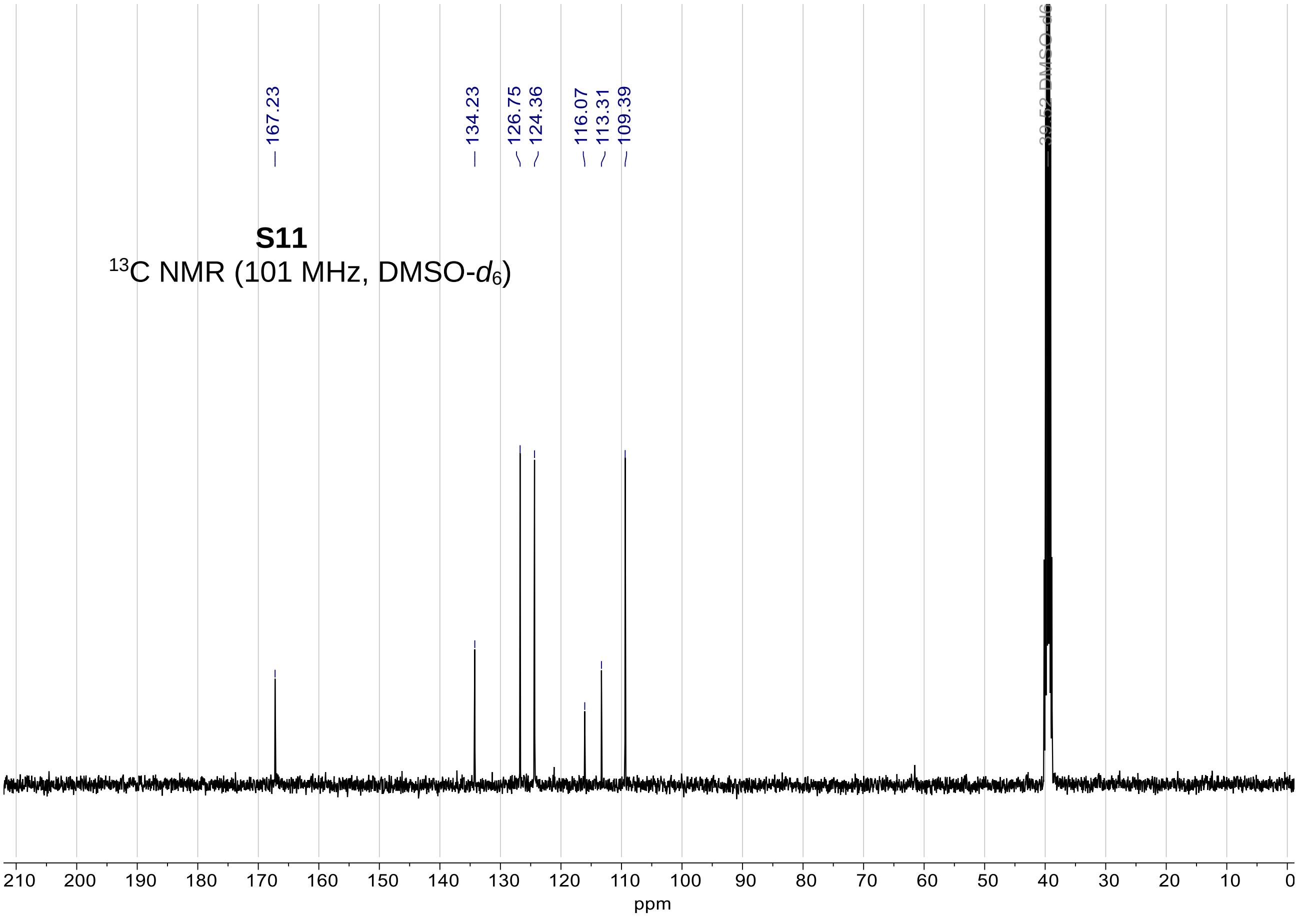

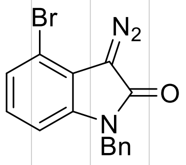

S12
^{1}H NMR (400 MHz, $CDCl_3$)

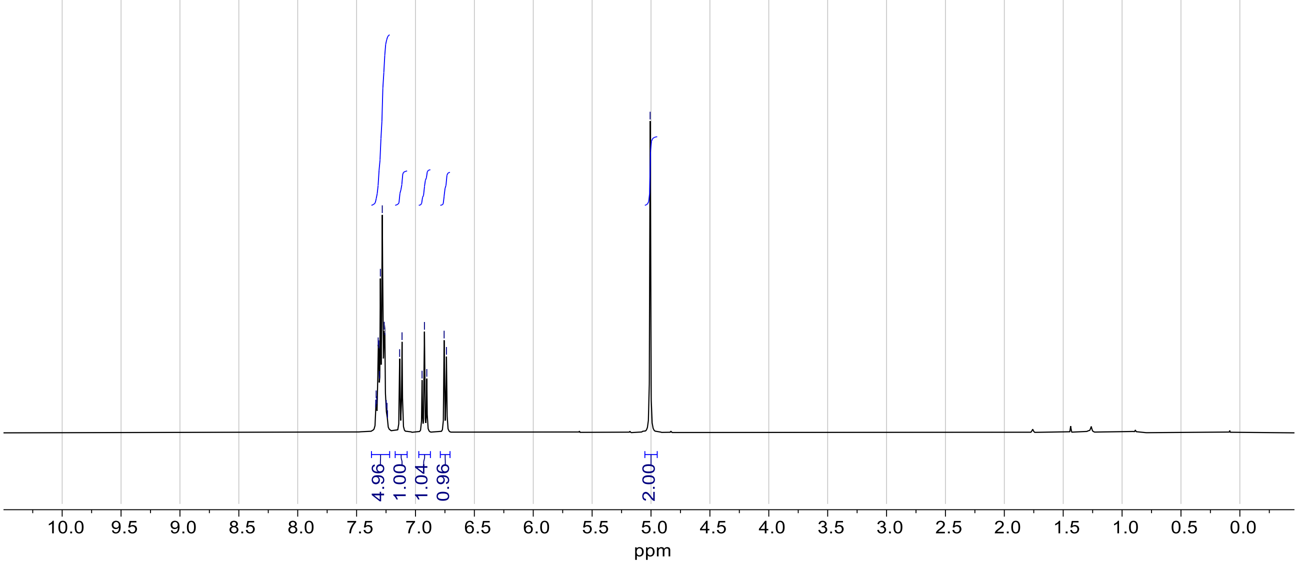

S12
^{13}C NMR (101 MHz, $CDCl_3$)

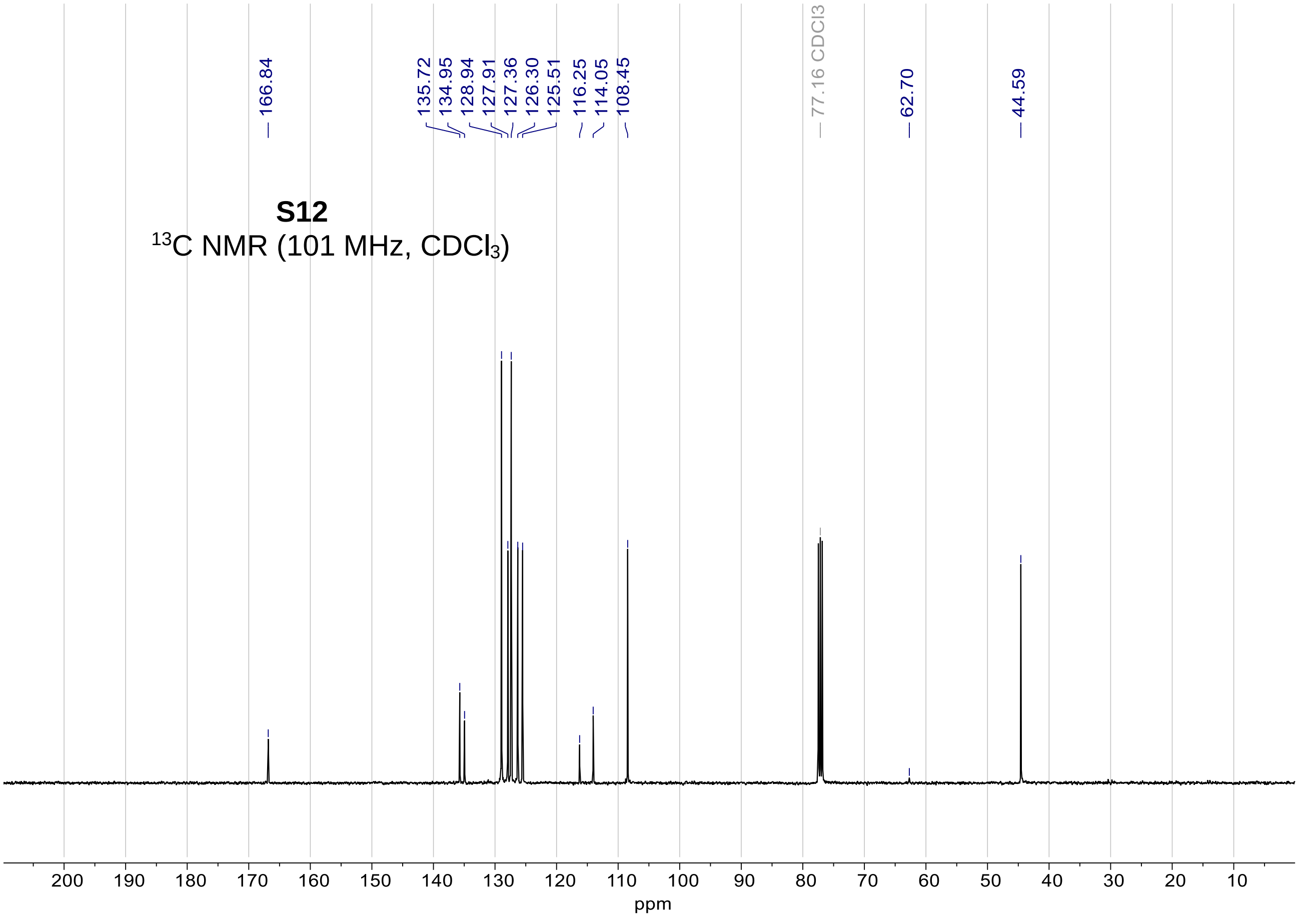

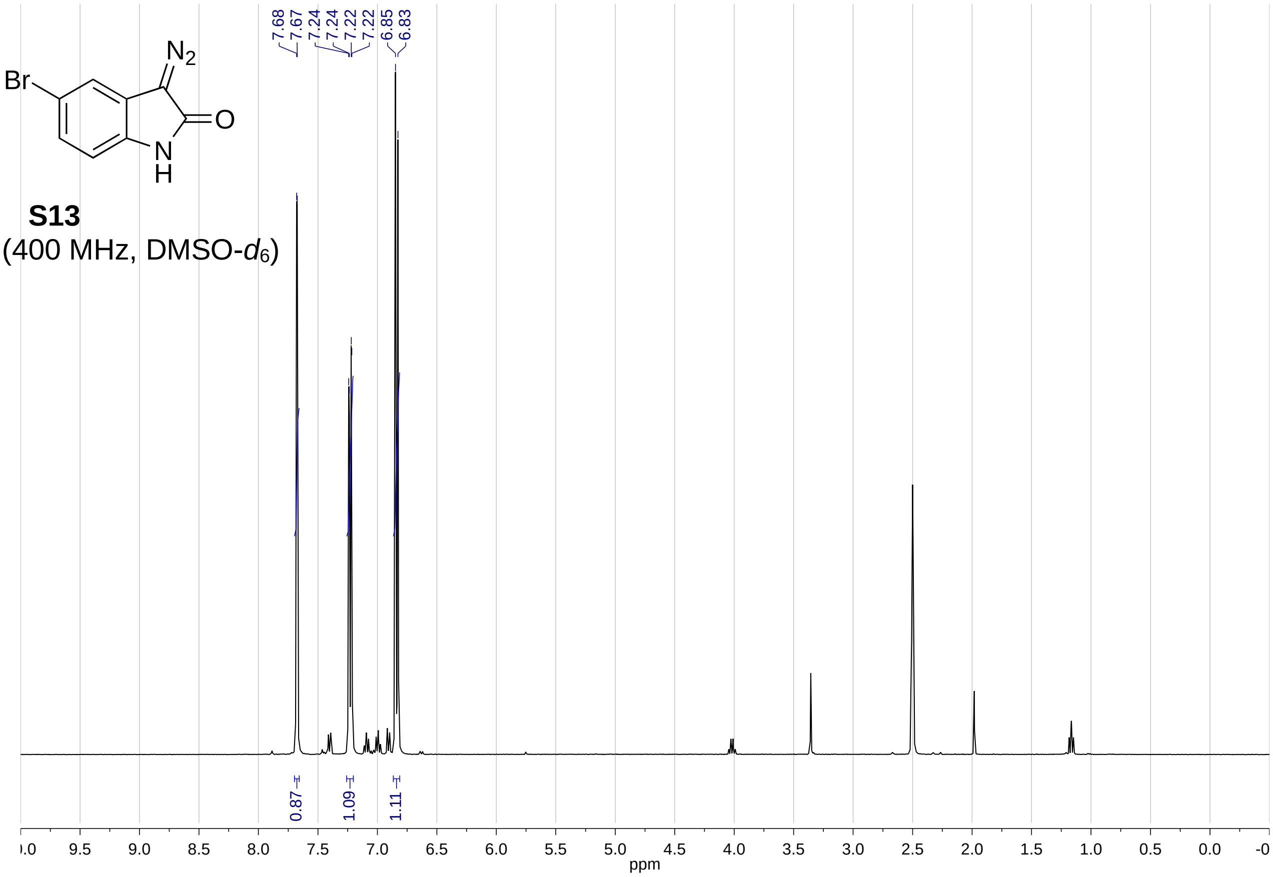

S13

^{1}H NMR (400 MHz, DMSO-d_6)

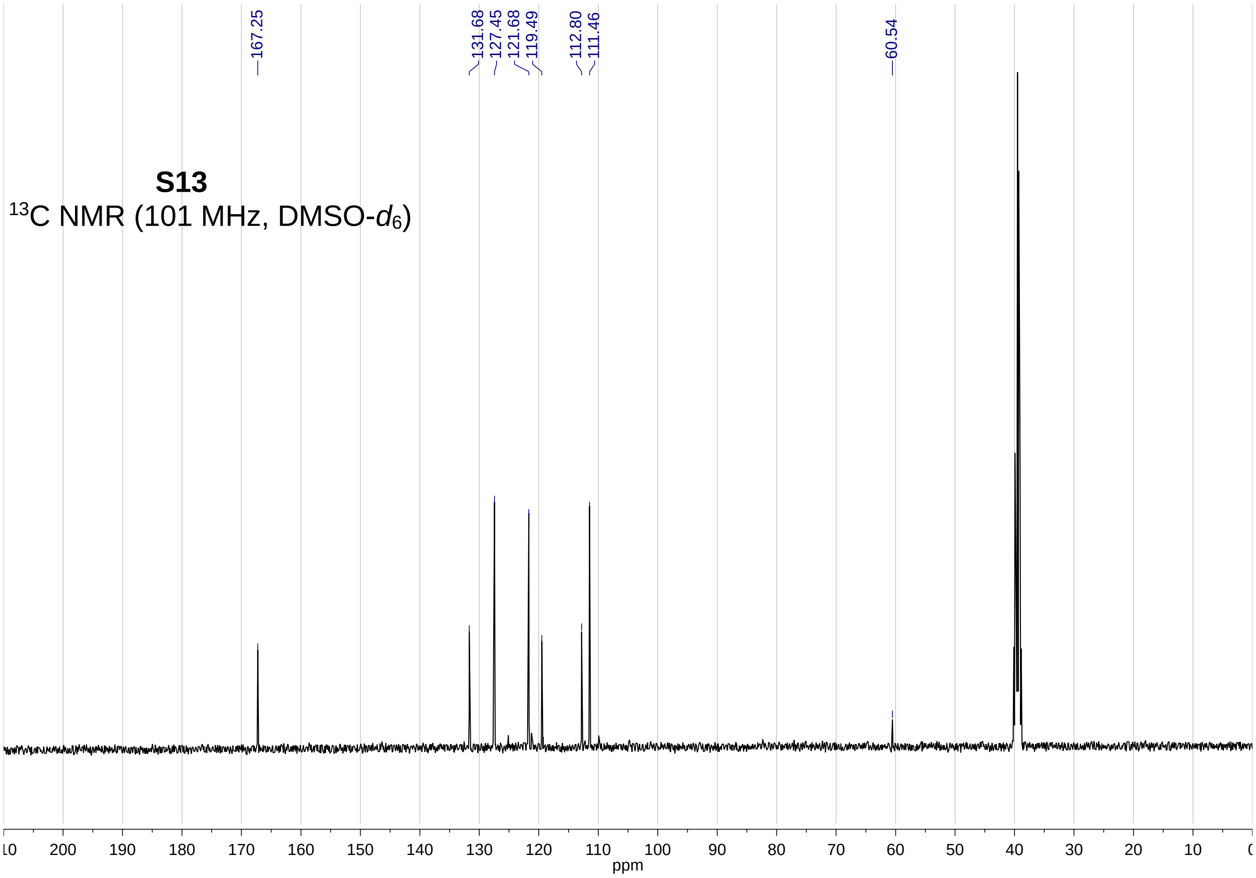

S13

^{13}C NMR (101 MHz, DMSO-d_6)

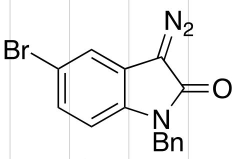

S14

^{1}H NMR (400 MHz, $CDCl_3$)

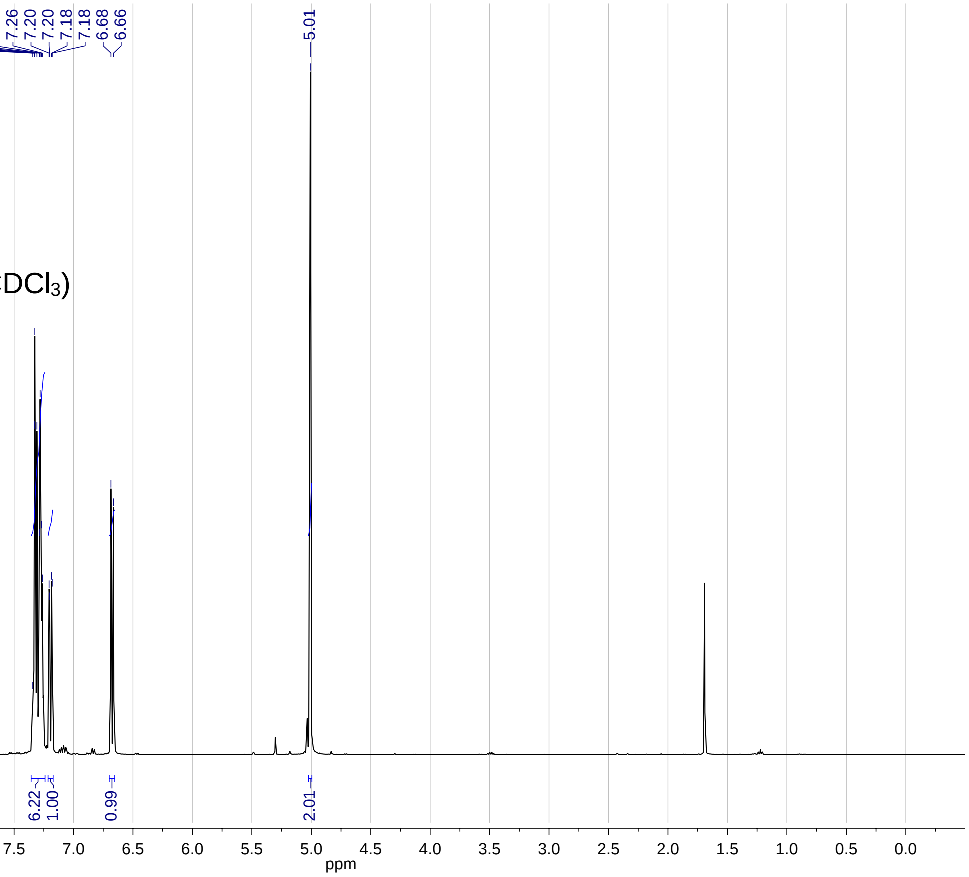

S14

^{13}C NMR (101 MHz, $CDCl_3$)

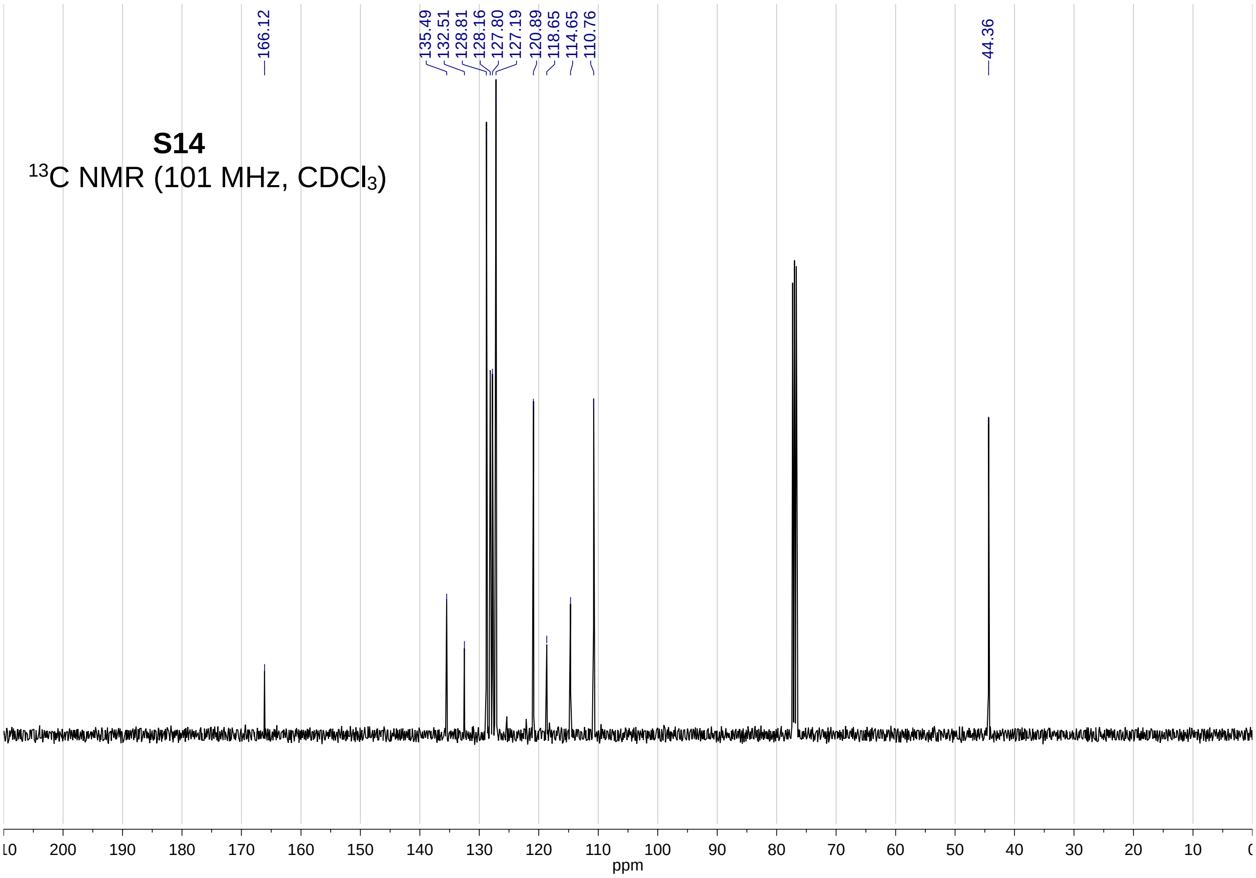

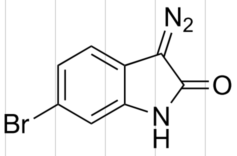

S15

^1H NMR (400 MHz, DMSO-d^6)

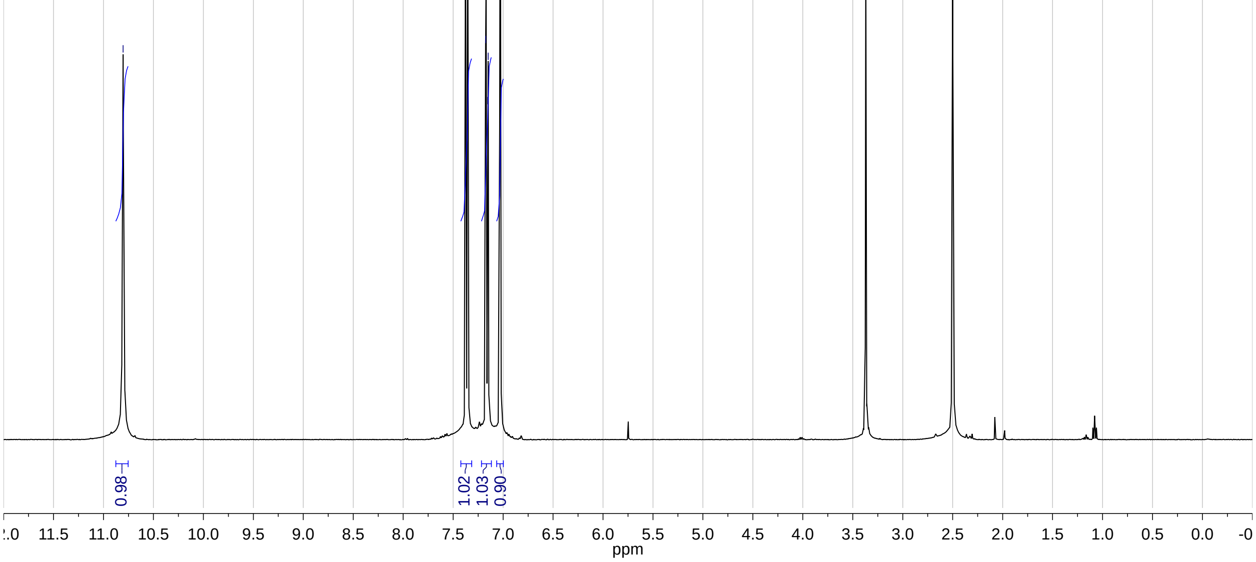

S16

^{13}C NMR (101 MHz, DMSO-d^6)

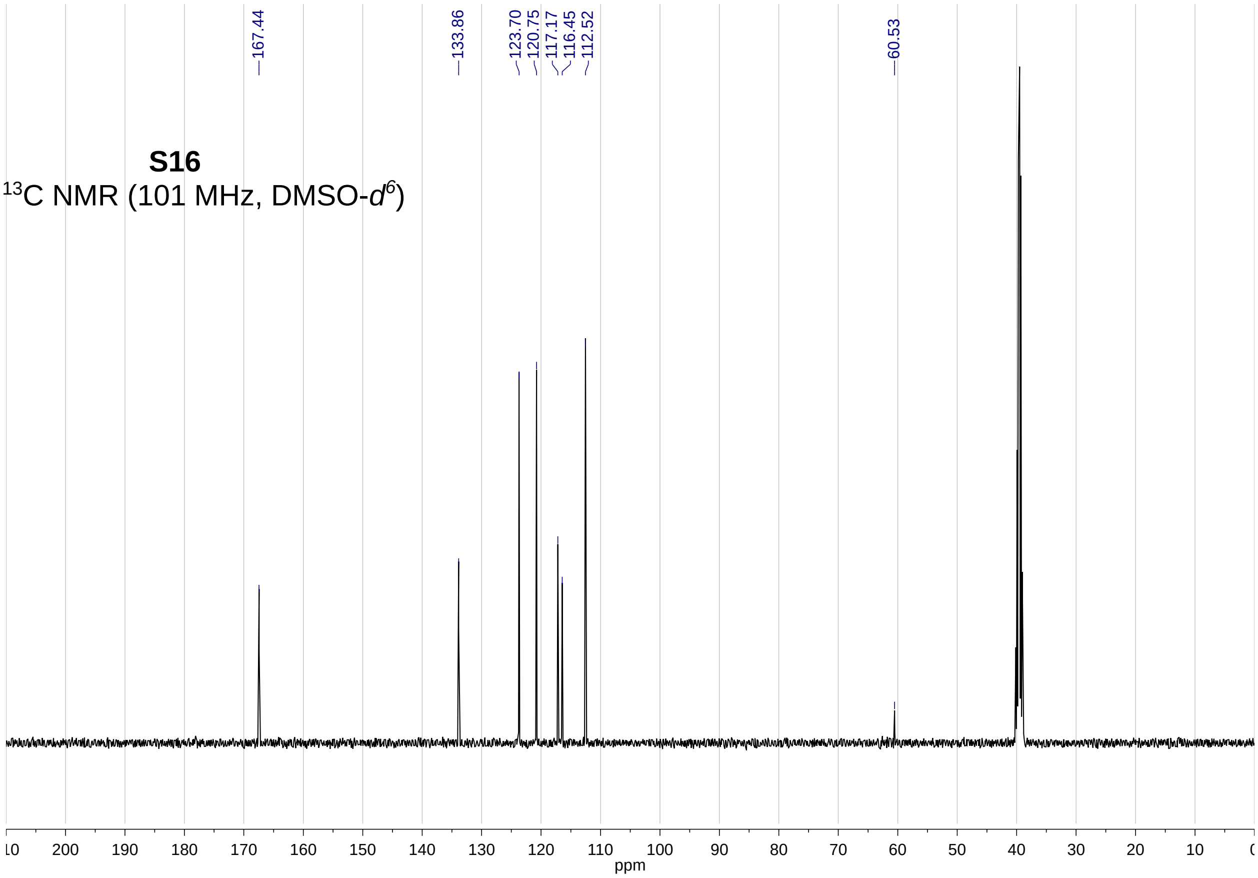

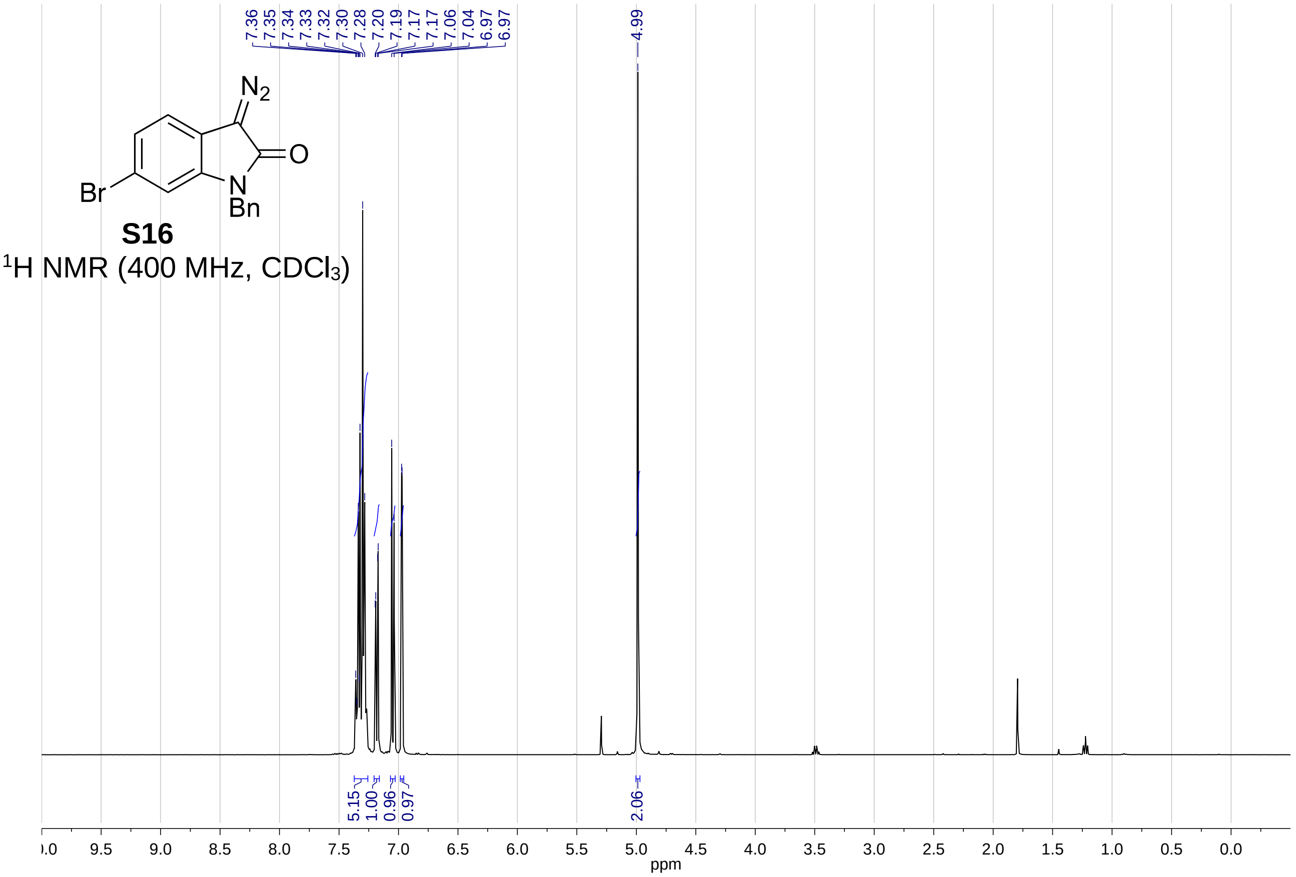
7.36 7.35 7.34 7.33 7.32 7.30 7.28 7.20 7.19 7.17 7.17 7.06 7.04 6.97 6.97
4.99
N2
O
N
Br
Bn
S16
1H NMR (400 MHz, CDCl3)
5.15 1.00 0.96 0.97
2.06
ppm

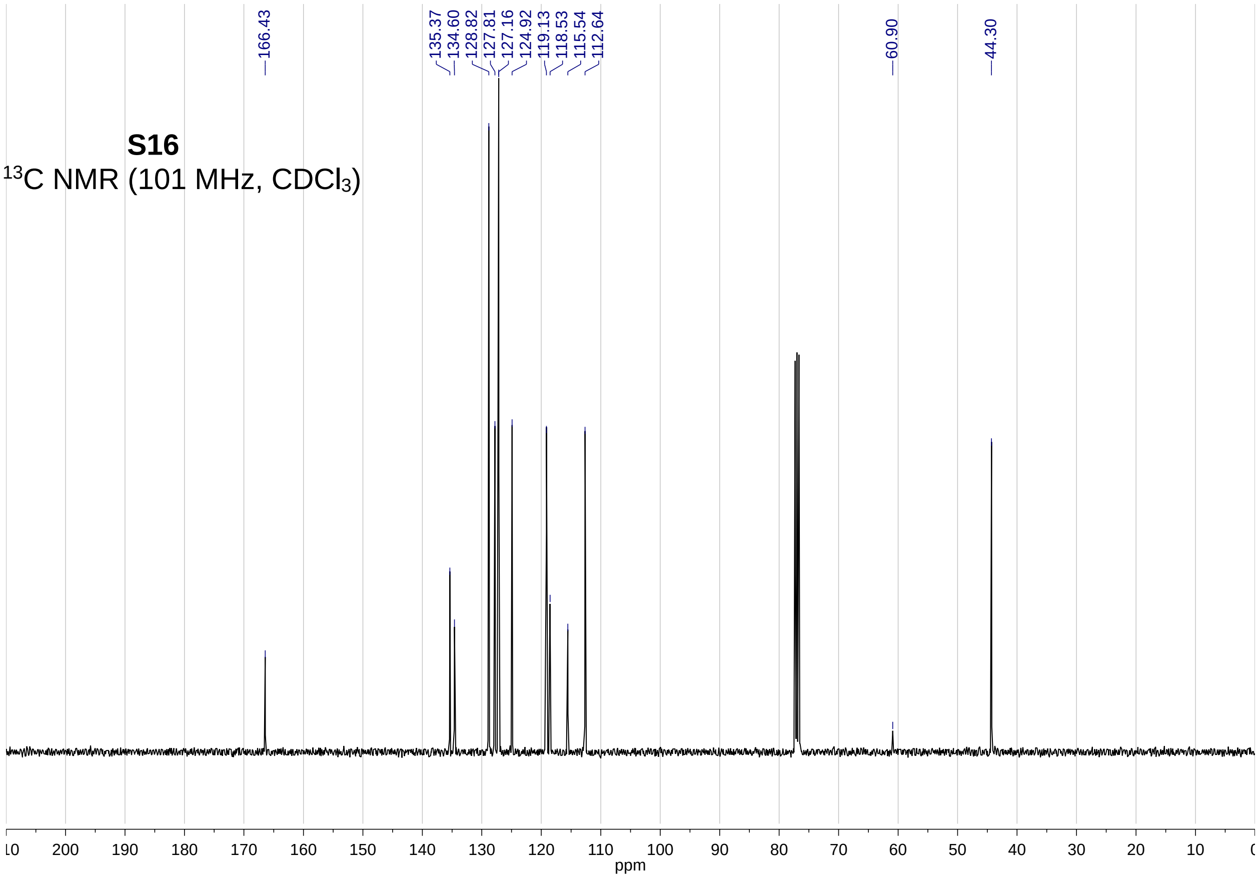
166.43
135.37 134.60 128.82 127.81 127.16 124.92 119.13 118.53 115.54 112.64
60.90
44.30
S16
13C NMR (101 MHz, CDCl3)
ppm

S17

^{1}H NMR (400 MHz, DMSO-d_6)

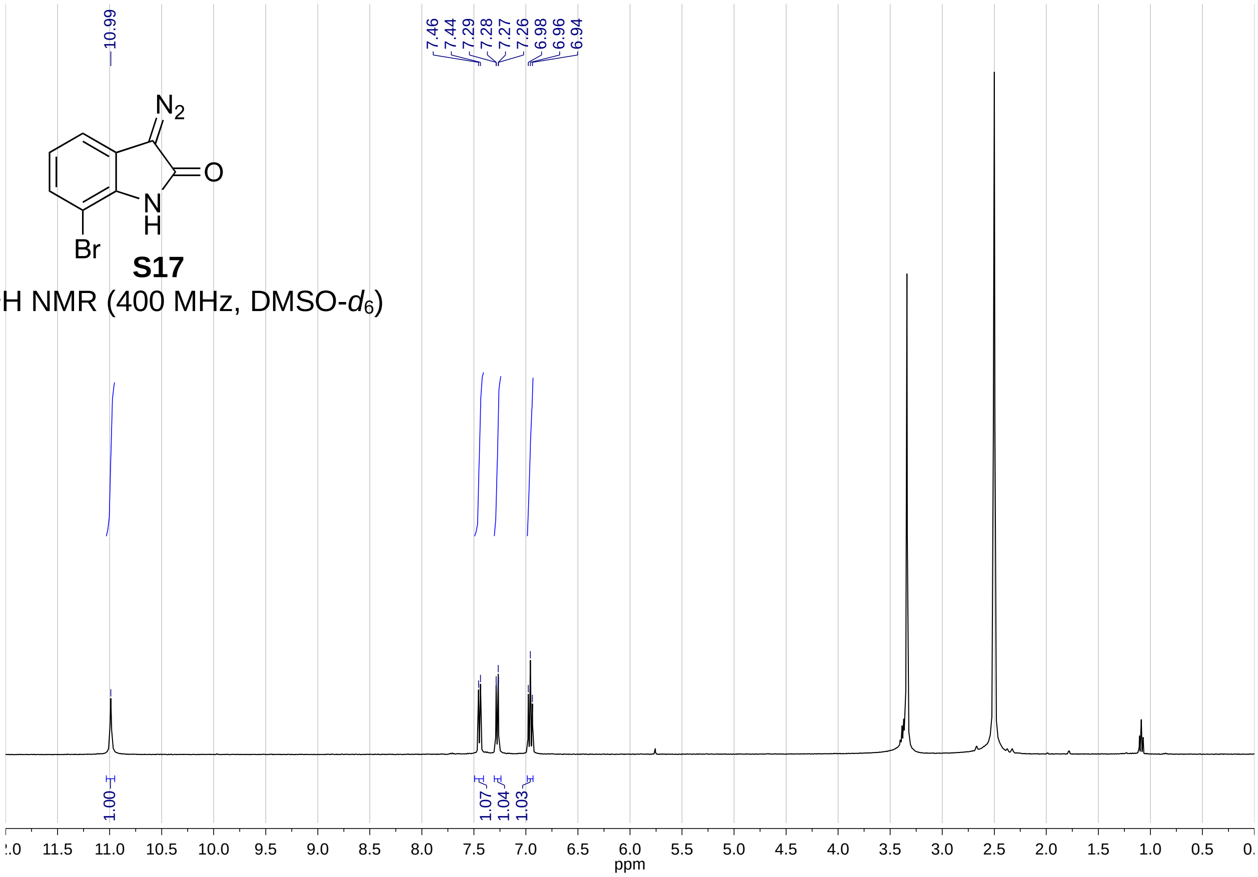

S17

^{13}C NMR (101 MHz, DMSO-d_6)

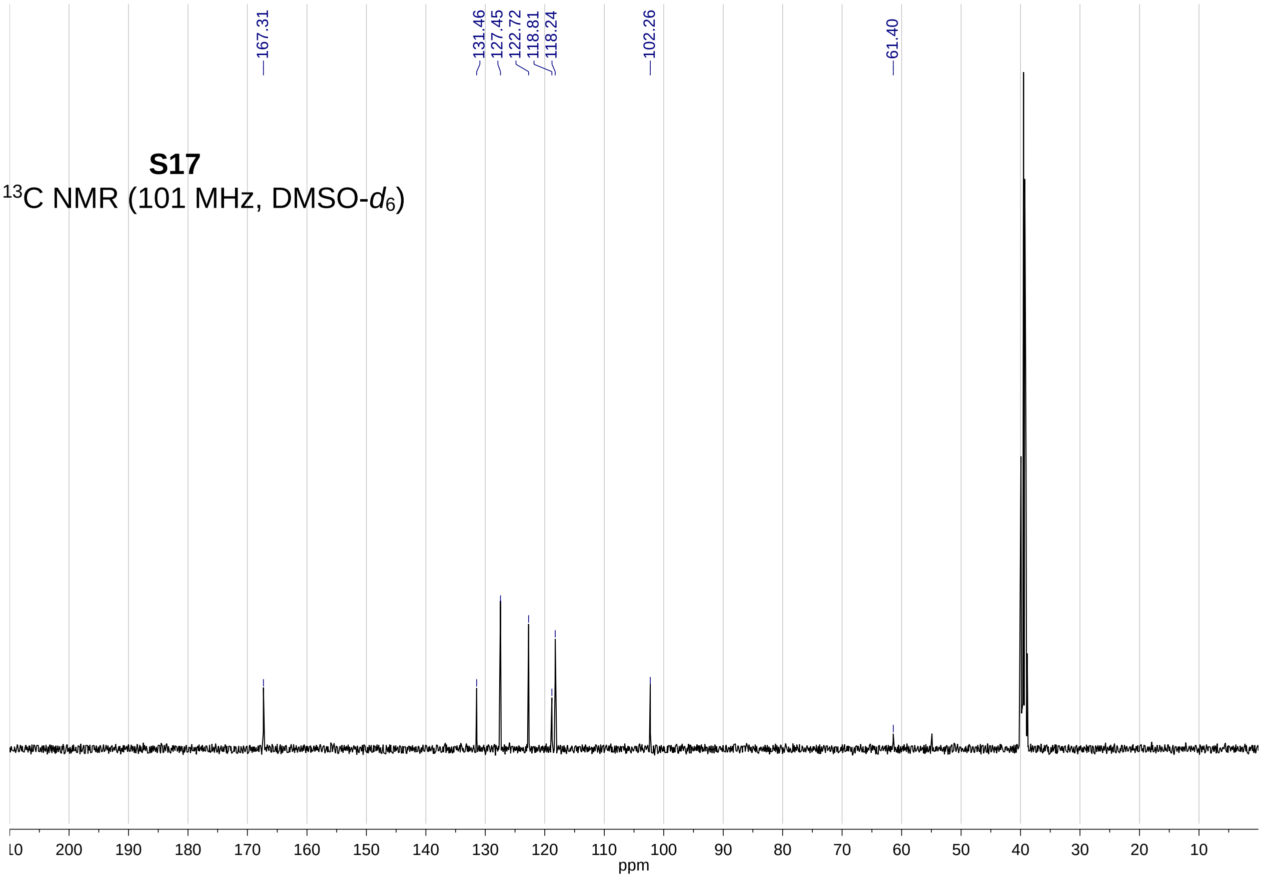

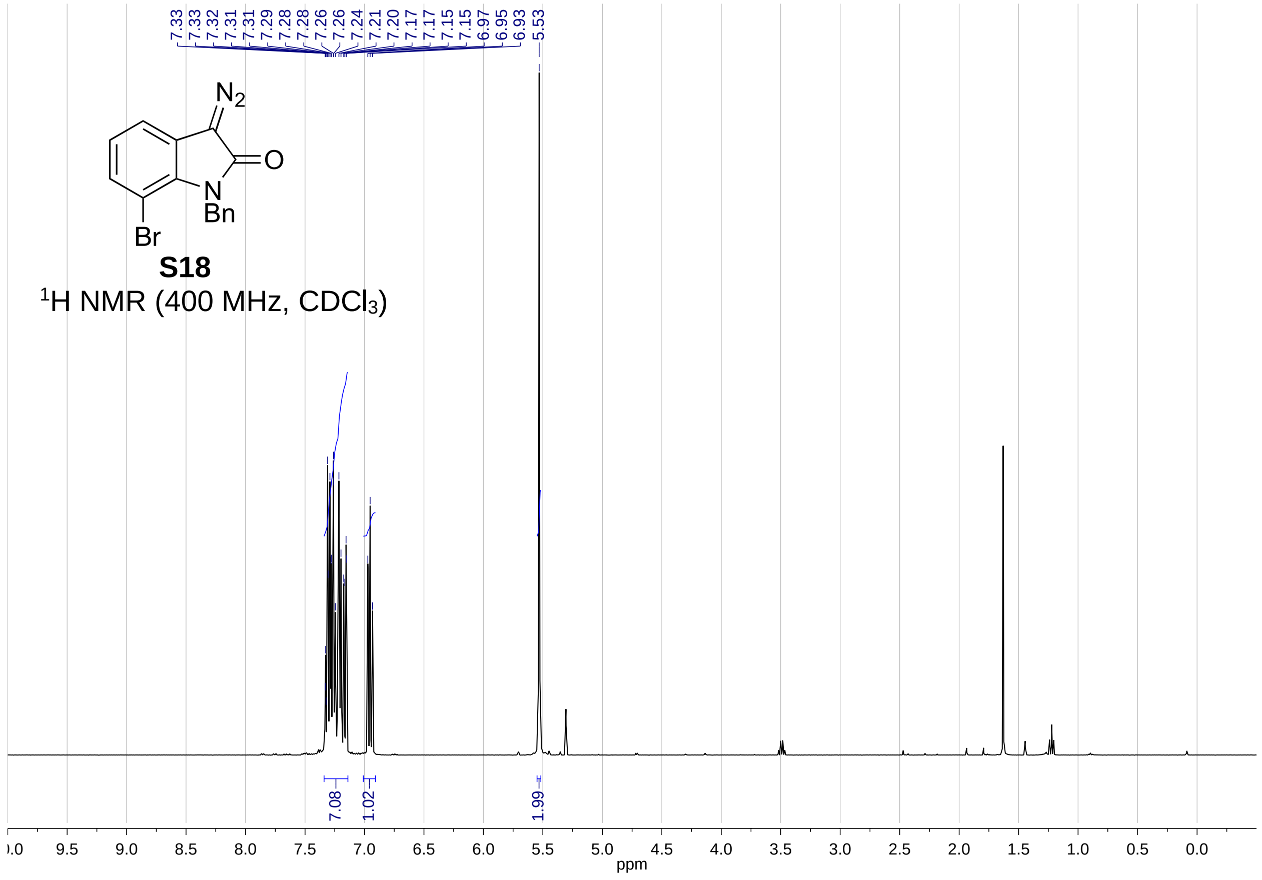

S18
^{1}H NMR (400 MHz, $CDCl_3$)

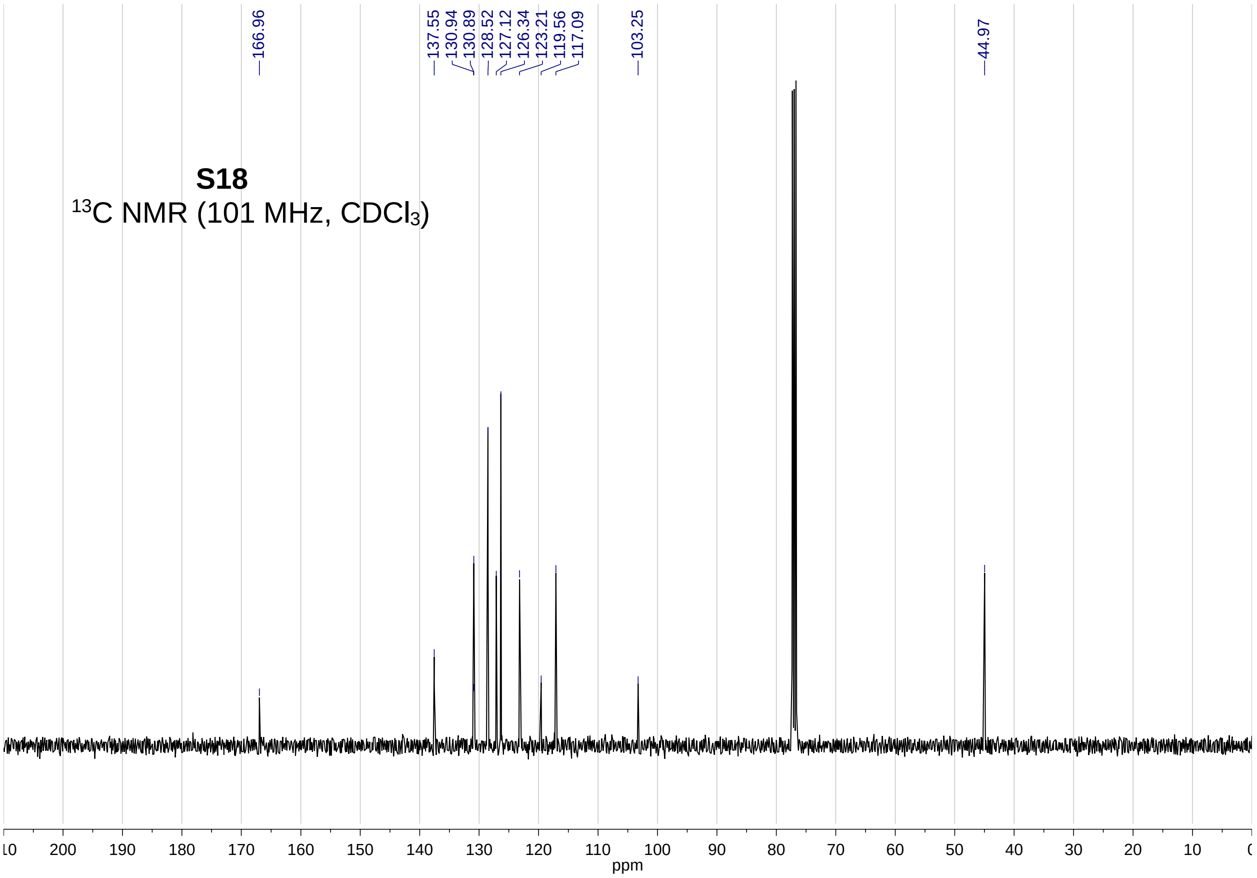

S18
^{13}C NMR (101 MHz, $CDCl_3$)

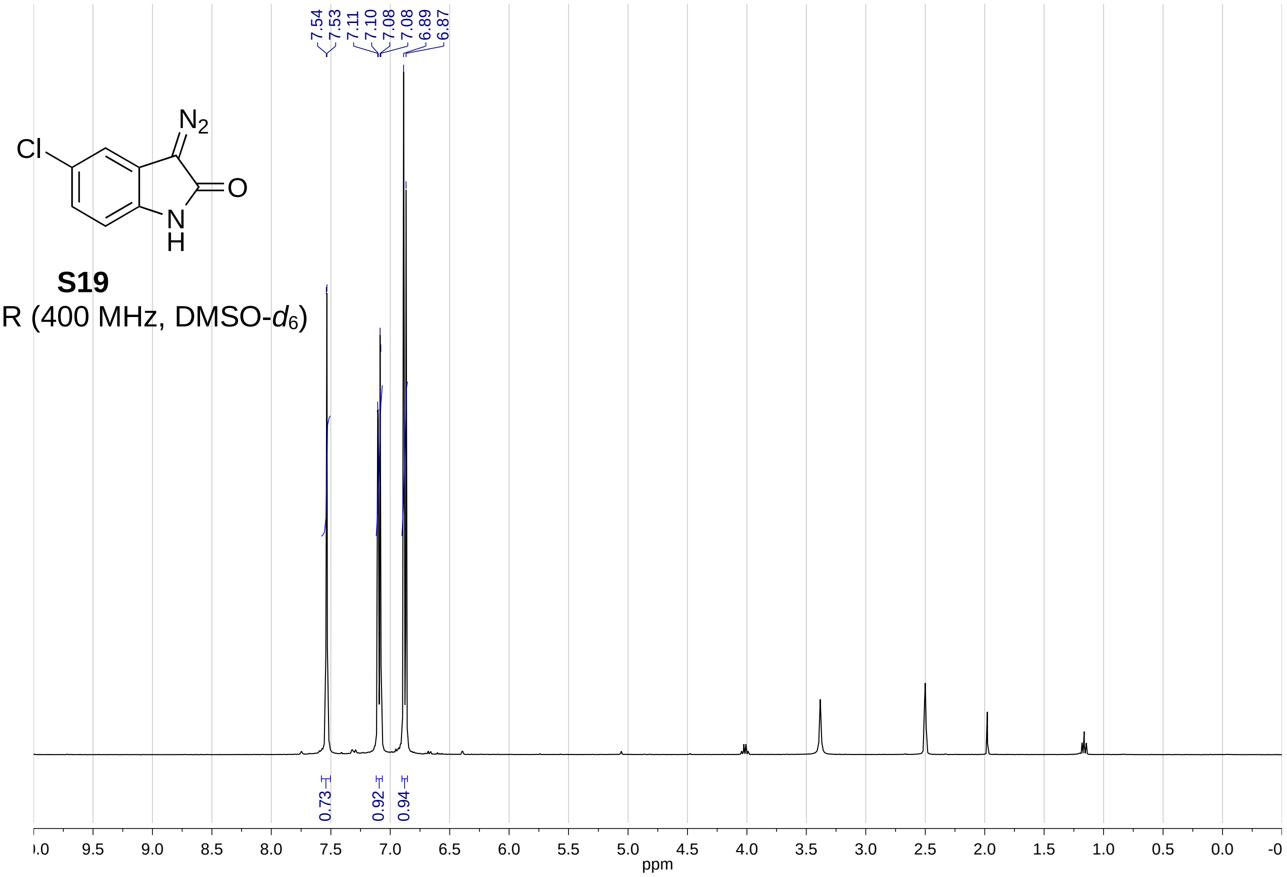

S19

^{1}H NMR (400 MHz, DMSO-d_6)

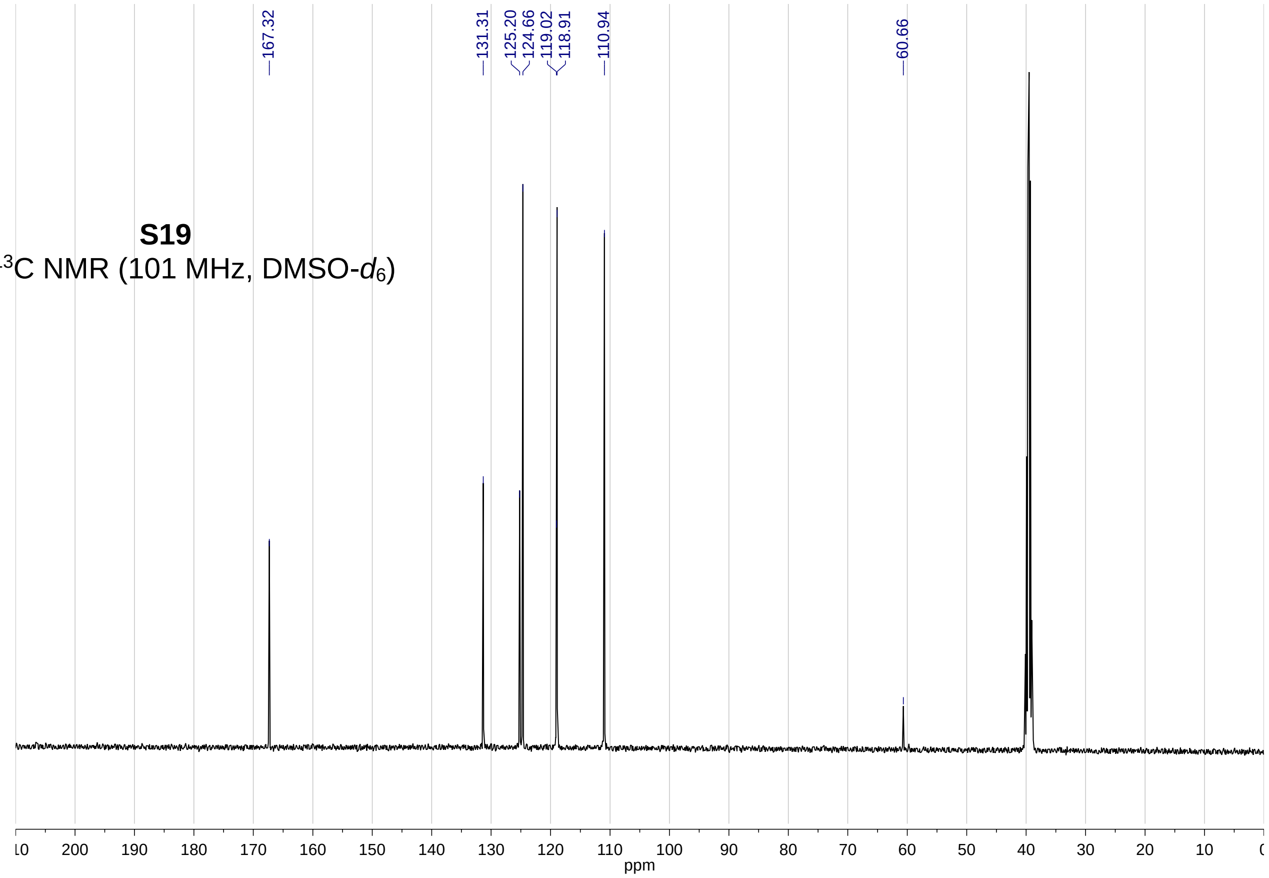

S19

^{13}C NMR (101 MHz, DMSO-d_6)

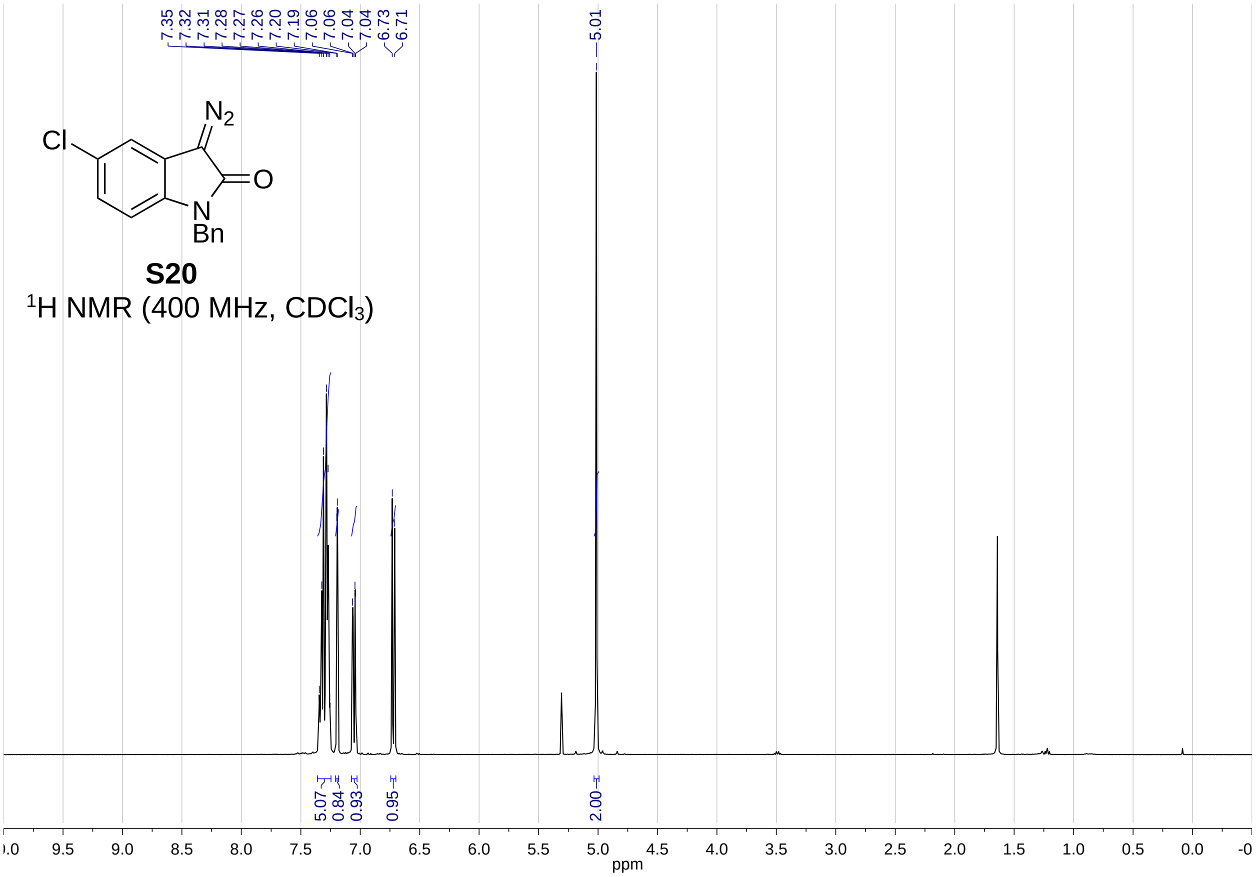

S20
^{1}H NMR (400 MHz, $CDCl_3$)

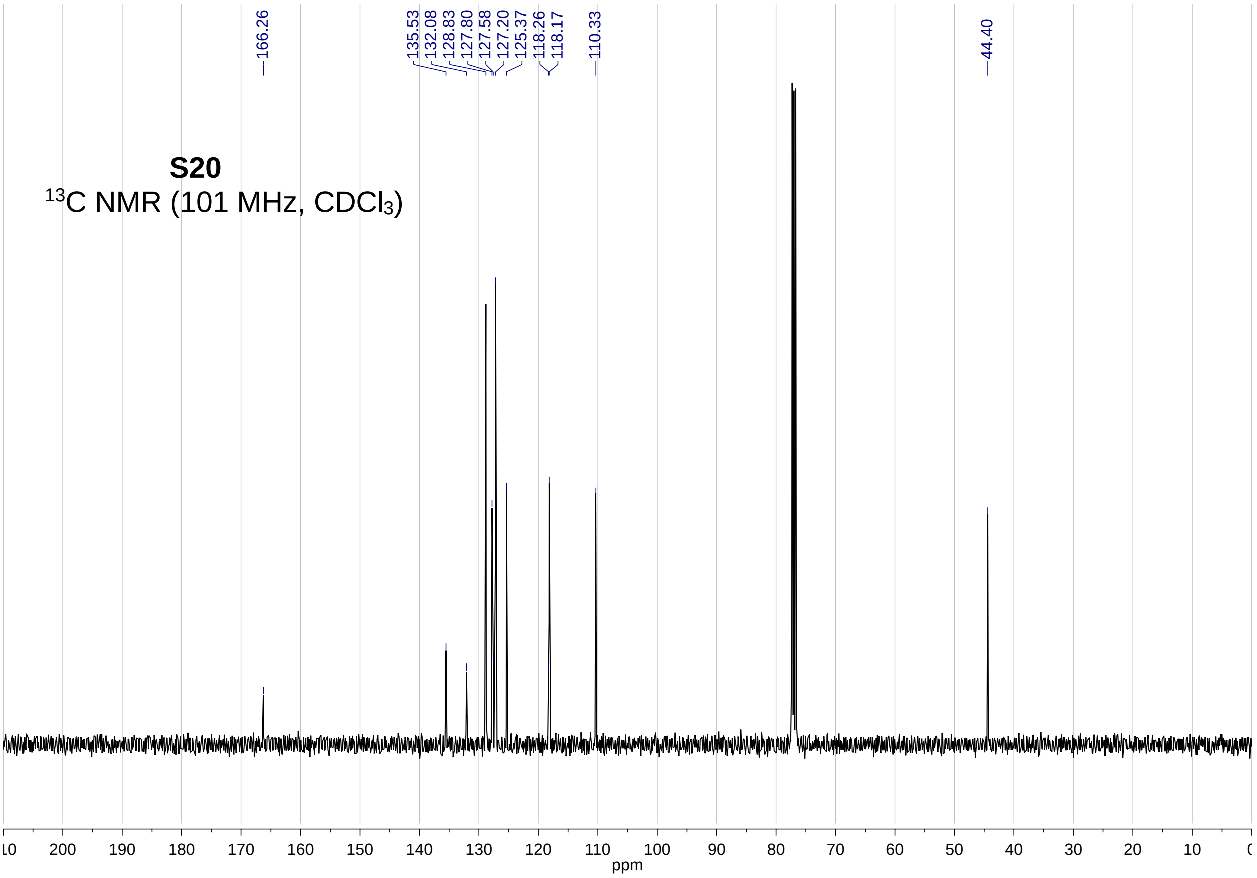

S20
^{13}C NMR (101 MHz, $CDCl_3$)

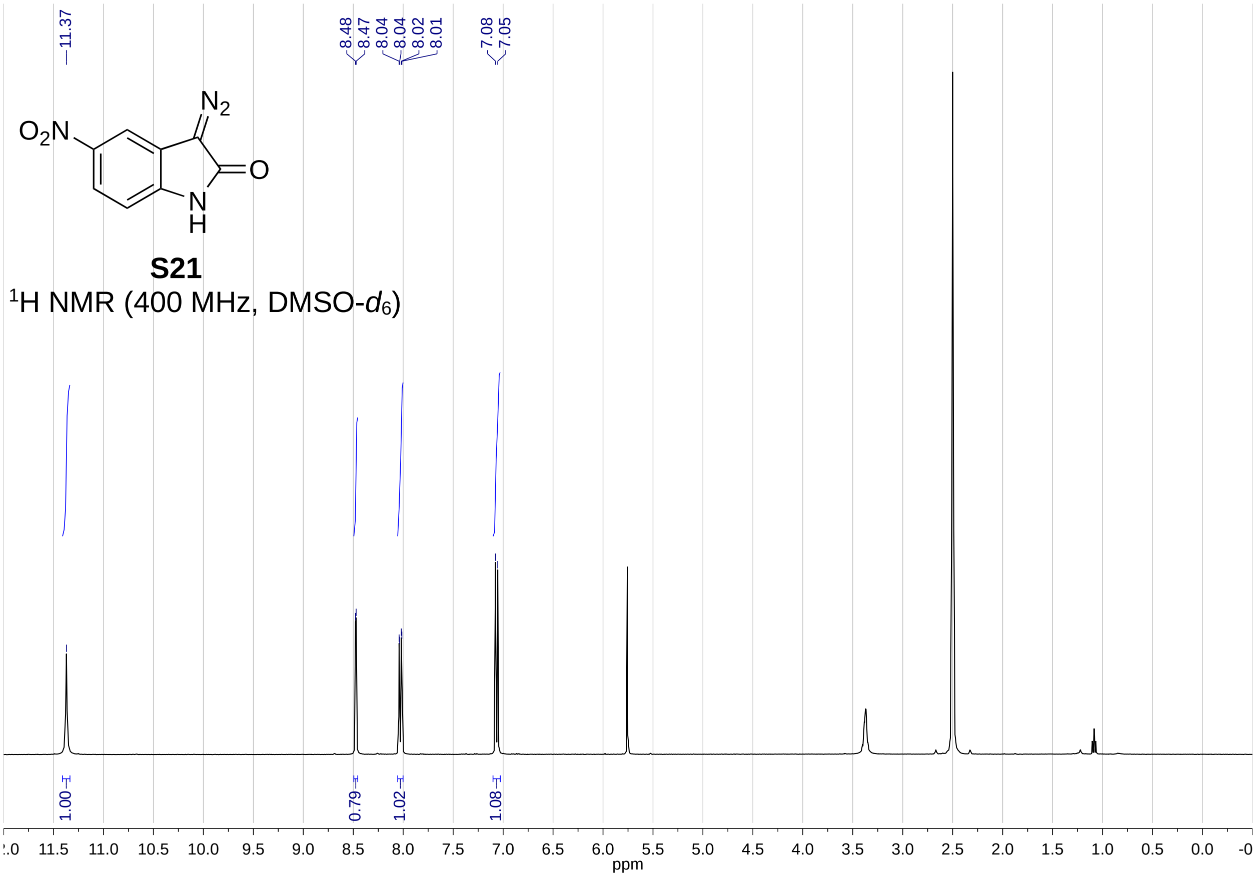

S21
^{1}H NMR (400 MHz, DMSO-d_6)

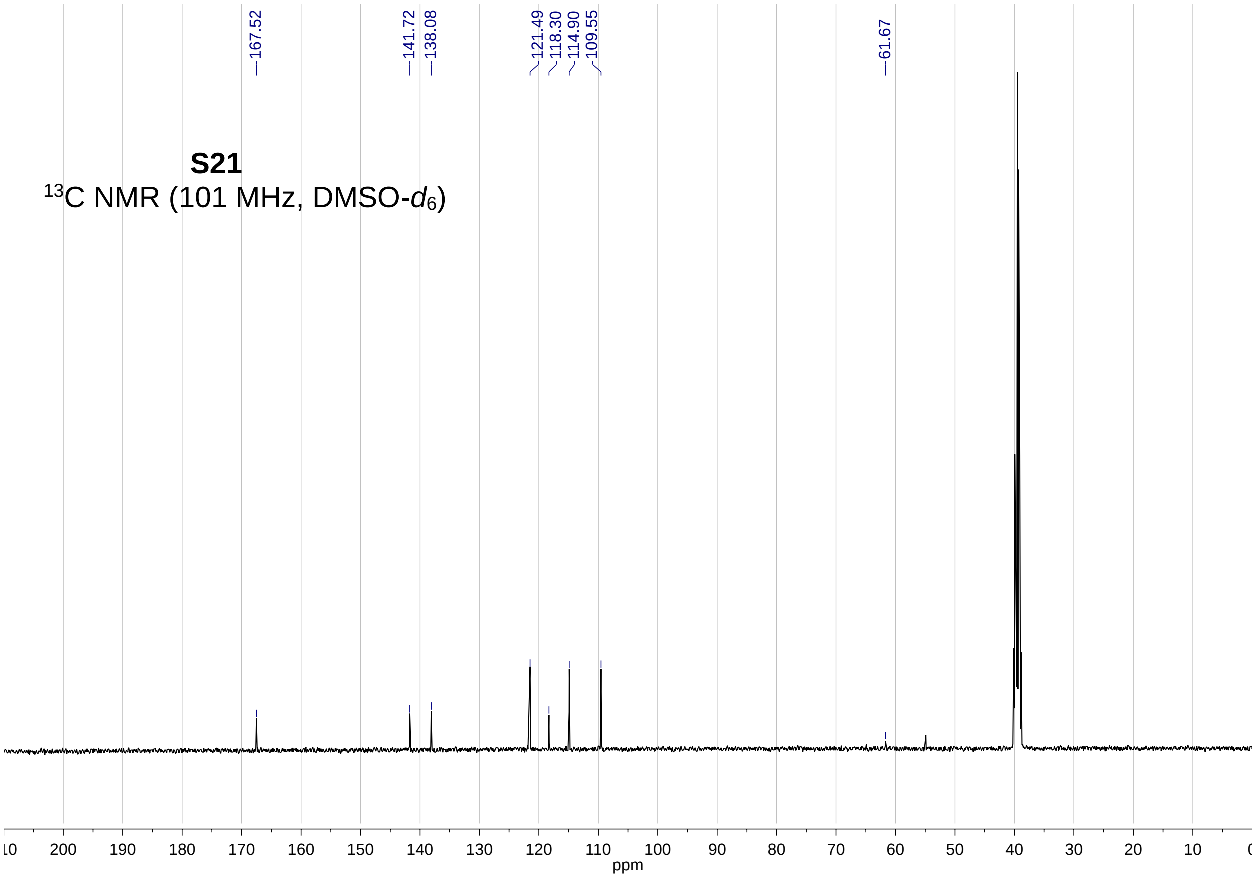

S21
^{13}C NMR (101 MHz, DMSO-d_6)

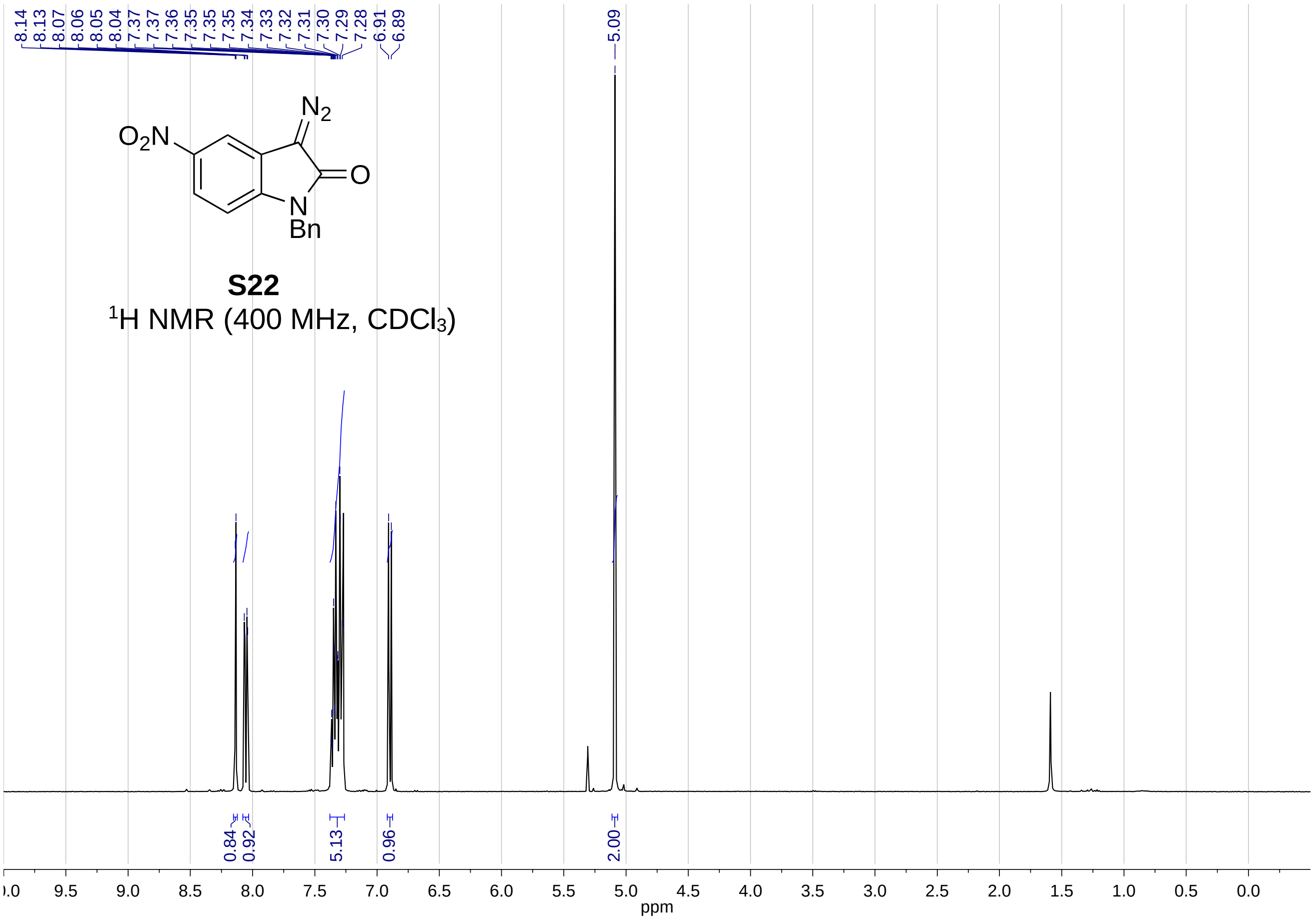
8.14
8.13
8.07
8.06
8.05
8.04
7.37
7.37
7.36
7.35
7.35
7.35
7.34
7.33
7.32
7.31
7.30
7.29
7.28
6.91
6.89
5.09
S22
¹H NMR (400 MHz, CDCl₃)
0.84
0.92
5.13
0.96
2.00
ppm

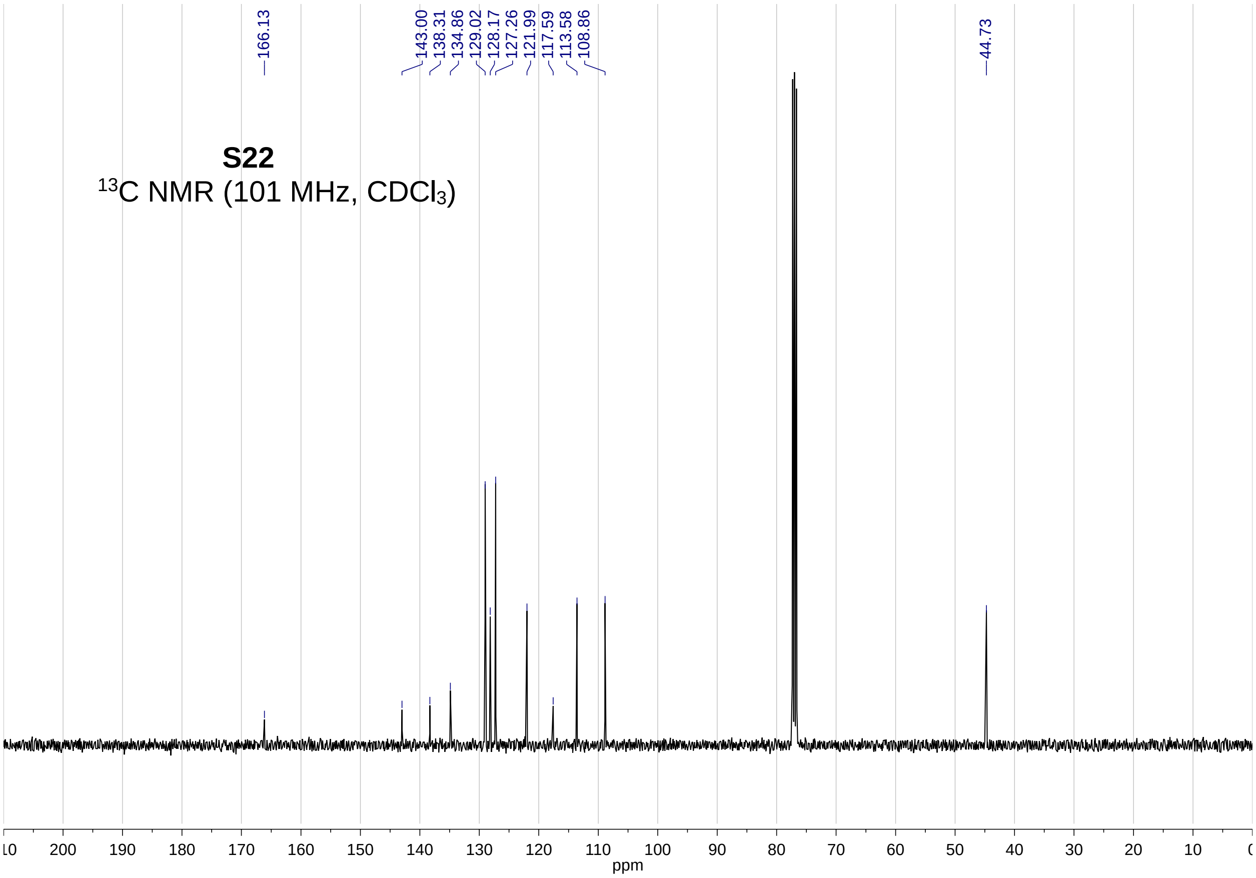
166.13
143.00
138.31
134.86
129.02
128.17
127.26
121.99
117.59
113.58
108.86
44.73
S22
¹³C NMR (101 MHz, CDCl₃)
ppm

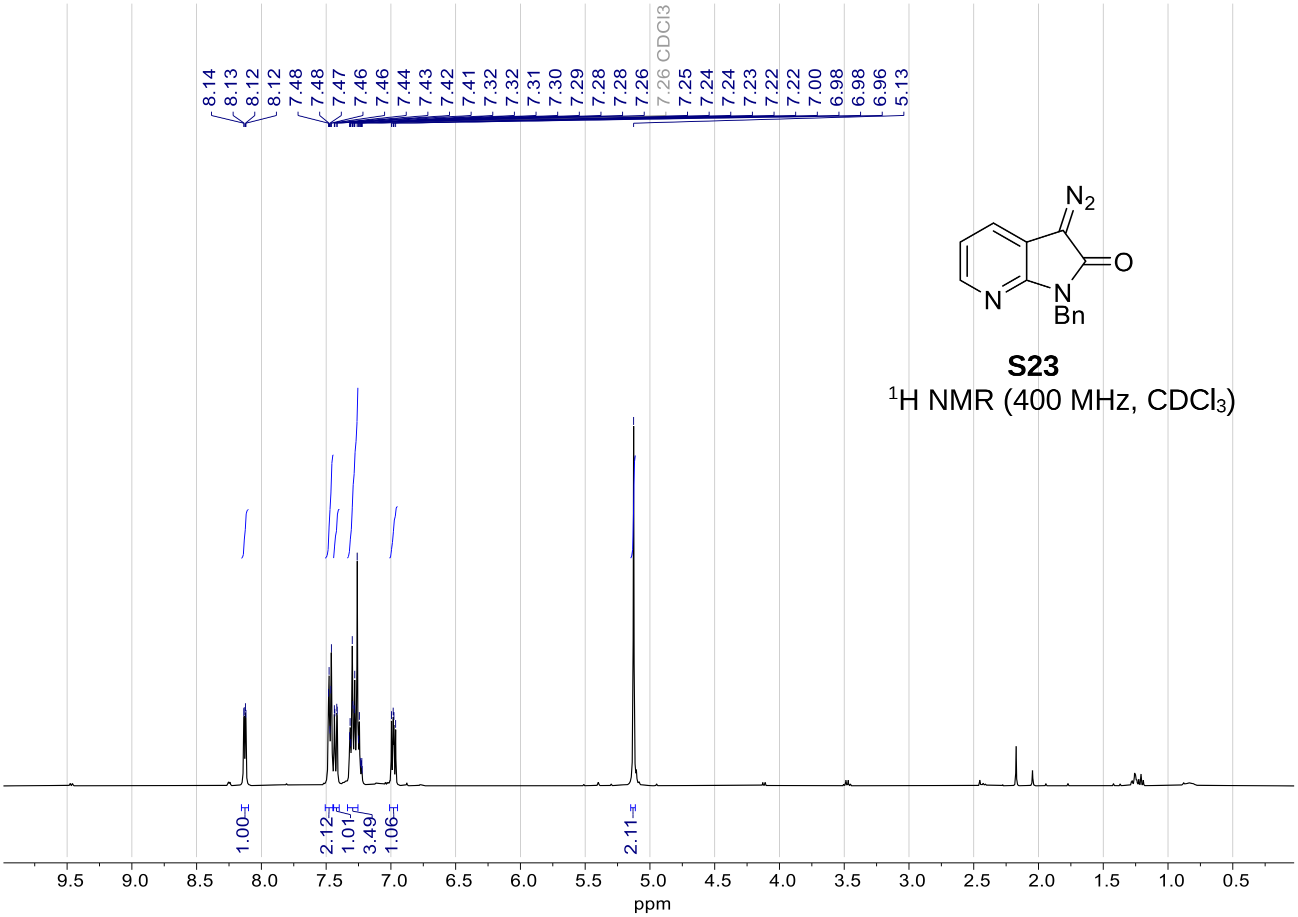

S23
^{1}H NMR (400 MHz, $CDCl_3$)

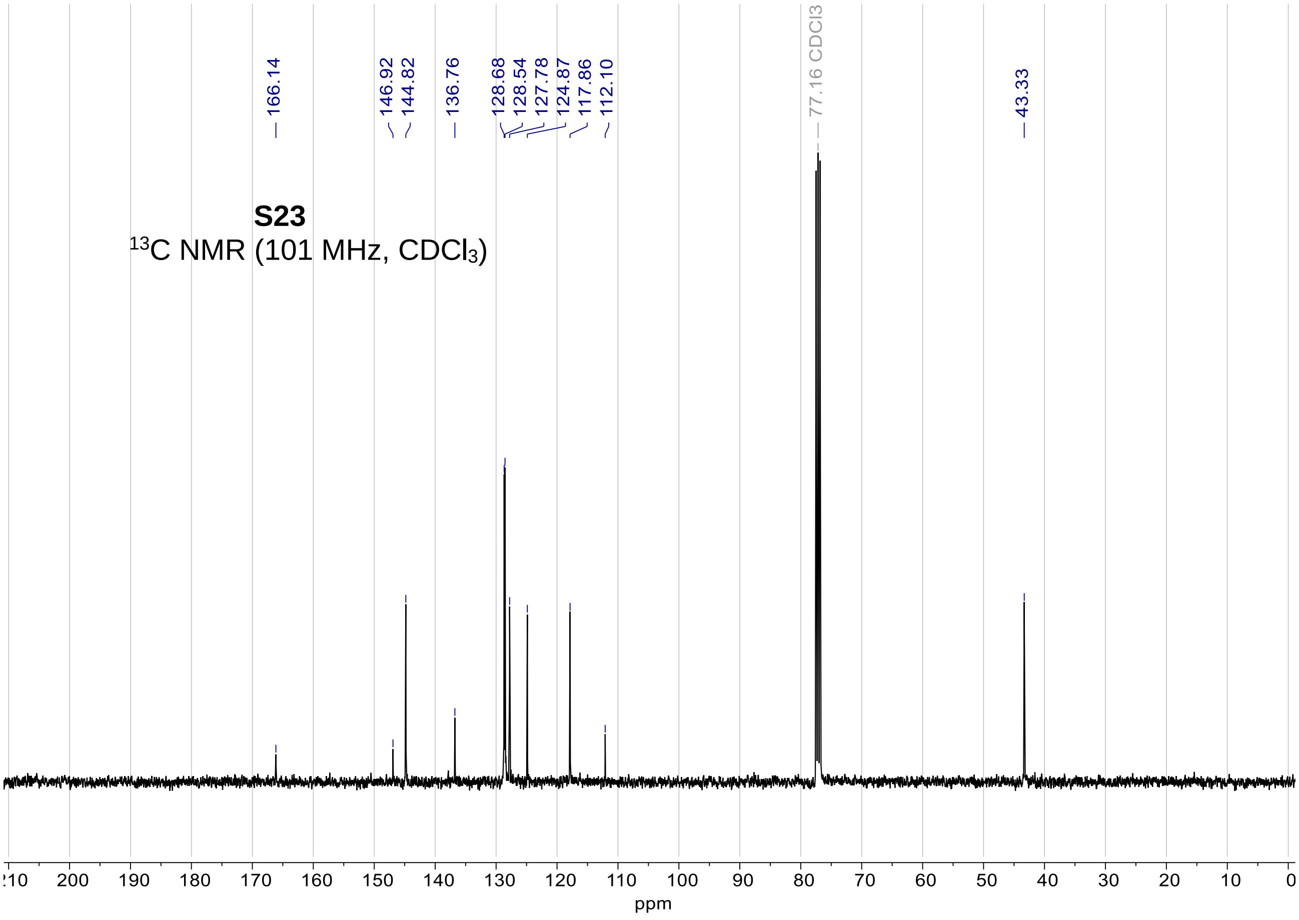

S23
^{13}C NMR (101 MHz, $CDCl_3$)

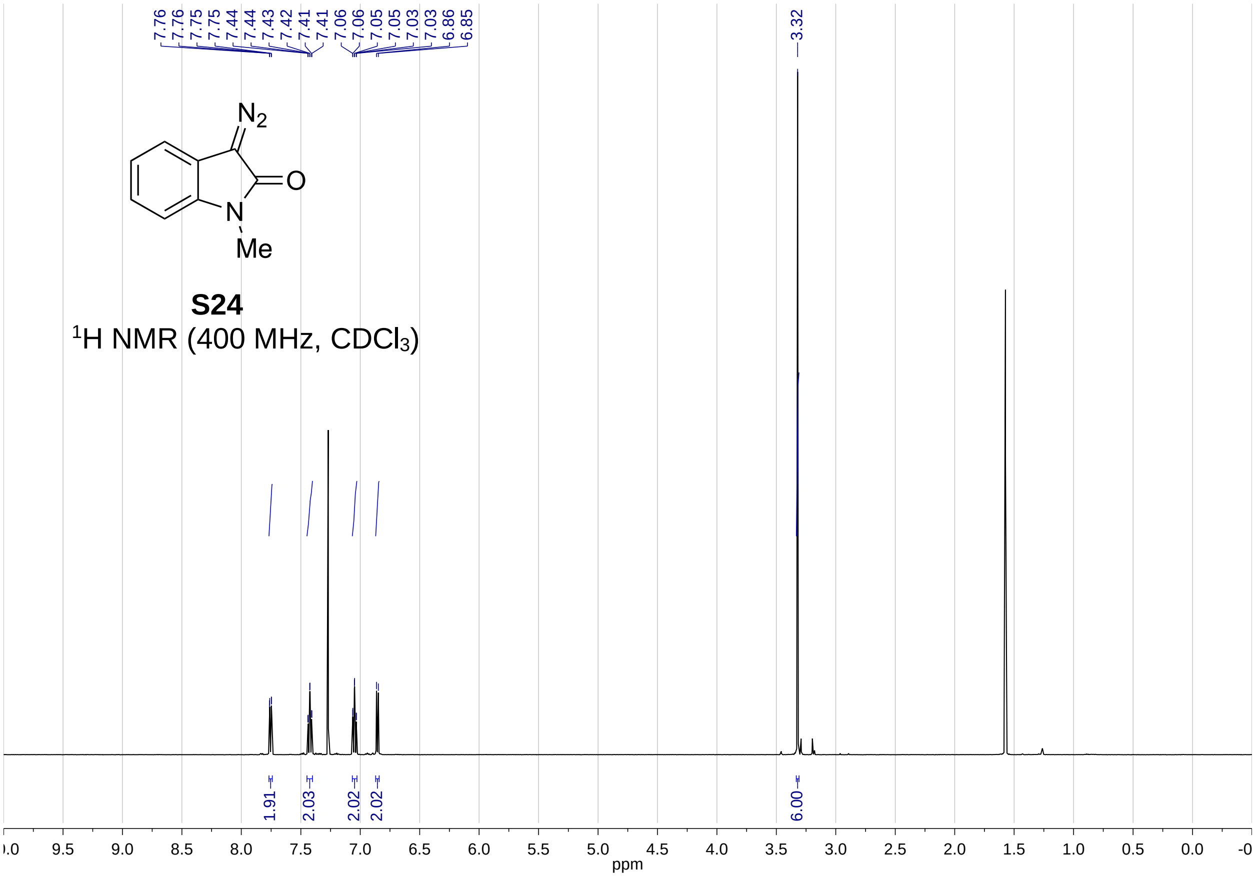
7.76
7.76
7.75
7.75
7.44
7.44
7.43
7.42
7.41
7.41
7.06
7.06
7.05
7.05
7.03
7.03
6.86
6.85
3.32
N2
O
N
Me
S24
1H NMR (400 MHz, CDCl3)
1.91
2.03
2.02
2.02
6.00
ppm

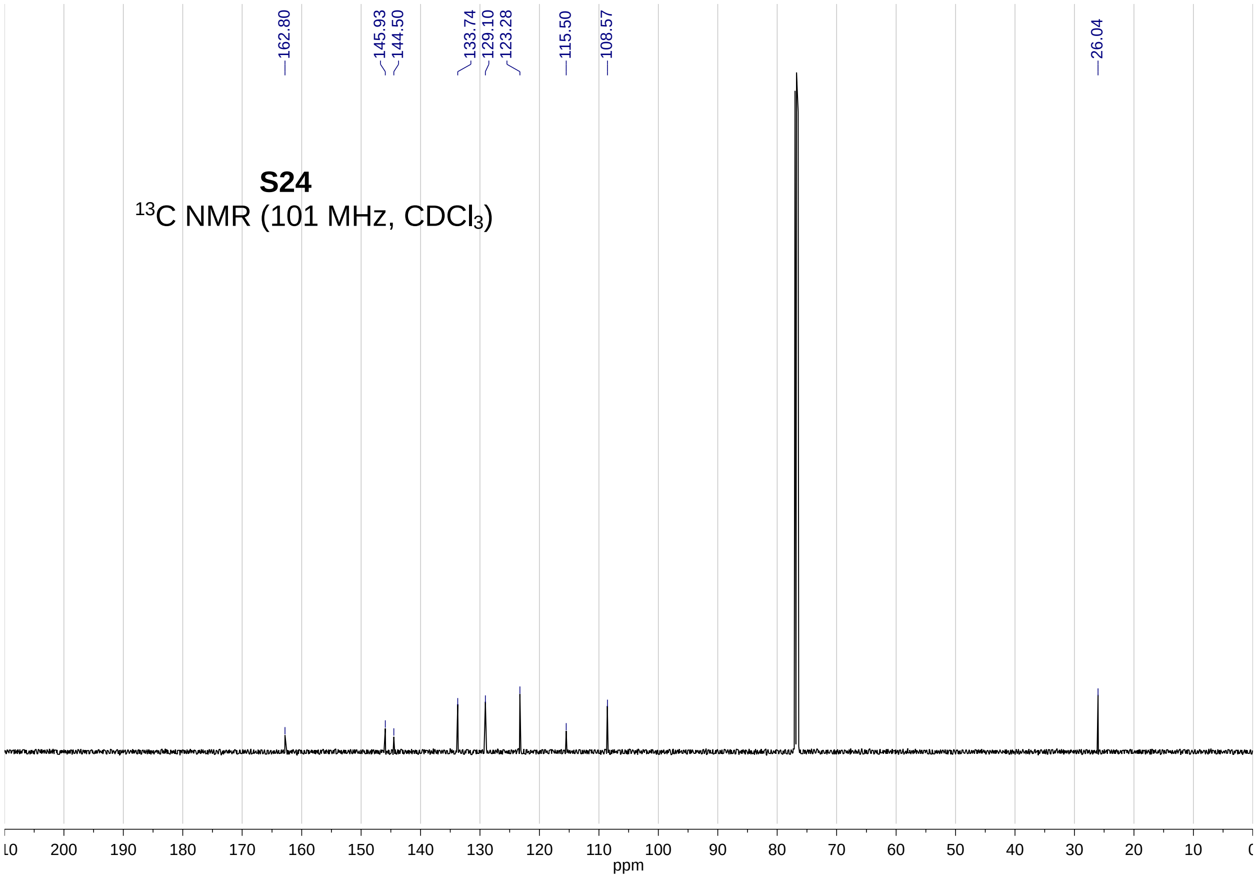
162.80
145.93
144.50
133.74
129.10
123.28
115.50
108.57
26.04
S24
13C NMR (101 MHz, CDCl3)
ppm

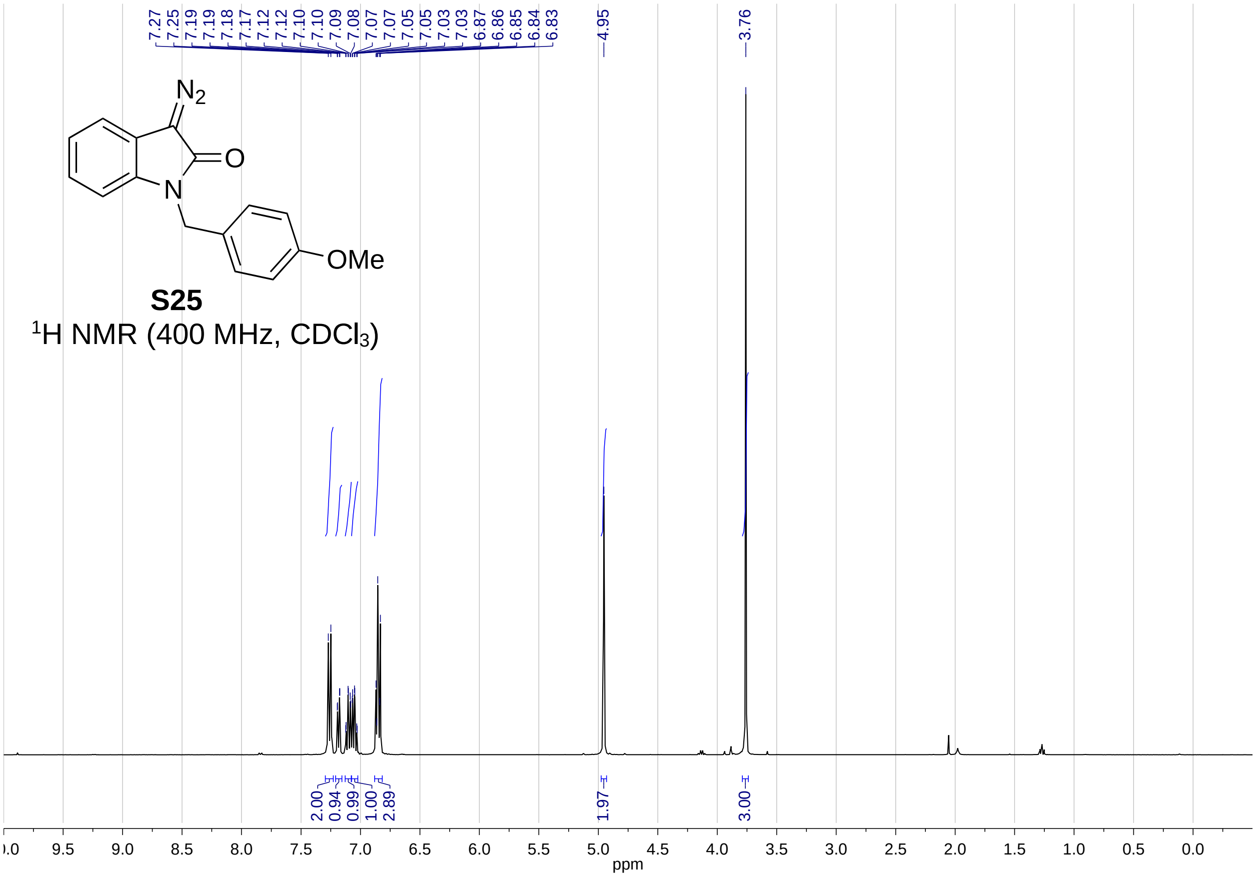

S25

^{1}H NMR (400 MHz, $CDCl_3$)

S25

^{13}C NMR (101 MHz, $CDCl_3$)

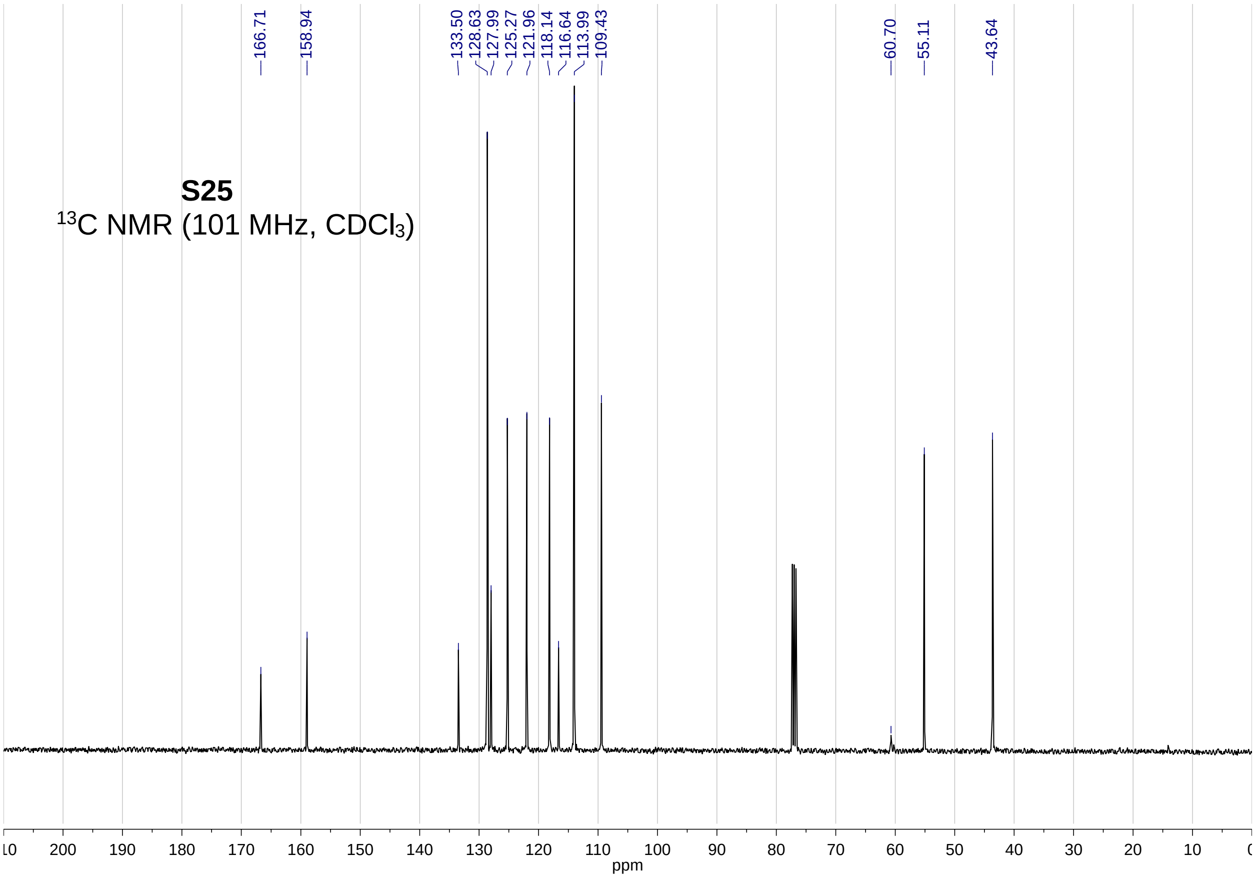

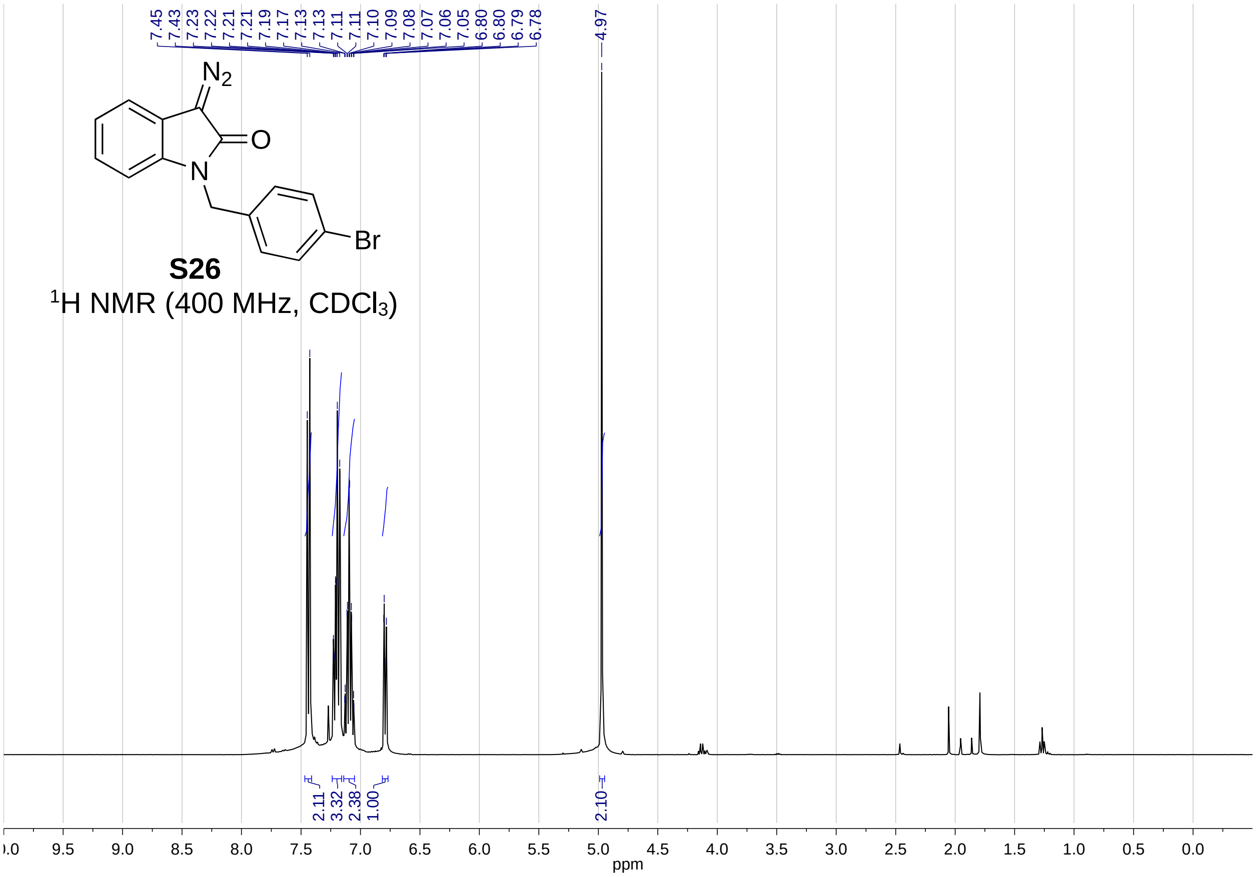
7.45
7.43
7.23
7.22
7.21
7.21
7.19
7.17
7.13
7.13
7.11
7.11
7.10
7.09
7.08
7.07
7.06
7.05
6.80
6.80
6.79
6.78
4.97
N2
O
N
Br
S26
1H NMR (400 MHz, CDCl3)
2.11
3.32
2.38
1.00
2.10
ppm

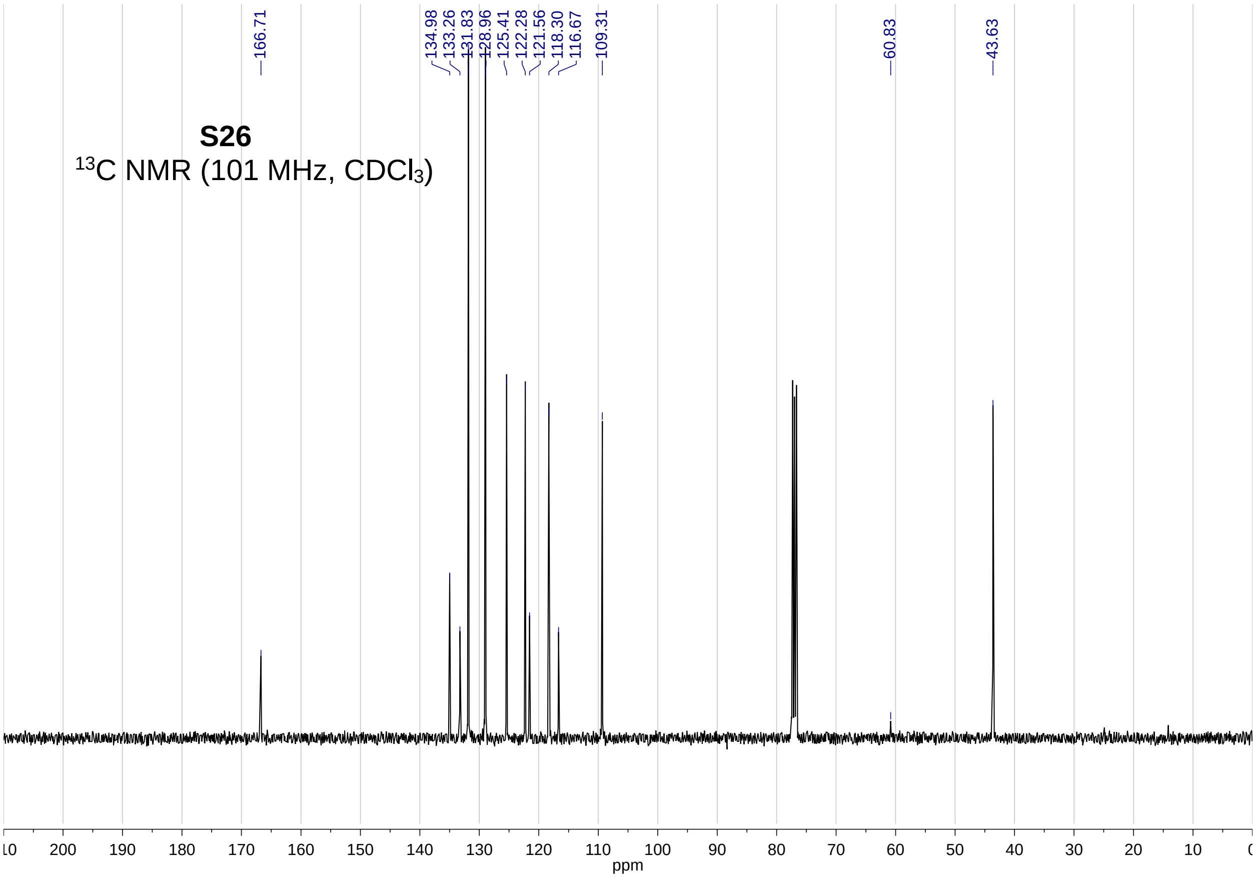
166.71
134.98
133.26
131.83
128.96
125.41
122.28
121.56
118.30
116.67
109.31
60.83
43.63
S26
13C NMR (101 MHz, CDCl3)
ppm

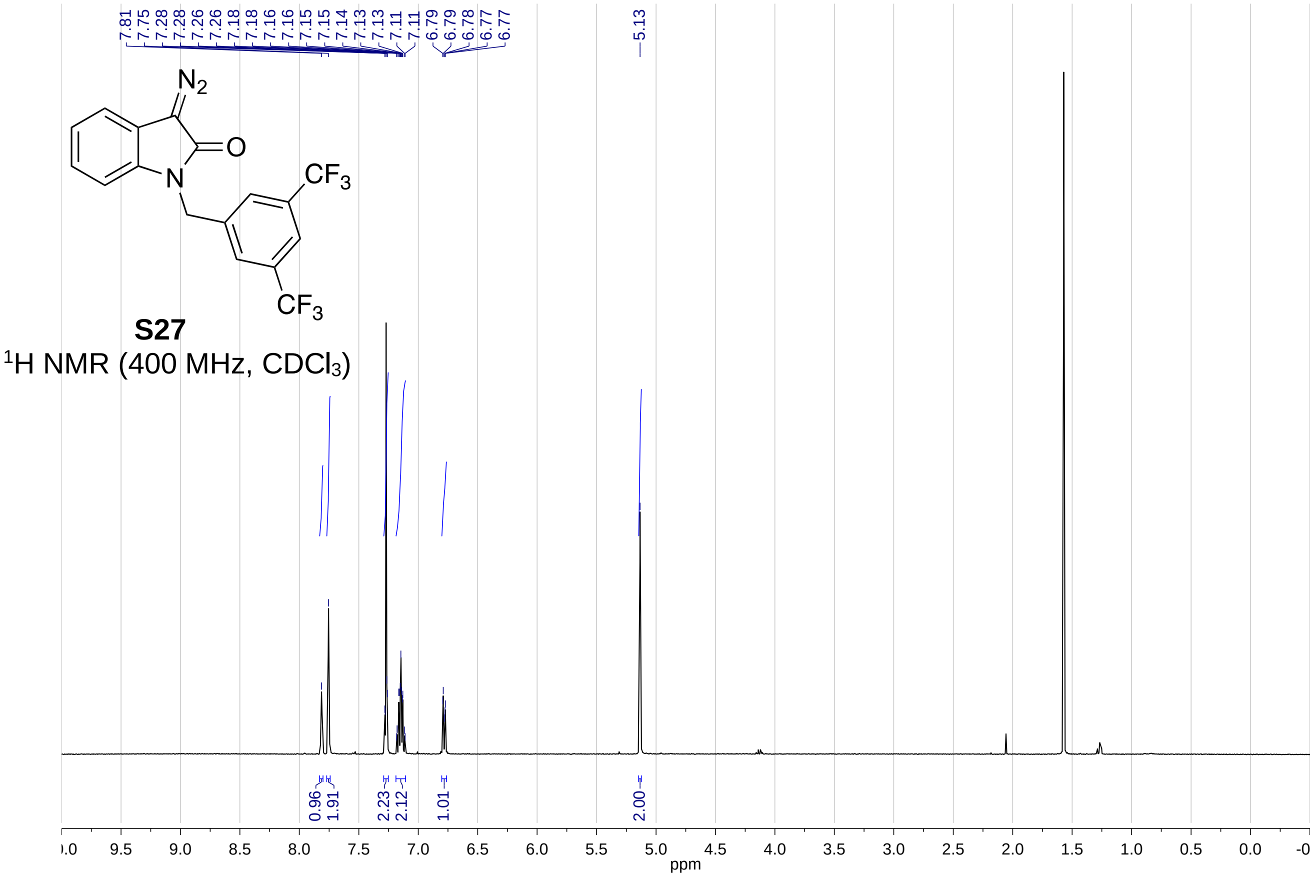
N2
O
N
CF3
CF3
S27
1H NMR (400 MHz, CDCl3)
7.81
7.75
7.28
7.28
7.26
7.26
7.18
7.18
7.16
7.16
7.15
7.15
7.14
7.13
7.13
7.11
7.11
6.79
6.79
6.78
6.77
6.77
5.13
0.96
1.91
2.23
2.12
1.01
2.00
ppm

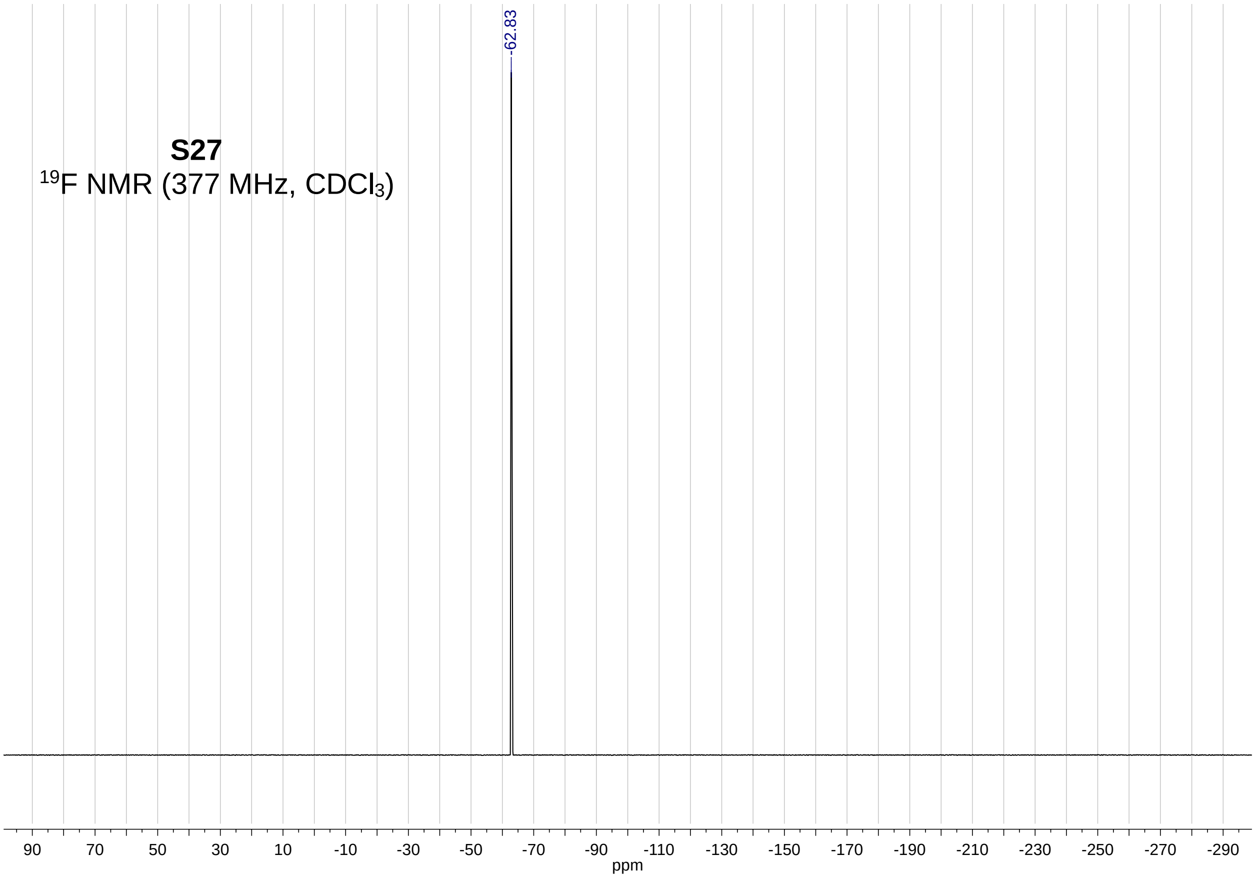
-62.83
S27
19F NMR (377 MHz, CDCl3)
ppm

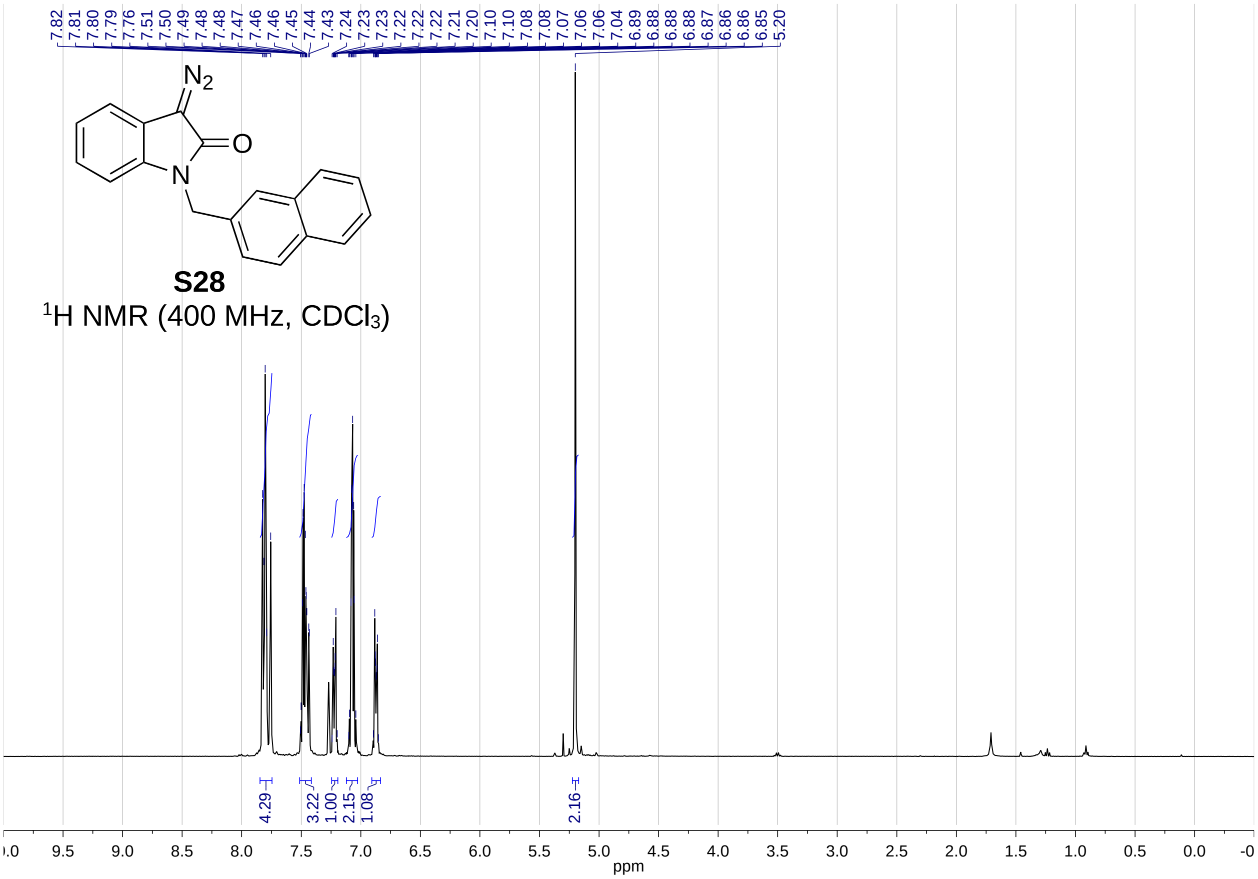

S28

^{1}H NMR (400 MHz, $CDCl_3$)

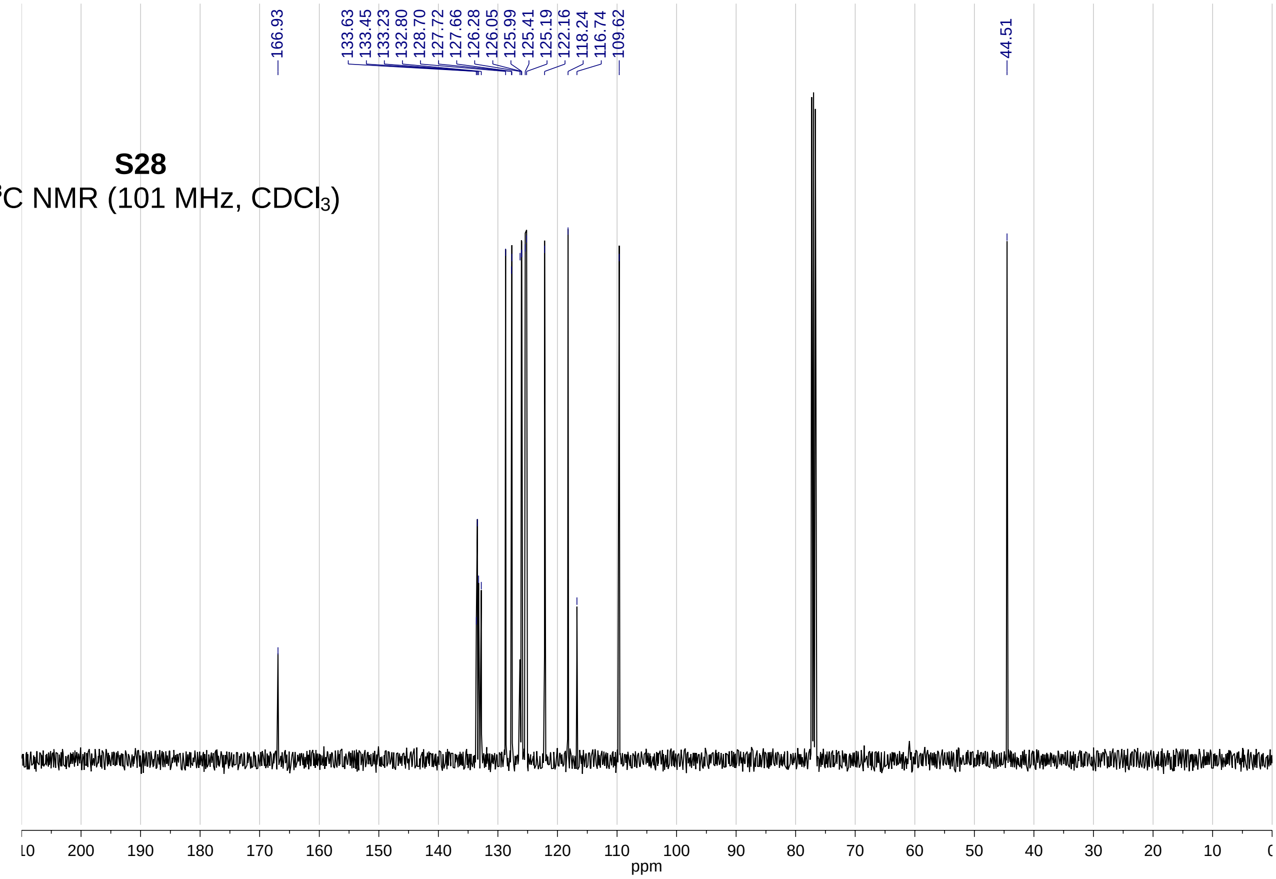

S28

^{13}C NMR (101 MHz, $CDCl_3$)

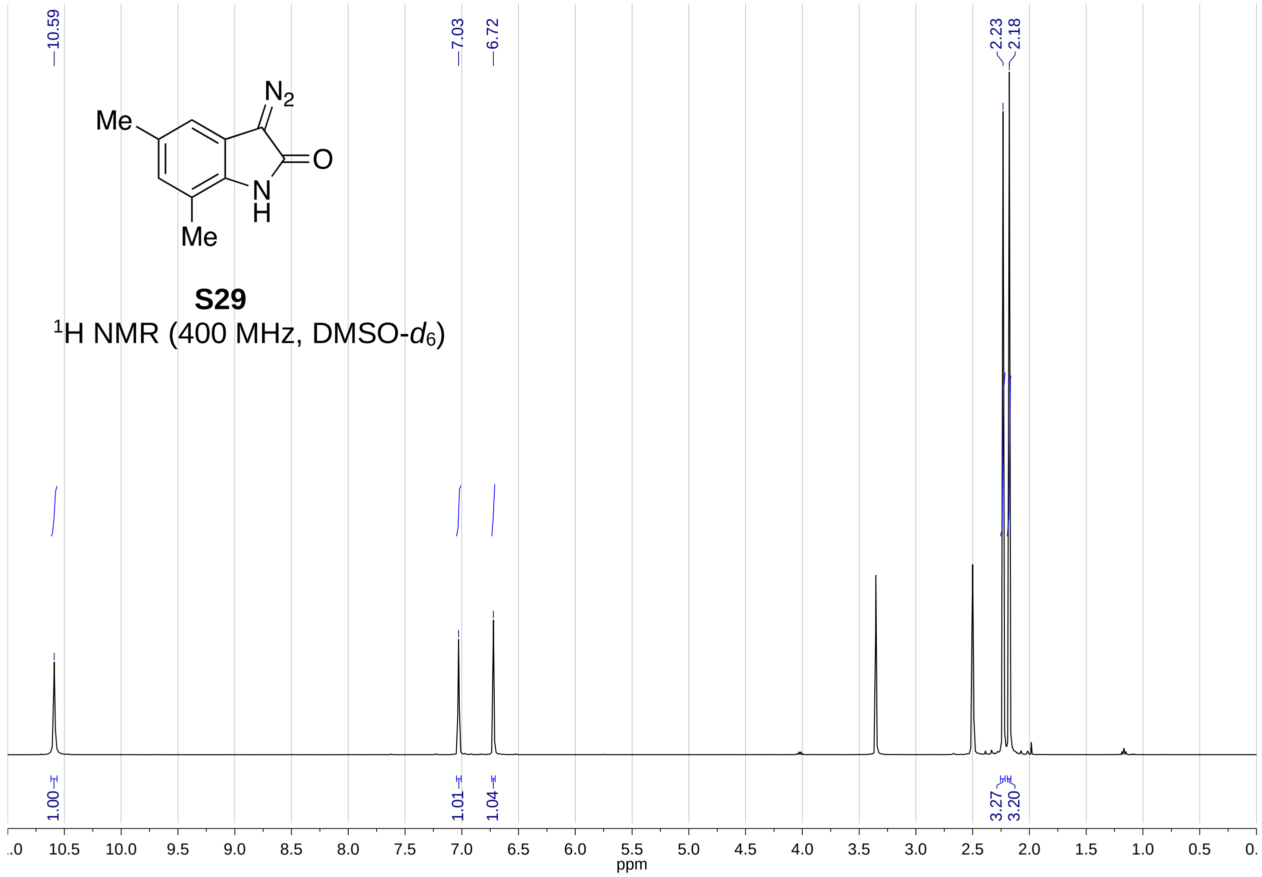
S29
^{1}H NMR (400 MHz, DMSO-d_6)
10.59
7.03
6.72
2.23
2.18
1.00
1.01
1.04
3.27
3.20
ppm

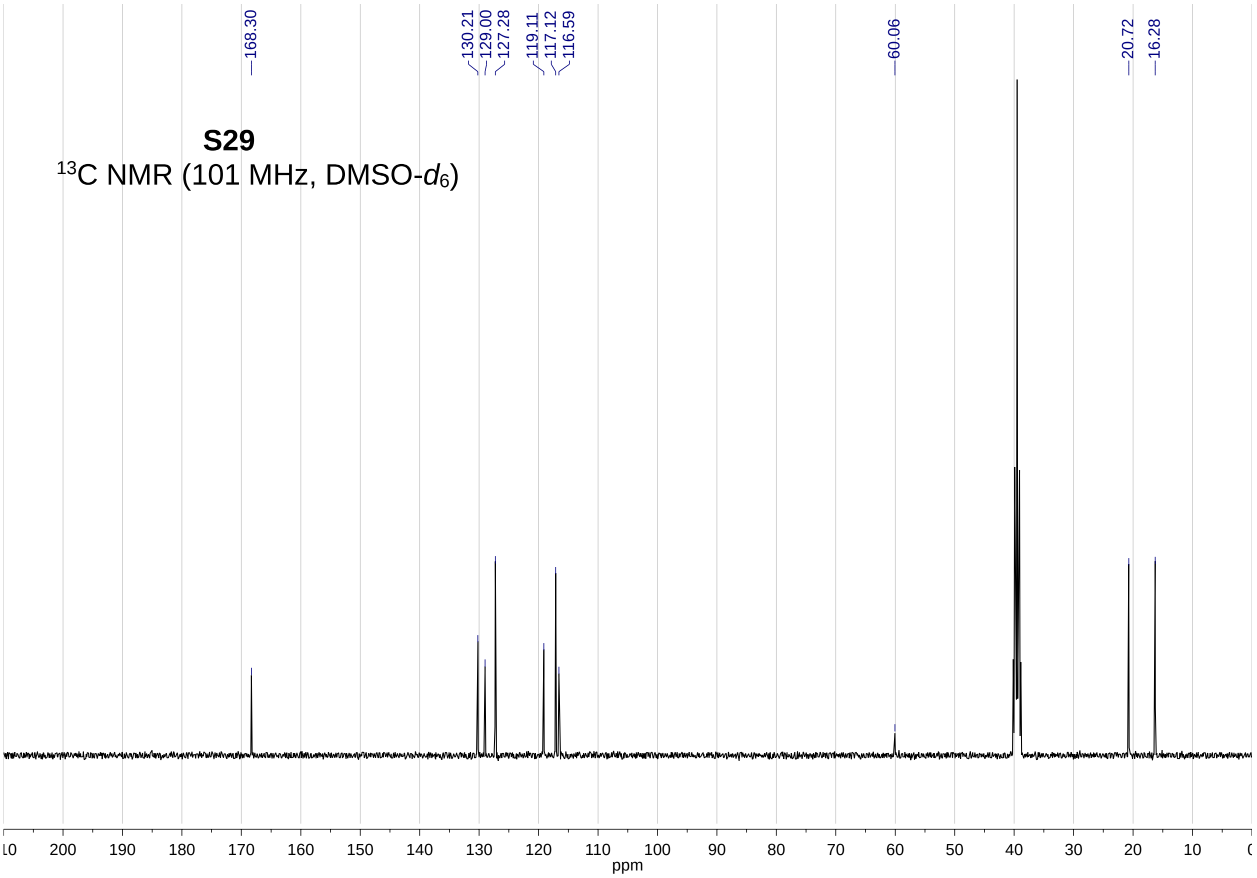
S29
^{13}C NMR (101 MHz, DMSO-d_6)
168.30
130.21
129.00
127.28
119.11
117.12
116.59
60.06
20.72
16.28
ppm

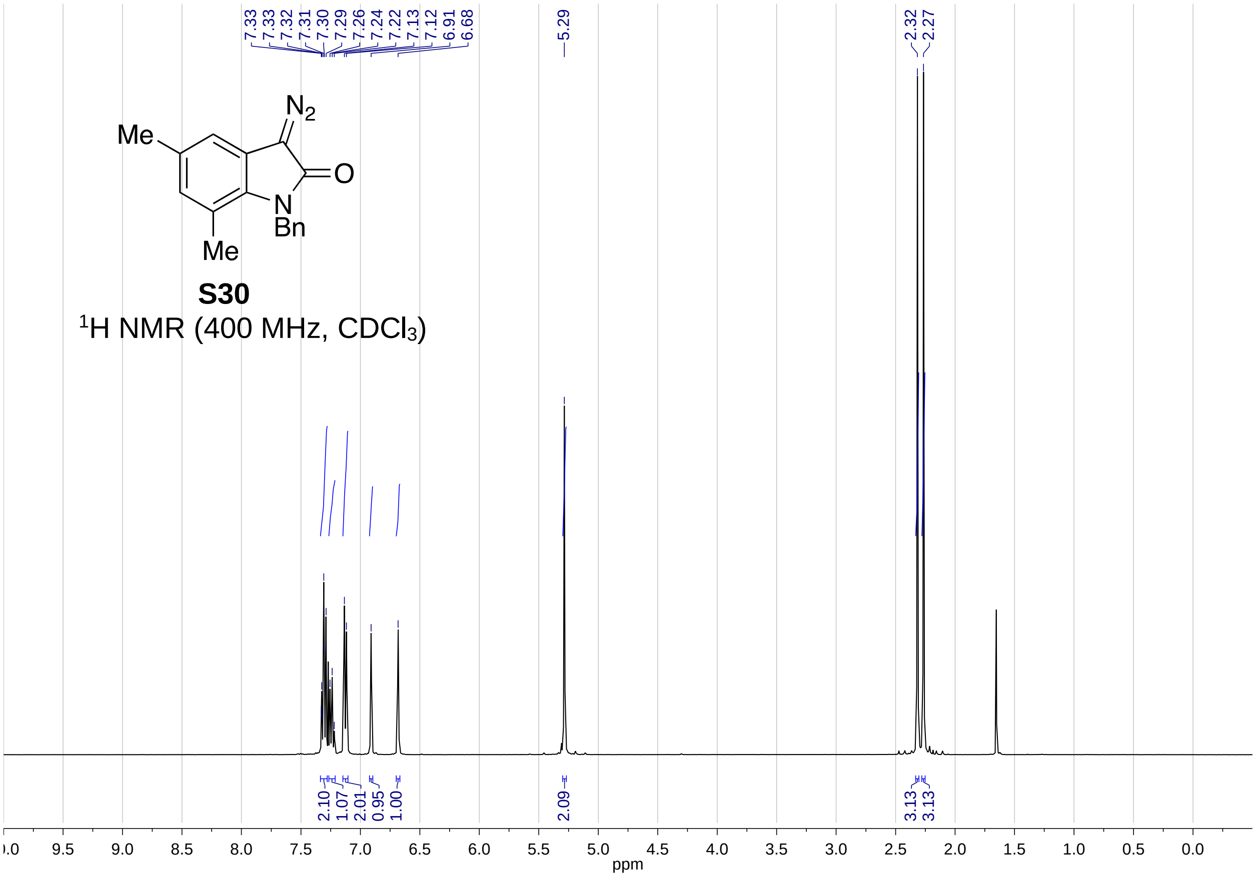

S30

^{1}H NMR (400 MHz, $CDCl_3$)

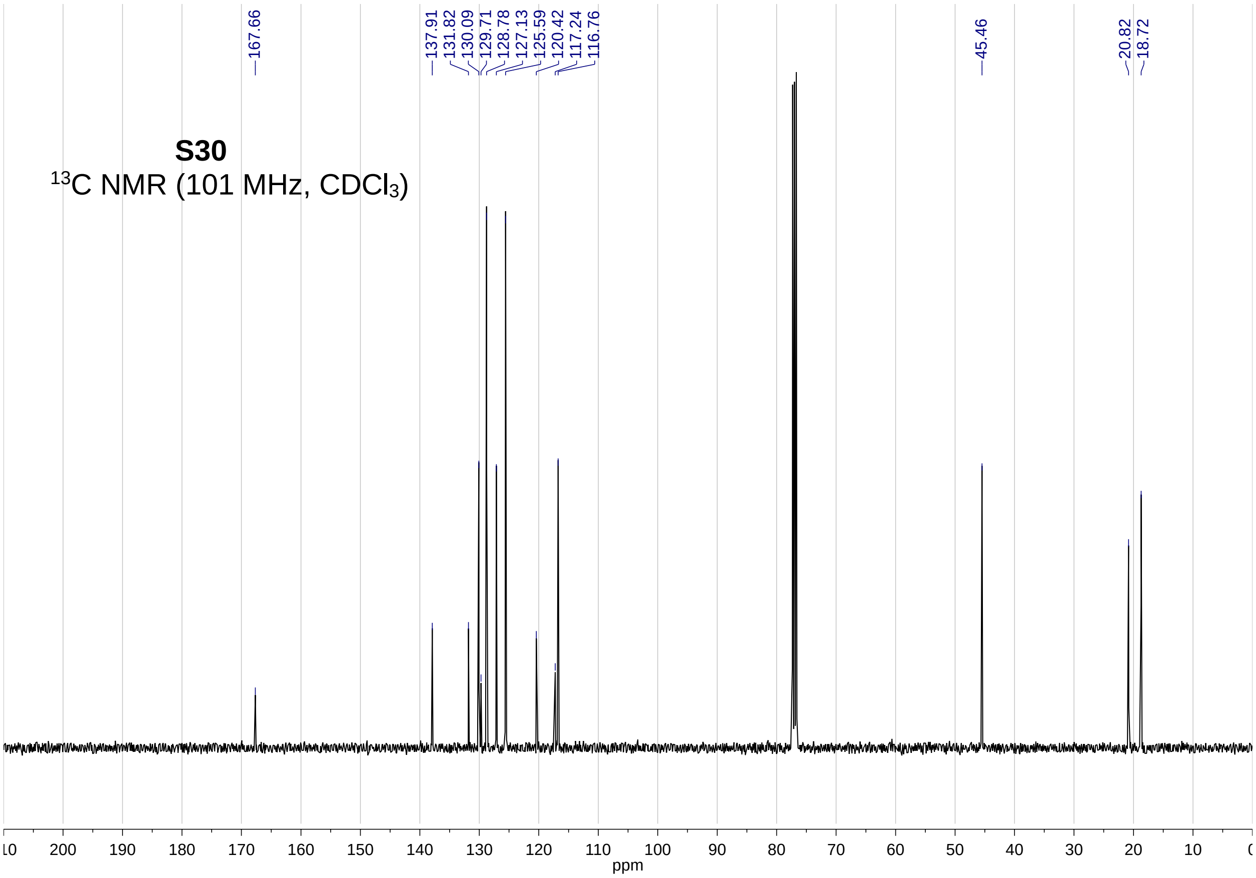

S30

^{13}C NMR (101 MHz, $CDCl_3$)

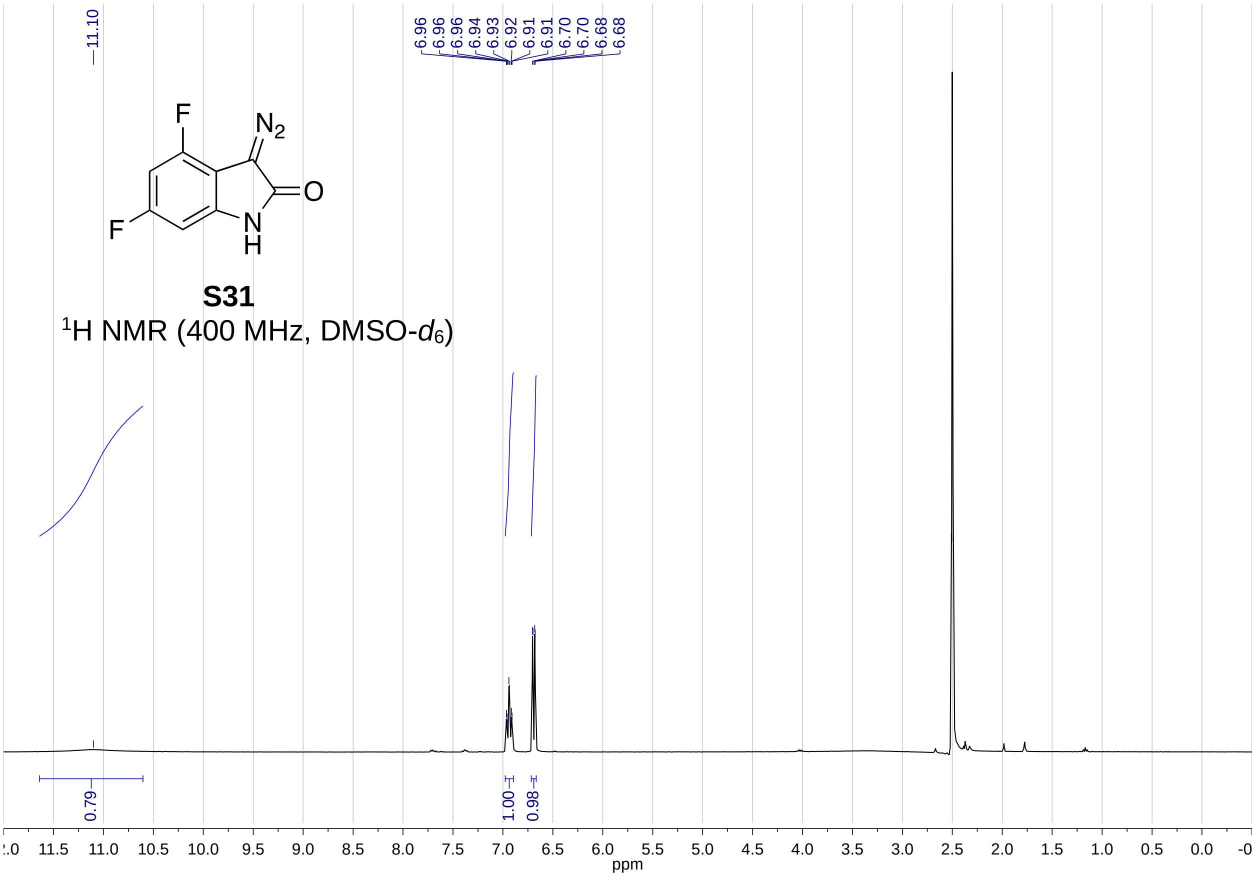

S31

^{1}H NMR (400 MHz, DMSO-d_6)

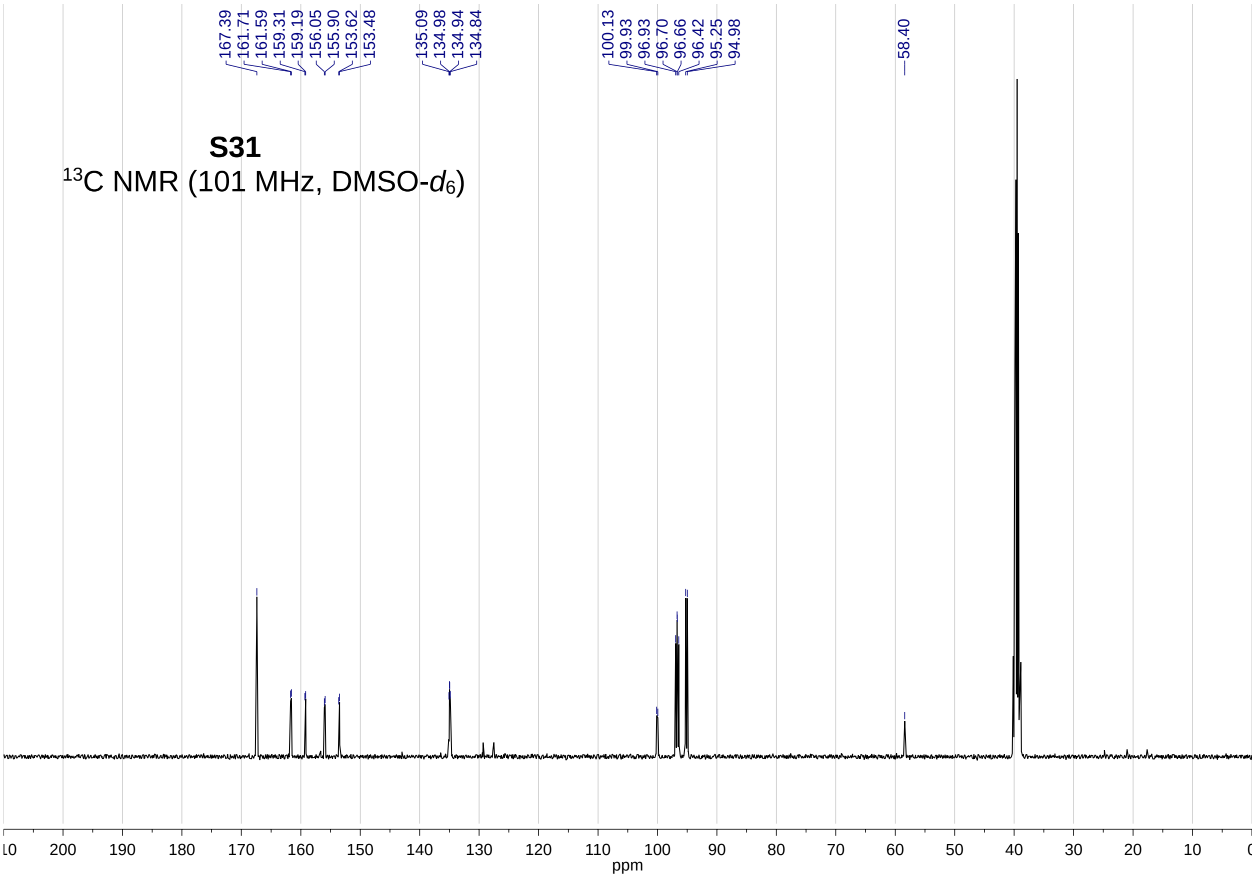

S31

^{13}C NMR (101 MHz, DMSO-d_6)

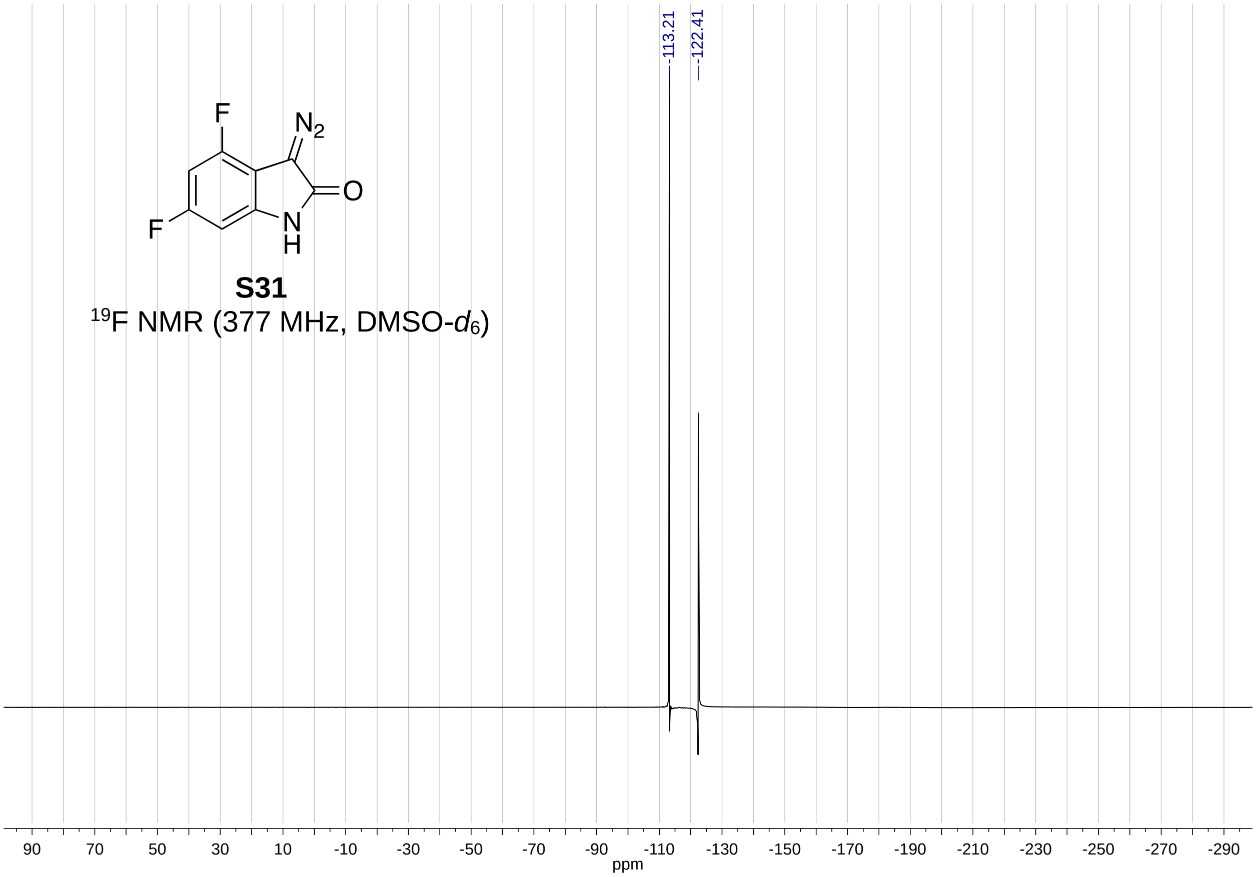

S31
^{19}F NMR (377 MHz, DMSO-d_6)

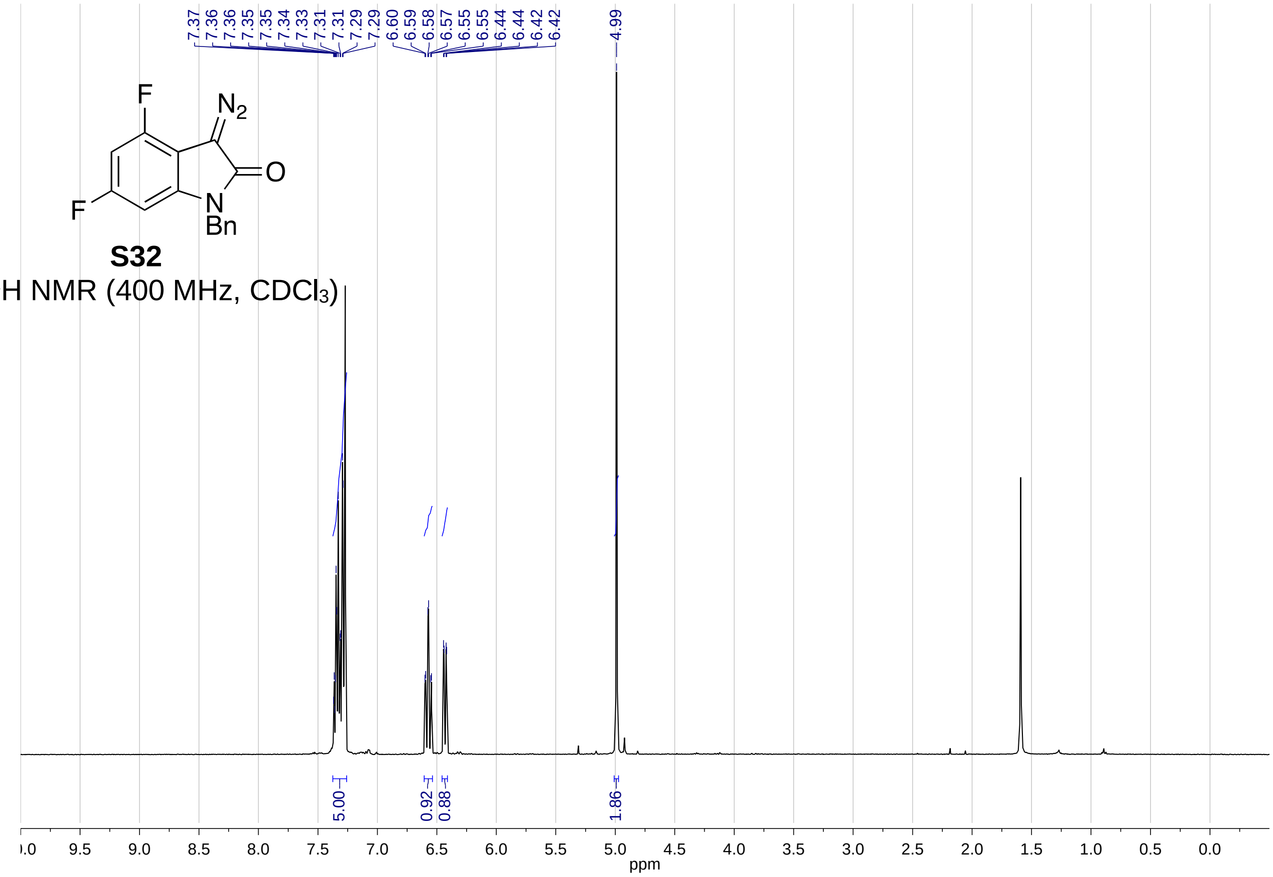

S32

^1H NMR (400 MHz, $CDCl_3$)

S32

^{13}C NMR (101 MHz, $CDCl_3$)

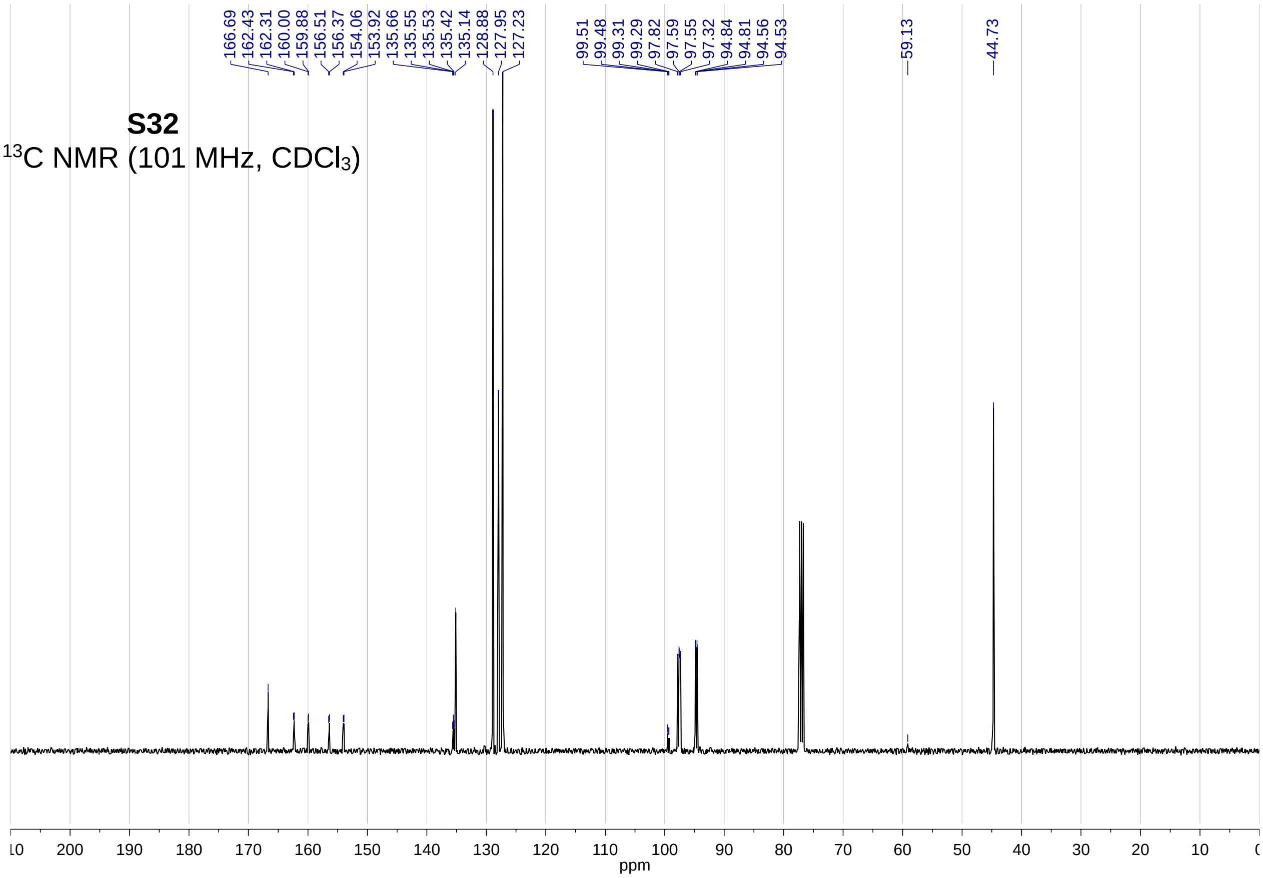

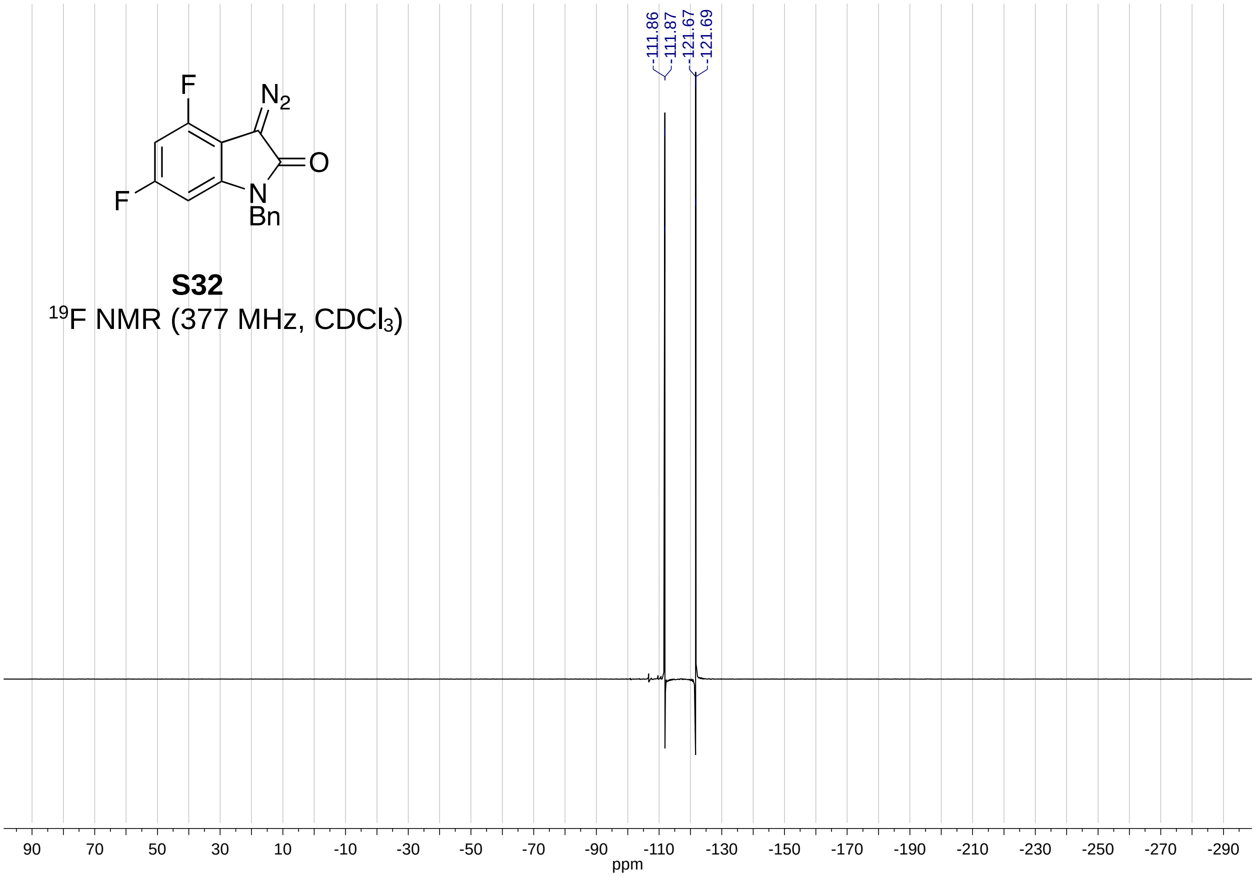

S32

^{19}F NMR (377 MHz, $CDCl_3$)

S40

^{1}H NMR (400 MHz, $CDCl_3$)

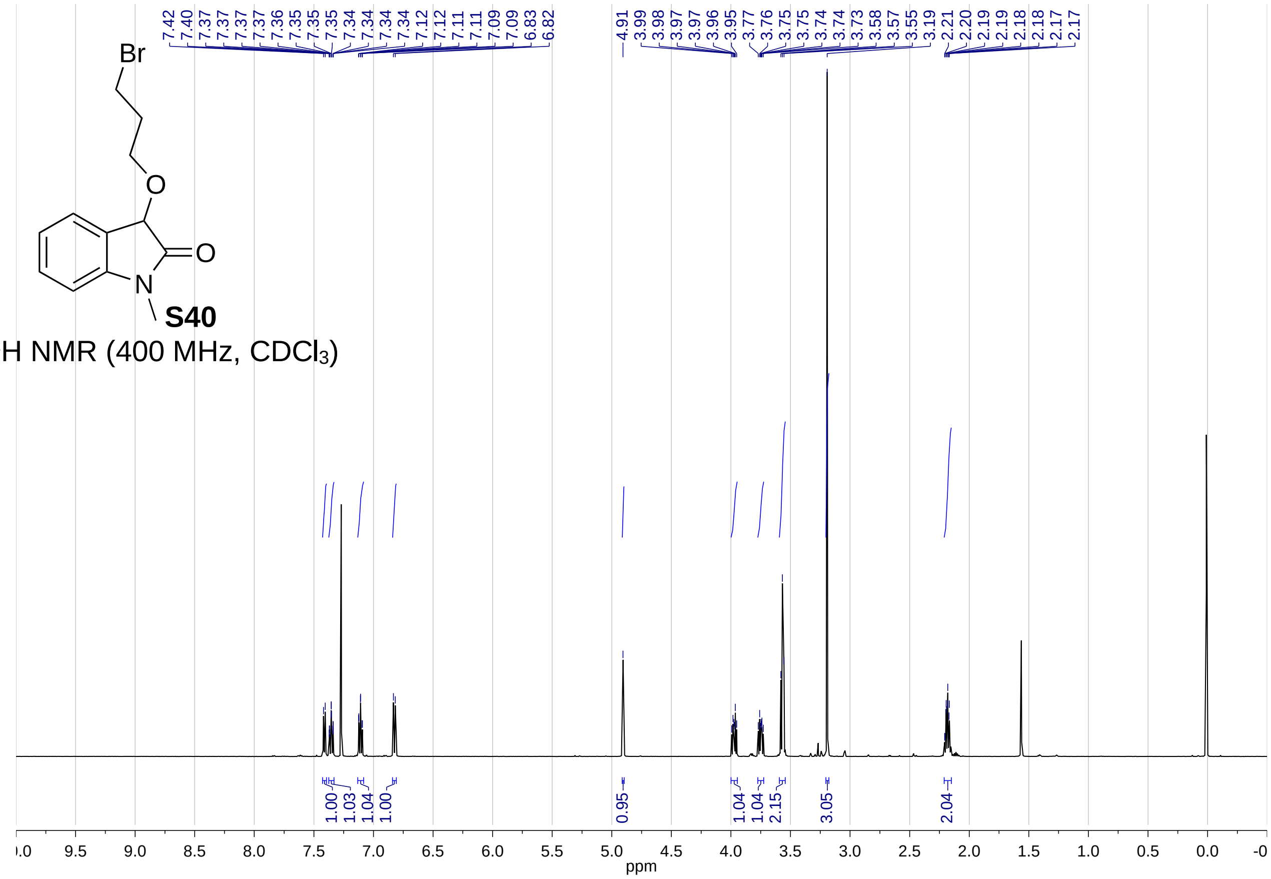

S40

^{13}C NMR (101 MHz, $CDCl_3$)

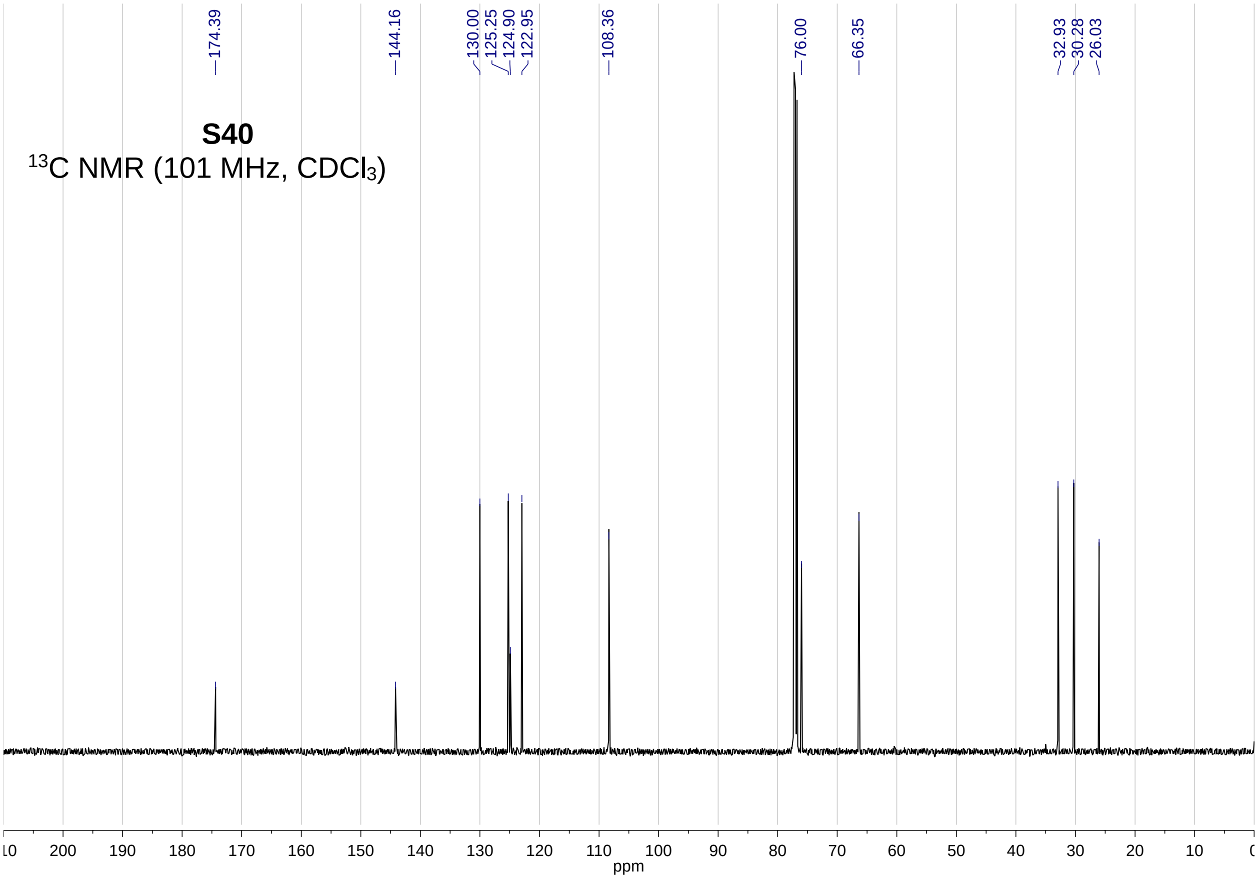

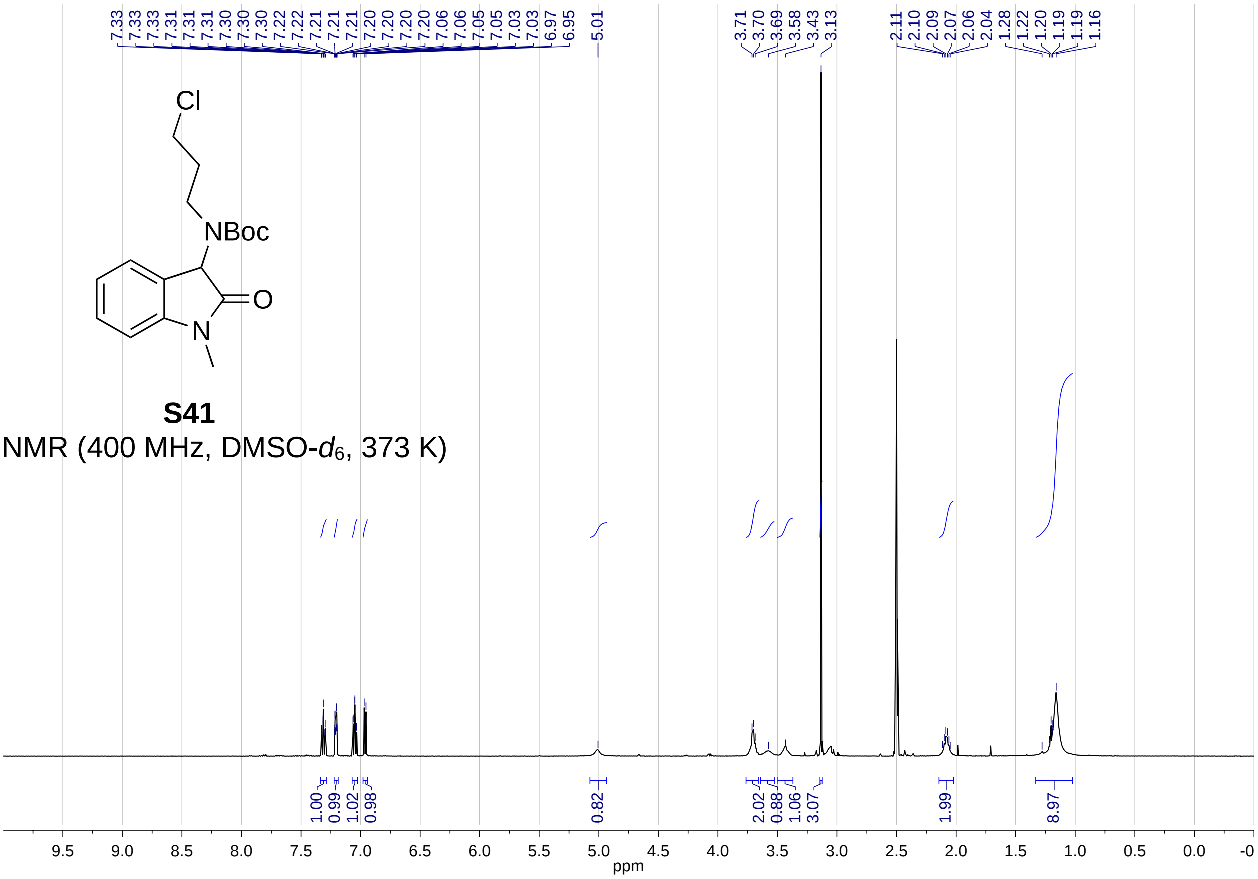

S41

^{1}H NMR (400 MHz, DMSO-d_6, 373 K)

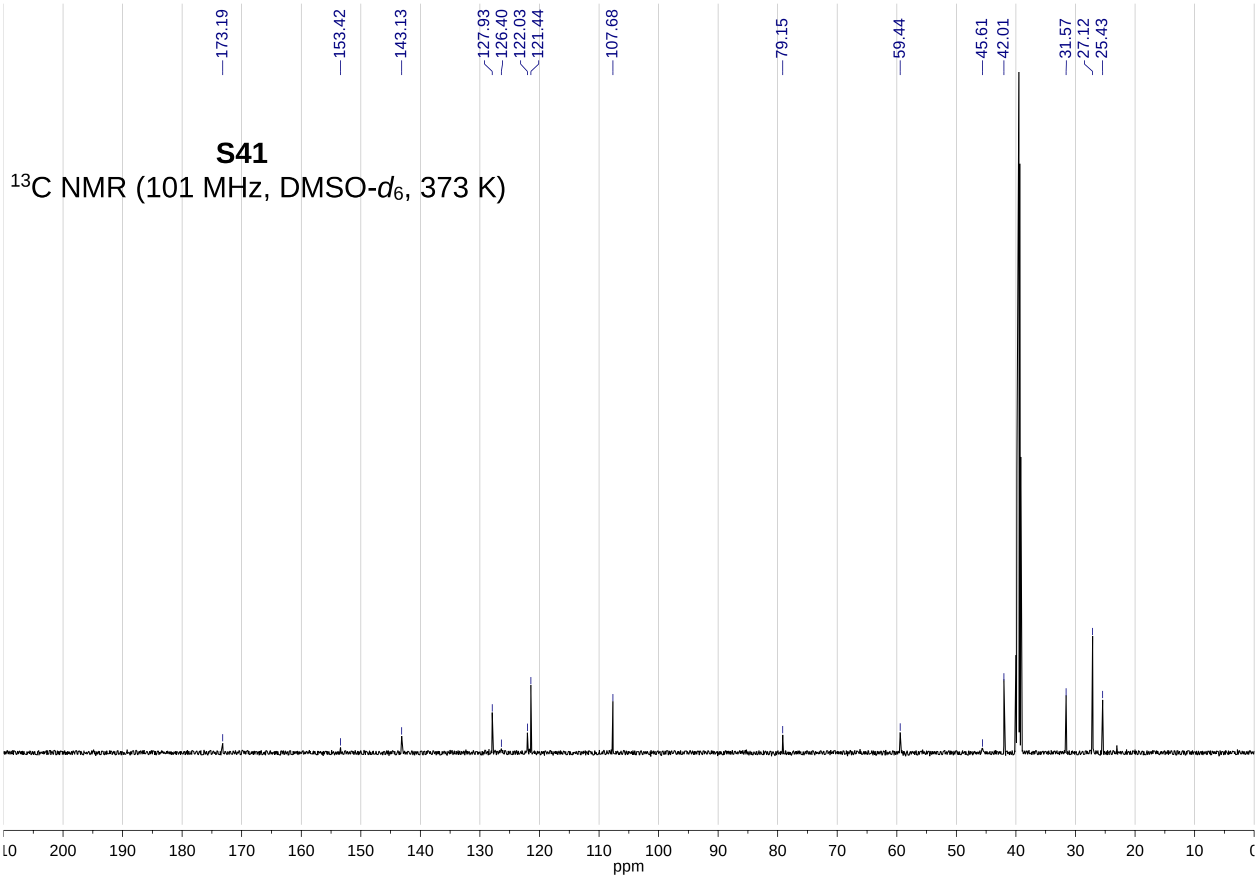

S41

^{13}C NMR (101 MHz, DMSO-d_6, 373 K)

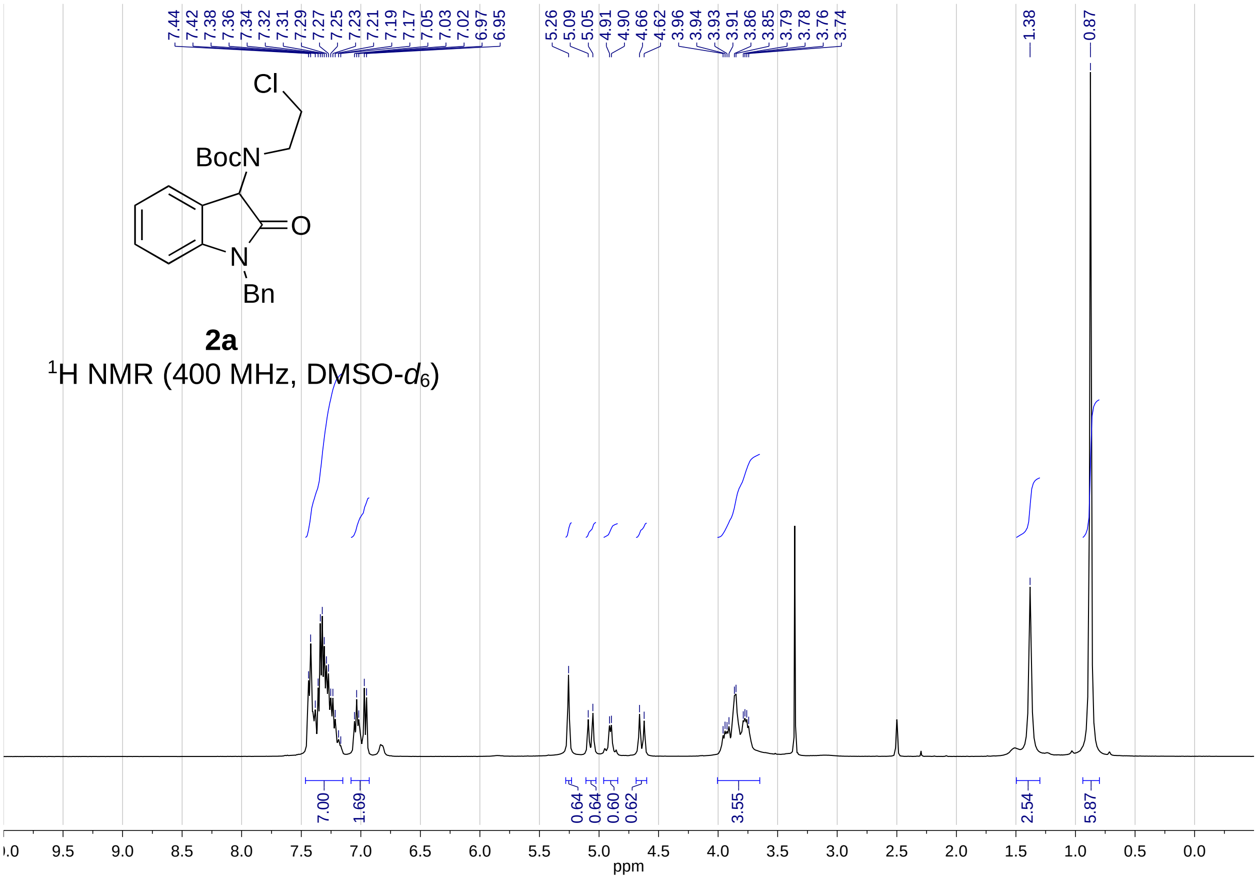

2a

^{1}H NMR (400 MHz, DMSO-d_6)

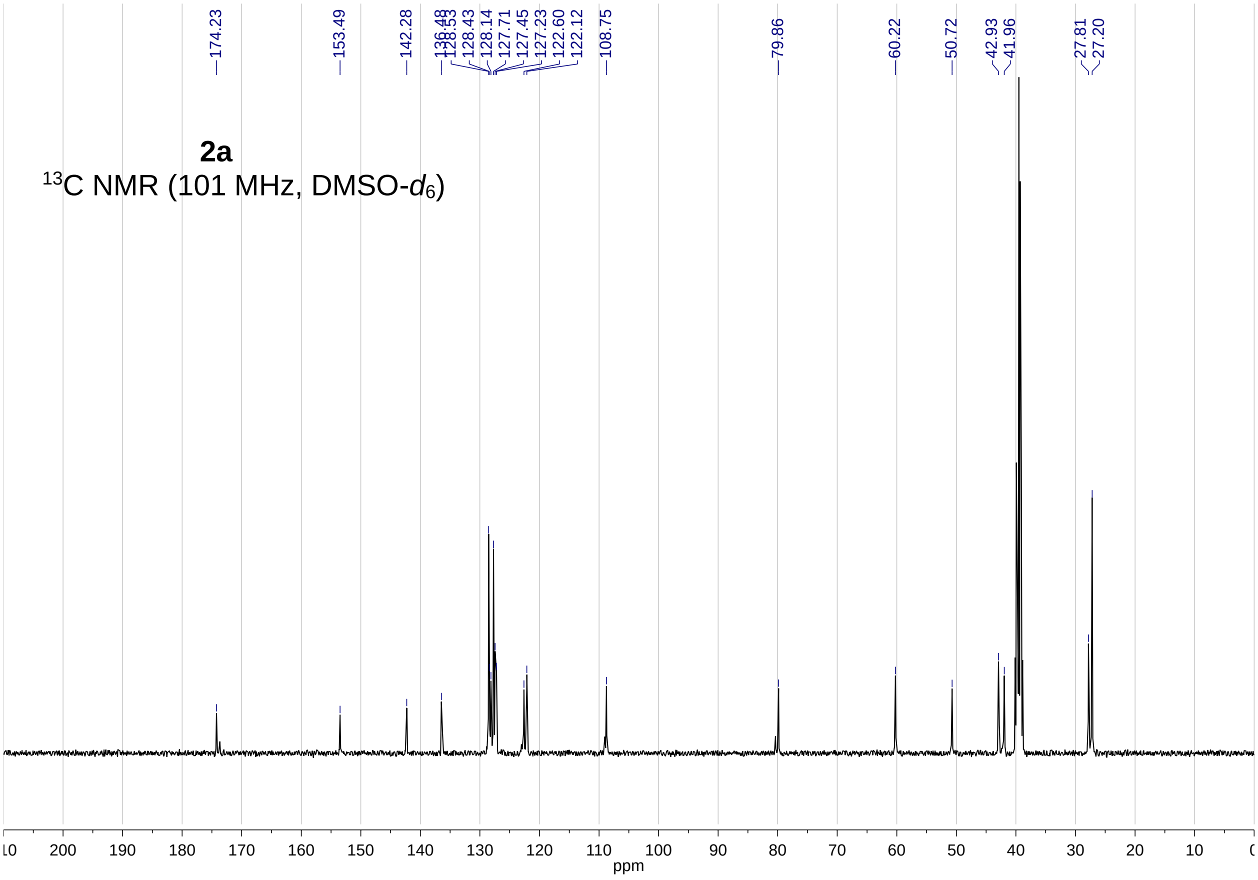

2a

^{13}C NMR (101 MHz, DMSO-d_6)

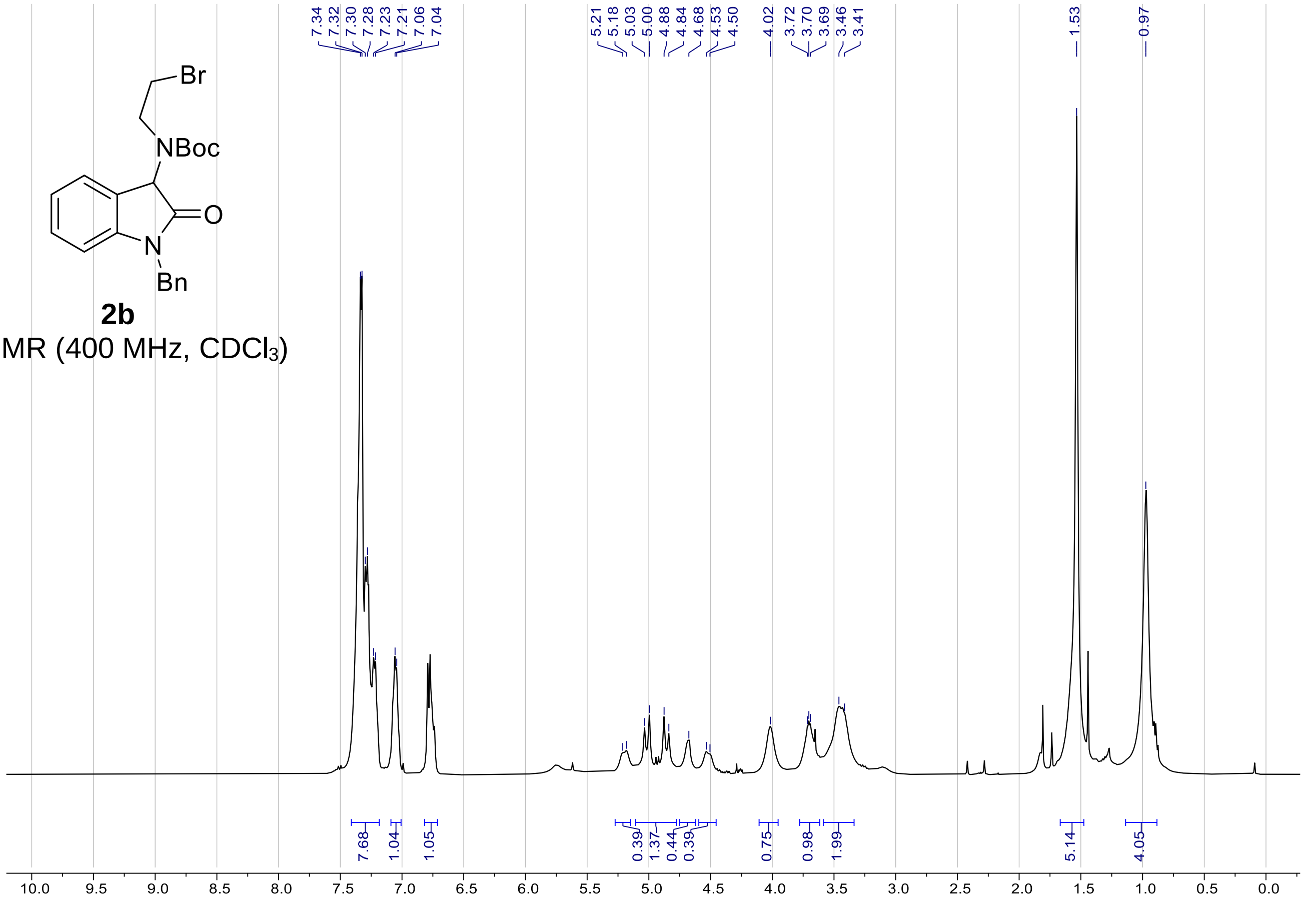

2b

^{1}H NMR (400 MHz, $CDCl_3$)

2b

^{13}C NMR (101 MHz, $CDCl_3$)

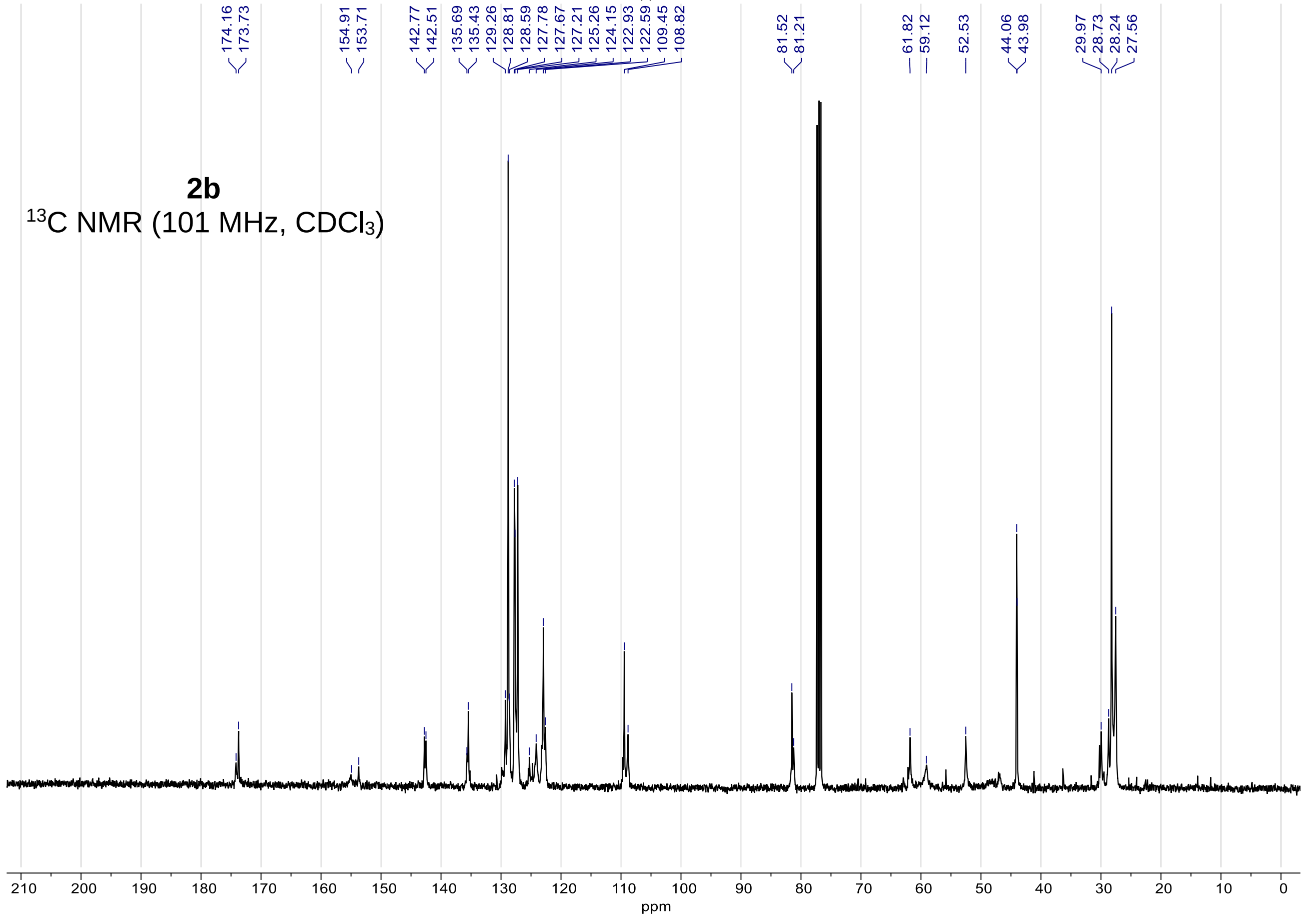

2c

^{1}H NMR (400 MHz, $CDCl_3$)

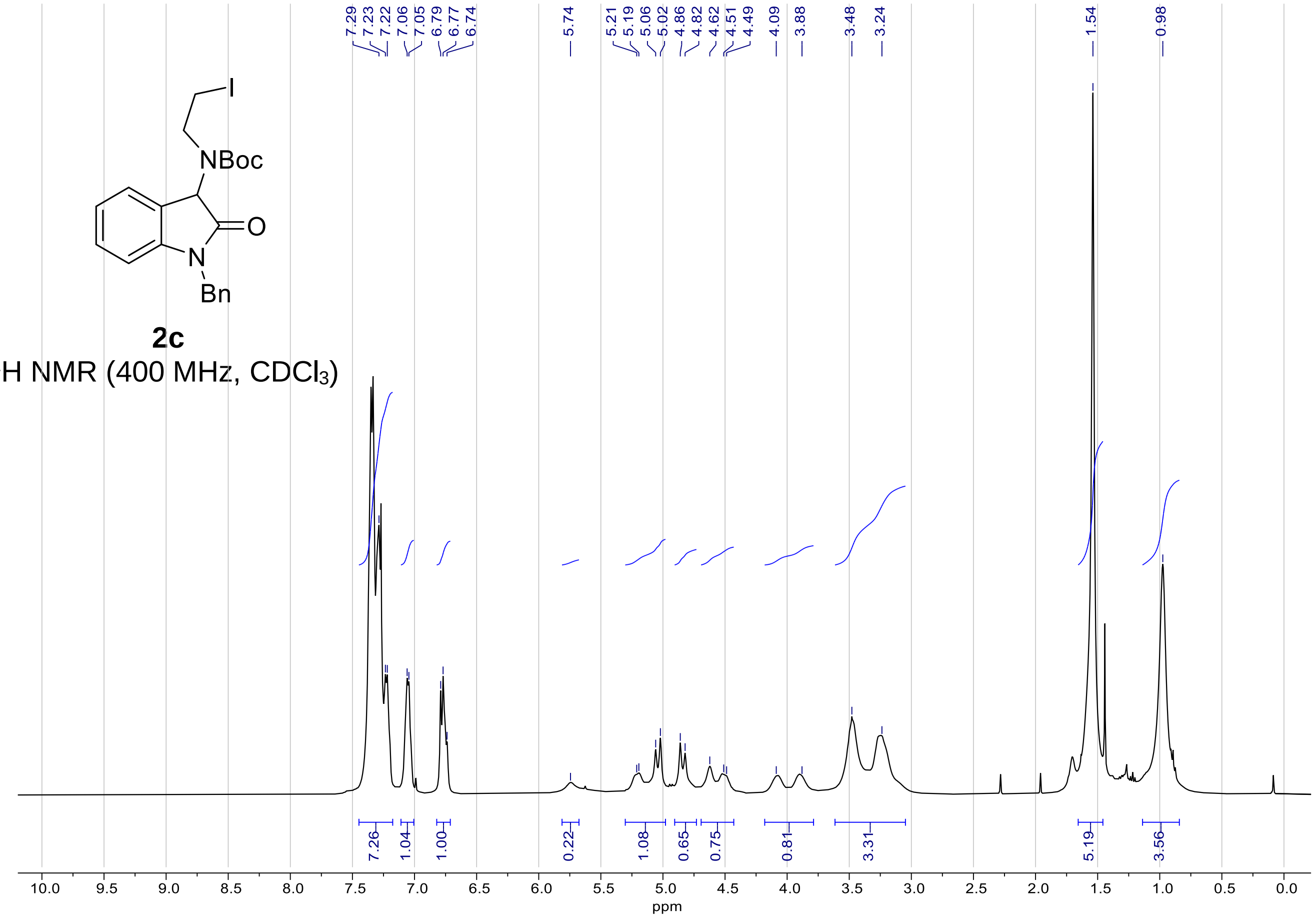

2c

^{13}C NMR (101 MHz, $CDCl_3$)

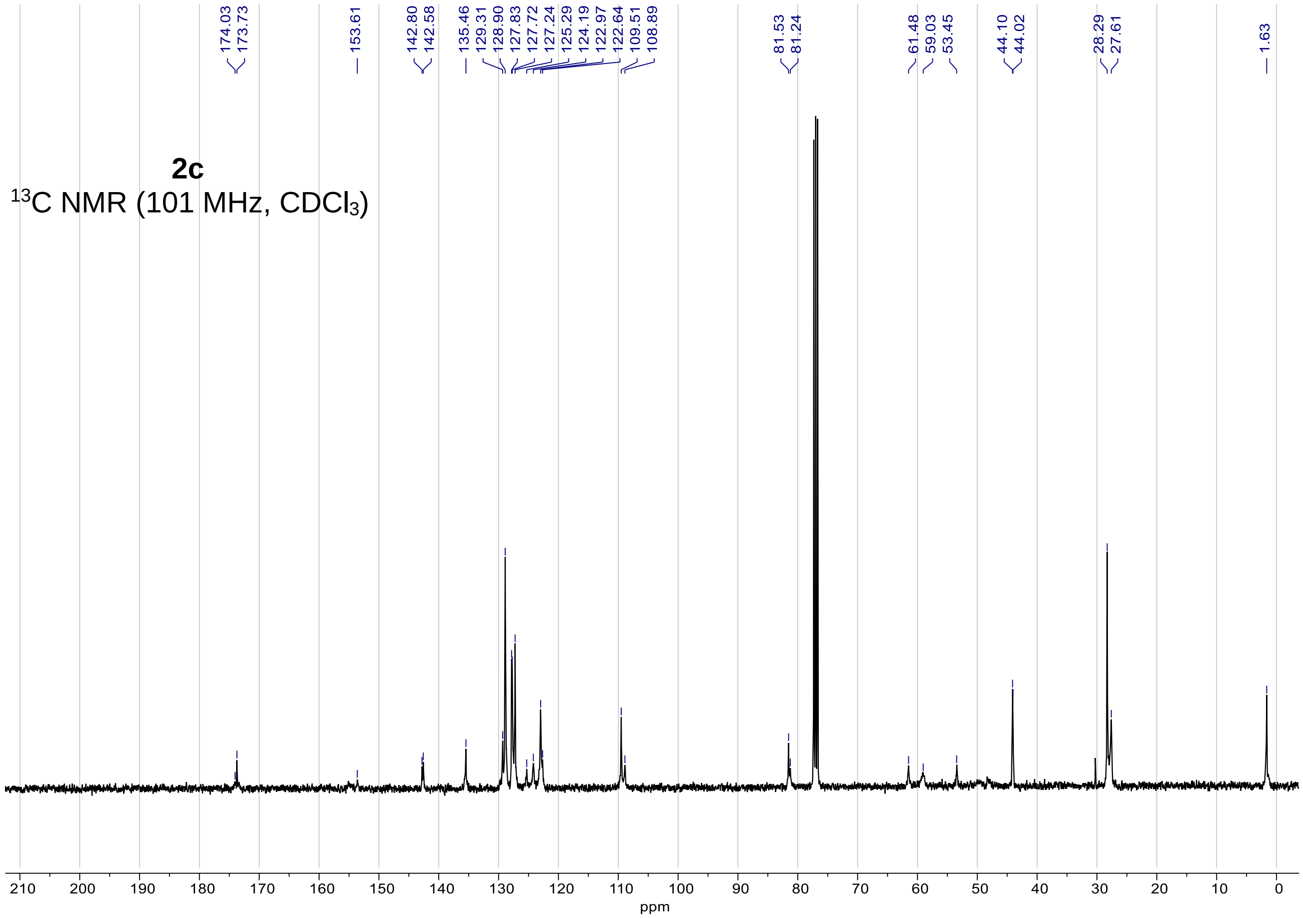

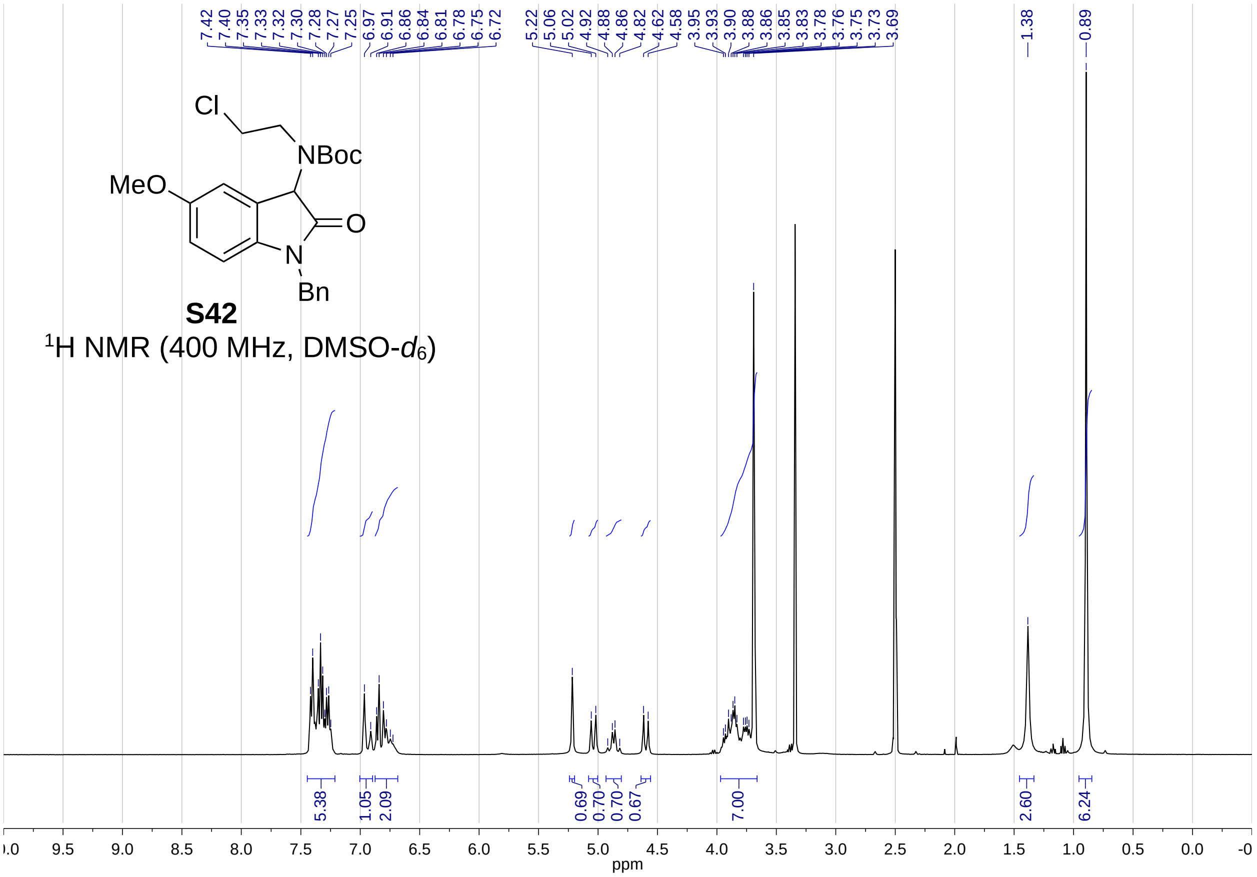

S42

^{1}H NMR (400 MHz, DMSO-d_6)

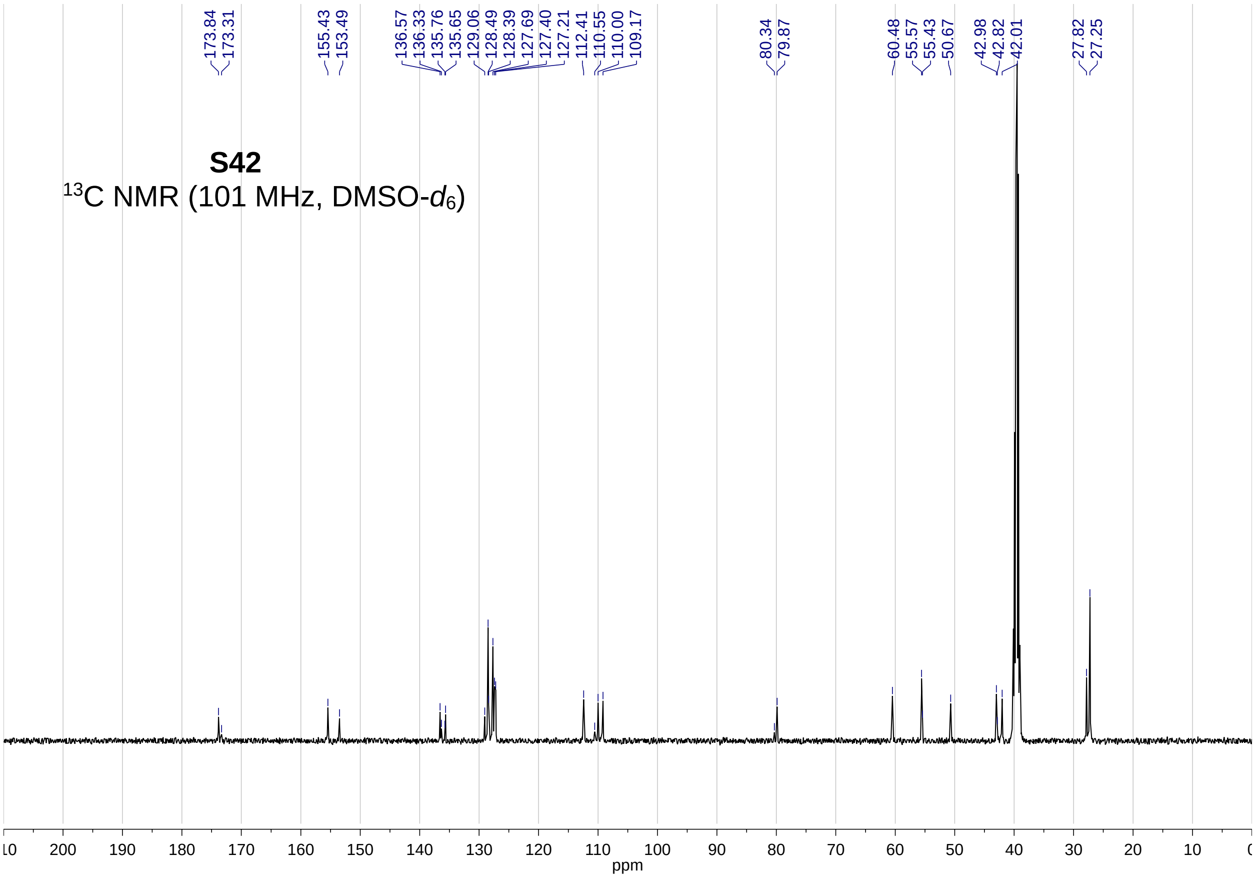

S42

^{13}C NMR (101 MHz, DMSO-d_6)

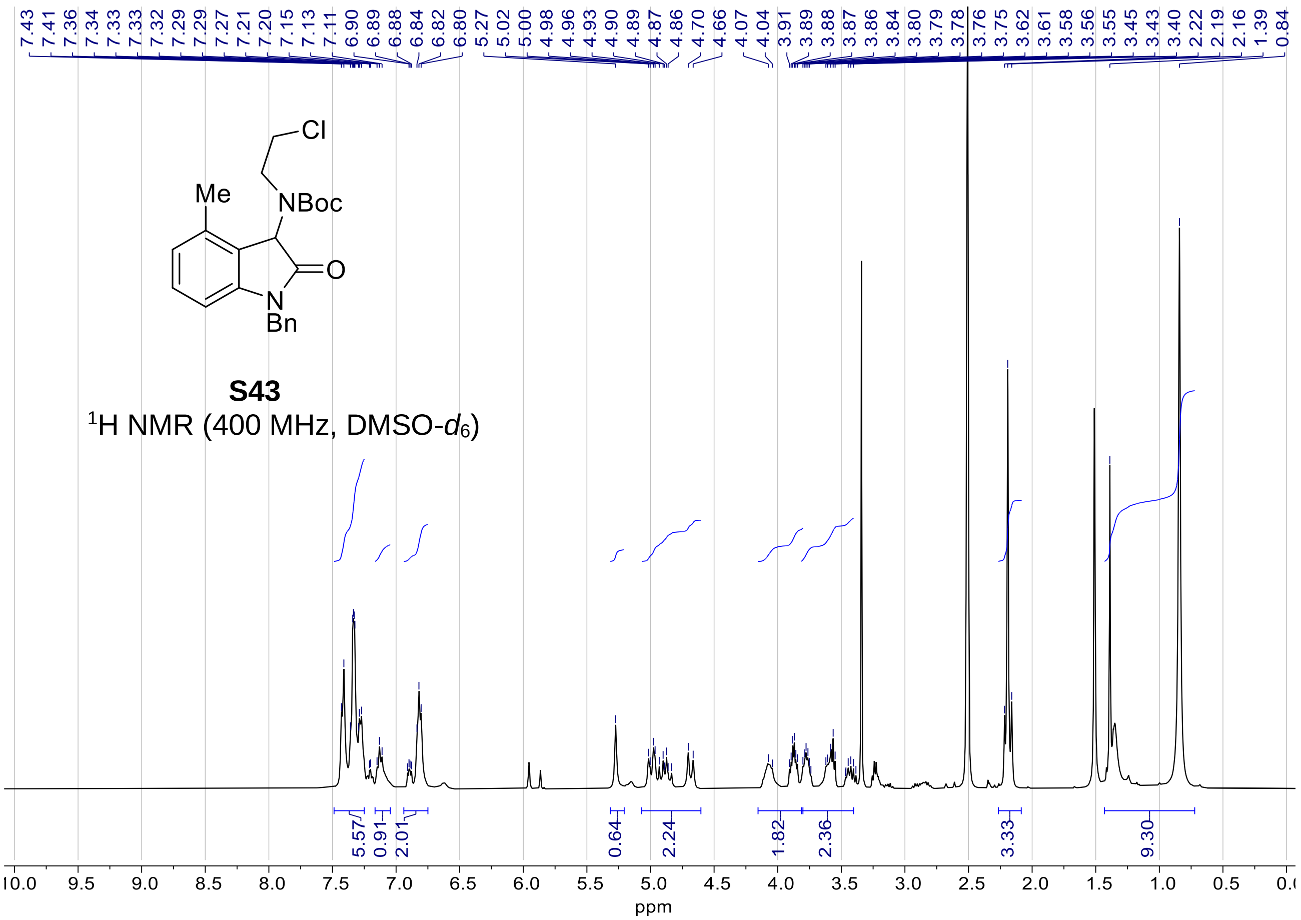
7.43
7.41
7.36
7.34
7.33
7.33
7.32
7.29
7.29
7.27
7.21
7.20
7.15
7.13
7.11
6.90
6.89
6.88
6.84
6.82
6.80
5.27
5.02
5.00
4.98
4.96
4.93
4.90
4.89
4.87
4.86
4.70
4.66
4.07
4.04
3.91
3.89
3.88
3.87
3.86
3.84
3.80
3.79
3.78
3.76
3.75
3.62
3.61
3.58
3.56
3.55
3.45
3.43
3.40
2.22
2.19
2.16
1.39
0.84
Cl
Me
NBoc
O
N
Bn
S43
1H NMR (400 MHz, DMSO-d6)
5.57
0.91
2.01
0.64
2.24
1.82
2.36
3.33
9.30
ppm

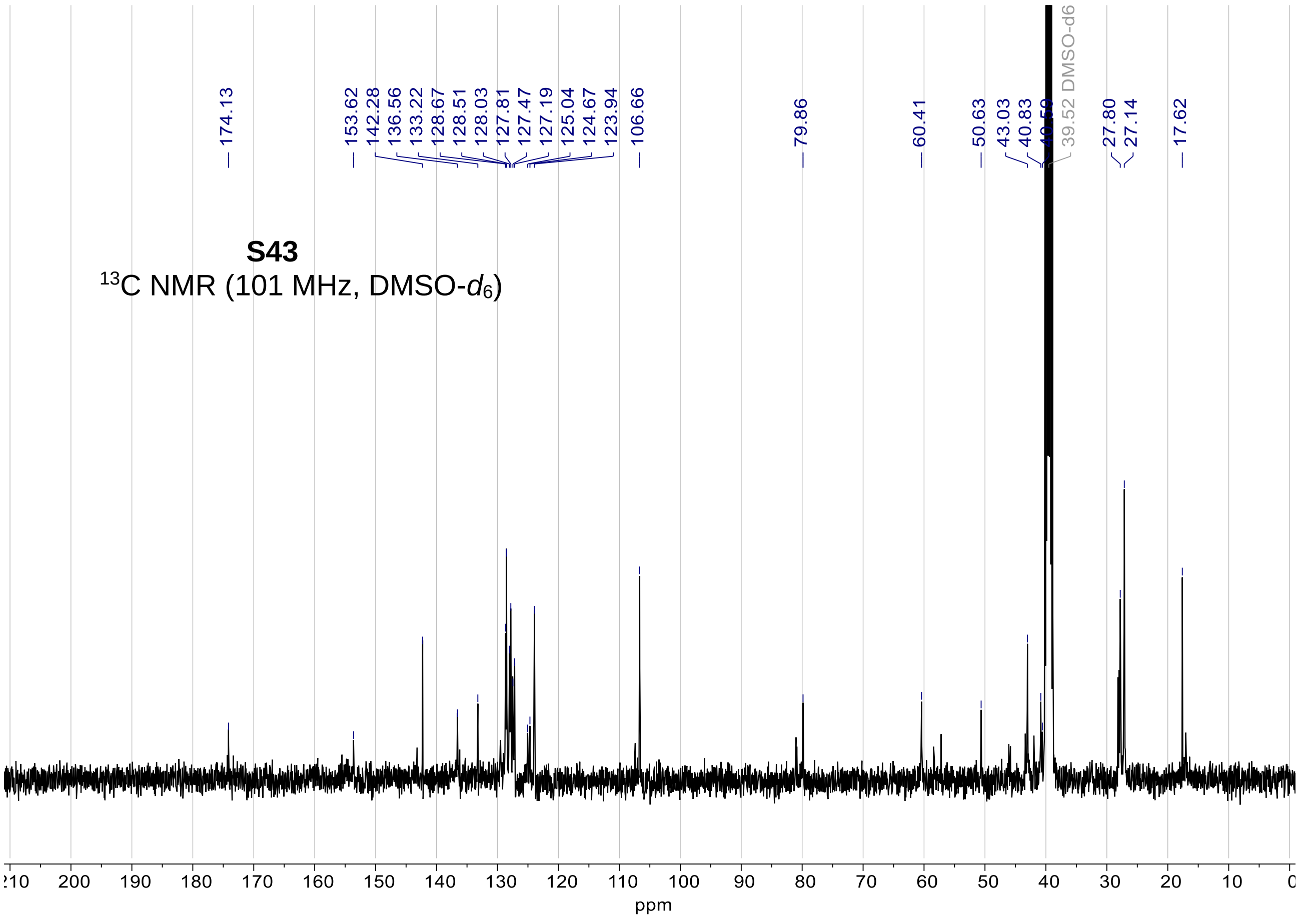
174.13
153.62
142.28
136.56
133.22
128.67
128.51
128.03
127.81
127.47
127.19
125.04
124.67
123.94
106.66
79.86
60.41
50.63
43.03
40.83
39.52 DMSO-d6
27.80
27.14
17.62
S43
13C NMR (101 MHz, DMSO-d6)
ppm

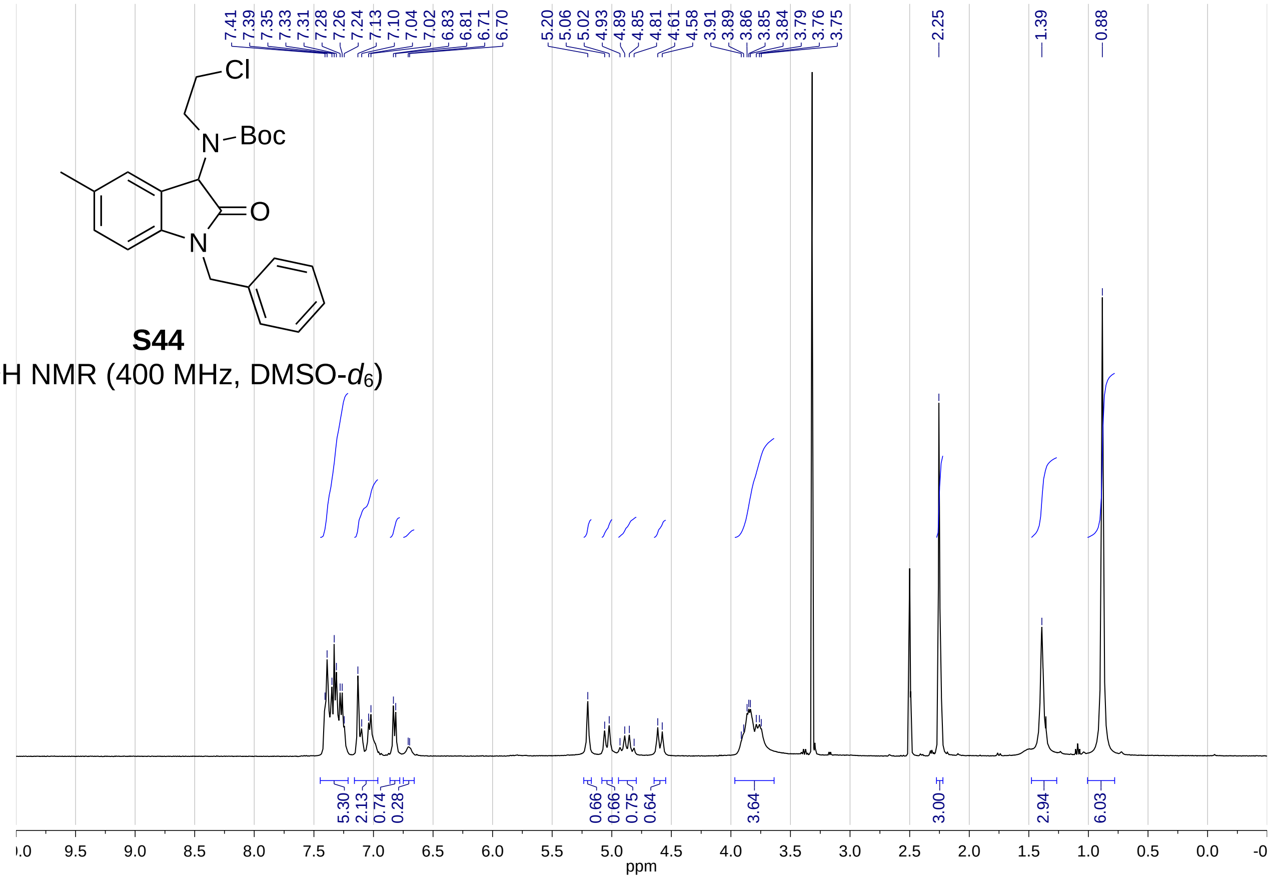

S44
^{1}H NMR (400 MHz, DMSO-d_6)

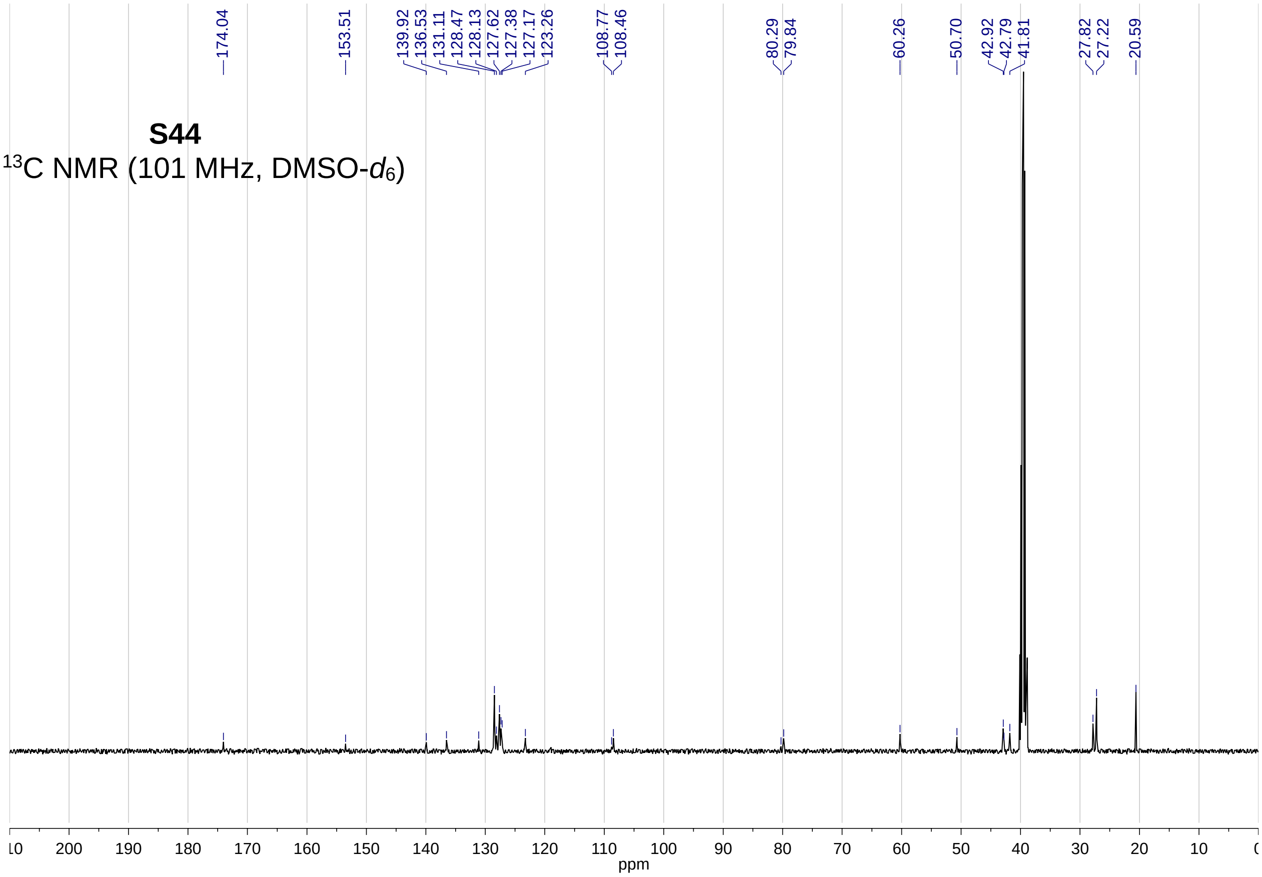

S44
^{13}C NMR (101 MHz, DMSO-d_6)

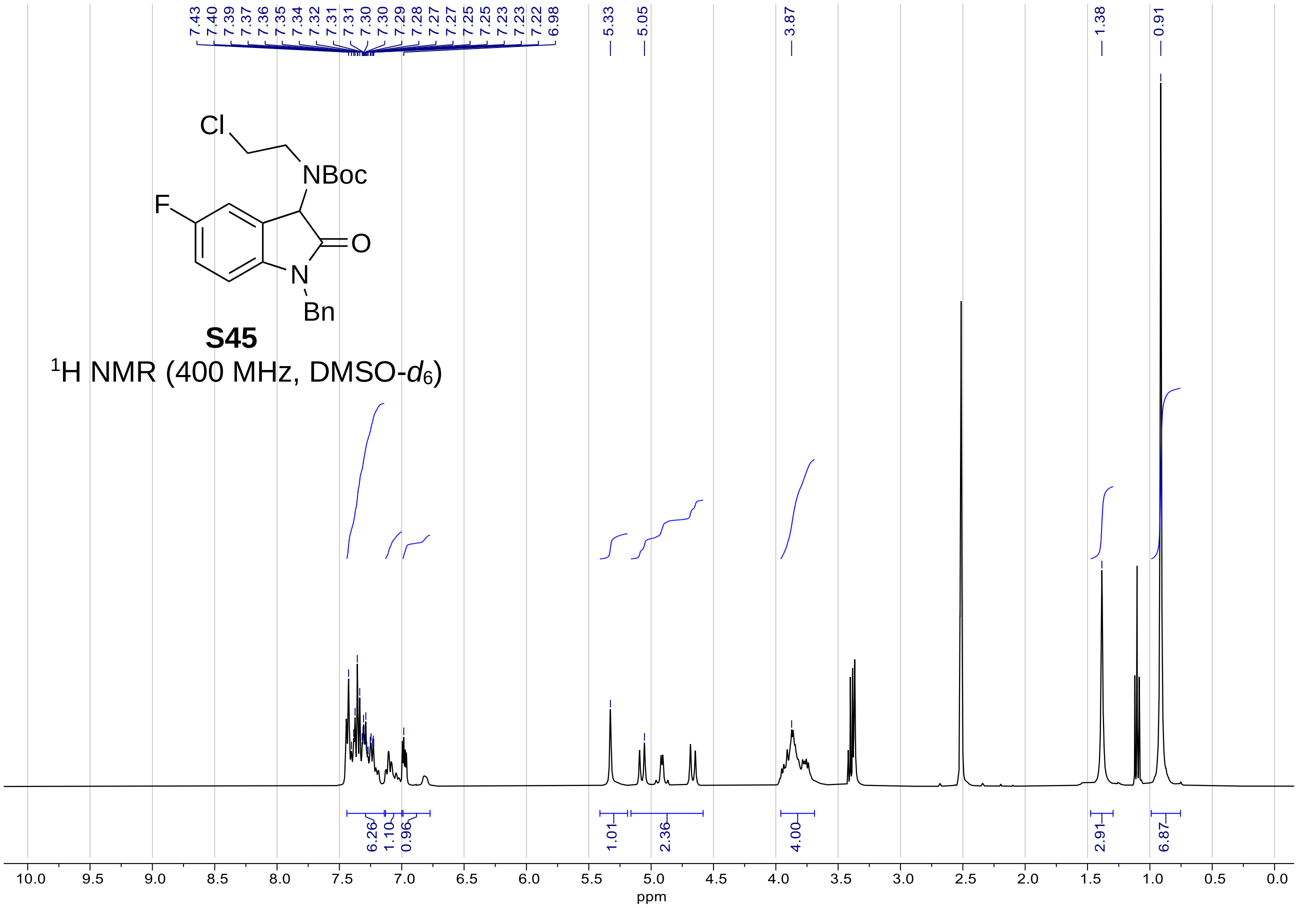
7.43 7.40 7.39 7.37 7.36 7.35 7.34 7.32 7.31 7.31 7.30 7.30 7.29 7.28 7.27 7.27 7.25 7.25 7.23 7.23 7.22 6.98
5.33
5.05
3.87
1.38
0.91
Cl
NBoc
F
O
N
Bn
S45
1H NMR (400 MHz, DMSO-d6)
6.26
1.10
0.96
1.01
2.36
4.00
2.91
6.87
ppm

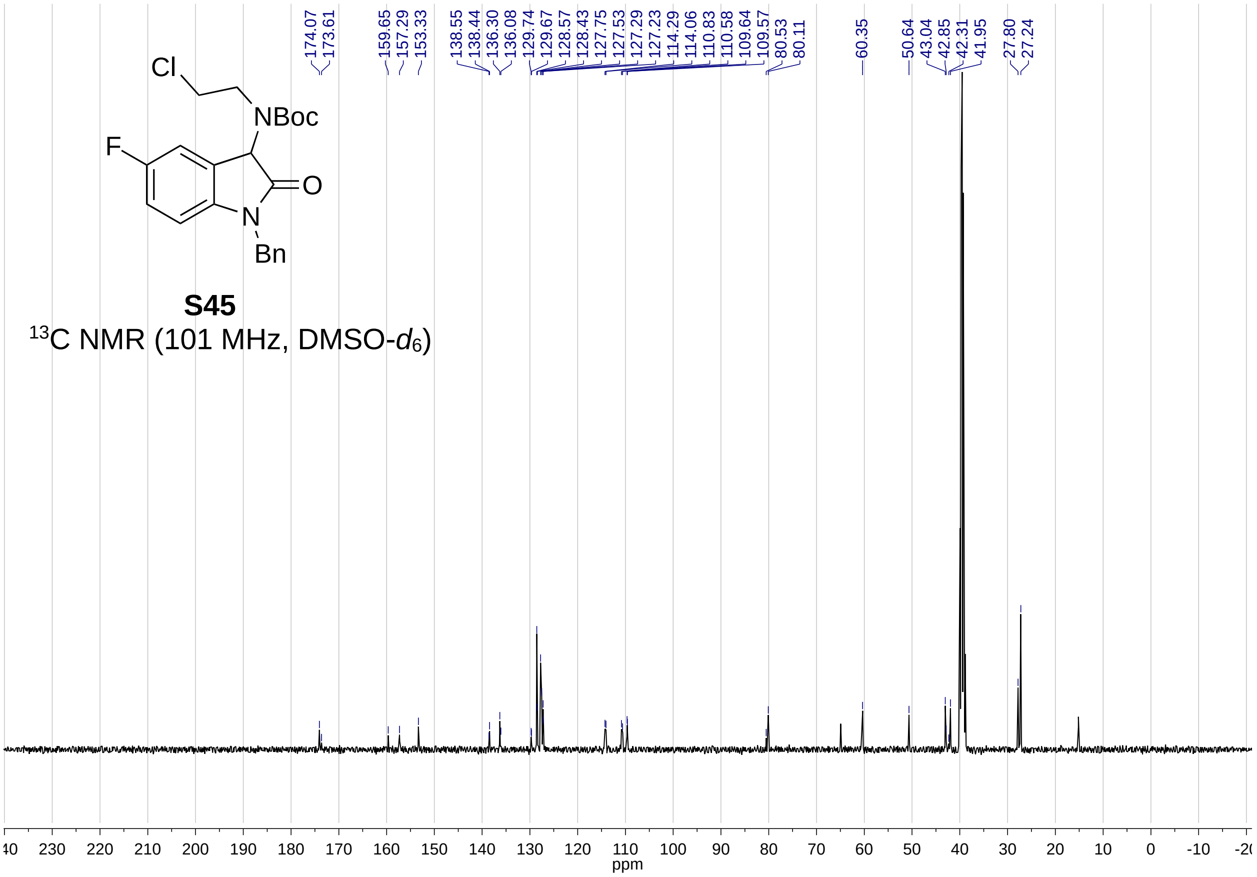
174.07 173.61
159.65 157.29 153.33
138.55 138.44 136.30 136.08 129.74 129.67 128.57 128.43 127.75 127.53 127.29 127.23 114.29 114.06 110.83 110.58 109.64 109.57 80.53 80.11
60.35
50.64 43.04 42.85 42.31 41.95
27.80 27.24
Cl
NBoc
F
O
N
Bn
S45
13C NMR (101 MHz, DMSO-d6)
ppm

S45
^{19}F NMR (377 MHz, DMSO-d_6)

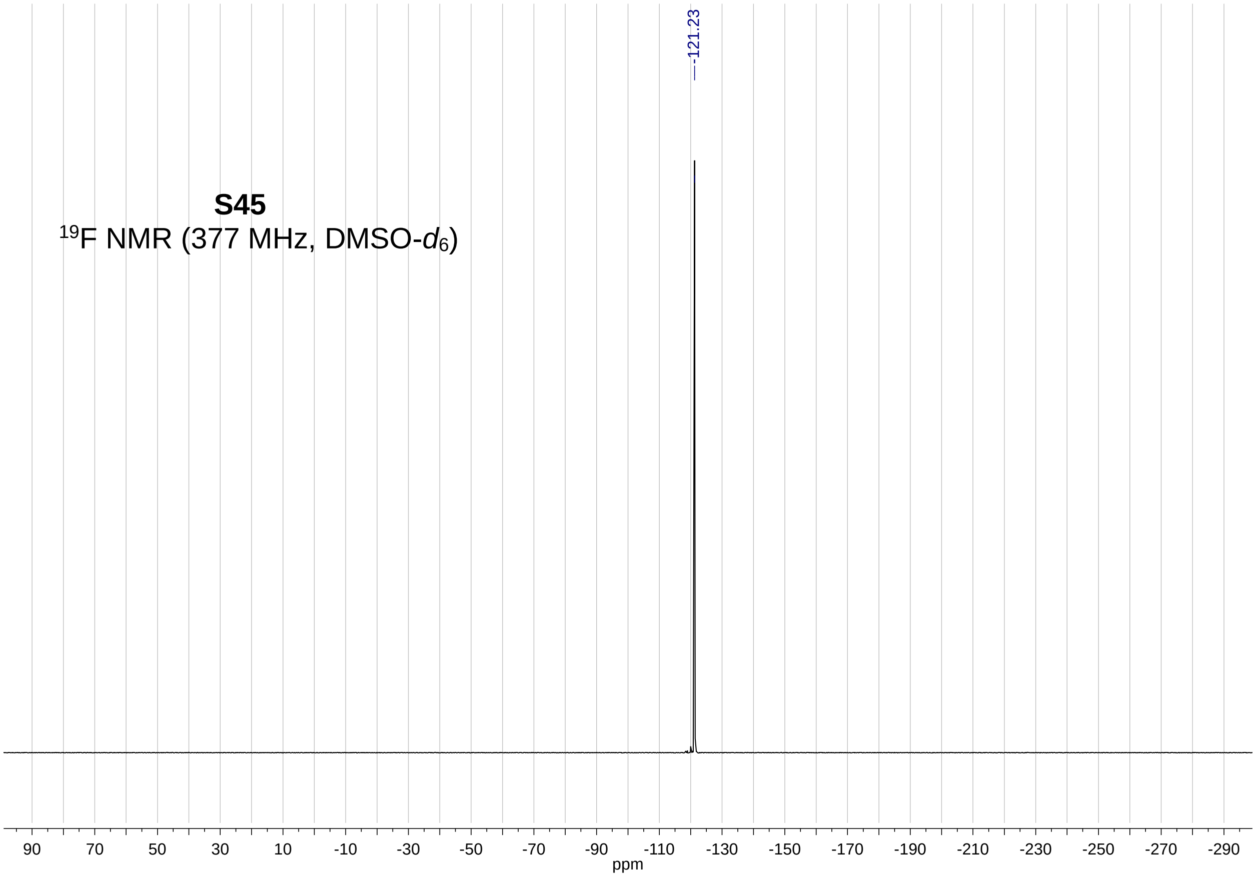

S46

^{1}H NMR (400 MHz, DMSO-d_6)

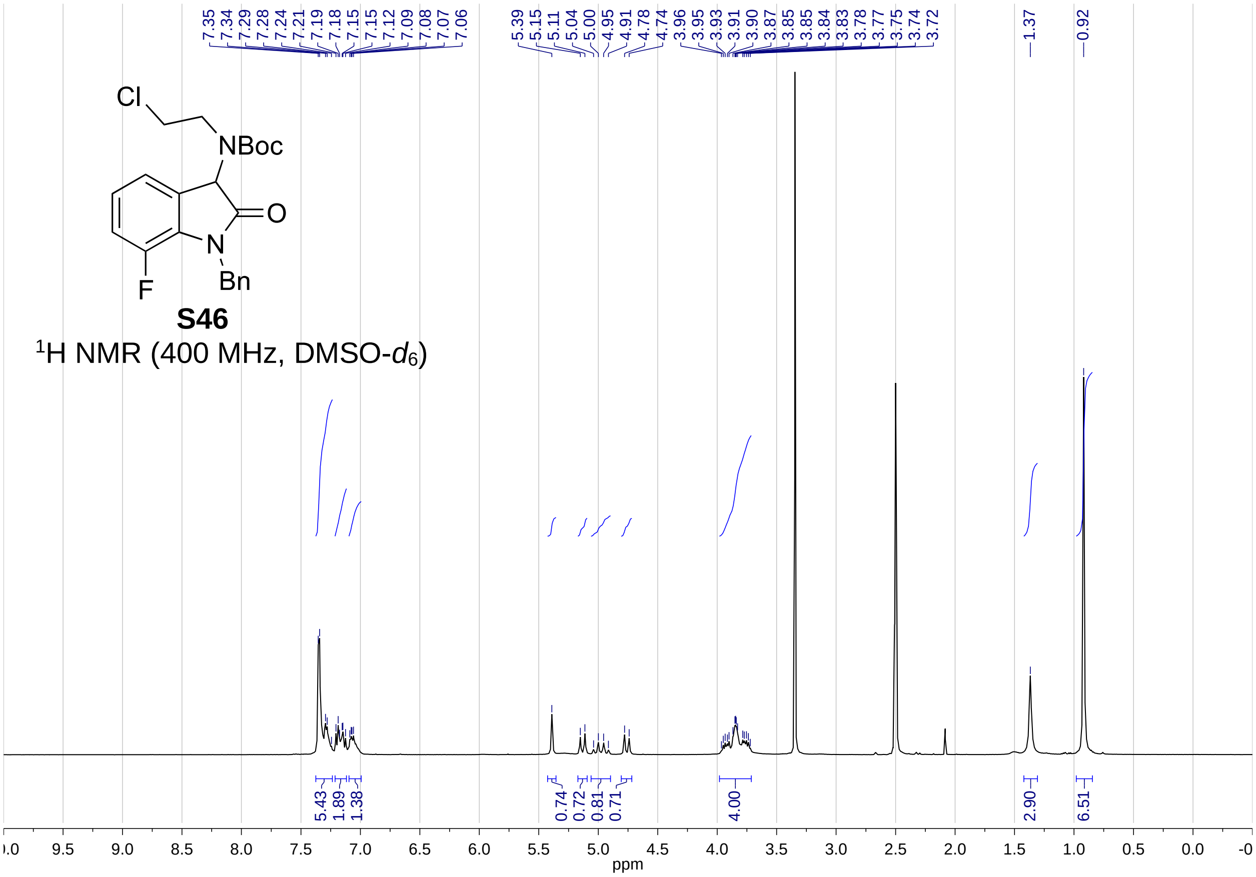

S46

^{13}C NMR (101 MHz, DMSO-d_6)

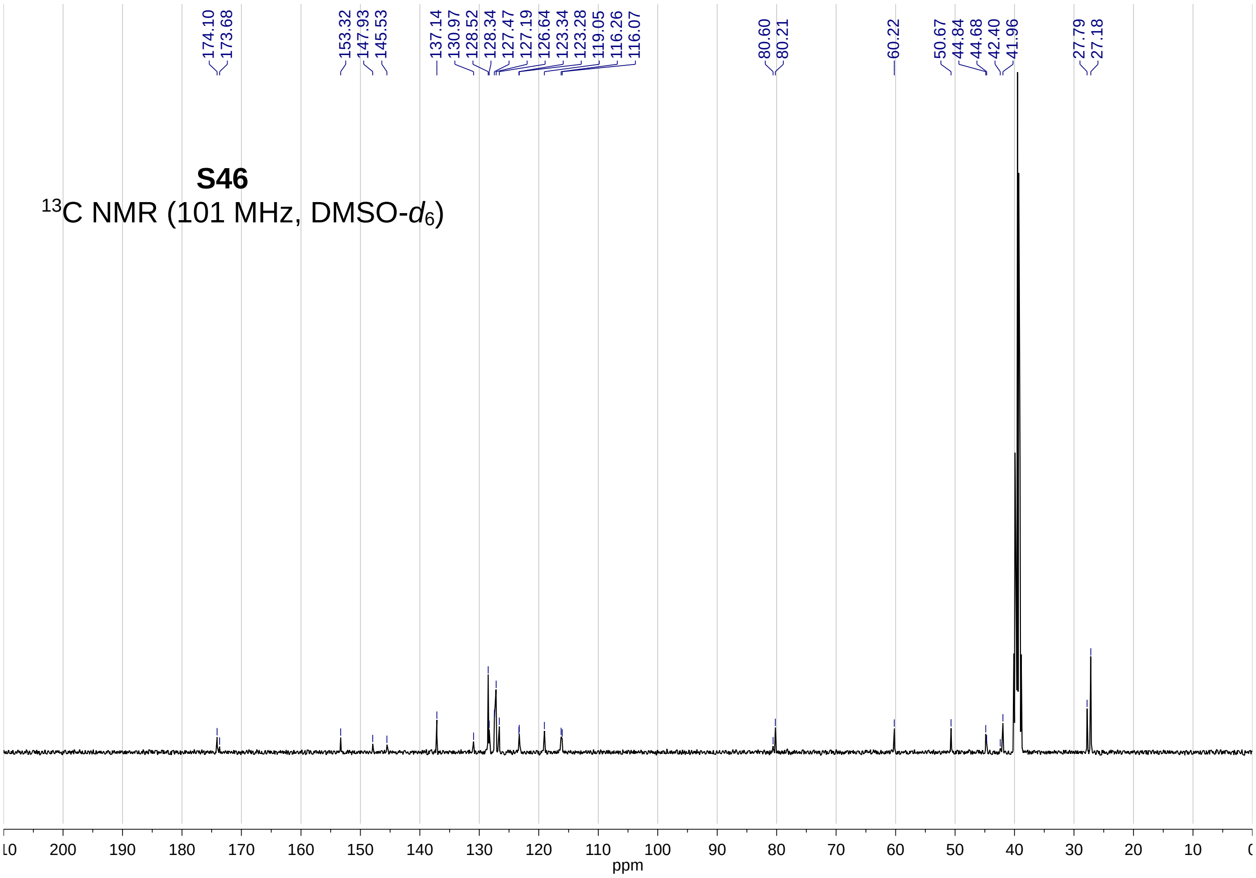

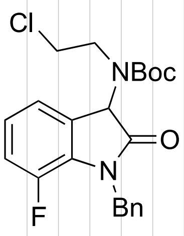

S46

^{19}F NMR (377 MHz, DMSO-d_6)

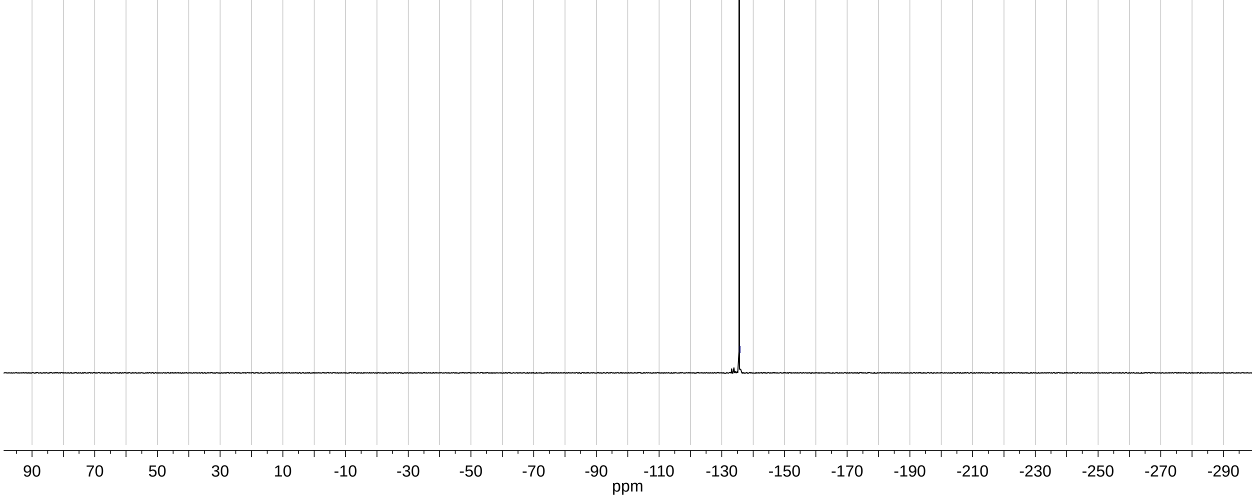

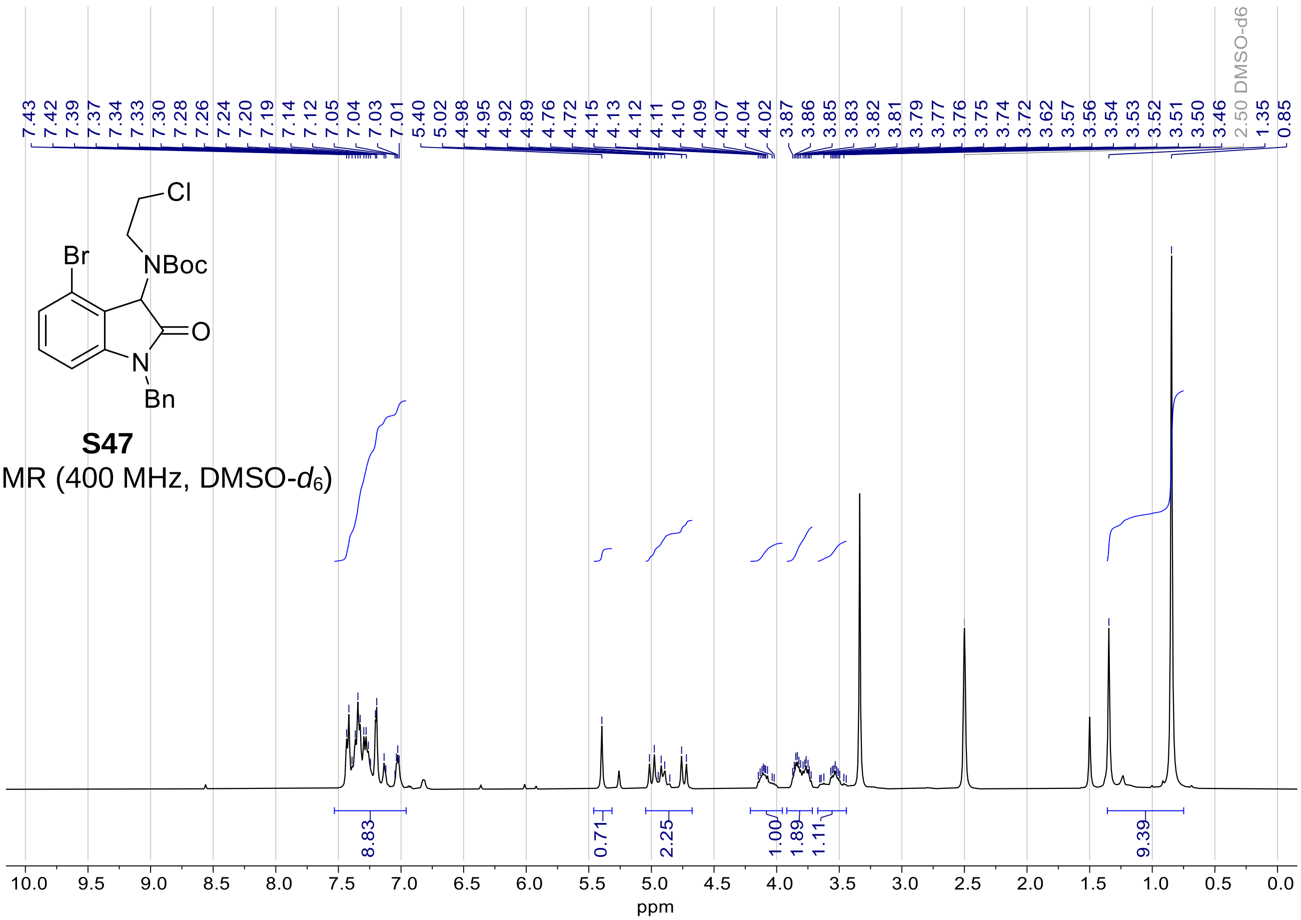

S47

^{1}H NMR (400 MHz, DMSO-d_6)

S47

^{13}C NMR (101 MHz, DMSO-d_6)

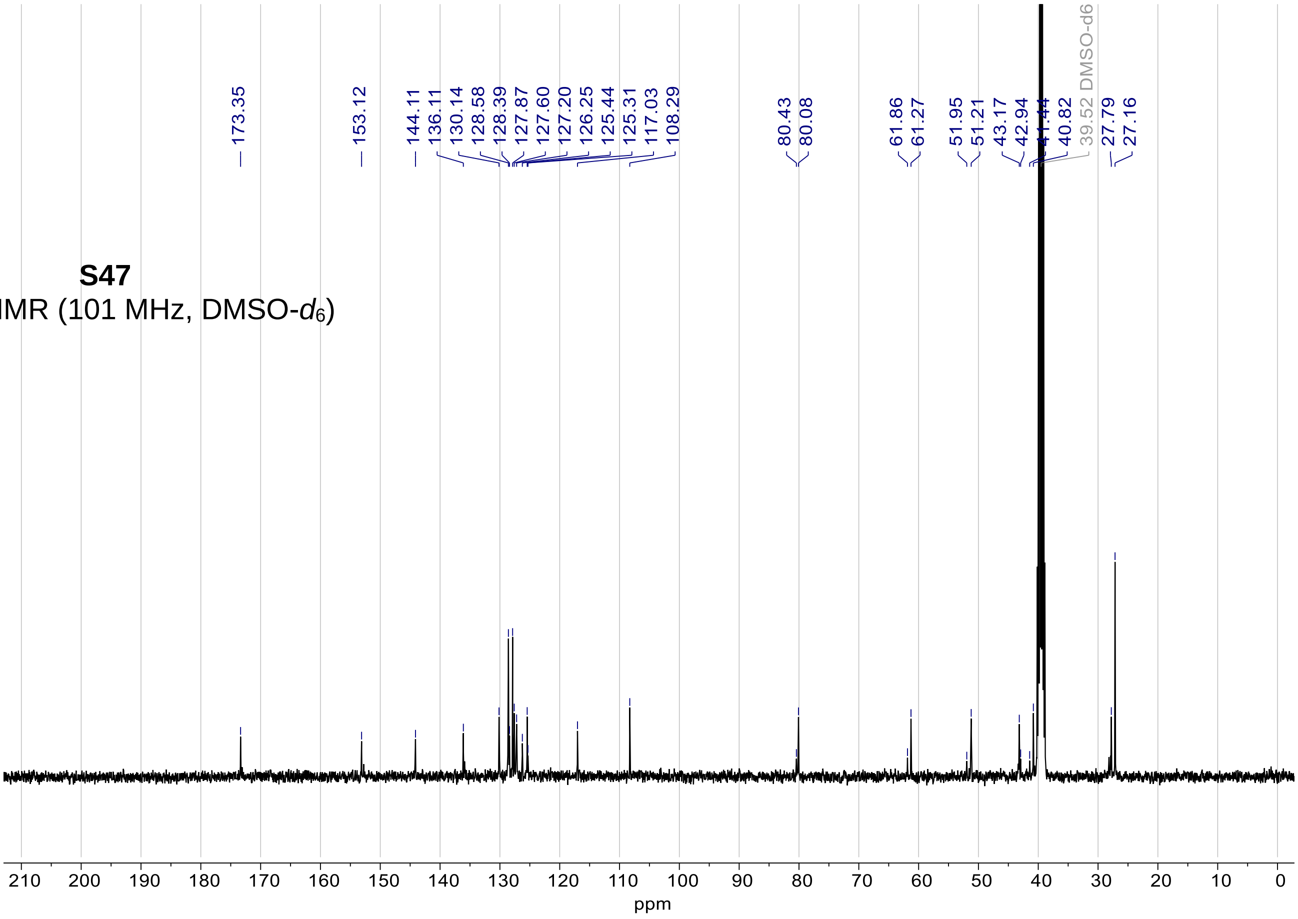

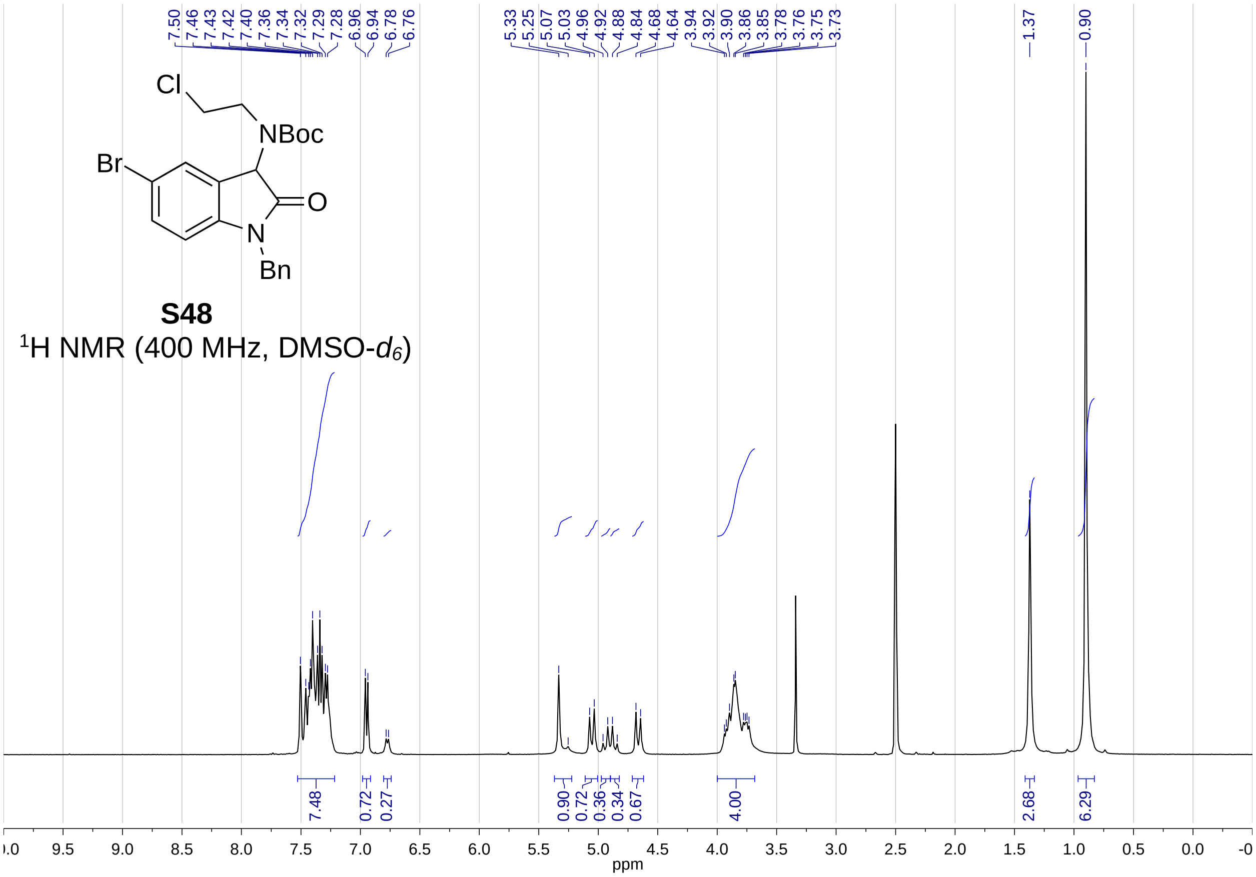

S48

^{1}H NMR (400 MHz, DMSO-d_6)

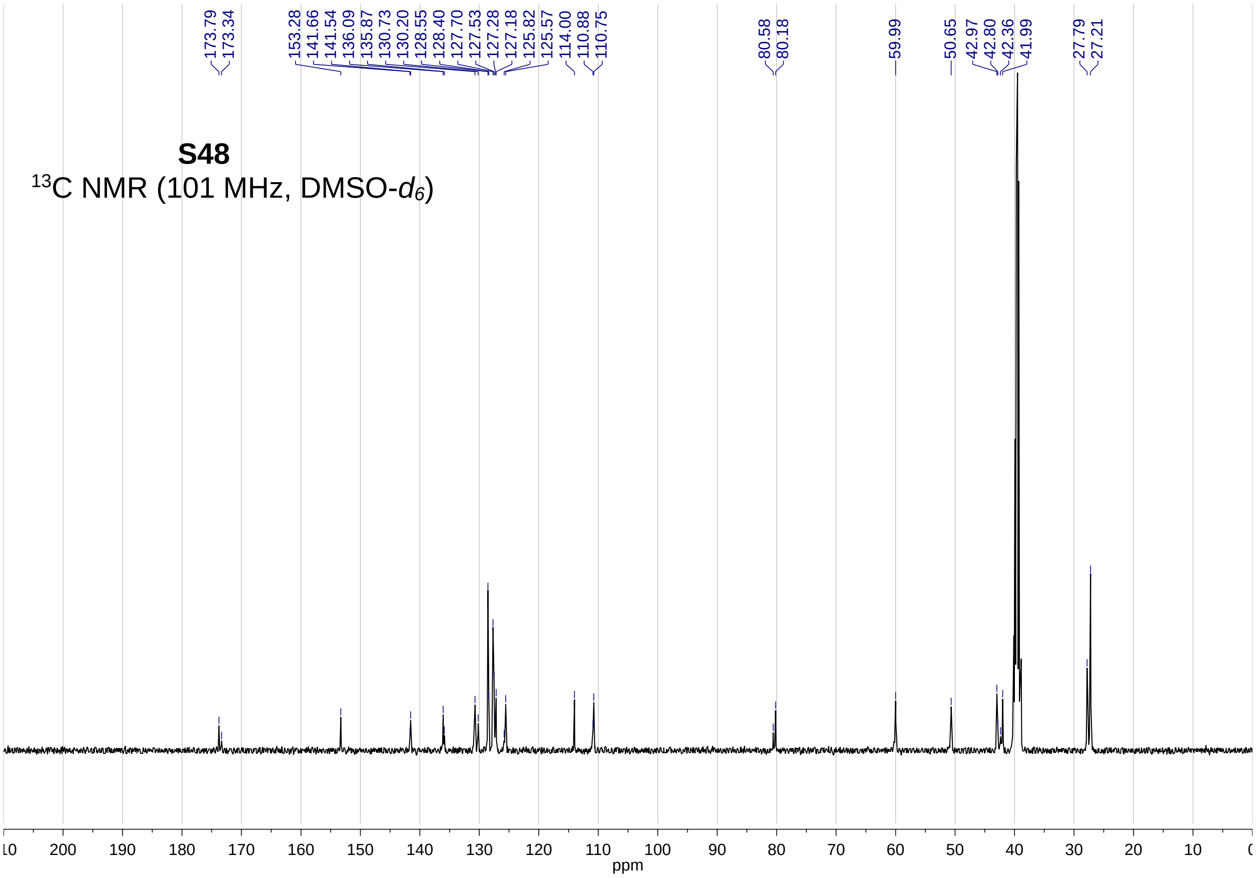

S48

^{13}C NMR (101 MHz, DMSO-d_6)

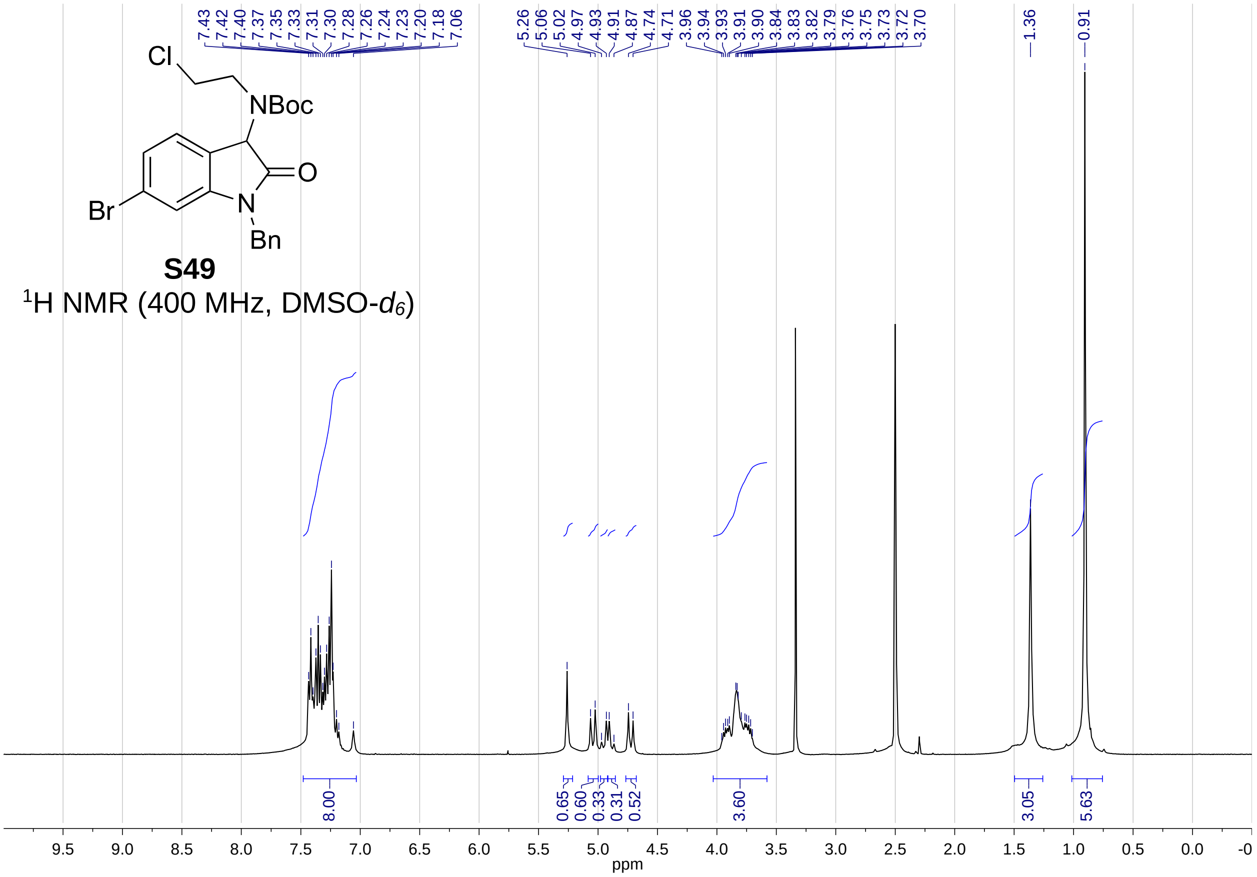

S49

^{1}H NMR (400 MHz, DMSO-d_6)

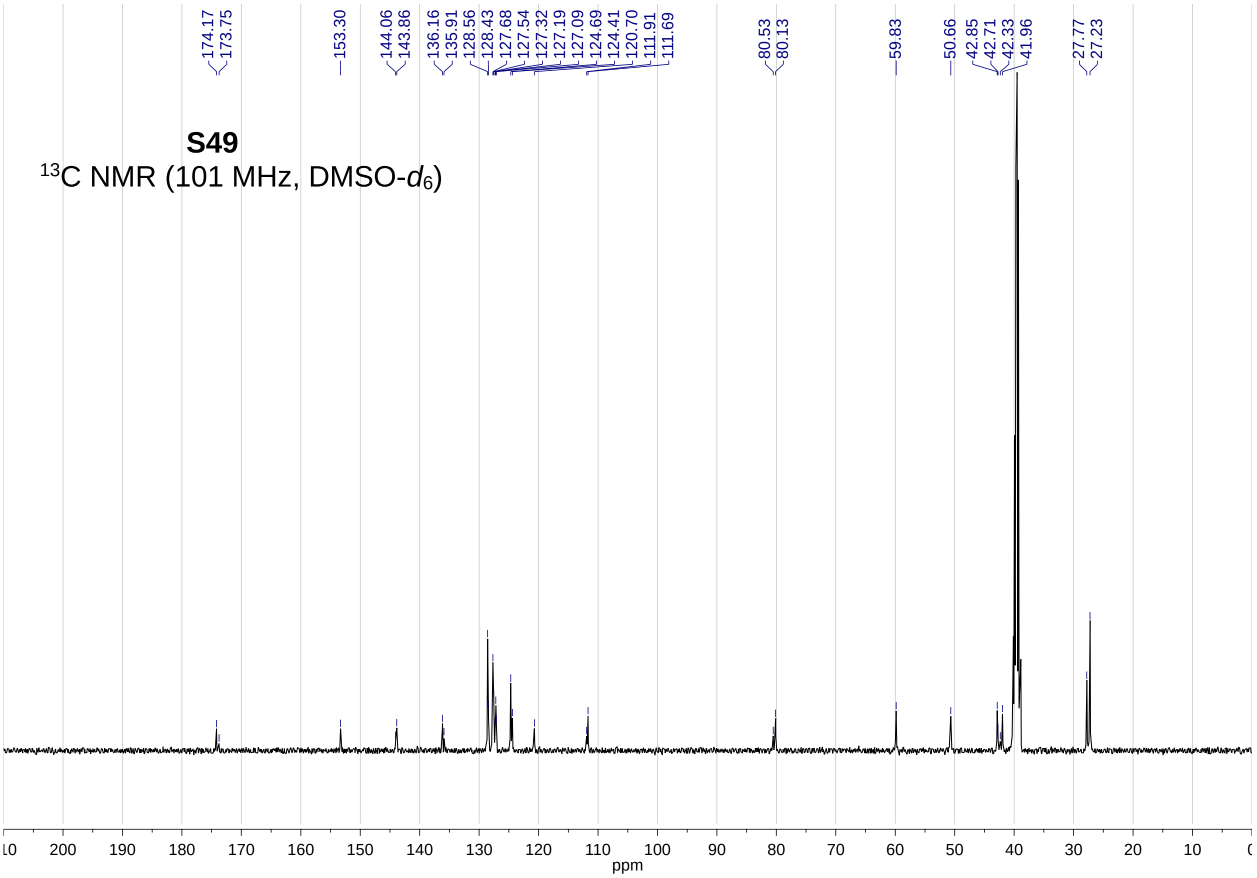

S49

^{13}C NMR (101 MHz, DMSO-d_6)

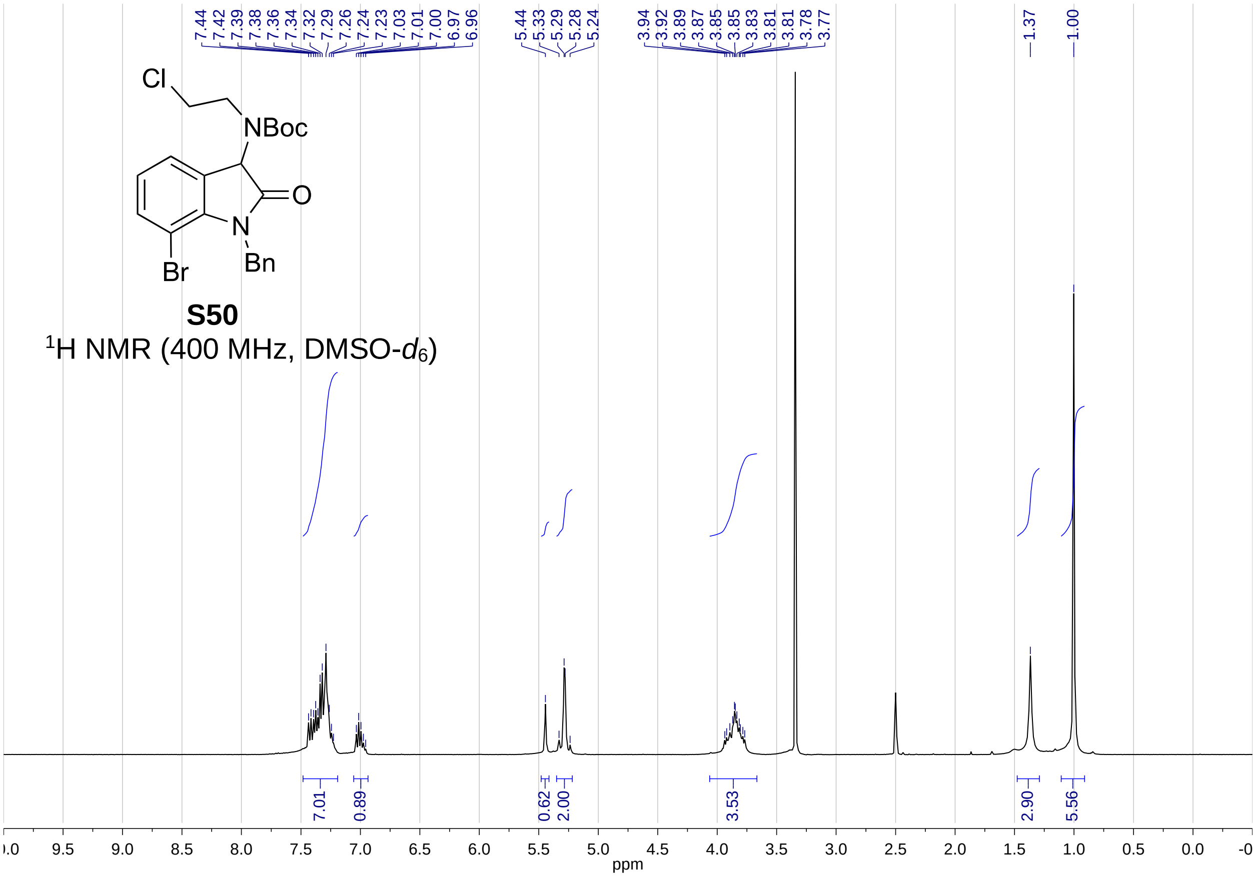

Cl
NBoc
O
N
Bn
Br
S50
^{1}H NMR (400 MHz, DMSO-d_6)
7.44 7.42 7.39 7.38 7.36 7.34 7.32 7.29 7.26 7.24 7.23 7.03 7.01 7.00 6.97 6.96
5.44 5.33 5.29 5.28 5.24
3.94 3.92 3.89 3.87 3.85 3.85 3.83 3.81 3.81 3.78 3.77
1.37
1.00
7.01
0.89
0.62
2.00
3.53
2.90
5.56
ppm

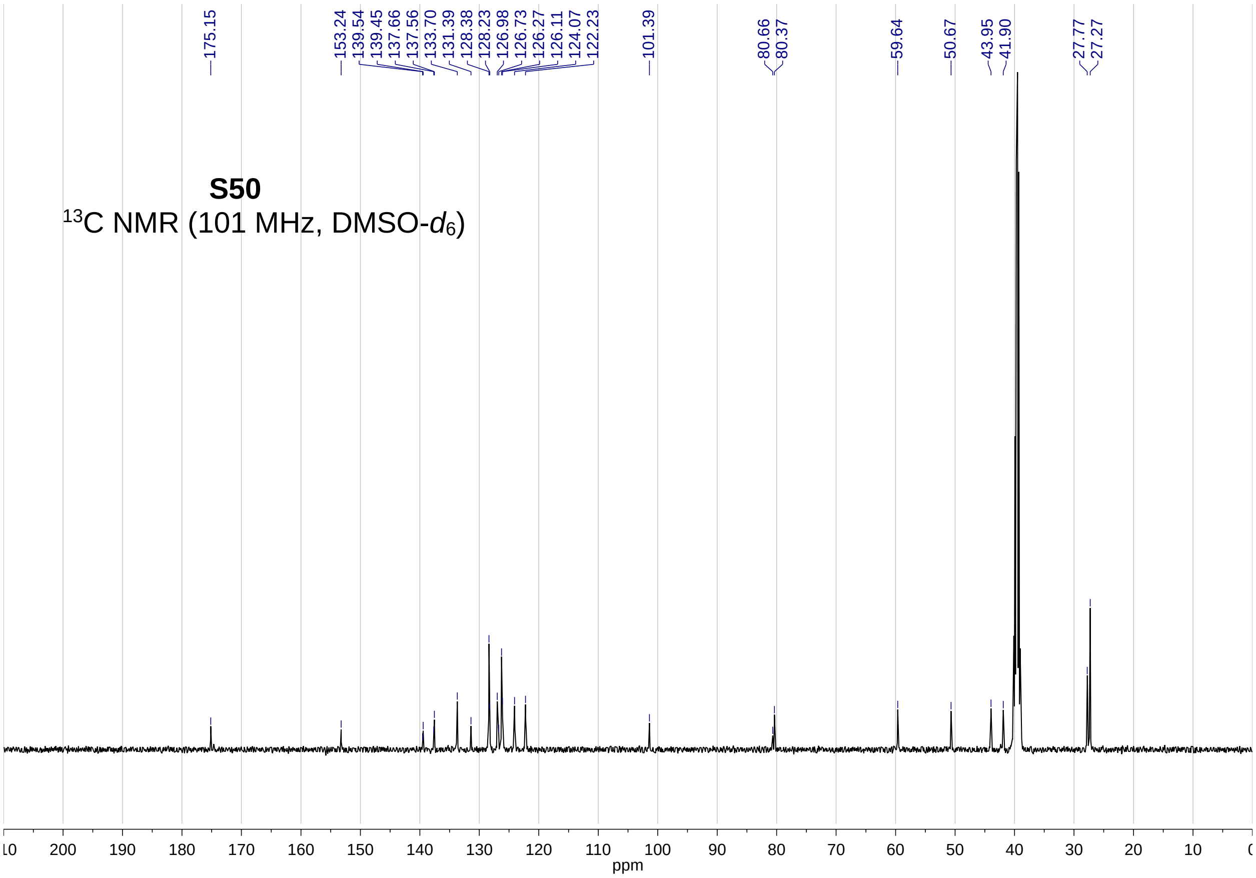

S50
^{13}C NMR (101 MHz, DMSO-d_6)
175.15
153.24 139.54 139.45 137.66 137.56 133.70 131.39 128.38 128.23 126.98 126.73 126.27 126.11 124.07 122.23
101.39
80.66 80.37
59.64
50.67
43.95 41.90
27.77 27.27
ppm

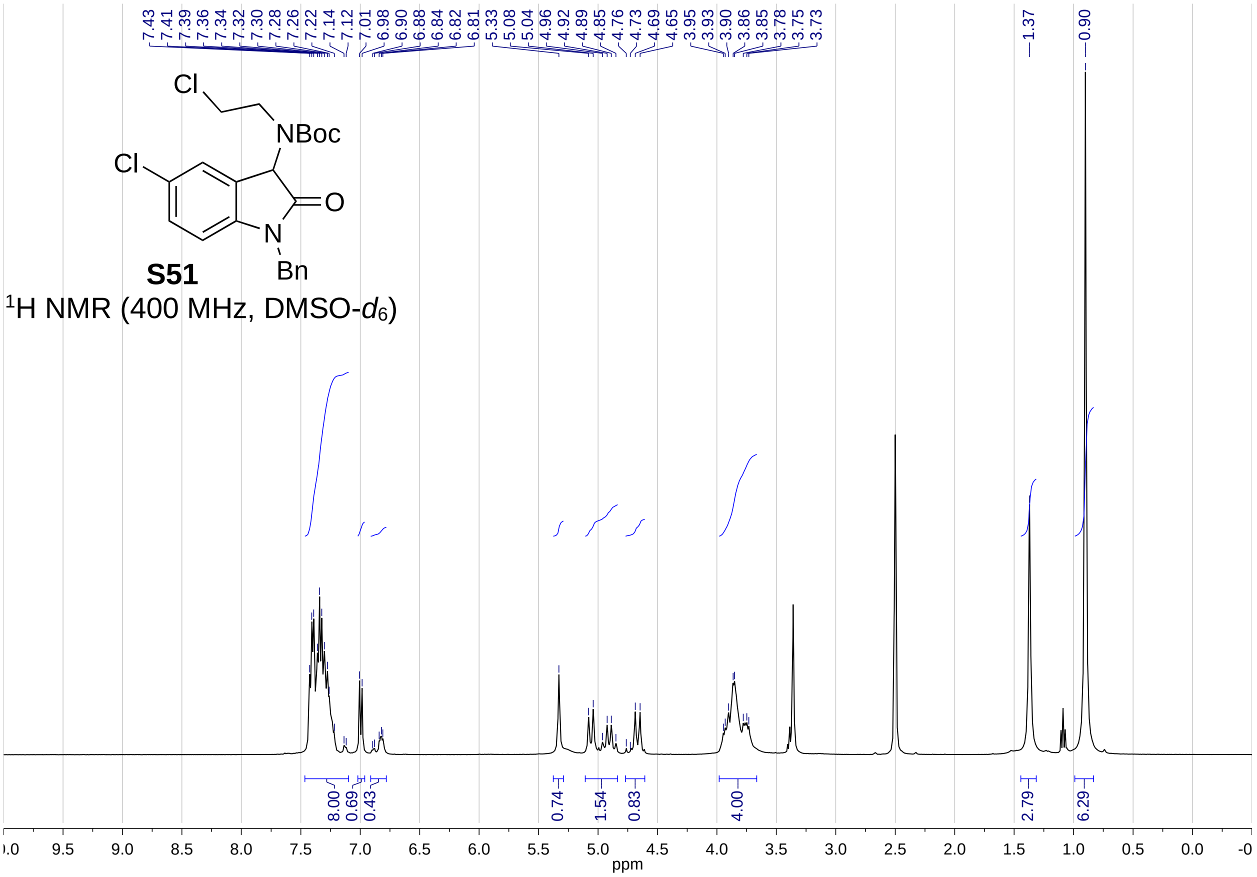
Cl
NBoc
Cl
O
N
Bn
S51
1H NMR (400 MHz, DMSO-d6)
7.43 7.41 7.39 7.36 7.34 7.32 7.30 7.28 7.26 7.22 7.14 7.12 7.01 6.98 6.90 6.88 6.84 6.82 6.81 5.33 5.08 5.04 4.96 4.92 4.89 4.85 4.76 4.73 4.69 4.65 3.95 3.93 3.90 3.86 3.85 3.78 3.75 3.73
1.37
0.90
8.00 0.69 0.43 0.74 1.54 0.83 4.00 2.79 6.29
ppm

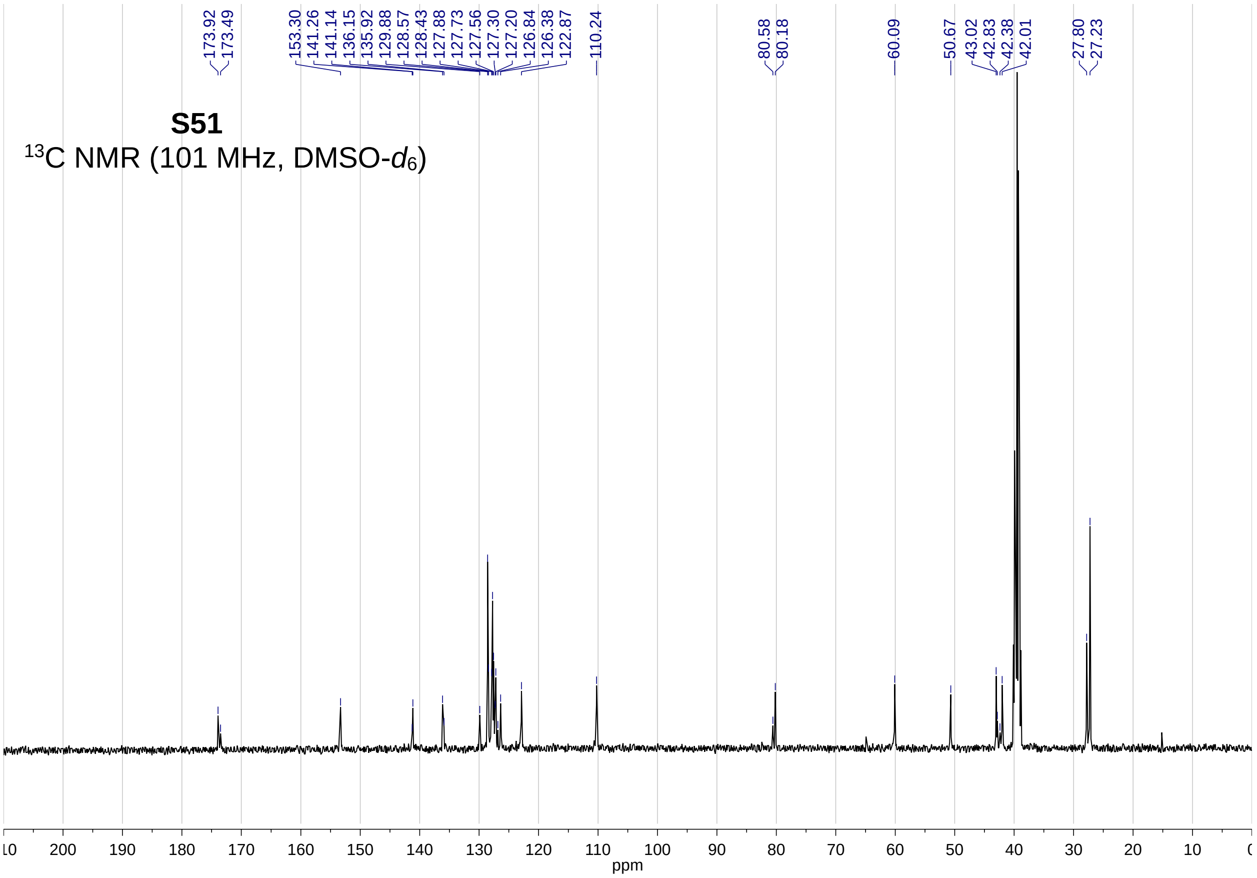
S51
13C NMR (101 MHz, DMSO-d6)
173.92 173.49
153.30 141.26 141.14 136.15 135.92 129.88 128.57 128.43 127.88 127.73 127.56 127.30 127.20 126.84 126.38 122.87
110.24
80.58 80.18
60.09
50.67 43.02 42.83 42.38 42.01
27.80 27.23
ppm

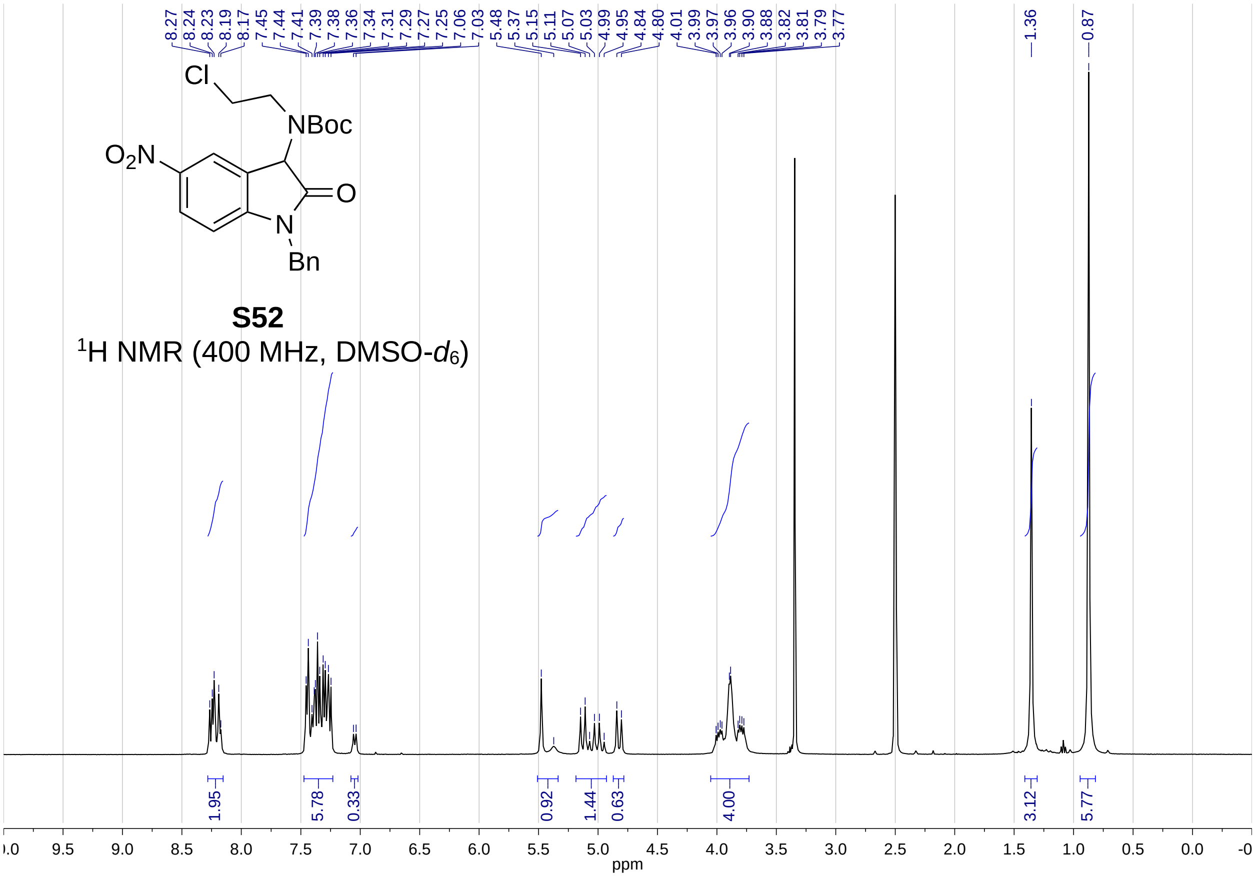

S52

^{1}H NMR (400 MHz, DMSO-d_6)

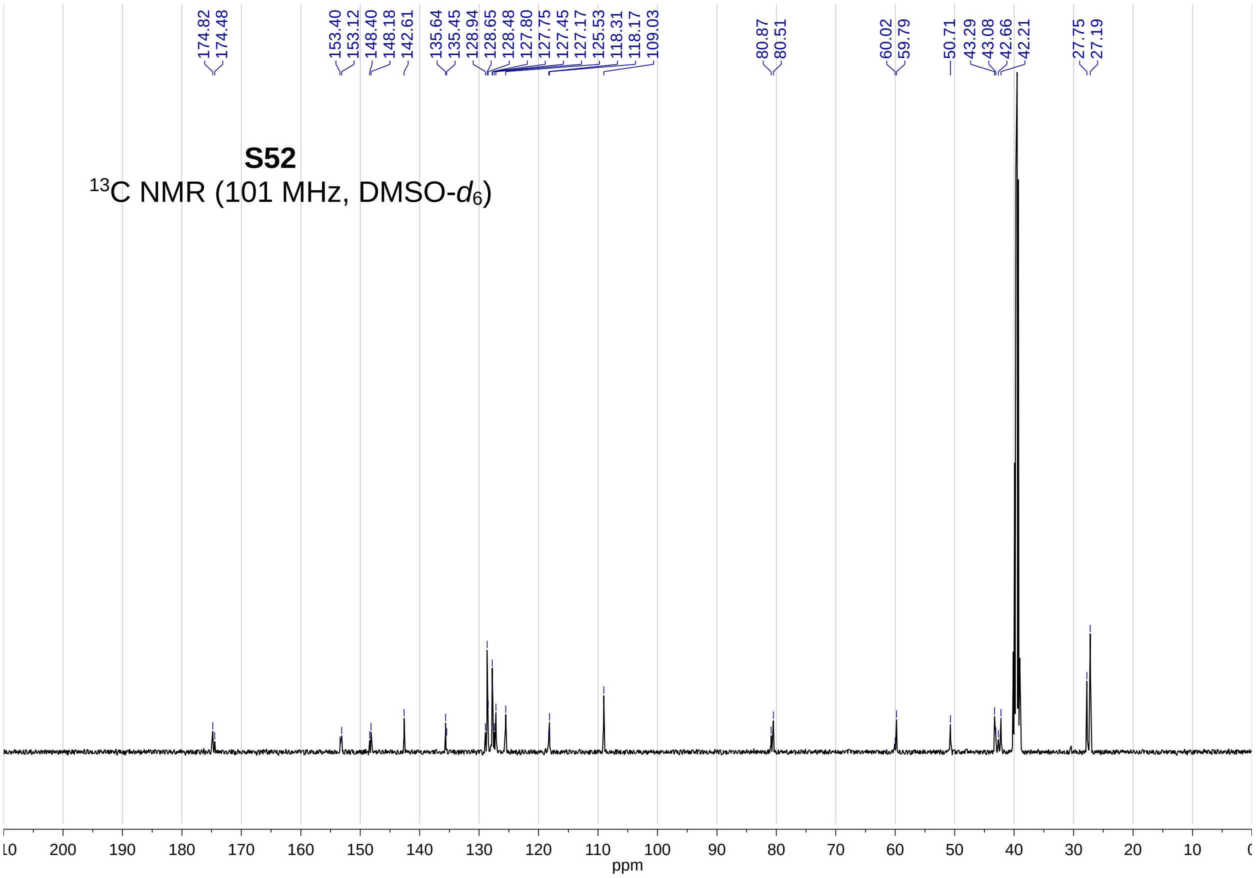

S52

^{13}C NMR (101 MHz, DMSO-d_6)

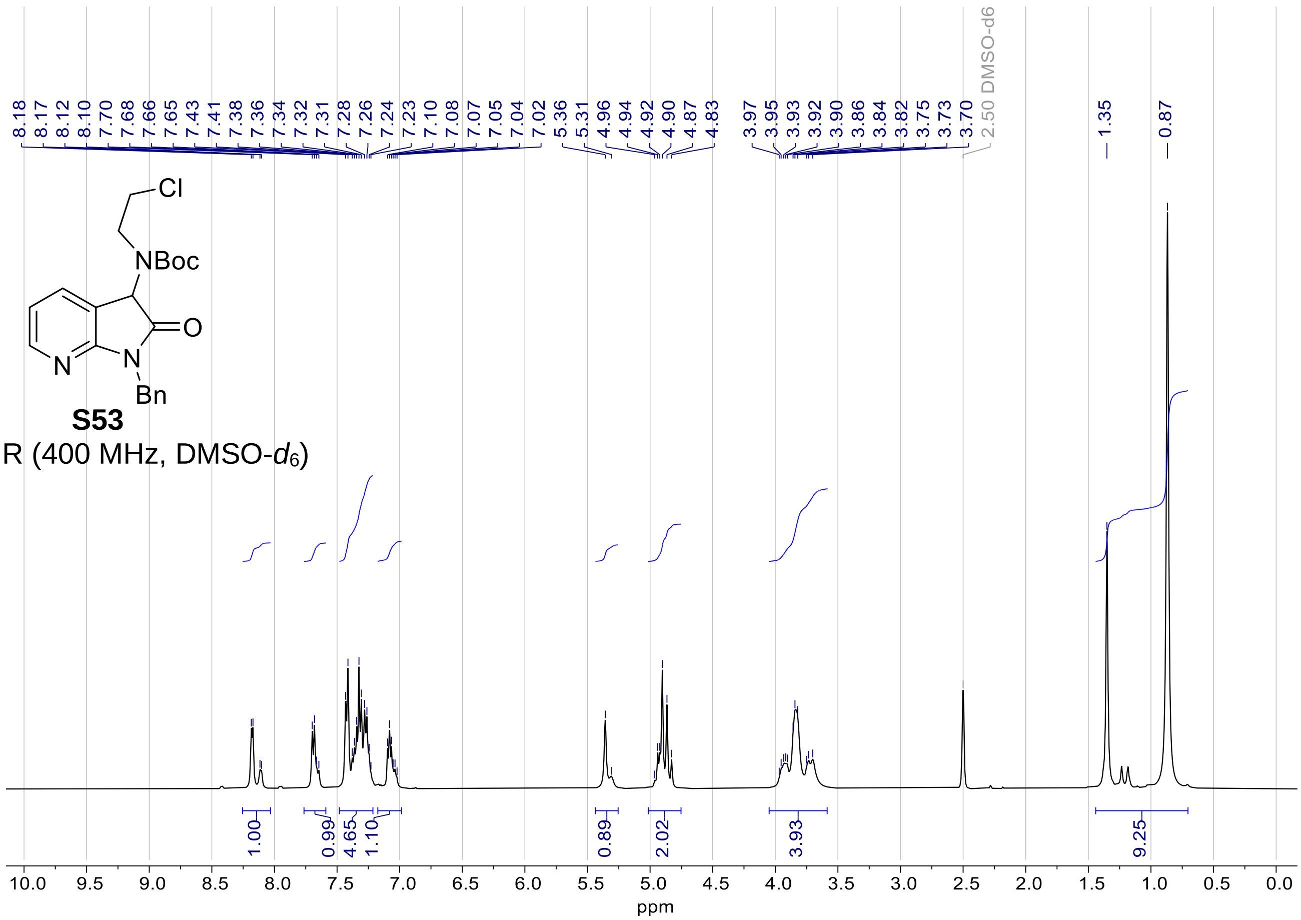

S53

^{1}H NMR (400 MHz, DMSO-d_6)

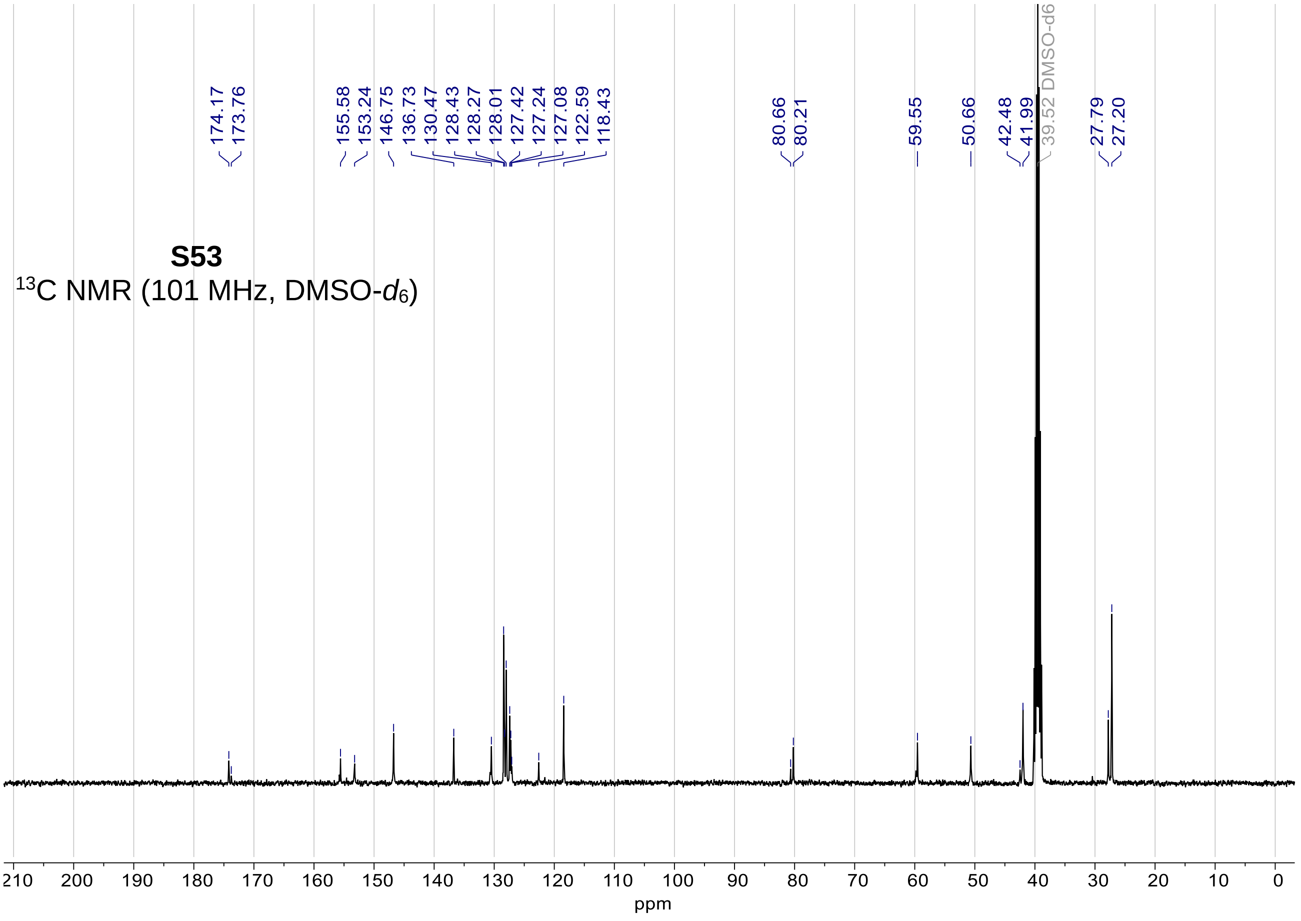

S53

^{13}C NMR (101 MHz, DMSO-d_6)

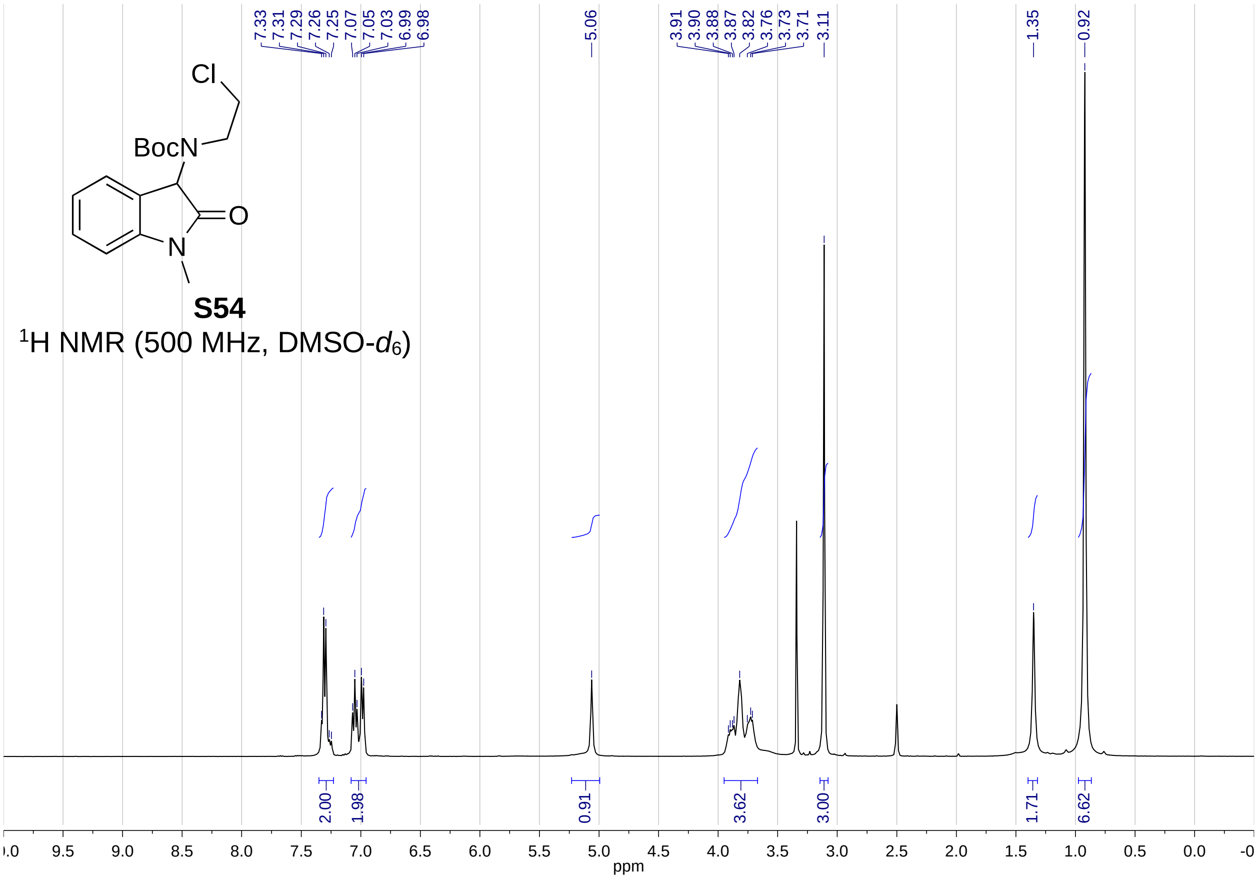

S54

^{1}H NMR (500 MHz, DMSO-d_6)

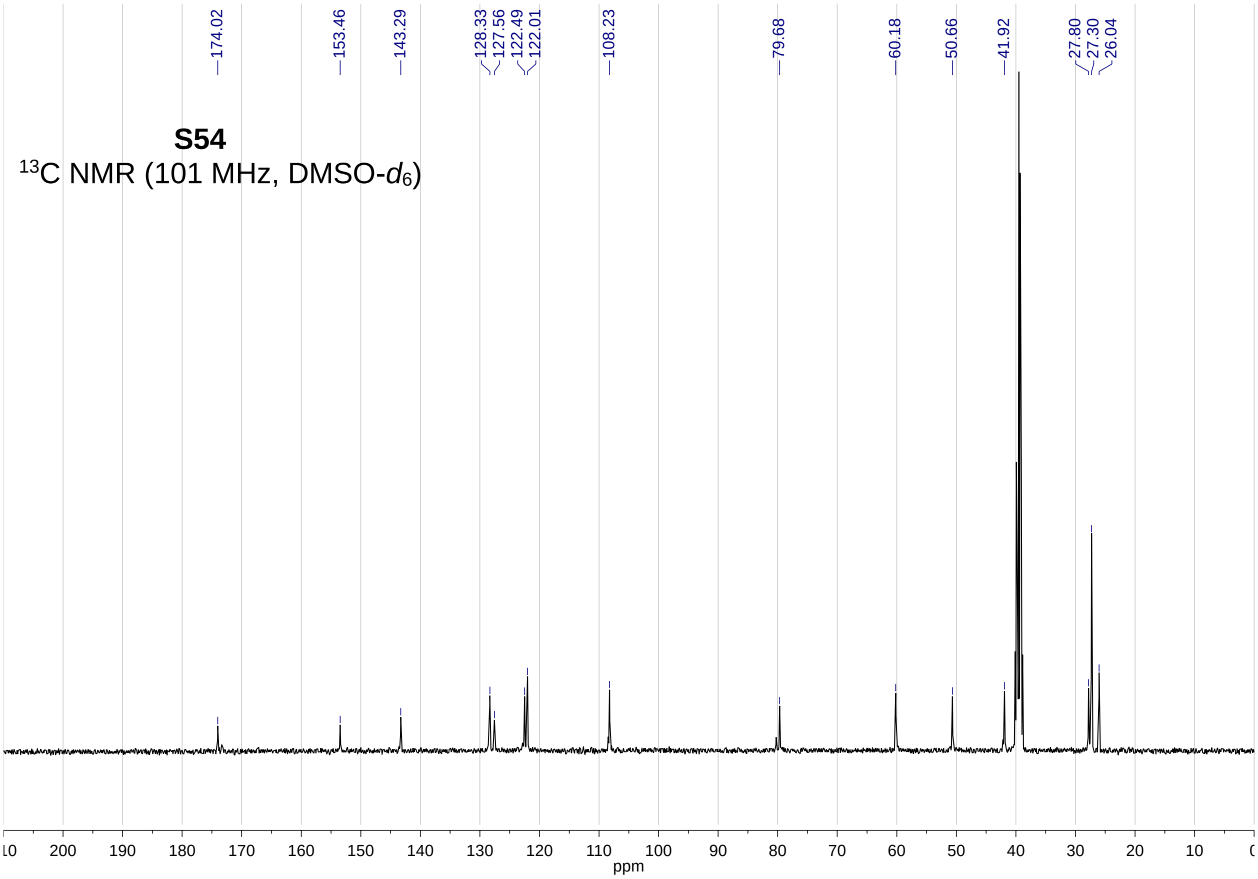

S54

^{13}C NMR (101 MHz, DMSO-d_6)

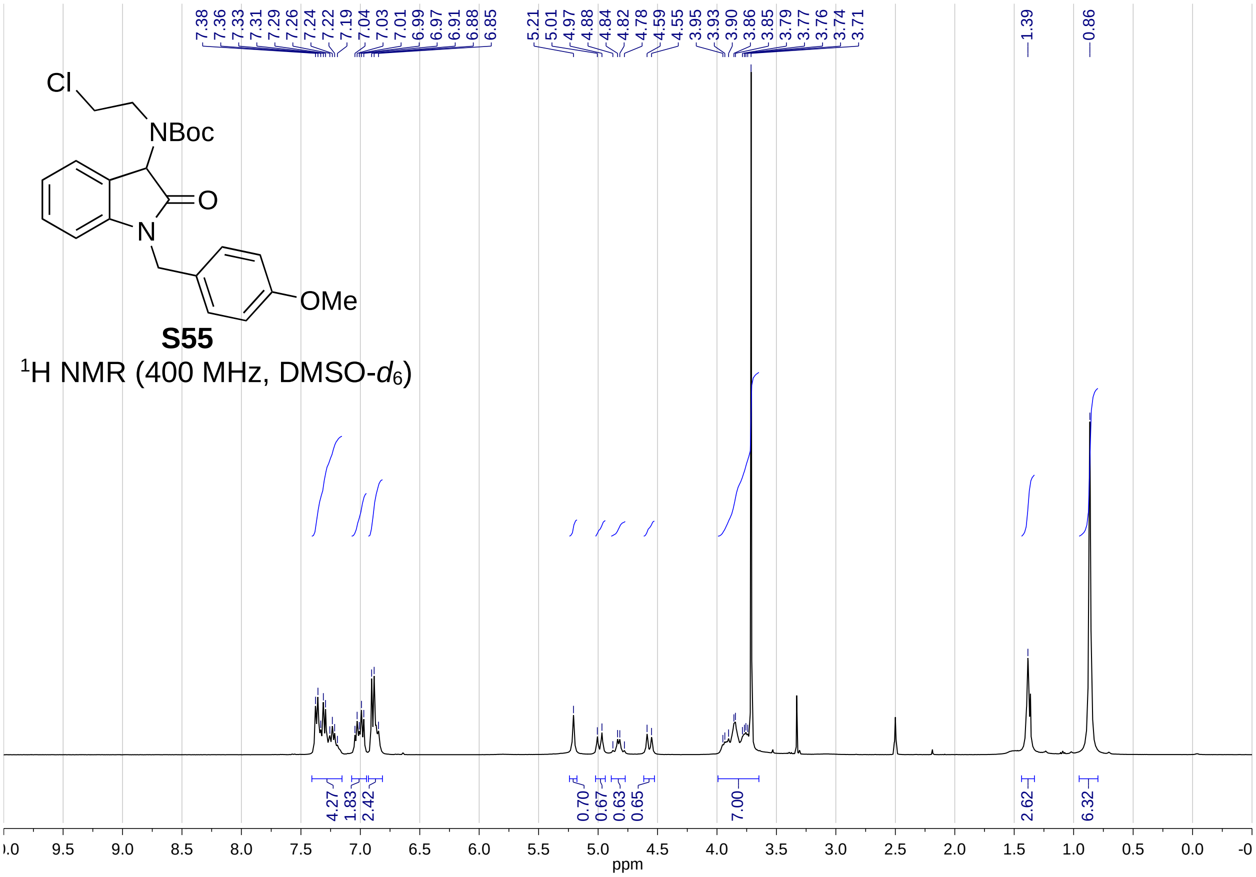

S55

^{1}H NMR (400 MHz, DMSO-d_6)

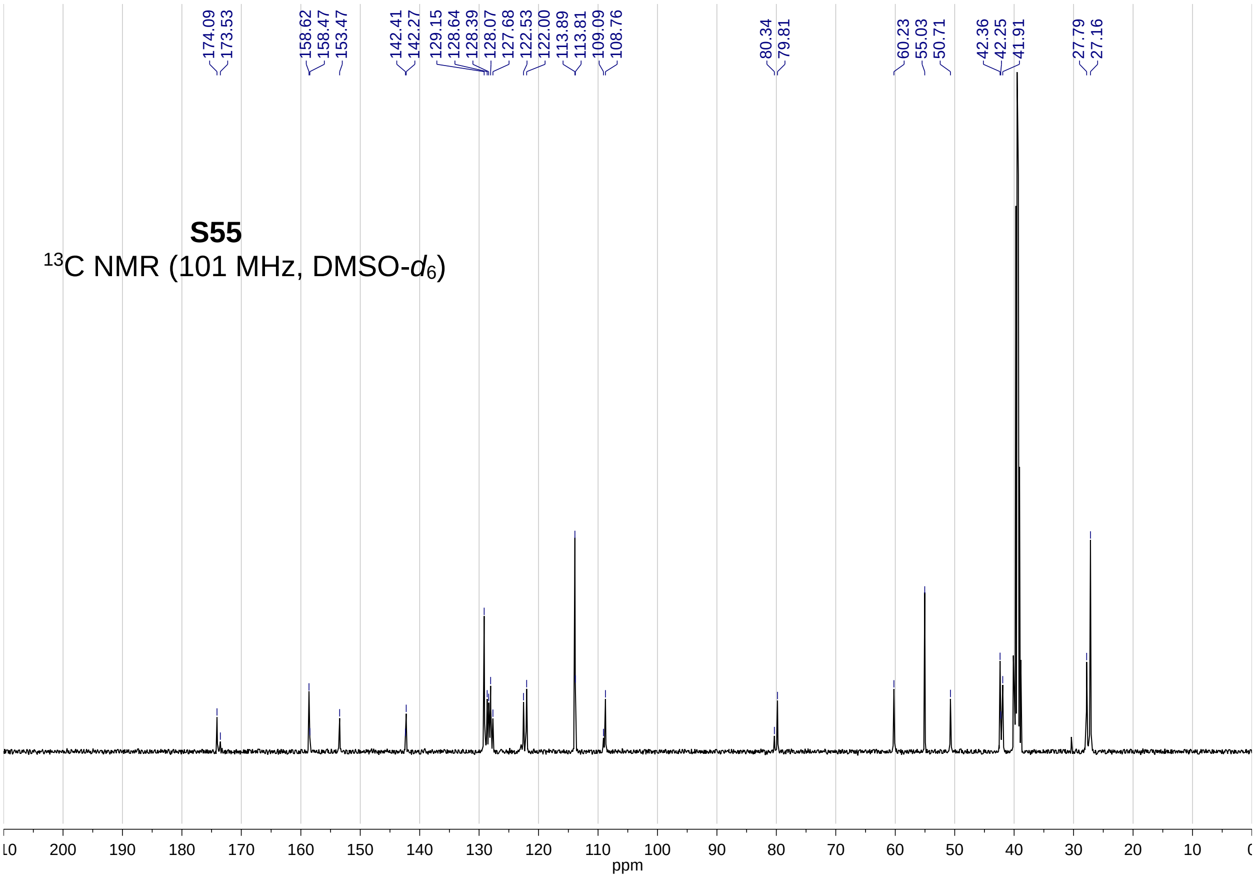

S55

^{13}C NMR (101 MHz, DMSO-d_6)

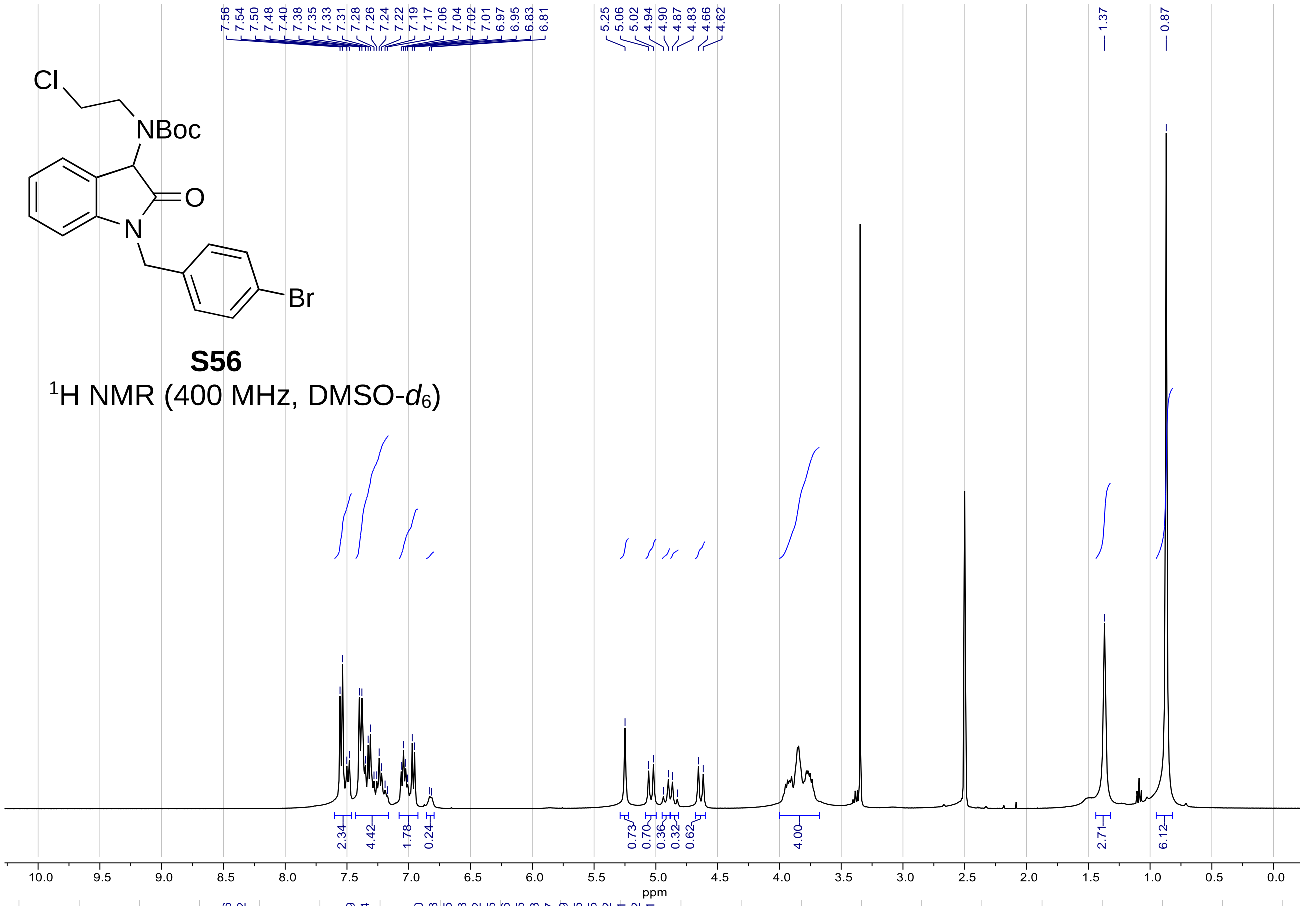

S56

^{1}H NMR (400 MHz, DMSO-d_6)

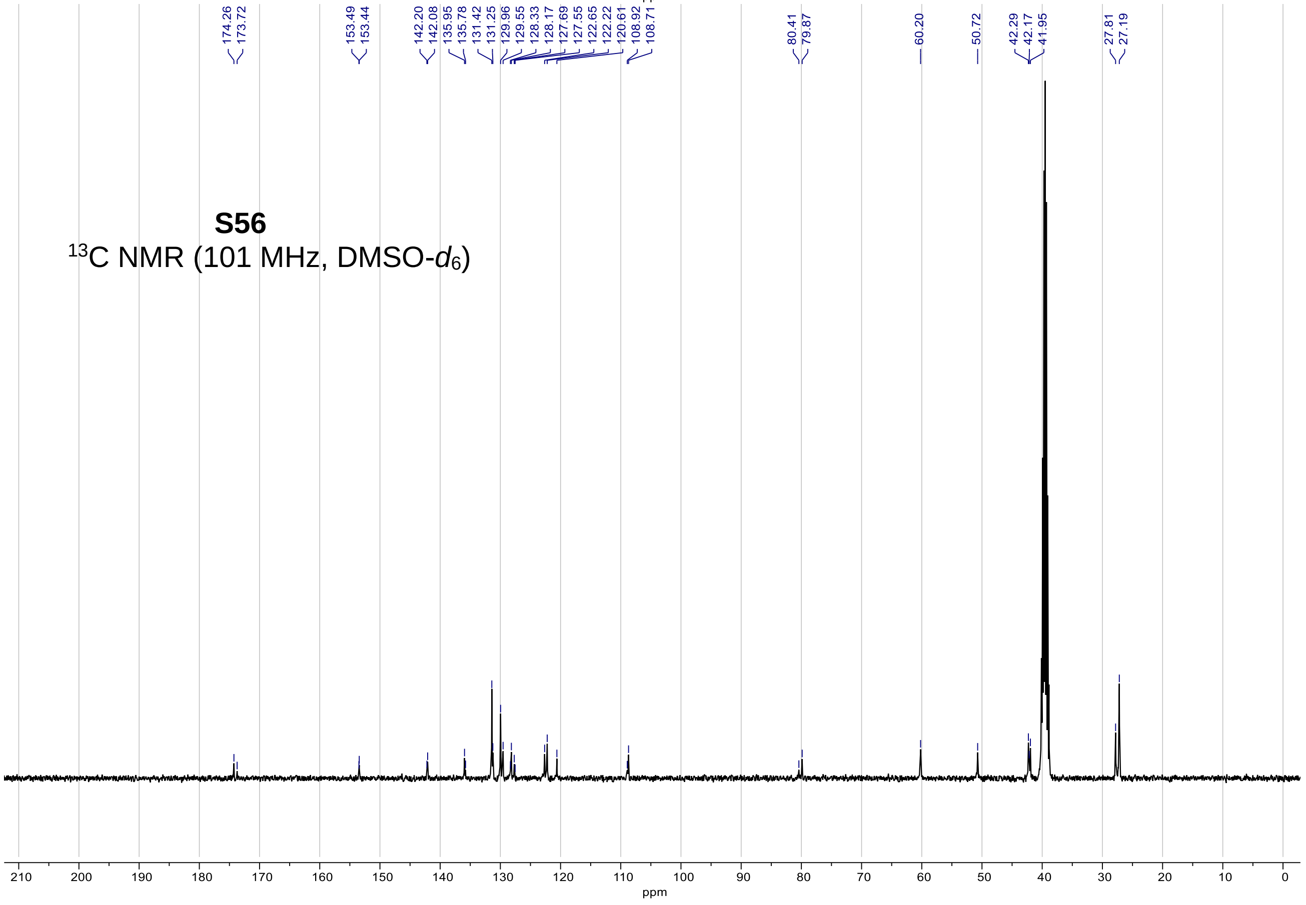

S56

^{13}C NMR (101 MHz, DMSO-d_6)

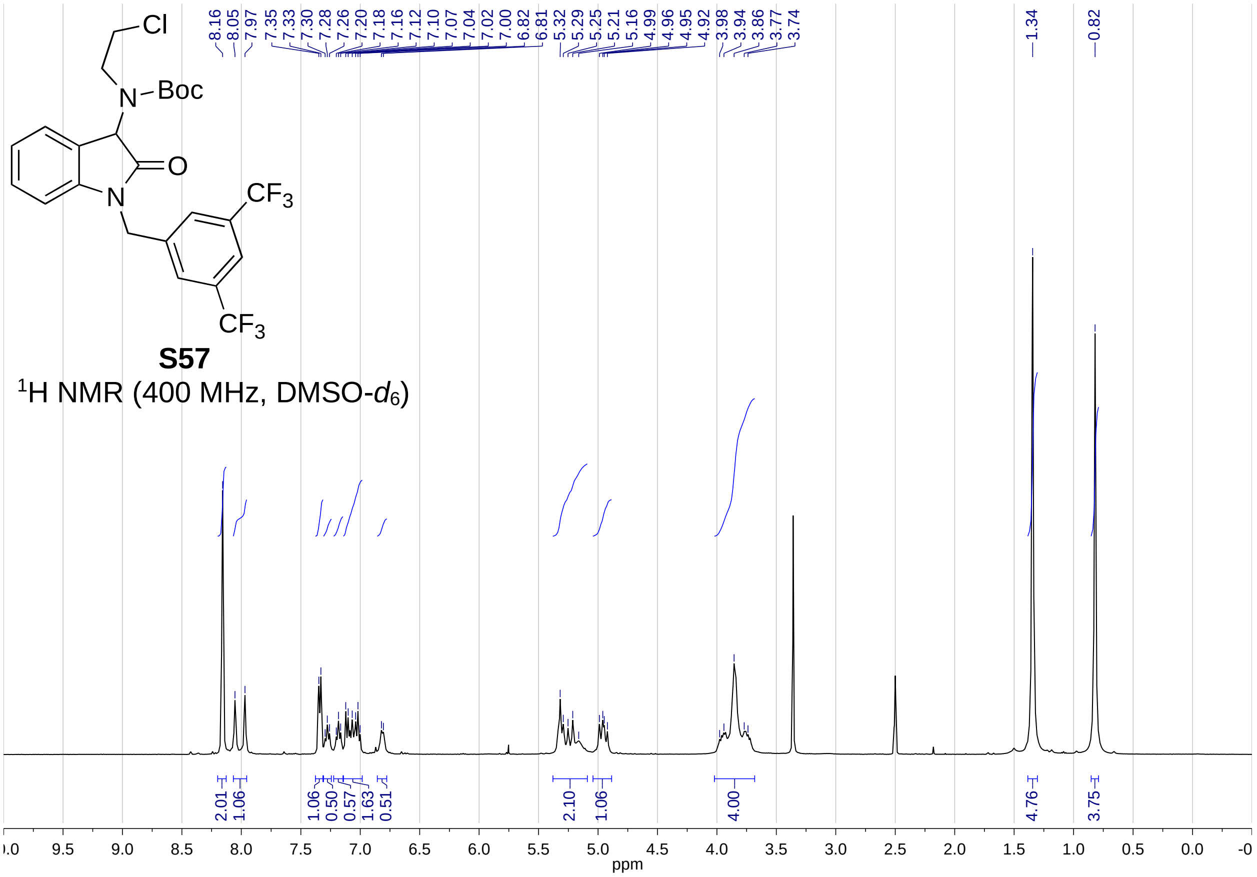

Cl
N
Boc
O
N
CF3
CF3
S57
1H NMR (400 MHz, DMSO-d6)
ppm

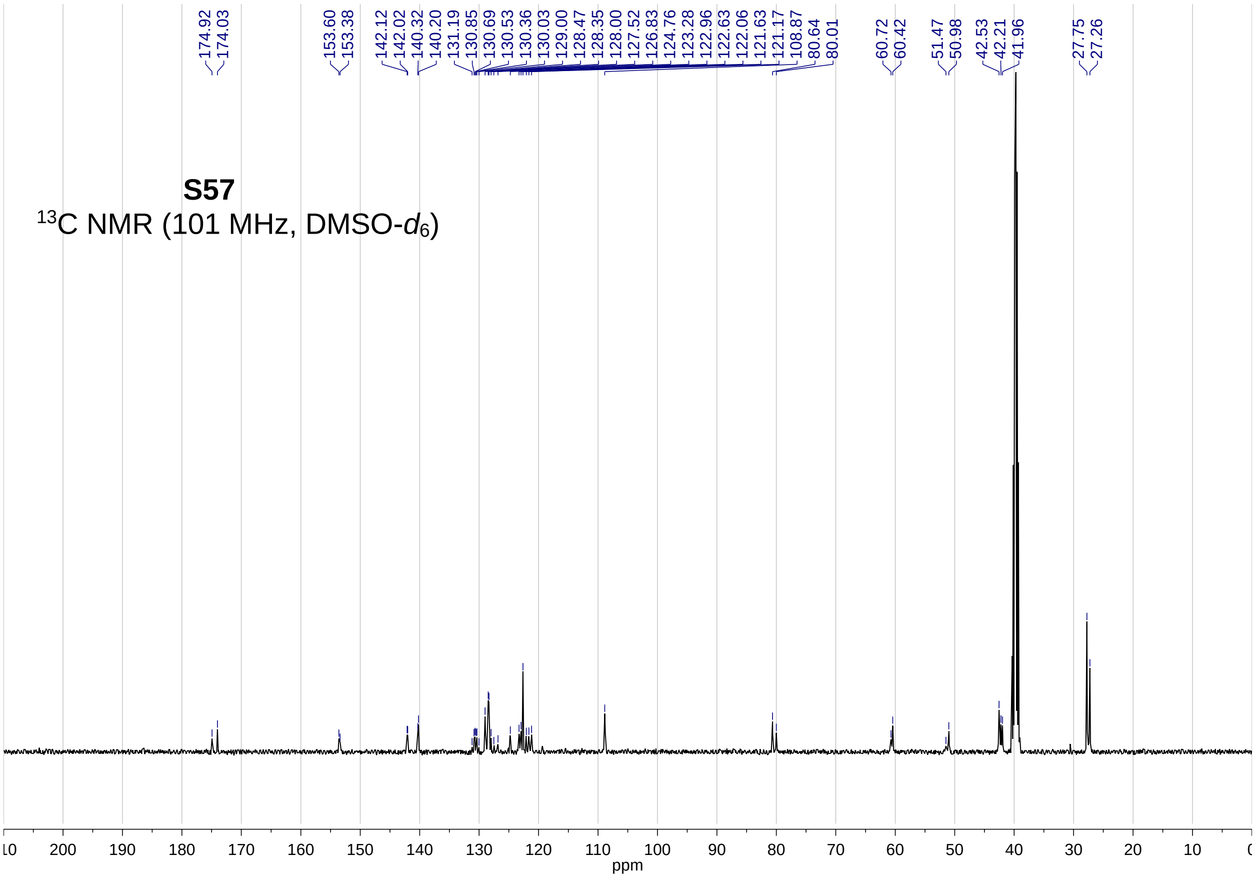

S57
13C NMR (101 MHz, DMSO-d6)
ppm

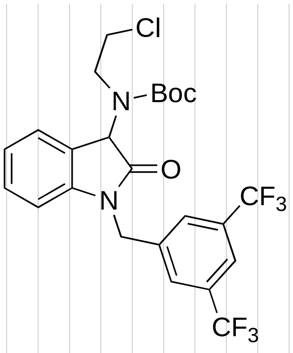

S57

^{19}F NMR (377 MHz, DMSO-d_6)

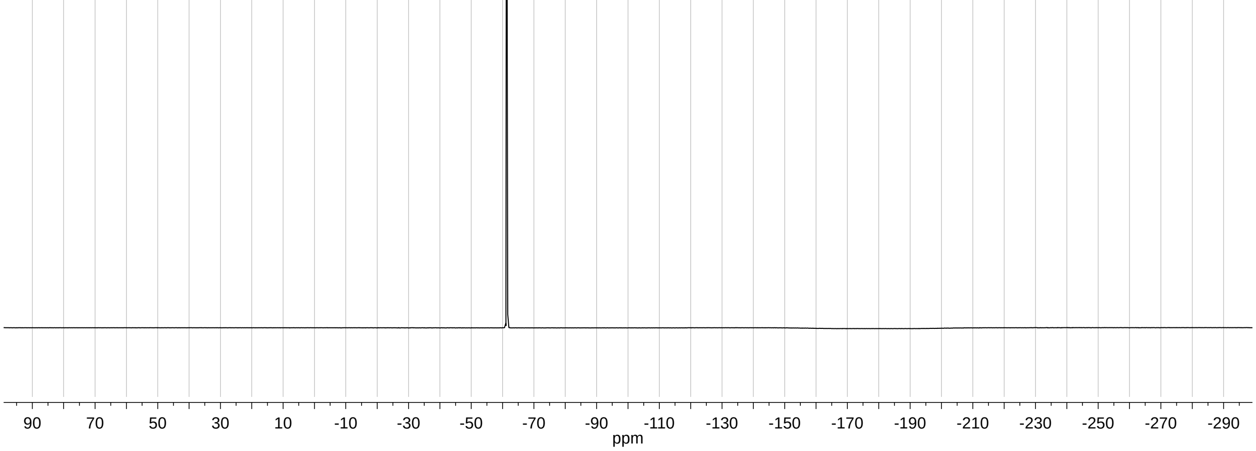

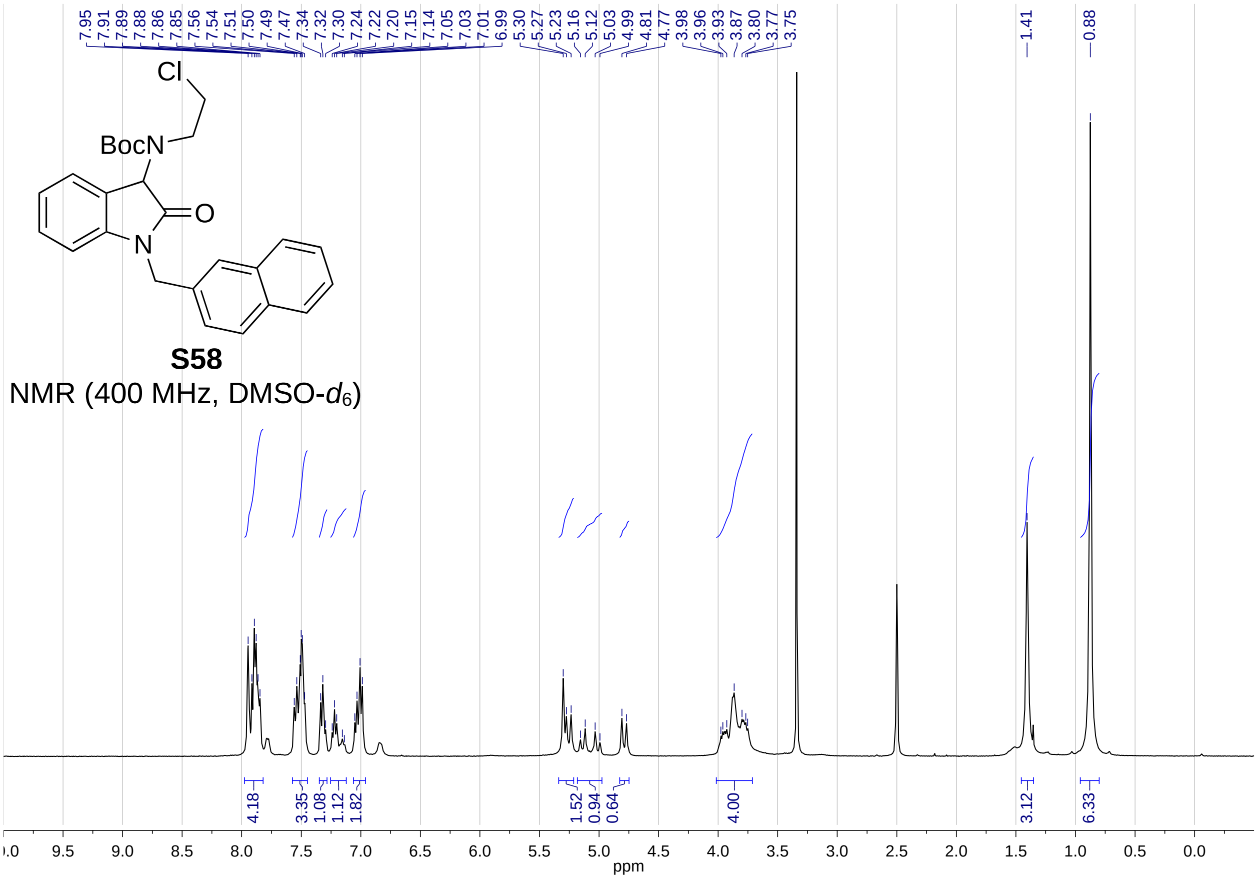

S58

^{1}H NMR (400 MHz, DMSO-d_6)

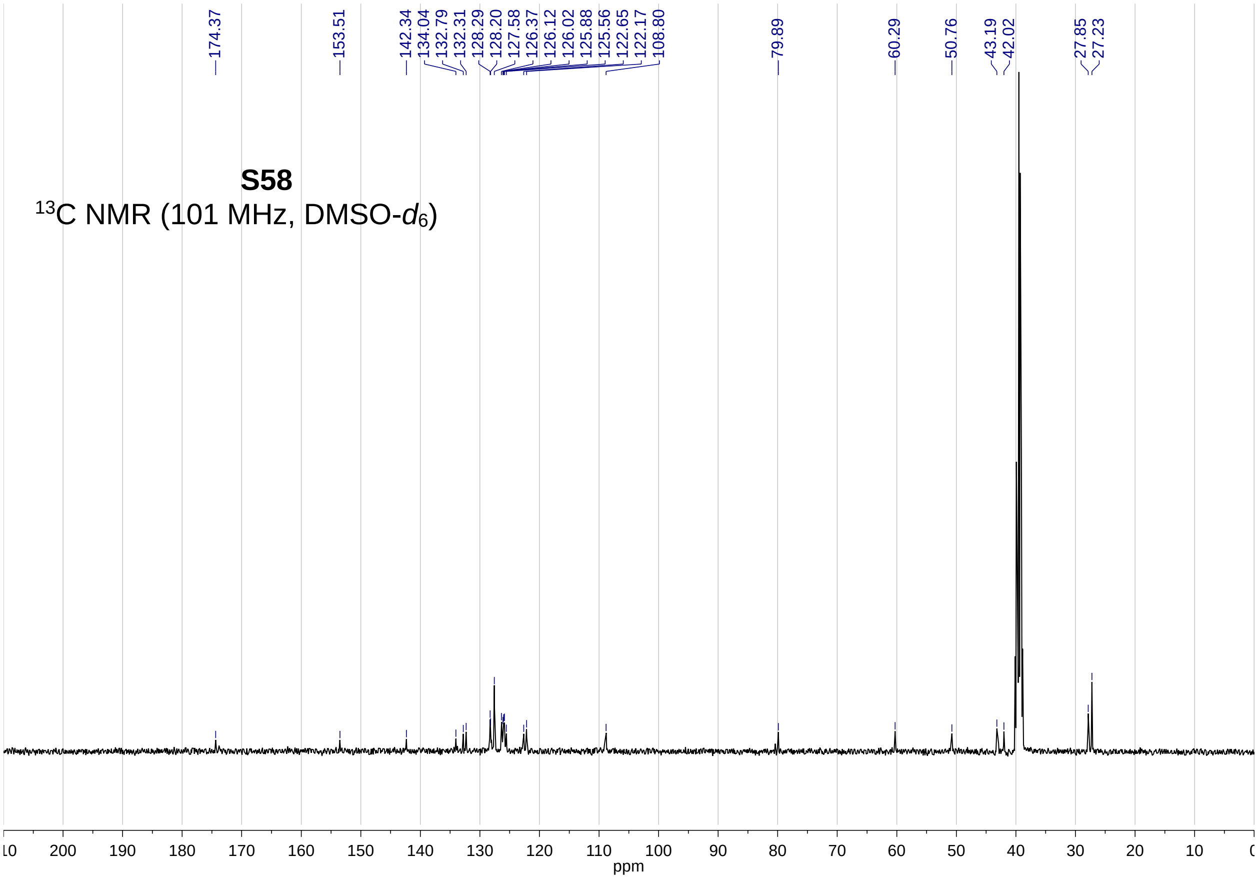

S58

^{13}C NMR (101 MHz, DMSO-d_6)

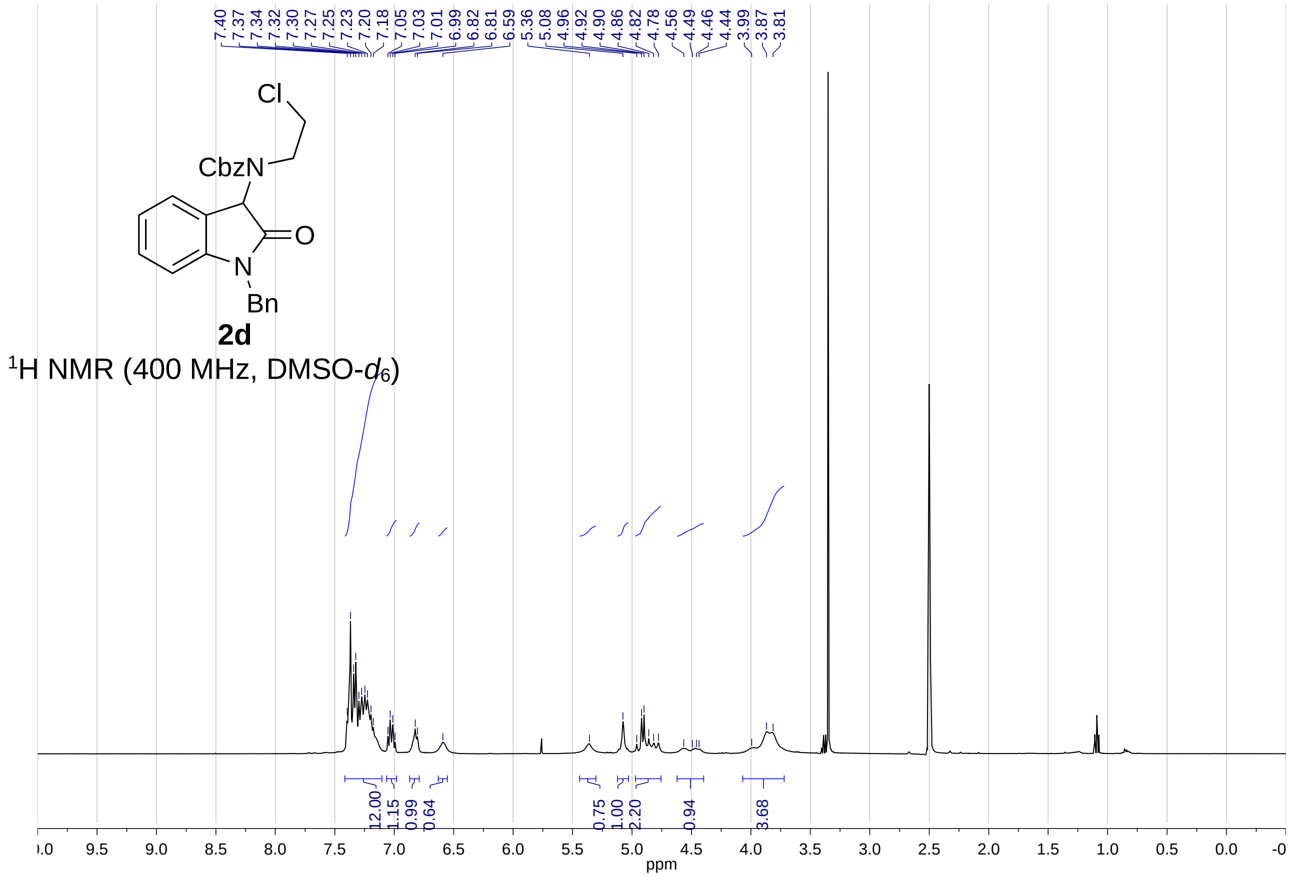

2d

^{1}H NMR (400 MHz, DMSO-d_6)

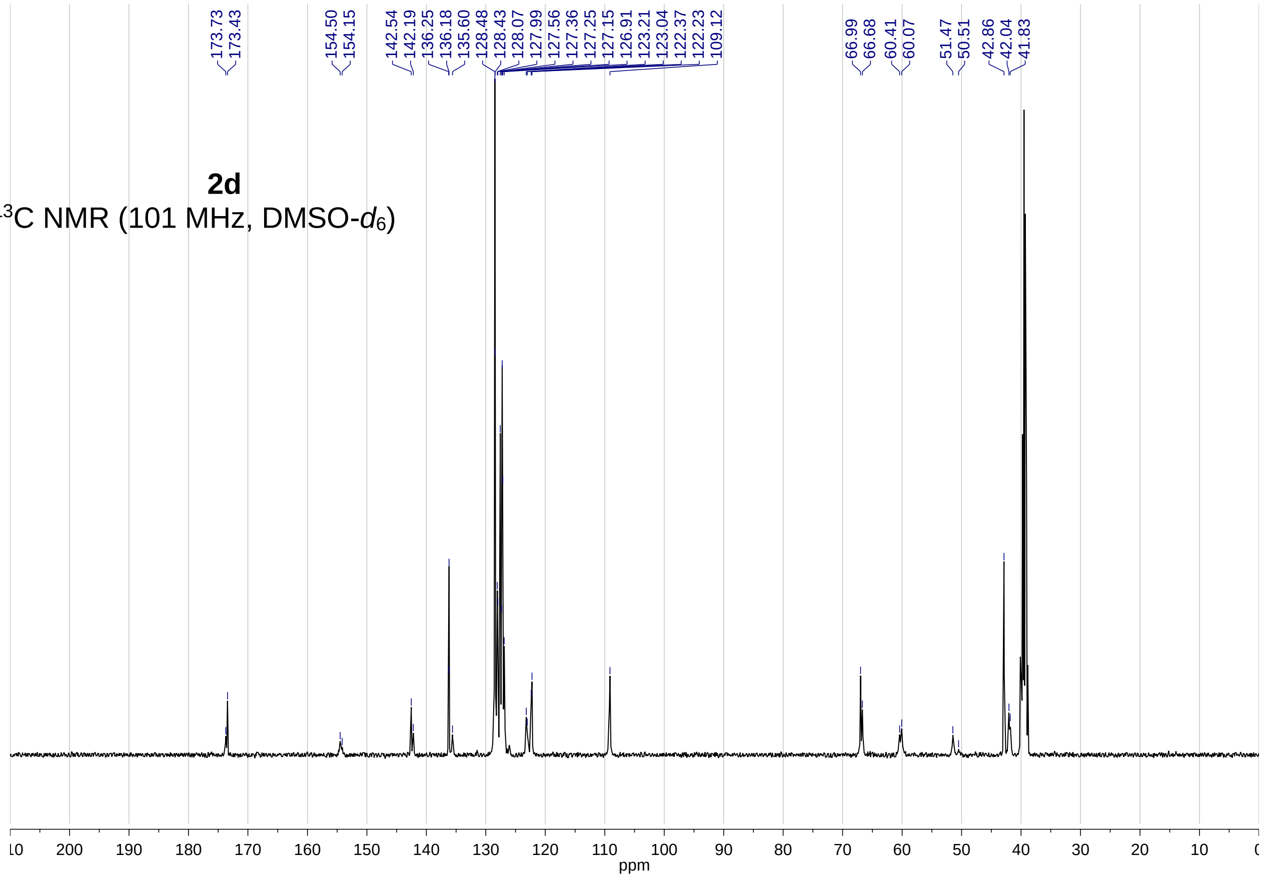

2d

^{13}C NMR (101 MHz, DMSO-d_6)

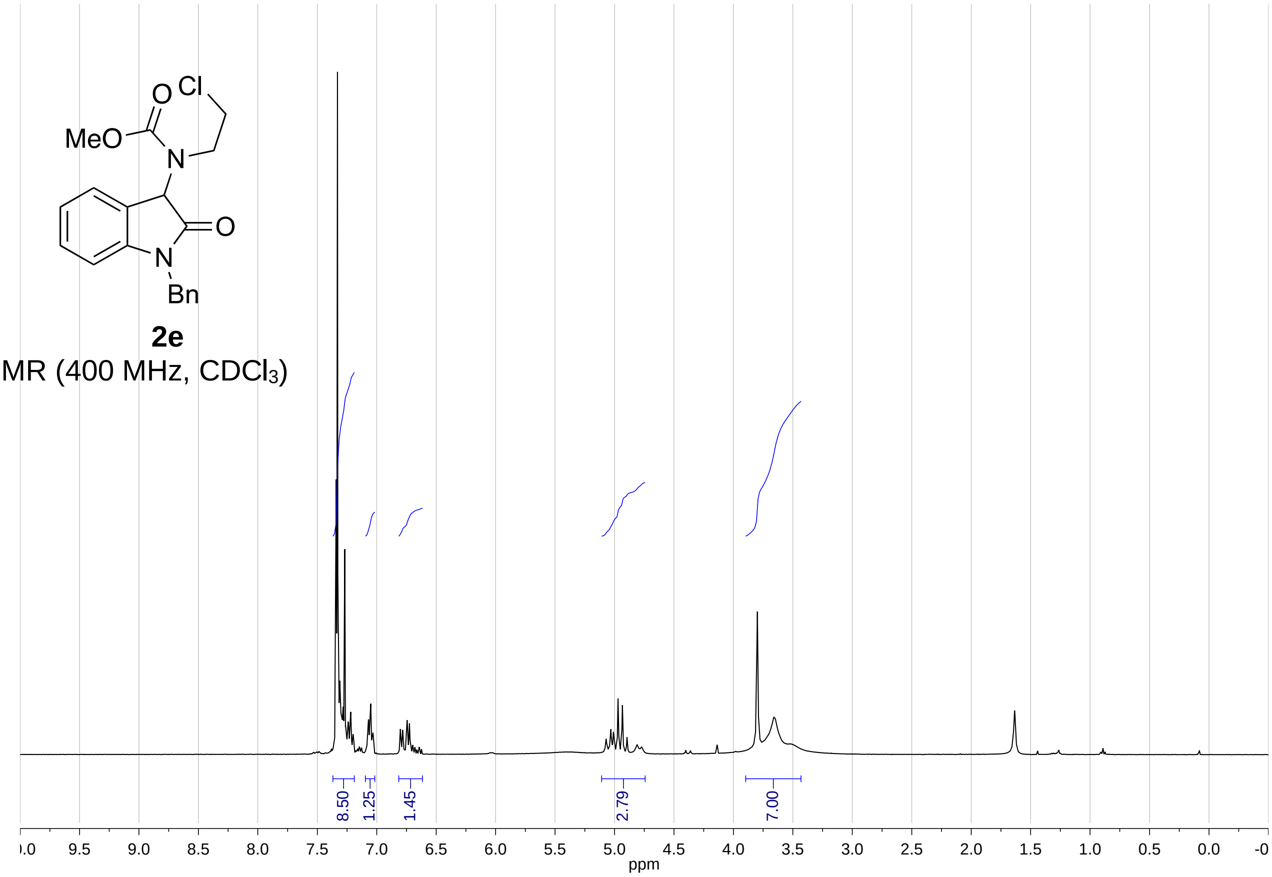

2e

^{1}H NMR (400 MHz, $CDCl_3$)

2e

^{13}C NMR (101 MHz, $CDCl_3$)

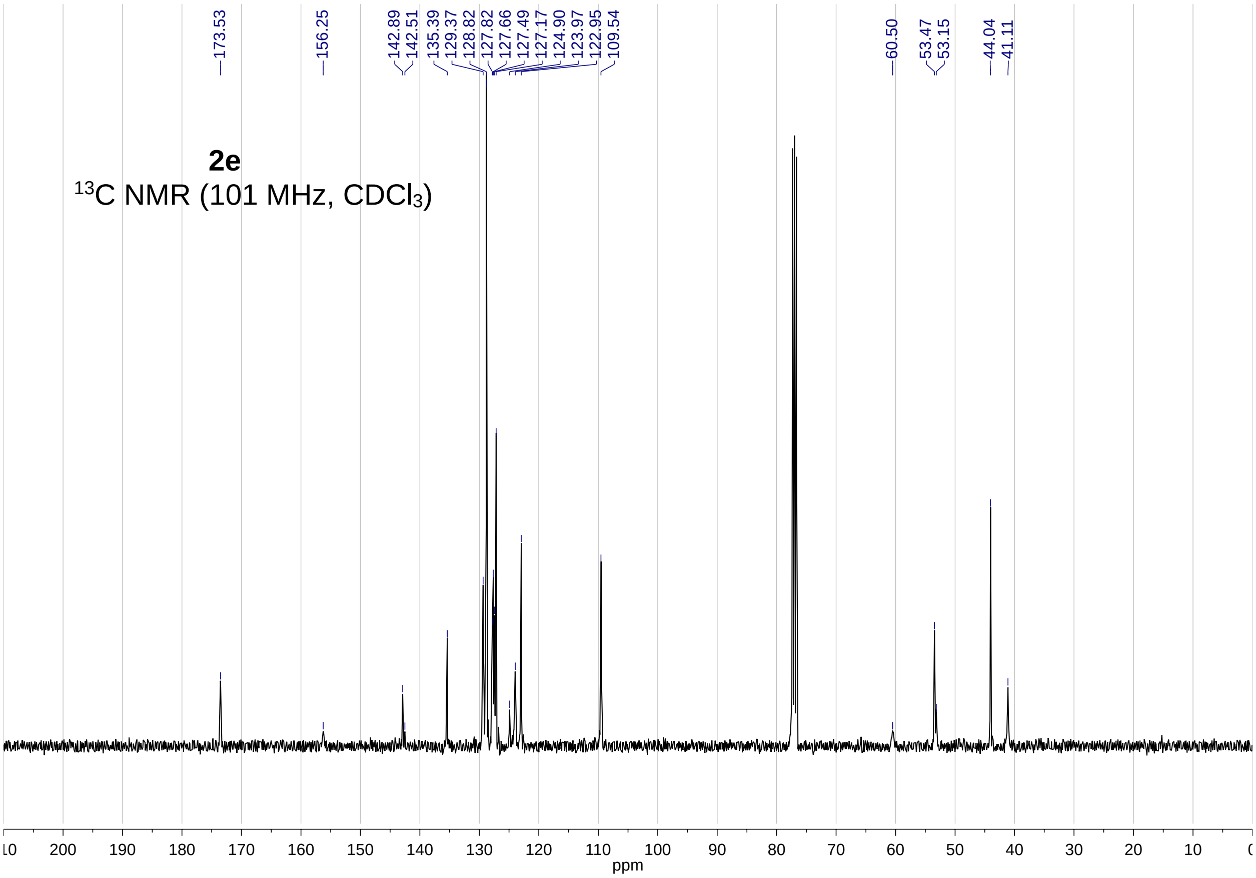

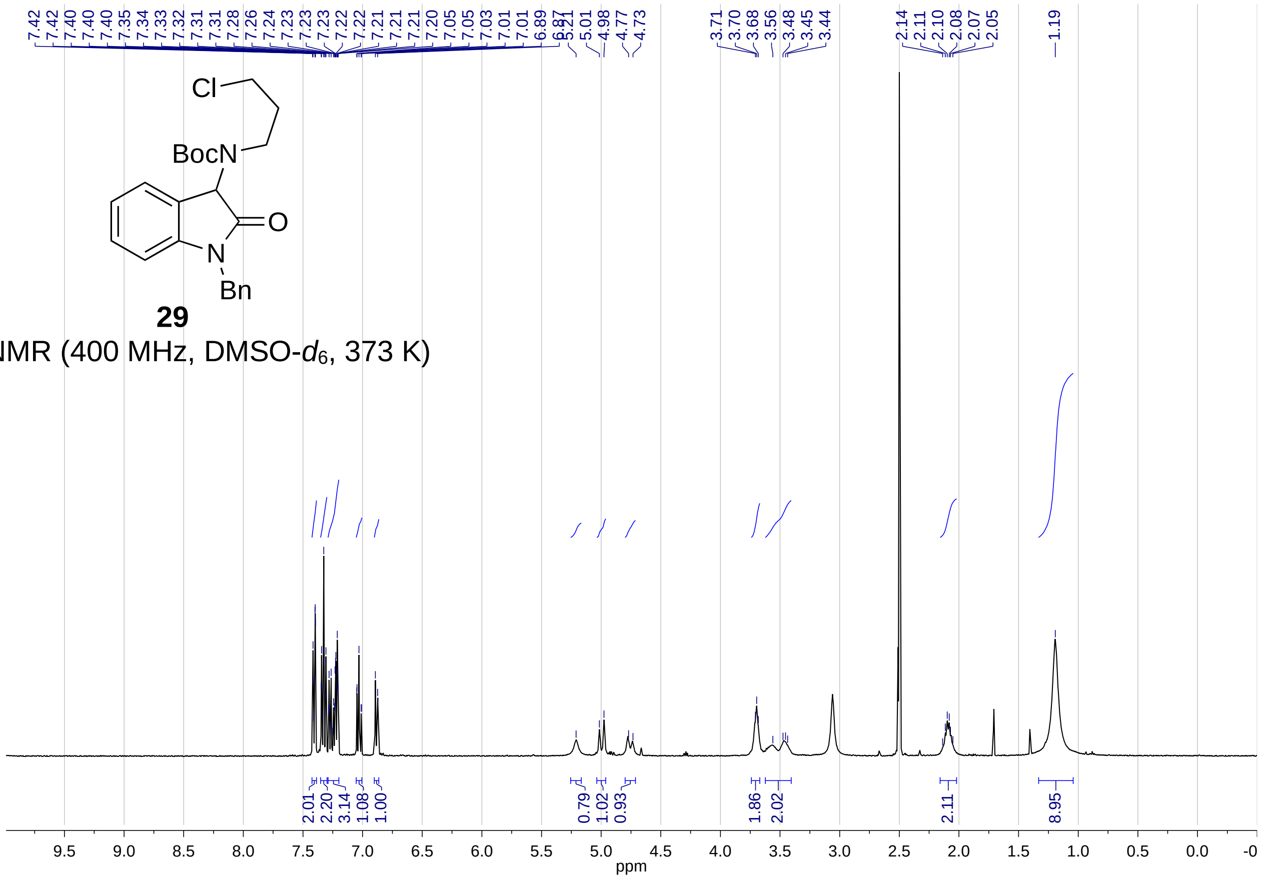

29

^{1}H NMR (400 MHz, DMSO-d_6, 373 K)

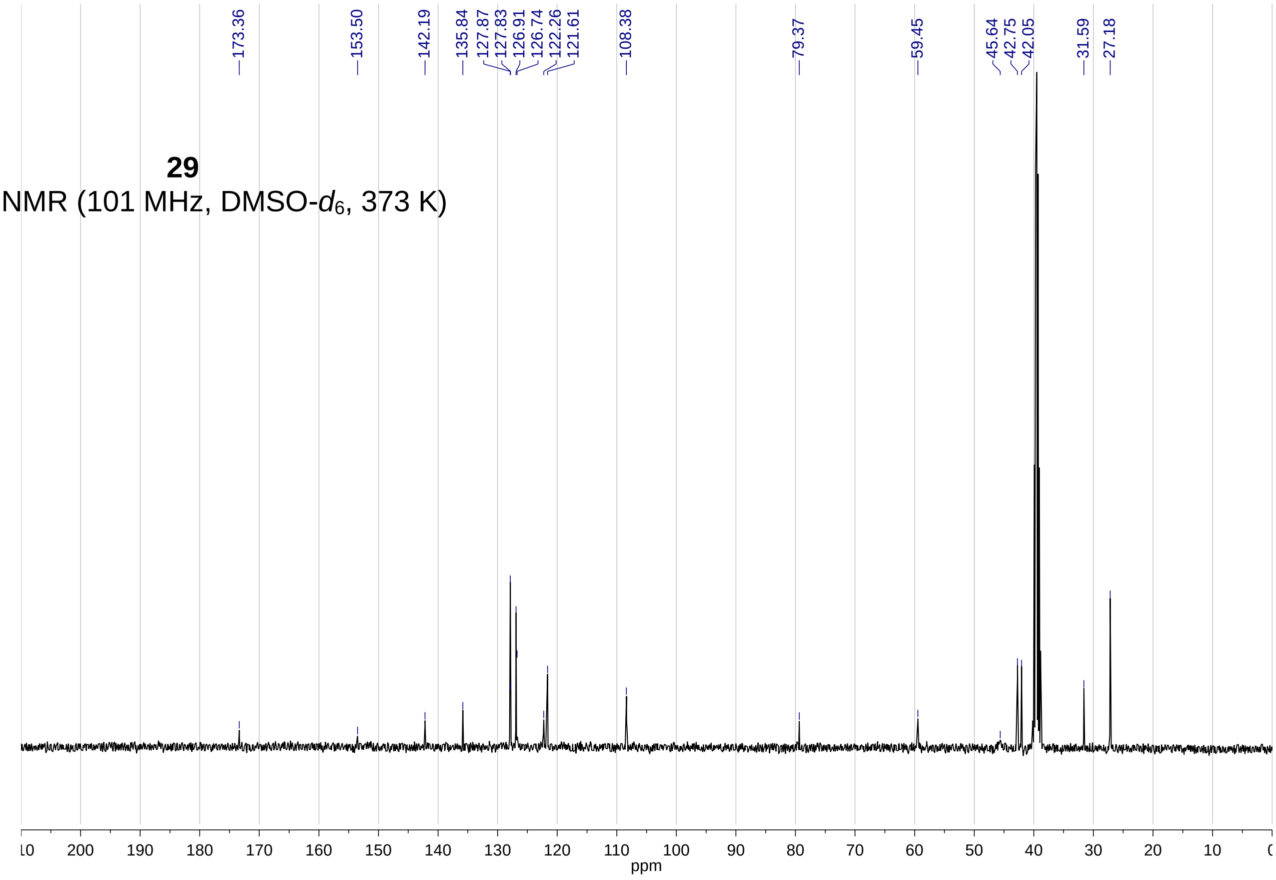

29

^{13}C NMR (101 MHz, DMSO-d_6, 373 K)

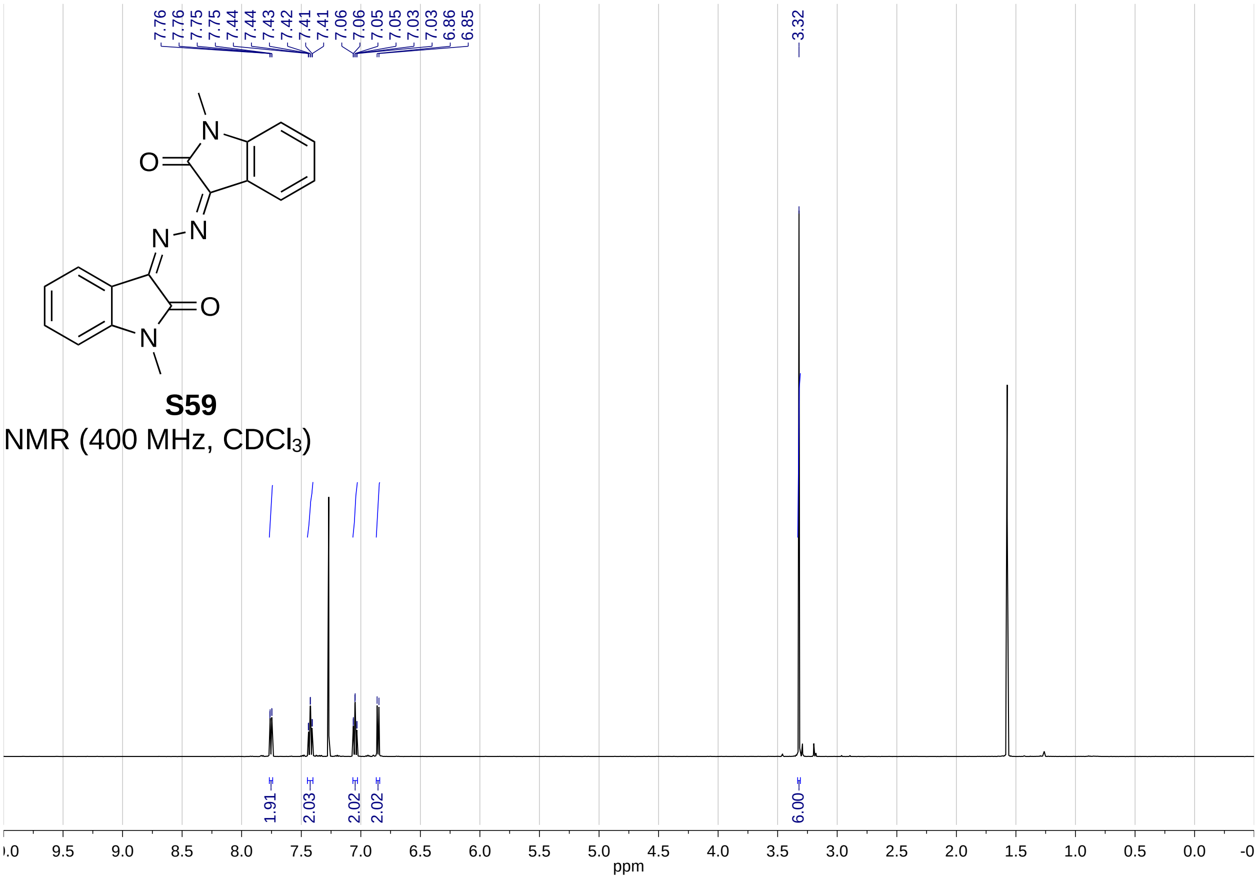

S59

^{1}H NMR (400 MHz, $CDCl_3$)

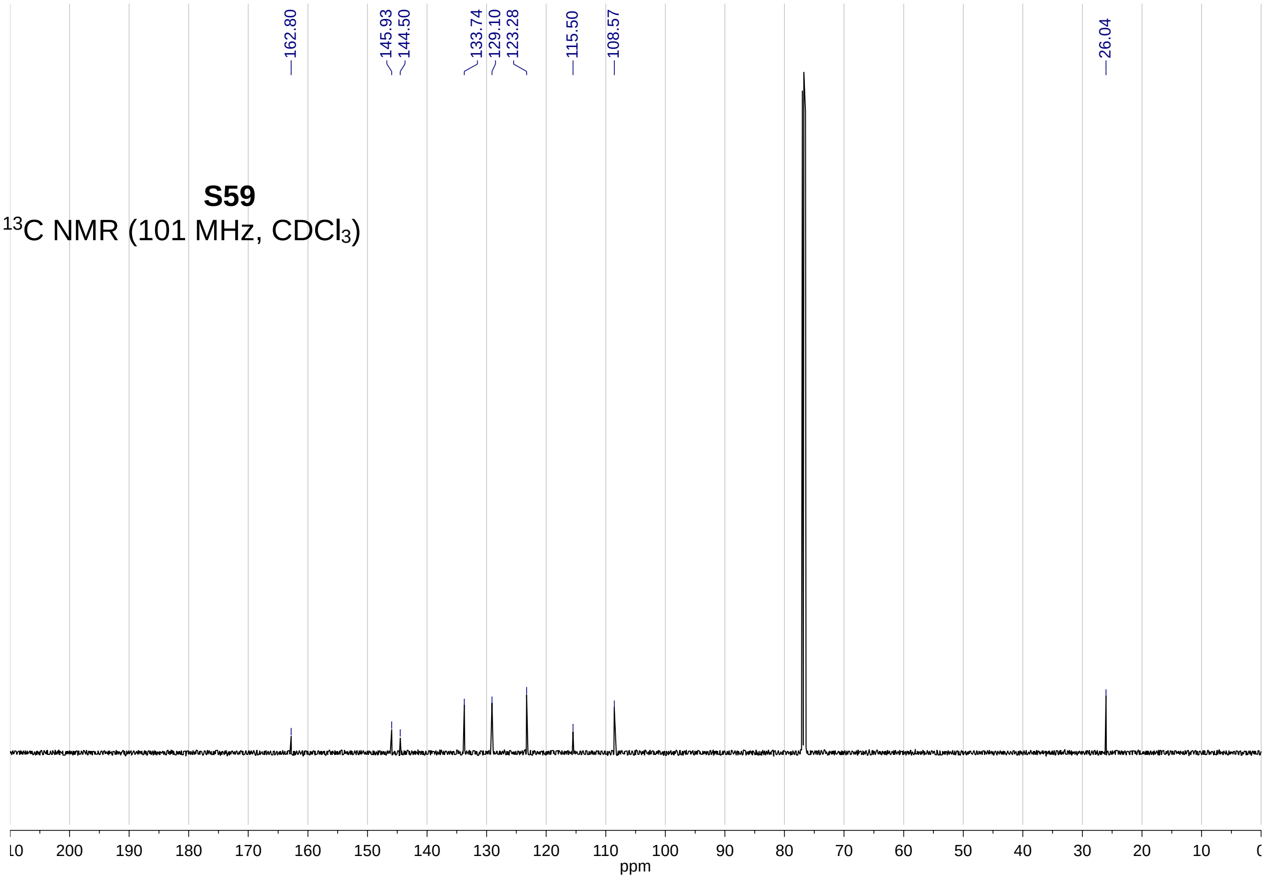

S59

^{13}C NMR (101 MHz, $CDCl_3$)

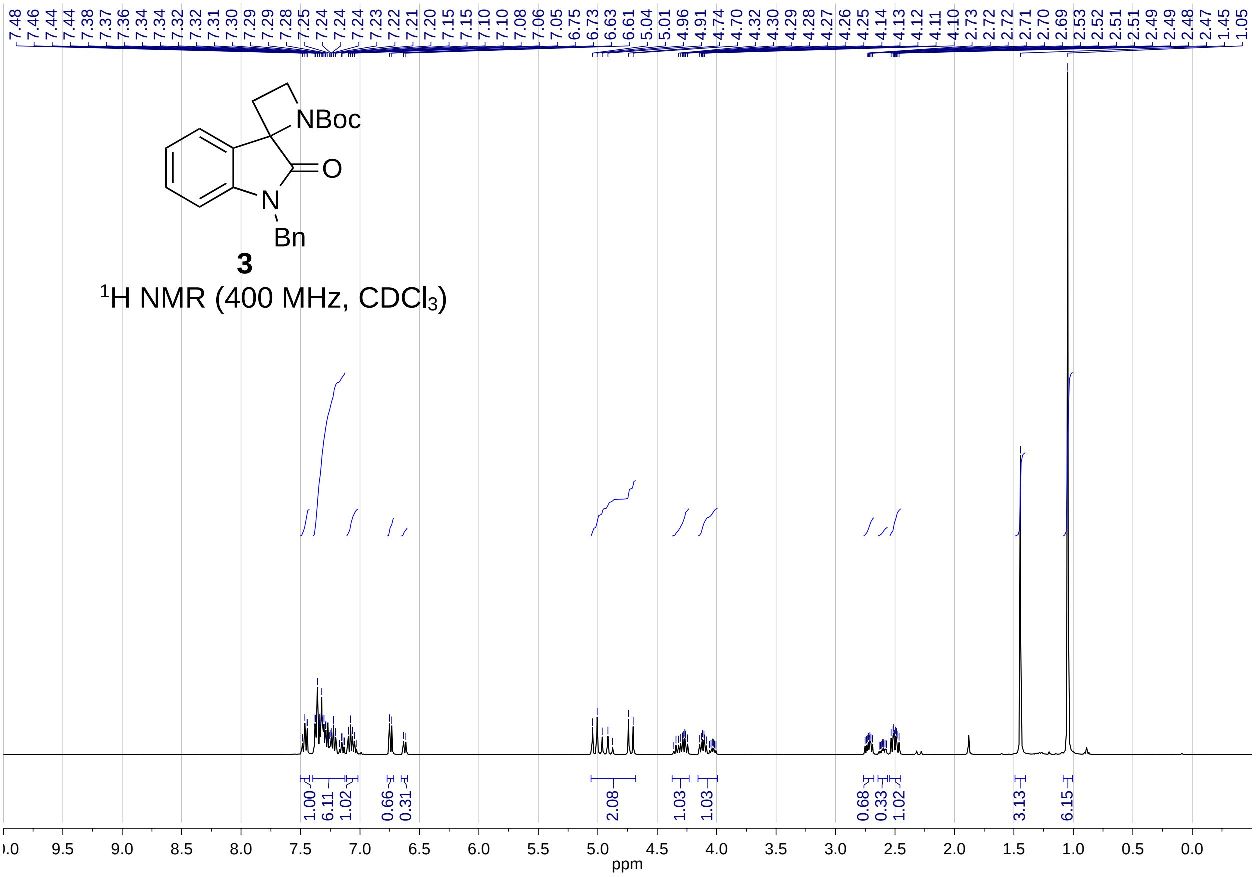
7.48 7.46 7.44 7.38 7.37 7.36 7.34 7.34 7.32 7.32 7.31 7.30 7.29 7.29 7.28 7.25 7.24 7.24 7.24 7.23 7.22 7.21 7.20 7.15 7.15 7.10 7.10 7.08 7.06 7.05 6.75 6.73 6.63 6.61 5.04 5.01 4.96 4.91 4.74 4.70 4.32 4.30 4.29 4.28 4.27 4.26 4.25 4.14 4.13 4.12 4.11 4.10 2.73 2.72 2.72 2.71 2.70 2.69 2.53 2.52 2.51 2.51 2.49 2.48 2.47 1.45 1.05
NBoc
O
N
Bn
3
1H NMR (400 MHz, CDCl3)
1.00 6.11 1.02 0.66 0.31 2.08 1.03 1.03 0.68 0.33 1.02 3.13 6.15
ppm

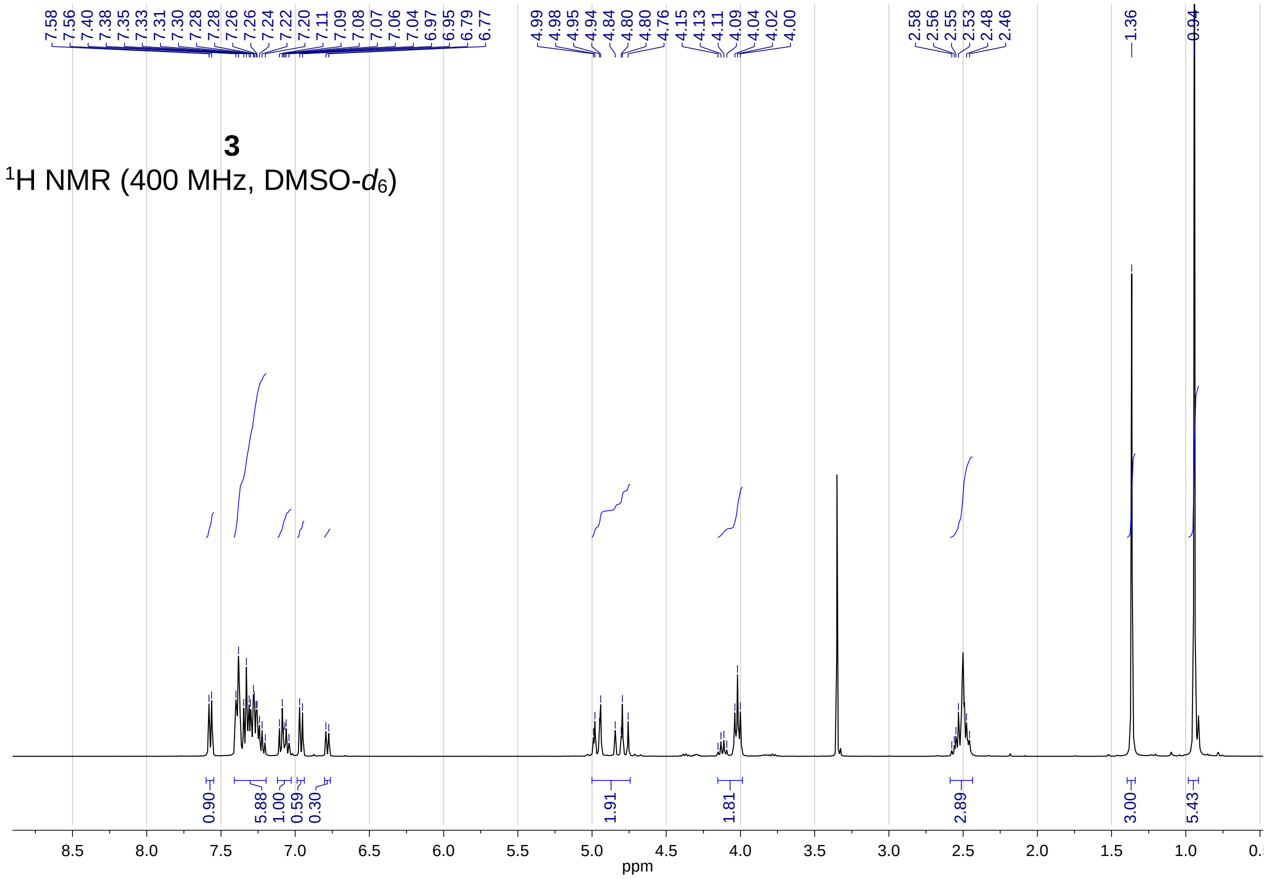
7.58 7.56 7.40 7.38 7.35 7.33 7.31 7.30 7.28 7.28 7.26 7.26 7.24 7.22 7.20 7.11 7.09 7.08 7.07 7.06 7.04 6.97 6.95 6.79 6.77 4.99 4.98 4.95 4.94 4.84 4.80 4.80 4.76 4.15 4.13 4.11 4.09 4.04 4.02 4.00 2.58 2.56 2.55 2.53 2.48 2.46 1.36 0.94
3
1H NMR (400 MHz, DMSO-d6)
0.90 5.88 1.00 0.59 0.30 1.91 1.81 2.89 3.00 5.43
ppm

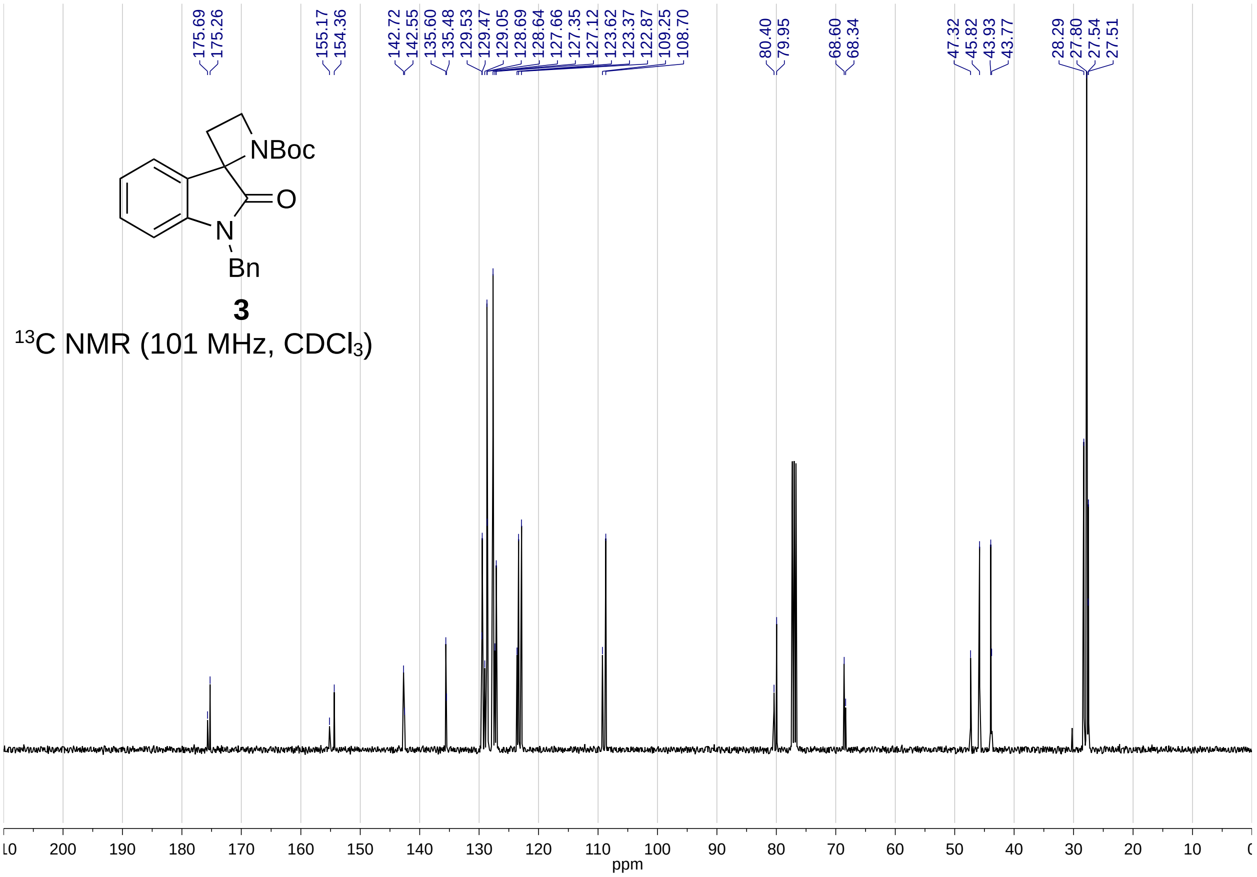

3

^{13}C NMR (101 MHz, DMSO-d_6)

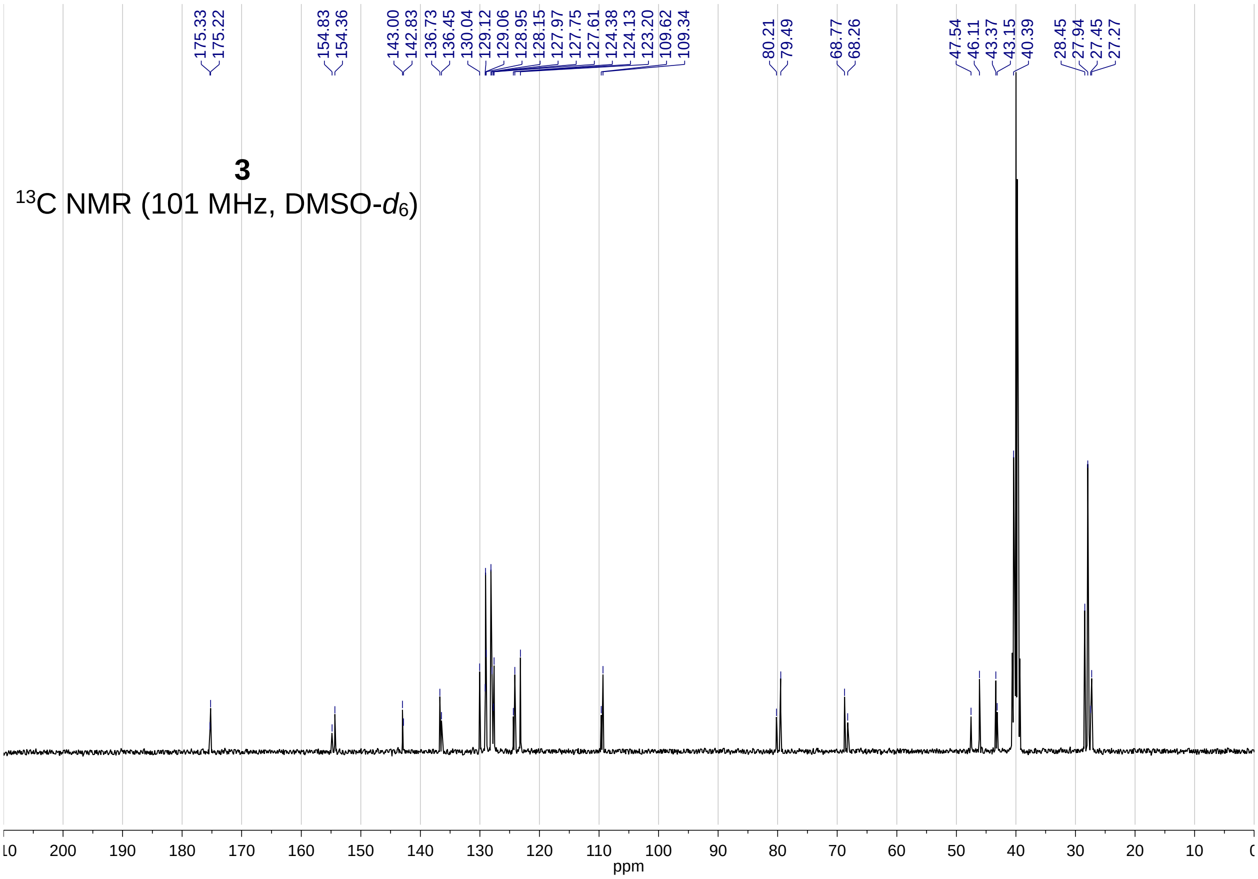

3
(HSQC, DMSO-d_6, 373 K)

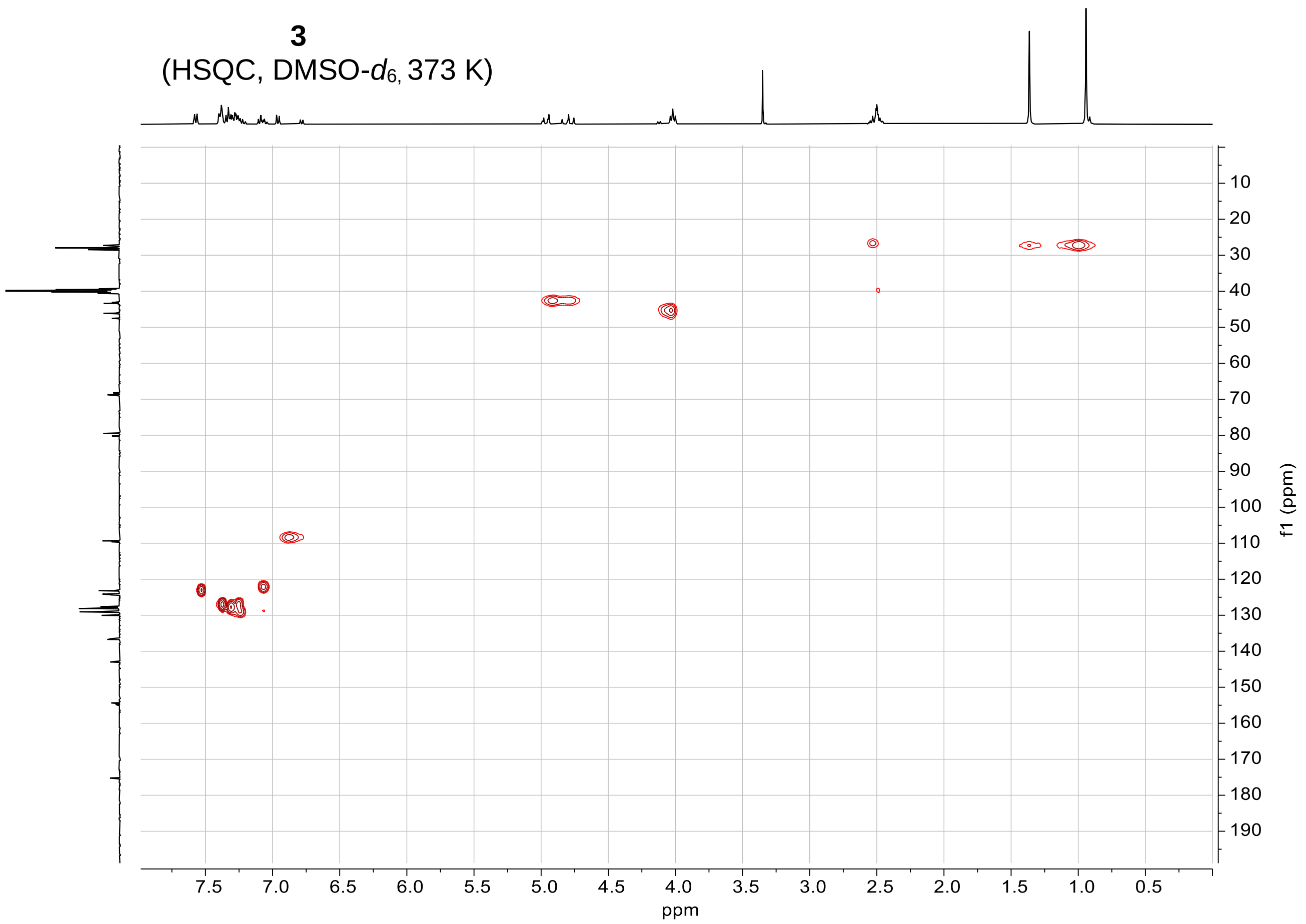

3
(HMBC, DMSO-d_6, 373 K)

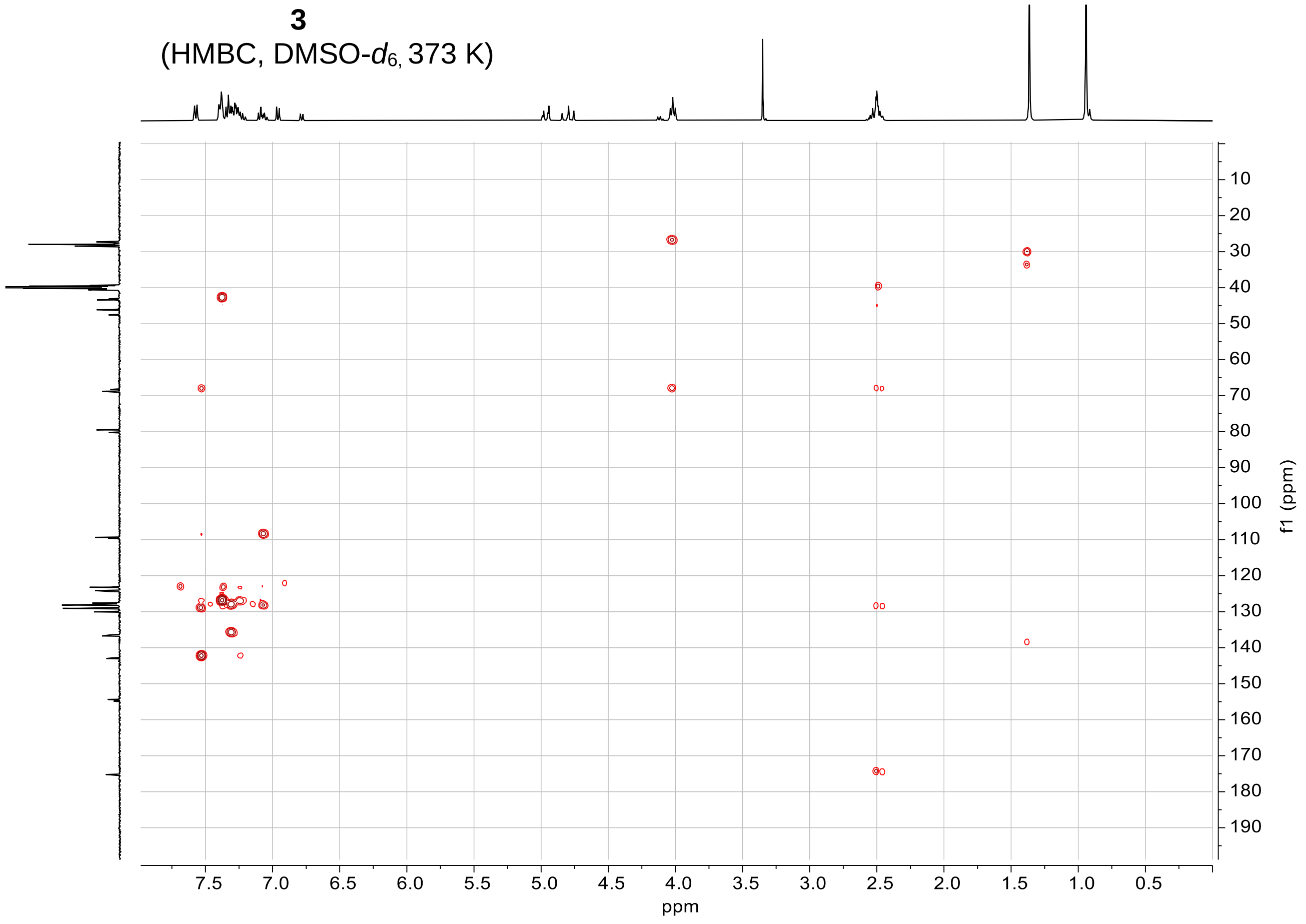

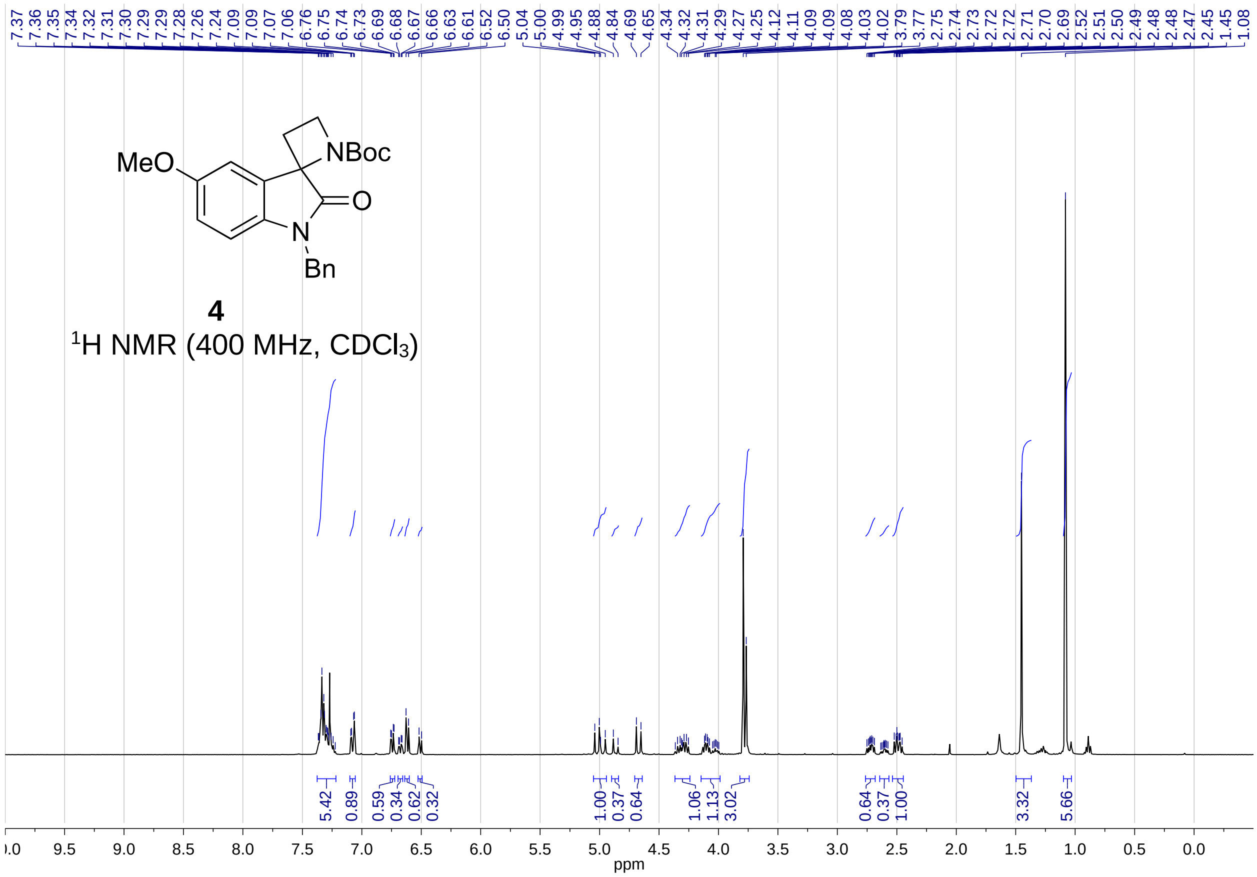
MeO
NBoc
O
N
Bn
4
1H NMR (400 MHz, CDCl3)
ppm

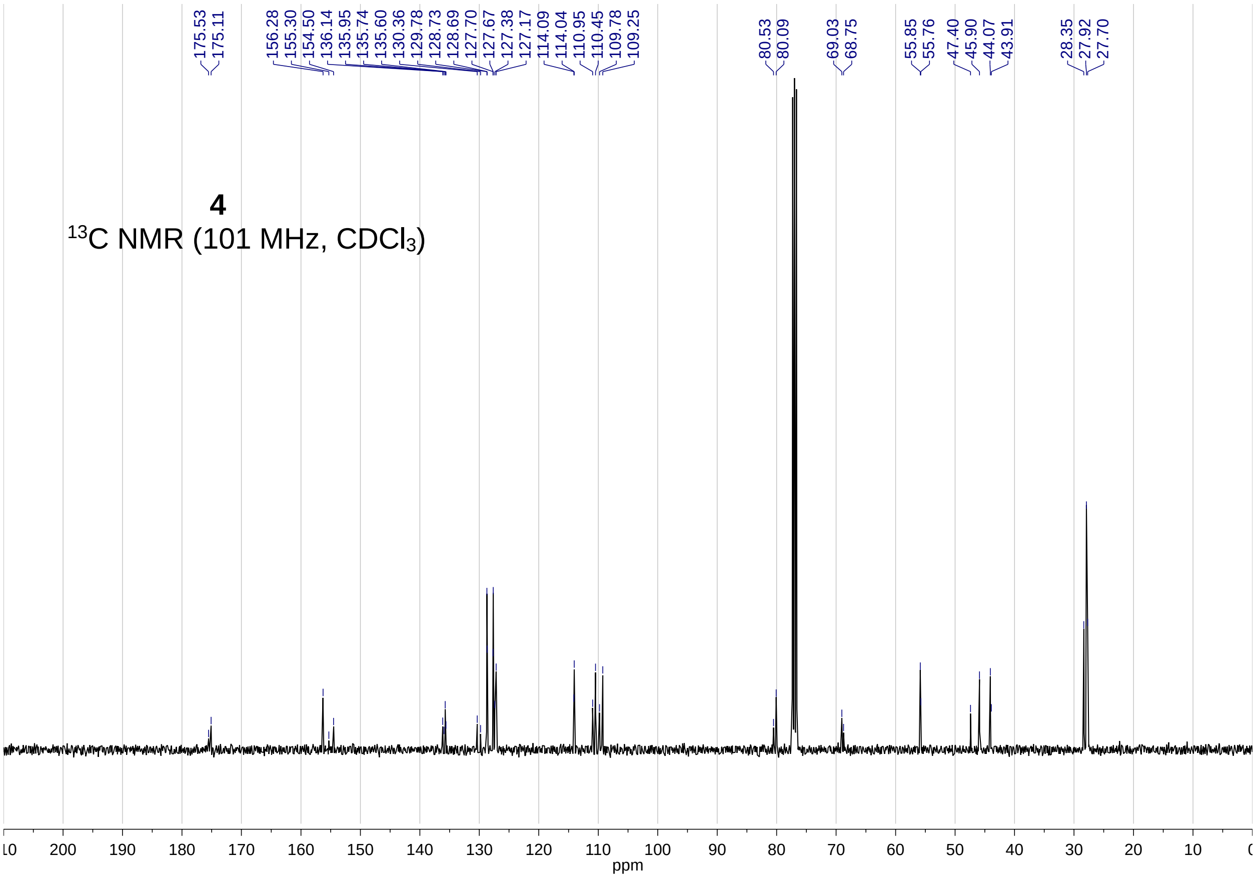
4
13C NMR (101 MHz, CDCl3)
ppm

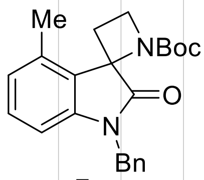

5

^{1}H NMR (400 MHz, $CDCl_3$)

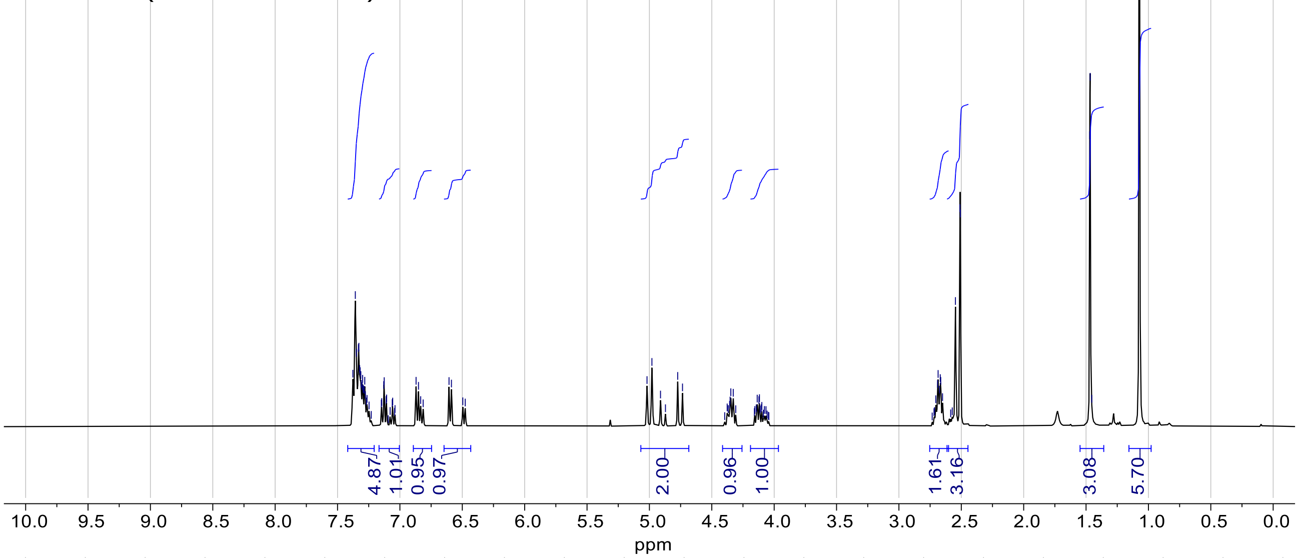

5

^{13}C NMR (101 MHz, $CDCl_3$)

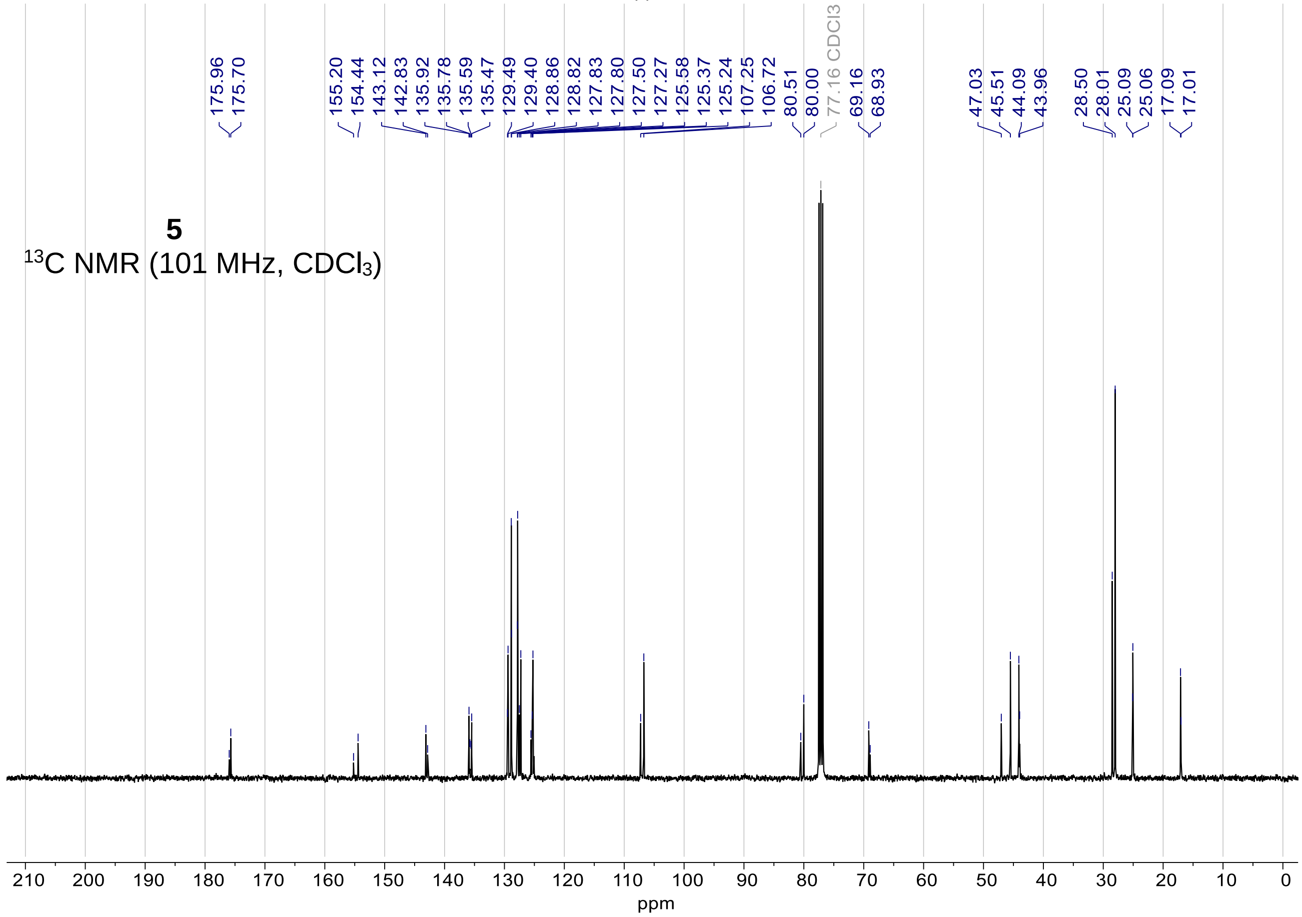

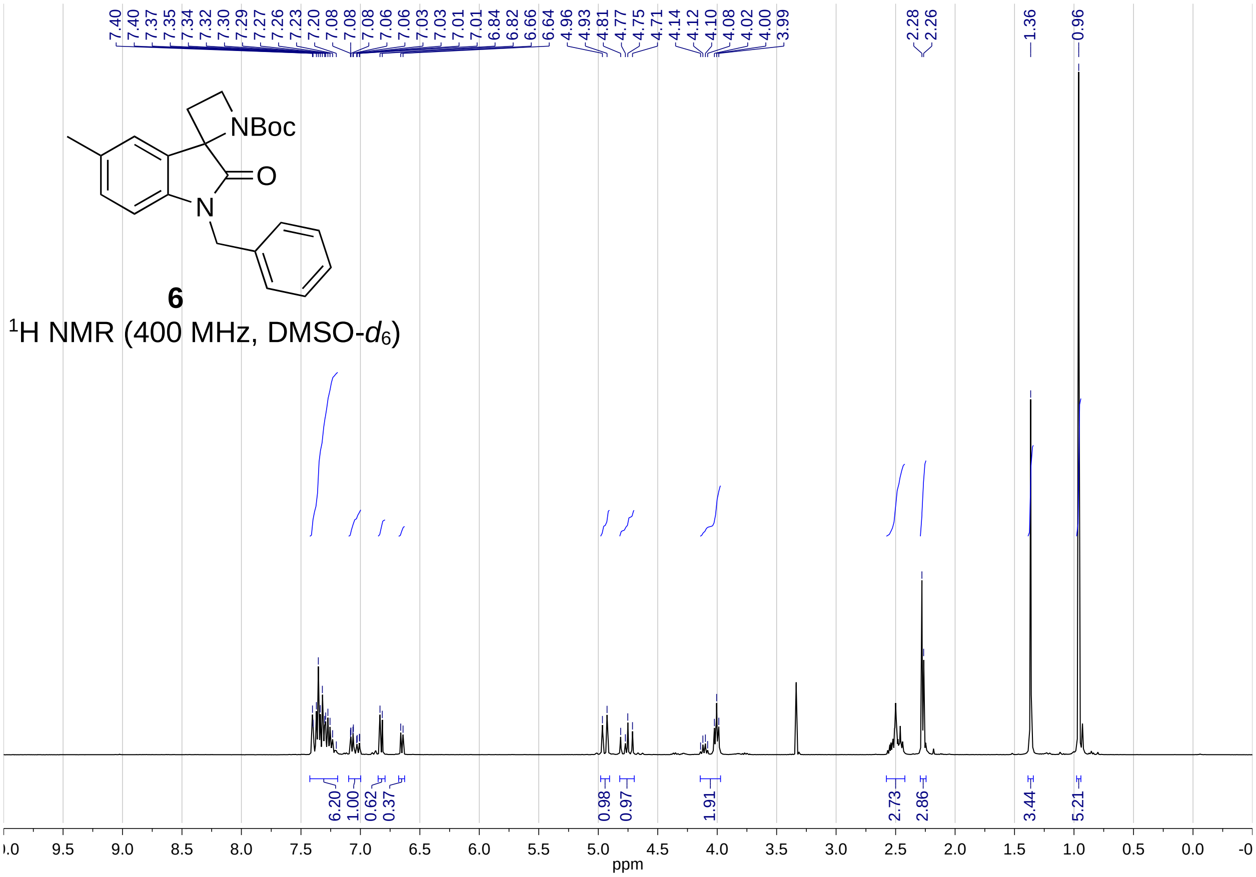

6

^{1}H NMR (400 MHz, DMSO-d_6)

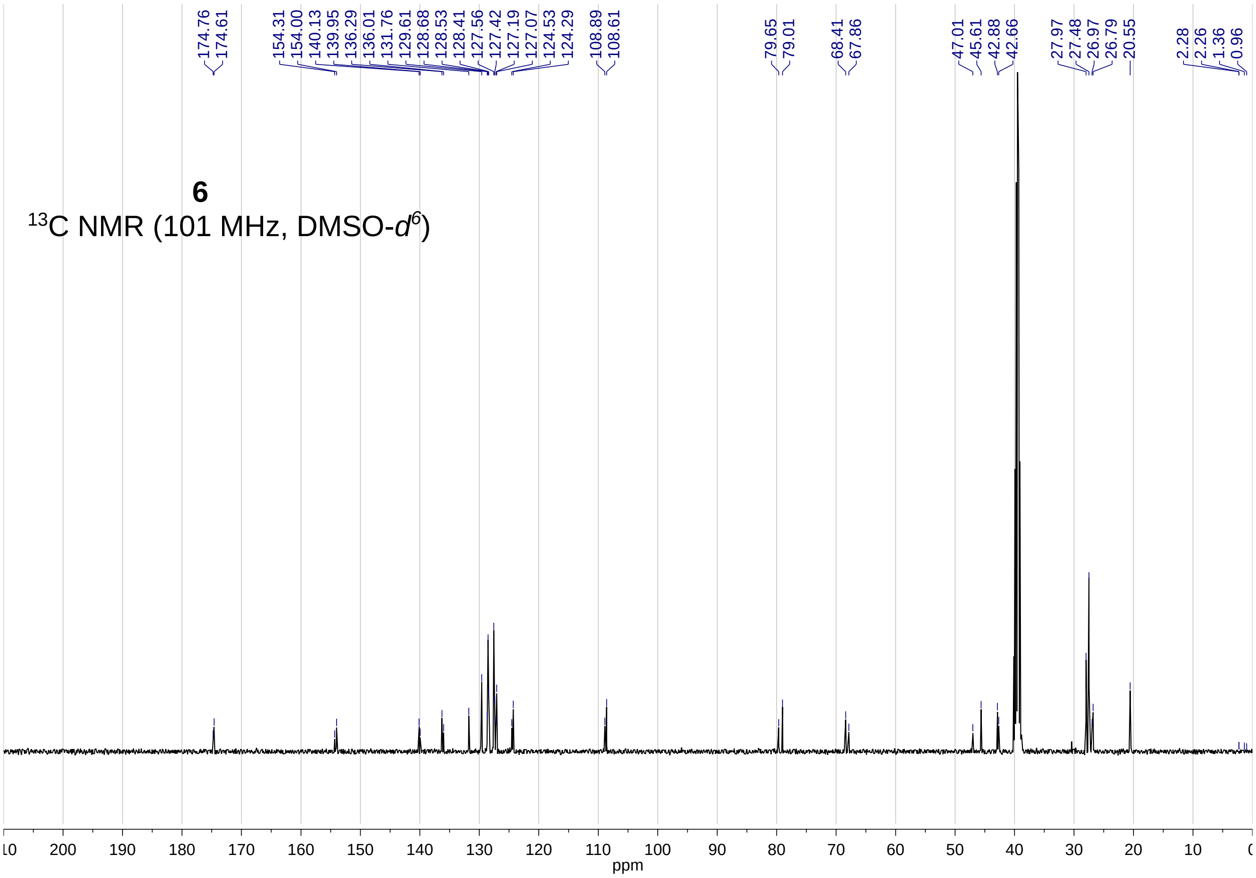

6

^{13}C NMR (101 MHz, DMSO-d^6)

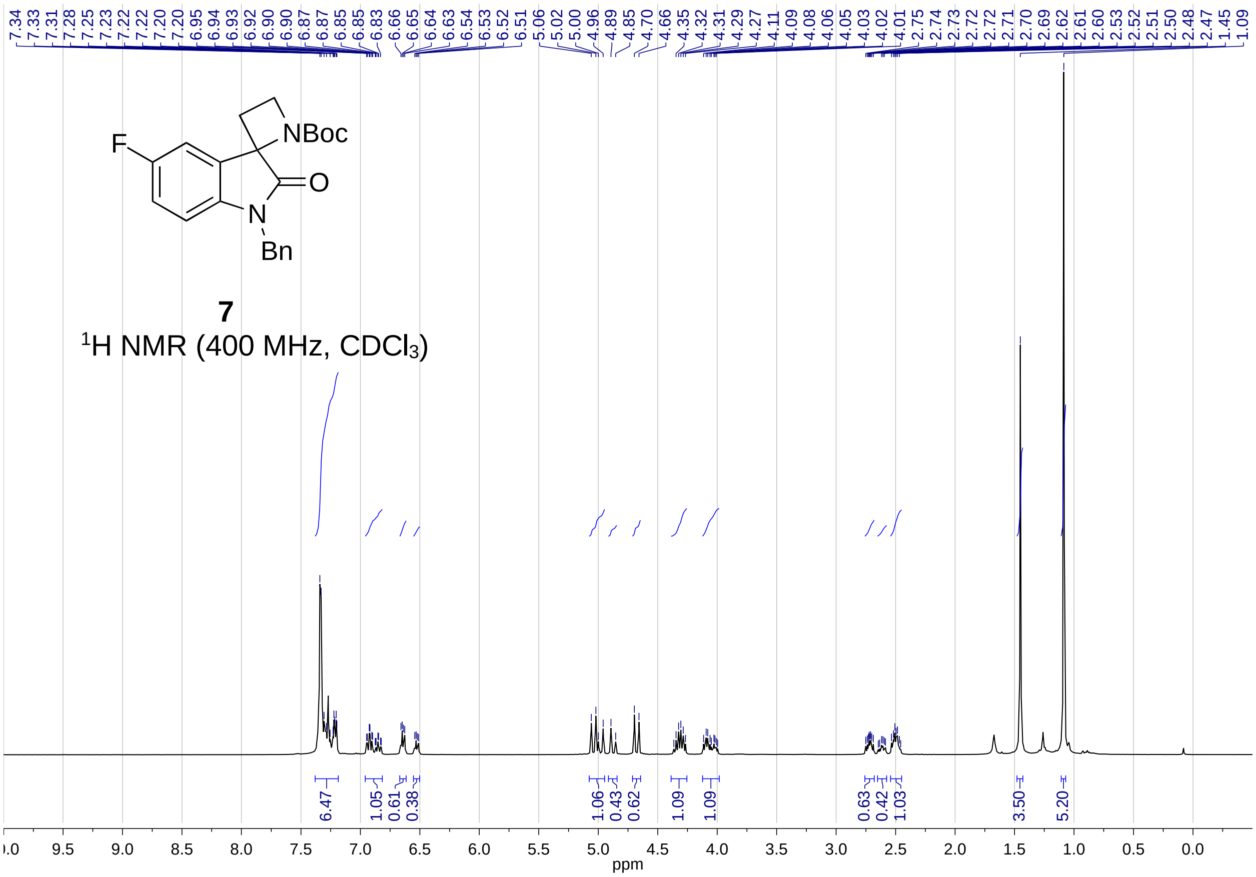

7

^{1}H NMR (400 MHz, $CDCl_3$)

7

^{13}C NMR (101 MHz, $CDCl_3$)

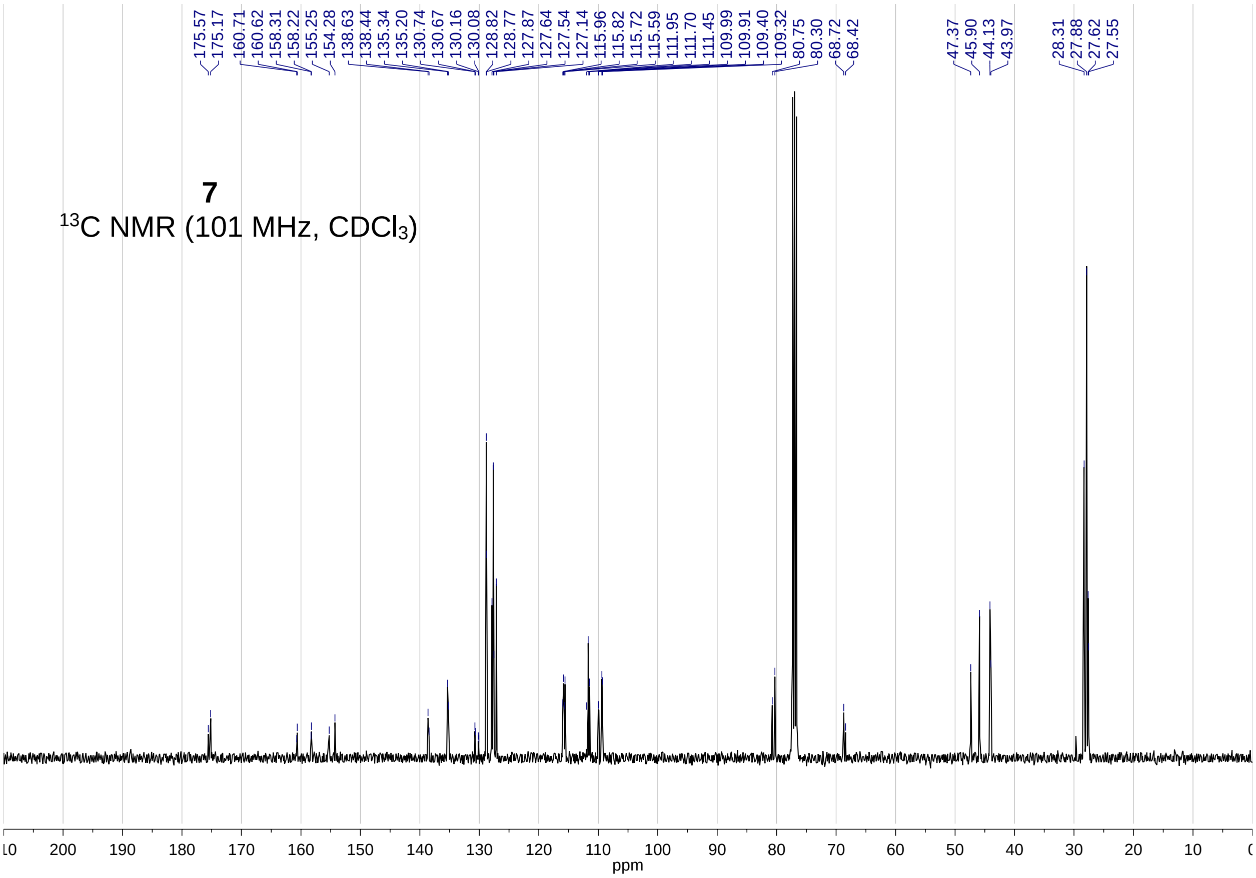

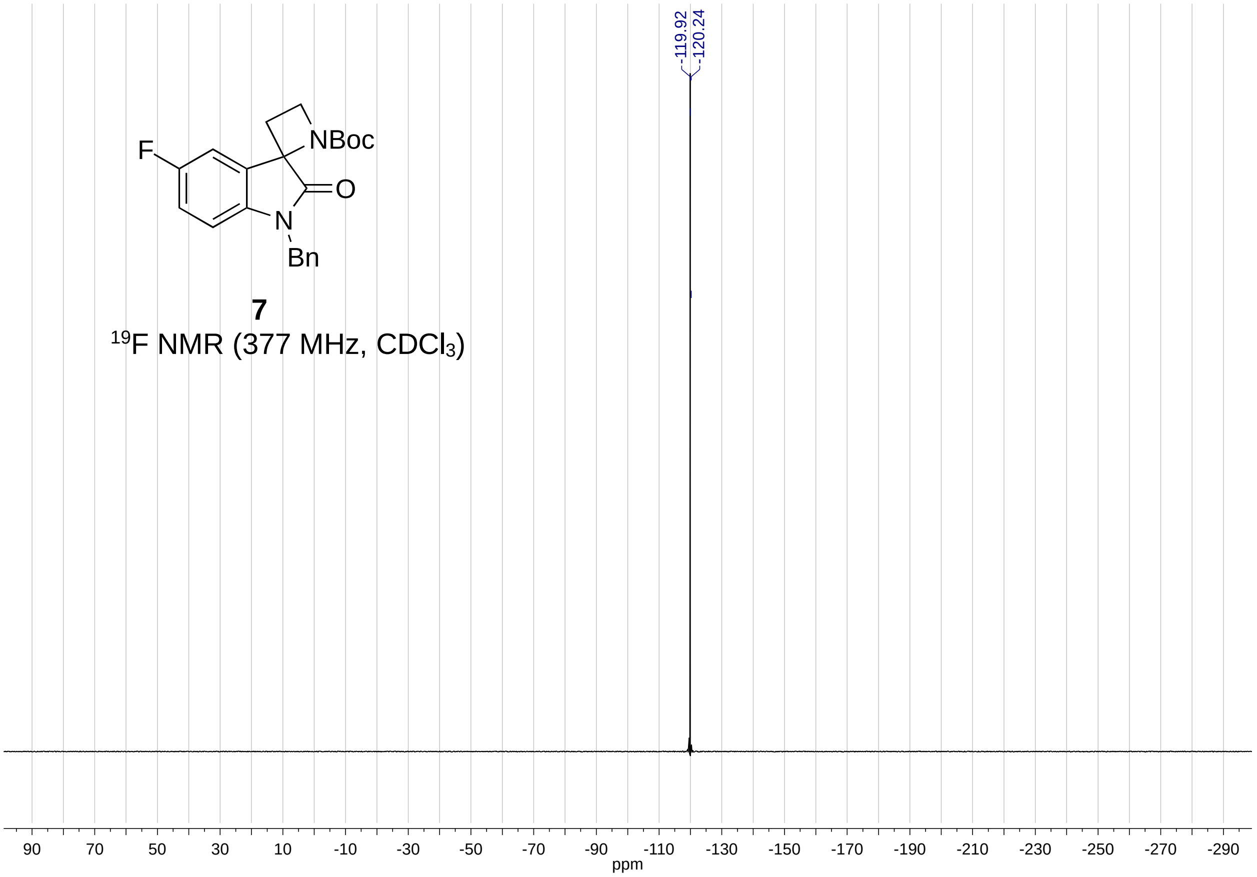
F
NBoc
O
N
Bn
7
19F NMR (377 MHz, CDCl3)
-119.92
-120.24
90
70
50
30
10
-10
-30
-50
-70
-90
-110
-130
-150
-170
-190
-210
-230
-250
-270
-290
ppm

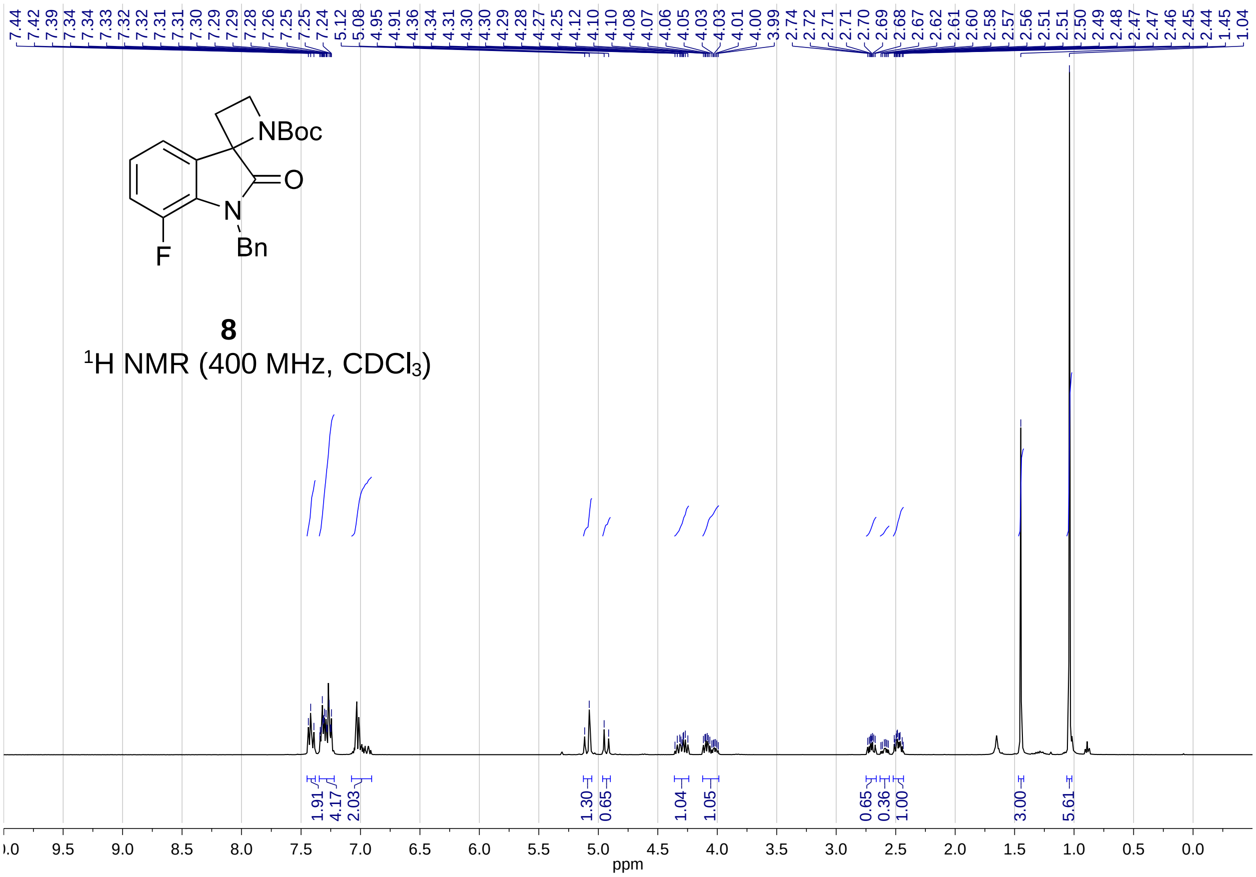
7.44 7.42 7.39 7.34 7.34 7.33 7.32 7.32 7.31 7.31 7.30 7.29 7.29 7.28 7.26 7.25 7.25 7.24 5.12 5.08 4.95 4.91 4.36 4.34 4.31 4.30 4.30 4.29 4.28 4.27 4.25 4.12 4.10 4.10 4.08 4.07 4.06 4.05 4.03 4.03 4.01 4.00 3.99 2.74 2.72 2.71 2.70 2.69 2.68 2.67 2.62 2.61 2.60 2.58 2.57 2.56 2.51 2.50 2.49 2.48 2.47 2.47 2.46 2.45 2.44 1.45 1.04
NBoc
O
N
Bn
F
8
1H NMR (400 MHz, CDCl3)
1.91 4.17 2.03 1.30 0.65 1.04 1.05 0.65 0.36 1.00 3.00 5.61
ppm

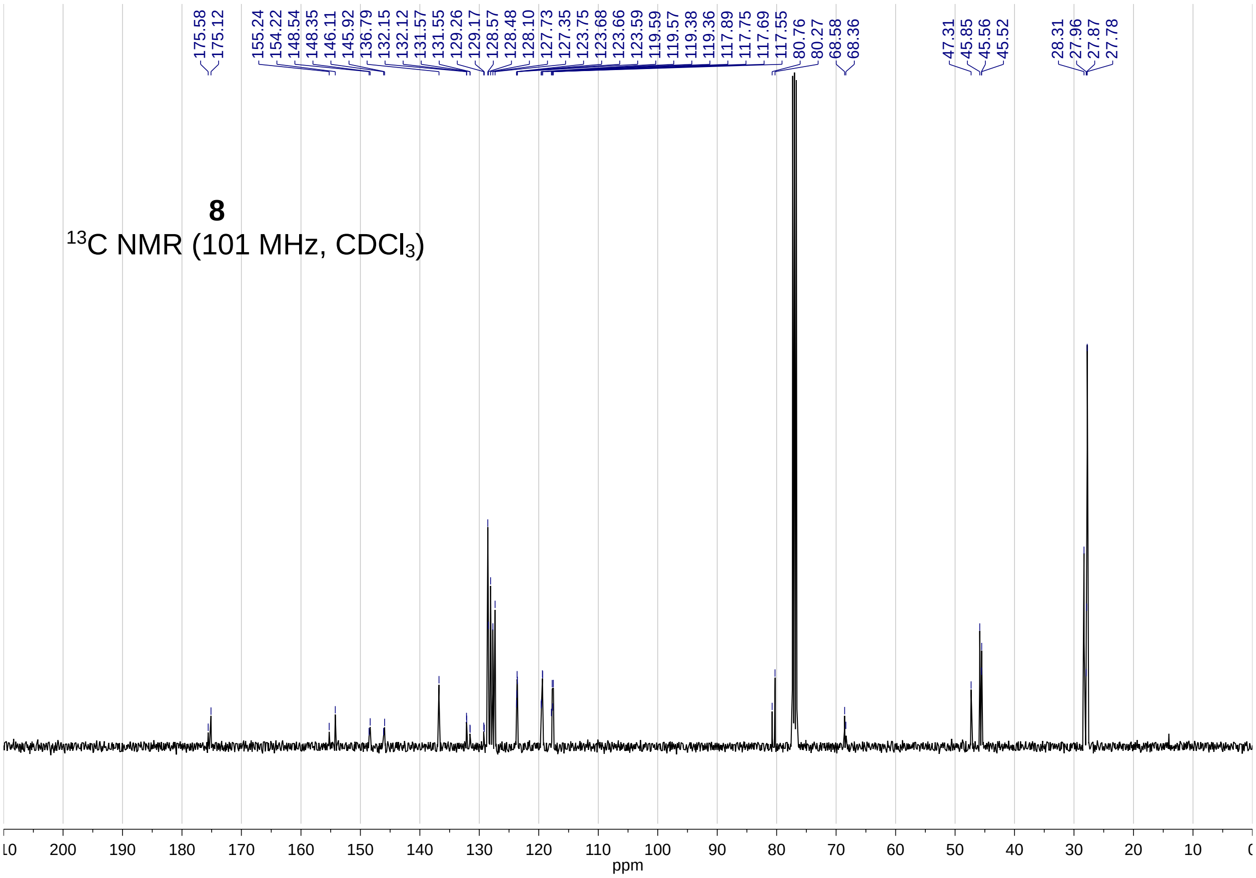
175.58 175.12 155.24 154.22 148.54 148.35 146.11 145.92 136.79 132.15 132.12 131.57 131.55 129.26 129.17 128.57 128.48 128.10 127.73 127.35 123.75 123.68 123.66 123.59 119.59 119.57 119.38 119.36 117.89 117.75 117.69 117.55 80.76 80.27 68.58 68.36 47.31 45.85 45.56 45.52 28.31 27.96 27.87 27.78
8
13C NMR (101 MHz, CDCl3)
ppm

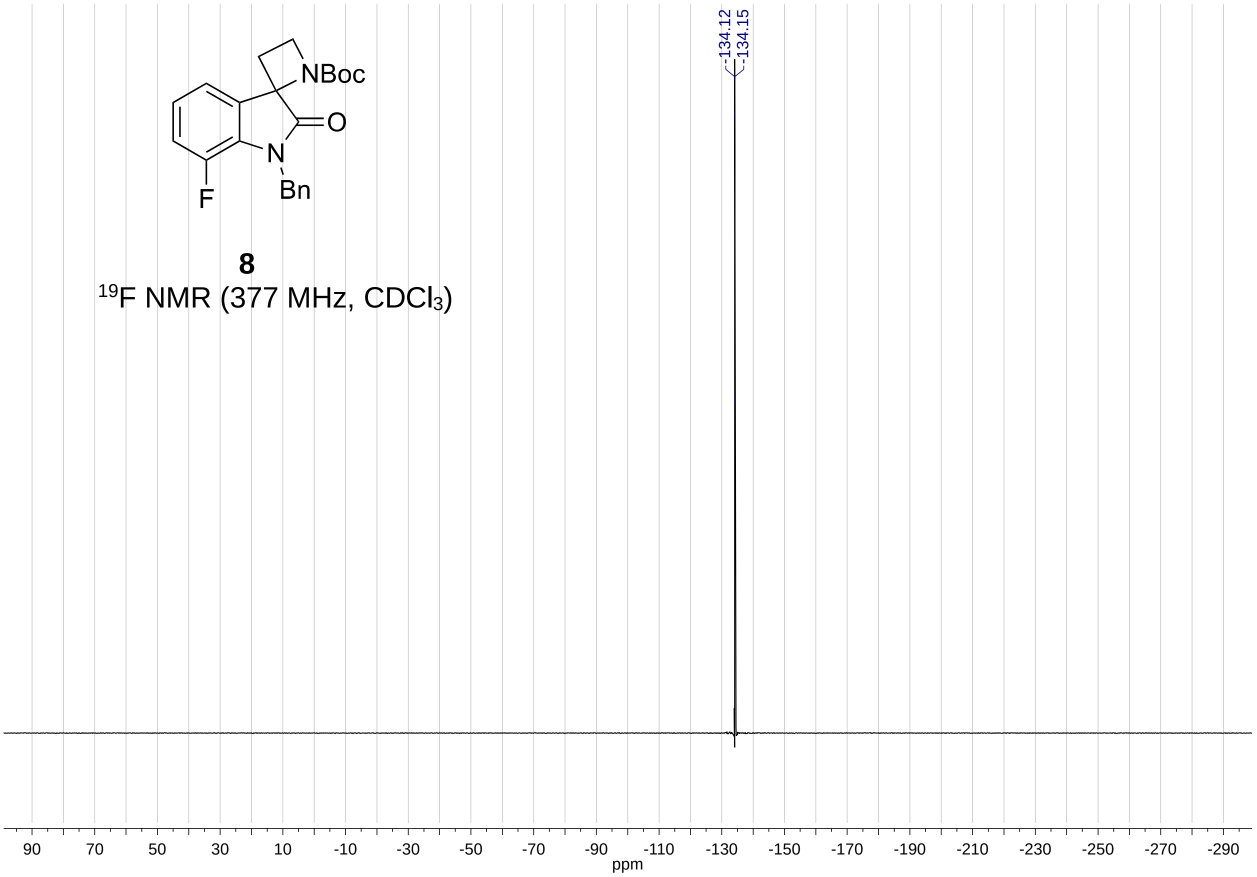

8

^{19}F NMR (377 MHz, $CDCl_3$)

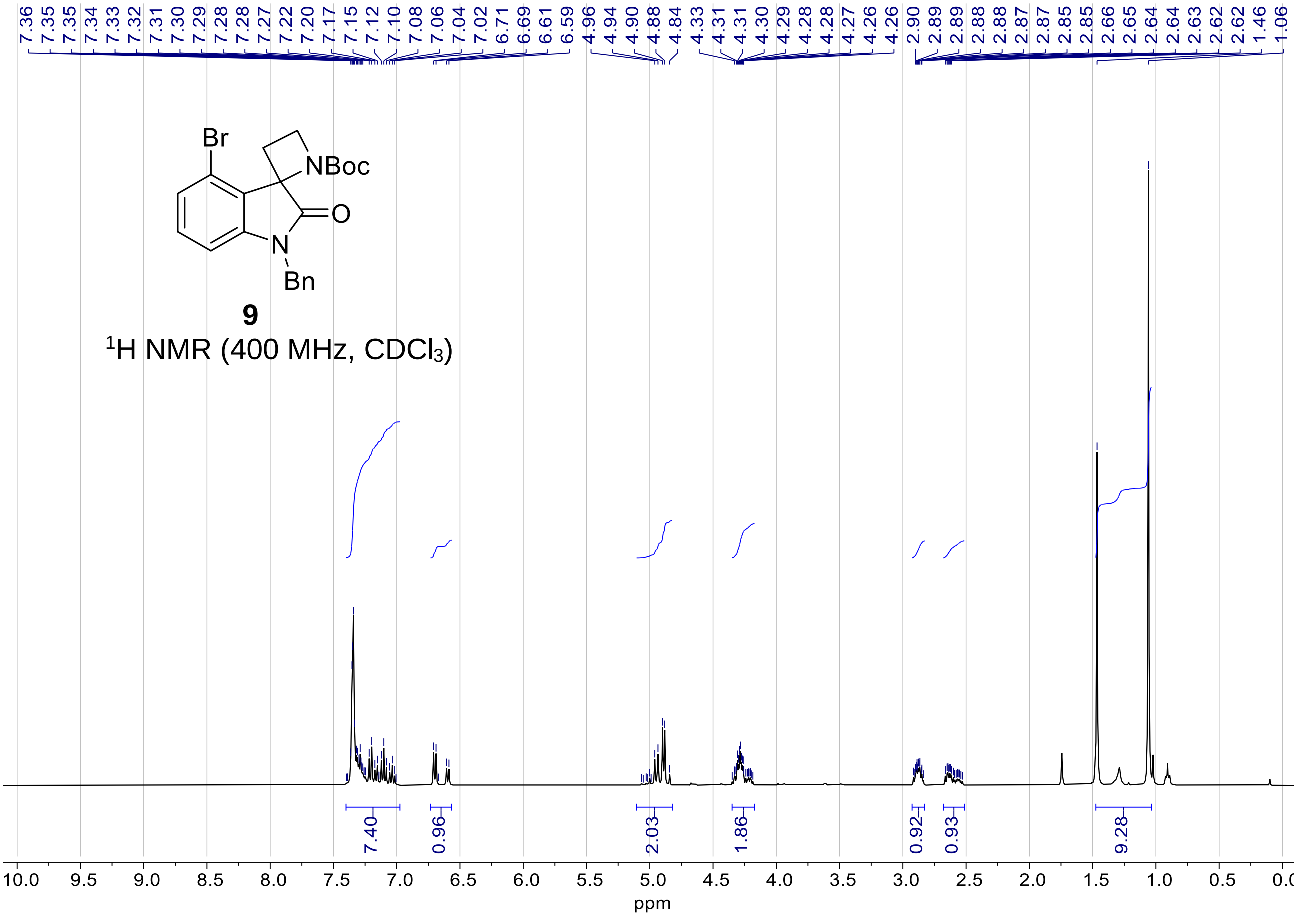

7.36 7.35 7.35 7.34 7.33 7.32 7.31 7.30 7.29 7.28 7.28 7.27 7.22 7.20 7.17 7.15 7.12 7.10 7.08 7.06 7.04 7.02 6.71 6.69 6.61 6.59 4.96 4.94 4.90 4.88 4.84 4.33 4.31 4.31 4.30 4.29 4.28 4.28 4.27 4.26 4.26 2.90 2.89 2.89 2.88 2.88 2.87 2.87 2.85 2.85 2.66 2.65 2.64 2.64 2.63 2.62 2.62 1.46 1.06
Br
NBoc
O
N
Bn
9
^{1}H NMR (400 MHz, CDCl$_3$)
7.40
0.96
2.03
1.86
0.92
0.93
9.28
ppm

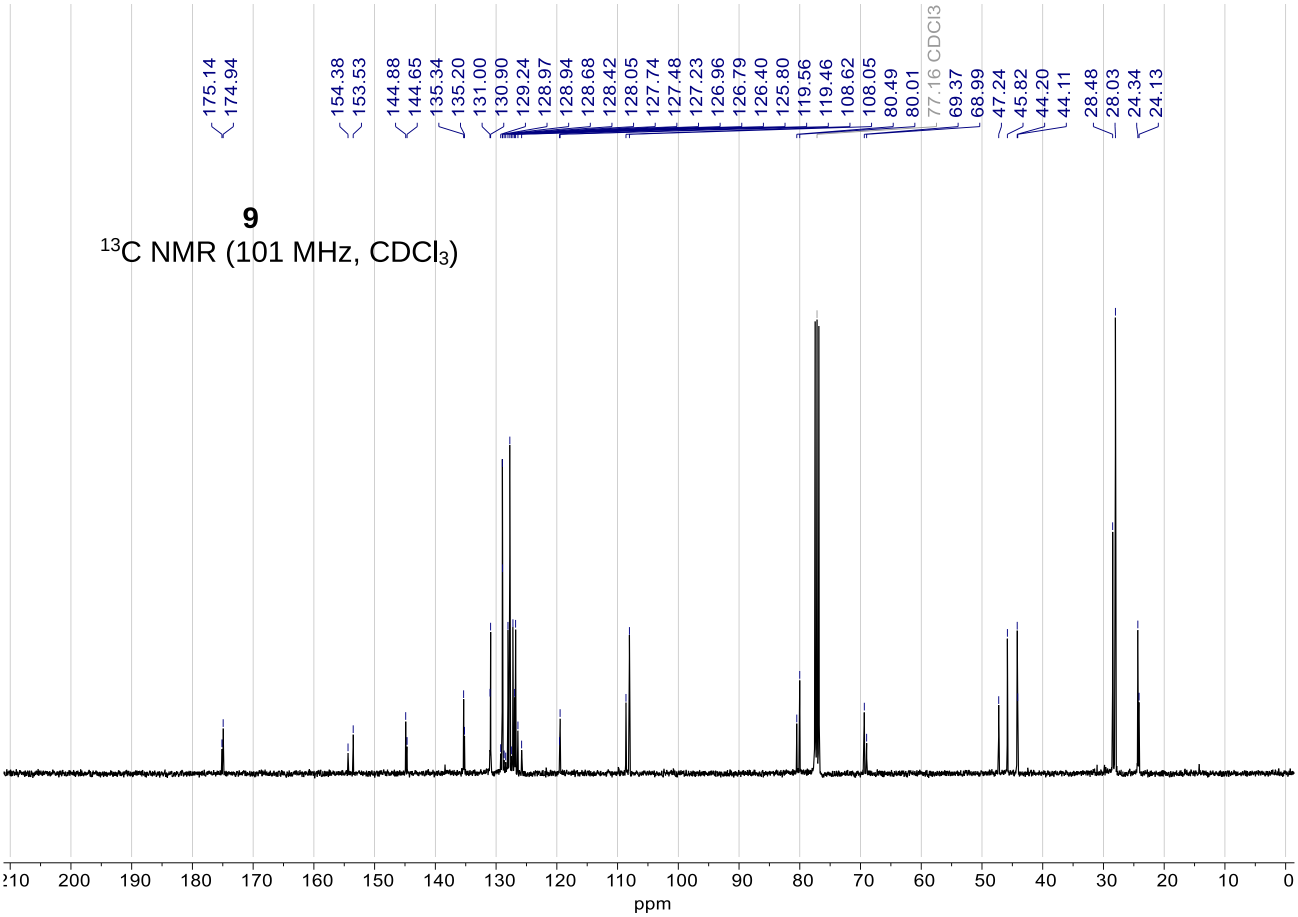

175.14 174.94 154.38 153.53 144.88 144.65 135.34 135.20 131.00 130.90 129.24 128.97 128.94 128.68 128.42 128.05 127.74 127.48 127.23 126.96 126.79 126.40 125.80 119.56 119.46 108.62 108.05 80.49 80.01 77.16 CDCl3 69.37 68.99 47.24 45.82 44.20 44.11 28.48 28.03 24.34 24.13
9
^{13}C NMR (101 MHz, CDCl$_3$)
ppm

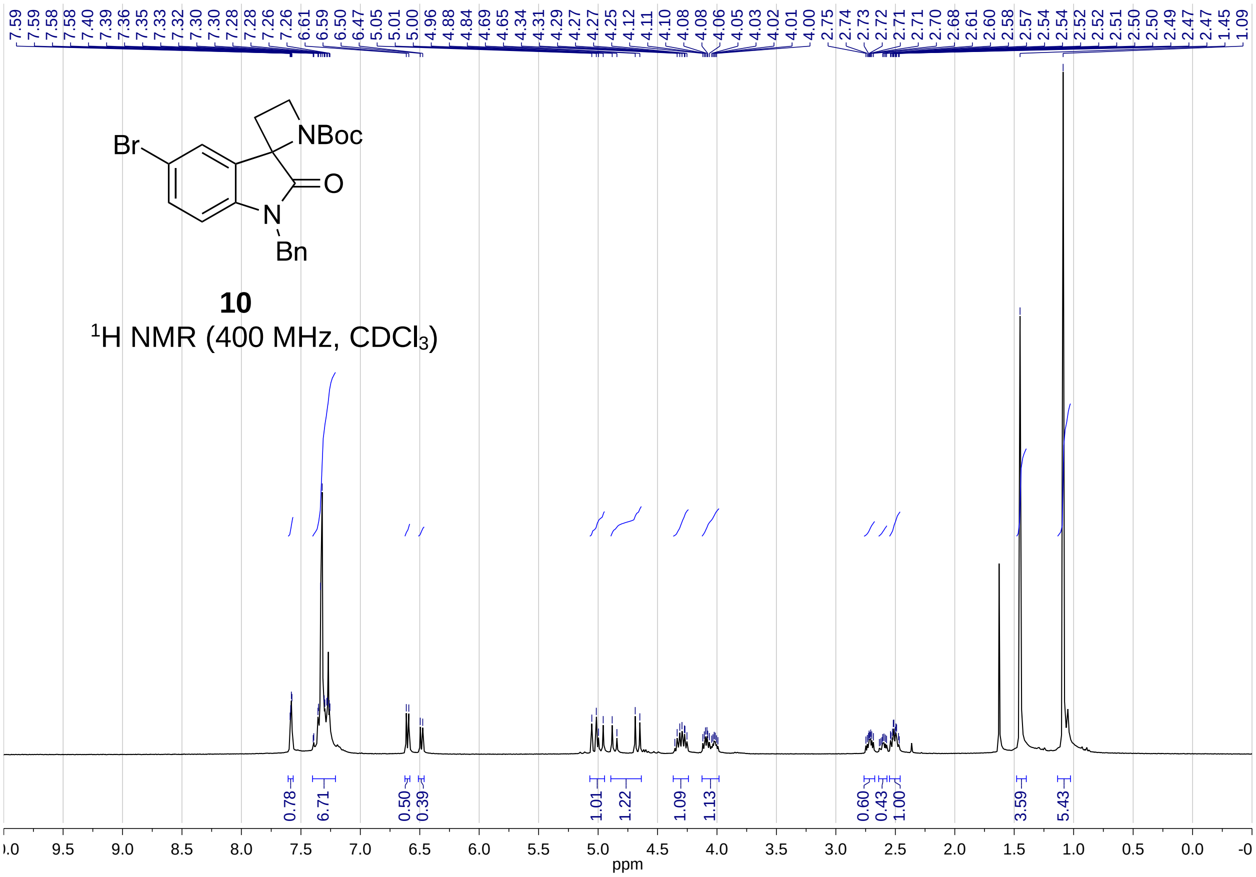

10

^{1}H NMR (400 MHz, $CDCl_3$)

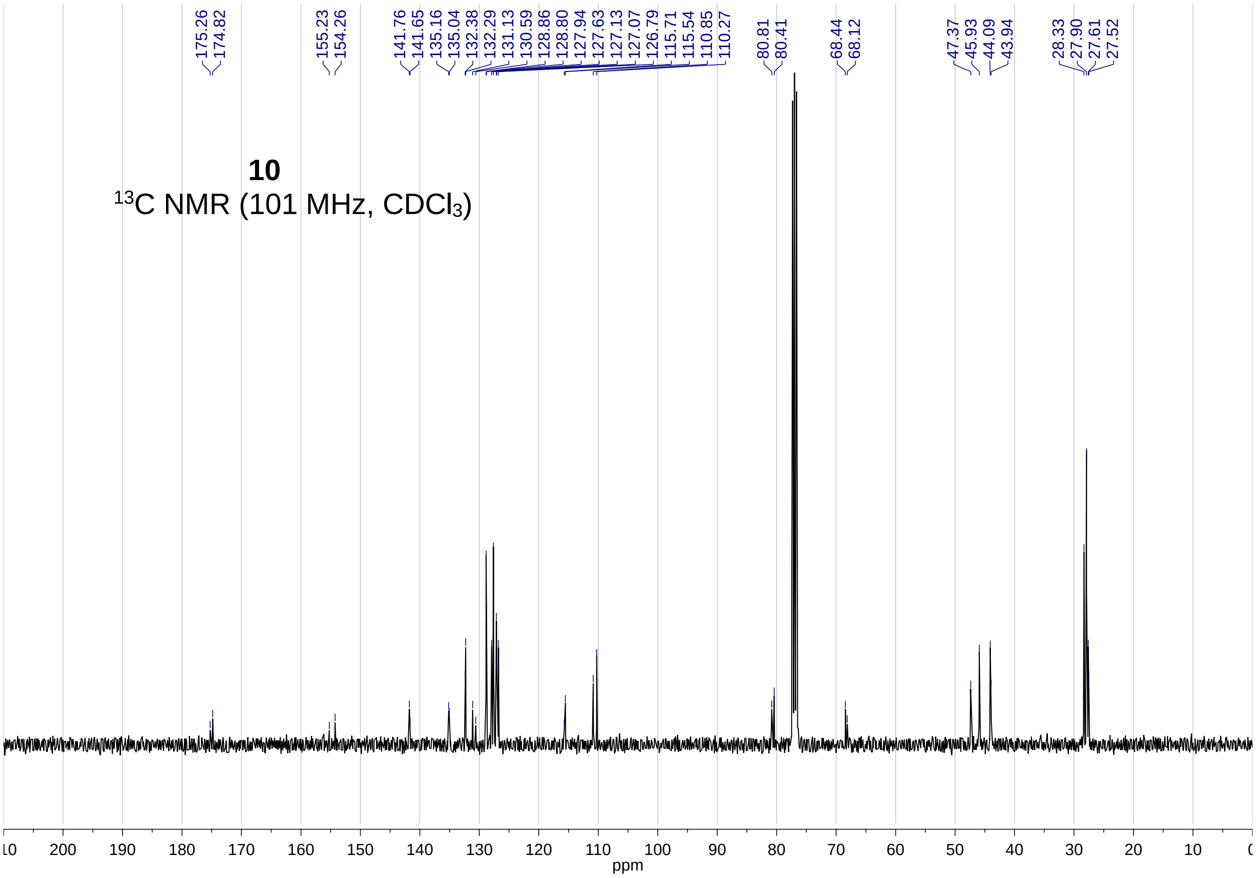

10

^{13}C NMR (101 MHz, $CDCl_3$)

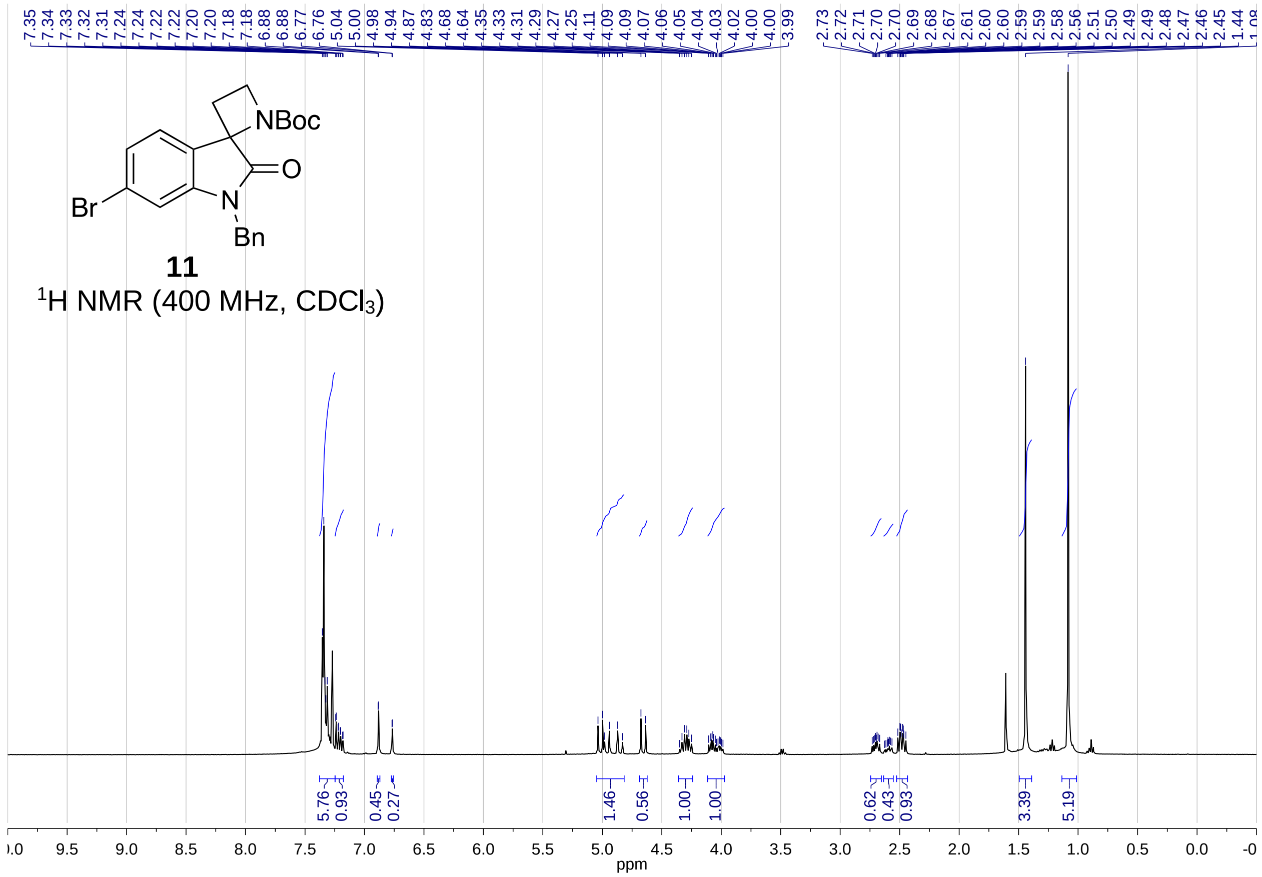

11

^{1}H NMR (400 MHz, $CDCl_3$)

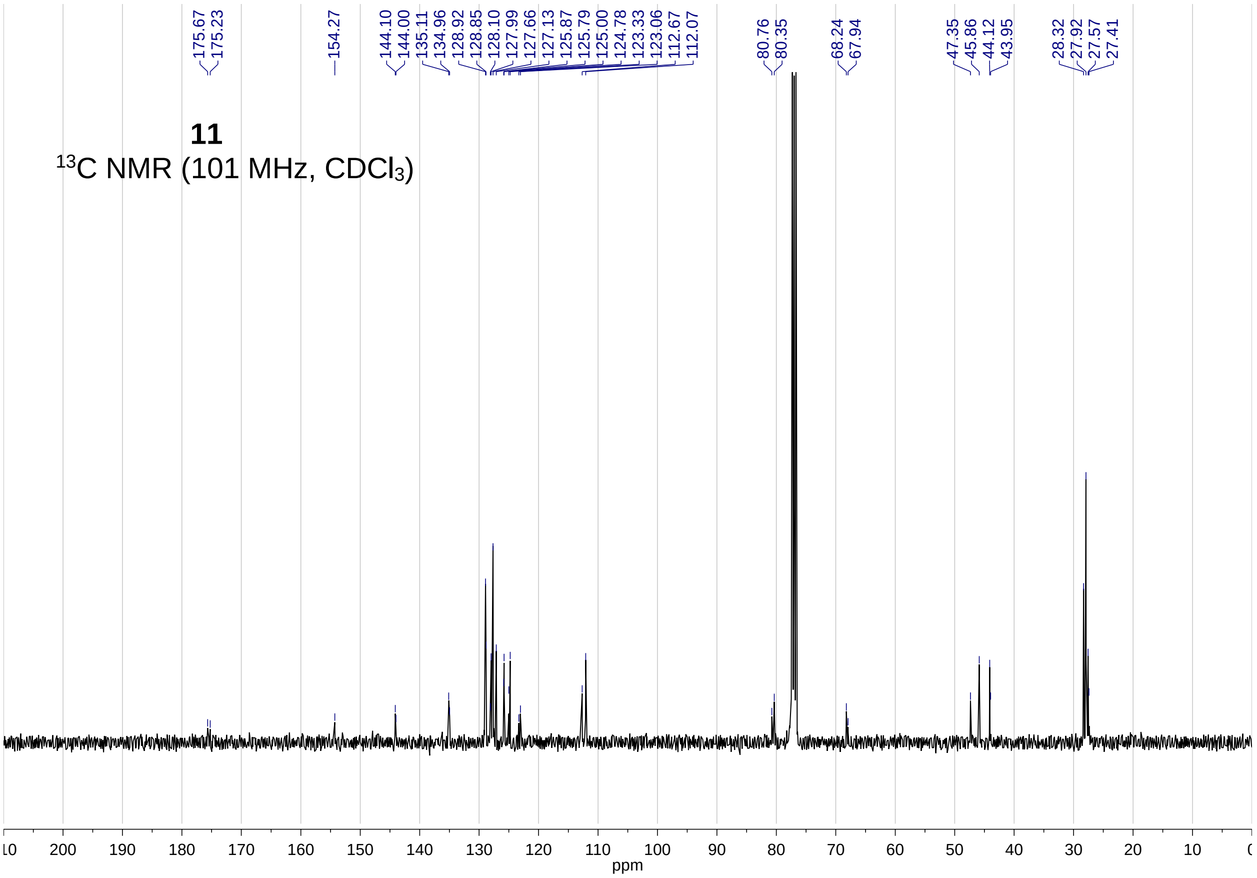

11

^{13}C NMR (101 MHz, $CDCl_3$)

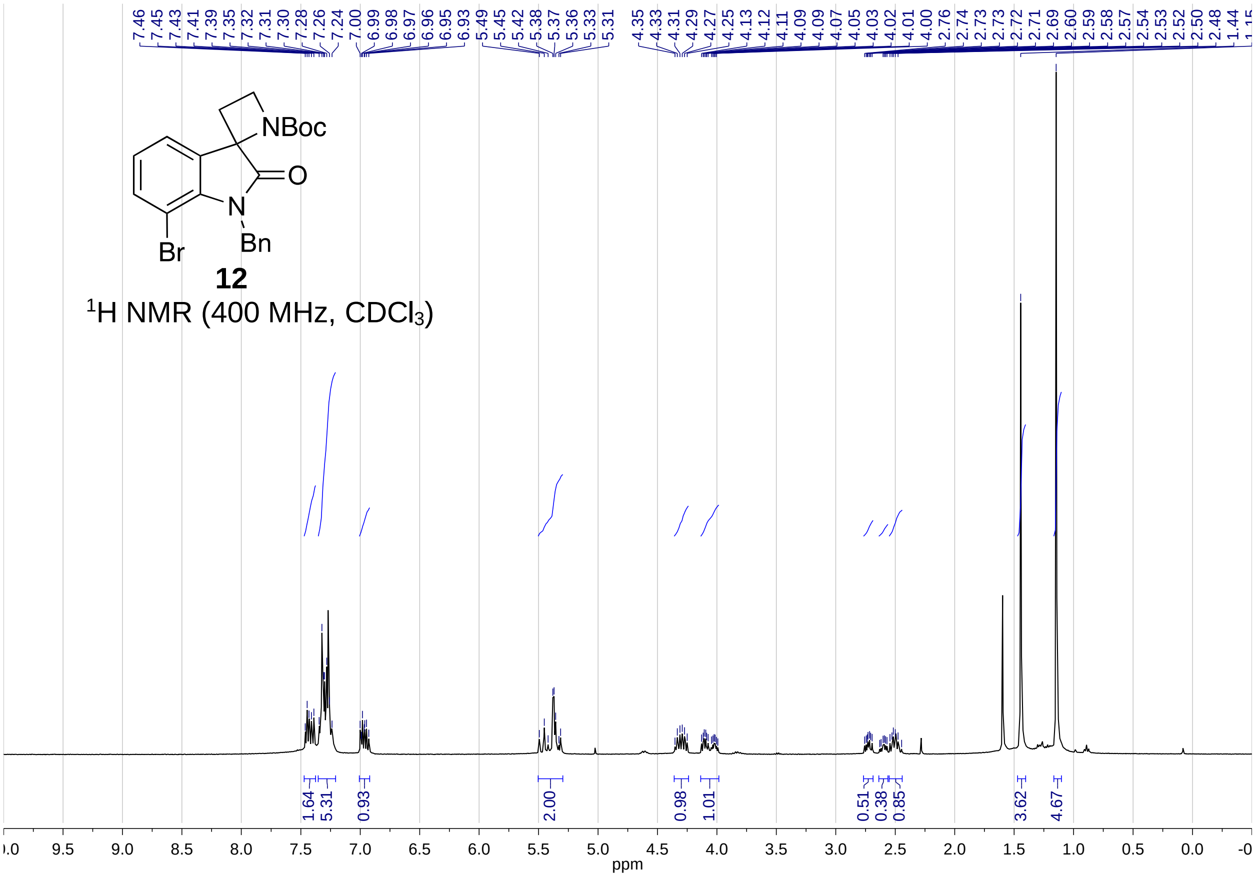

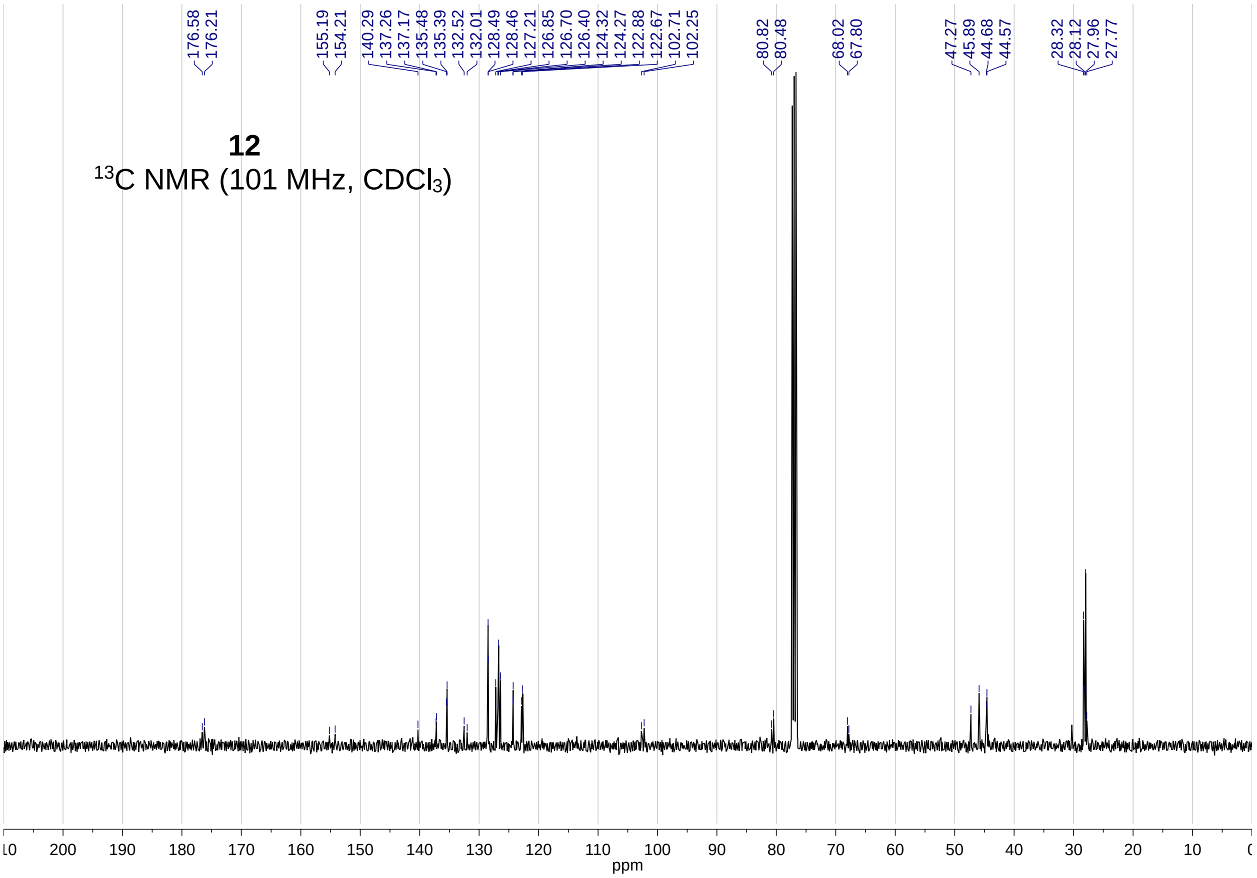

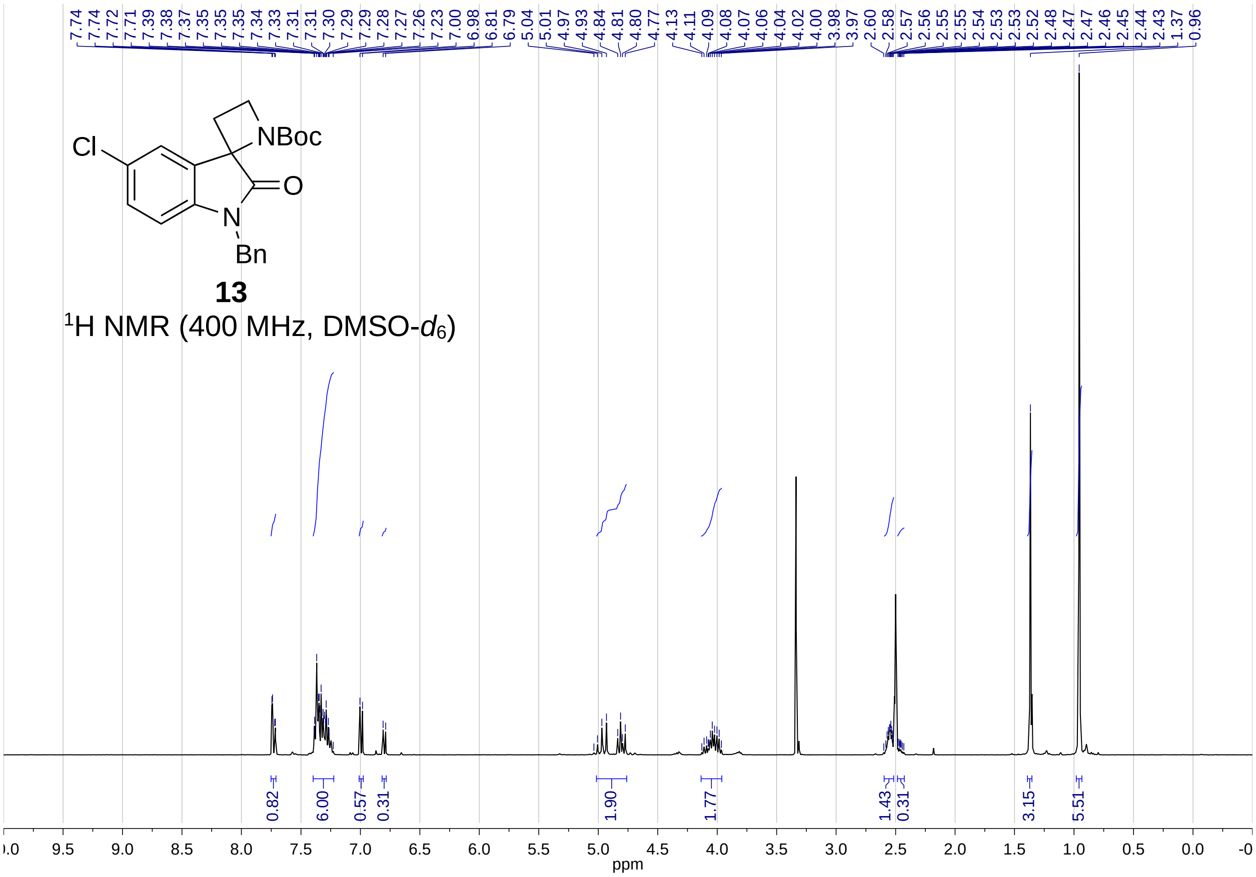
7.74 7.74 7.72 7.71 7.39 7.38 7.37 7.35 7.35 7.35 7.34 7.33 7.31 7.31 7.30 7.29 7.29 7.28 7.27 7.26 7.23 7.00 6.98 6.81 6.79 5.04 5.01 4.97 4.93 4.84 4.81 4.80 4.77 4.13 4.11 4.09 4.08 4.07 4.06 4.04 4.02 4.00 3.98 3.97 2.60 2.58 2.57 2.56 2.55 2.55 2.54 2.53 2.53 2.52 2.48 2.47 2.47 2.46 2.45 2.44 2.43 1.37 0.96
Cl
NBoc
O
N
Bn
13
^{1}H NMR (400 MHz, DMSO-d_6)
0.82 6.00 0.57 0.31 1.90 1.77 1.43 0.31 3.15 5.51
ppm

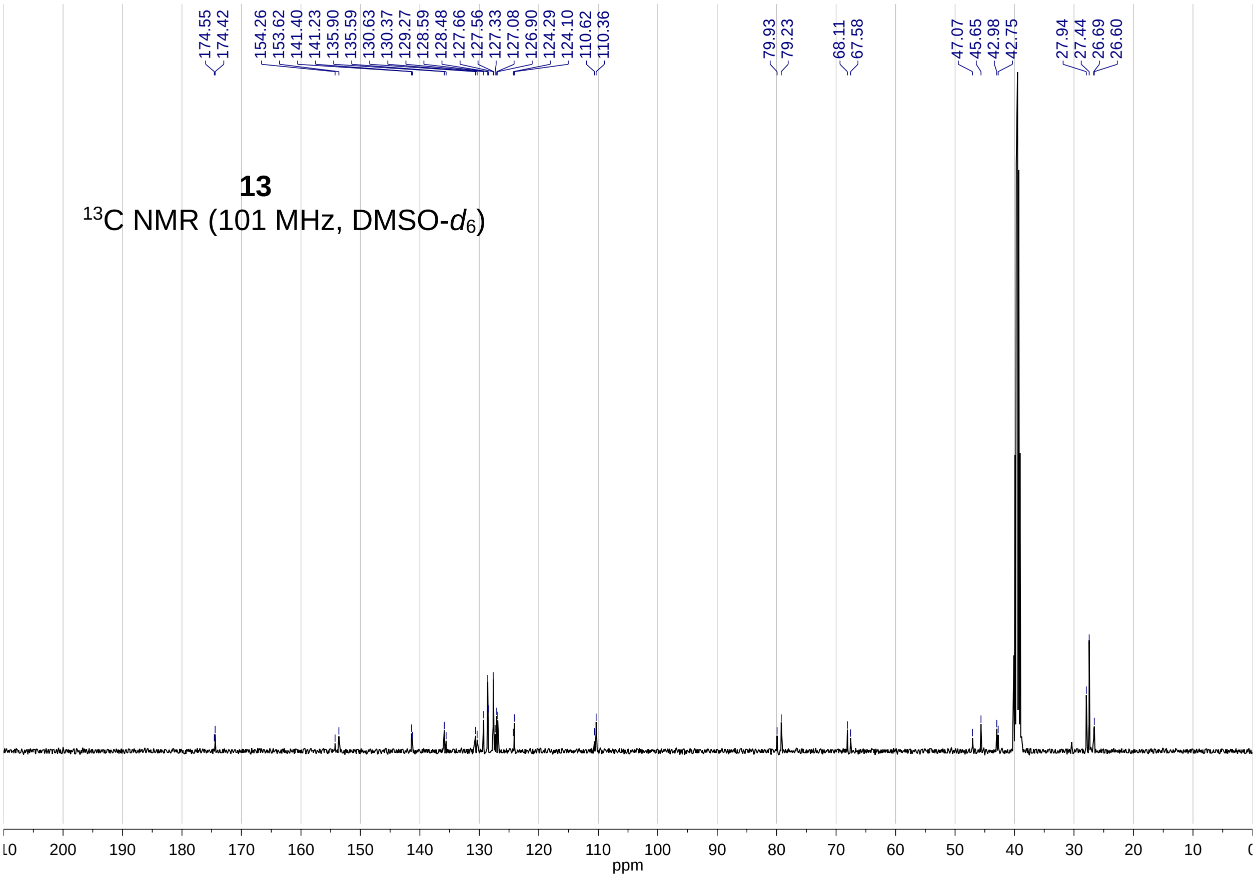
174.55 174.42 154.26 153.62 141.40 141.23 135.90 135.59 130.63 130.37 129.27 128.59 128.48 127.66 127.56 127.33 127.08 126.90 124.29 124.10 110.62 110.36 79.93 79.23 68.11 67.58 47.07 45.65 42.98 42.75 27.94 27.44 26.69 26.60
13
^{13}C NMR (101 MHz, DMSO-d_6)
ppm

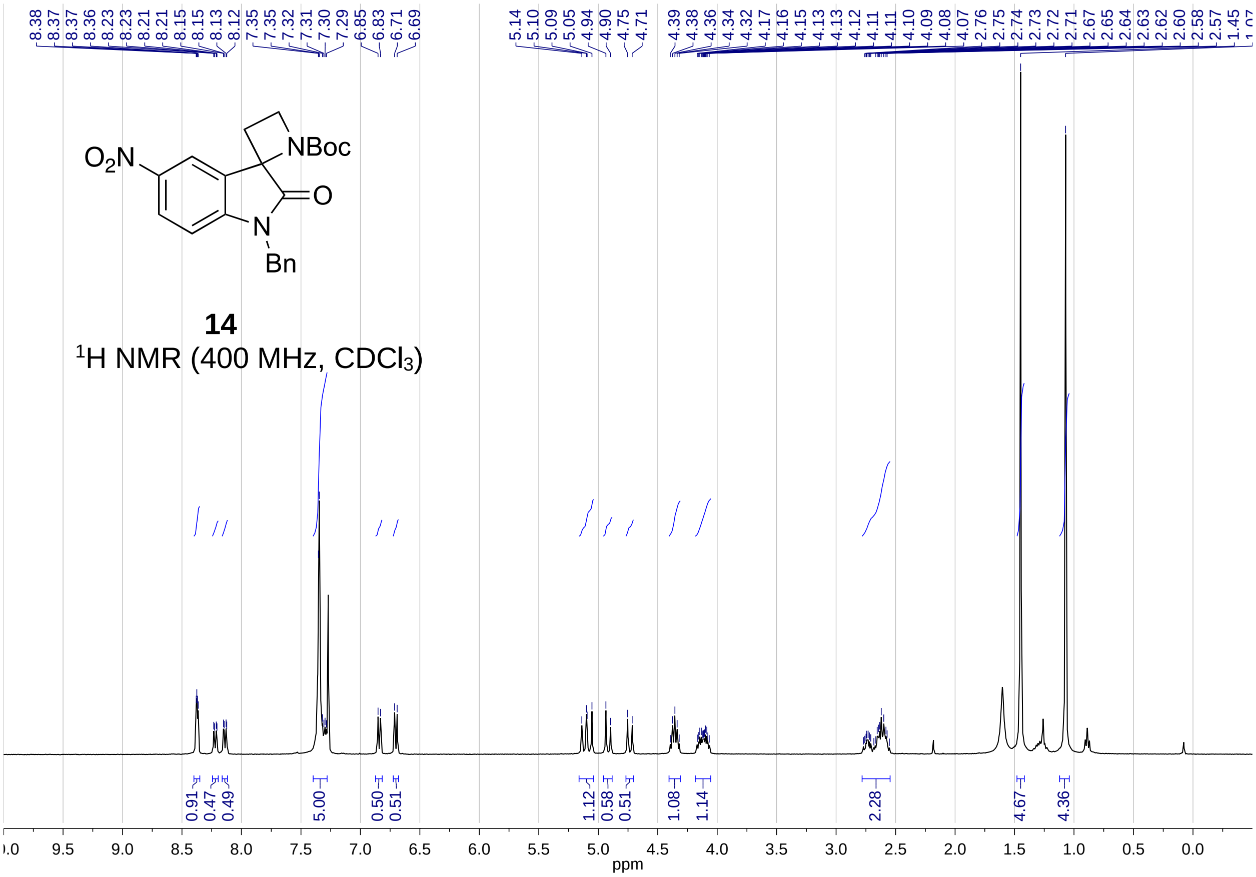

14

^{1}H NMR (400 MHz, $CDCl_3$)

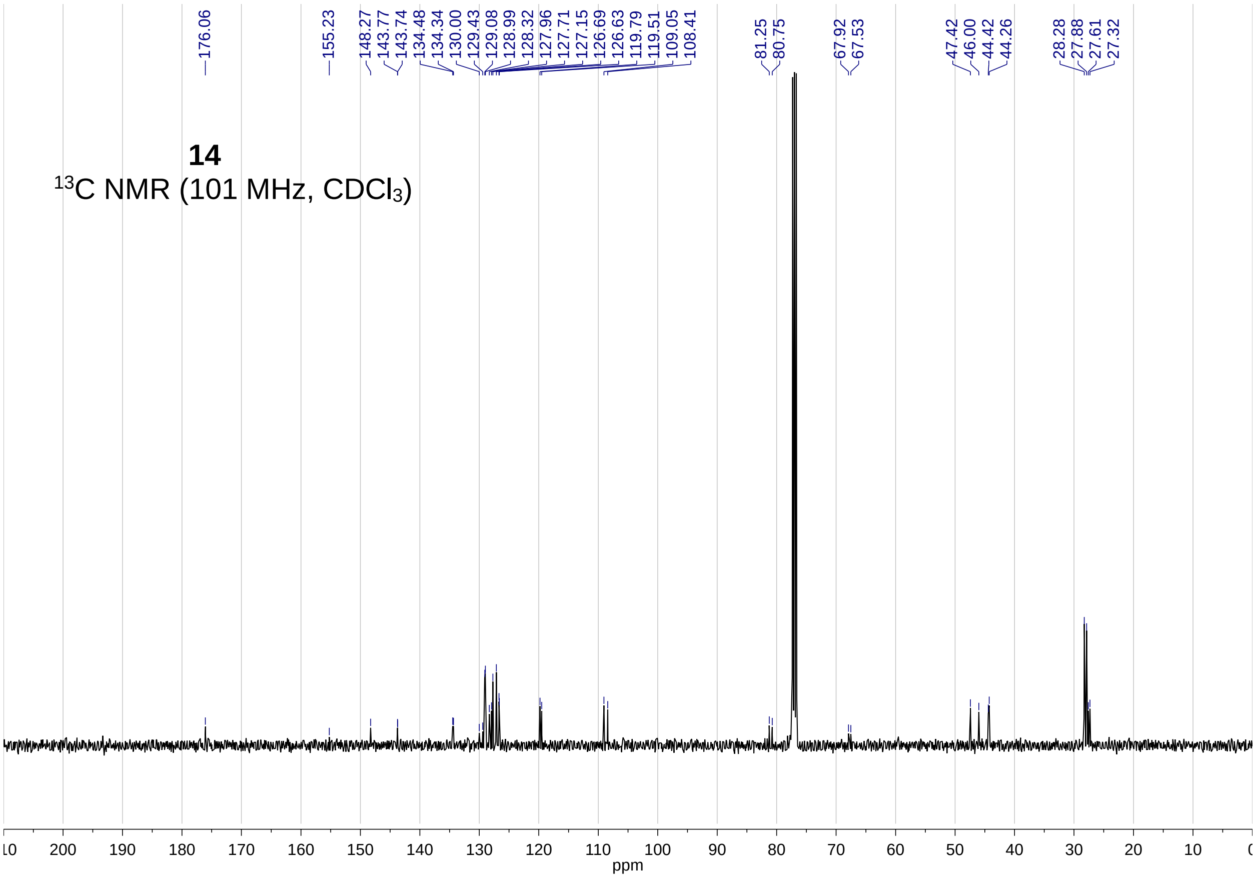

14

^{13}C NMR (101 MHz, $CDCl_3$)

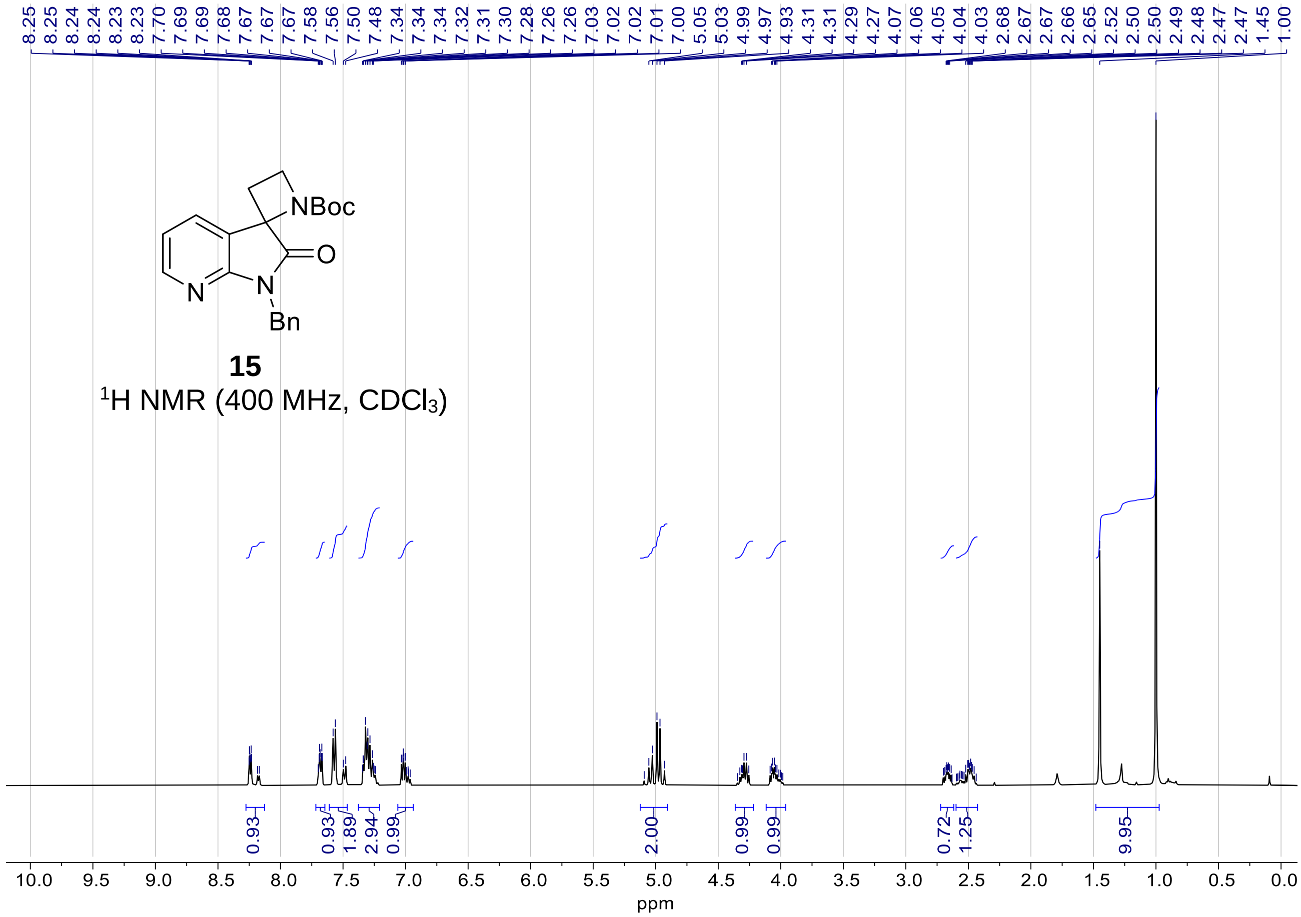

15

^{1}H NMR (400 MHz, $CDCl_3$)

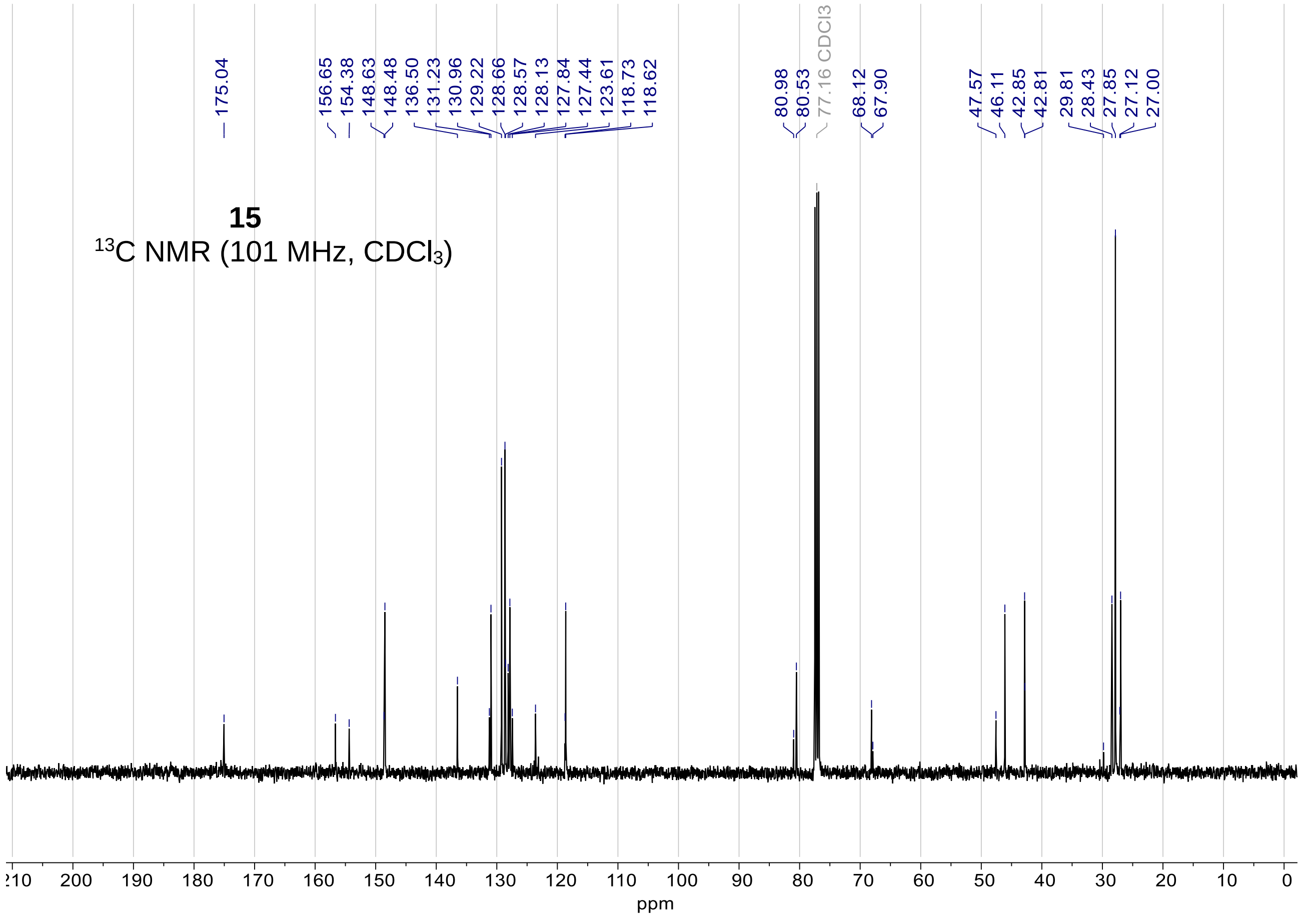

15

^{13}C NMR (101 MHz, $CDCl_3$)

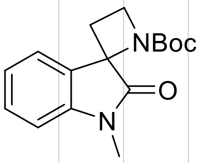

16

^{1}H NMR (400 MHz, DMSO-d_6)

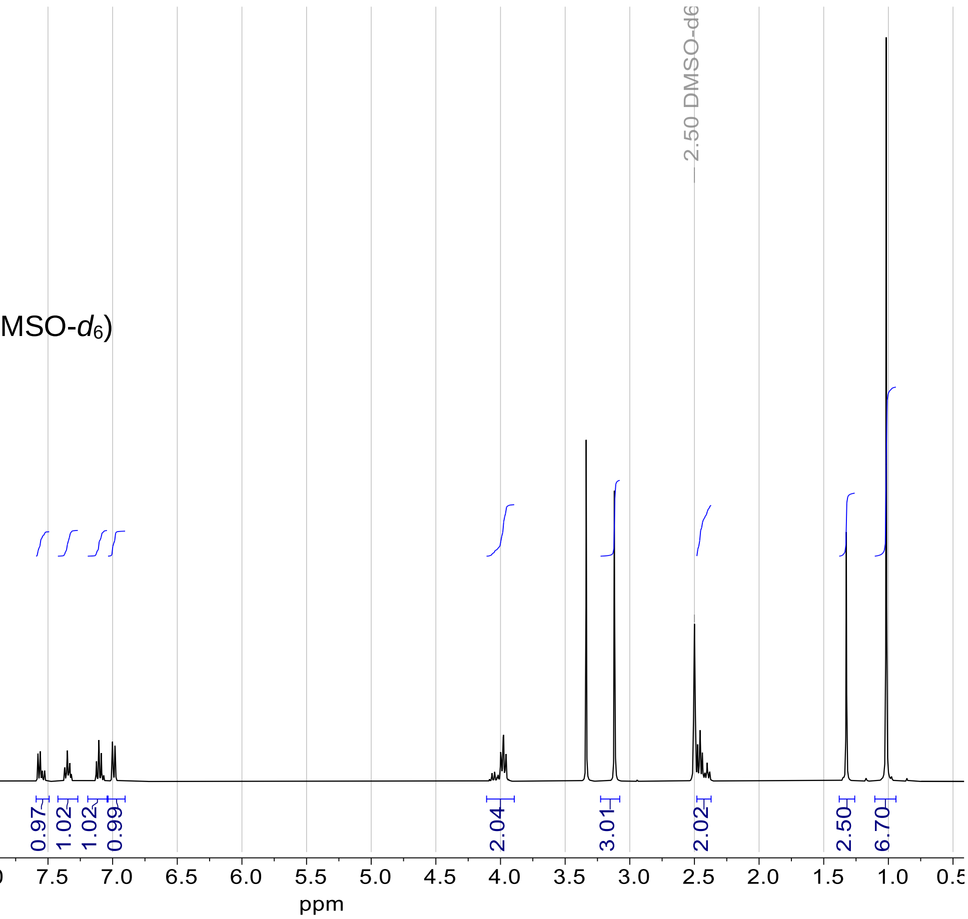

16

^{13}C NMR (400 MHz, DMSO-d_6)

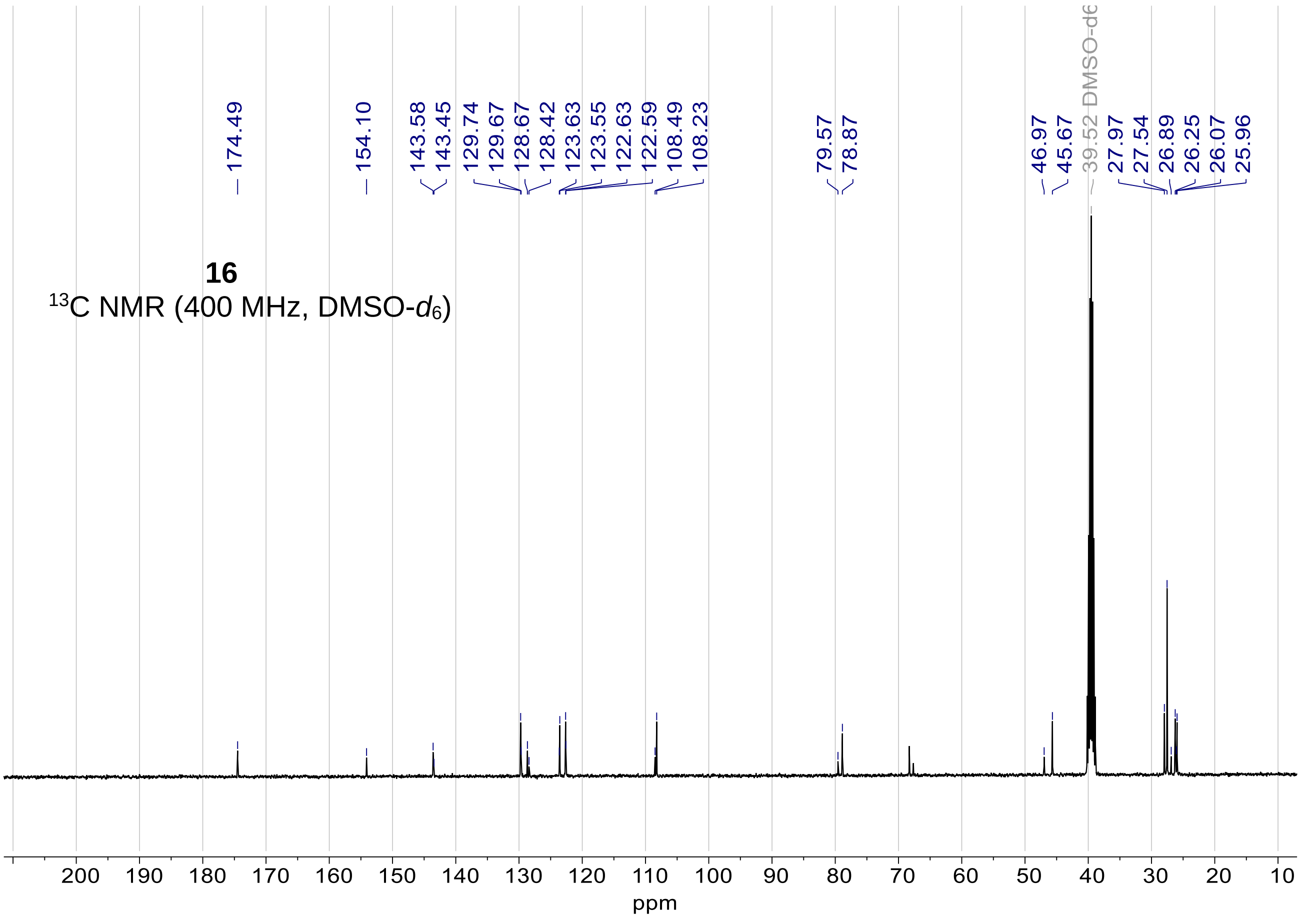

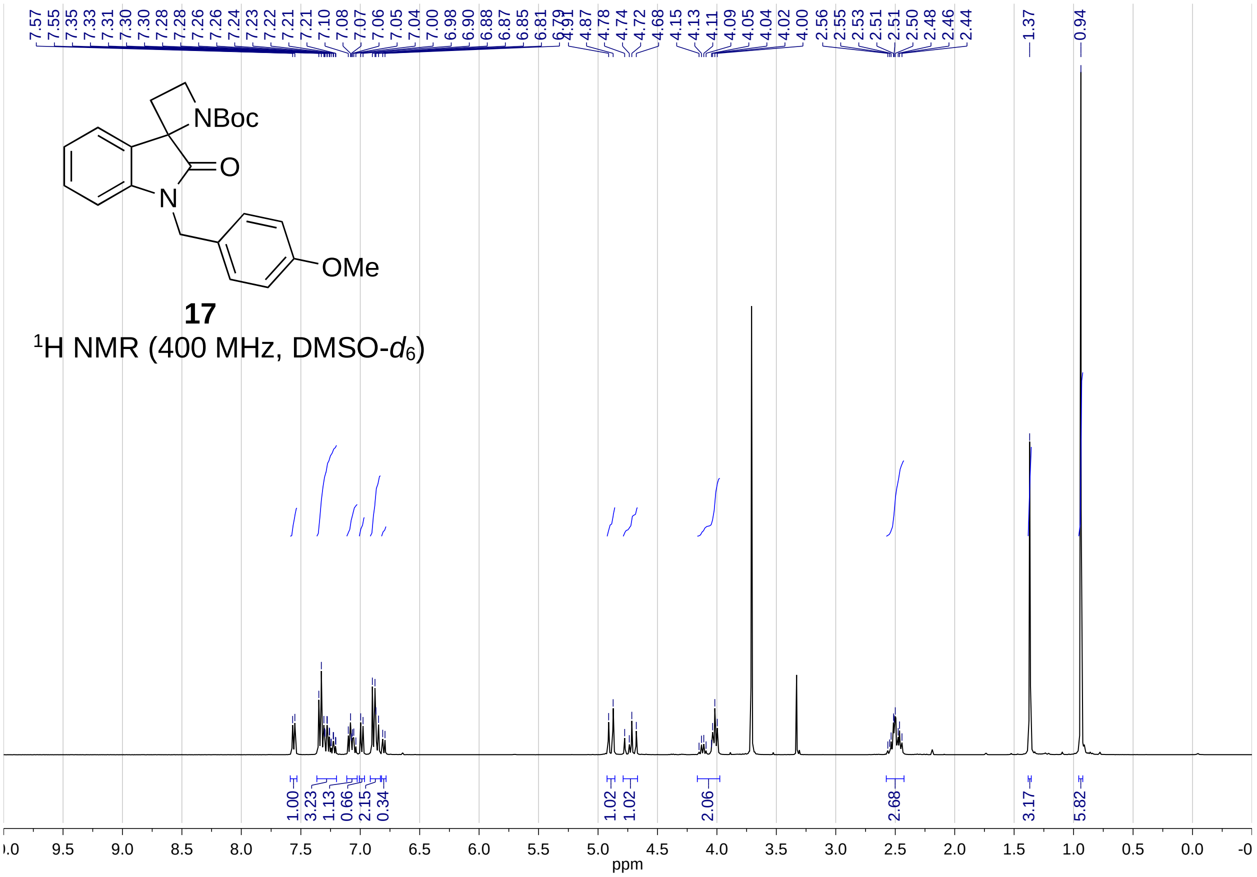
7.57 7.55 7.35 7.33 7.31 7.30 7.30 7.28 7.26 7.26 7.24 7.24 7.23 7.22 7.21 7.21 7.10 7.08 7.07 7.06 7.05 7.04 7.00 6.98 6.90 6.88 6.87 6.85 6.81 6.79 4.91 4.87 4.78 4.74 4.72 4.68 4.15 4.13 4.11 4.09 4.05 4.04 4.02 4.00 2.56 2.55 2.53 2.51 2.51 2.50 2.48 2.46 2.44 1.37 0.94
NBoc
O
N
OMe
17
1H NMR (400 MHz, DMSO-d6)
1.00 3.23 1.13 0.66 2.15 0.34 1.02 1.02 2.06 2.68 3.17 5.82
ppm

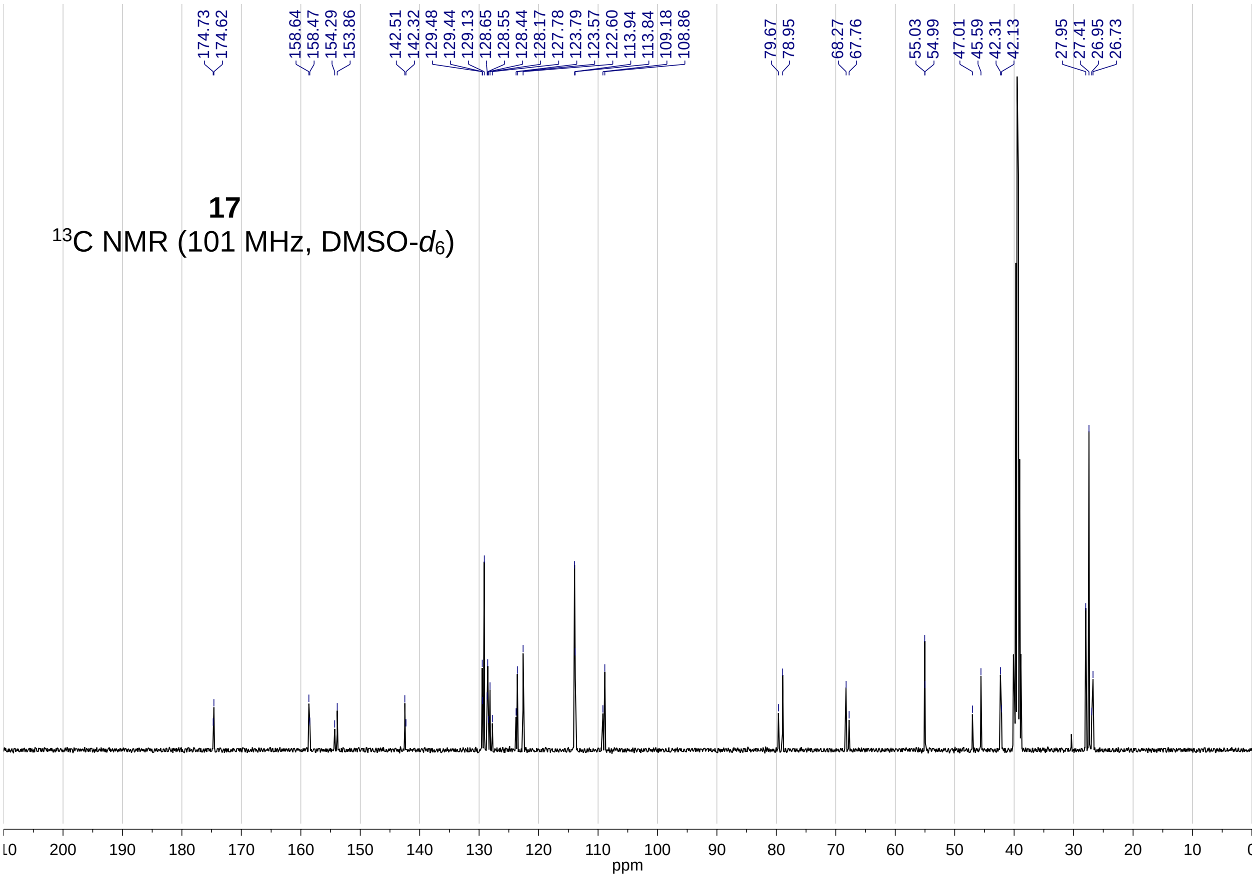
174.73 174.62 158.64 158.47 154.29 153.86 142.51 142.32 129.48 129.44 129.13 128.65 128.55 128.44 128.17 127.78 123.79 123.57 122.60 113.94 113.84 109.18 108.86 79.67 78.95 68.27 67.76 55.03 54.99 47.01 45.59 42.31 42.13 27.95 27.41 26.95 26.73
17
13C NMR (101 MHz, DMSO-d6)
ppm

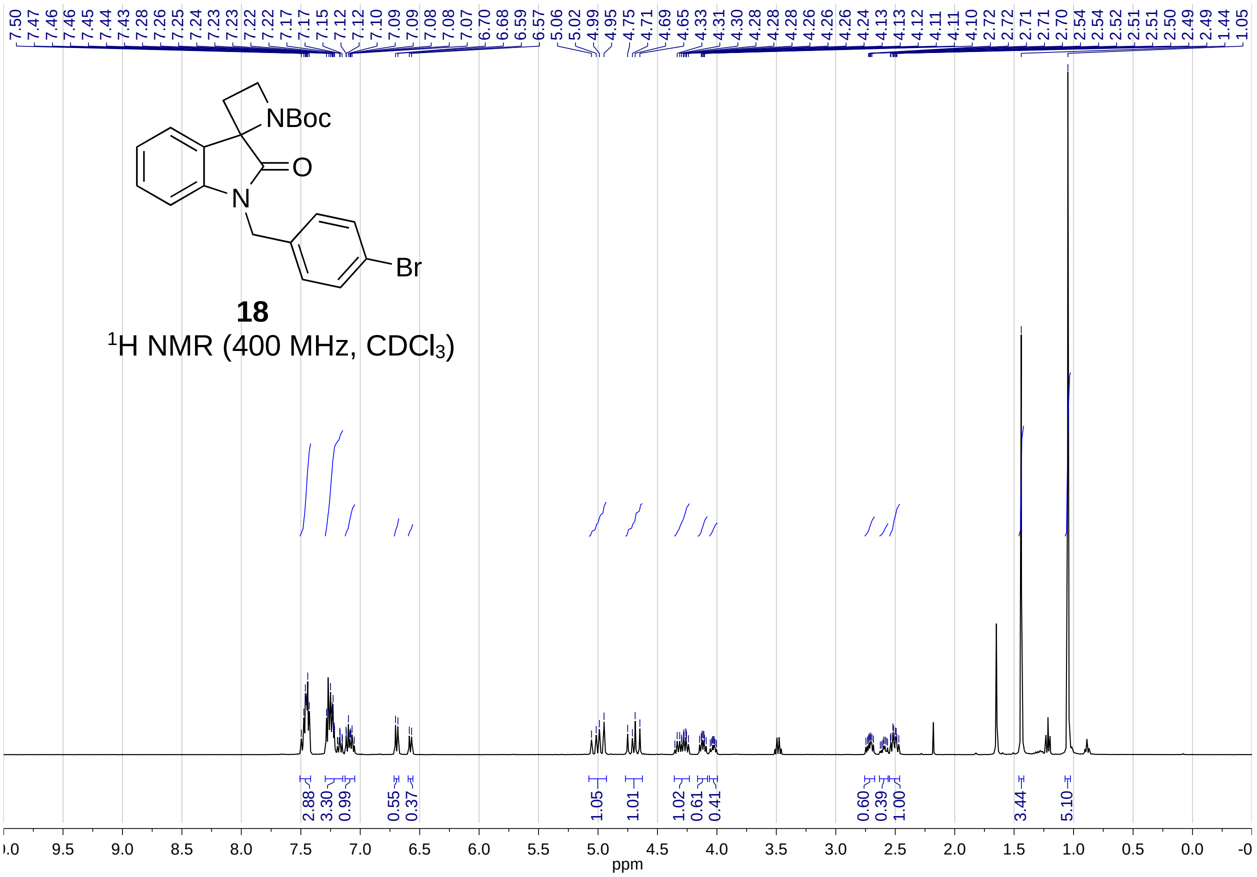
7.50 7.47 7.46 7.45 7.44 7.43 7.28 7.26 7.25 7.24 7.23 7.23 7.22 7.22 7.17 7.17 7.15 7.12 7.12 7.10 7.09 7.09 7.08 7.08 7.07 6.70 6.68 6.59 6.57 5.06 5.02 4.99 4.95 4.75 4.71 4.69 4.65 4.33 4.31 4.30 4.28 4.28 4.28 4.26 4.26 4.26 4.24 4.13 4.13 4.12 4.11 4.11 4.10 2.72 2.72 2.71 2.71 2.70 2.54 2.54 2.52 2.51 2.50 2.49 1.44 1.05
NBoc
O
N
Br
18
¹H NMR (400 MHz, CDCl₃)
2.88 3.30 0.99 0.55 0.37 1.05 1.01 1.02 0.61 0.41 0.60 0.39 1.00 3.44 5.10
ppm

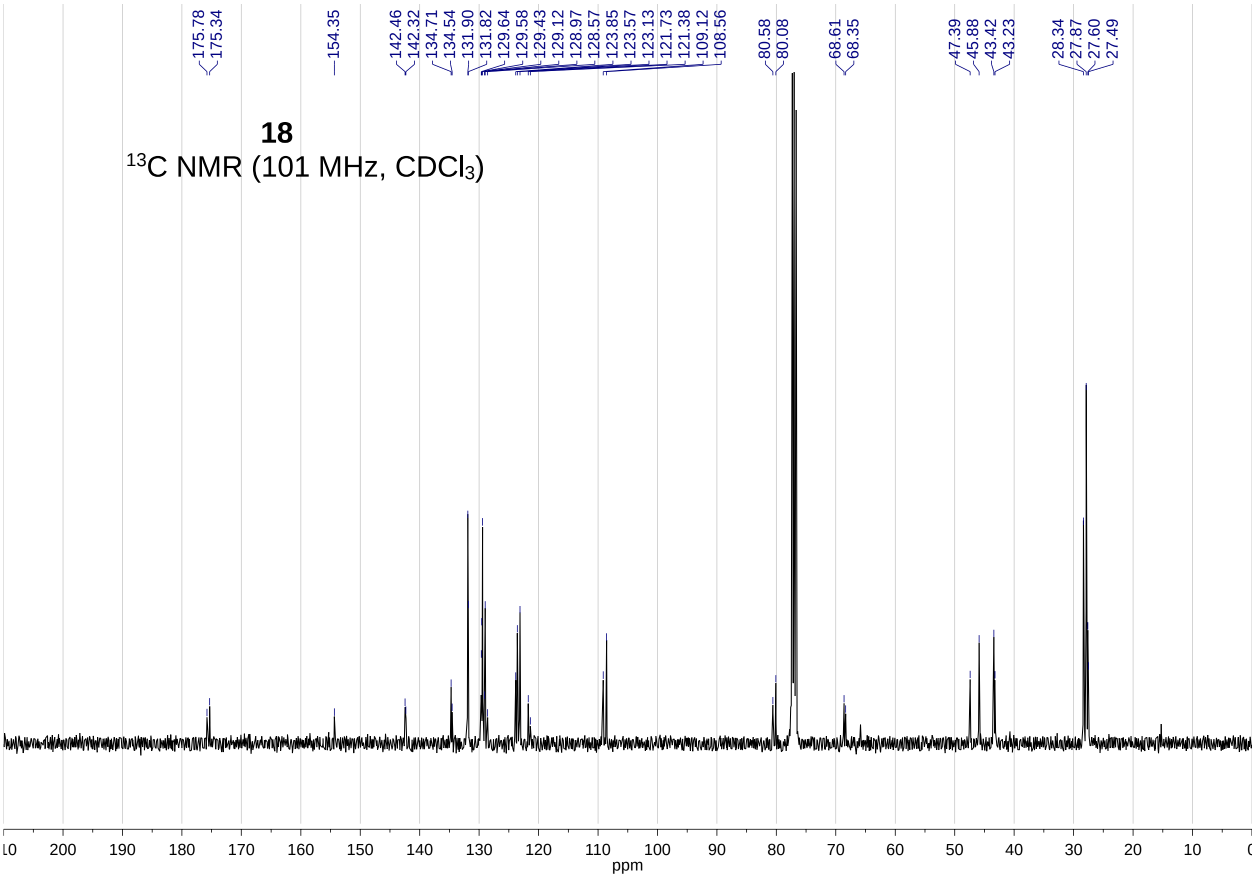
175.78 175.34 154.35 142.46 142.32 134.71 134.54 131.90 131.82 129.64 129.58 129.43 129.12 128.97 128.57 123.85 123.57 123.13 121.73 121.38 109.12 108.56 80.58 80.08 68.61 68.35 47.39 45.88 43.42 43.23 28.34 27.87 27.60 27.49
18
¹³C NMR (101 MHz, CDCl₃)
ppm

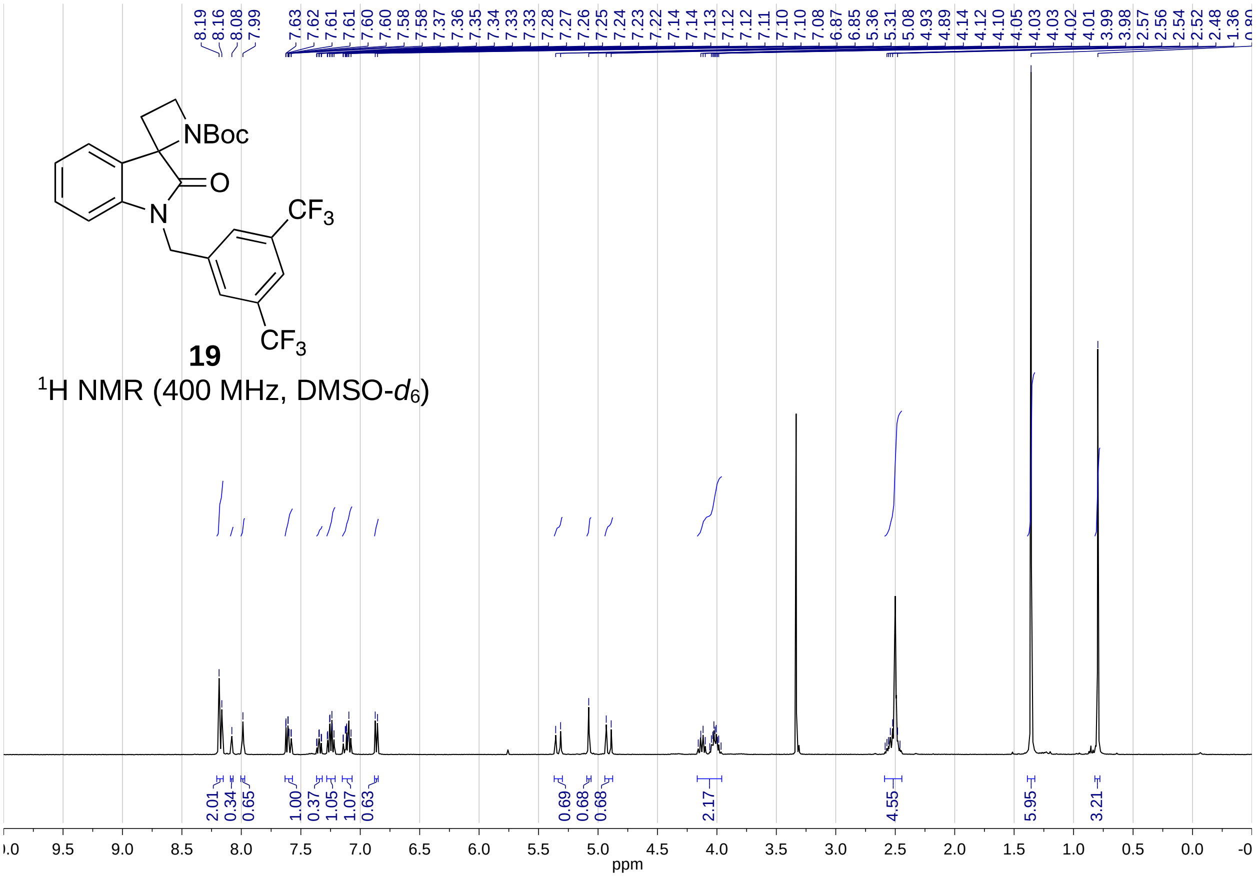

19

^{1}H NMR (400 MHz, DMSO-d_6)

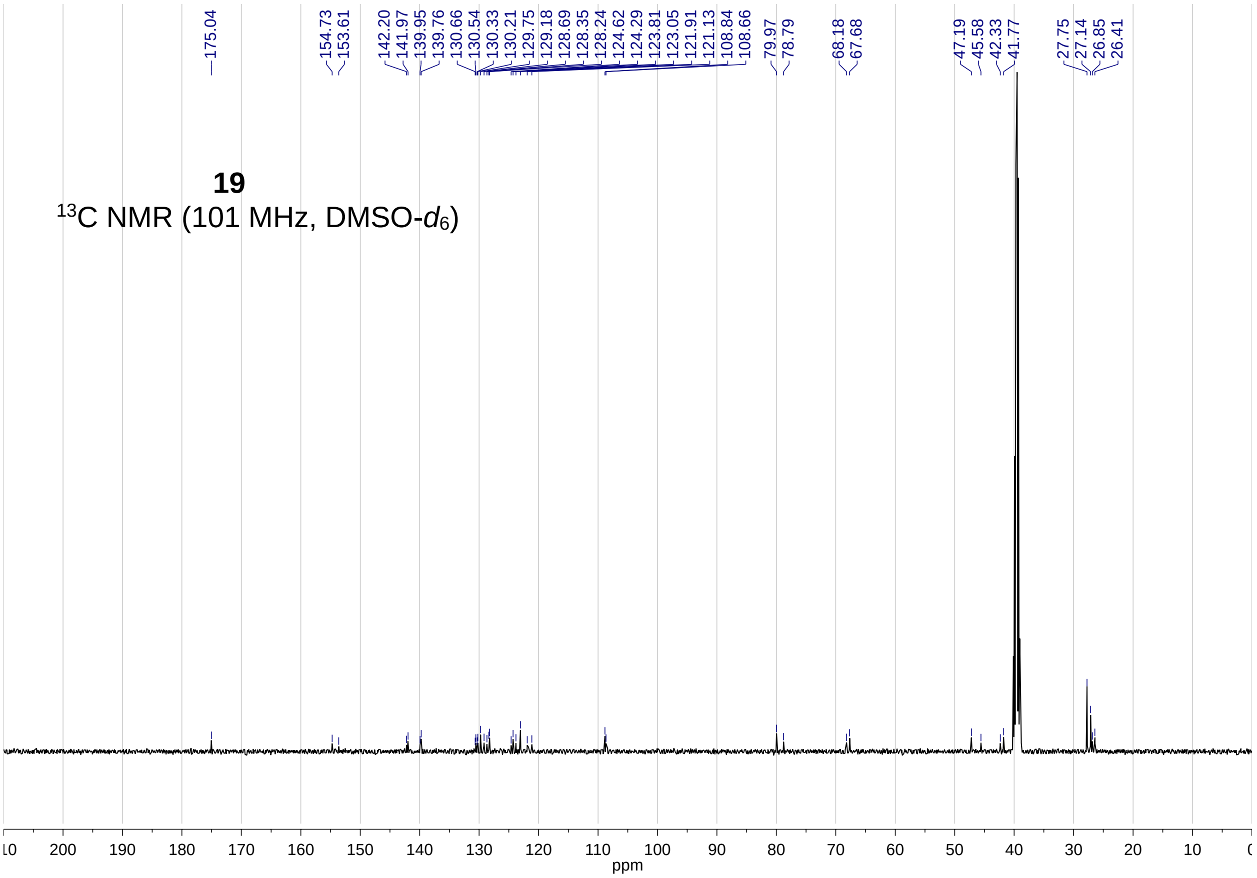

19

^{13}C NMR (101 MHz, DMSO-d_6)

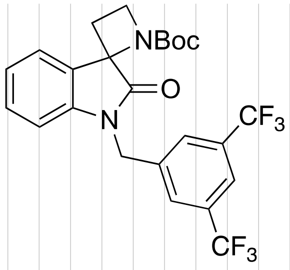

19

$^{19}F\{^{1}H\}$ NMR (377 MHz, DMSO-d_6)

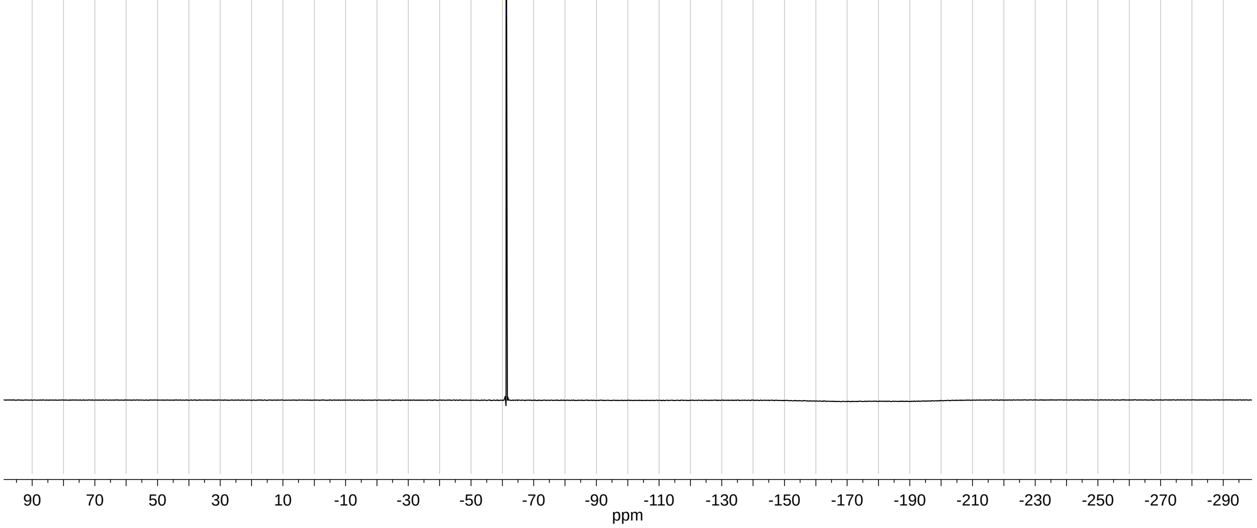

20

^{1}H NMR (400 MHz, DMSO-d_6)

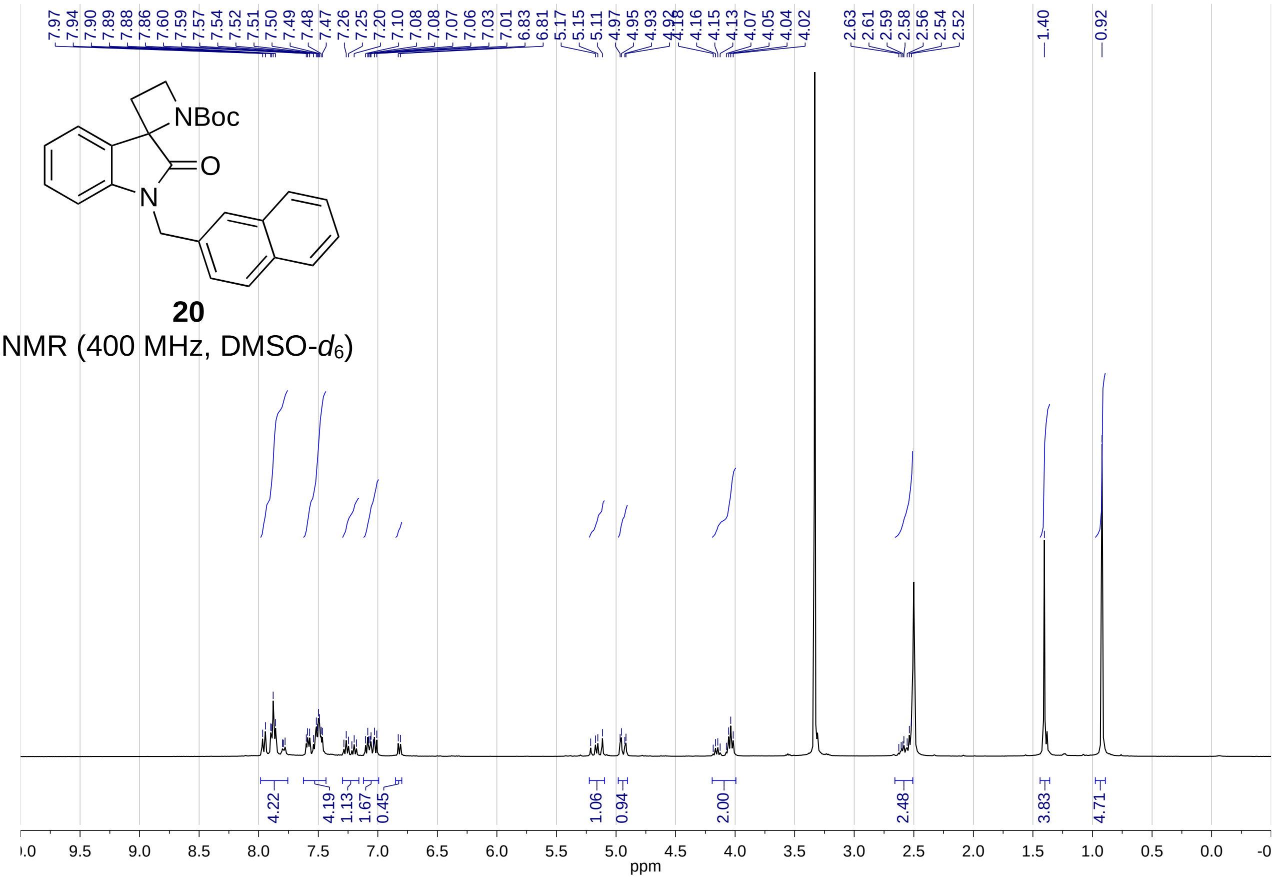

20

^{13}C NMR (101 MHz, DMSO-d_6)

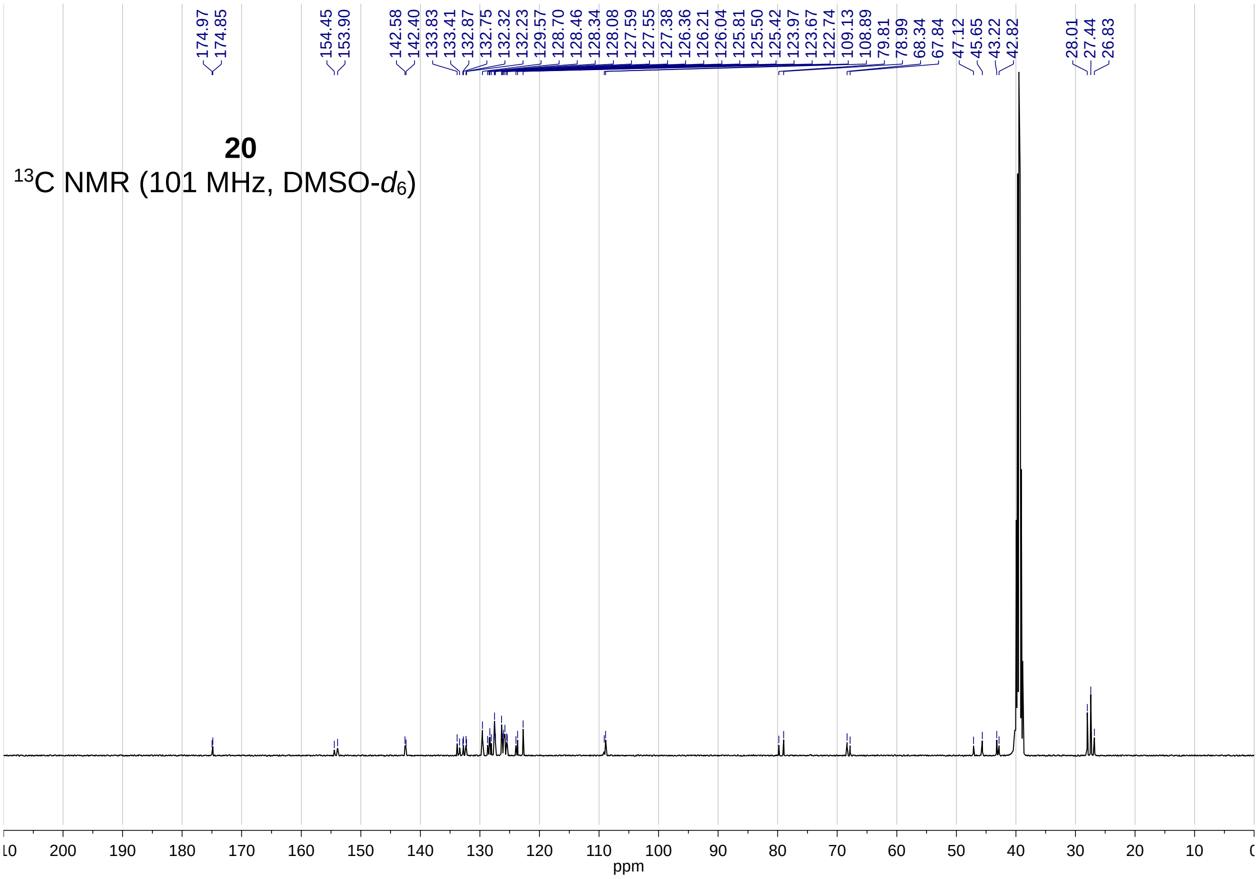

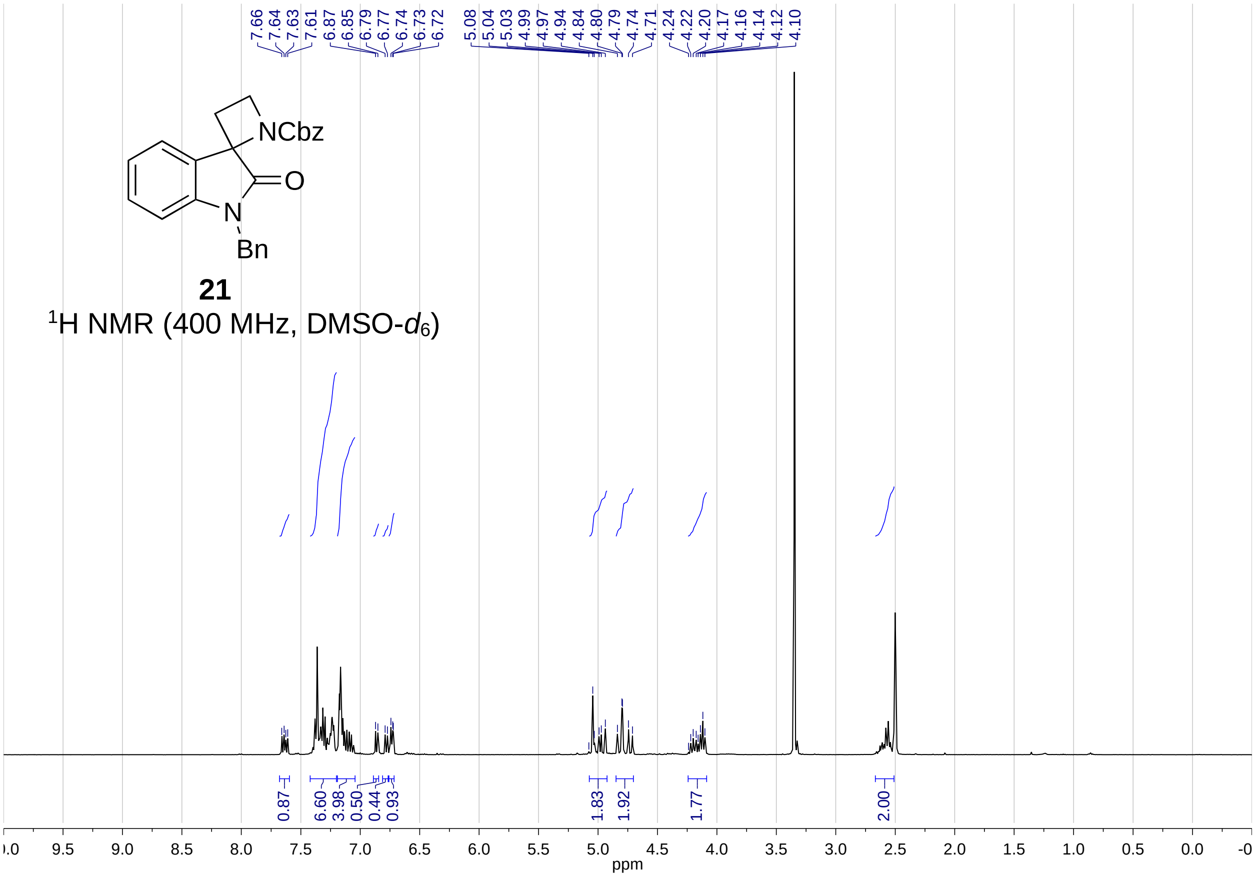

21

^{1}H NMR (400 MHz, DMSO-d_6)

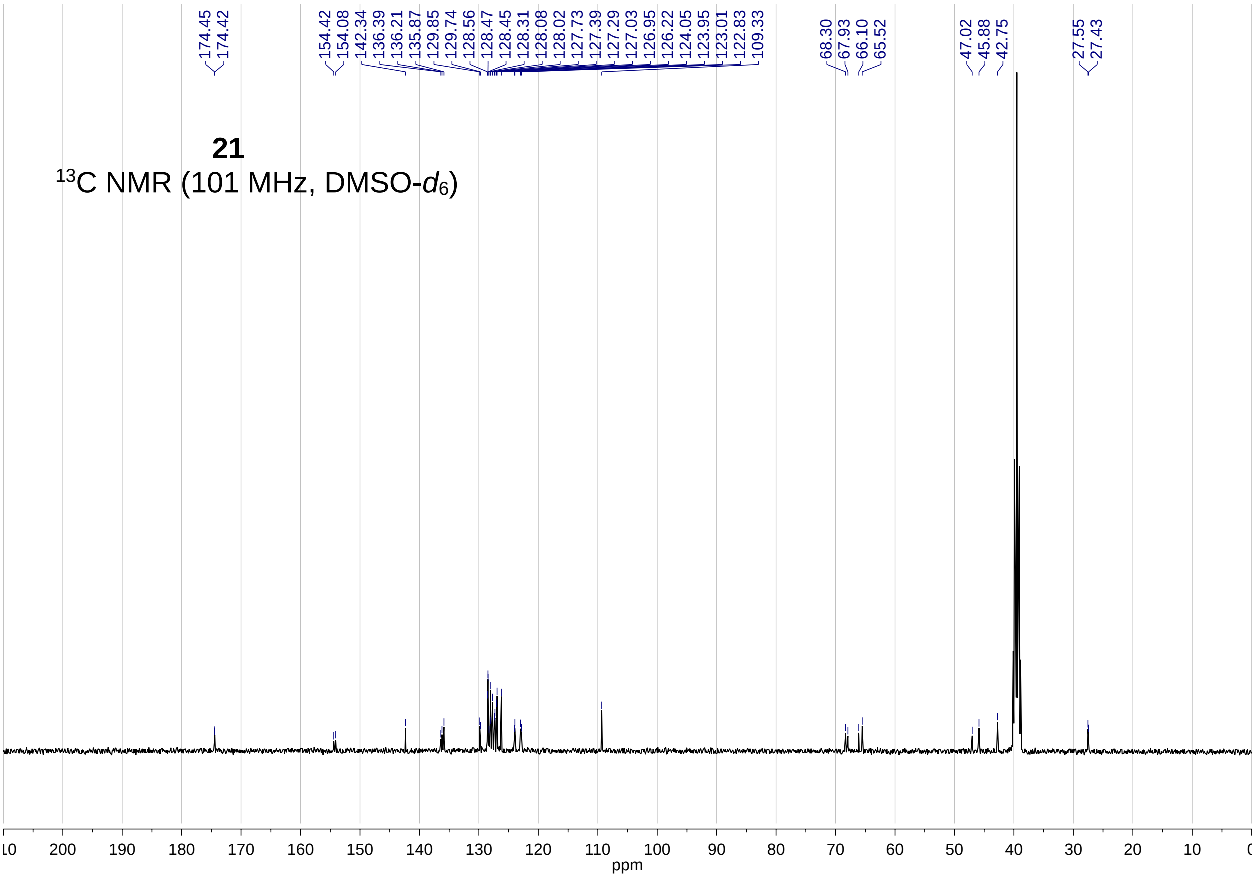

21

^{13}C NMR (101 MHz, DMSO-d_6)

22

^{1}H NMR (400 MHz, $CDCl_3$)

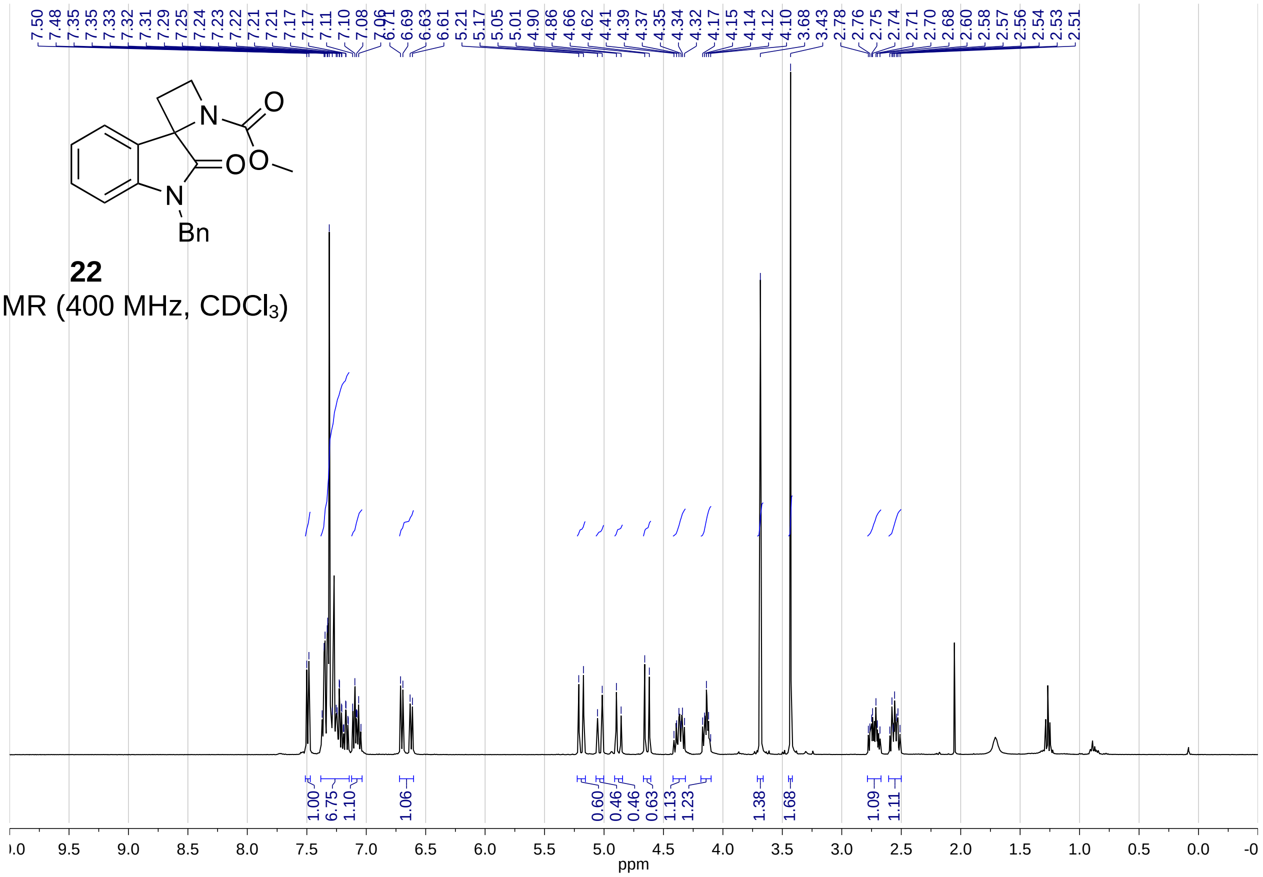

22

^{13}C NMR (101 MHz, $CDCl_3$)

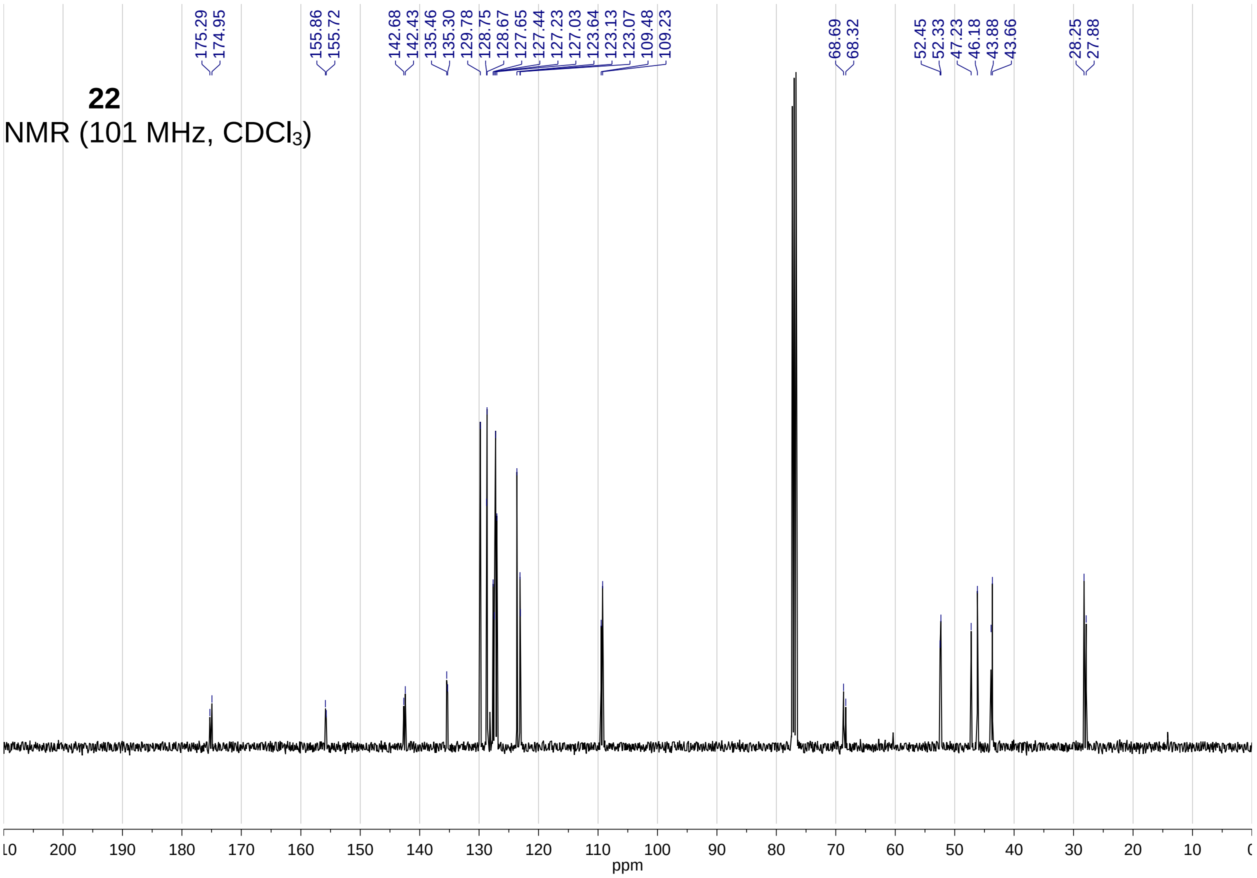

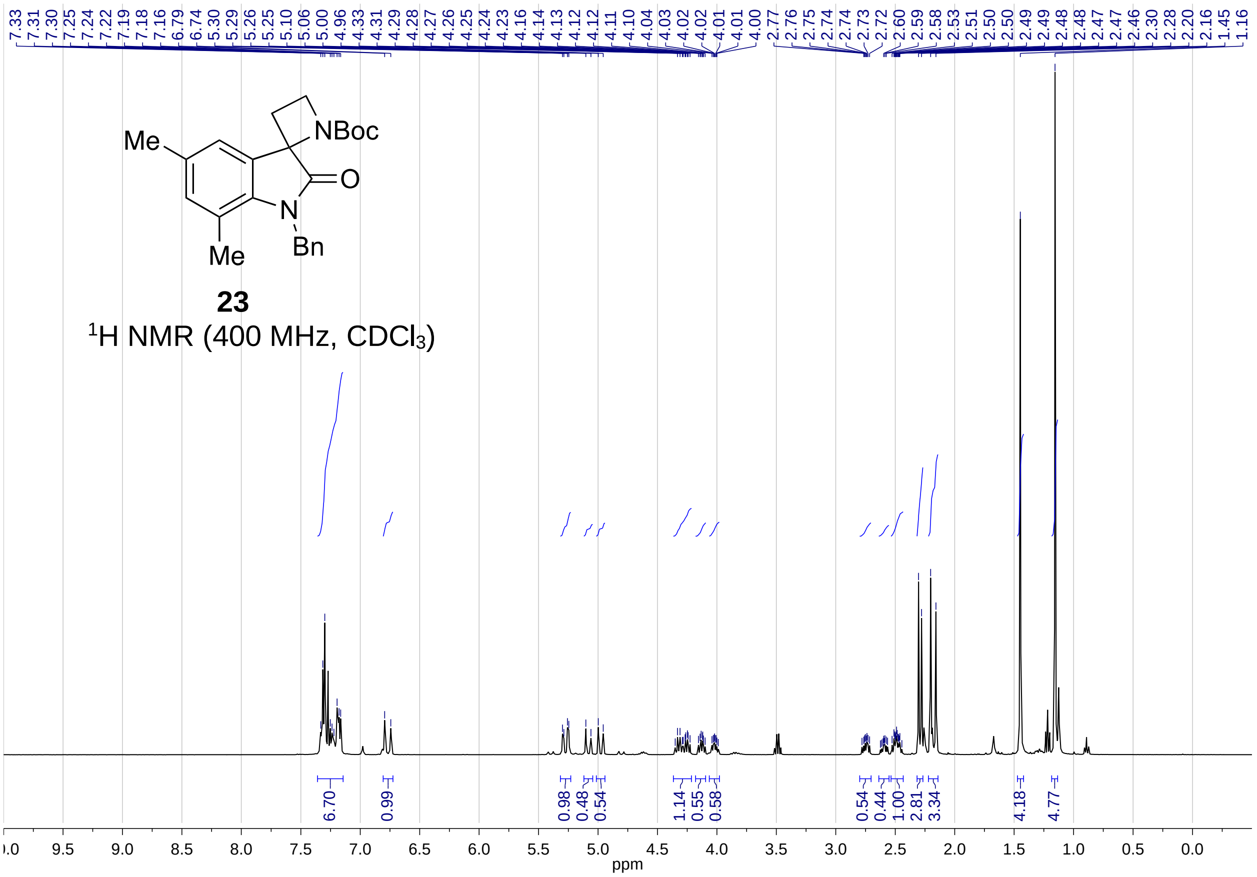

23

^{1}H NMR (400 MHz, $CDCl_3$)

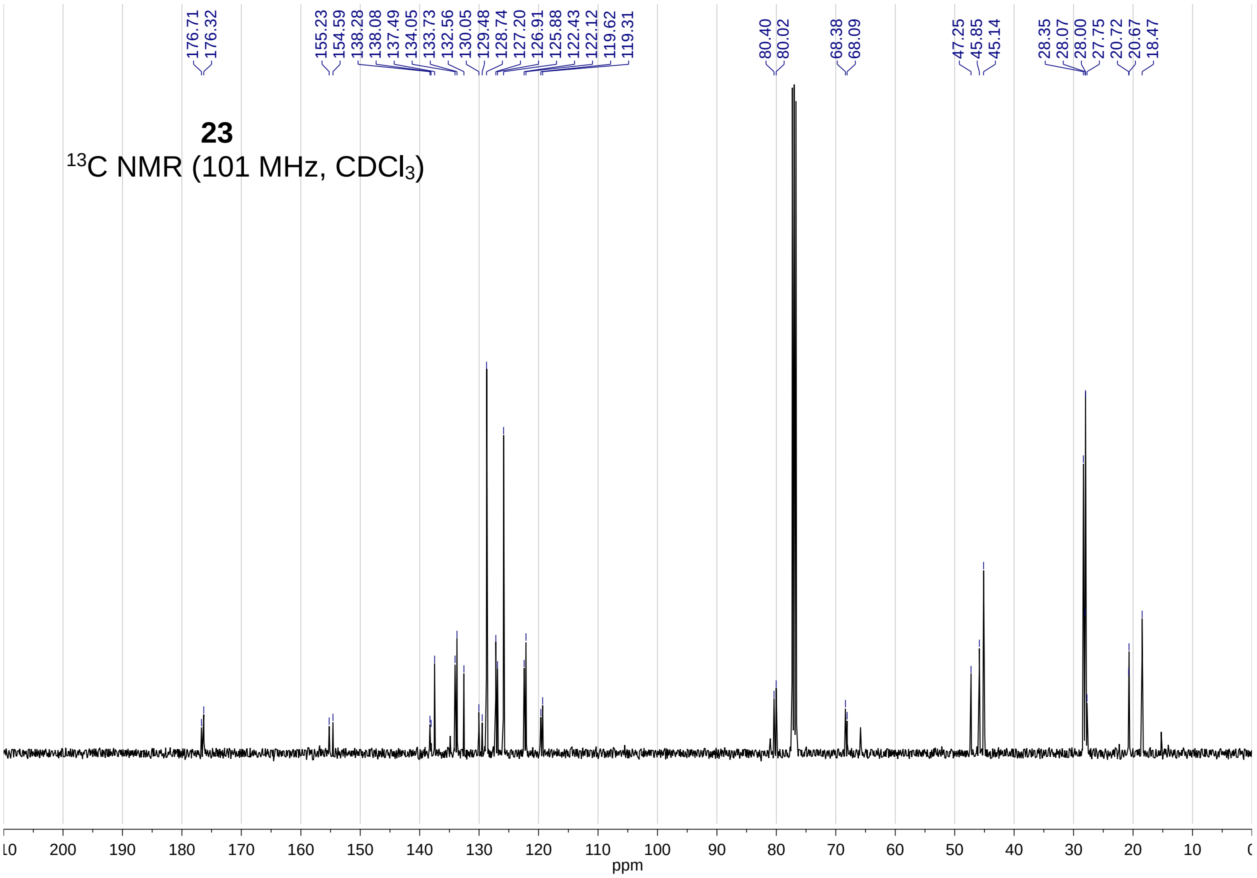

23

^{13}C NMR (101 MHz, $CDCl_3$)

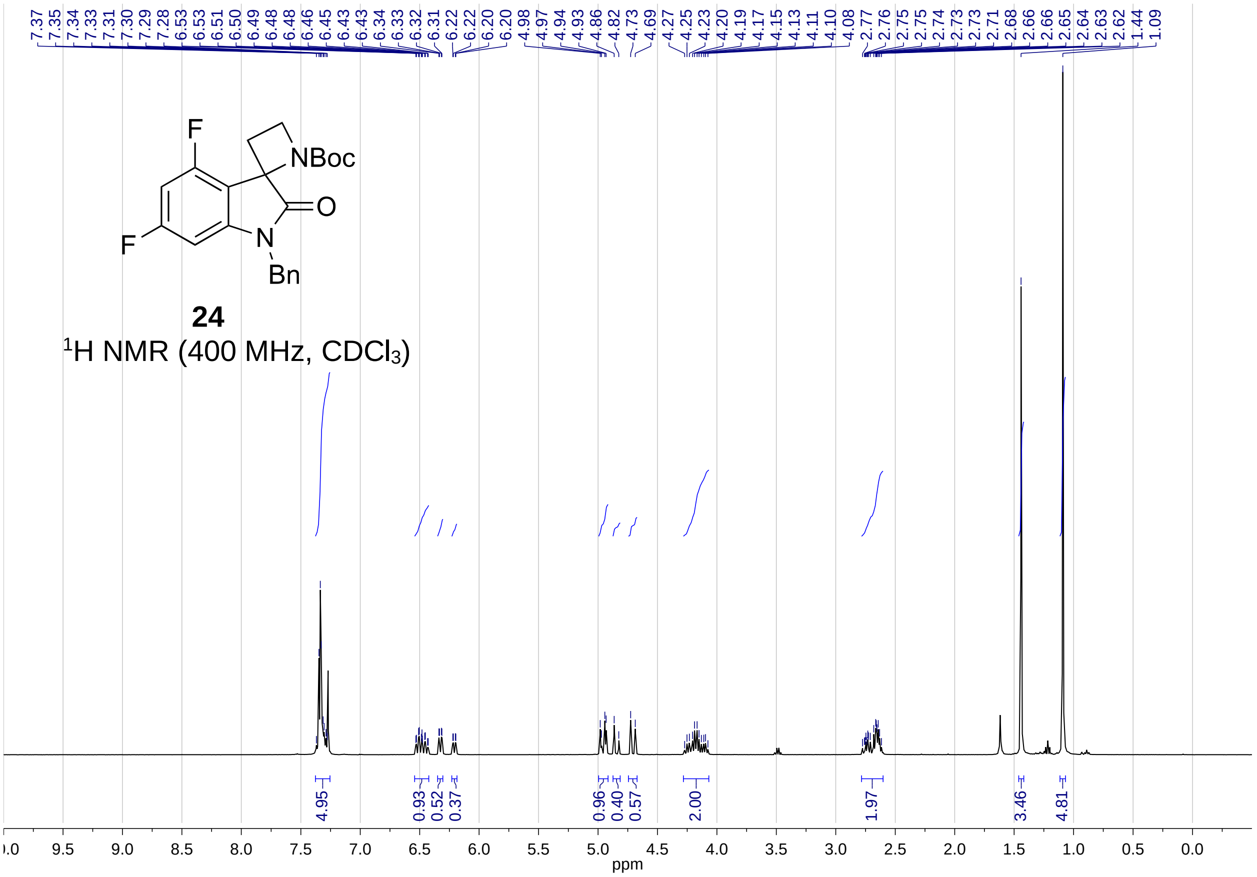
7.37 7.35 7.34 7.33 7.31 7.30 7.29 7.28 6.53 6.53 6.51 6.50 6.49 6.48 6.48 6.46 6.45 6.43 6.43 6.34 6.33 6.32 6.31 6.22 6.22 6.20 6.20 4.98 4.97 4.94 4.93 4.86 4.82 4.73 4.69 4.27 4.25 4.23 4.20 4.19 4.17 4.15 4.13 4.11 4.10 4.08 2.77 2.76 2.75 2.75 2.74 2.73 2.73 2.71 2.68 2.66 2.65 2.64 2.63 2.62 1.44 1.09
F
NBoc
O
F
N
Bn
24
^{1}H NMR (400 MHz, $CDCl_3$)
4.95 0.93 0.52 0.37 0.96 0.40 0.57 2.00 1.97 3.46 4.81
ppm

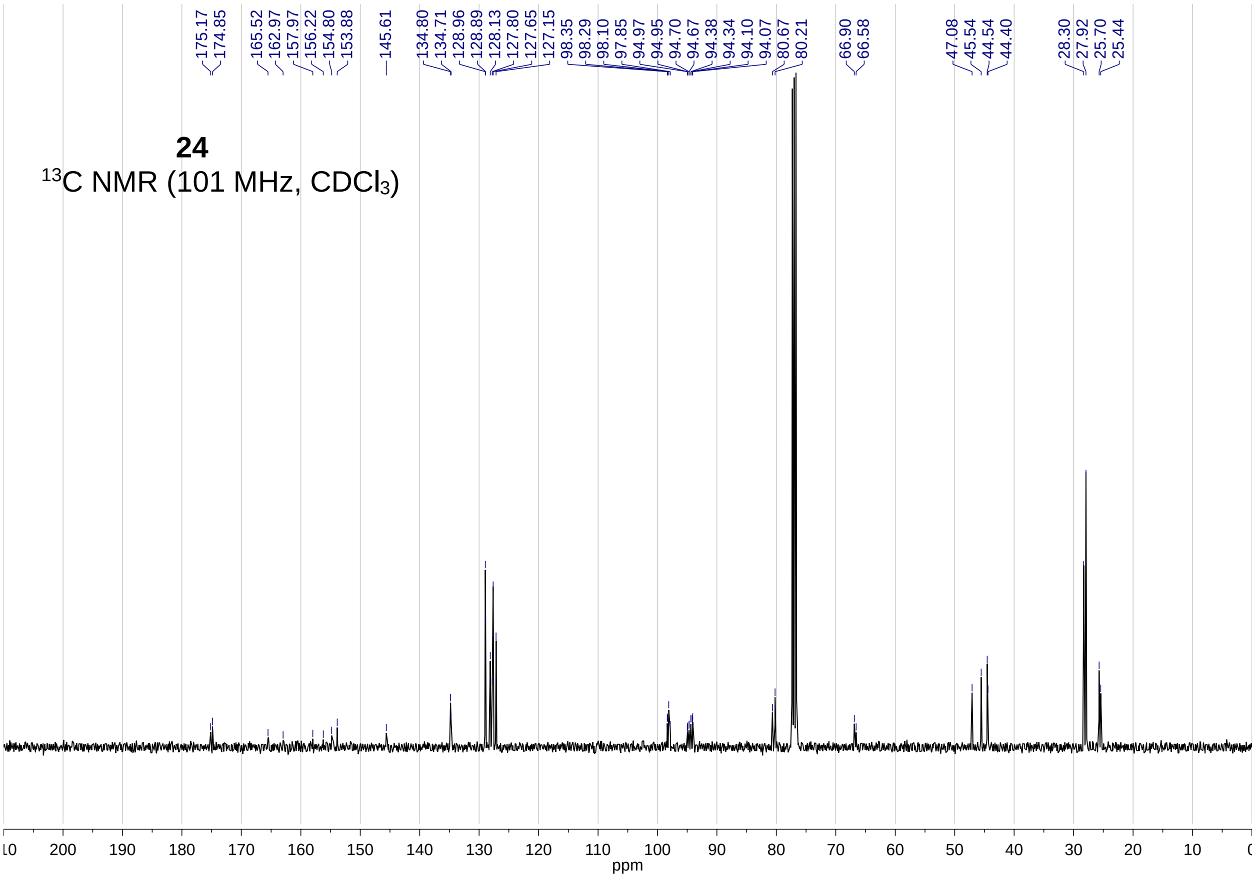
175.17 174.85 165.52 162.97 157.97 156.22 154.80 153.88 145.61 134.80 134.71 128.96 128.89 128.13 127.80 127.65 127.15 98.35 98.29 98.10 97.85 94.97 94.95 94.70 94.67 94.38 94.34 94.10 94.07 80.67 80.21 66.90 66.58 47.08 45.54 44.54 44.40 28.30 27.92 25.70 25.44
24
^{13}C NMR (101 MHz, $CDCl_3$)
ppm

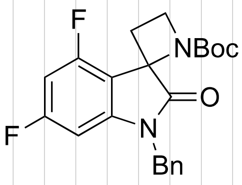

24

^{19}F NMR (377 MHz, $CDCl_3$)

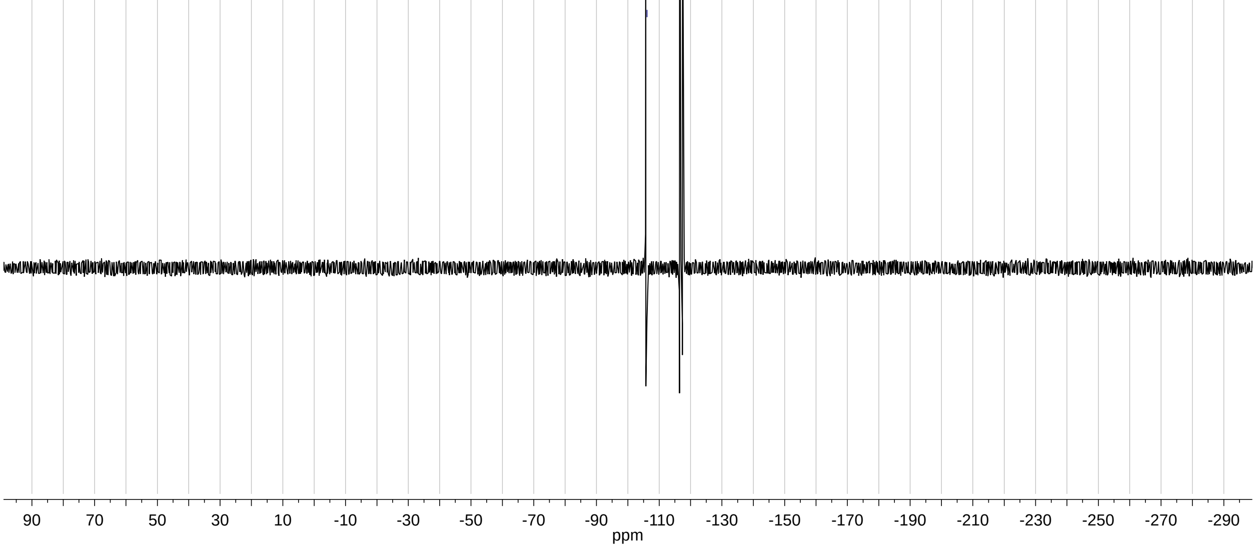

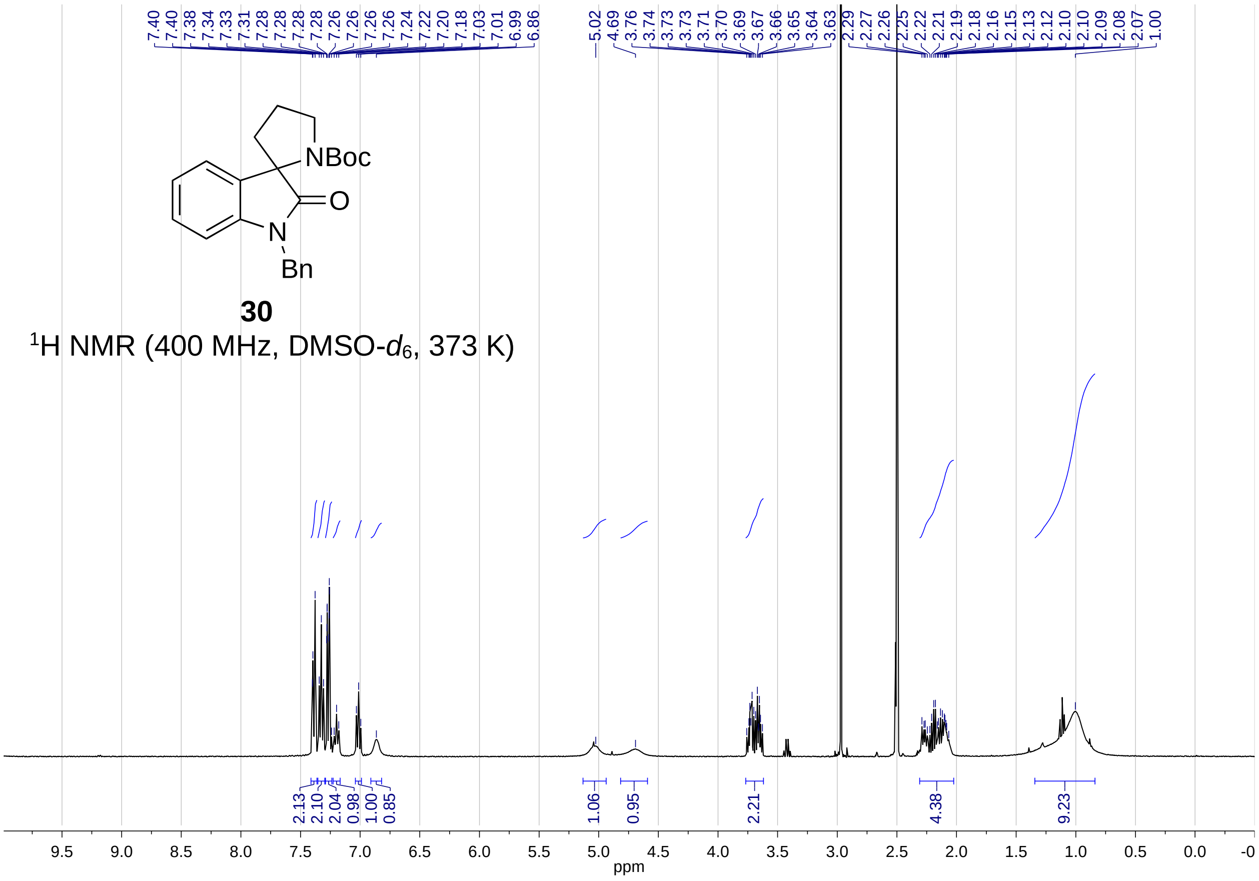

30

^{1}H NMR (400 MHz, DMSO-d_6, 373 K)

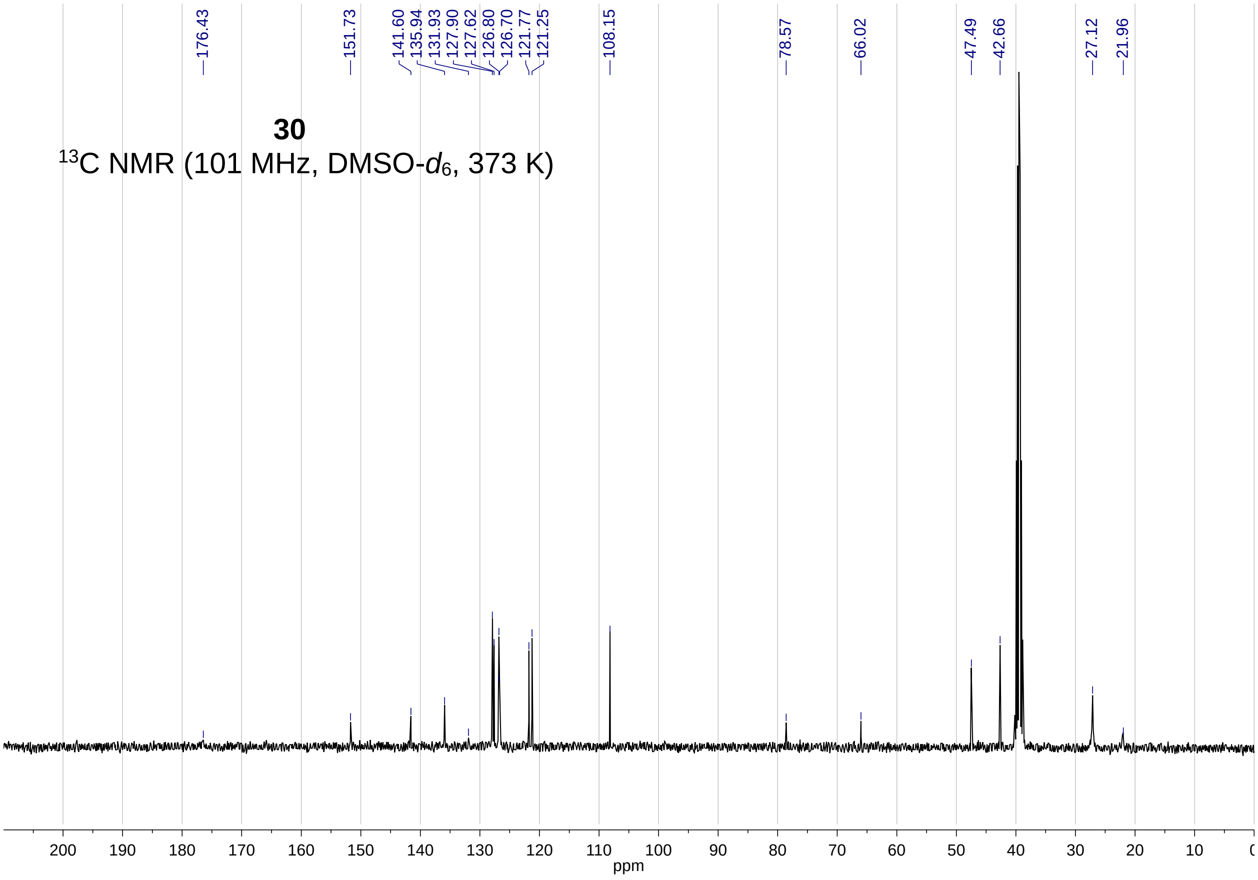

30

^{13}C NMR (101 MHz, DMSO-d_6, 373 K)

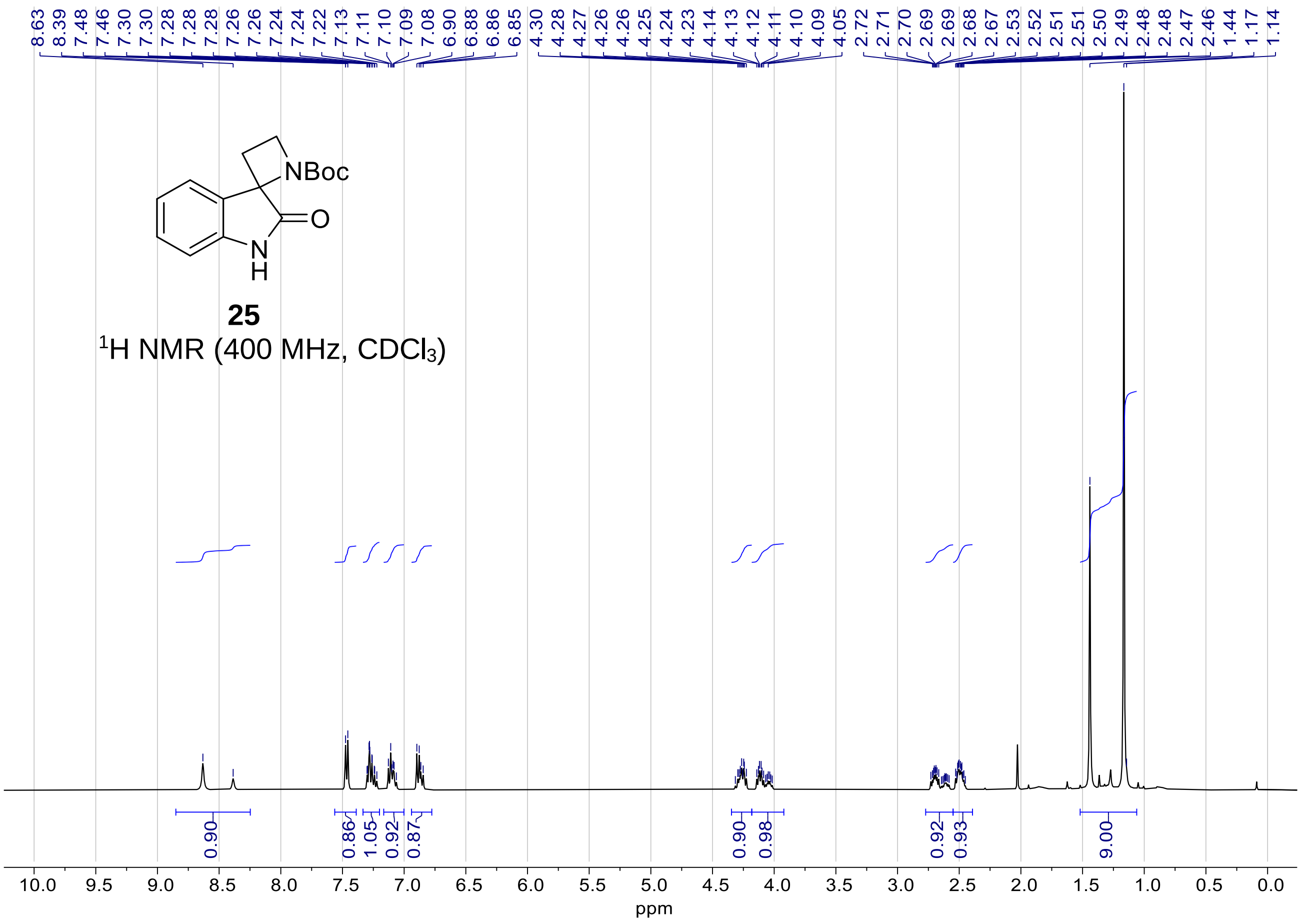
8.63 8.39 7.48 7.46 7.30 7.28 7.28 7.28 7.26 7.26 7.24 7.24 7.22 7.13 7.11 7.10 7.09 7.08 6.90 6.88 6.86 6.85 4.30 4.28 4.27 4.26 4.26 4.25 4.24 4.23 4.14 4.13 4.12 4.11 4.10 4.09 4.05 2.72 2.71 2.70 2.69 2.69 2.68 2.67 2.53 2.52 2.51 2.51 2.50 2.49 2.48 2.48 2.47 2.46 1.44 1.17 1.14
NBoc
O
N
H
25
1H NMR (400 MHz, CDCl3)
0.90
0.86
1.05
0.92
0.87
0.90
0.98
0.92
0.93
9.00
10.0 9.5 9.0 8.5 8.0 7.5 7.0 6.5 6.0 5.5 5.0 4.5 4.0 3.5 3.0 2.5 2.0 1.5 1.0 0.5 0.0
ppm

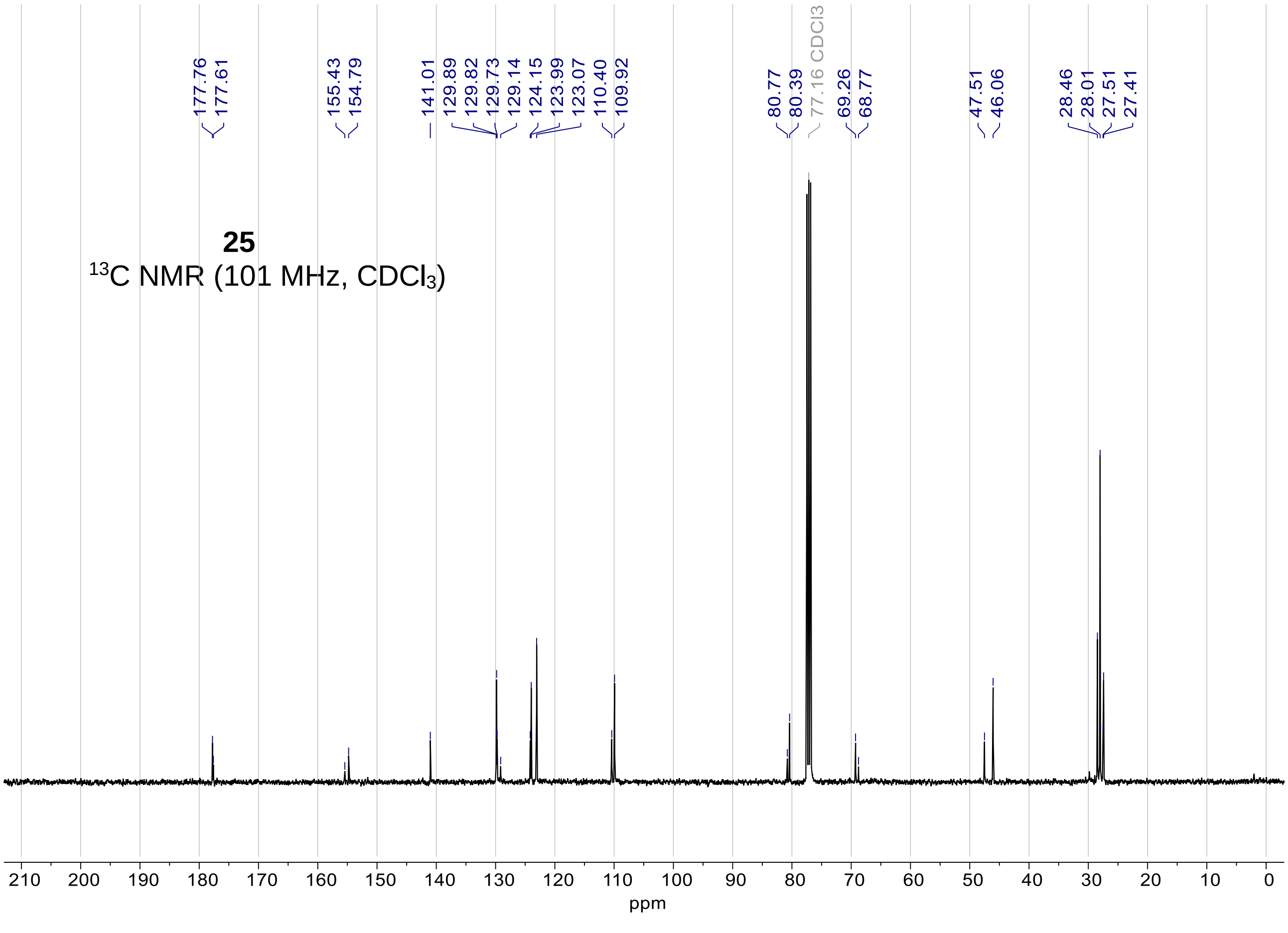
177.76 177.61
155.43 154.79
141.01 129.89 129.82 129.73 129.14 124.15 123.99 123.07 110.40 109.92
80.77 80.39 77.16 CDCl3 69.26 68.77
47.51 46.06
28.46 28.01 27.51 27.41
25
13C NMR (101 MHz, CDCl3)
210 200 190 180 170 160 150 140 130 120 110 100 90 80 70 60 50 40 30 20 10 0
ppm

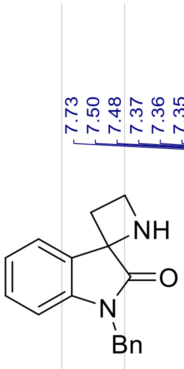

26

^{1}H NMR (400 MHz, DMSO-d_6)

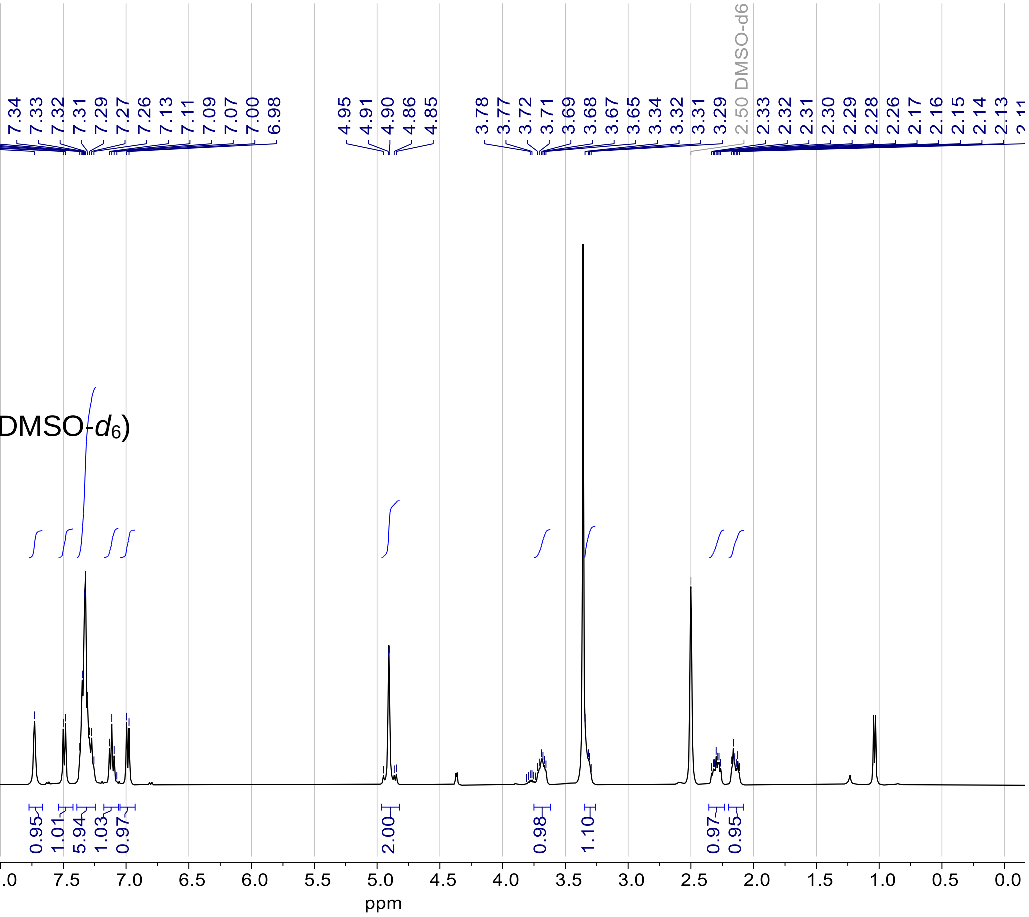

26

^{13}C NMR (101 MHz, DMSO-d_6)

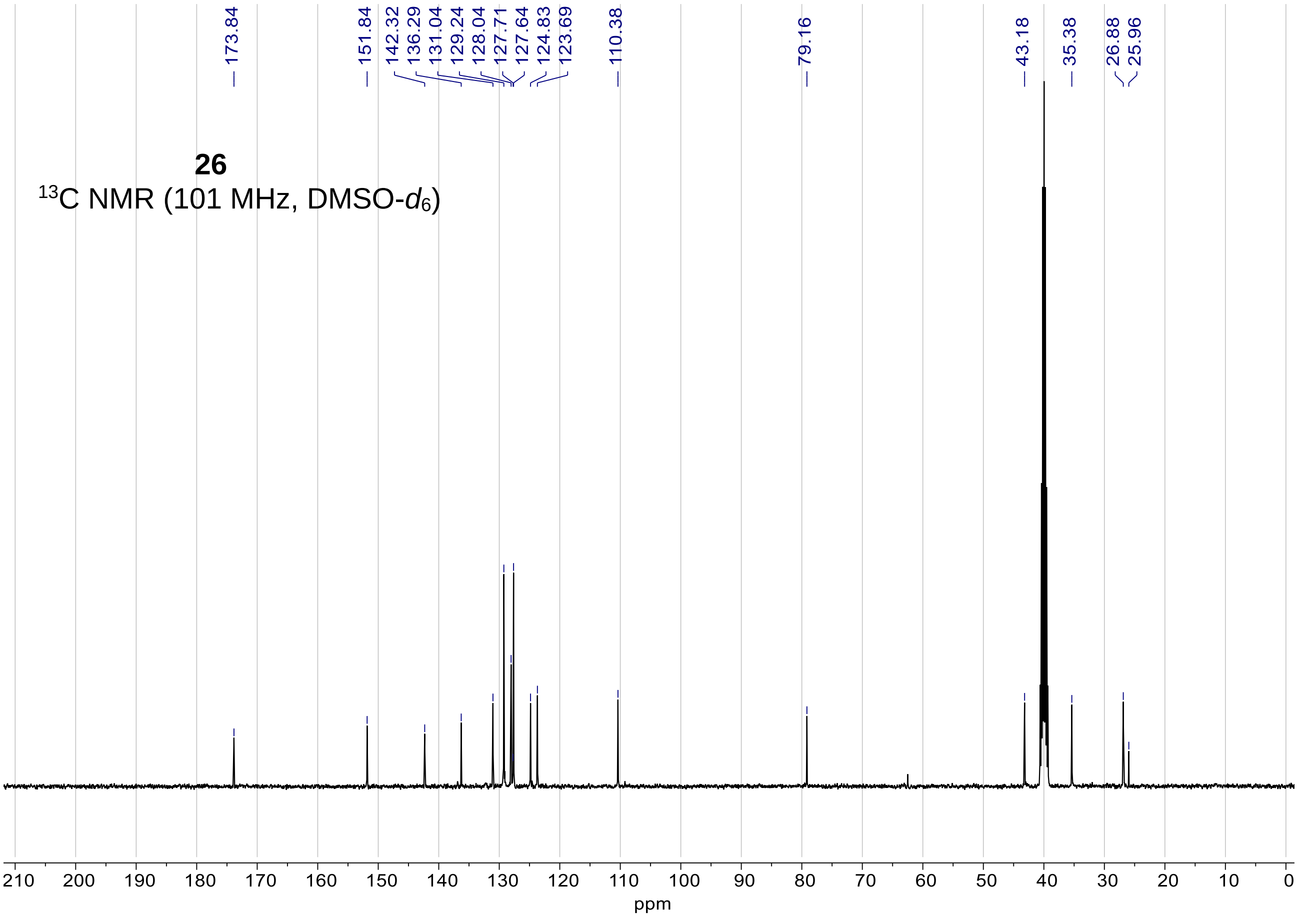

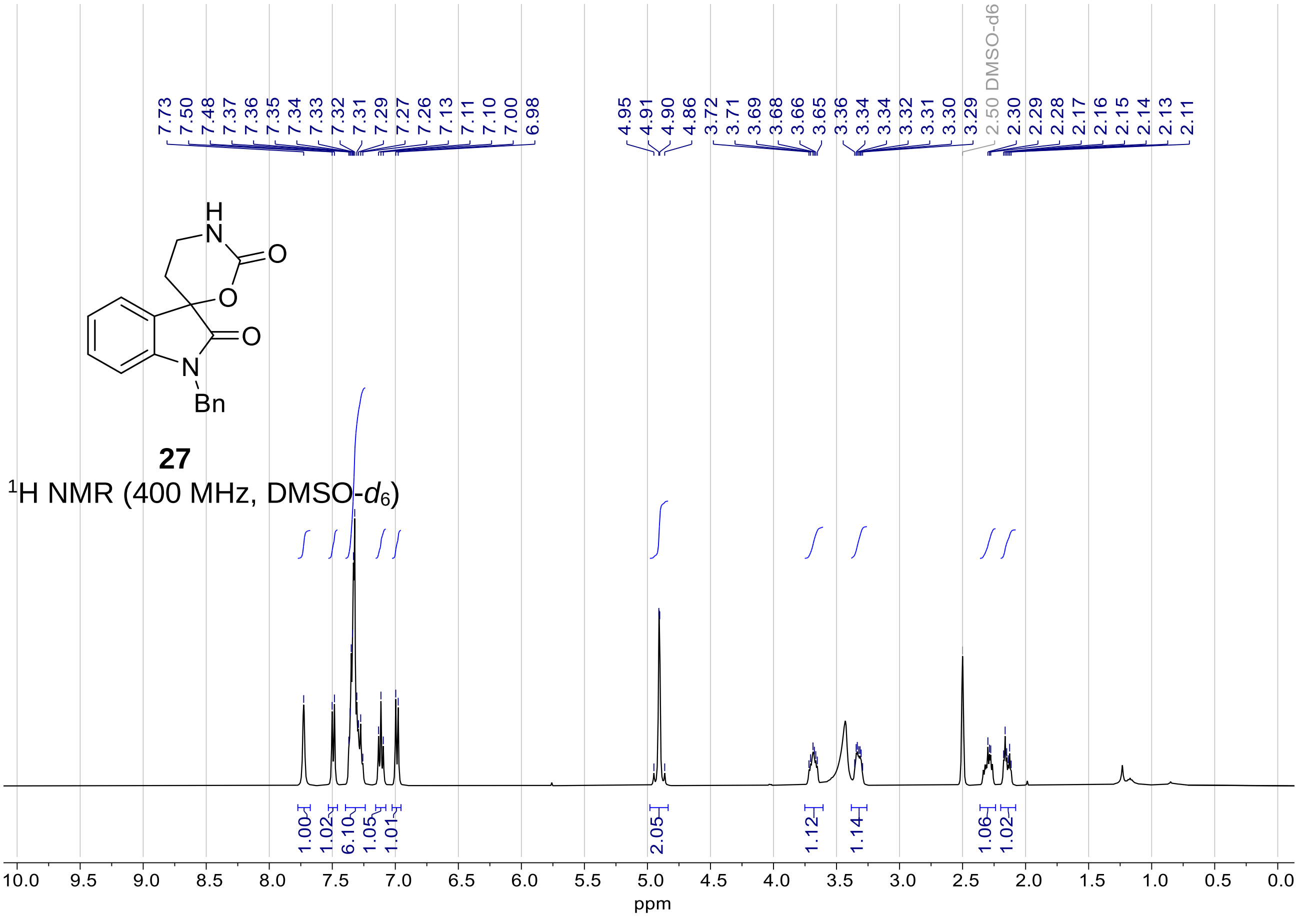

27

^{1}H NMR (400 MHz, DMSO-d_6)

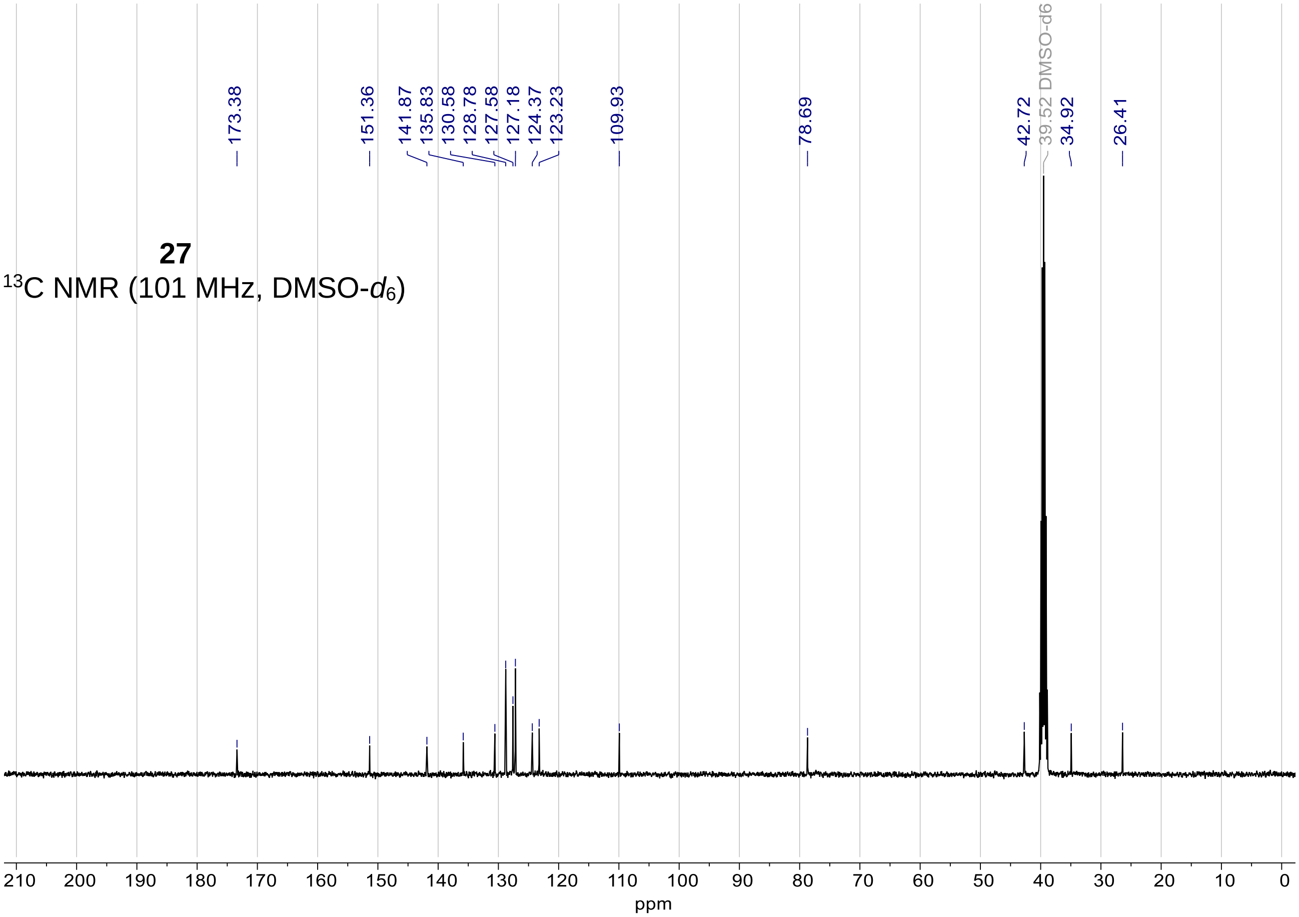

27

^{13}C NMR (101 MHz, DMSO-d_6)

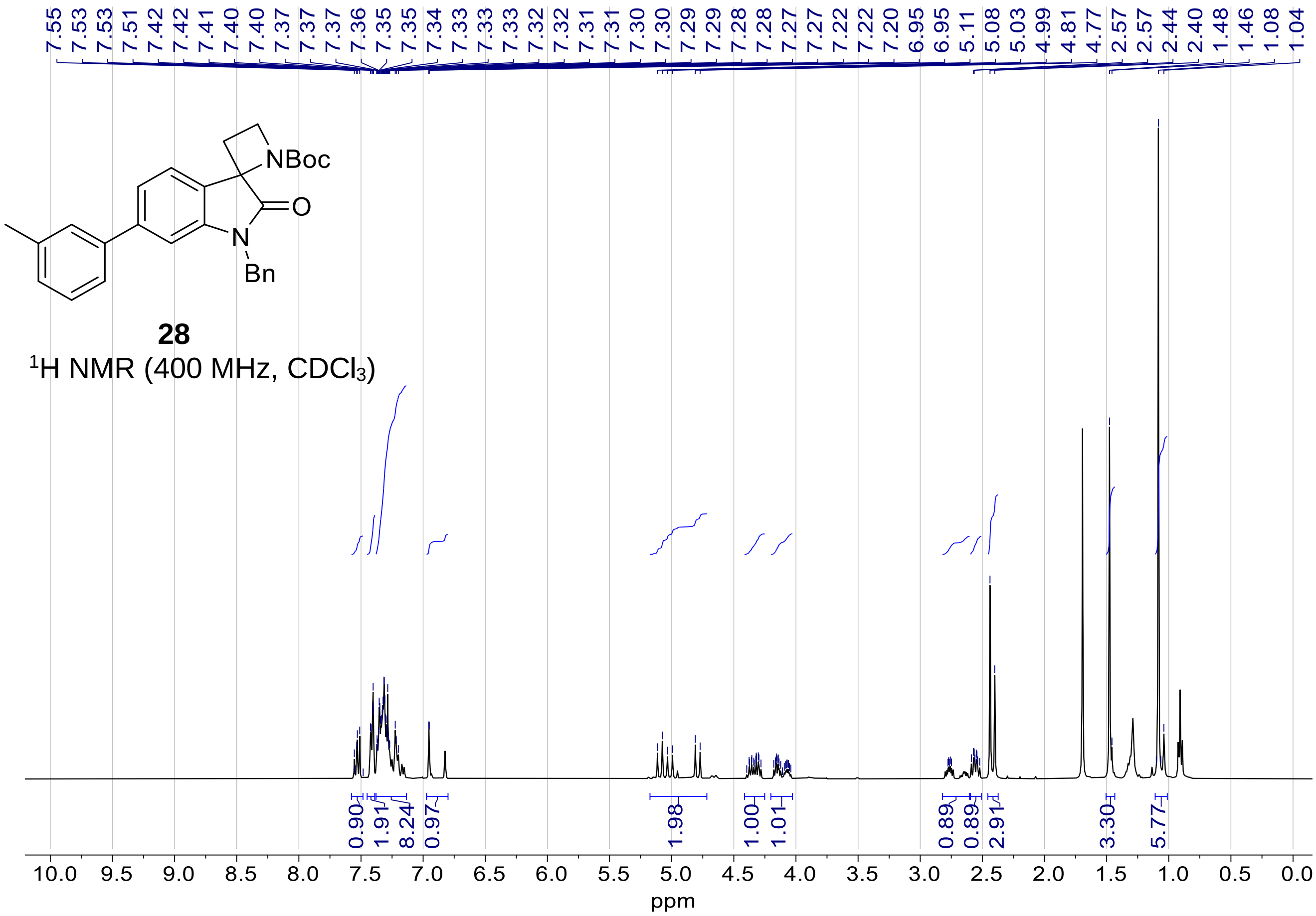

28
^{1}H NMR (400 MHz, $CDCl_3$)

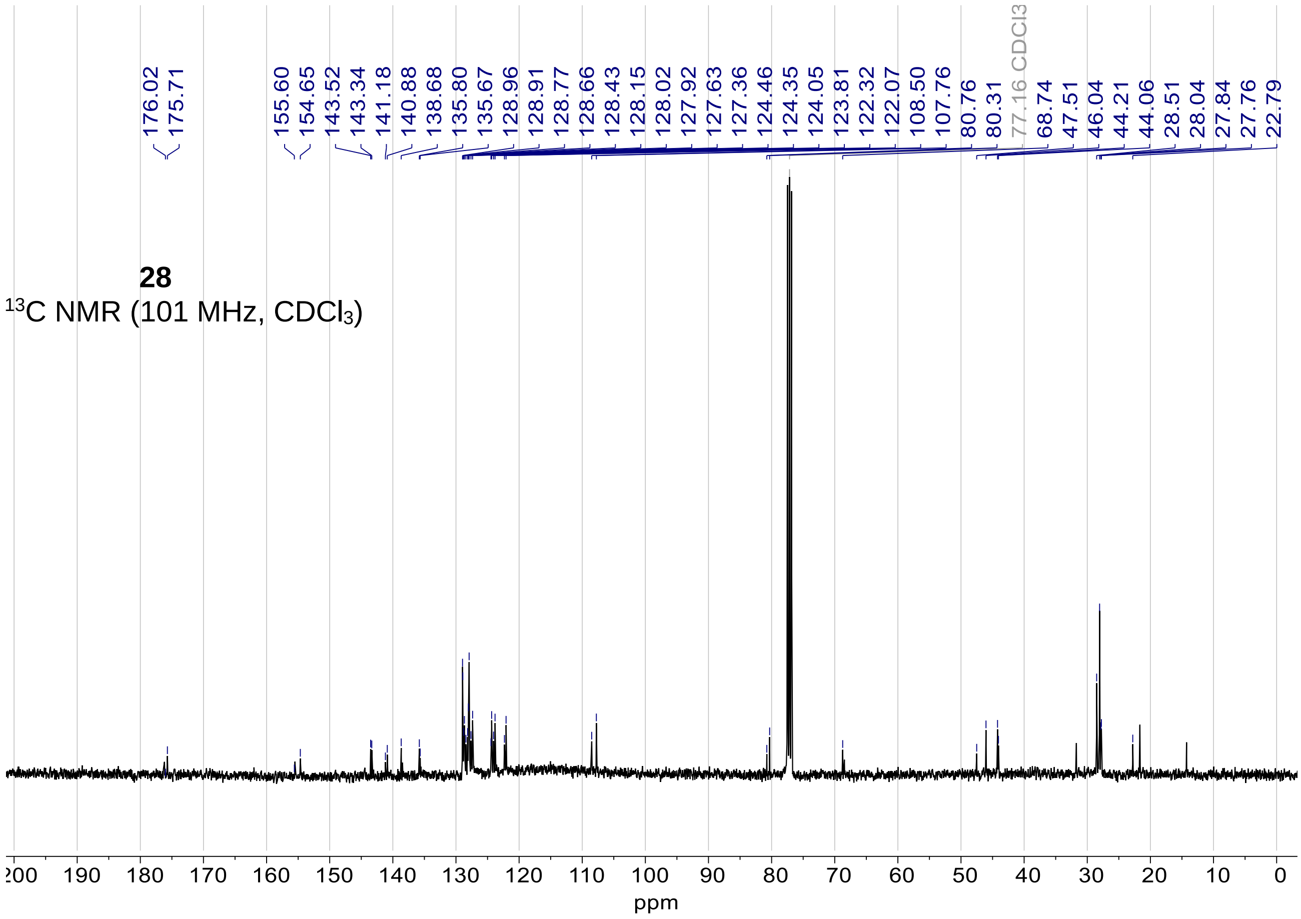

28
^{13}C NMR (101 MHz, $CDCl_3$)

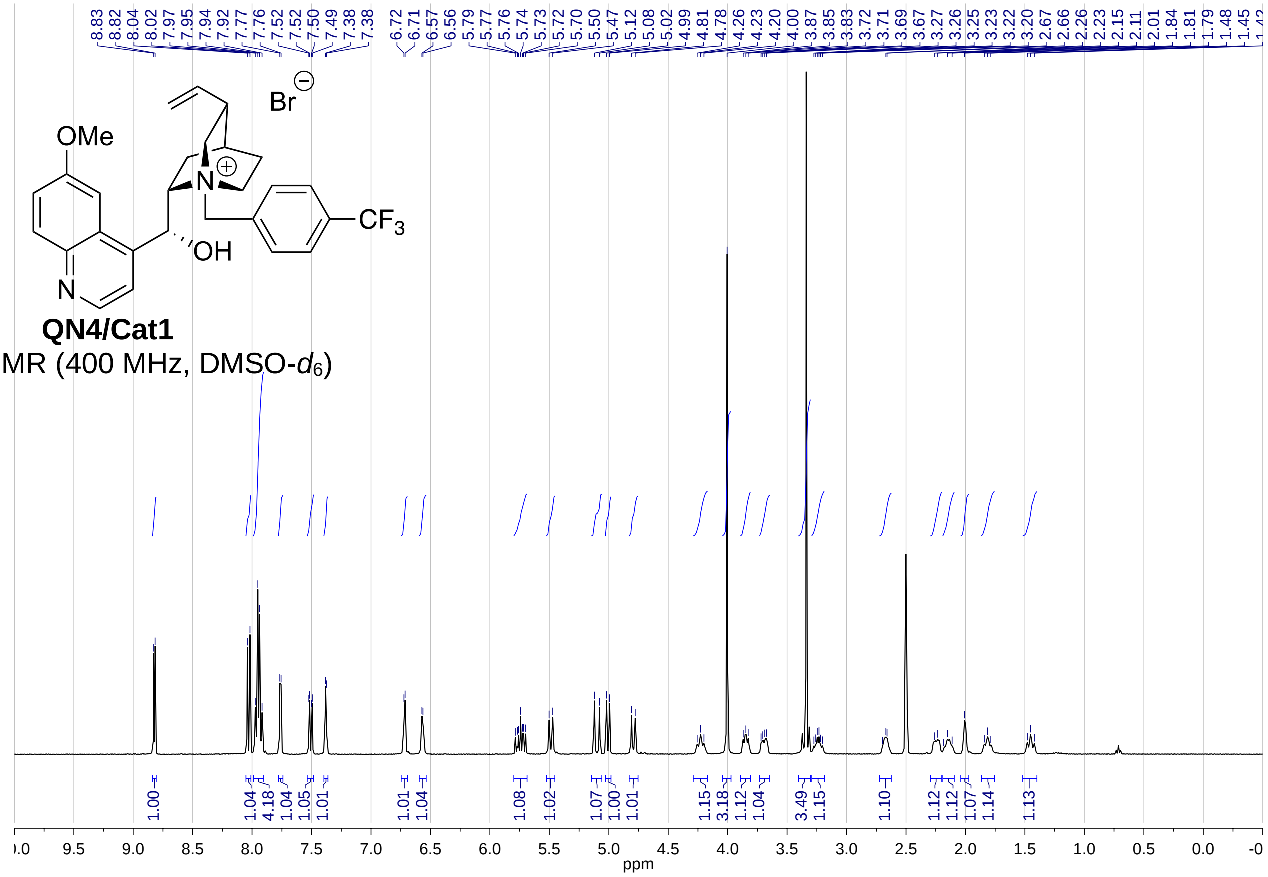

QN4/Cat1
^{1}H NMR (400 MHz, DMSO-d_6)

QN4/Cat1
^{1}H NMR (400 MHz, $CDCl_3$)

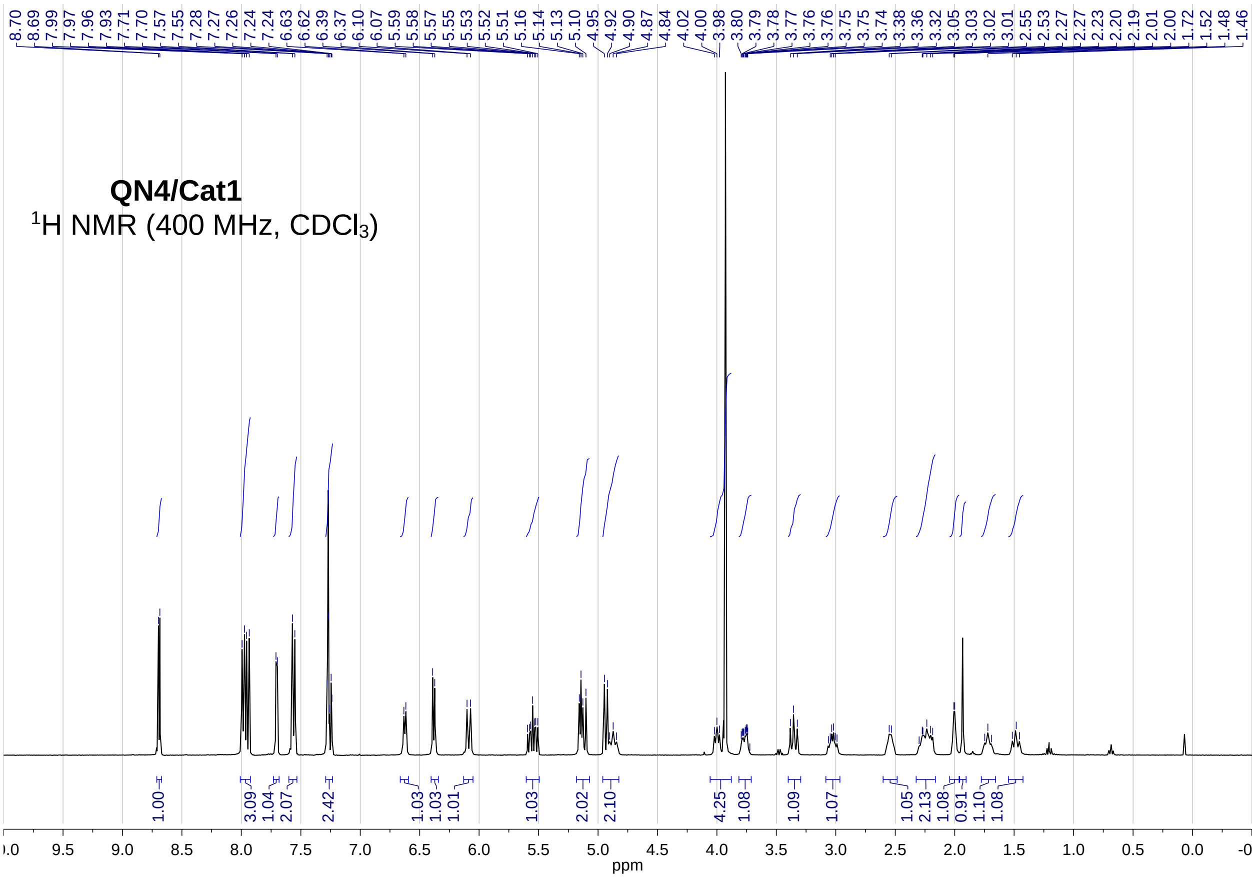

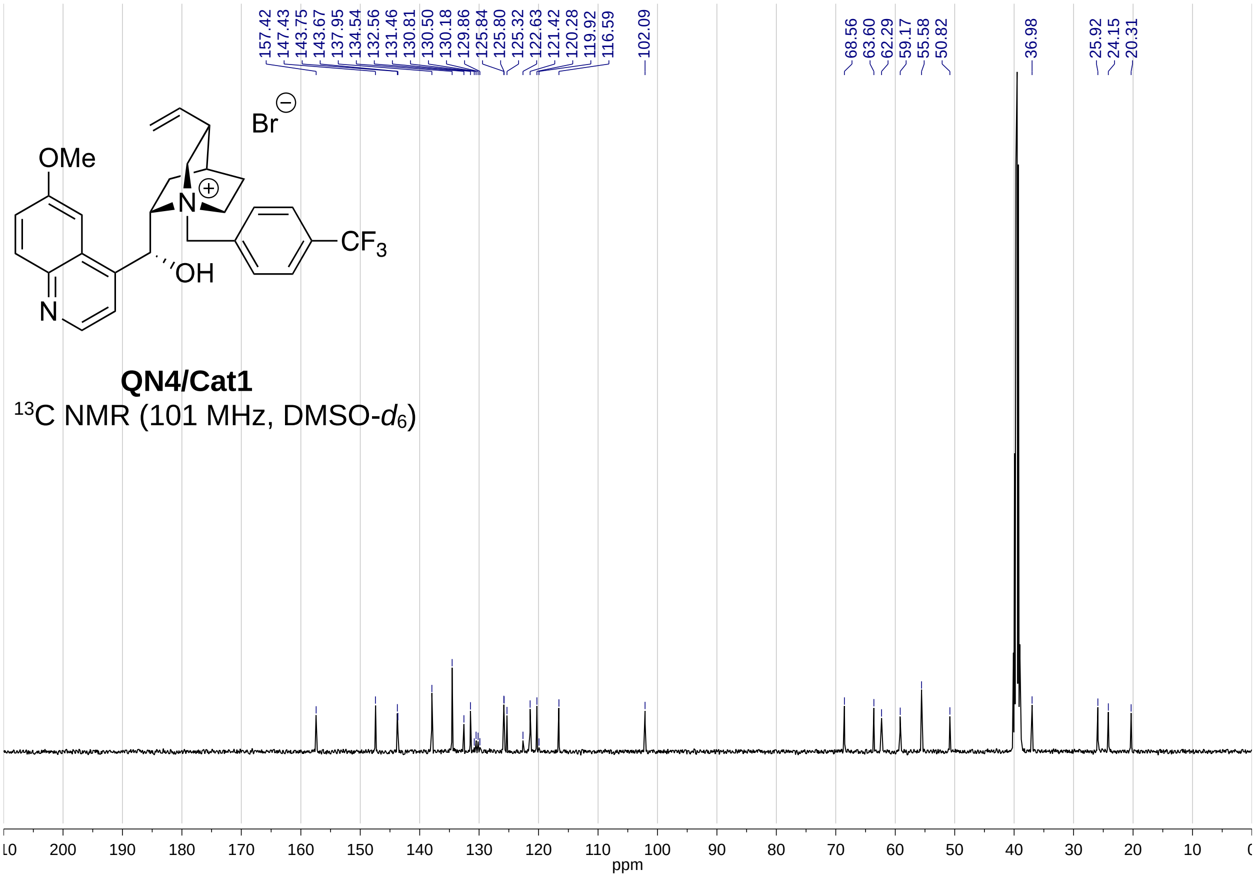
157.42
147.43
143.75
143.67
137.95
134.54
132.56
131.46
130.81
130.50
130.18
129.86
125.84
125.80
125.32
122.63
121.42
120.28
119.92
116.59
102.09
68.56
63.60
62.29
59.17
55.58
50.82
36.98
25.92
24.15
20.31
OMe
Br
N
CF3
OH
N
QN4/Cat1
^{13}C NMR (101 MHz, DMSO-d_6)
ppm

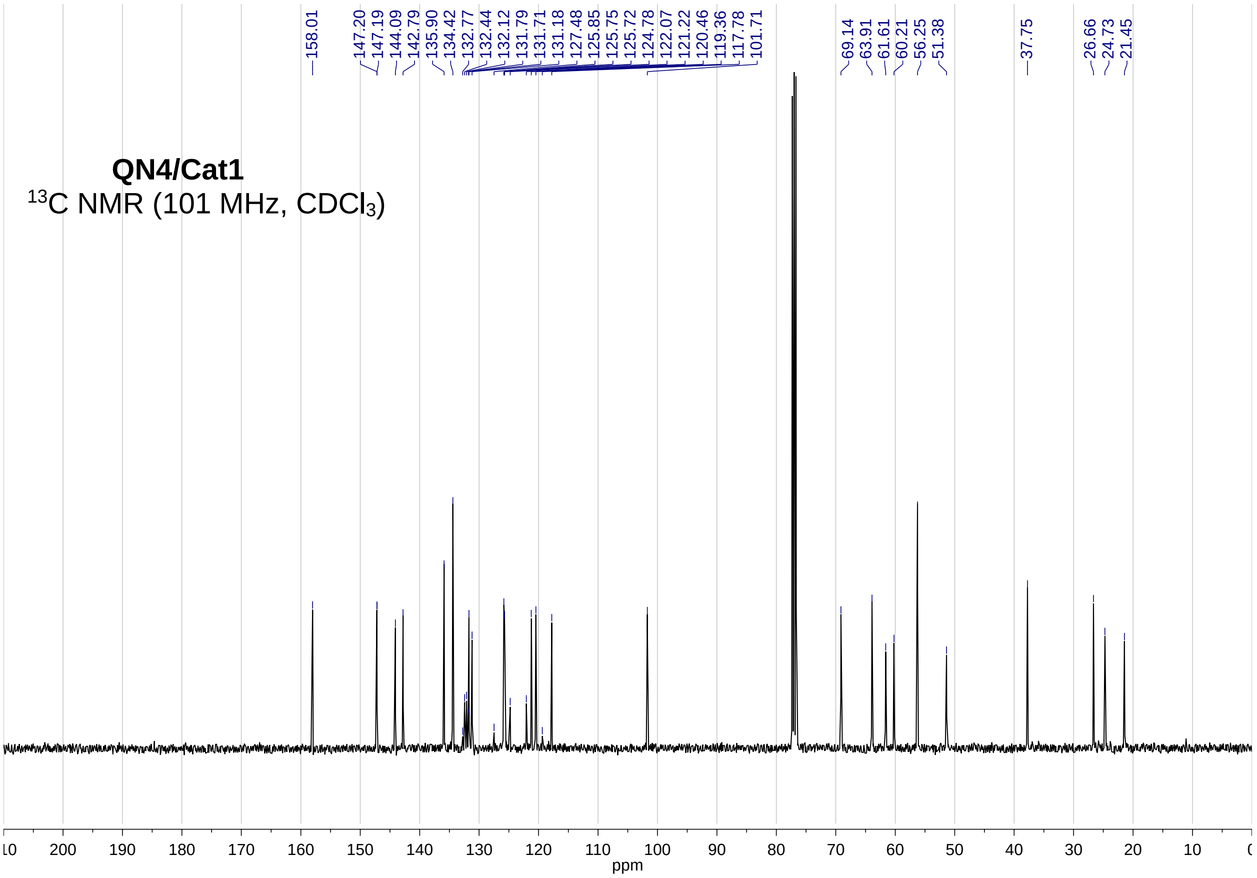
158.01
147.20
147.19
144.09
142.79
135.90
134.42
132.77
132.44
132.12
131.79
131.71
131.18
127.48
125.85
125.75
125.72
124.78
122.07
121.22
120.46
119.36
117.78
101.71
69.14
63.91
61.61
60.21
56.25
51.38
37.75
26.66
24.73
21.45
QN4/Cat1
^{13}C NMR (101 MHz, $CDCl_3$)
ppm

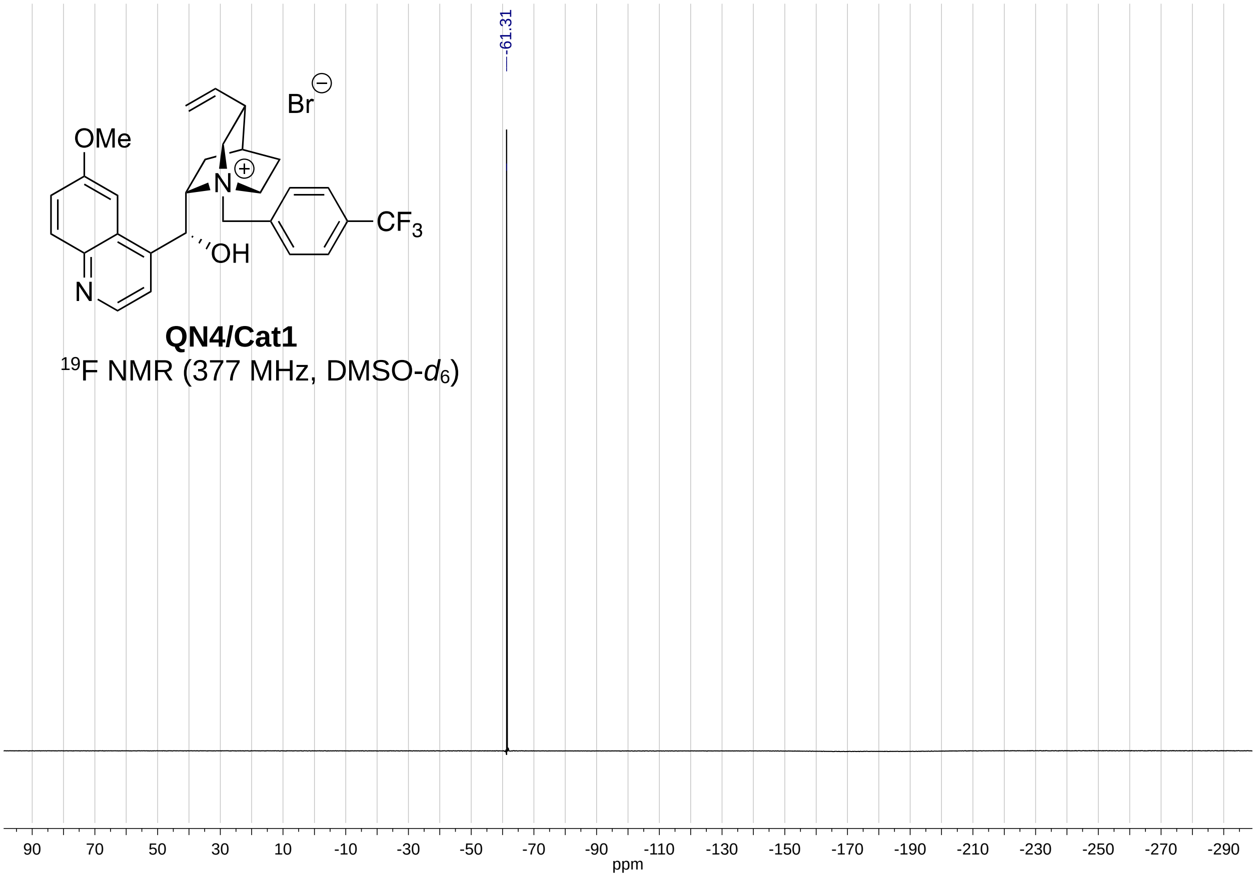

-61.31
OMe
Br
N
OH
CF3
N
QN4/Cat1
19F NMR (377 MHz, DMSO-d6)
90 70 50 30 10 -10 -30 -50 -70 -90 -110 -130 -150 -170 -190 -210 -230 -250 -270 -290
ppm

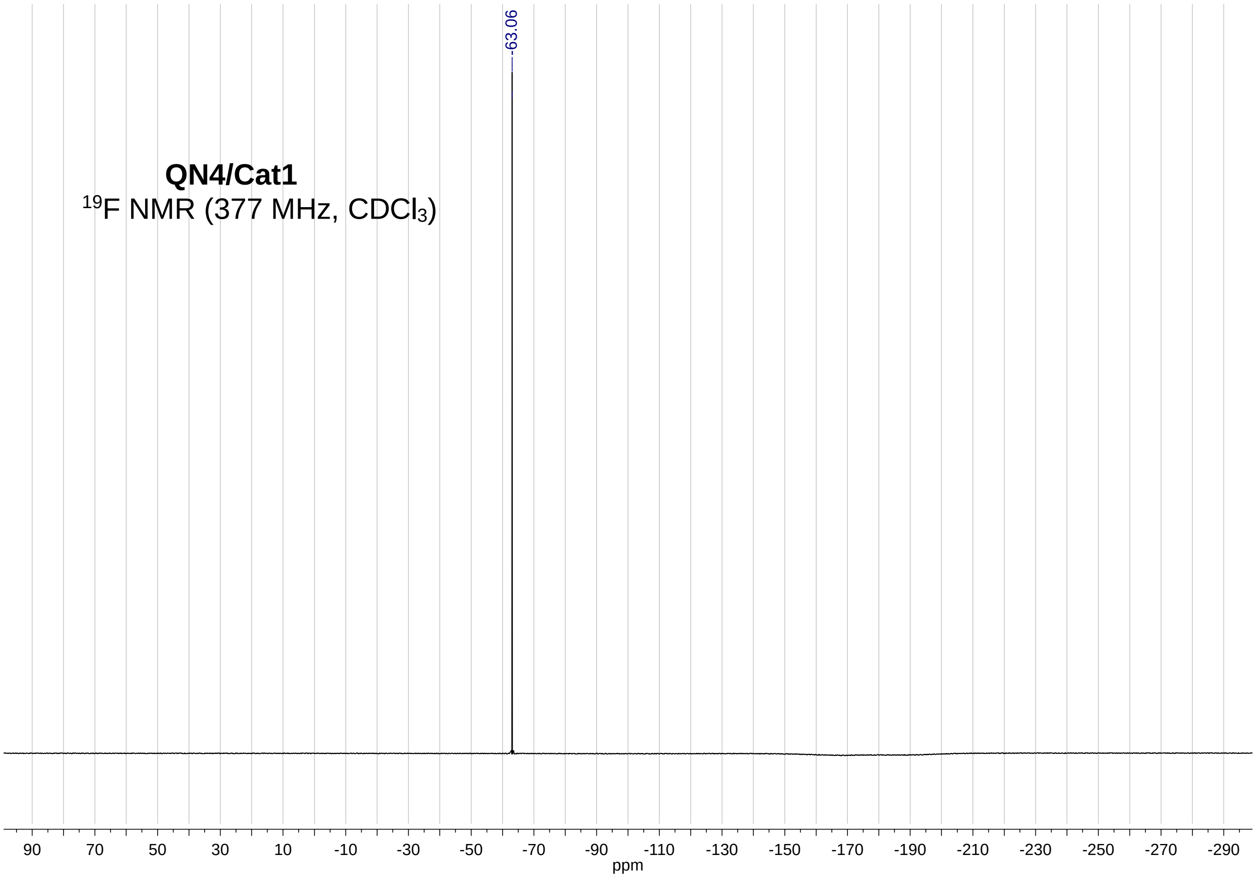

-63.06
QN4/Cat1
19F NMR (377 MHz, CDCl3)
90 70 50 30 10 -10 -30 -50 -70 -90 -110 -130 -150 -170 -190 -210 -230 -250 -270 -290
ppm

S60

^{1}H NMR (400 MHz, $CDCl_3$)

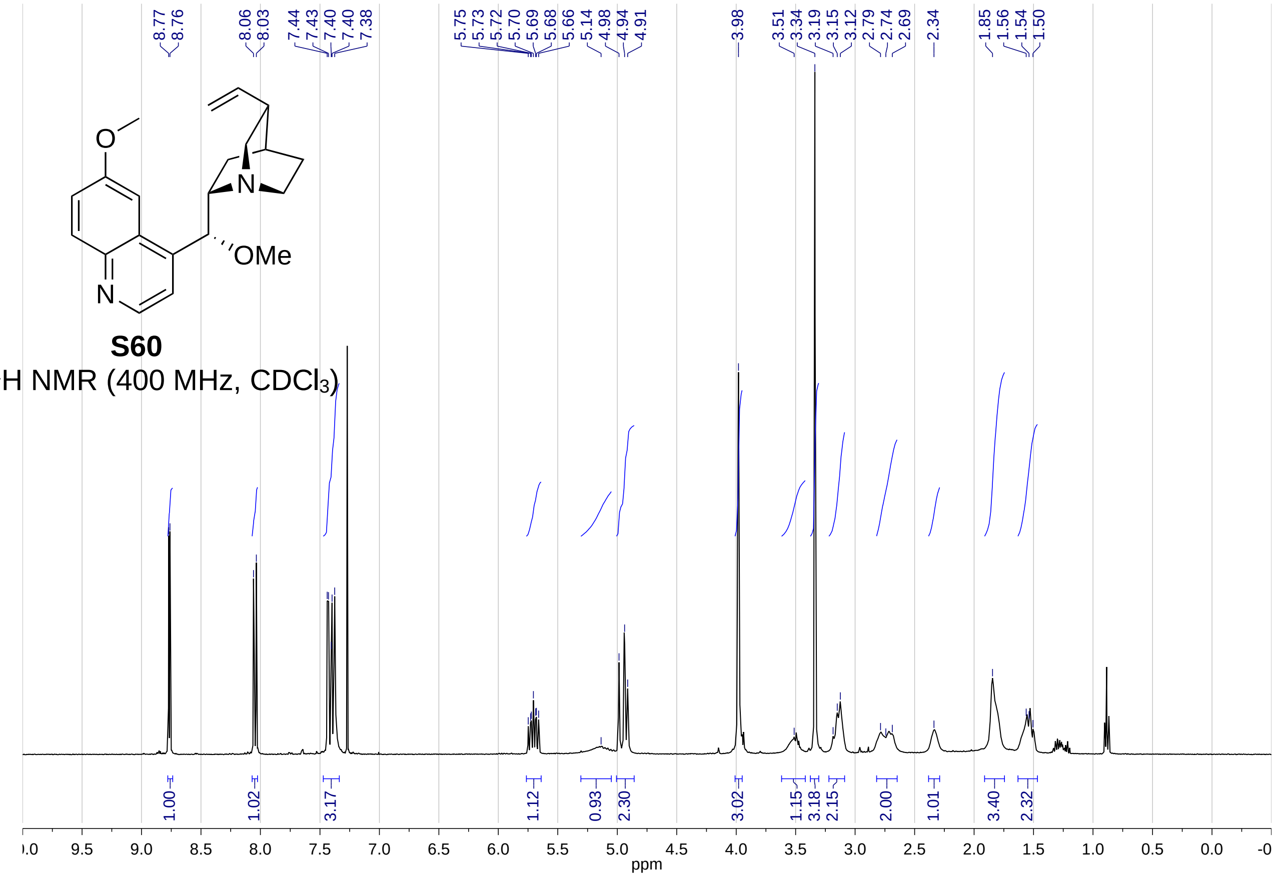

S60

^{13}C NMR (101 MHz, $CDCl_3$)

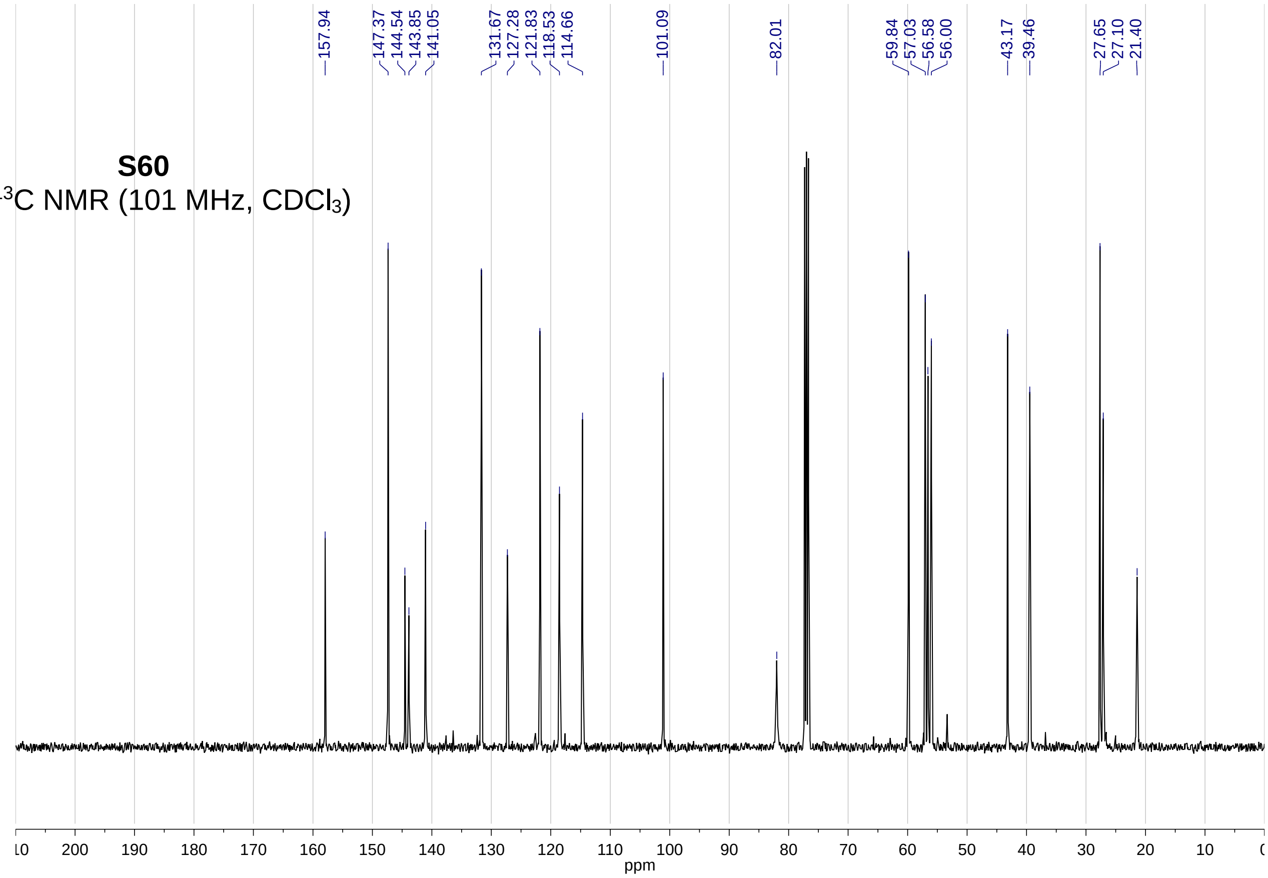

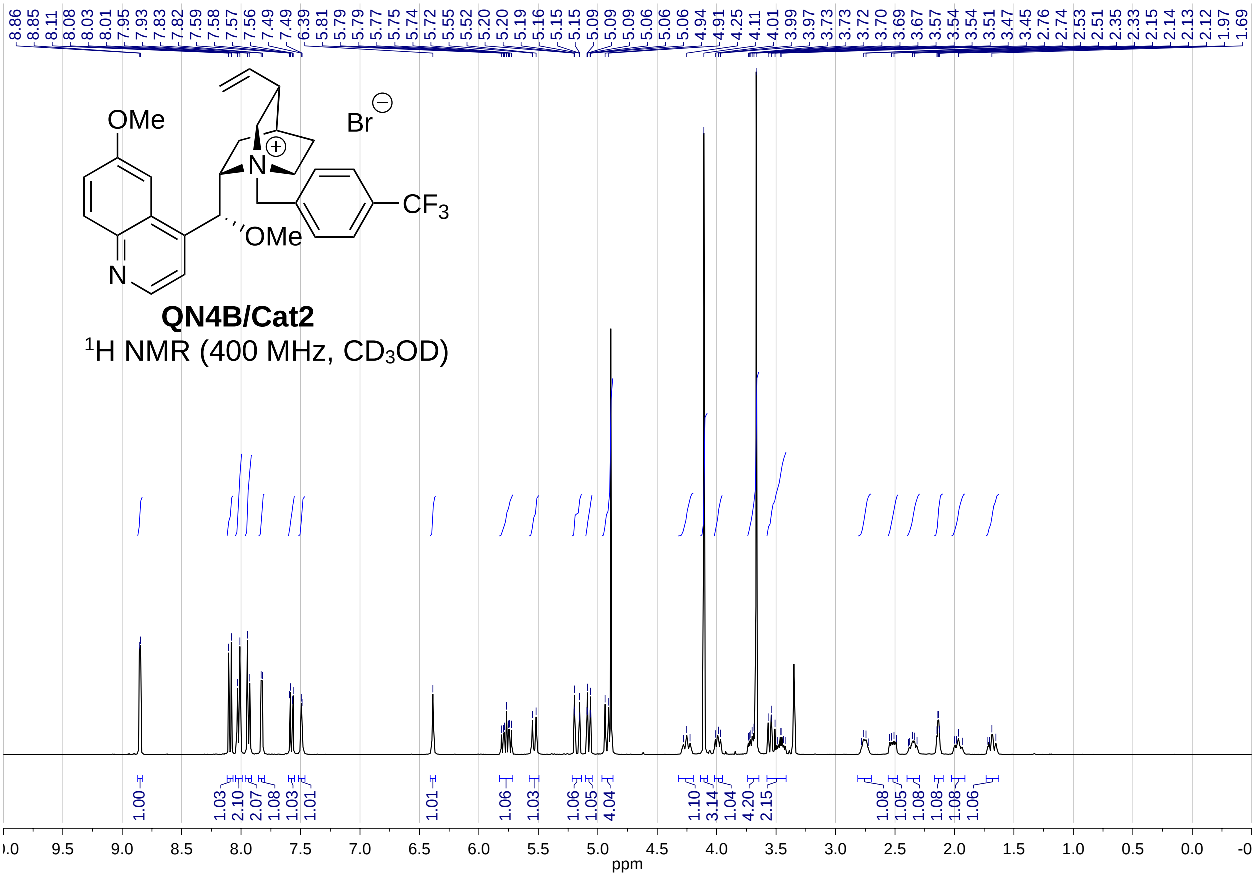
QN4B/Cat2
¹H NMR (400 MHz, CD₃OD)
ppm

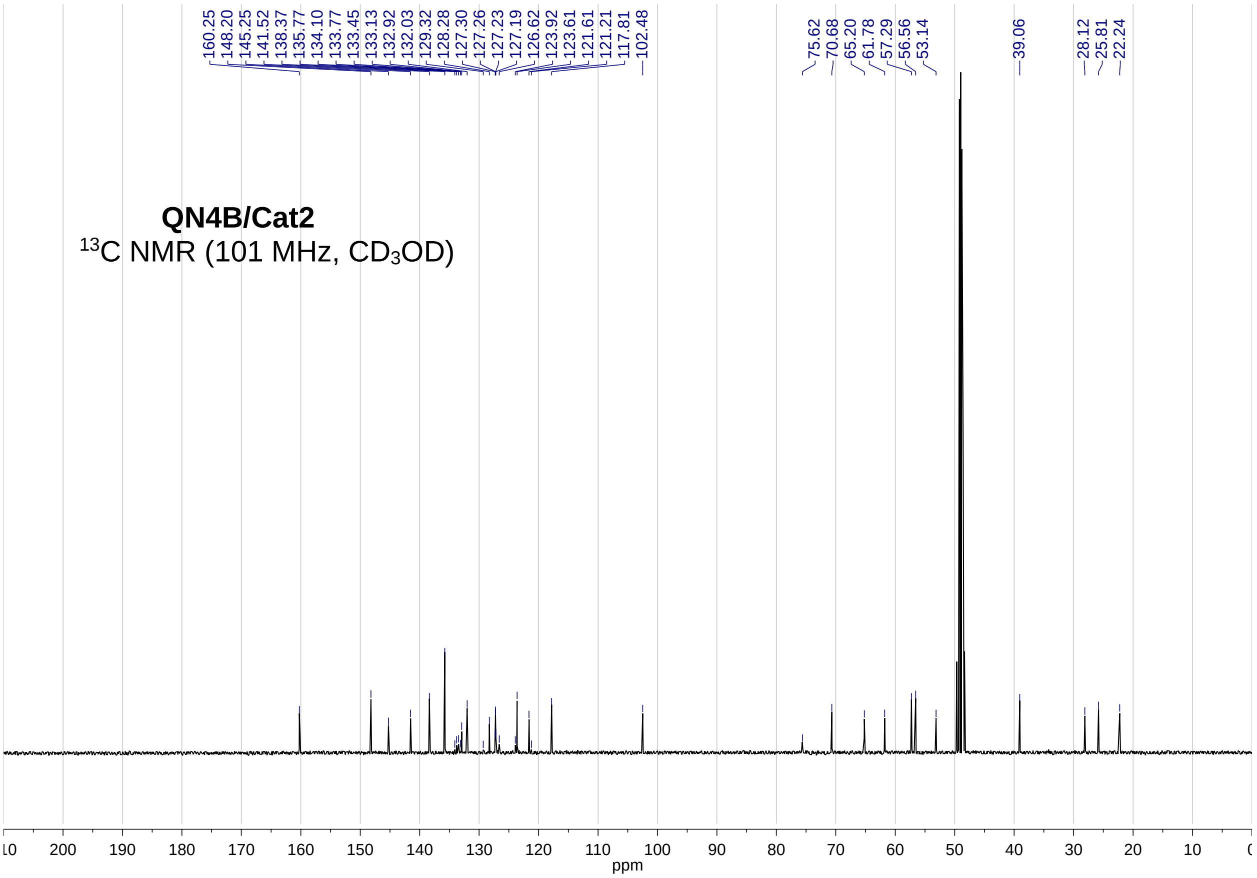
QN4B/Cat2
¹³C NMR (101 MHz, CD₃OD)
ppm

QN4B/Cat2

^{19}F NMR (377 MHz, CD_3OD)

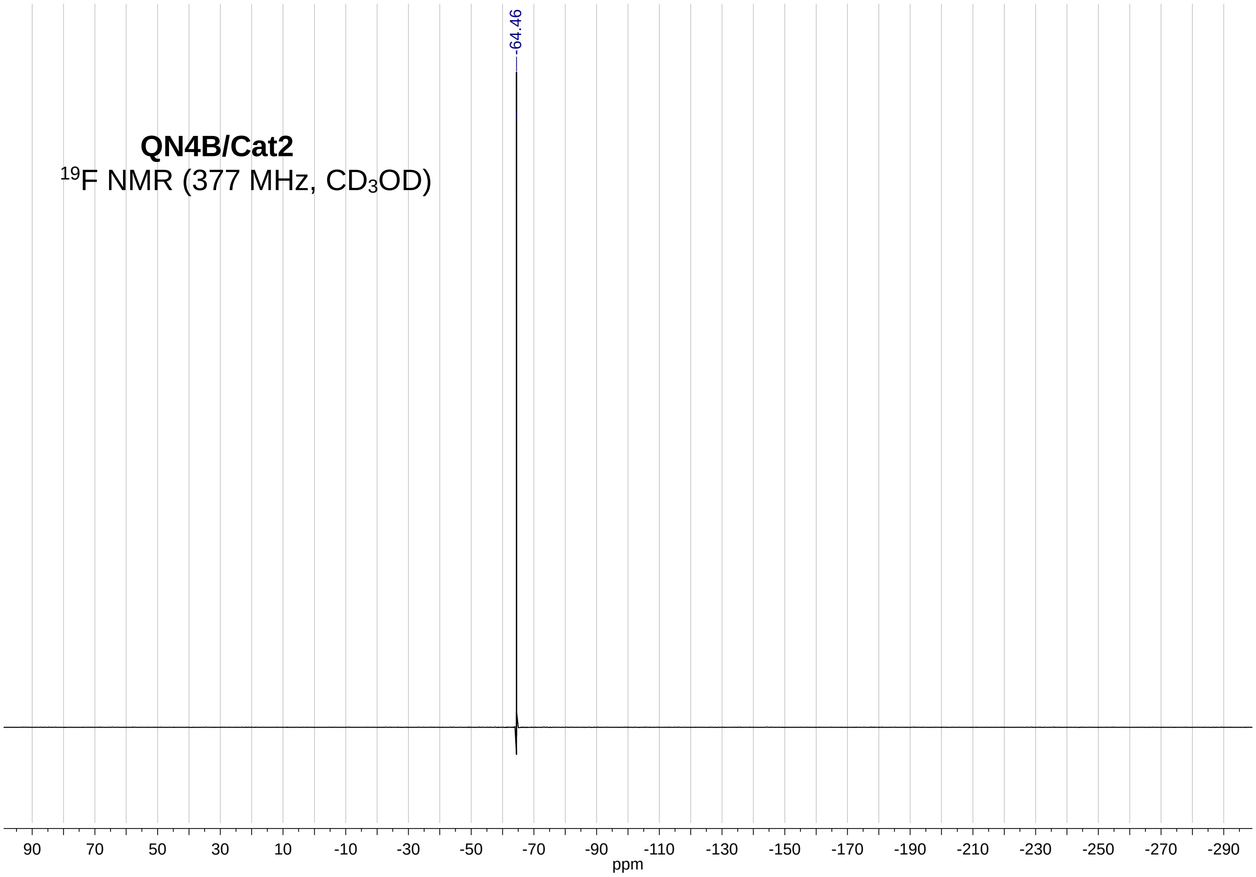

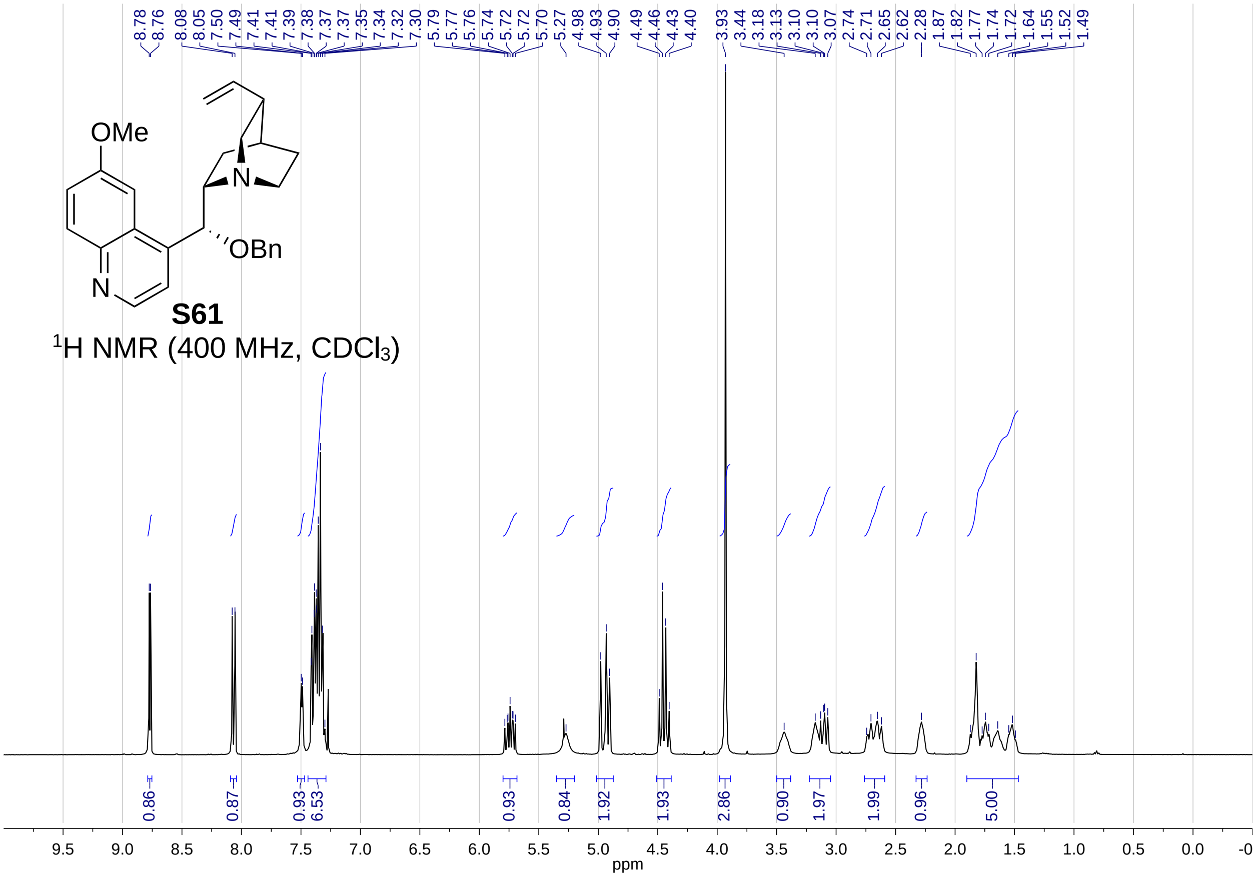

S61

^{1}H NMR (400 MHz, $CDCl_3$)

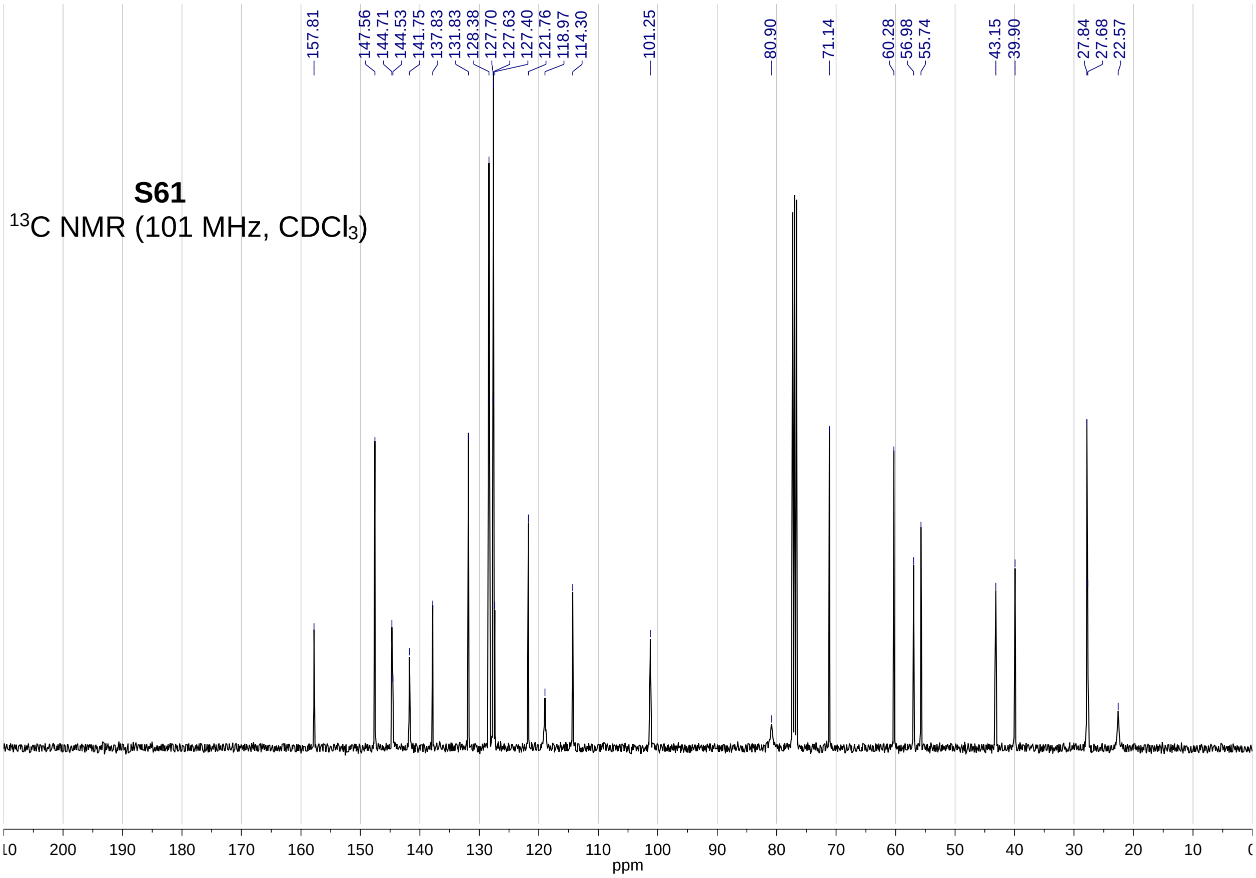

S61

^{13}C NMR (101 MHz, $CDCl_3$)

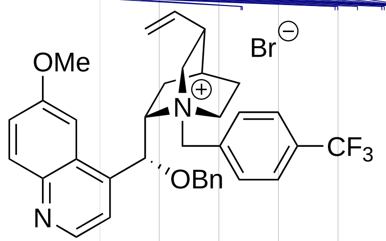

QN4C

^{1}H NMR (400 MHz, $CDCl_3$)

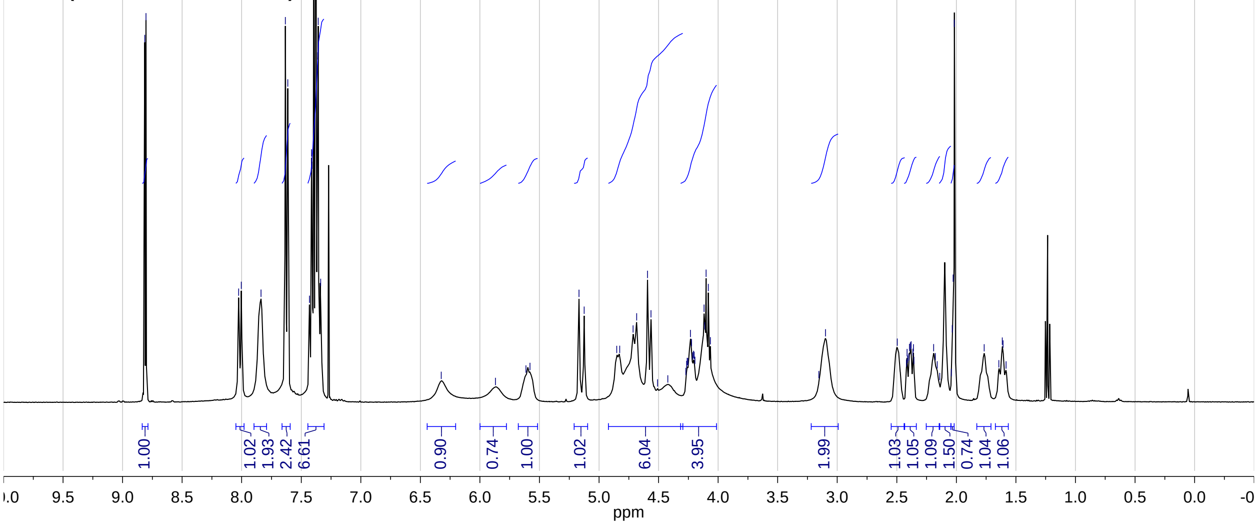

QN4C

^{13}C NMR (101 MHz, $CDCl_3$)

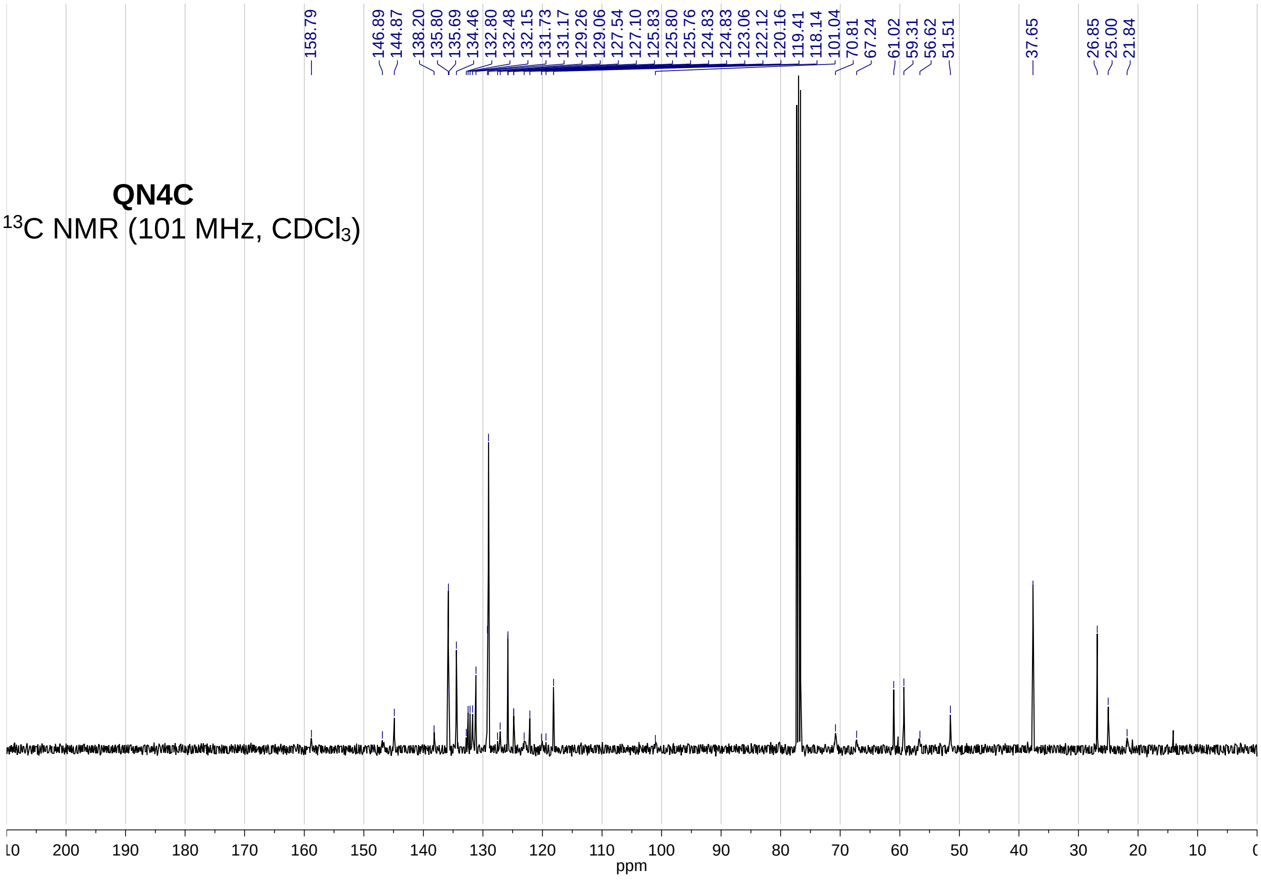

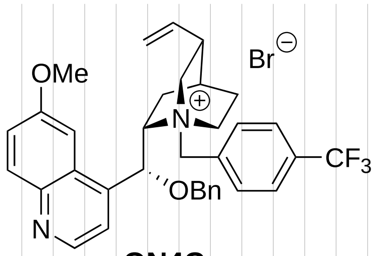

QN4C

^{19}F NMR (377 MHz, $CDCl_3$)

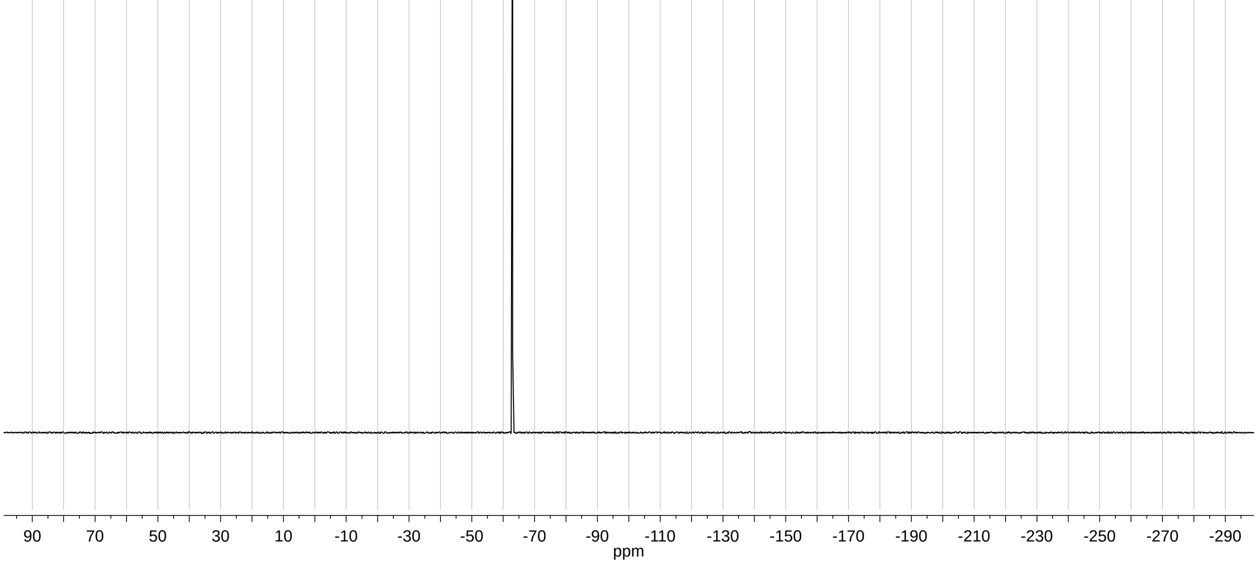

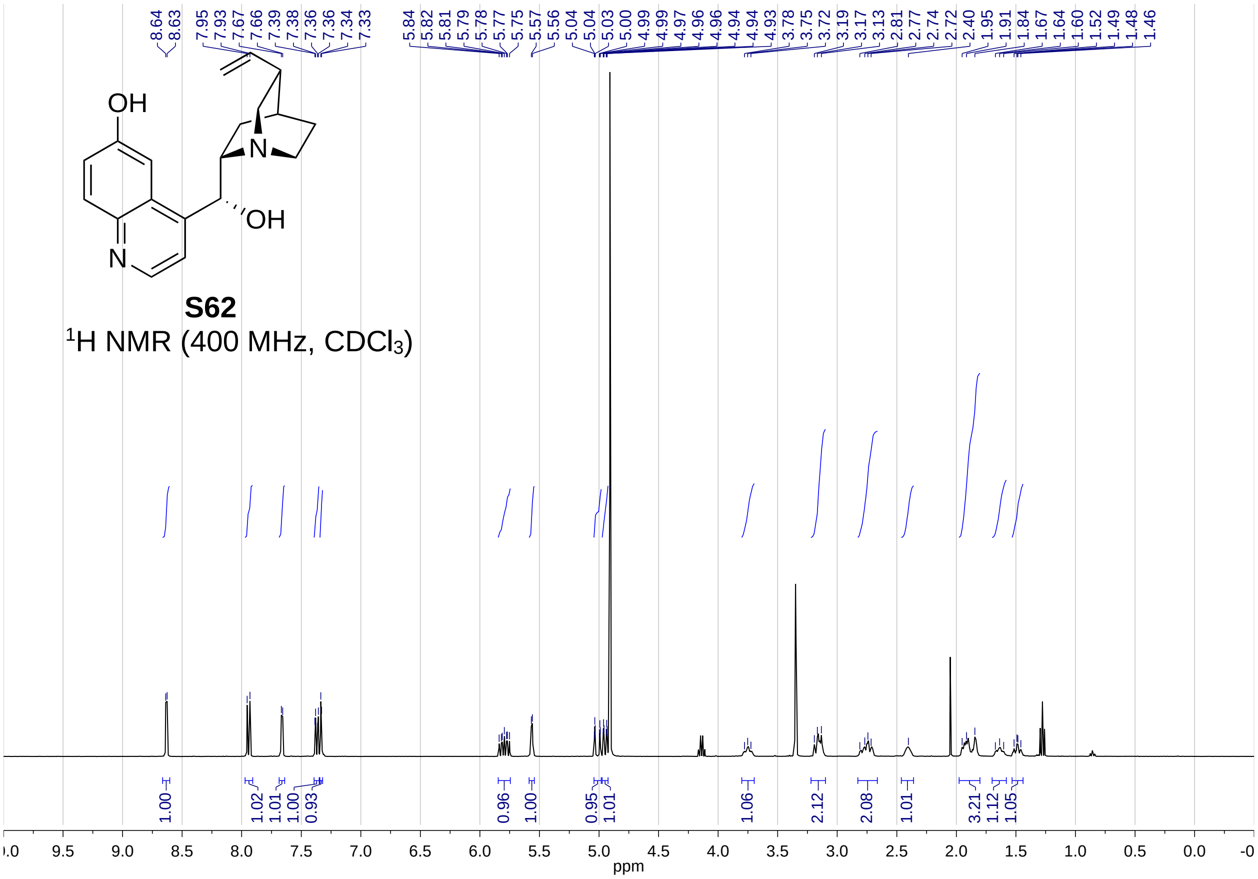
S62
^{1}H NMR (400 MHz, CDCl$_3$)
OH
N
OH
N
ppm

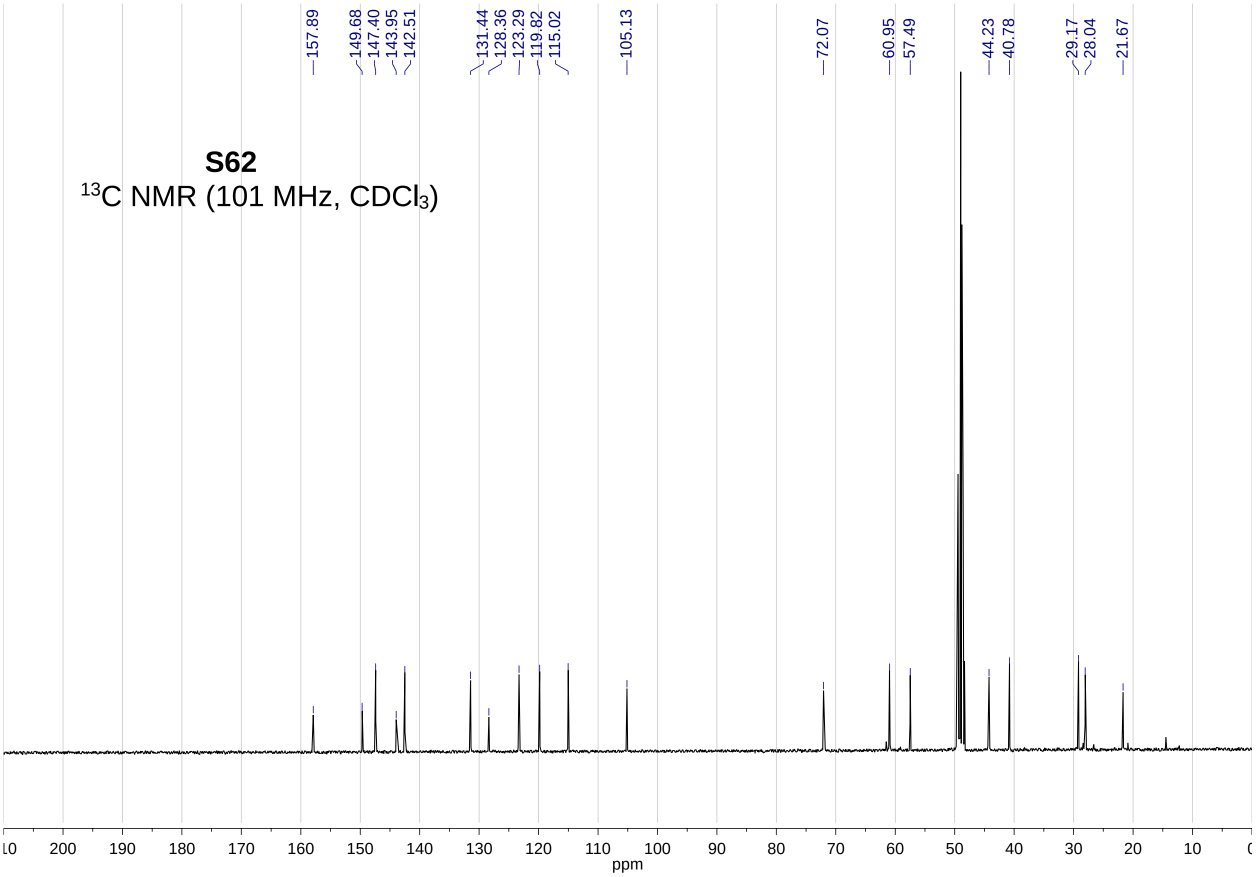
S62
^{13}C NMR (101 MHz, CDCl$_3$)
157.89
149.68
147.40
143.95
142.51
131.44
128.36
123.29
119.82
115.02
105.13
72.07
60.95
57.49
44.23
40.78
29.17
28.04
21.67
ppm

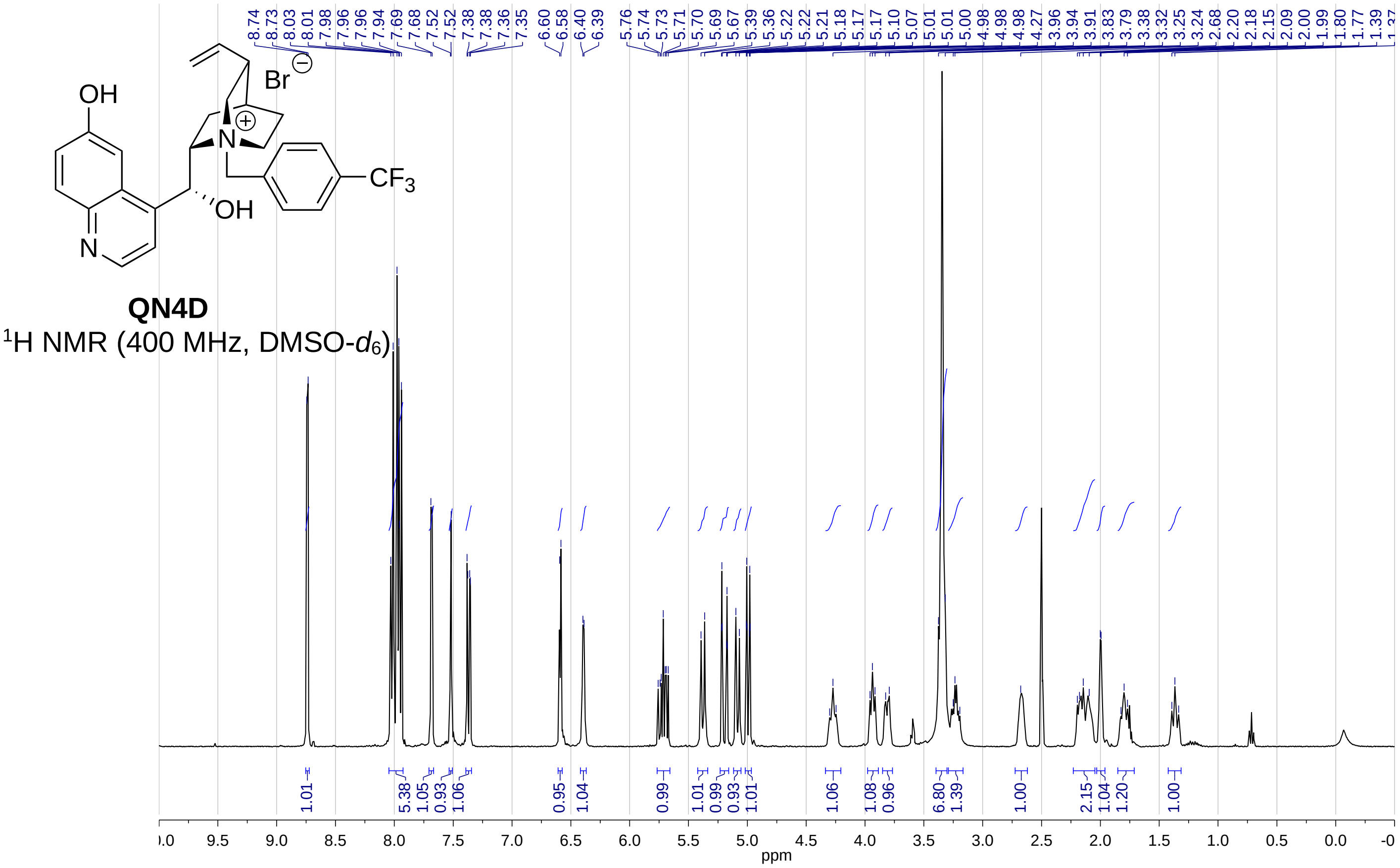

QN4D

^1H NMR (400 MHz, DMSO-d_6)

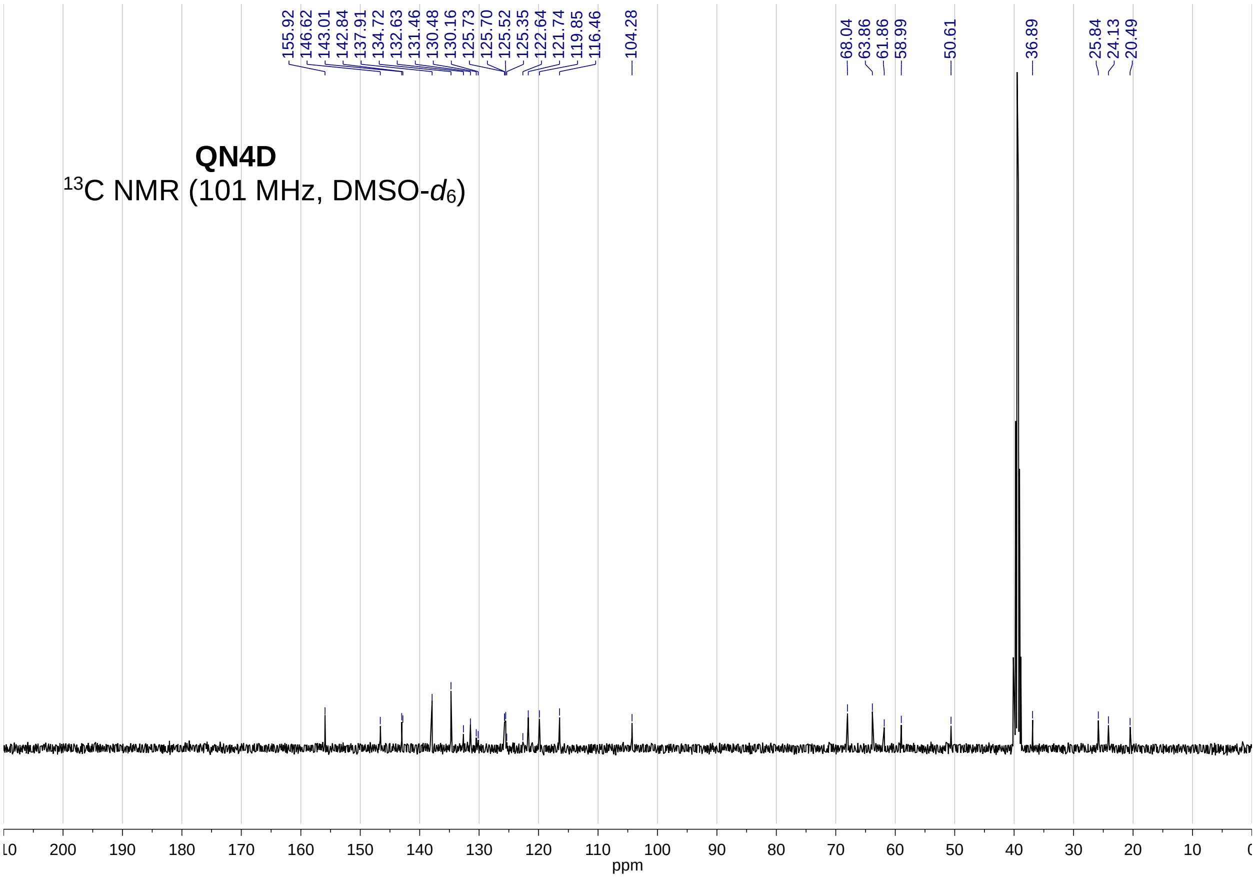

QN4D

^{13}C NMR (101 MHz, DMSO-d_6)

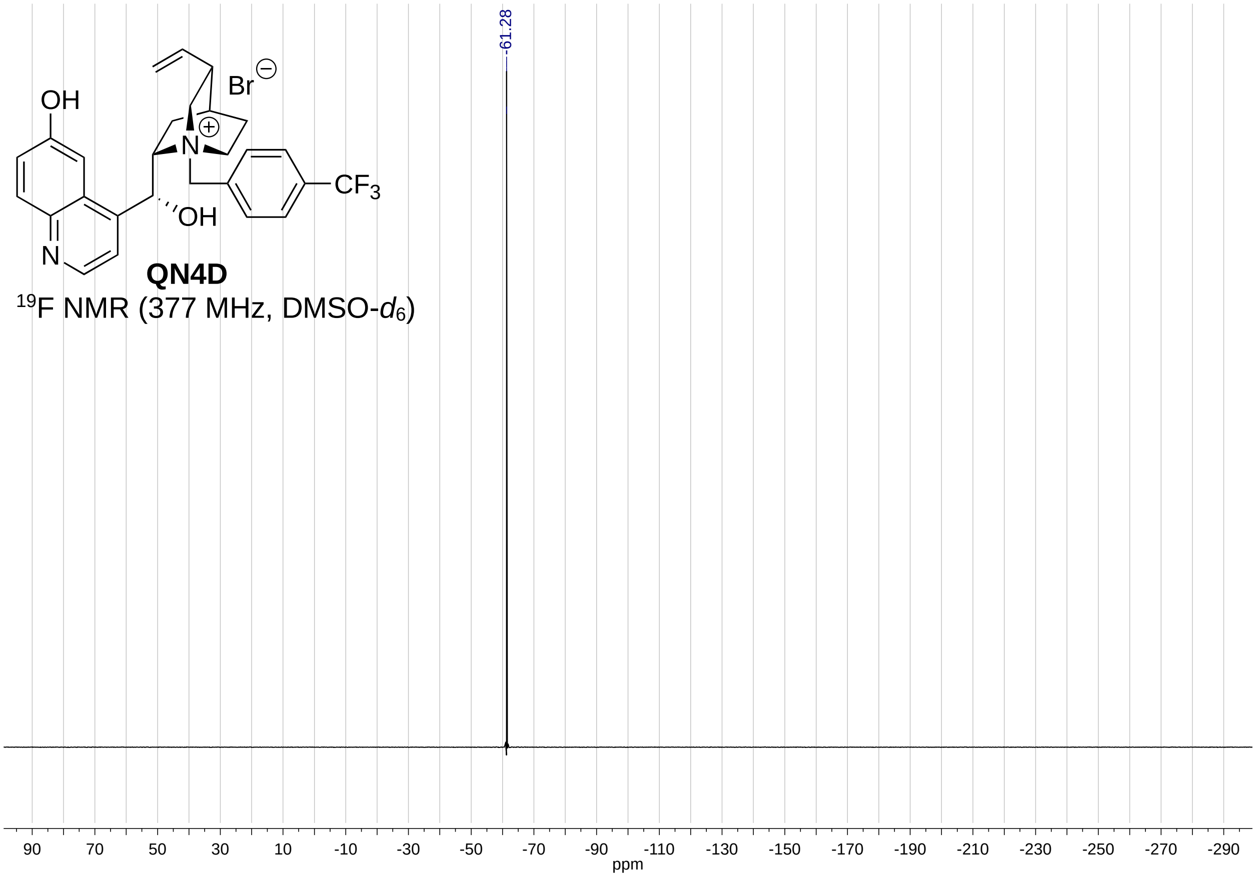
OH
Br
N
CF3
OH
N
QN4D
19F NMR (377 MHz, DMSO-d6)
-61.28
90
70
50
30
10
-10
-30
-50
-70
-90
-110
-130
-150
-170
-190
-210
-230
-250
-270
-290
ppm

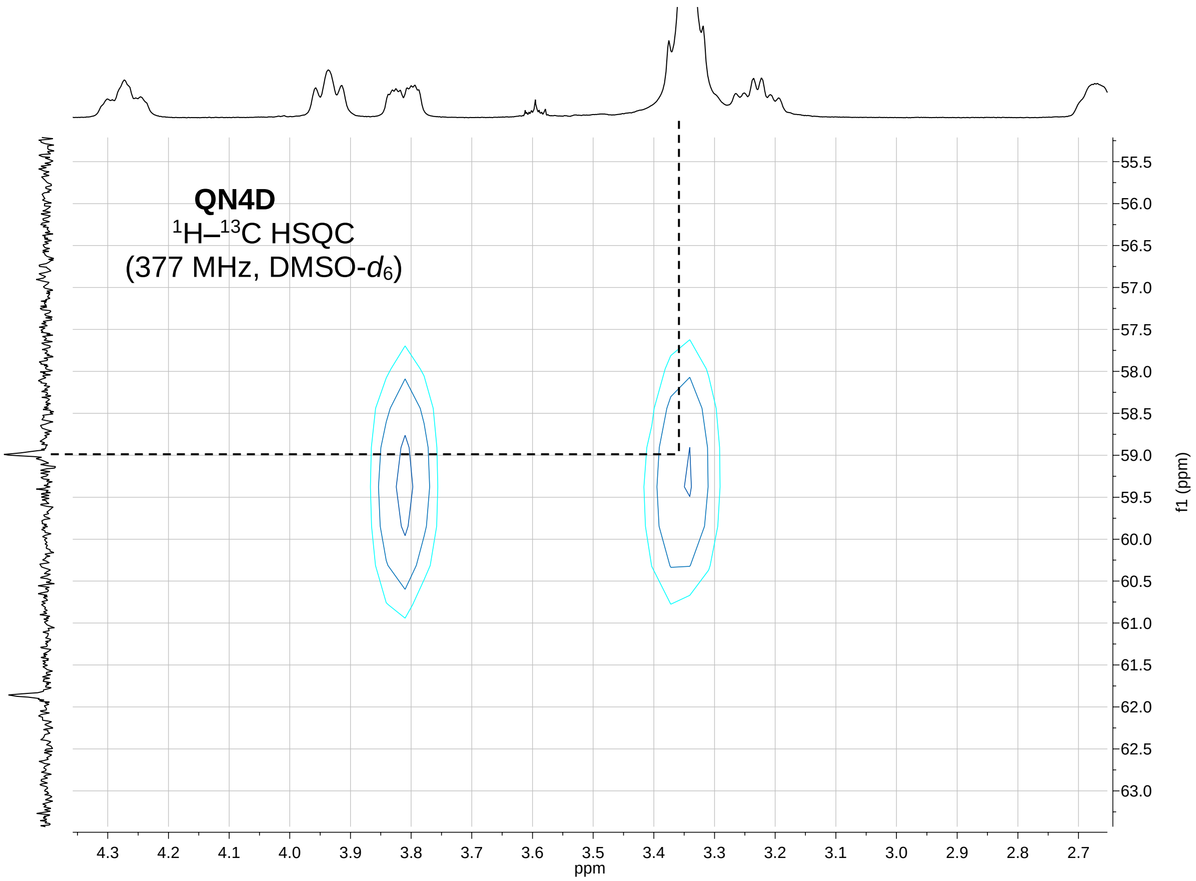
QN4D
1H–13C HSQC
(377 MHz, DMSO-d6)
55.5
56.0
56.5
57.0
57.5
58.0
58.5
59.0
59.5
60.0
60.5
61.0
61.5
62.0
62.5
63.0
f1 (ppm)
4.3
4.2
4.1
4.0
3.9
3.8
3.7
3.6
3.5
3.4
3.3
3.2
3.1
3.0
2.9
2.8
2.7
ppm

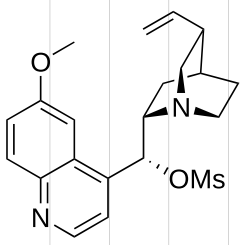

S63

^{1}H NMR (400 MHz, $CDCl_3$)

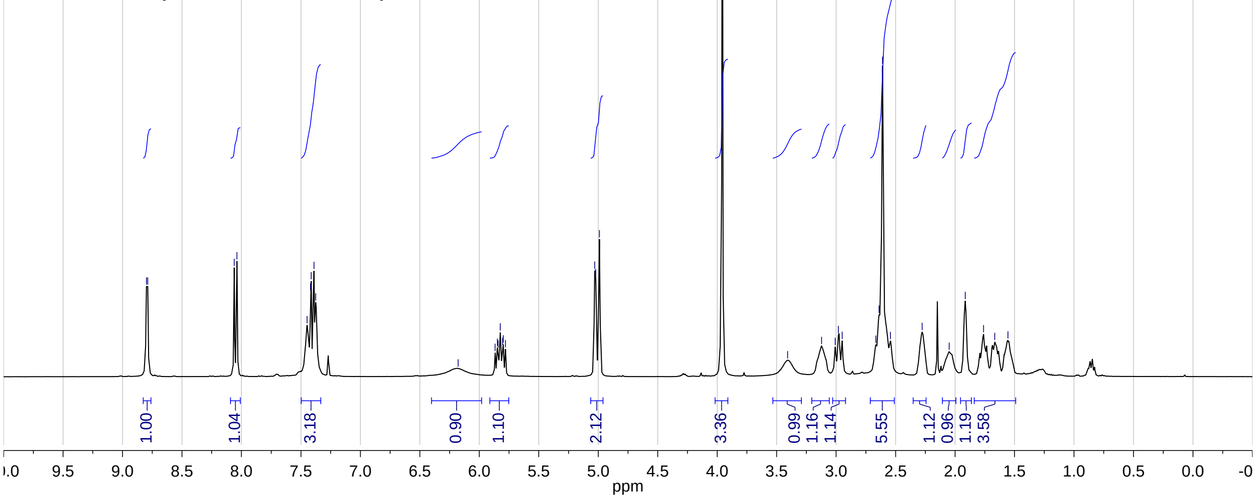

S63

^{13}C NMR (101 MHz, $CDCl_3$)

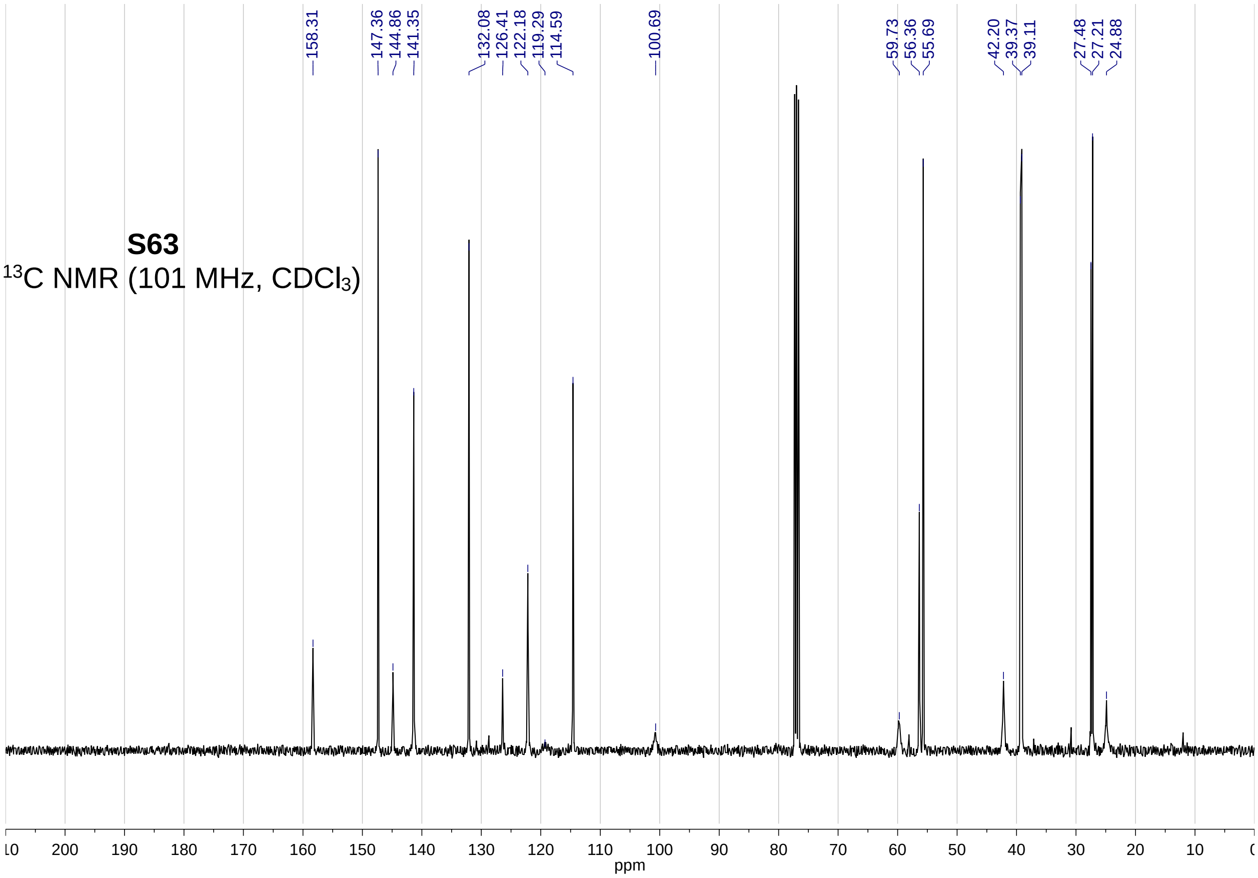

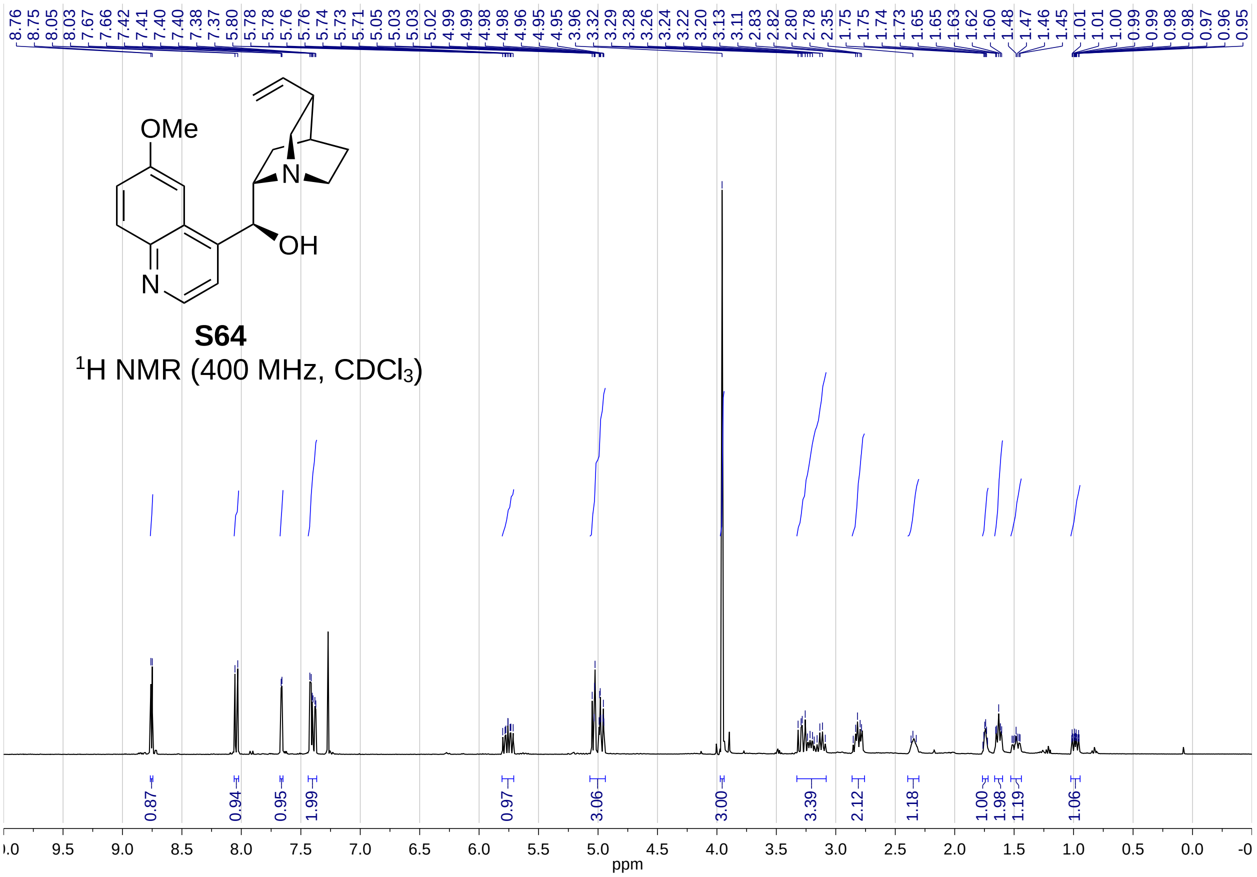

S64
^{1}H NMR (400 MHz, $CDCl_3$)

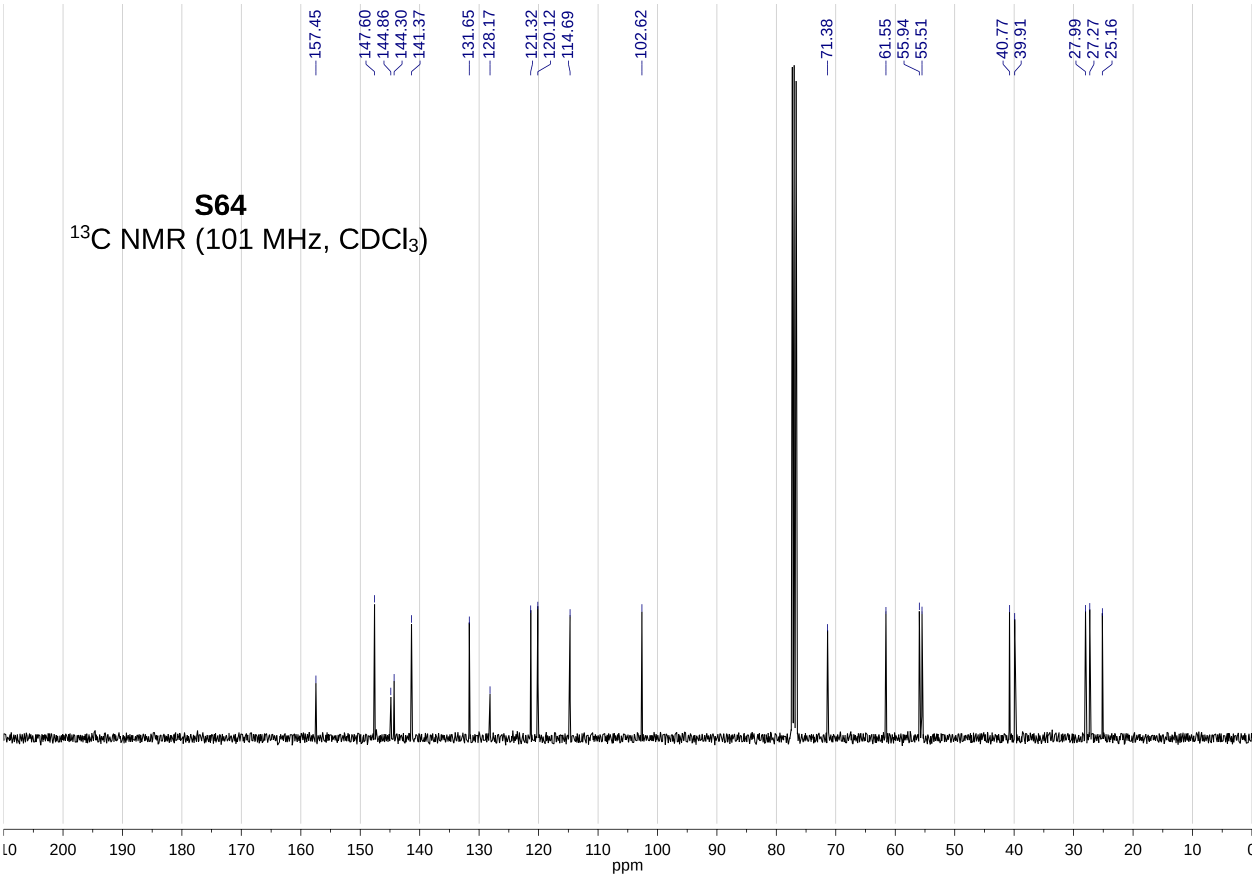

S64
^{13}C NMR (101 MHz, $CDCl_3$)

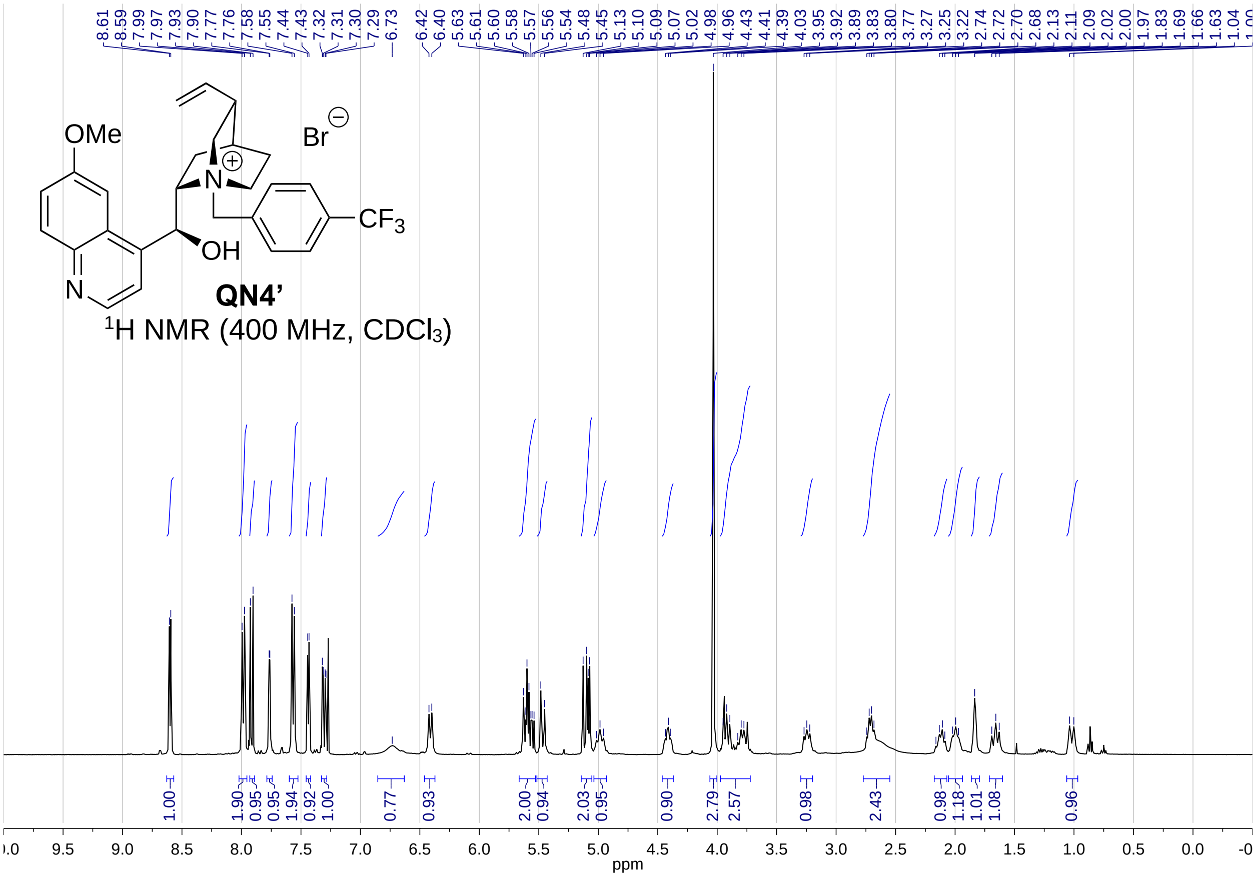

OMe
Br
N
OH
CF3
N
QN4'
1H NMR (400 MHz, CDCl3)
ppm

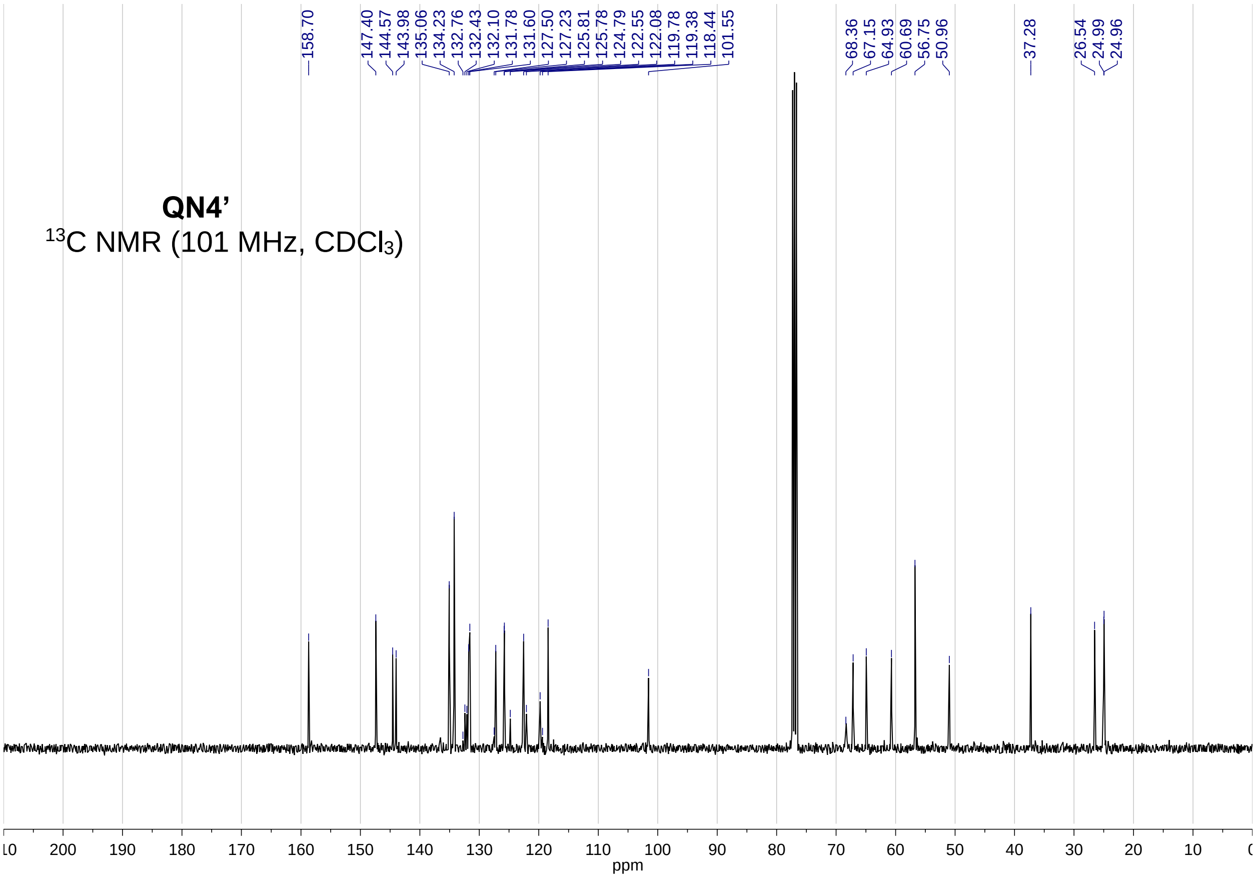

QN4'
13C NMR (101 MHz, CDCl3)
ppm

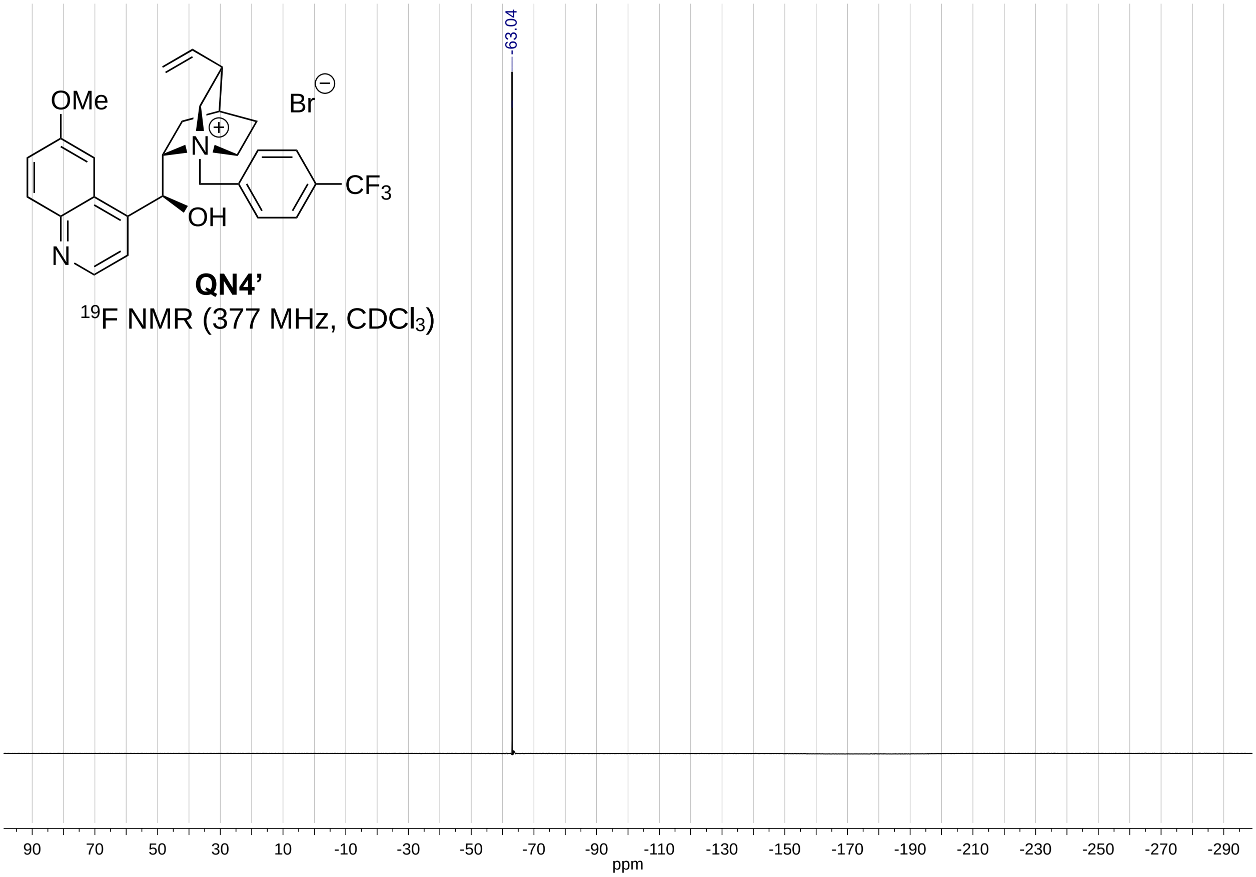

QN4'

^{19}F NMR (377 MHz, $CDCl_3$)

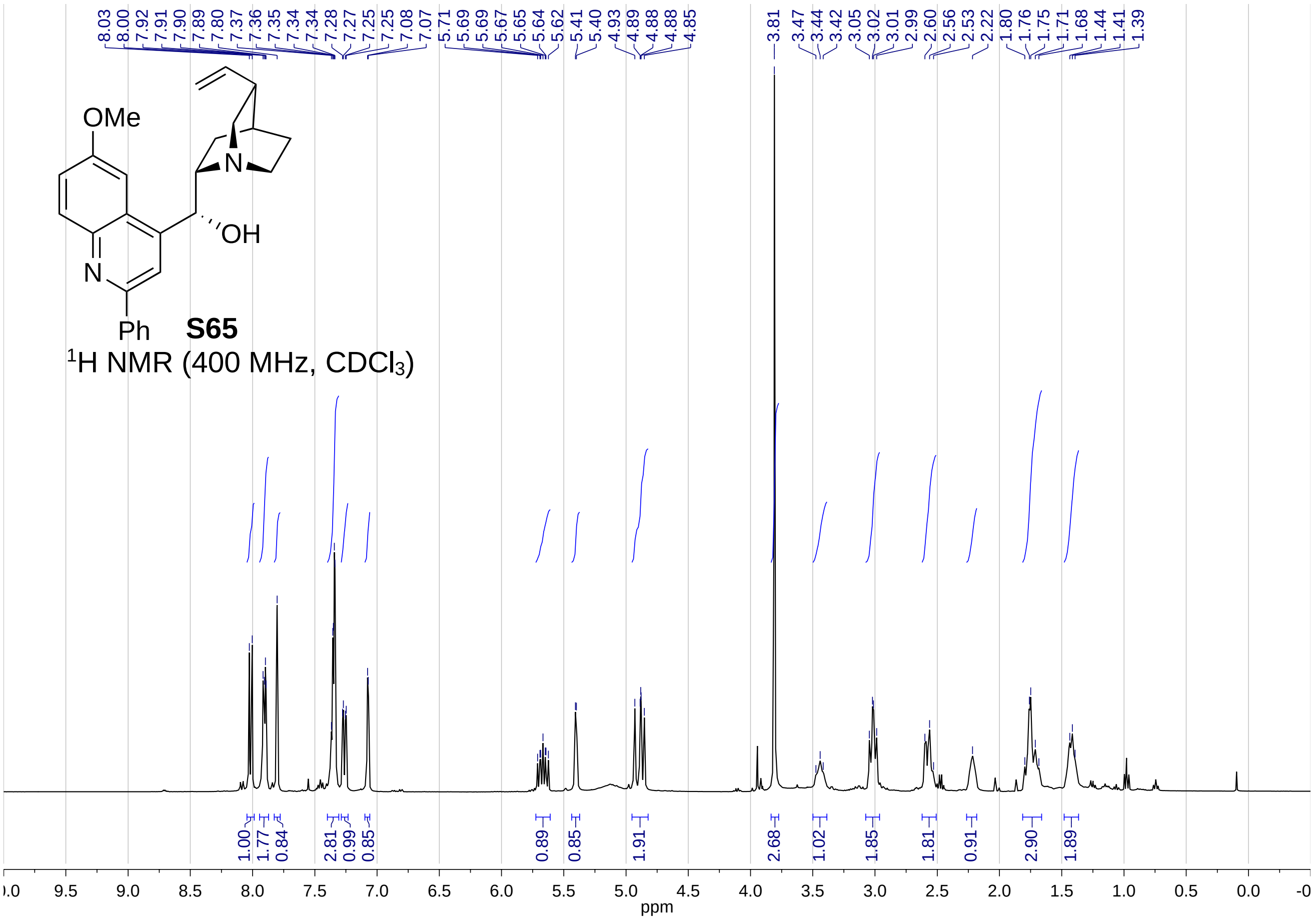
8.03
8.00
7.92
7.91
7.90
7.89
7.80
7.37
7.36
7.35
7.34
7.34
7.28
7.27
7.25
7.25
7.08
7.07
5.71
5.69
5.69
5.67
5.65
5.64
5.62
5.41
5.40
4.93
4.89
4.88
4.88
4.85
3.81
3.47
3.44
3.42
3.05
3.02
3.01
2.99
2.60
2.56
2.53
2.22
1.80
1.76
1.75
1.71
1.68
1.44
1.41
1.39
OMe
N
OH
N
Ph
S65
¹H NMR (400 MHz, CDCl₃)
1.00
1.77
0.84
2.81
0.99
0.85
0.89
0.85
1.91
2.68
1.02
1.85
1.81
0.91
2.90
1.89
ppm

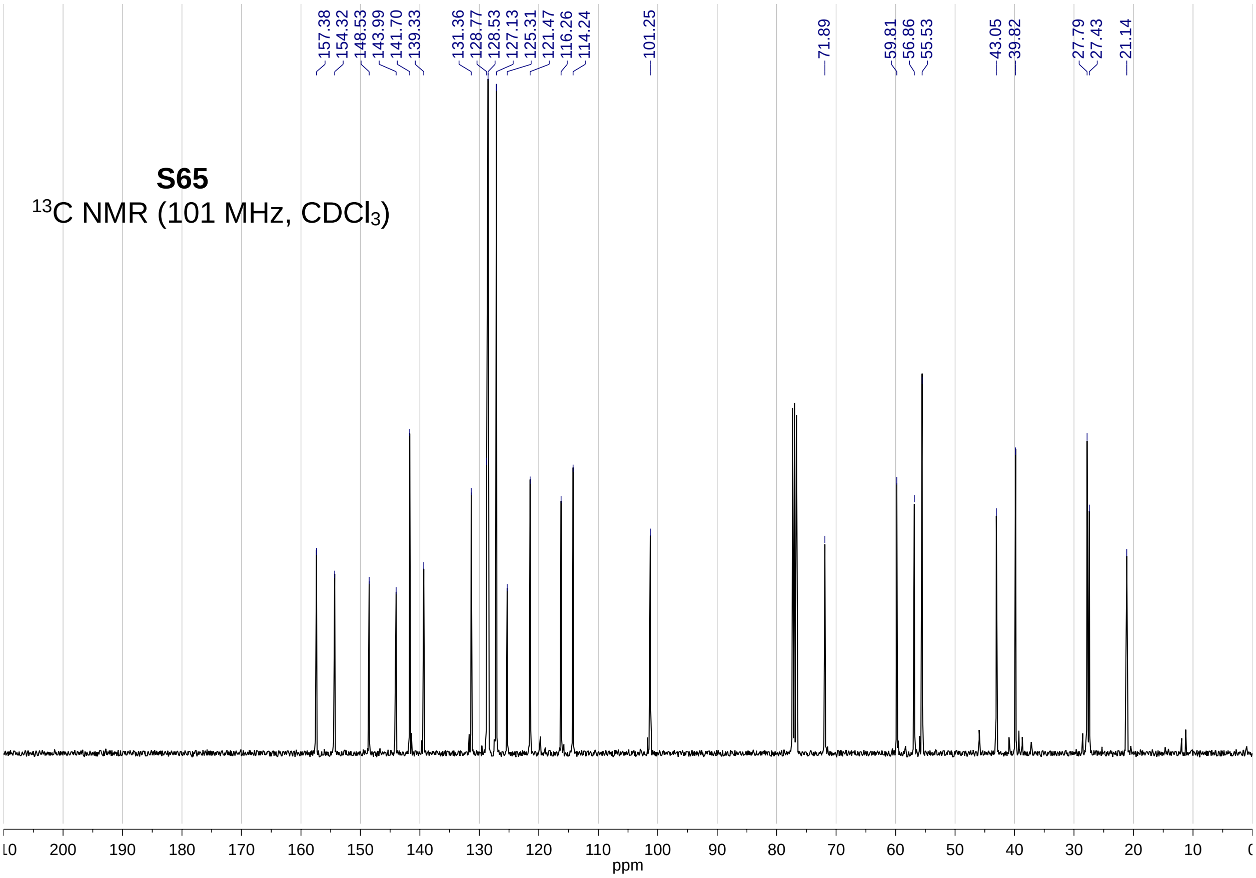
157.38
154.32
148.53
143.99
141.70
139.33
131.36
128.77
128.53
127.13
125.31
121.47
116.26
114.24
101.25
71.89
59.81
56.86
55.53
43.05
39.82
27.79
27.43
21.14
S65
¹³C NMR (101 MHz, CDCl₃)
ppm

QN4E/Cat4

^{1}H NMR (400 MHz, $CDCl_3$)

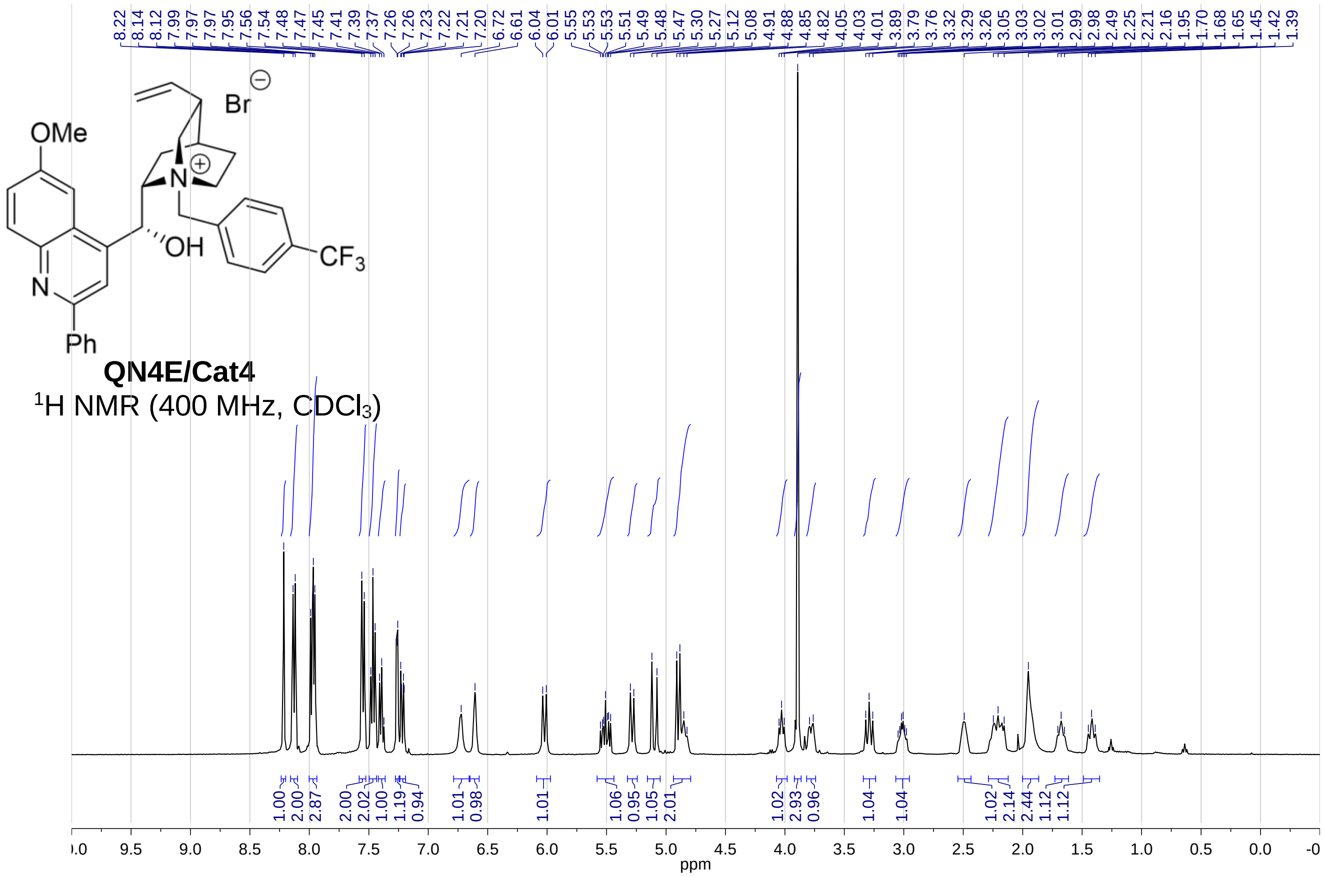

QN4E/Cat4

^{13}C NMR (101 MHz, $CDCl_3$)

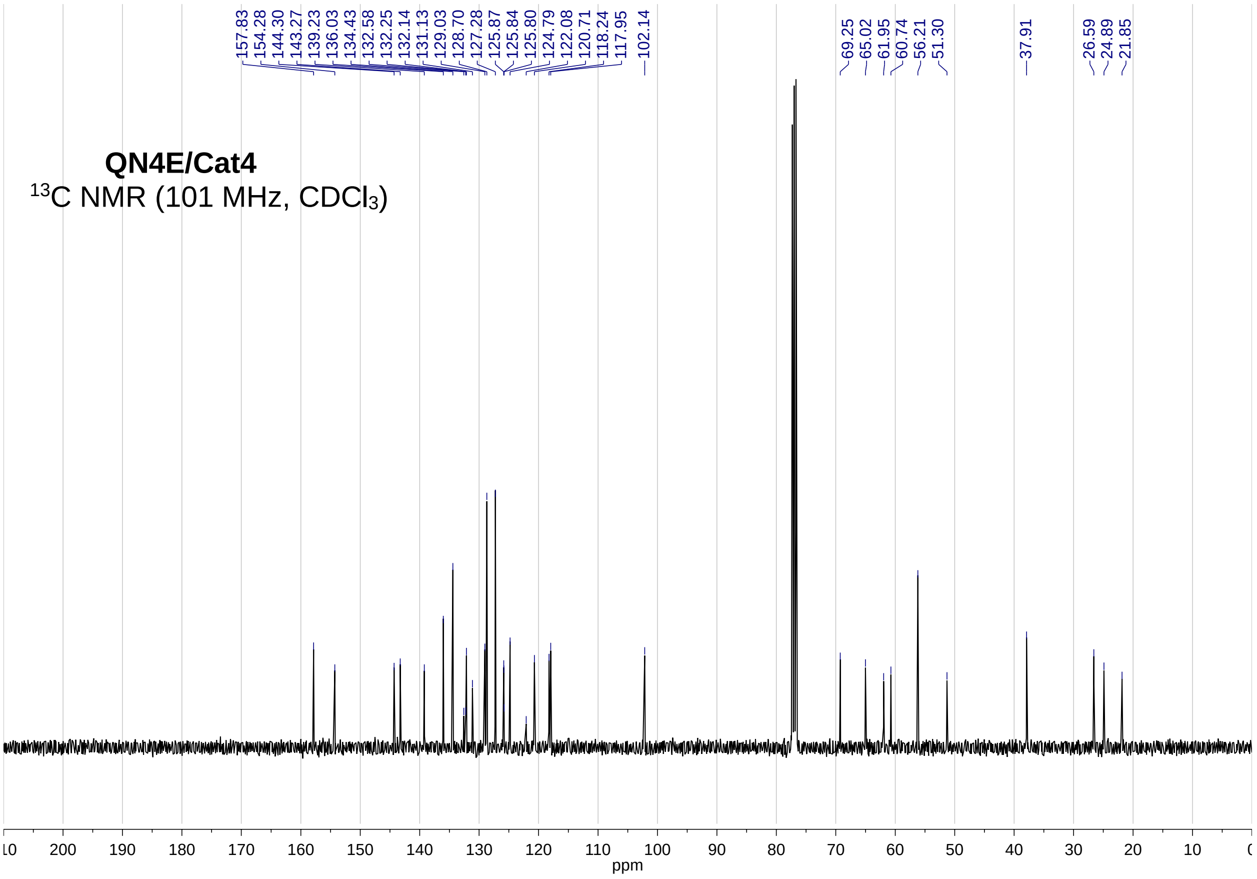

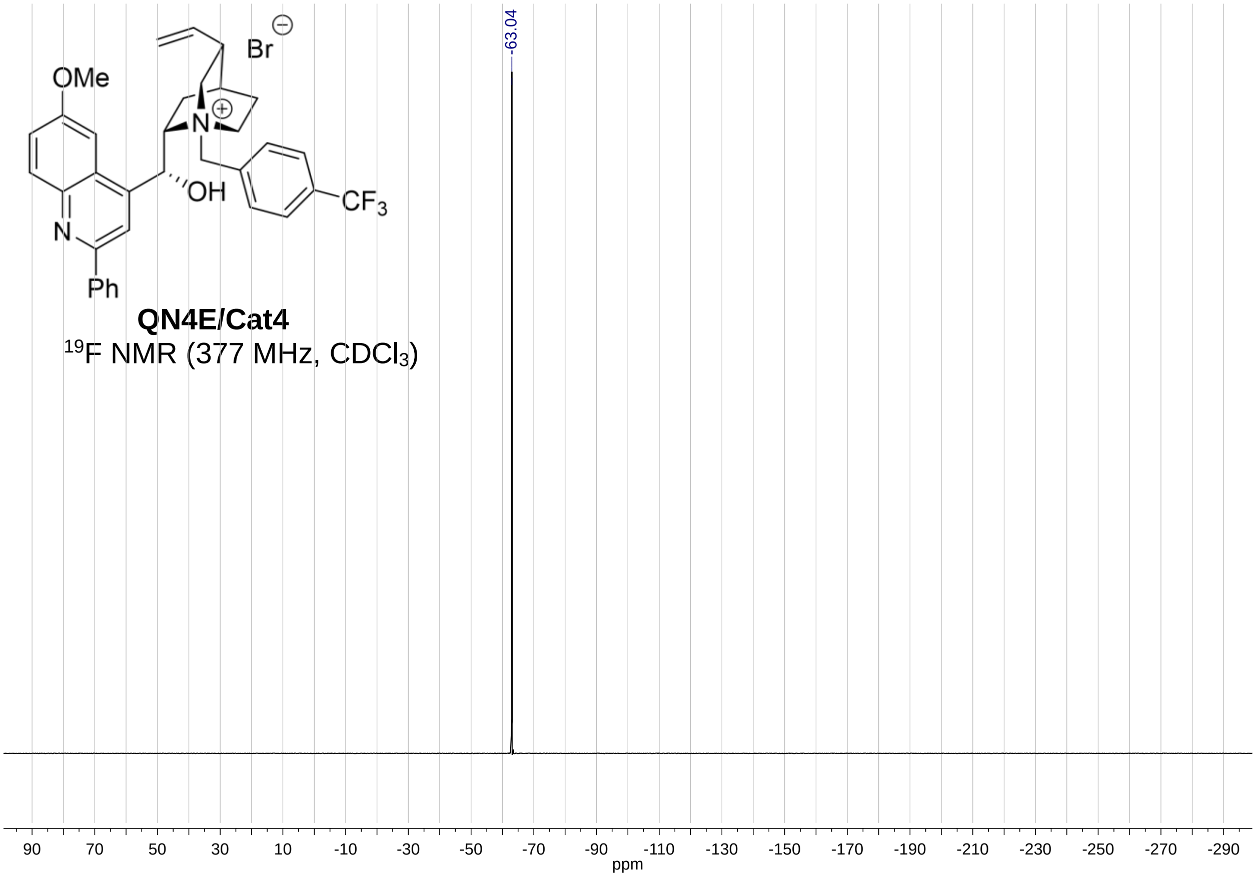

QN4E/Cat4

^{19}F NMR (377 MHz, $CDCl_3$)

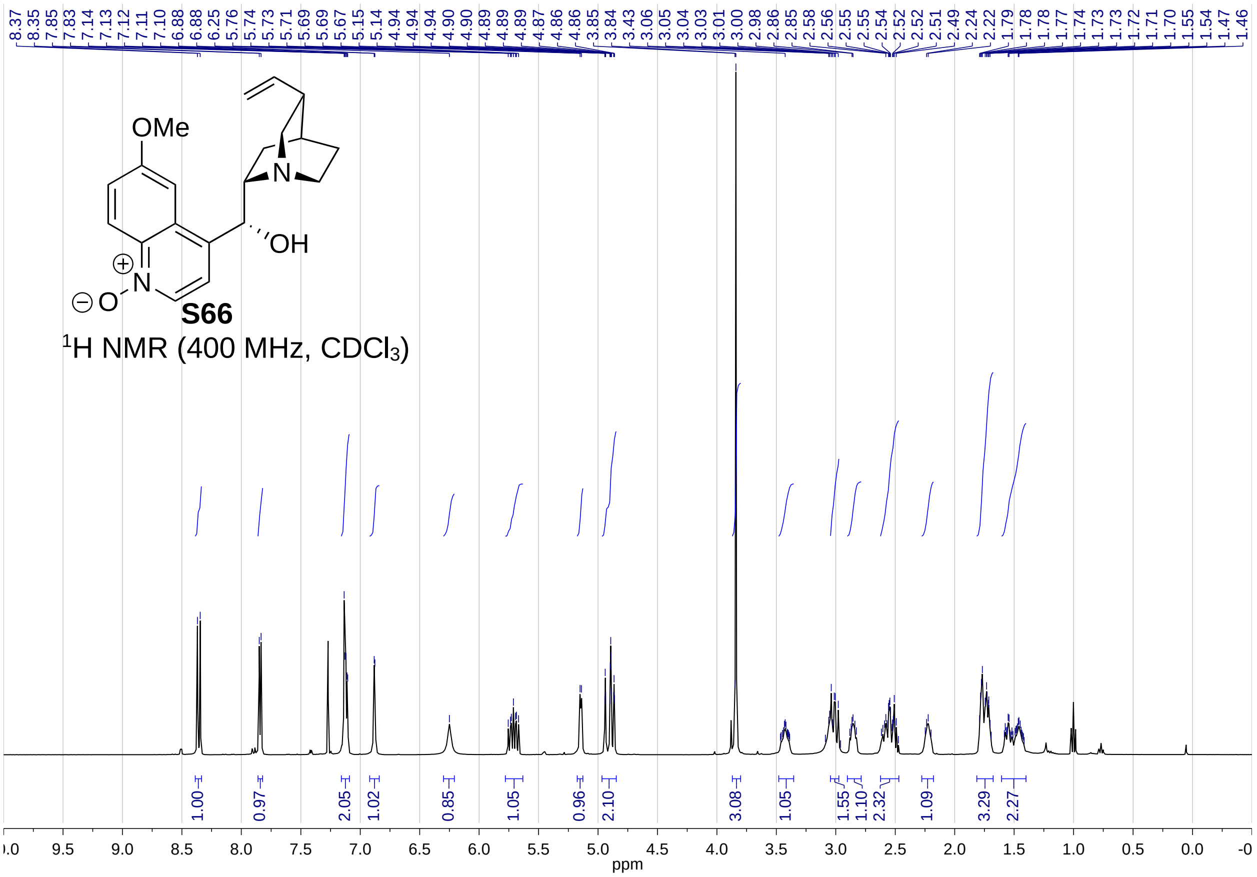

S66

^{1}H NMR (400 MHz, $CDCl_3$)

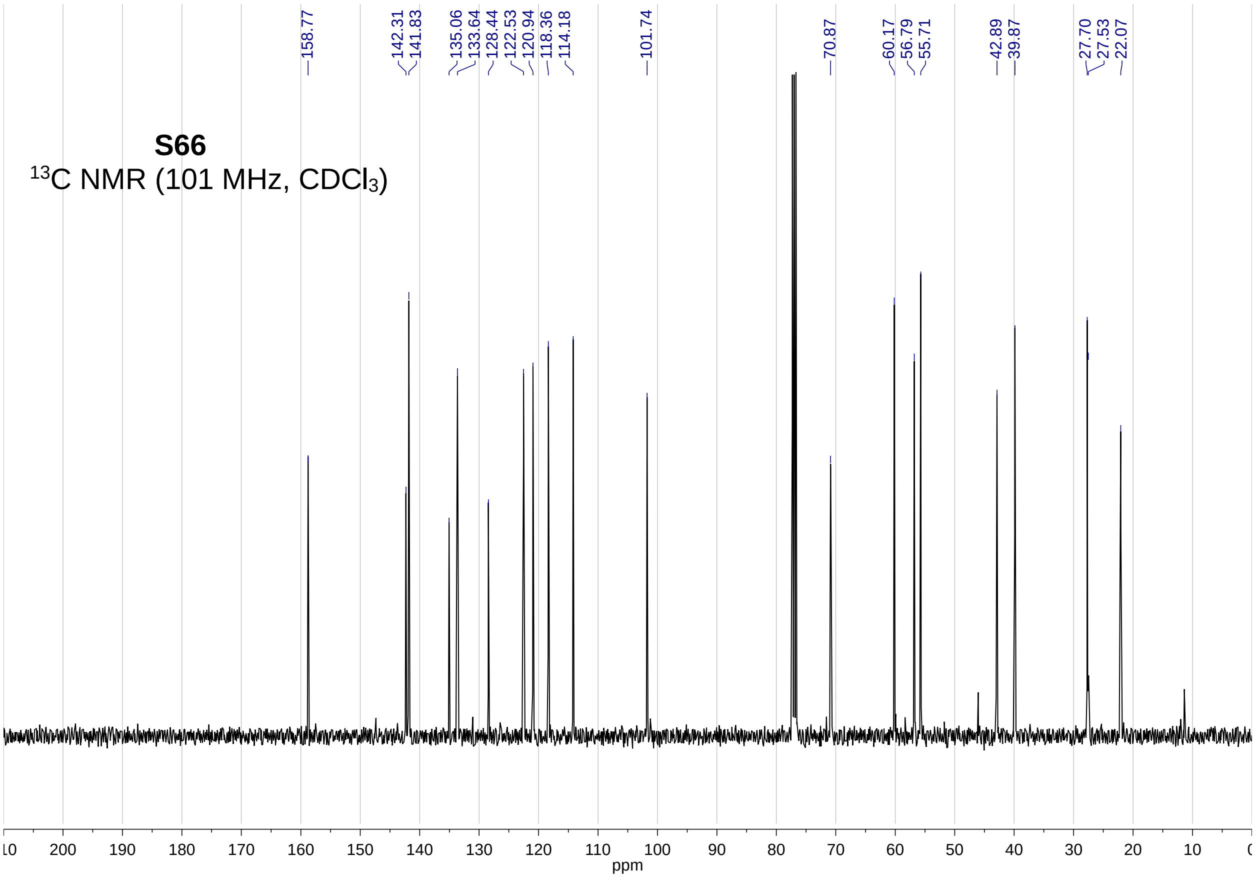

S66

^{13}C NMR (101 MHz, $CDCl_3$)

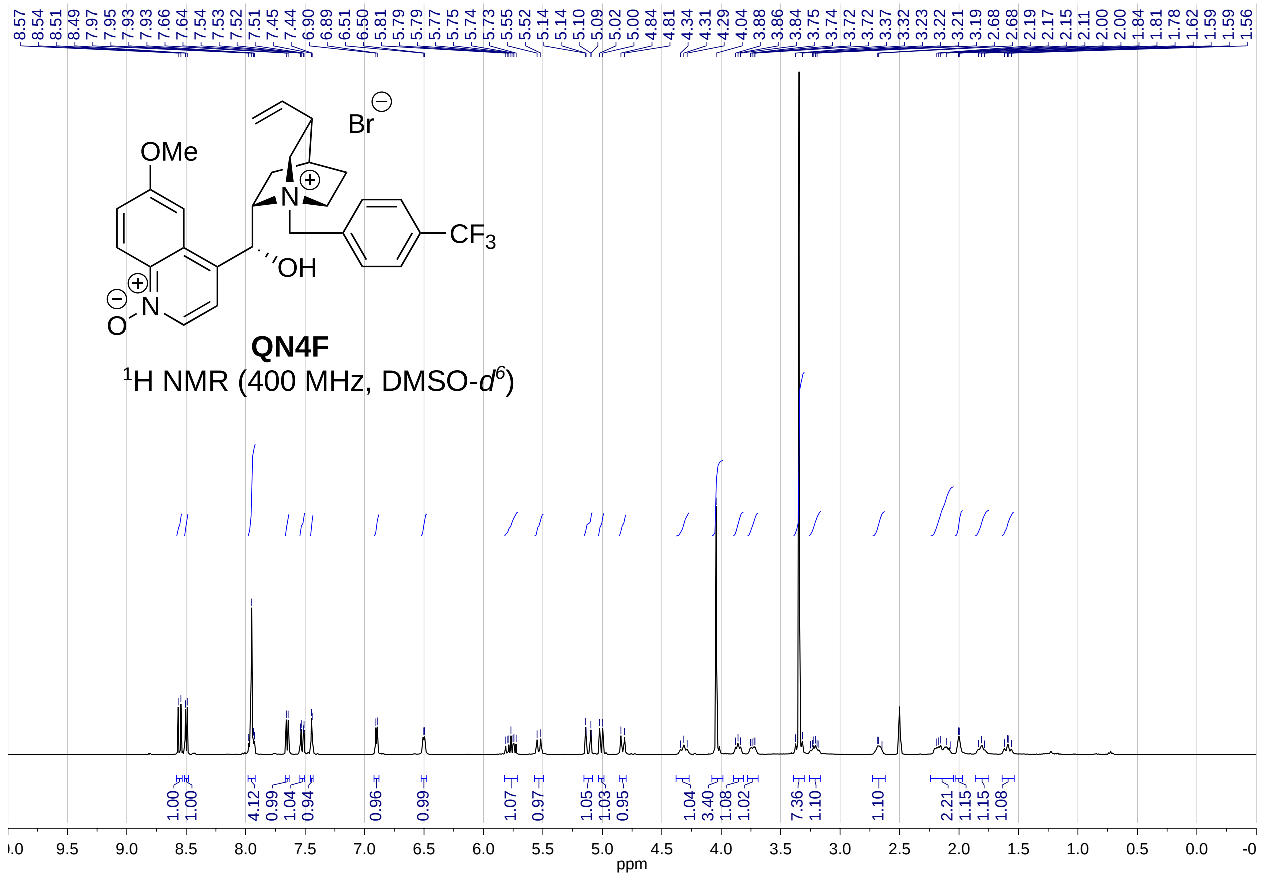
OMe
Br
N
OH
CF3
N
O
QN4F
1H NMR (400 MHz, DMSO-d6)
ppm

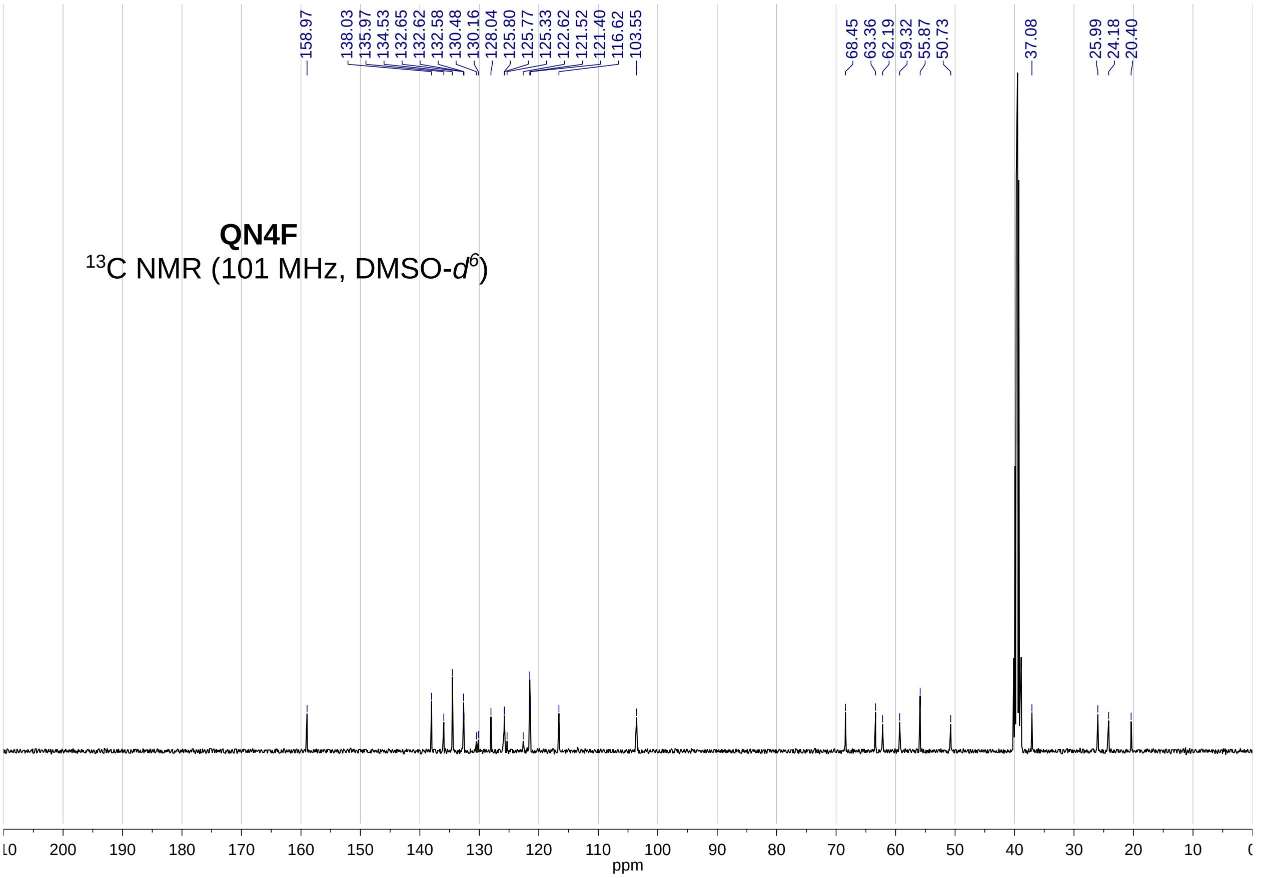
QN4F
13C NMR (101 MHz, DMSO-d6)
ppm

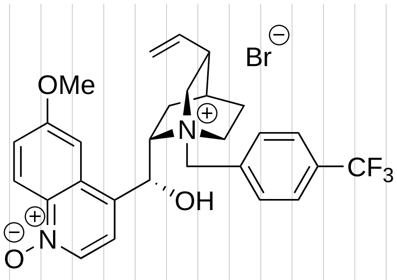

QN4F

^{19}F NMR (377 MHz, DMSO-d^6)

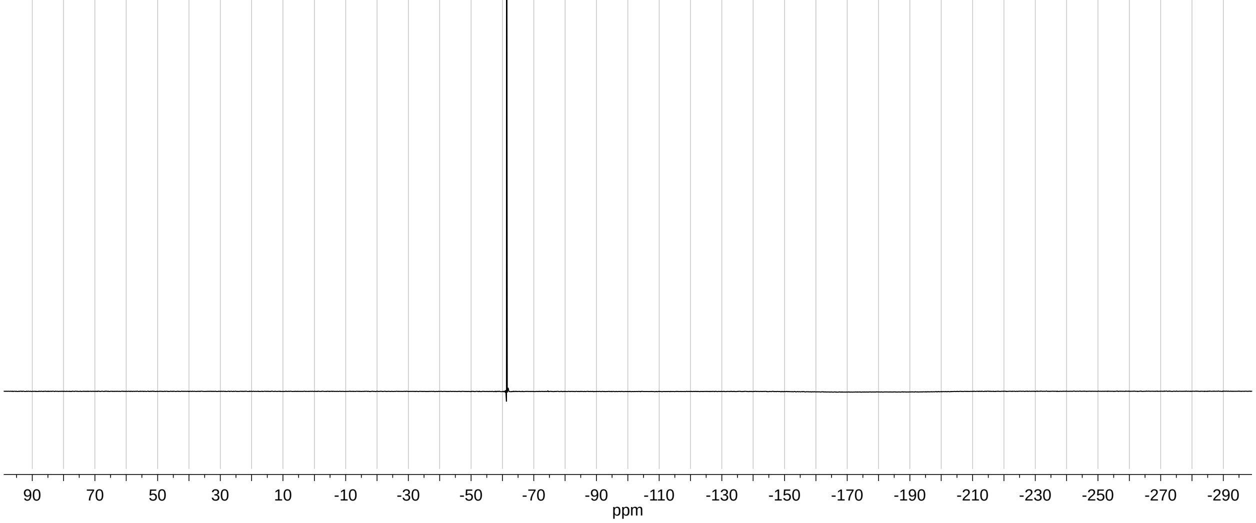

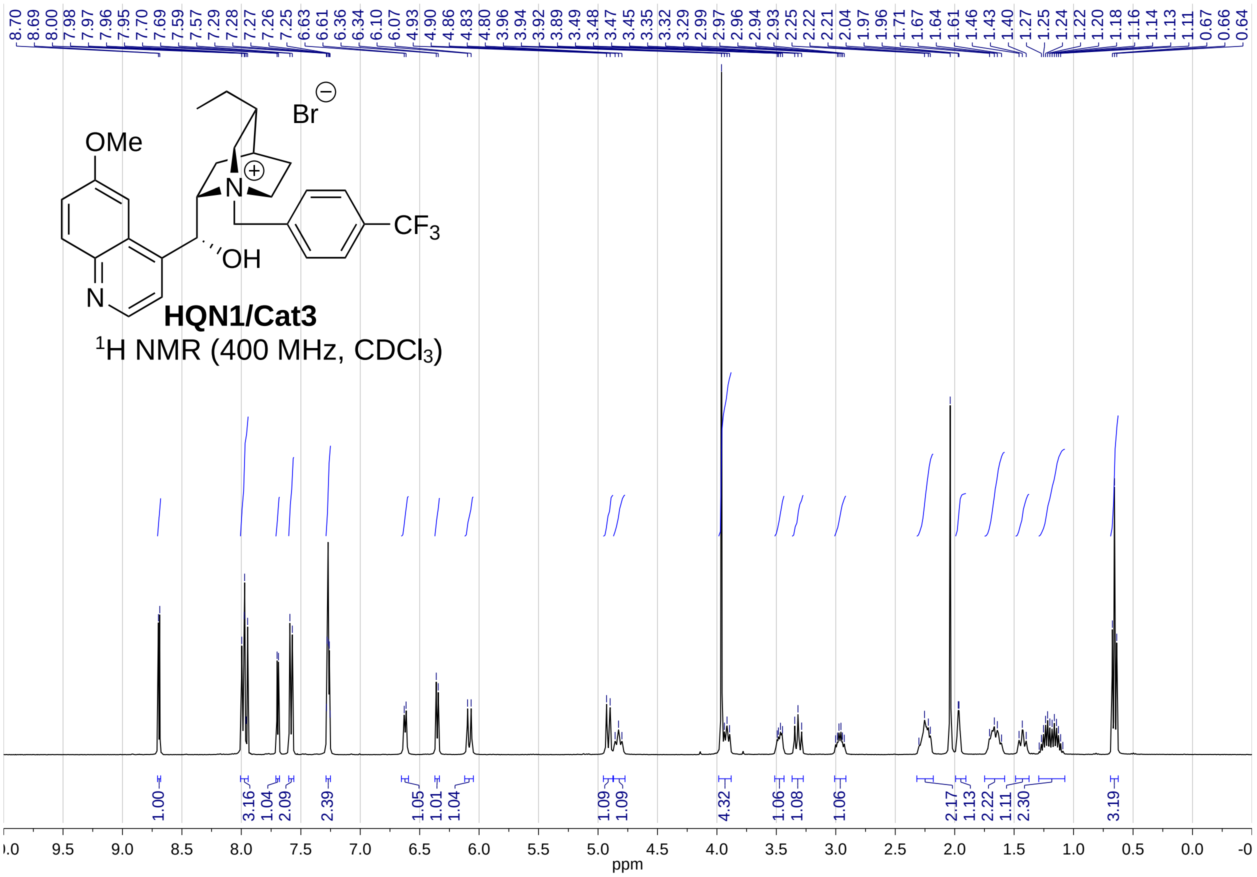
OMe
Br
N
N
CF3
OH
HQN1/Cat3
1H NMR (400 MHz, CDCl3)
ppm

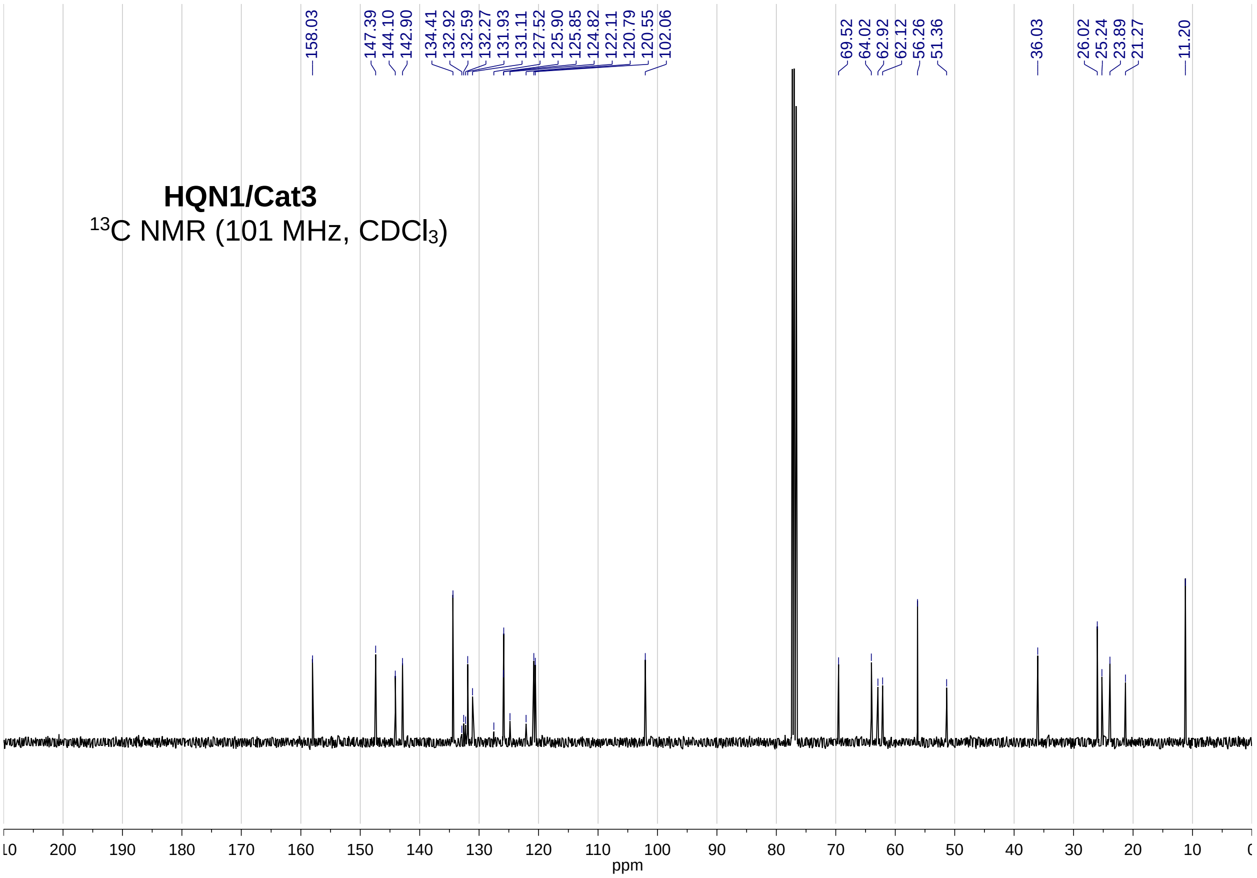
HQN1/Cat3
13C NMR (101 MHz, CDCl3)
ppm

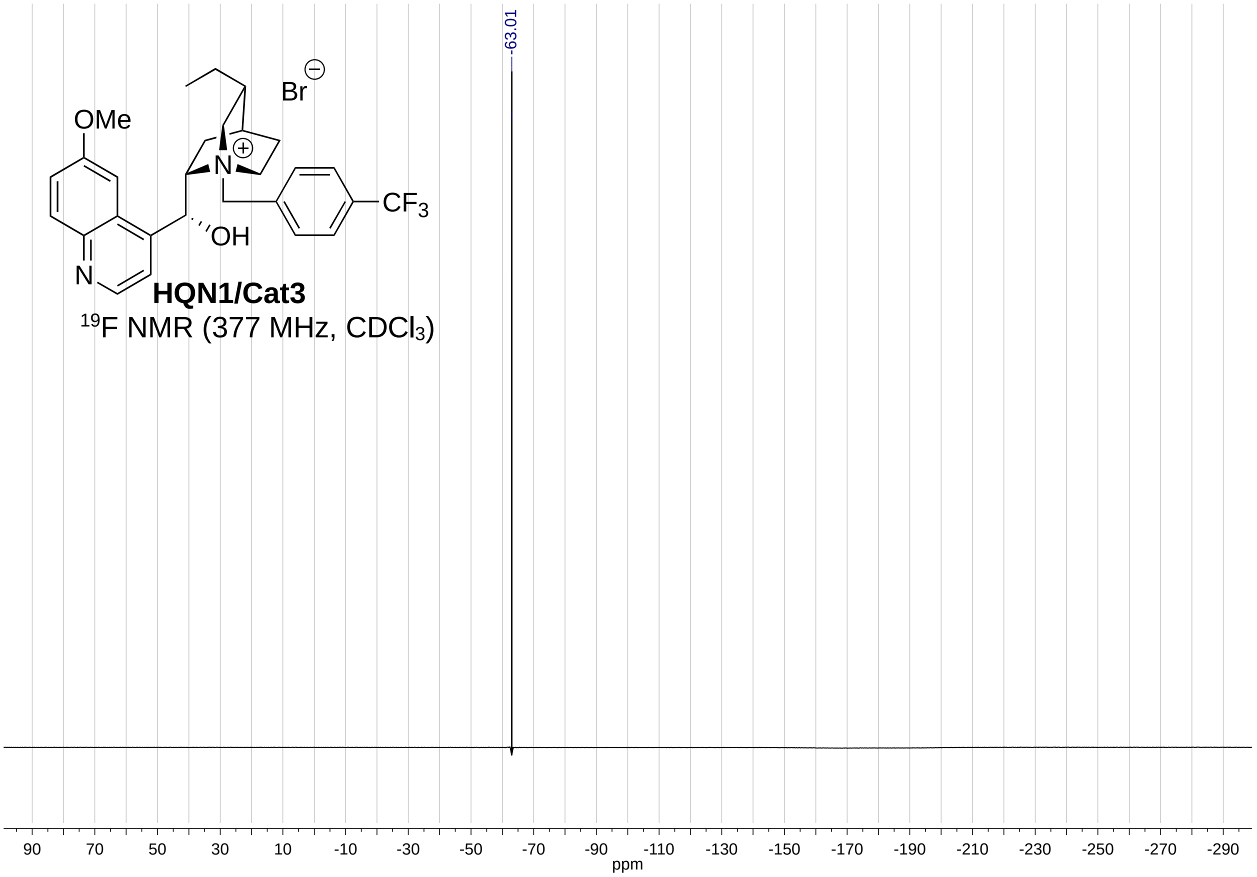

HQN1/Cat3

^{19}F NMR (377 MHz, $CDCl_3$)

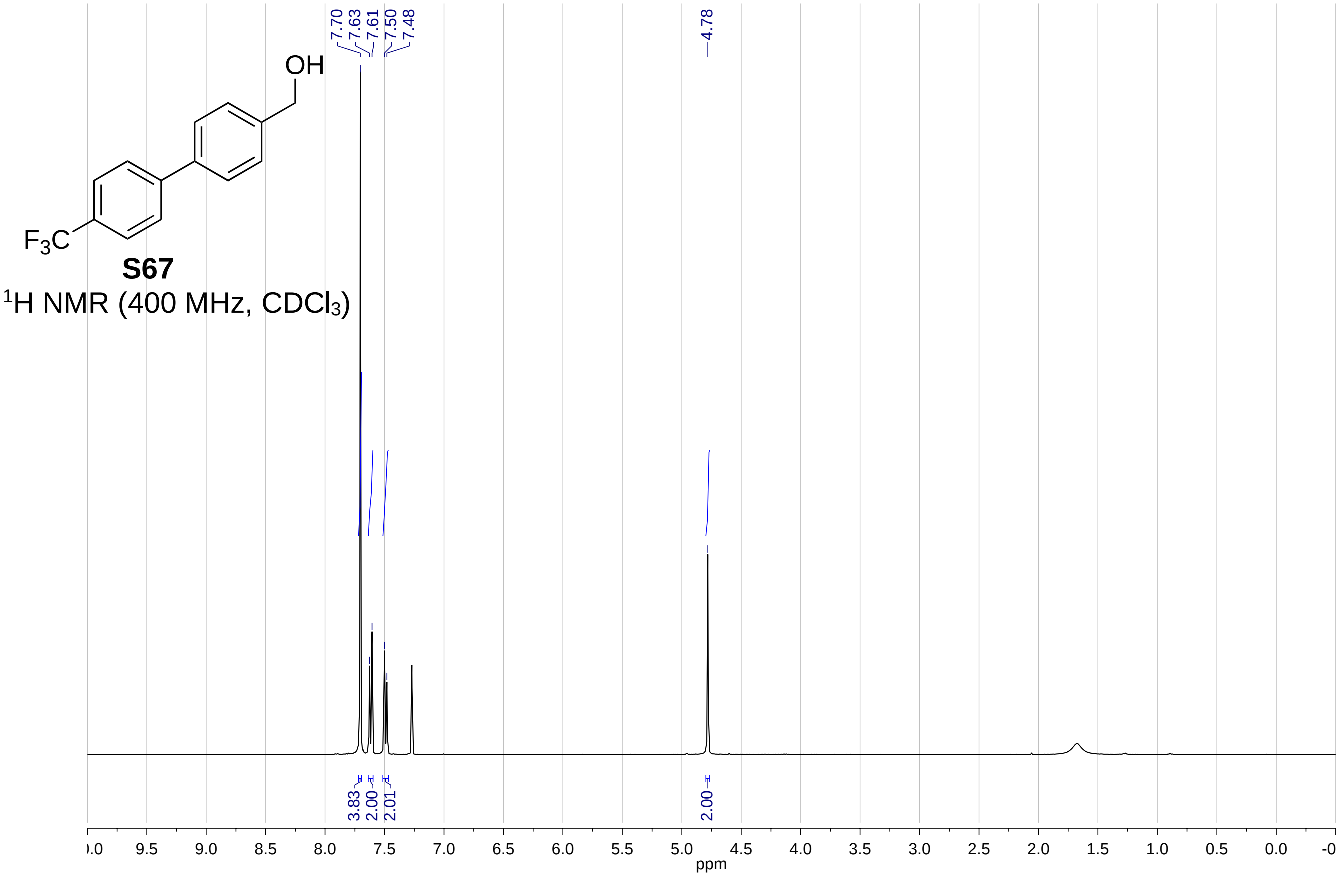

S67

^{1}H NMR (400 MHz, $CDCl_3$)

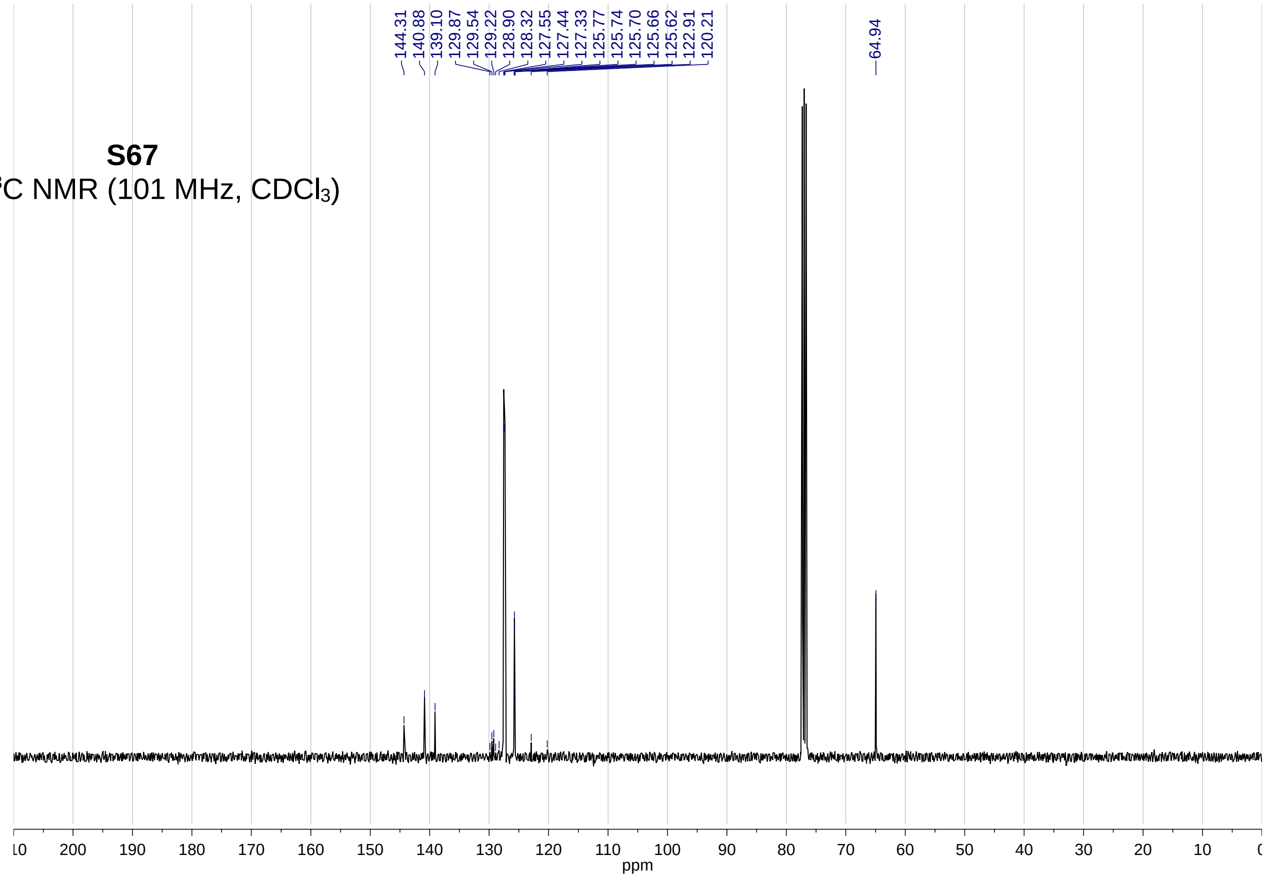

S67

^{13}C NMR (101 MHz, $CDCl_3$)

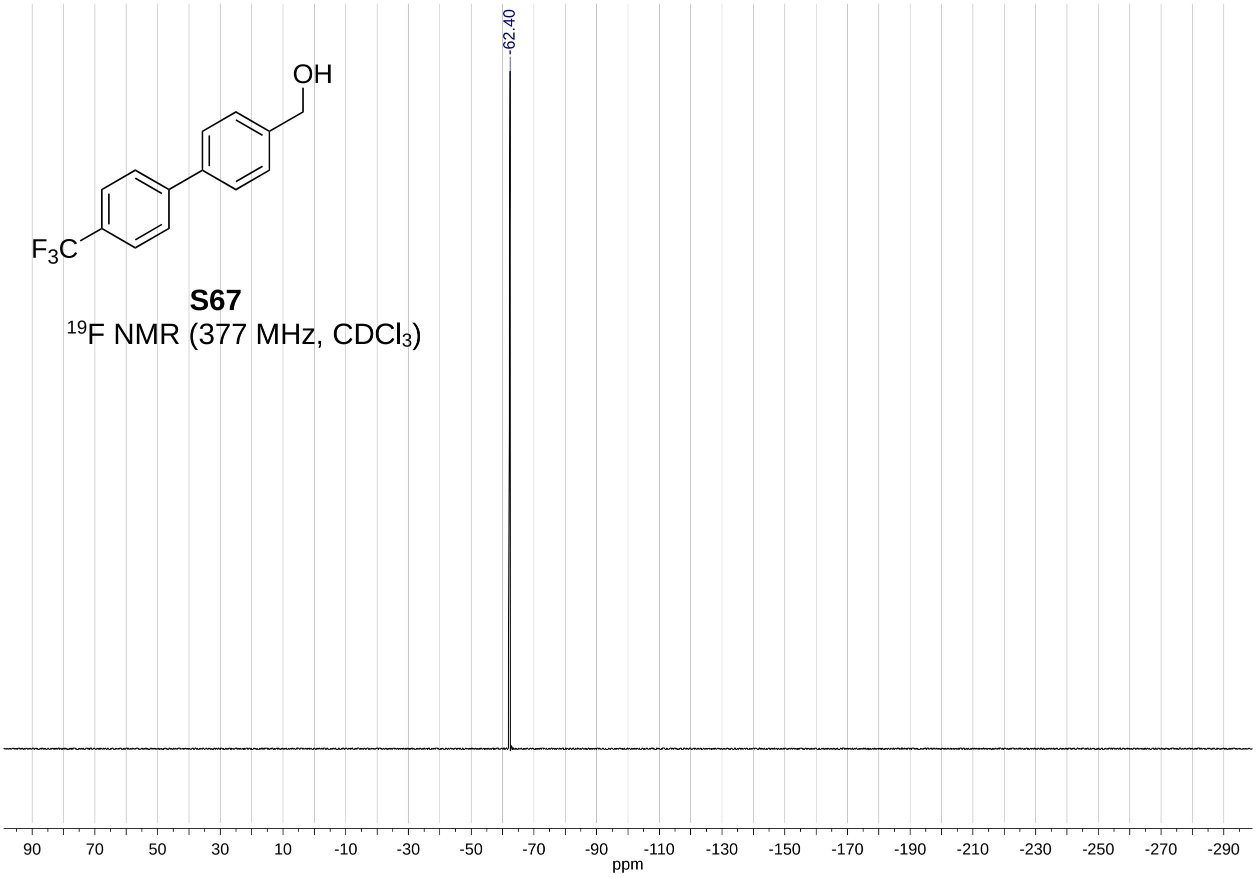
-62.40
OH
F_3C
S67
19F NMR (377 MHz, CDCl3)
90
70
50
30
10
-10
-30
-50
-70
-90
-110
-130
-150
-170
-190
-210
-230
-250
-270
-290
ppm

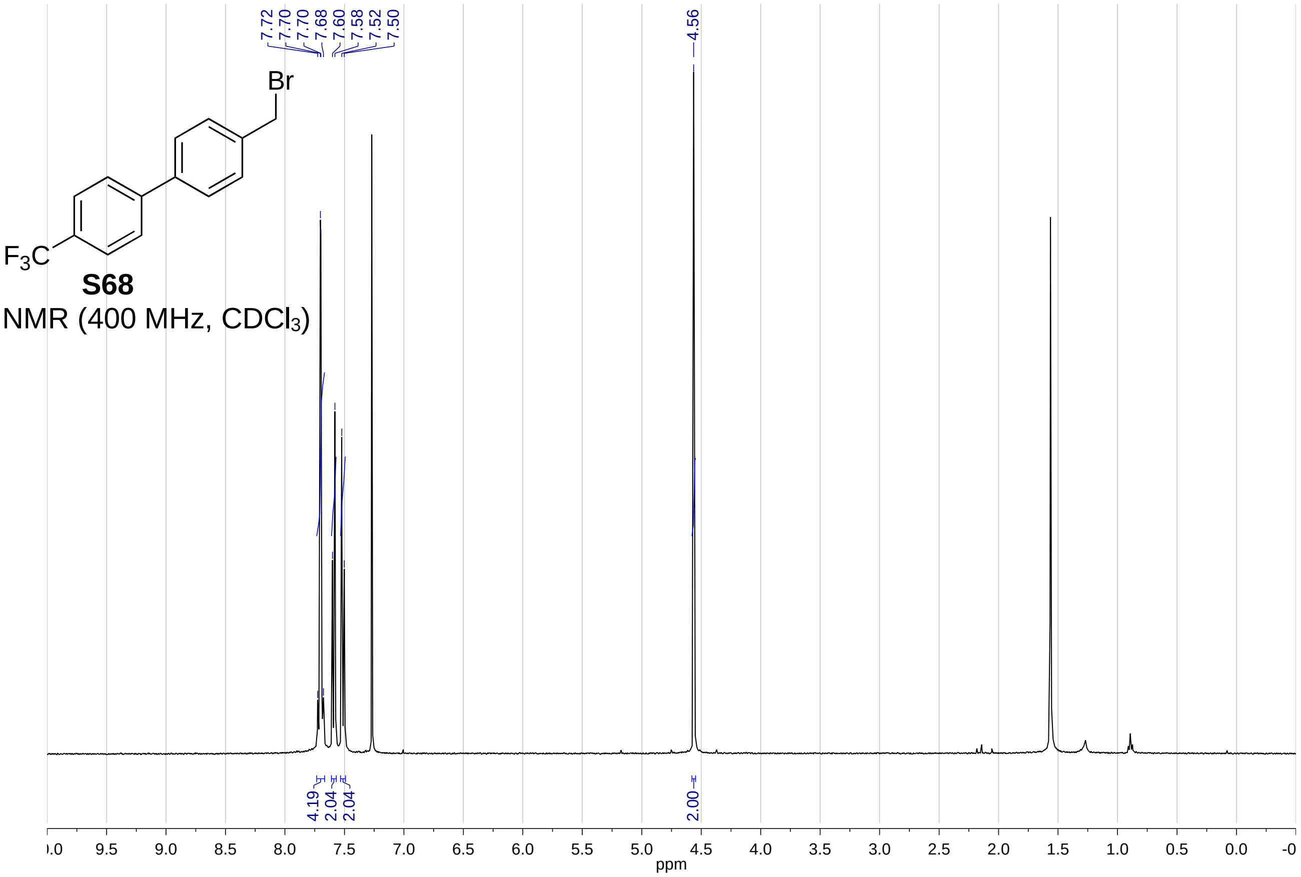

S68
^{1}H NMR (400 MHz, $CDCl_3$)

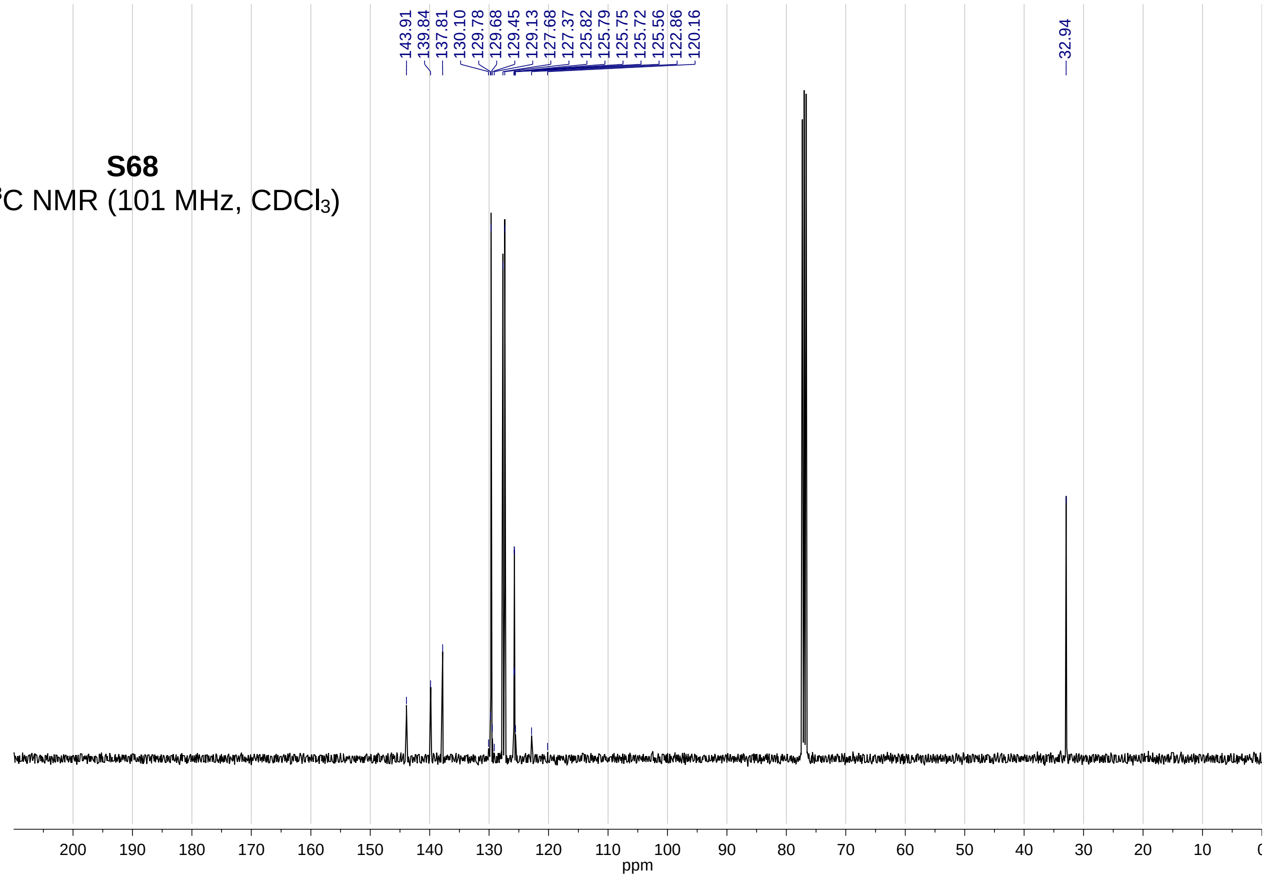

S68
^{13}C NMR (101 MHz, $CDCl_3$)

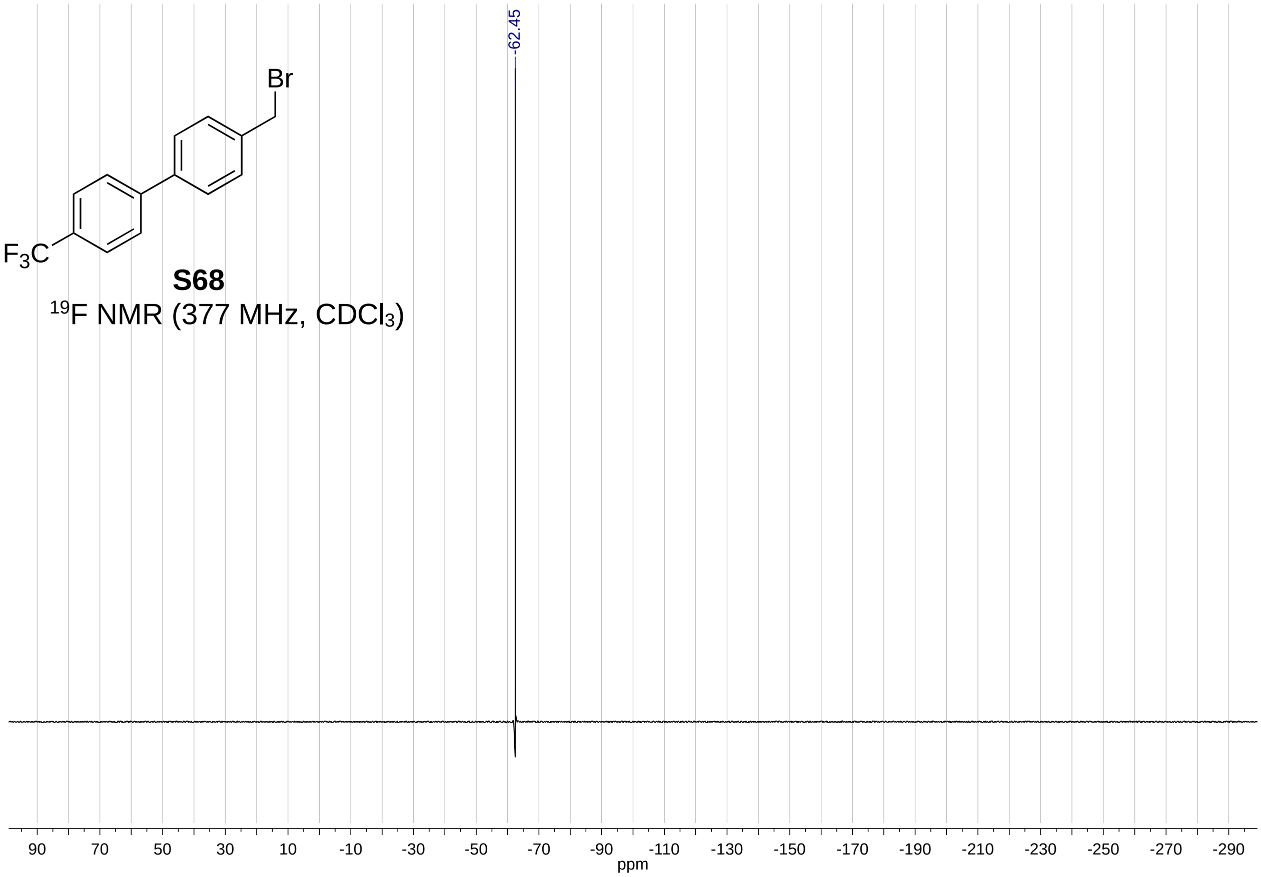

S68

^{19}F NMR (377 MHz, $CDCl_3$)

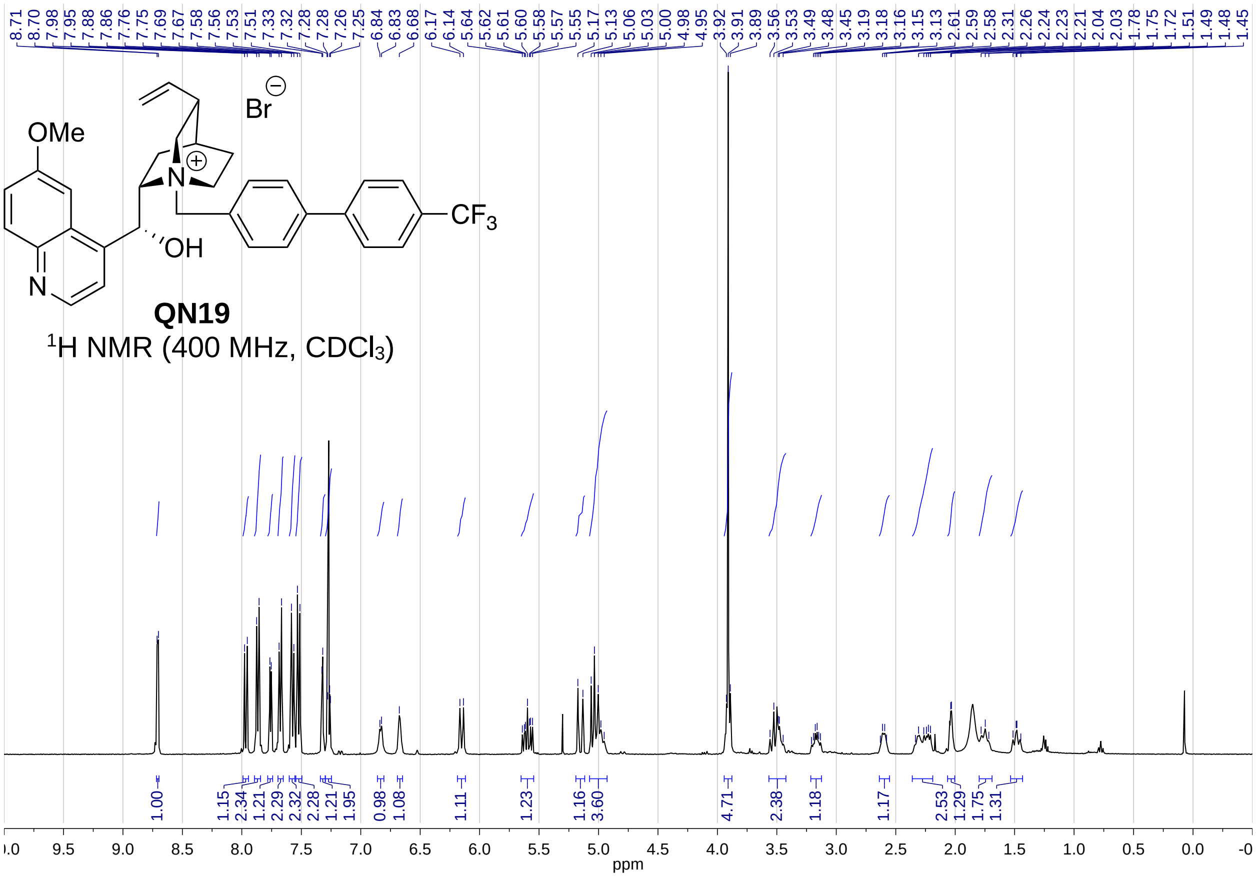
OMe
Br
N
OH
N
CF3
QN19
1H NMR (400 MHz, CDCl3)
ppm

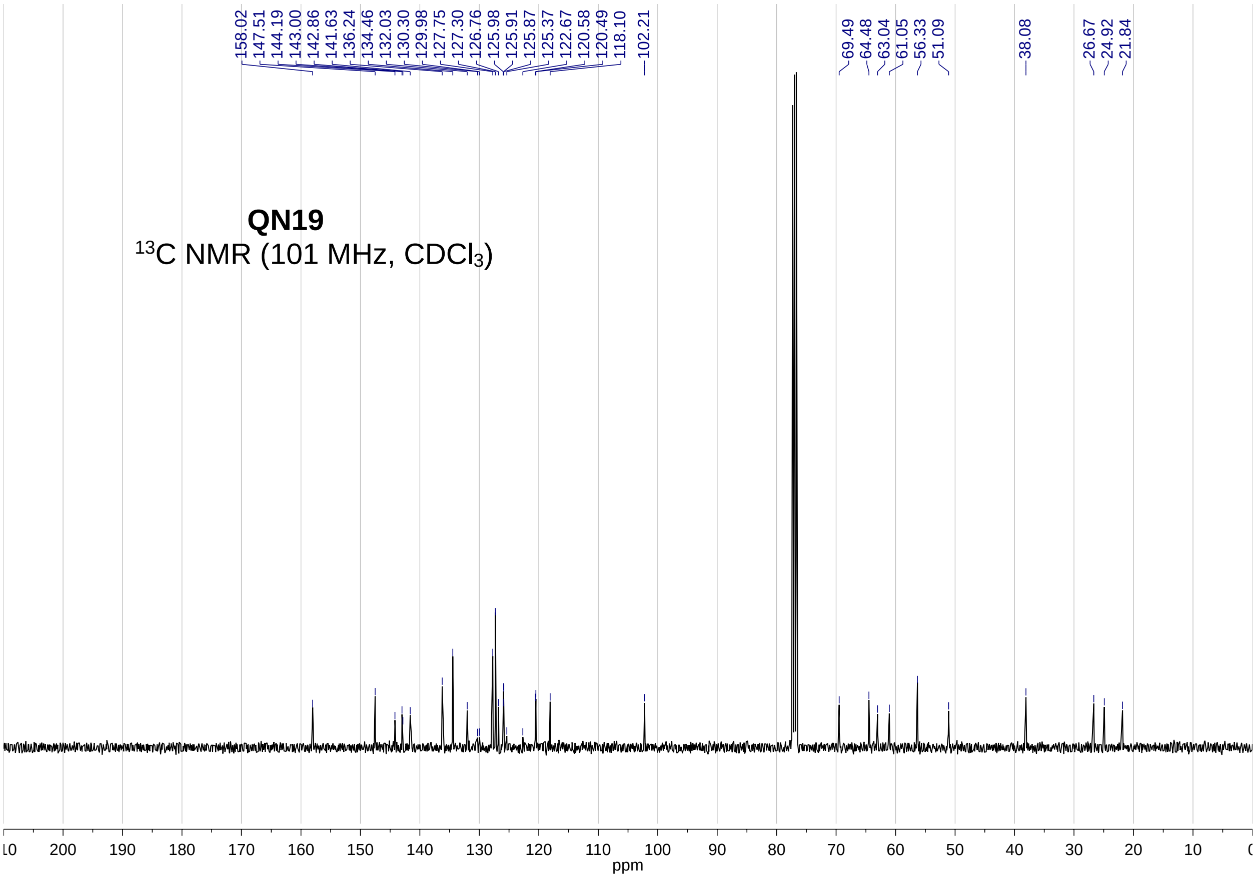
QN19
13C NMR (101 MHz, CDCl3)
ppm

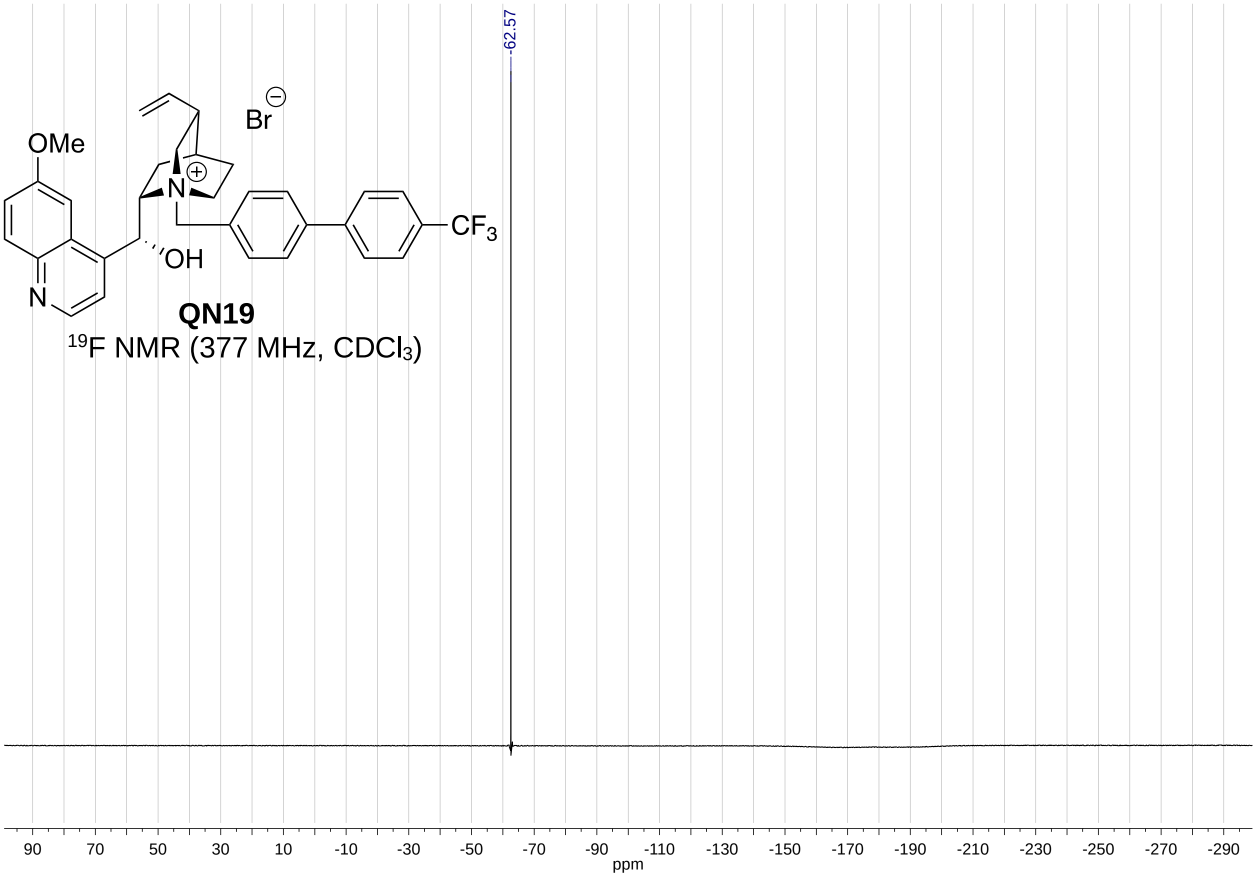

QN19

^{19}F NMR (377 MHz, $CDCl_3$)

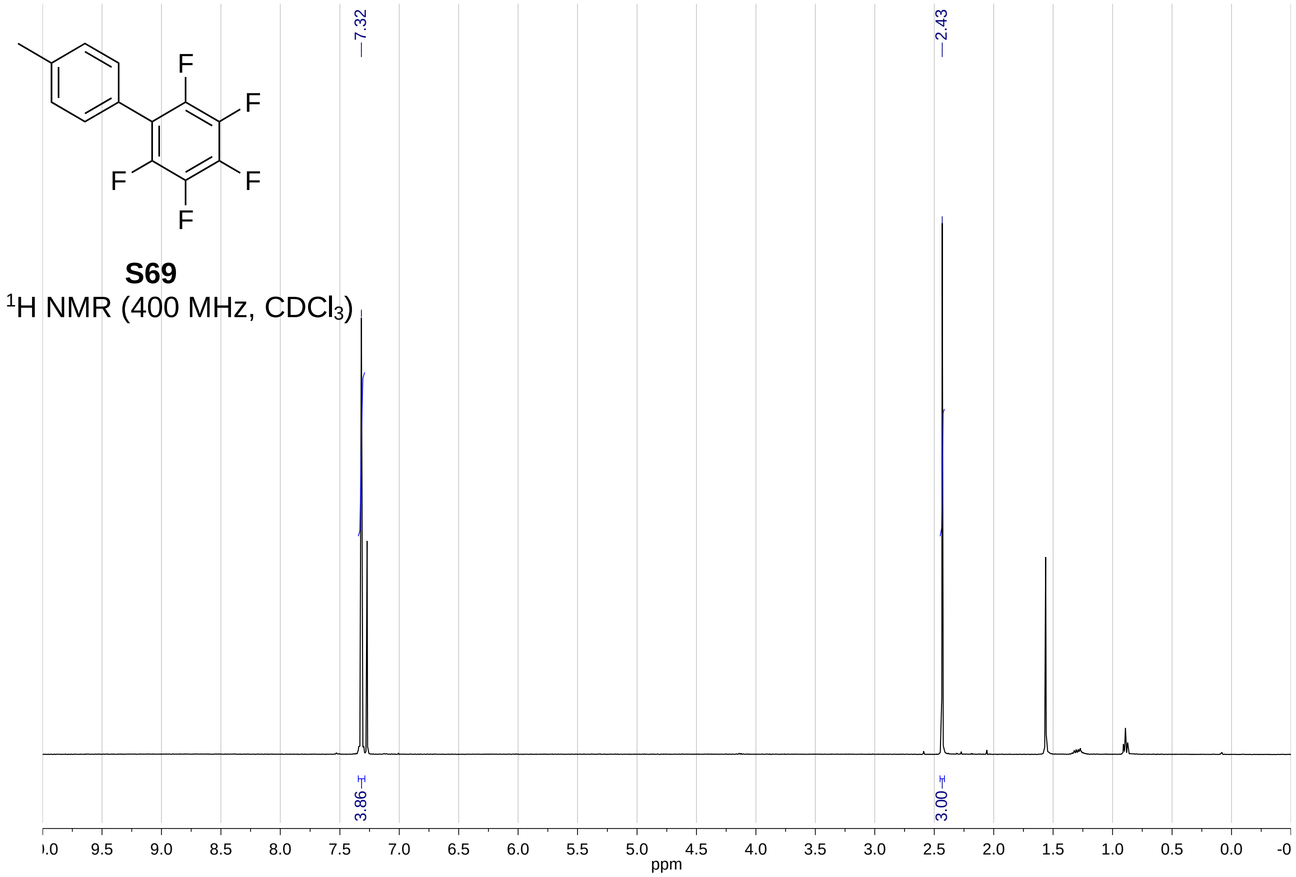

F
F
F
F
F
S69
^{1}H NMR (400 MHz, CDCl$_3$)
7.32
2.43
3.86
3.00
10.0 9.5 9.0 8.5 8.0 7.5 7.0 6.5 6.0 5.5 5.0 4.5 4.0 3.5 3.0 2.5 2.0 1.5 1.0 0.5 0.0 -0
ppm

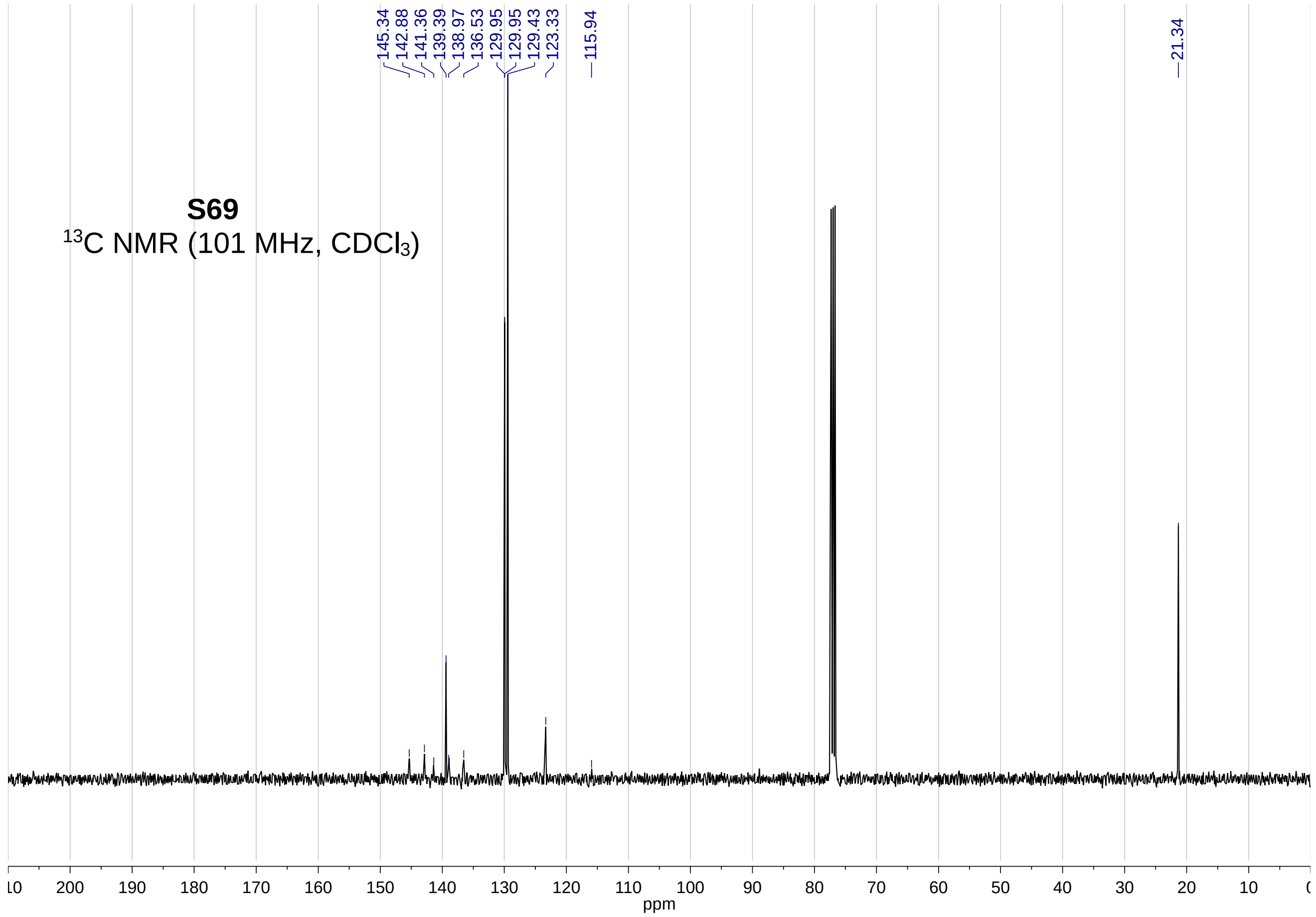

145.34
142.88
141.36
139.39
138.97
136.53
129.95
129.95
129.43
123.33
115.94
21.34
S69
^{13}C NMR (101 MHz, CDCl$_3$)
210 200 190 180 170 160 150 140 130 120 110 100 90 80 70 60 50 40 30 20 10 0
ppm

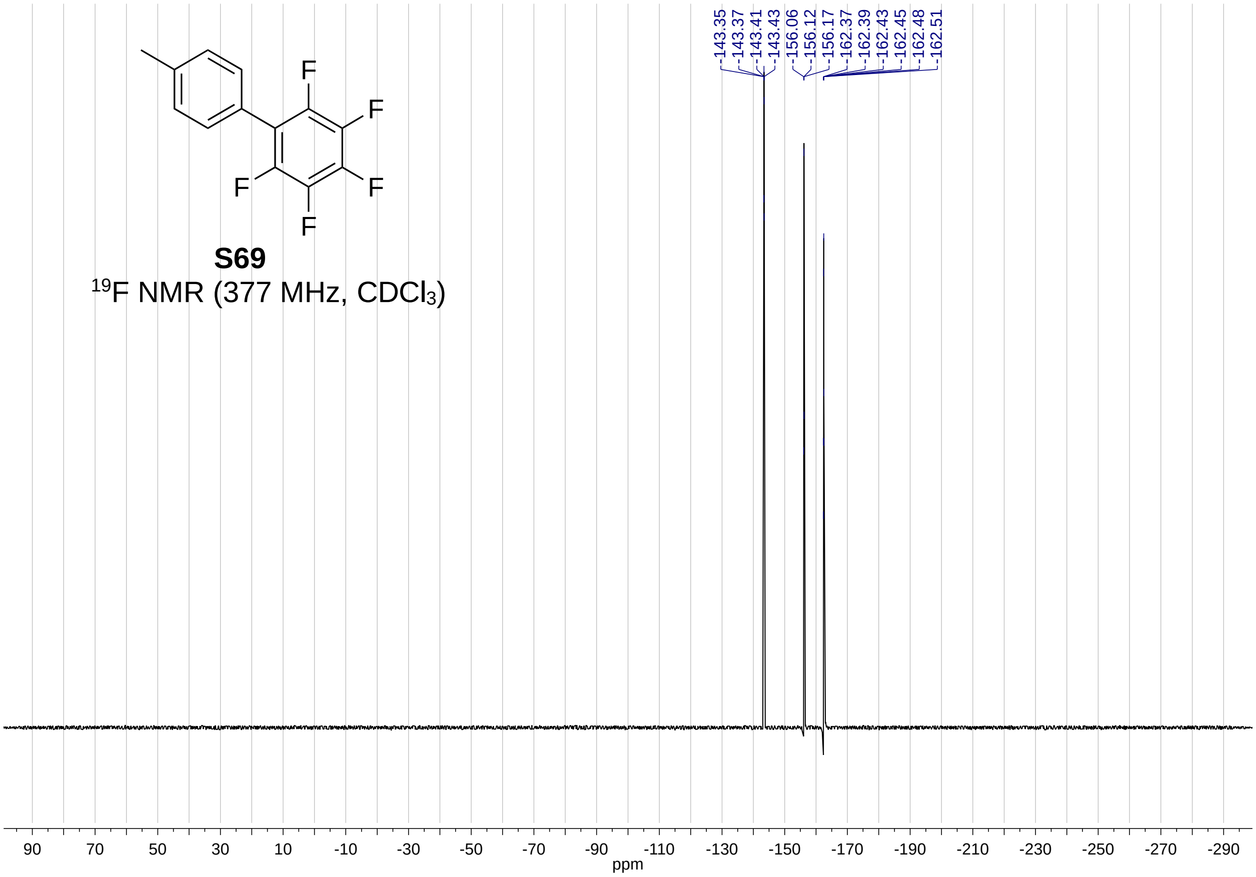

S69

^{19}F NMR (377 MHz, $CDCl_3$)

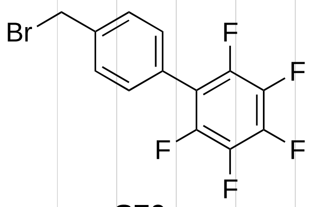

S70

^{1}H NMR (400 MHz, $CDCl_3$)

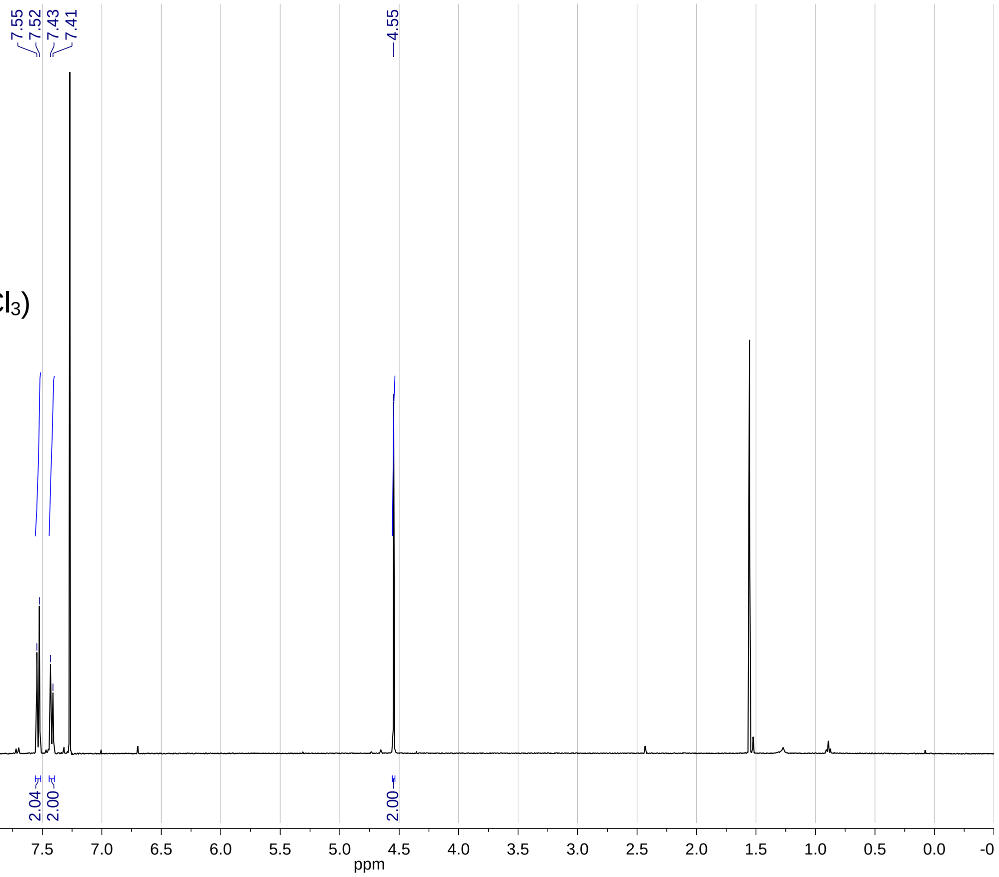

S70

^{13}C NMR (101 MHz, $CDCl_3$)

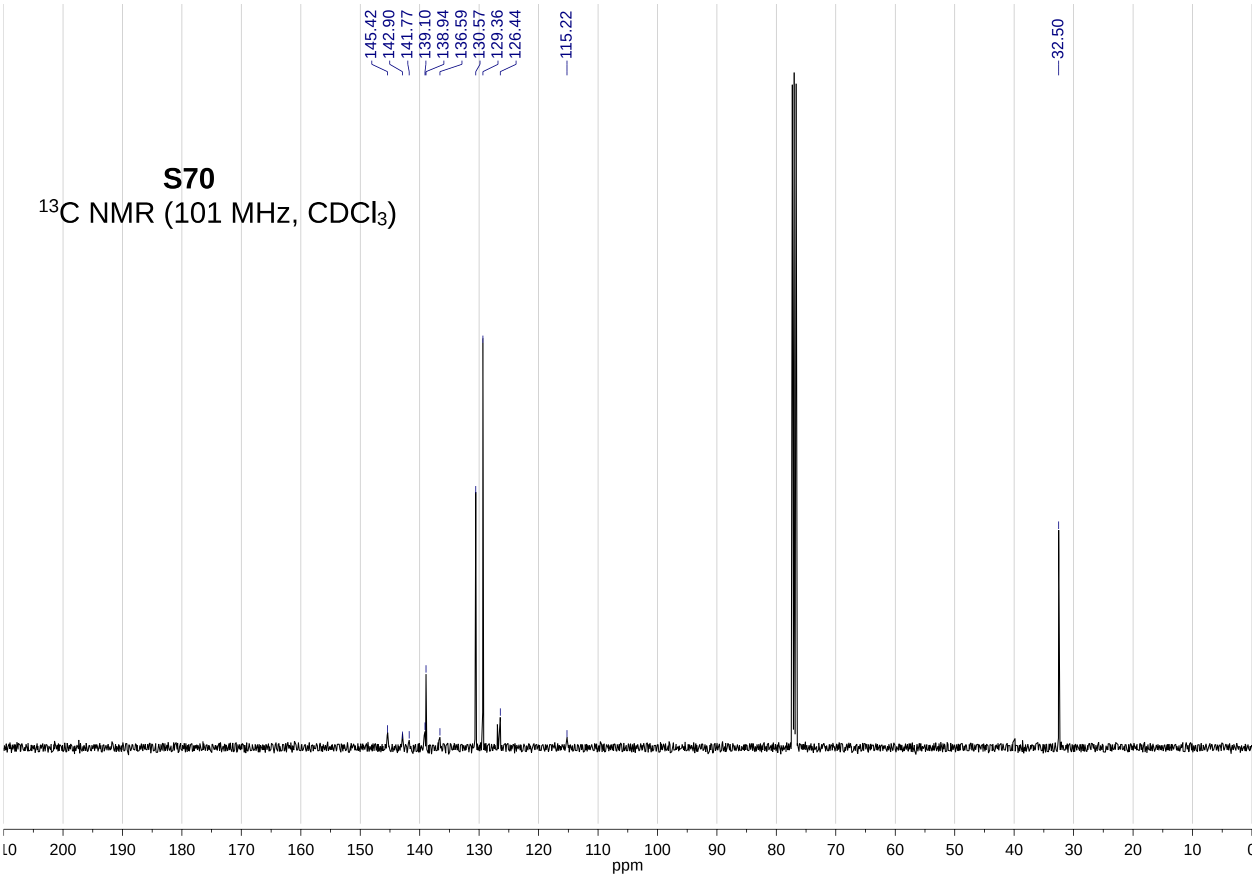

S70

^{19}F NMR (377 MHz, $CDCl_3$)

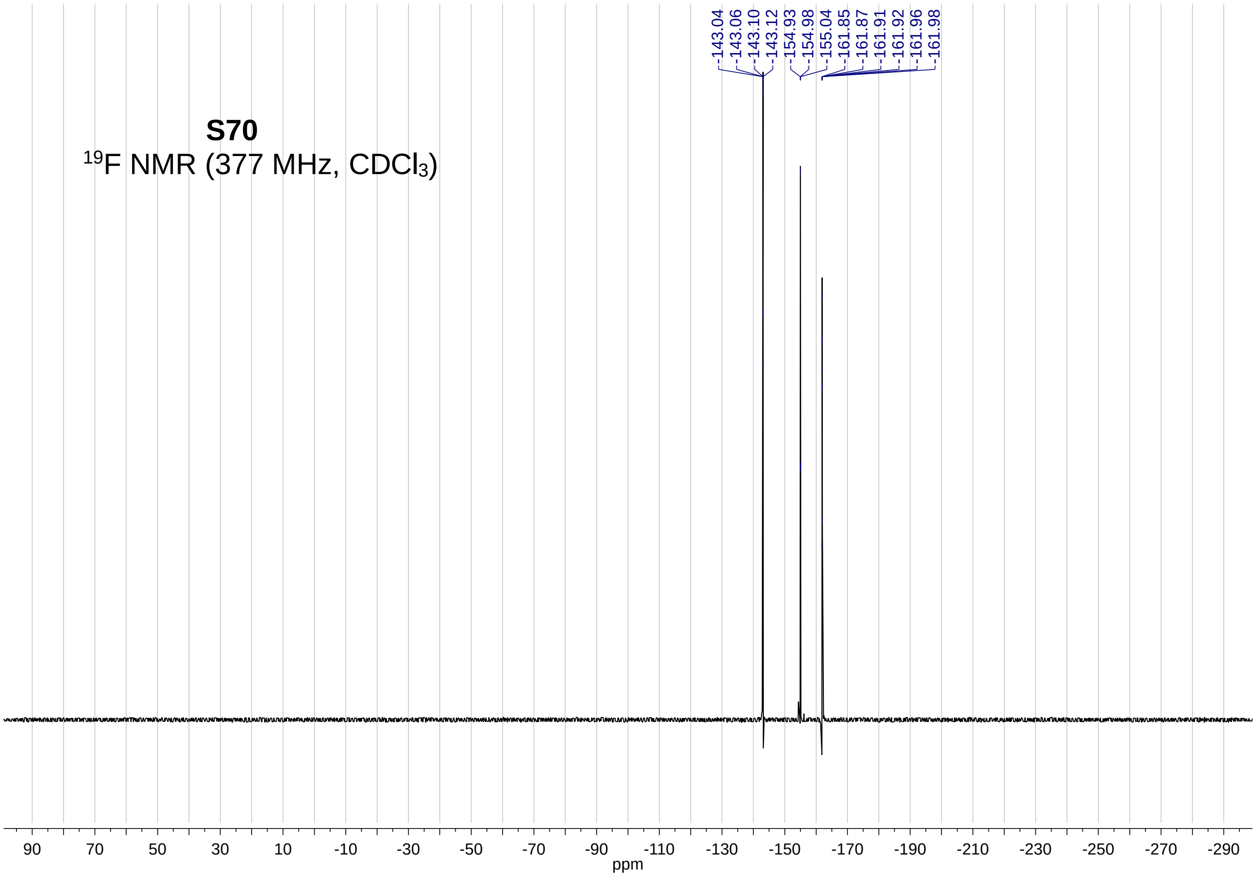

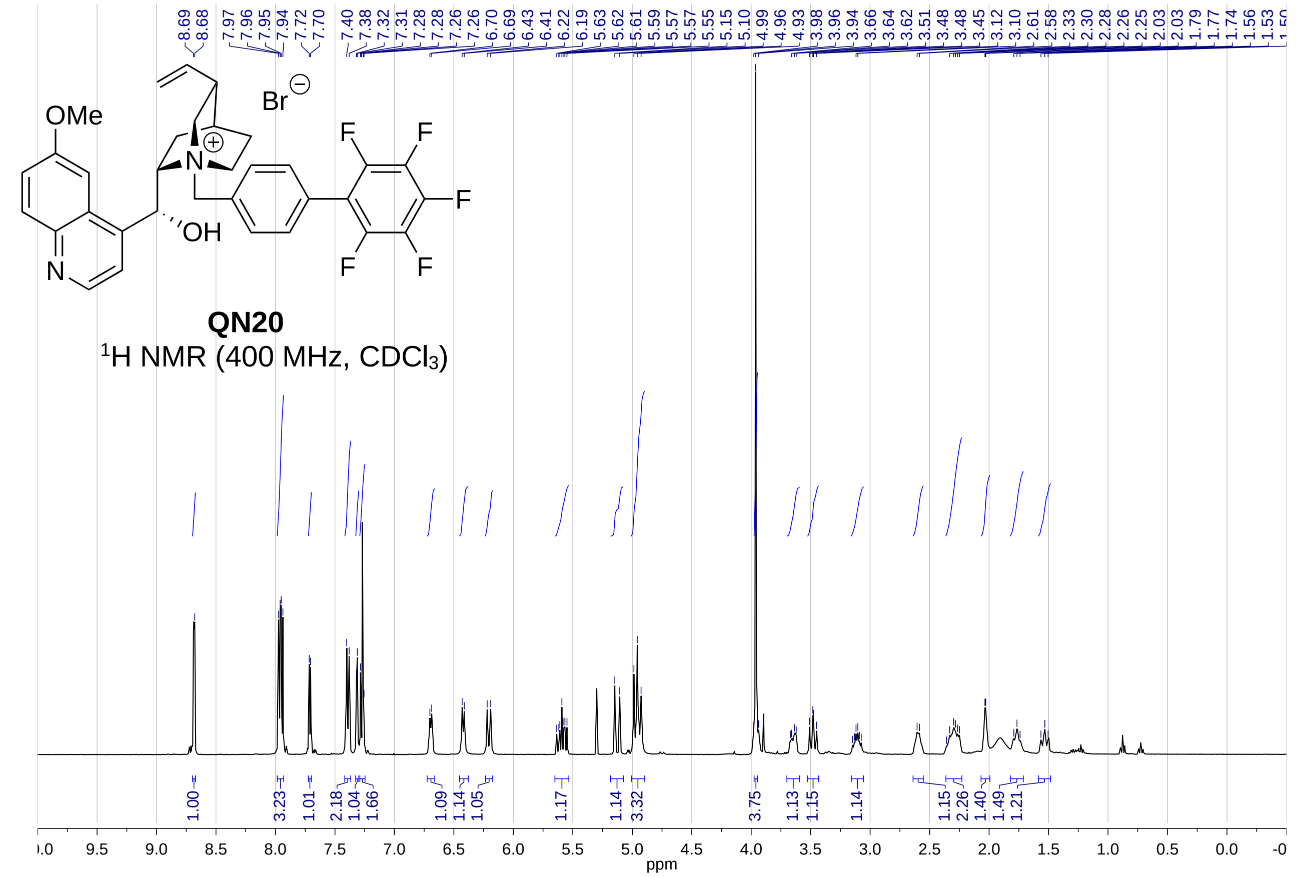
8.69
8.68
7.97
7.96
7.95
7.94
7.72
7.70
7.40
7.38
7.32
7.31
7.28
7.28
7.26
7.26
6.70
6.69
6.43
6.41
6.22
6.19
5.63
5.62
5.61
5.59
5.57
5.57
5.55
5.55
5.15
5.10
4.99
4.96
4.93
3.98
3.96
3.94
3.66
3.64
3.62
3.51
3.48
3.48
3.45
3.12
3.10
2.61
2.58
2.33
2.30
2.28
2.26
2.25
2.03
2.03
1.79
1.77
1.74
1.56
1.53
OMe
Br
N
OH
F
F
F
F
F
N
QN20
^{1}H NMR (400 MHz, $CDCl_3$)
1.00
3.23
1.01
2.18
1.04
1.66
1.09
1.14
1.05
1.17
1.14
3.32
3.75
1.13
1.15
1.14
1.15
2.26
1.40
1.49
1.21
9.5
9.0
8.5
8.0
7.5
7.0
6.5
6.0
5.5
5.0
4.5
4.0
3.5
3.0
2.5
2.0
1.5
1.0
0.5
0.0
ppm

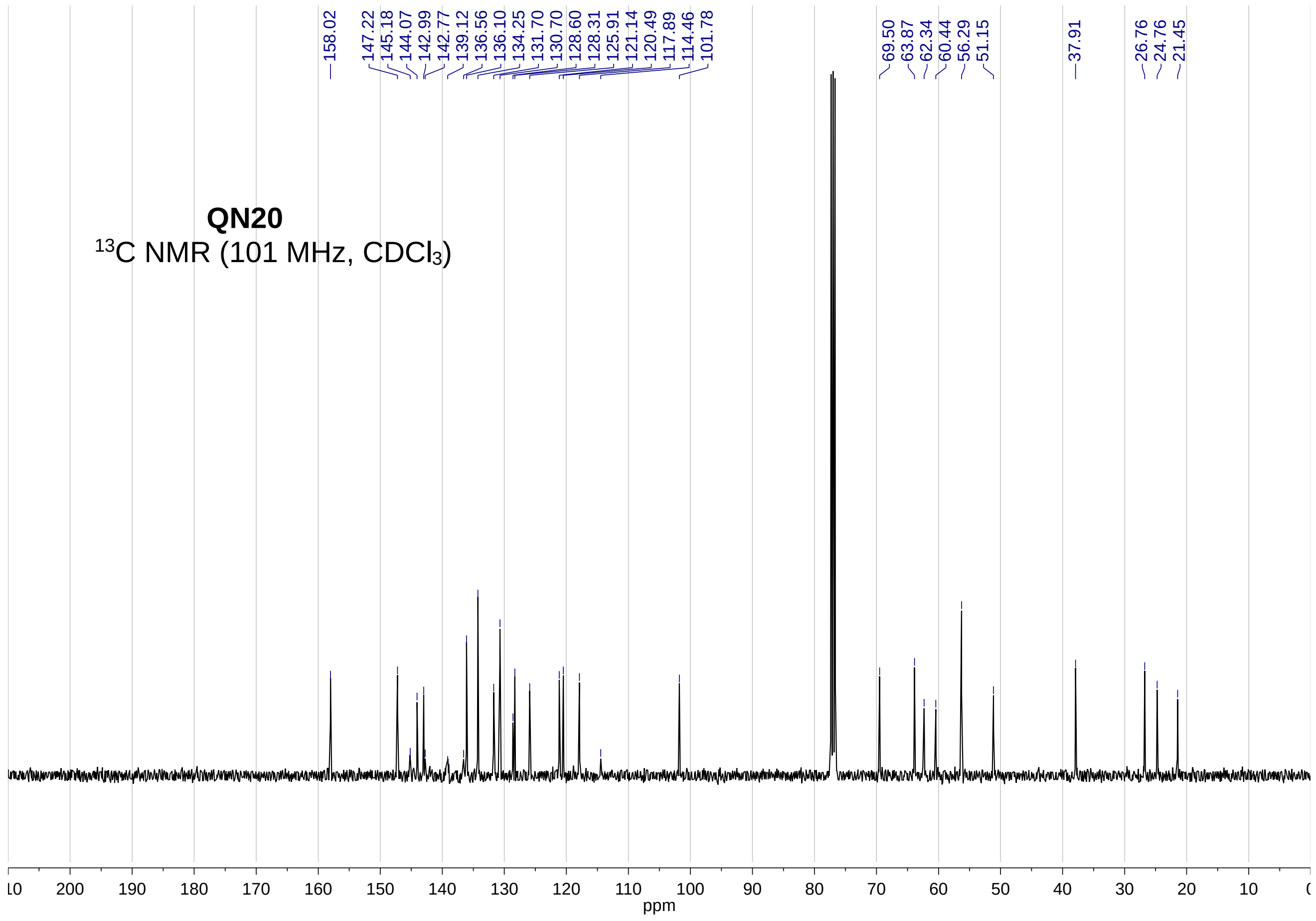
158.02
147.22
145.18
144.07
142.99
142.77
139.12
136.56
136.10
134.25
131.70
130.70
128.60
128.31
125.91
121.14
120.49
117.89
114.46
101.78
69.50
63.87
62.34
60.44
56.29
51.15
37.91
26.76
24.76
21.45
QN20
^{13}C NMR (101 MHz, $CDCl_3$)
200
190
180
170
160
150
140
130
120
110
100
90
80
70
60
50
40
30
20
10
ppm

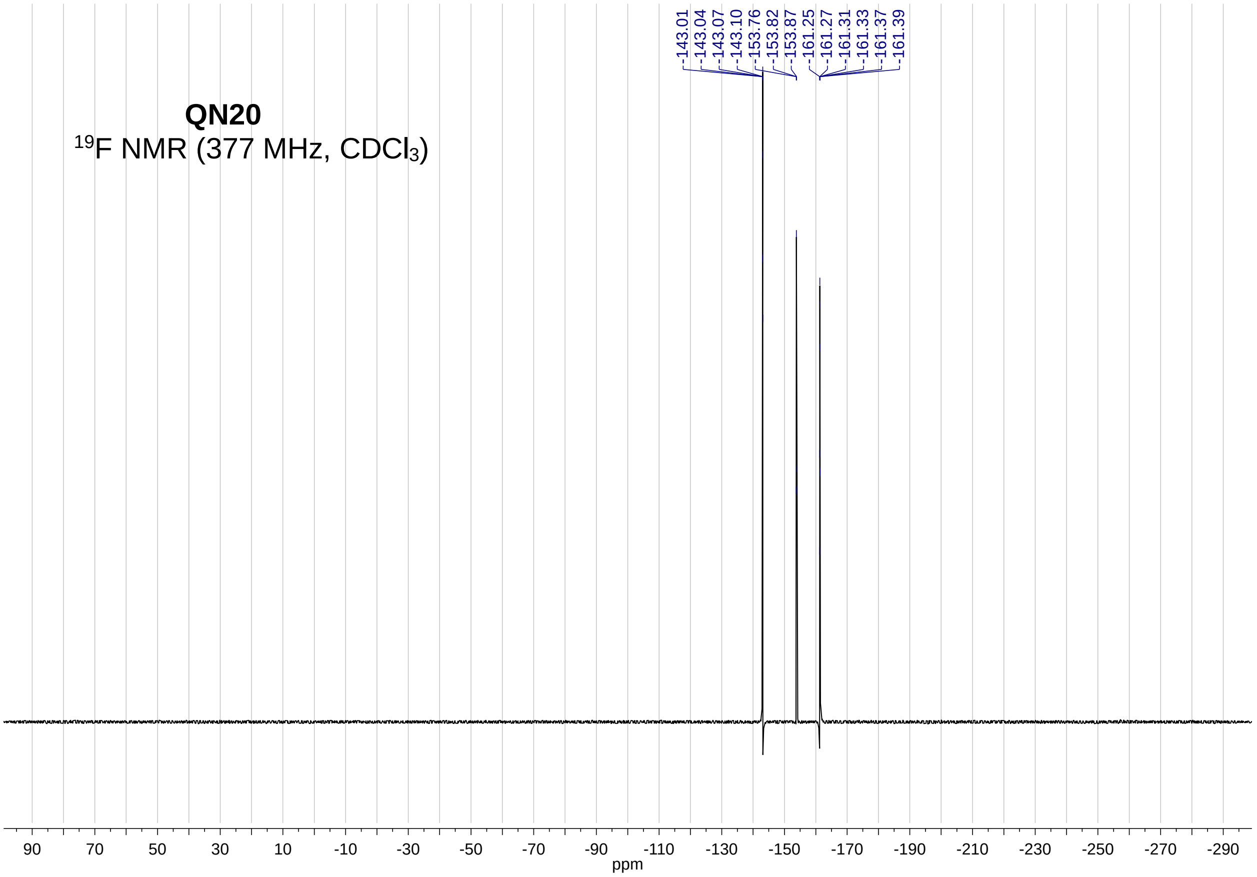
-143.01
-143.04
-143.07
-143.10
-153.76
-153.82
-153.87
-161.25
-161.27
-161.31
-161.33
-161.37
-161.39
QN20
19F NMR (377 MHz, CDCl3)
90 70 50 30 10 -10 -30 -50 -70 -90 -110 -130 -150 -170 -190 -210 -230 -250 -270 -290
ppm

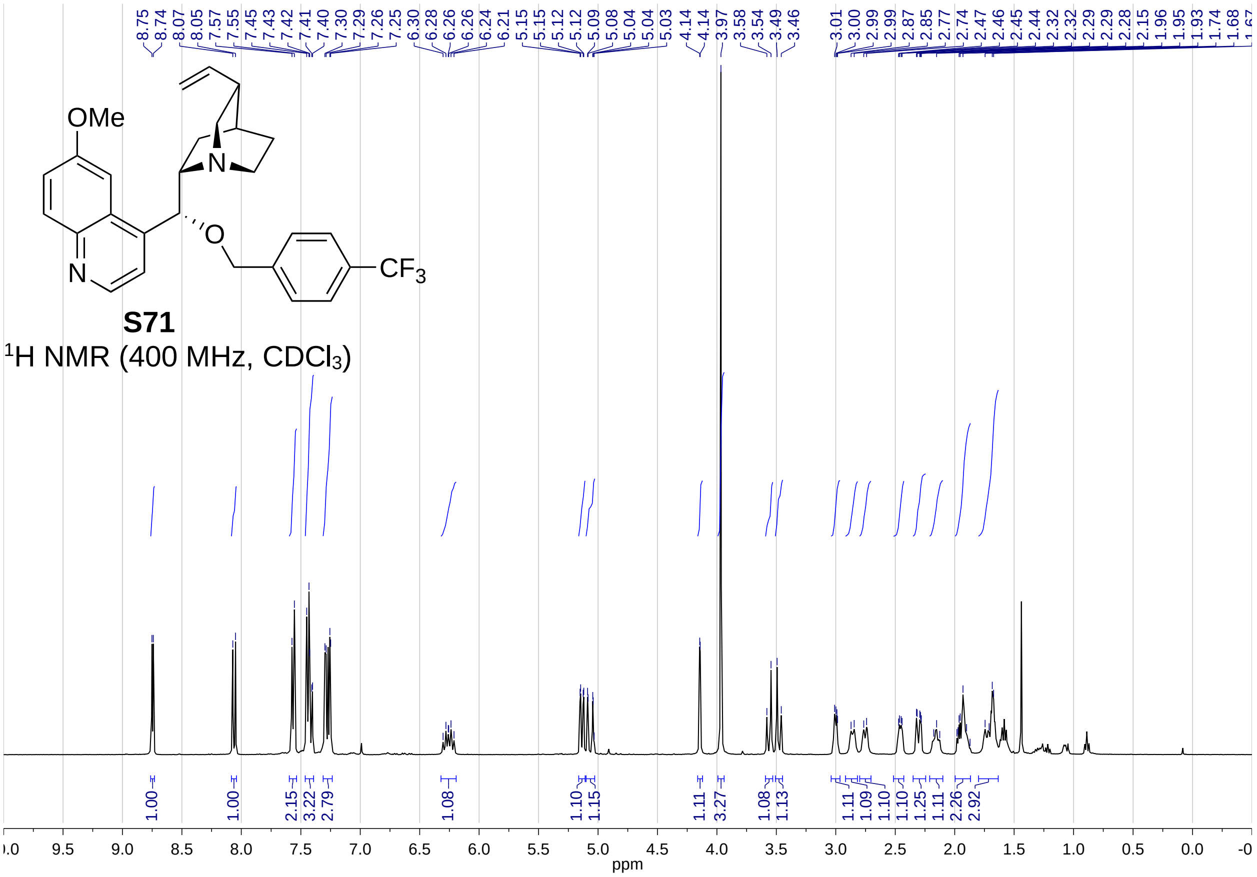
OMe
N
O
CF3
N
S71
1H NMR (400 MHz, CDCl3)
ppm

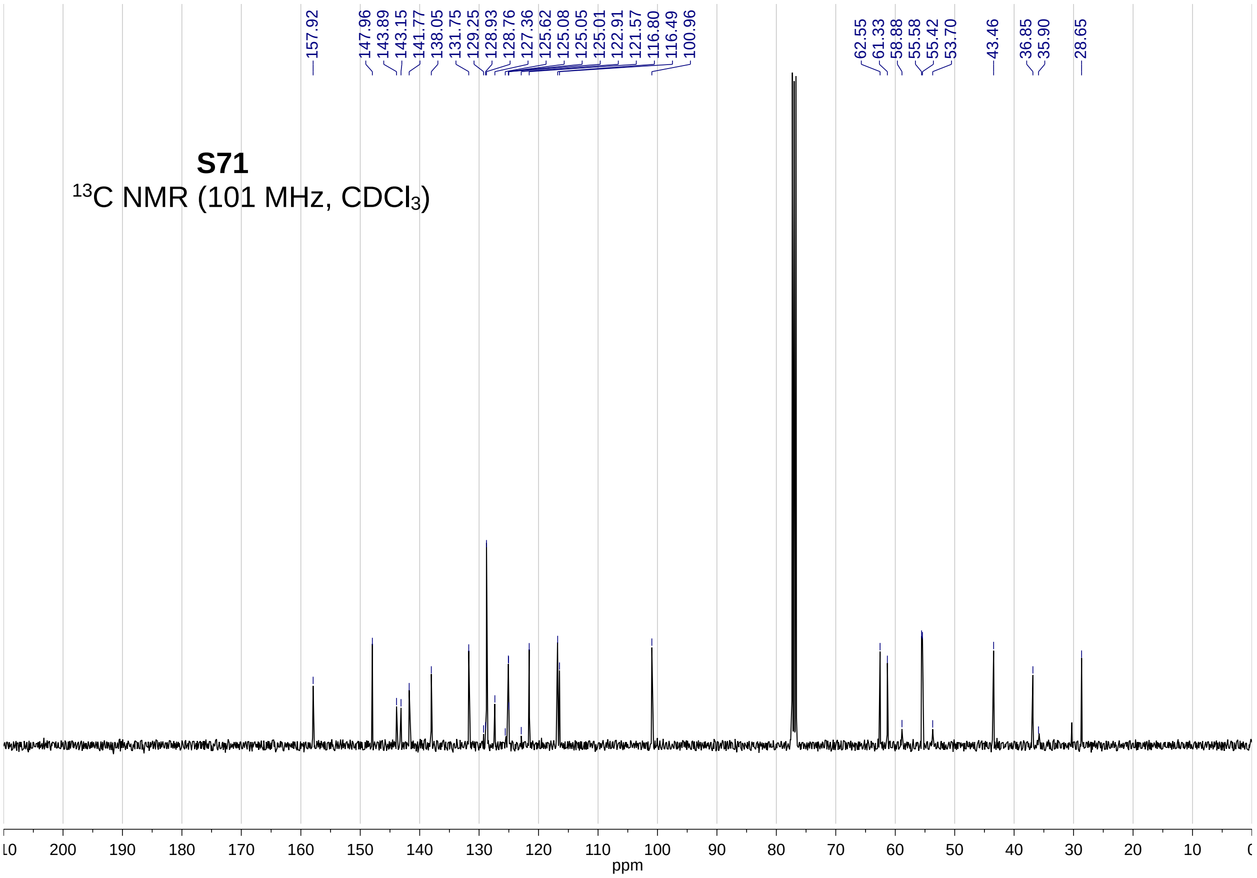
S71
13C NMR (101 MHz, CDCl3)
ppm

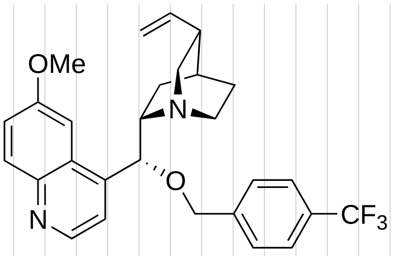

S71

^{19}F NMR (377 MHz, $CDCl_3$)

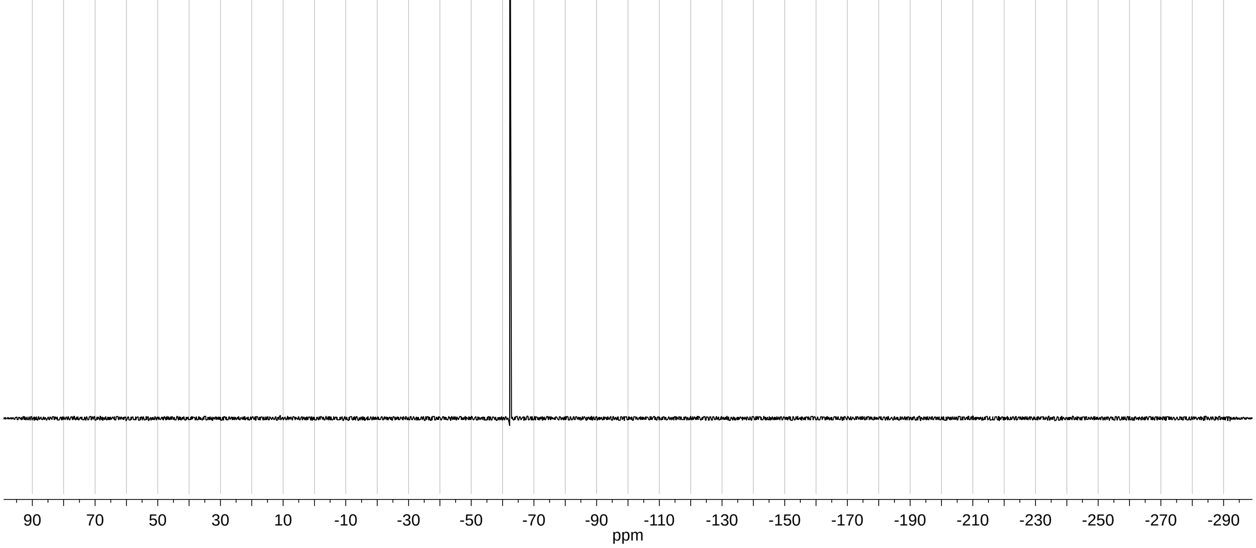

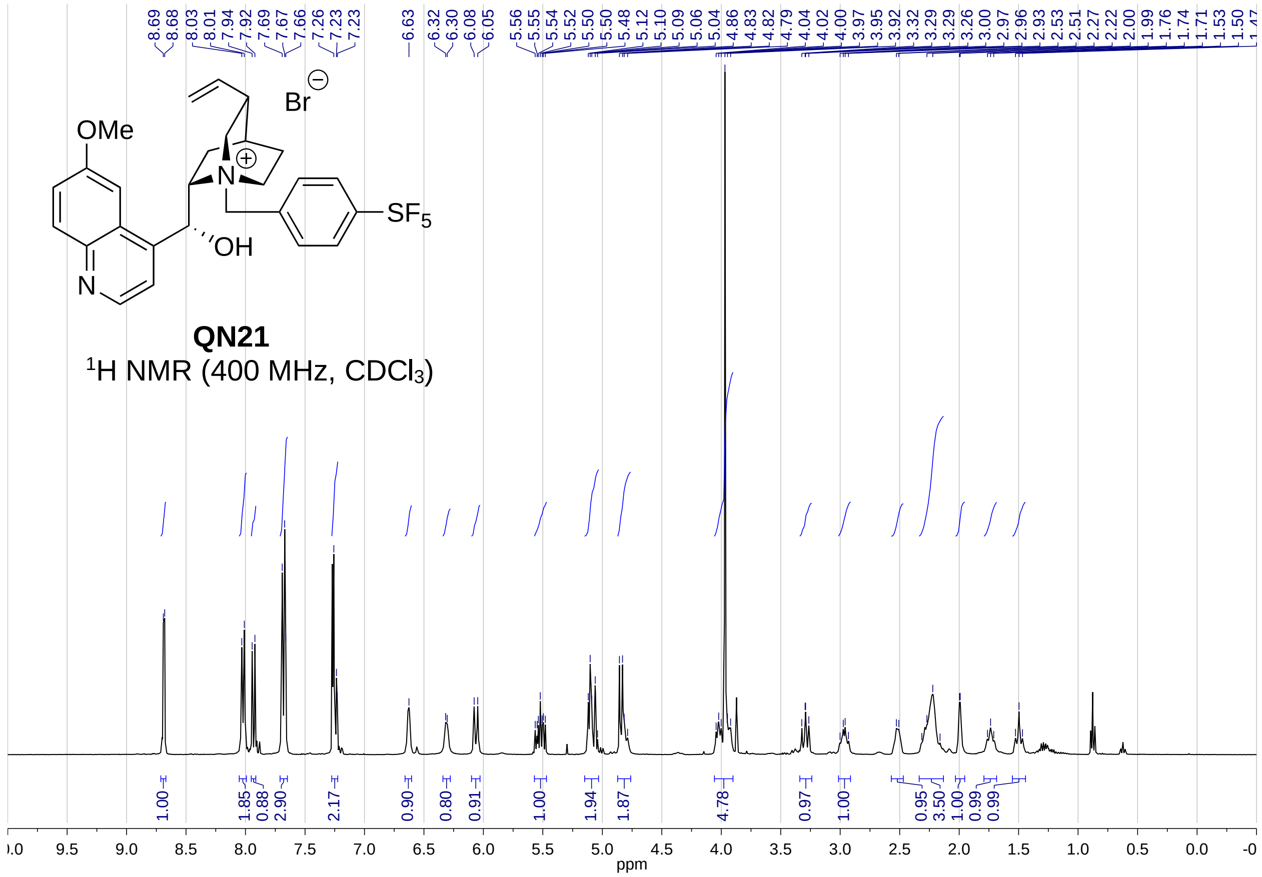

QN21

^{1}H NMR (400 MHz, $CDCl_3$)

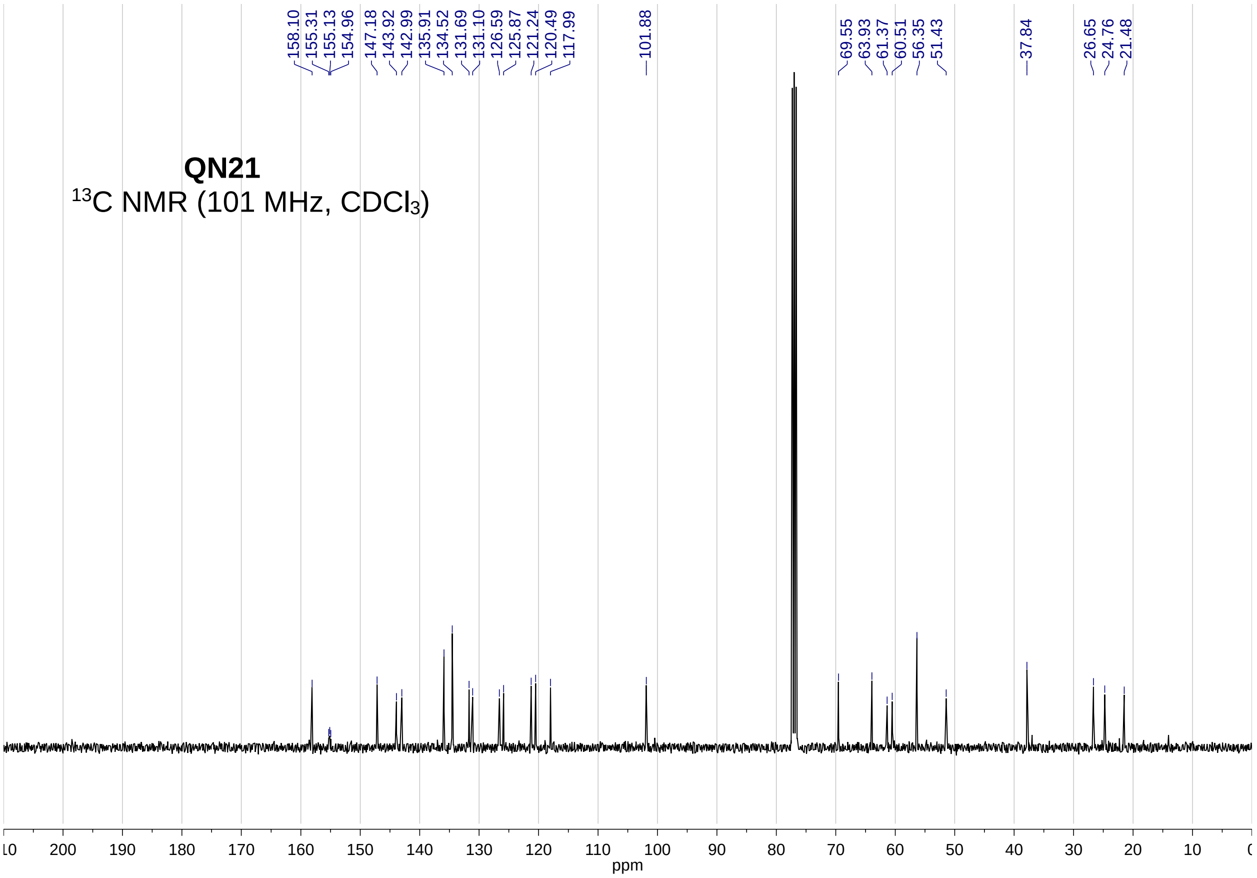

QN21

^{13}C NMR (101 MHz, $CDCl_3$)

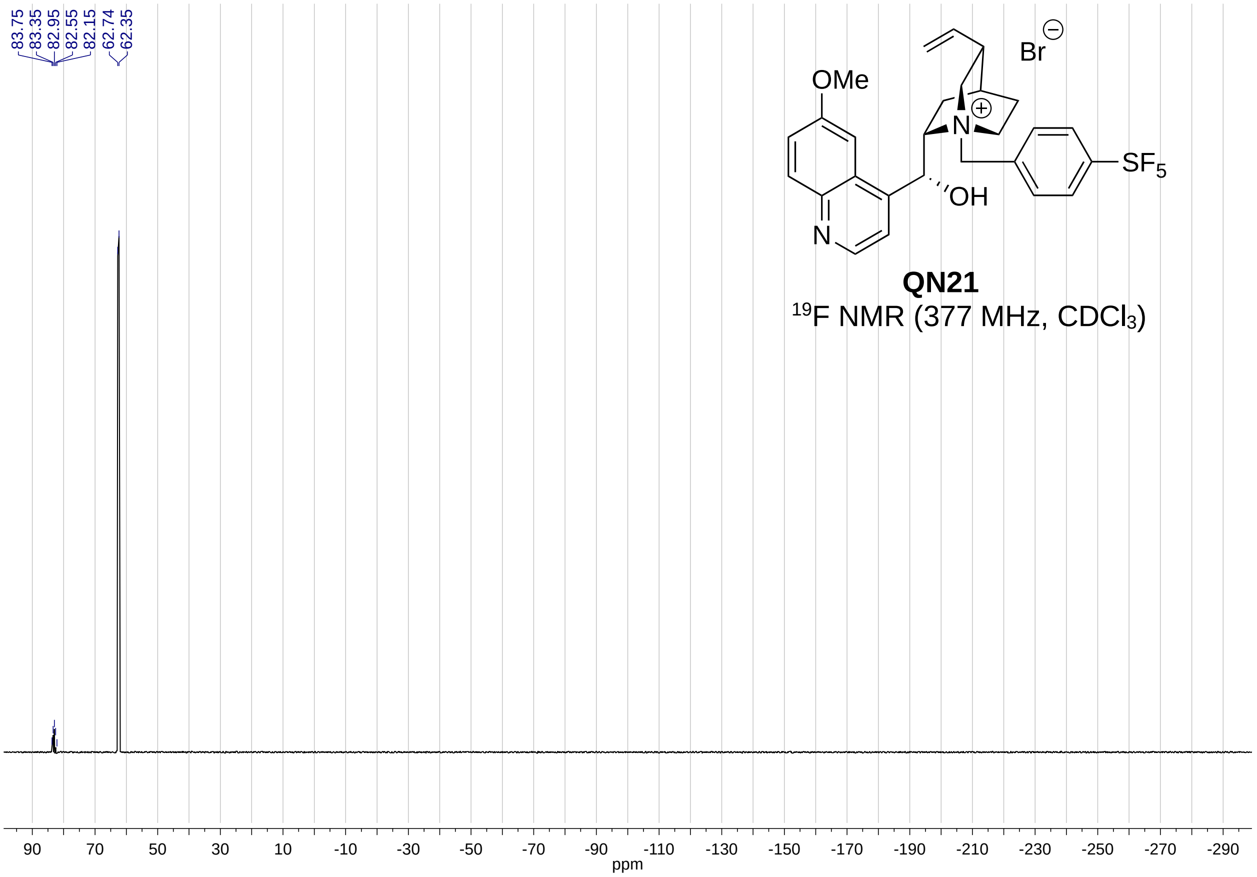

QN21

^{19}F NMR (377 MHz, $CDCl_3$)

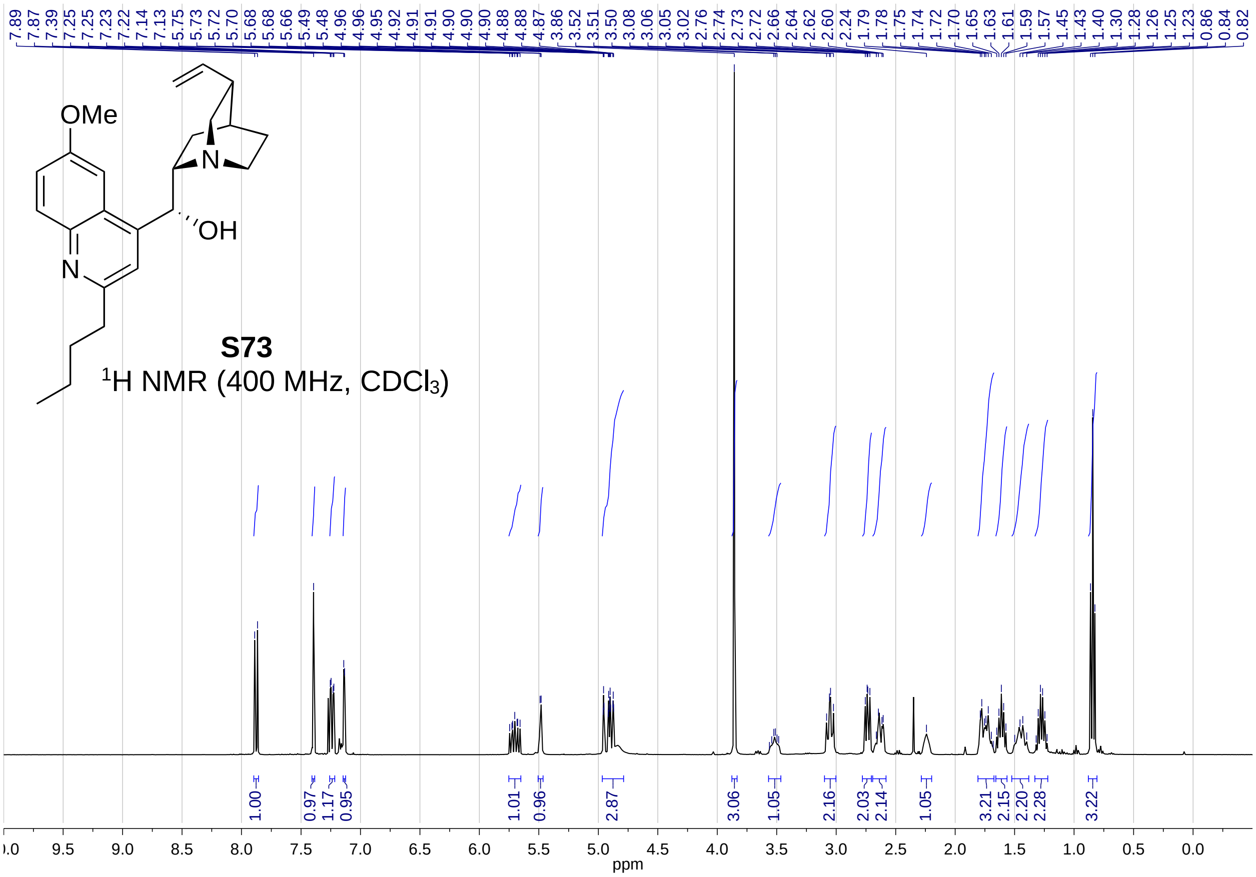
7.89 7.87 7.39 7.25 7.25 7.23 7.22 7.14 7.13 5.75 5.73 5.72 5.70 5.68 5.68 5.66 5.49 5.48 4.96 4.96 4.95 4.92 4.91 4.91 4.90 4.90 4.88 4.88 4.87 3.86 3.52 3.51 3.50 3.08 3.06 3.05 3.02 2.76 2.74 2.73 2.72 2.66 2.64 2.62 2.60 2.24 1.79 1.78 1.75 1.74 1.72 1.70 1.65 1.63 1.61 1.59 1.57 1.45 1.43 1.40 1.30 1.28 1.26 1.25 1.23 0.86 0.84 0.82
OMe
N
N
OH
S73
1H NMR (400 MHz, CDCl3)
1.00 0.97 1.17 0.95 1.01 0.96 2.87 3.06 1.05 2.16 2.03 2.14 1.05 3.21 2.15 2.20 2.28 3.22
ppm

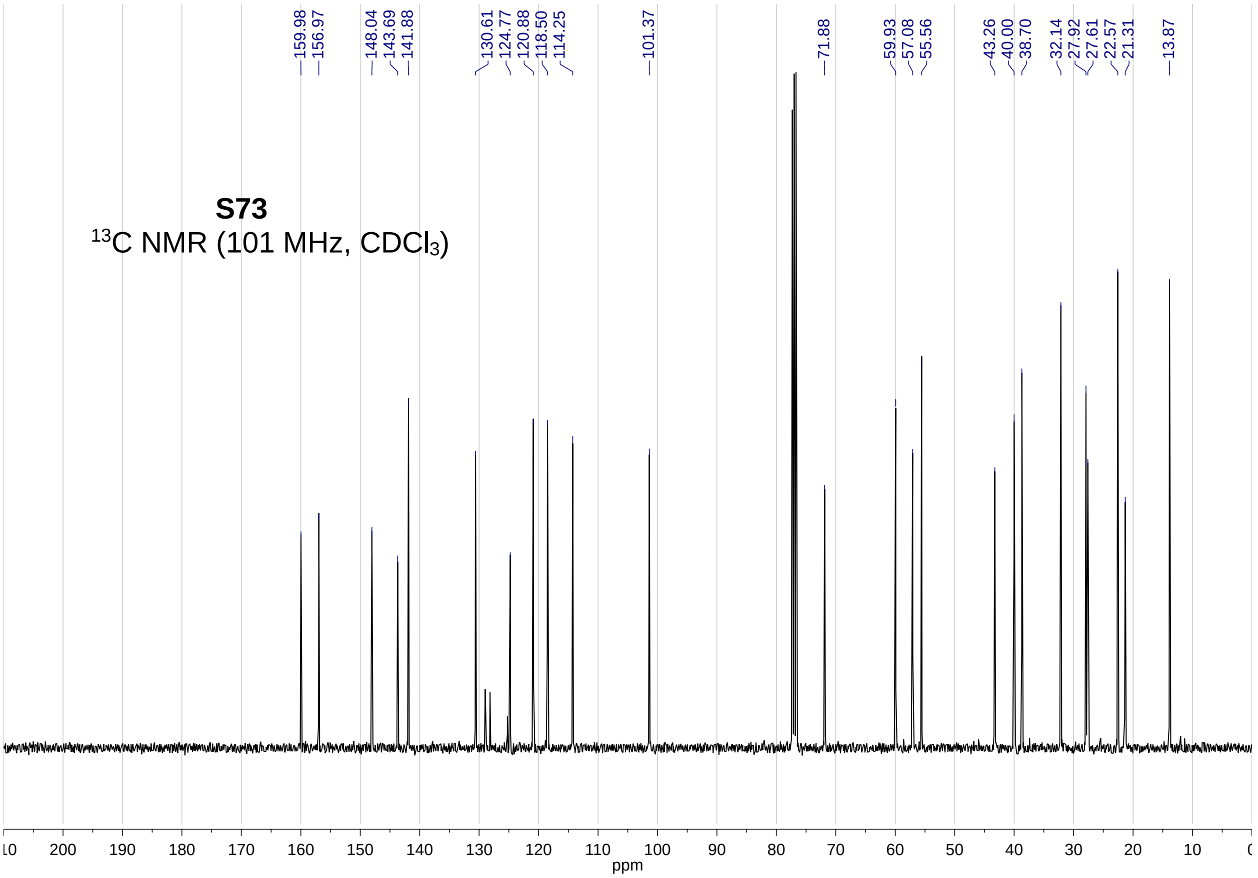
159.98 156.97 148.04 143.69 141.88 130.61 124.77 120.88 118.50 114.25 101.37 71.88 59.93 57.08 55.56 43.26 40.00 38.70 32.14 27.92 27.61 22.57 21.31 13.87
S73
13C NMR (101 MHz, CDCl3)
ppm

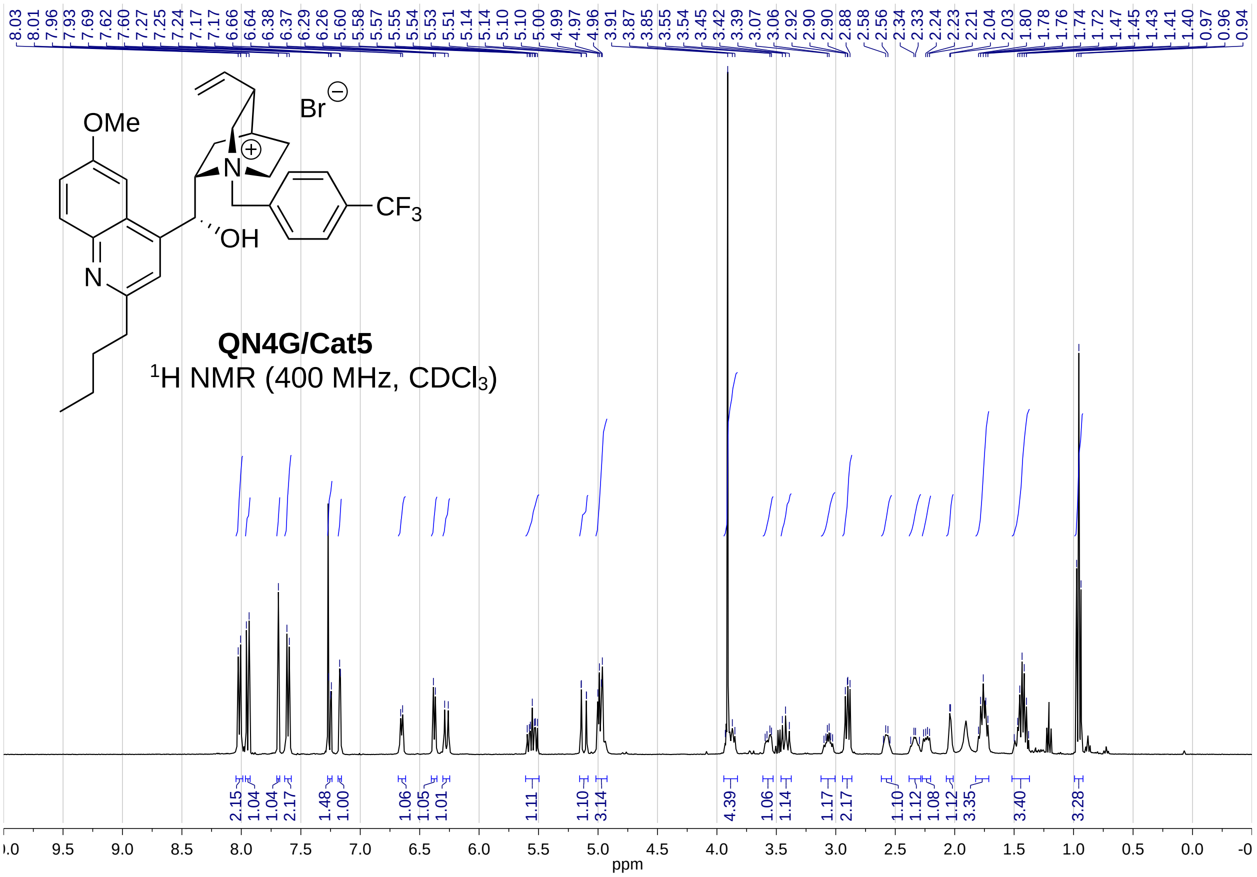
QN4G/Cat5
^{1}H NMR (400 MHz, CDCl$_3$)
ppm

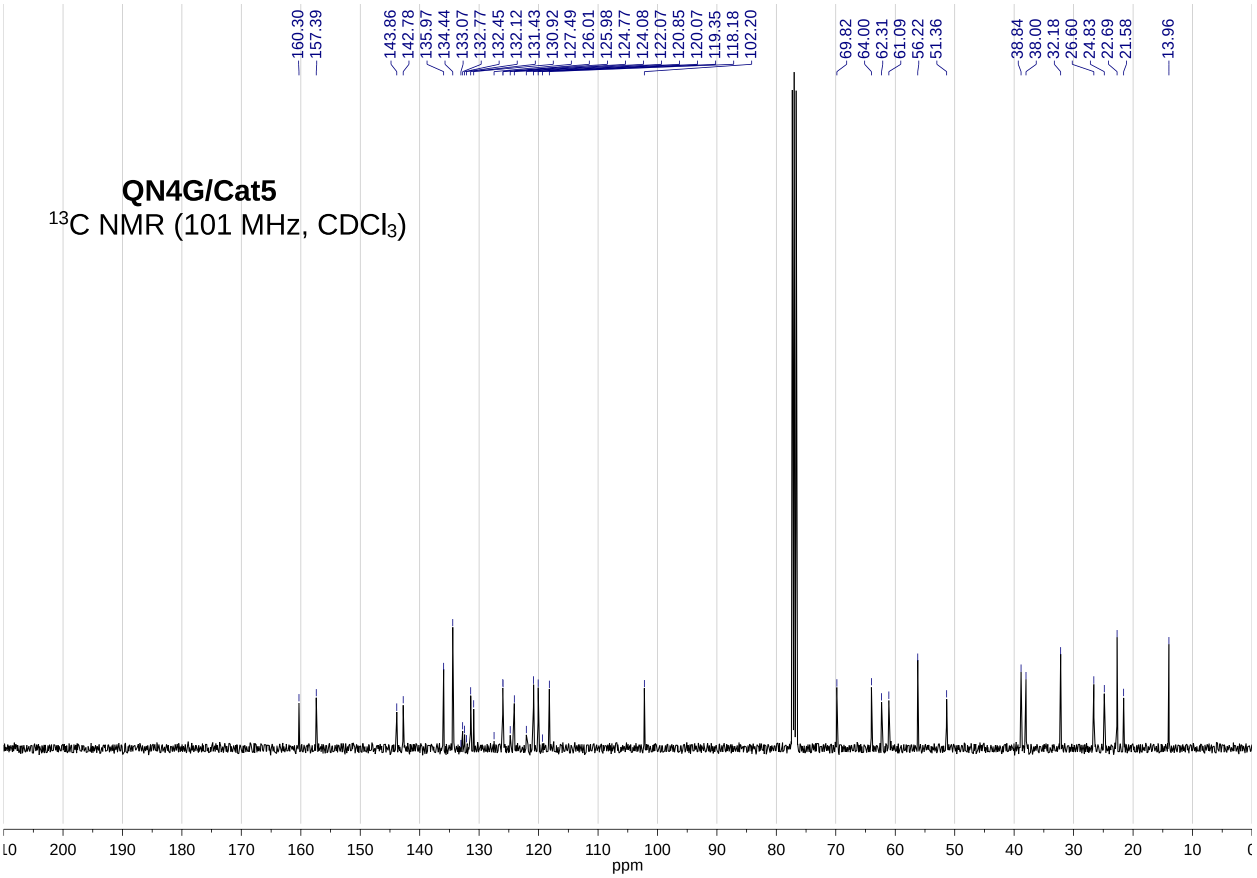
QN4G/Cat5
^{13}C NMR (101 MHz, CDCl$_3$)
ppm

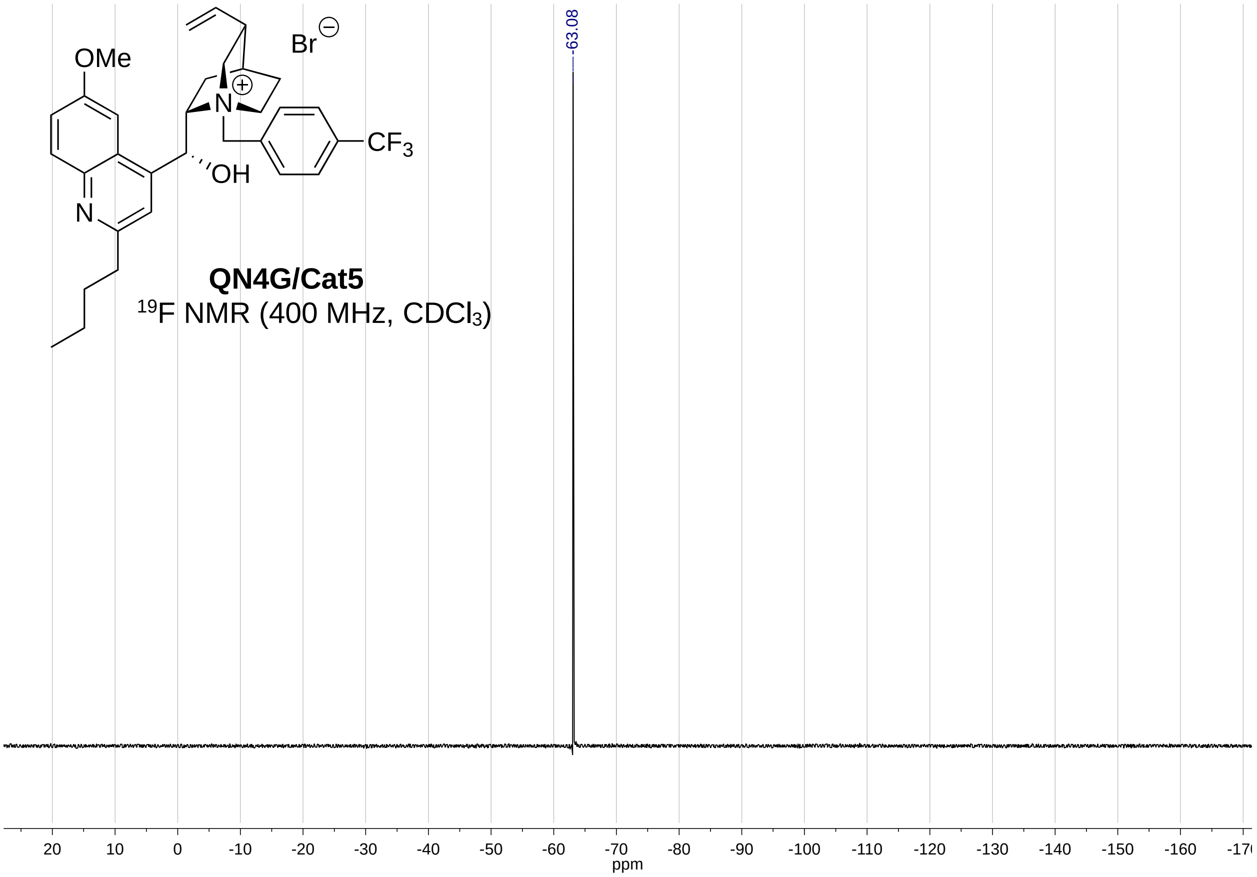
OMe
Br
N
OH
CF3
N
QN4G/Cat5
19F NMR (400 MHz, CDCl3)
-63.08
20
10
0
-10
-20
-30
-40
-50
-60
-70
-80
-90
-100
-110
-120
-130
-140
-150
-160
ppm

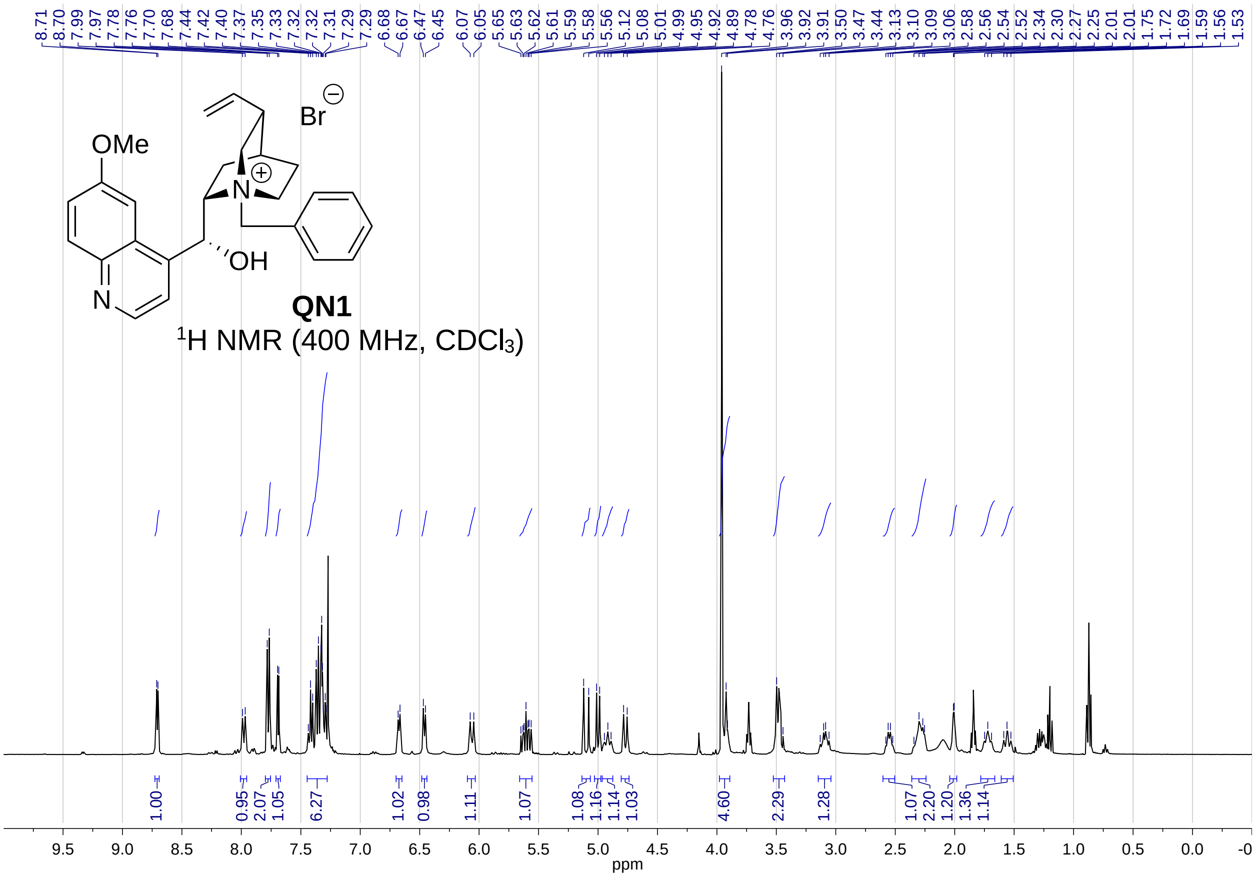
OMe
Br
N
N
OH
QN1
1H NMR (400 MHz, CDCl3)
ppm

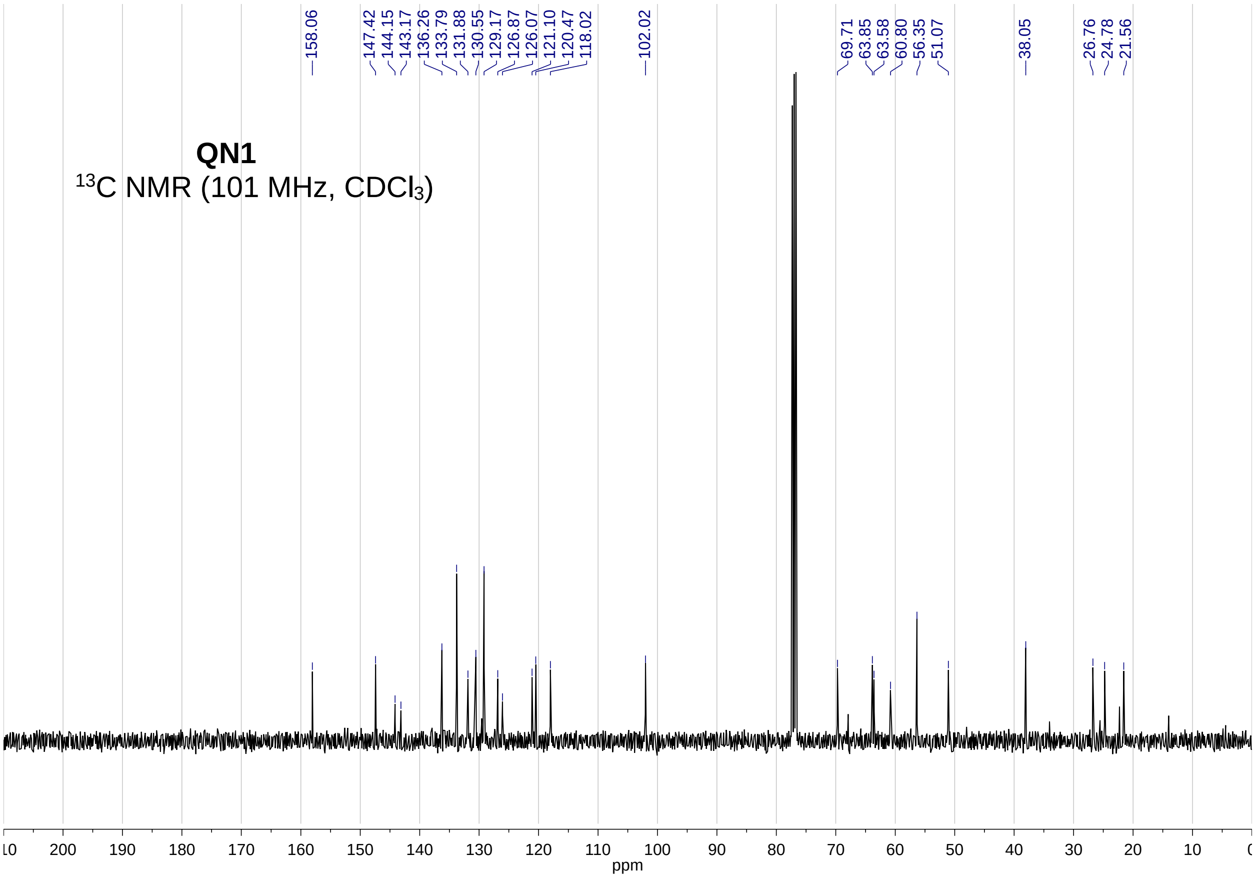
QN1
13C NMR (101 MHz, CDCl3)
ppm

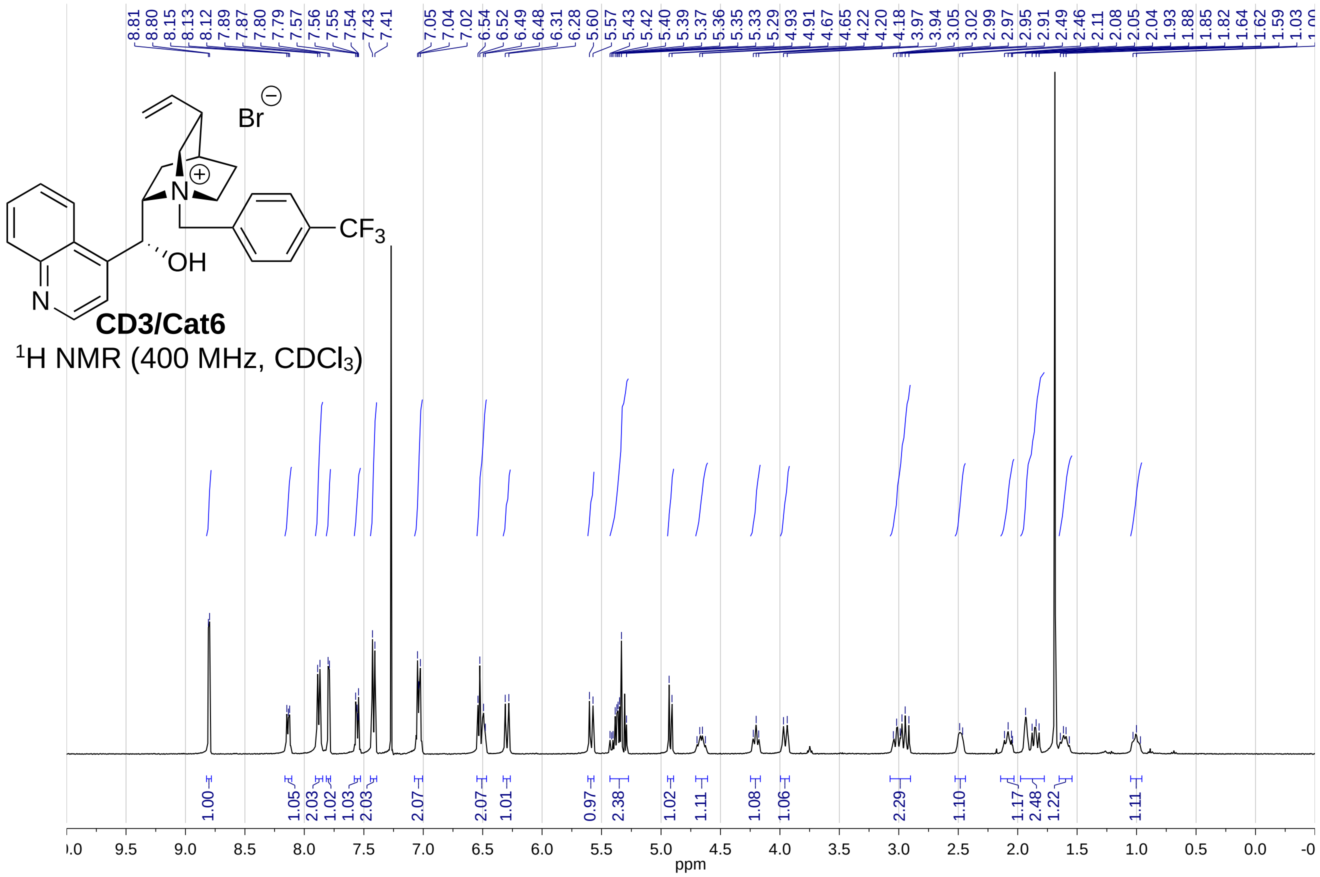

CD3/Cat6

^{1}H NMR (400 MHz, $CDCl_3$)

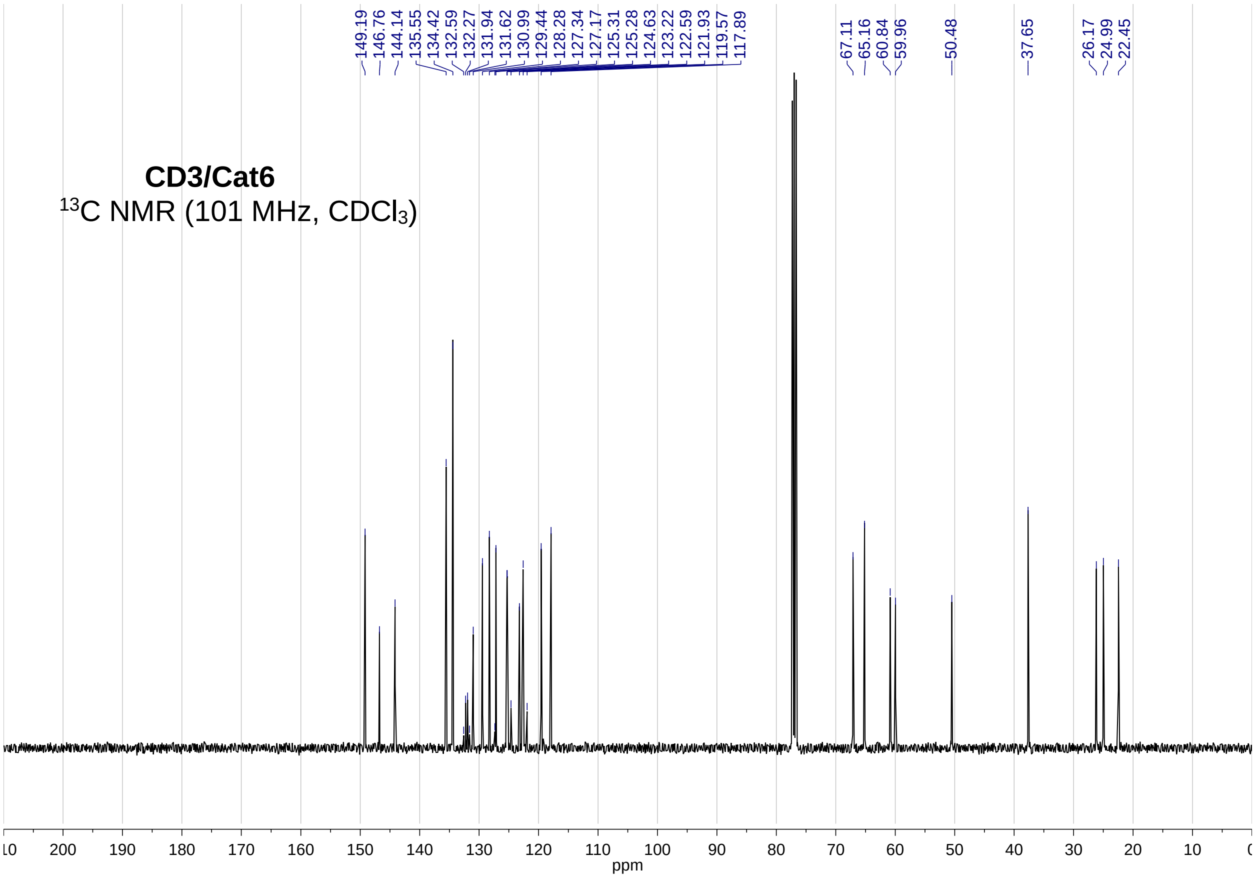

CD3/Cat6

^{13}C NMR (101 MHz, $CDCl_3$)

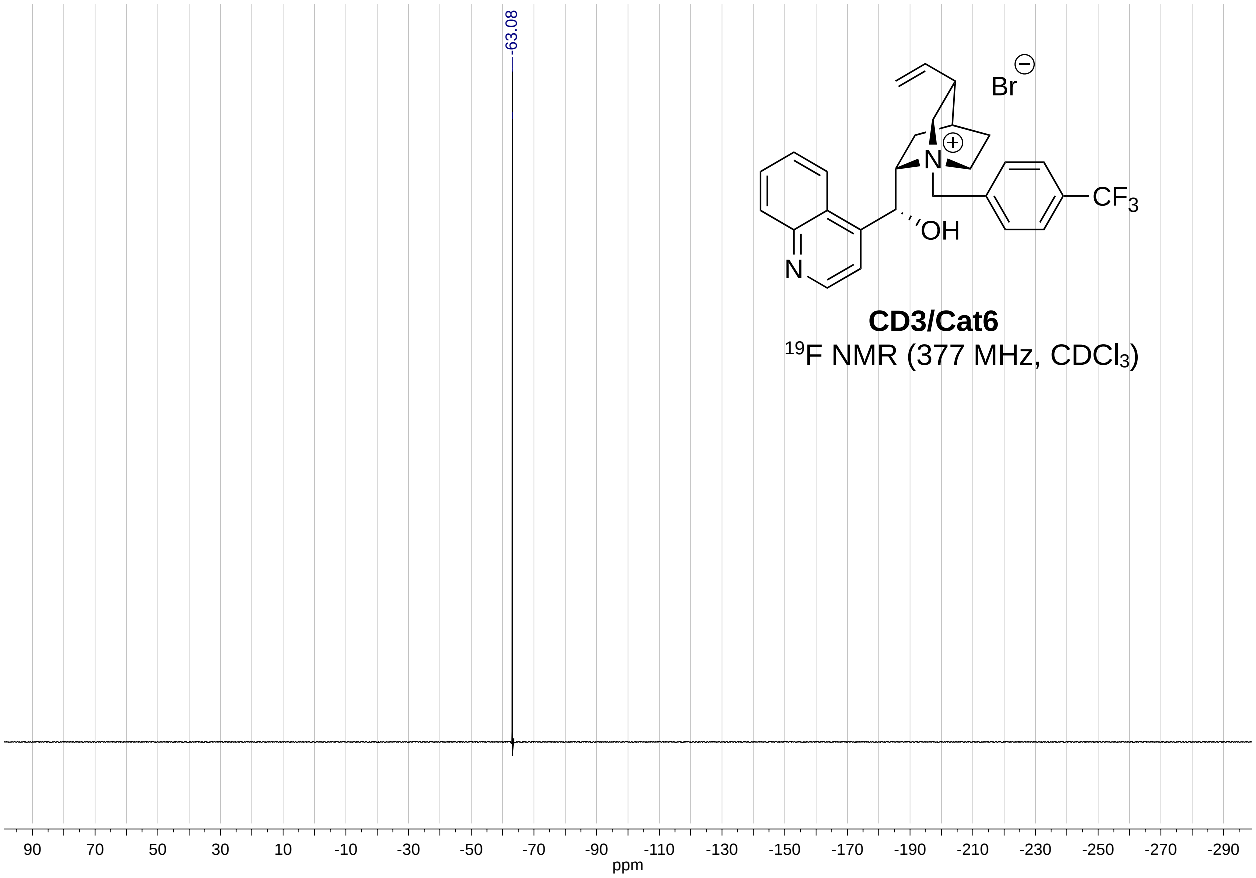
-63.08
Br
OH
N
CF3
CD3/Cat6
19F NMR (377 MHz, CDCl3)
90
70
50
30
10
-10
-30
-50
-70
-90
-110
-130
-150
-170
-190
-210
-230
-250
-270
-290
ppm

CN1
^{1}H NMR (400 MHz, DMSO-d_6)

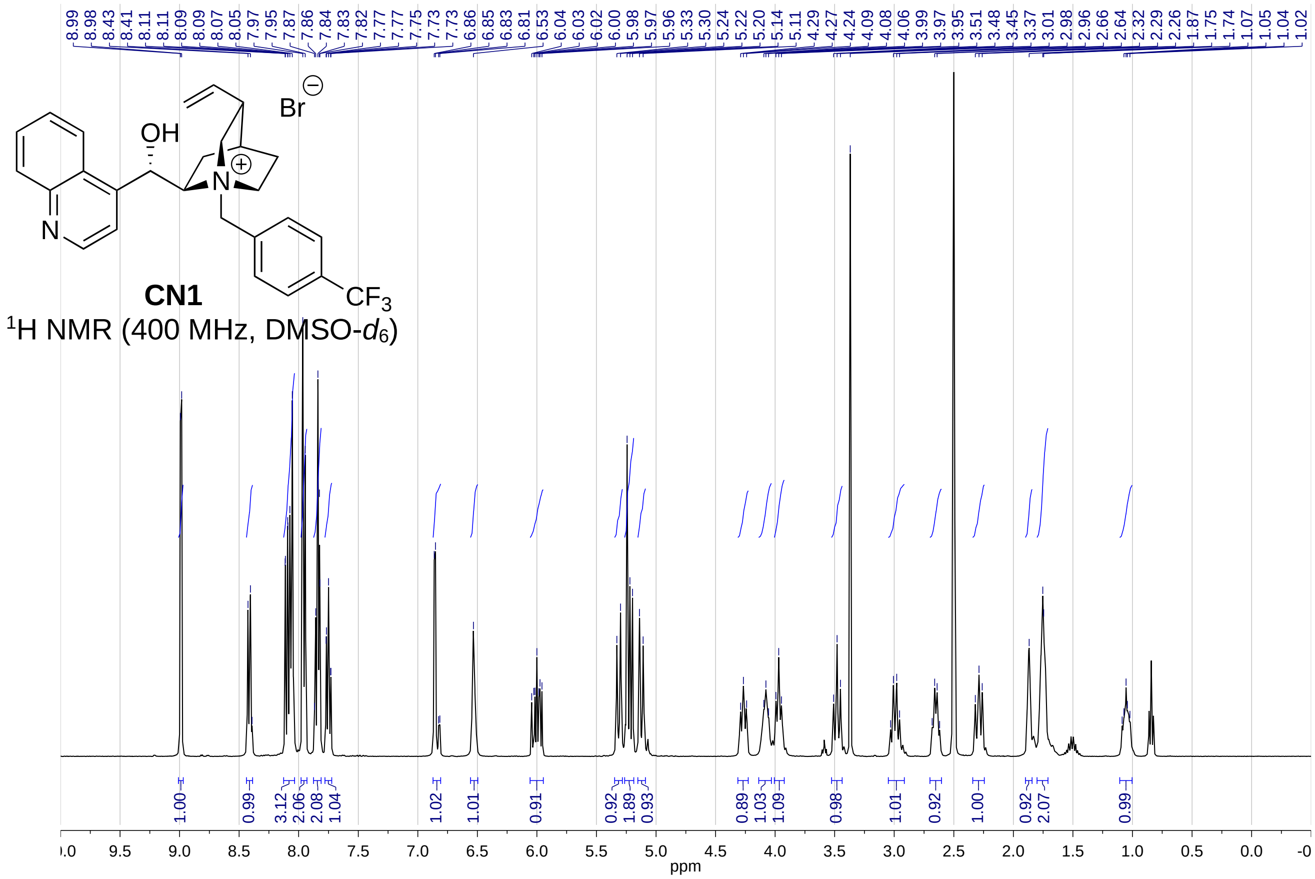

CN1
^{13}C NMR (101 MHz, DMSO-d_6)

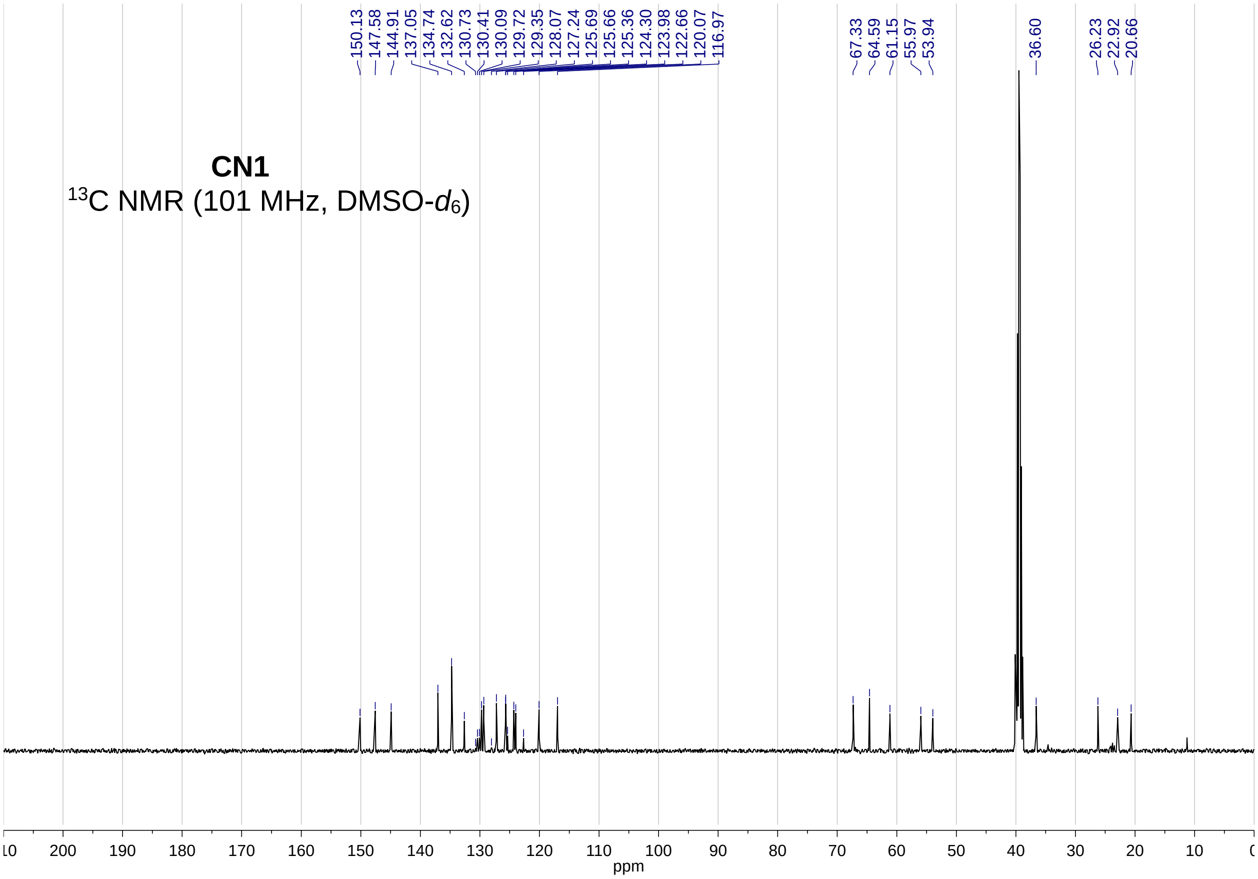

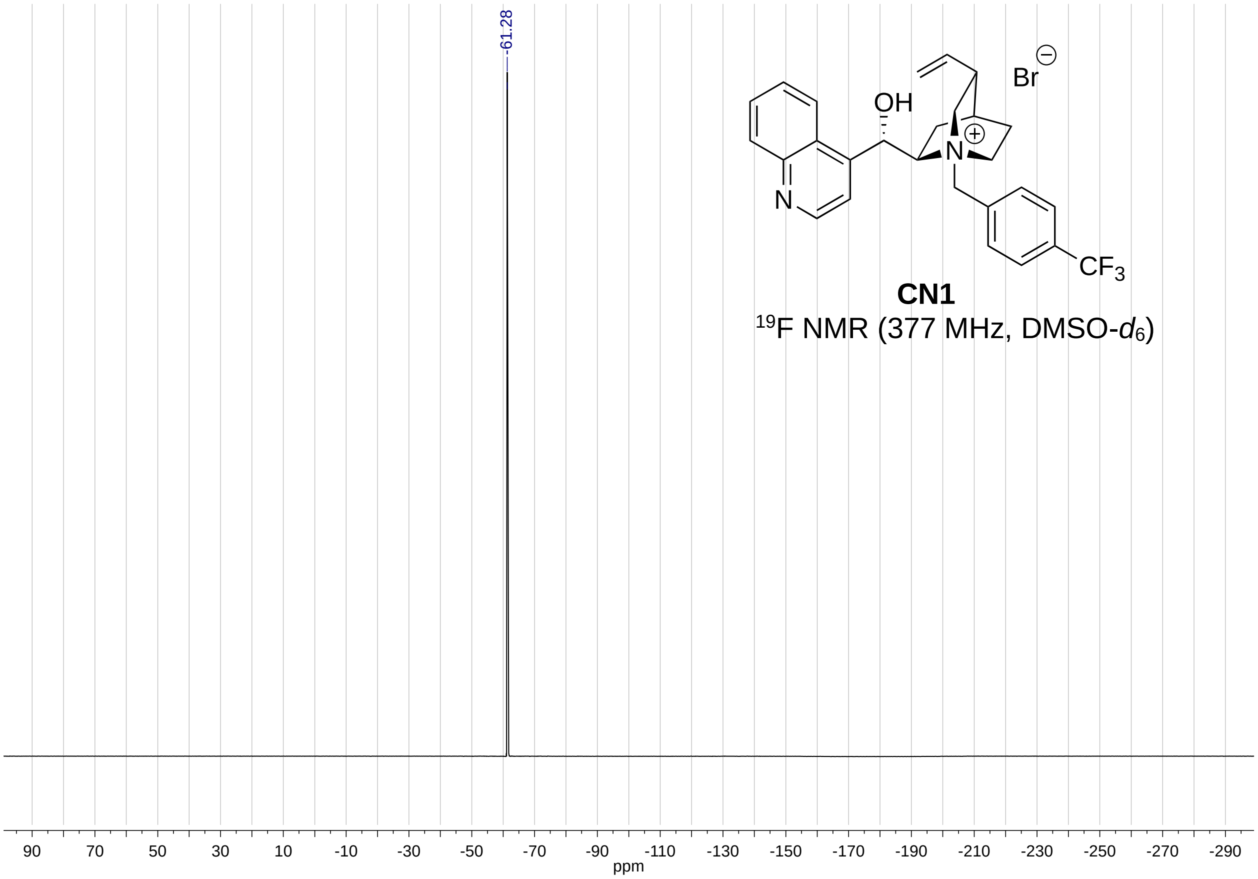

CN1

^{19}F NMR (377 MHz, DMSO-d_6)

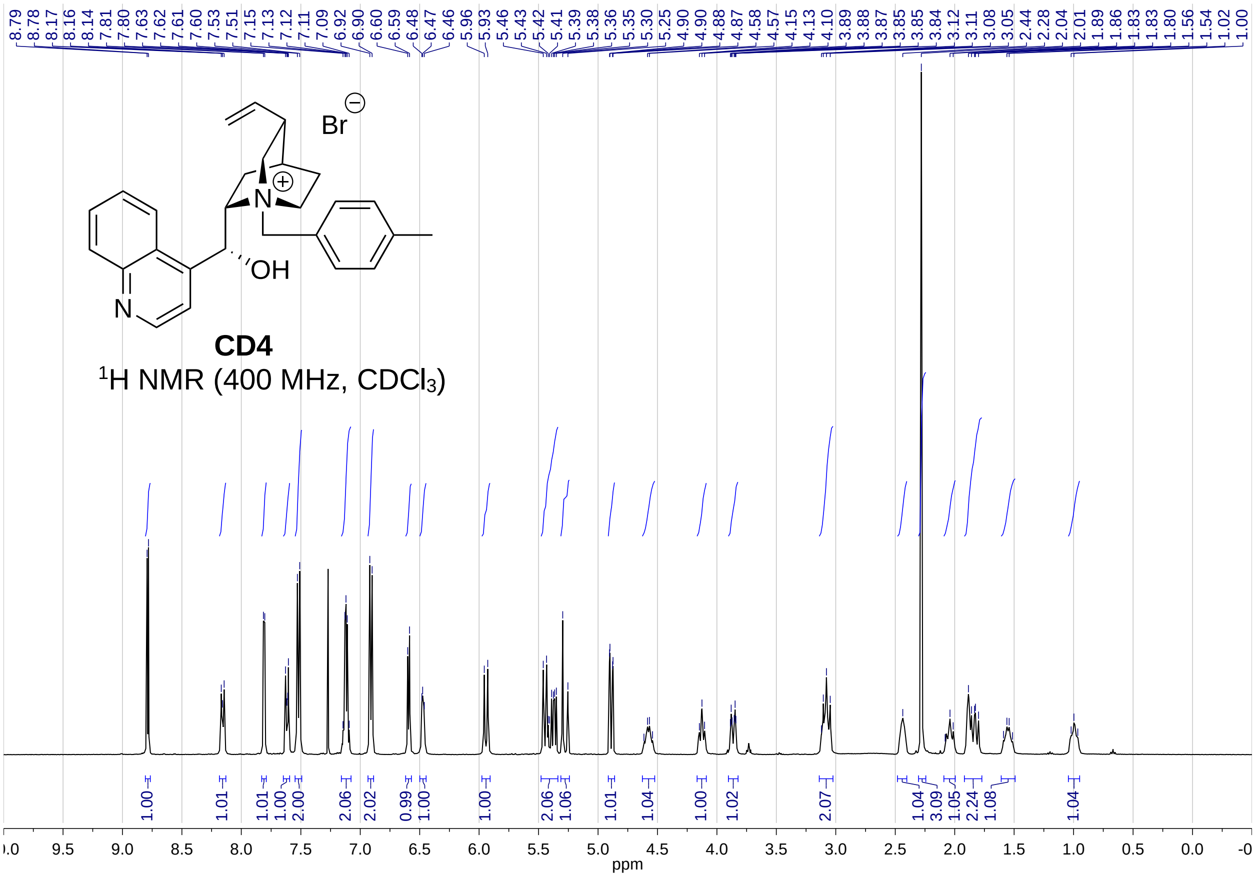
CD4
^{1}H NMR (400 MHz, CDCl$_3$)
ppm

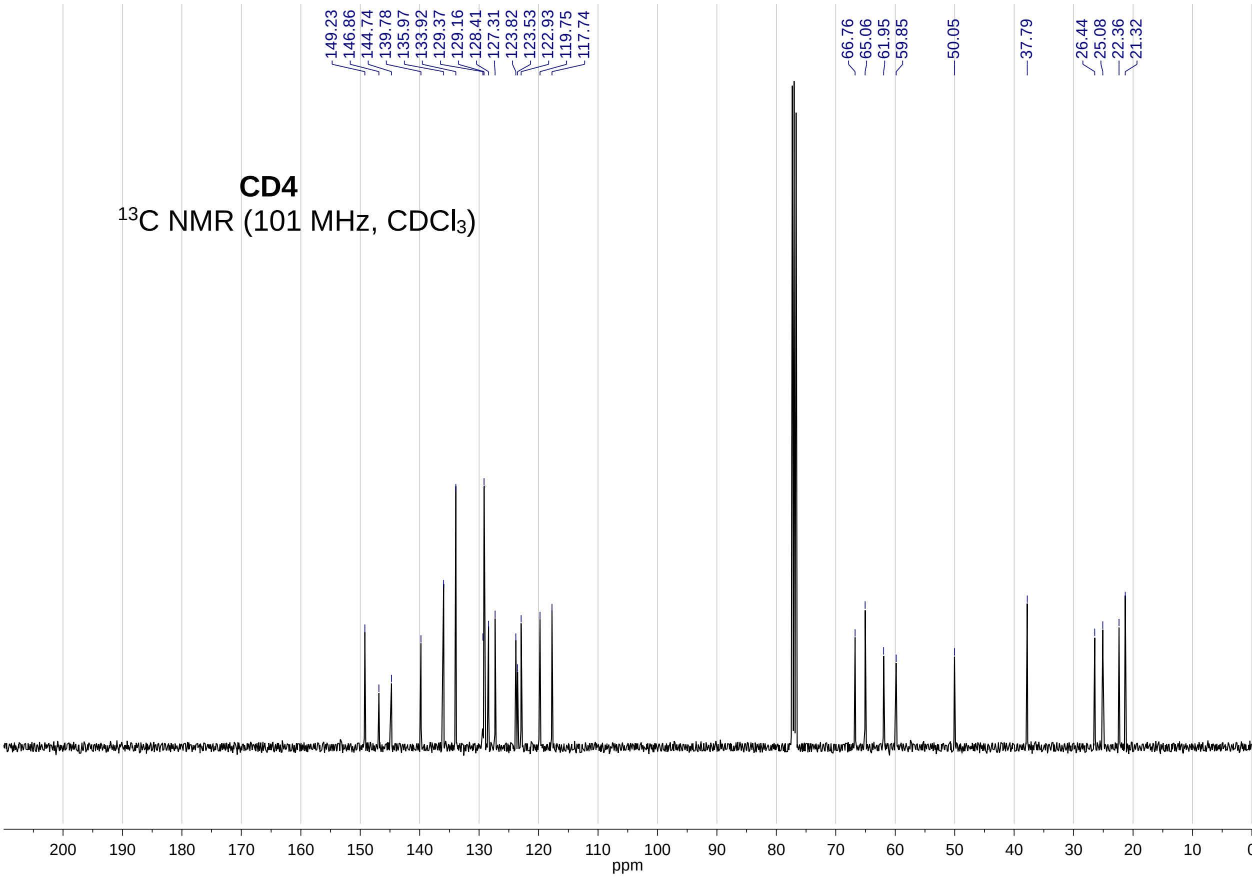
CD4
^{13}C NMR (101 MHz, CDCl$_3$)
ppm

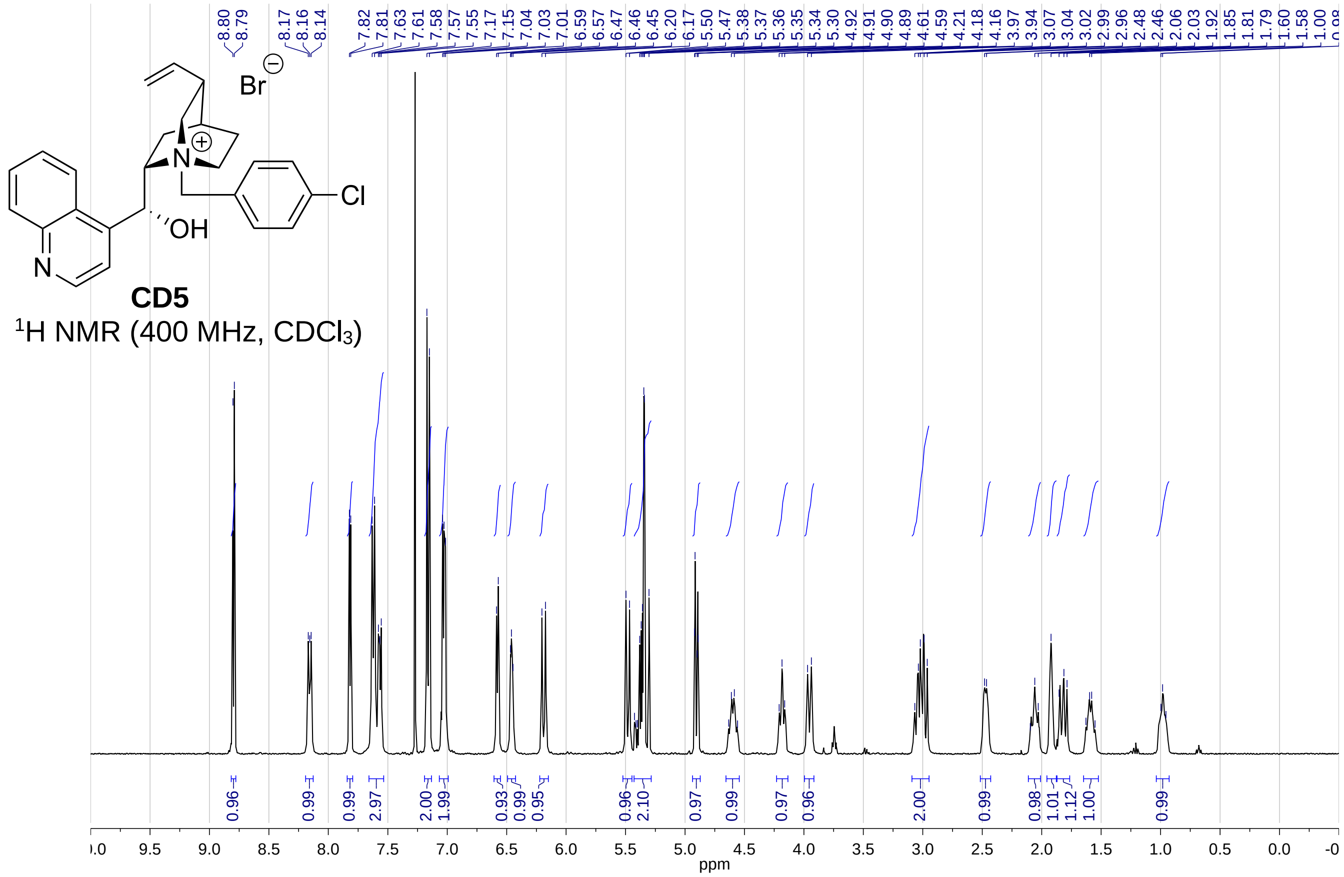

CD5

^{1}H NMR (400 MHz, $CDCl_3$)

CD5

^{13}C NMR (101 MHz, $CDCl_3$)

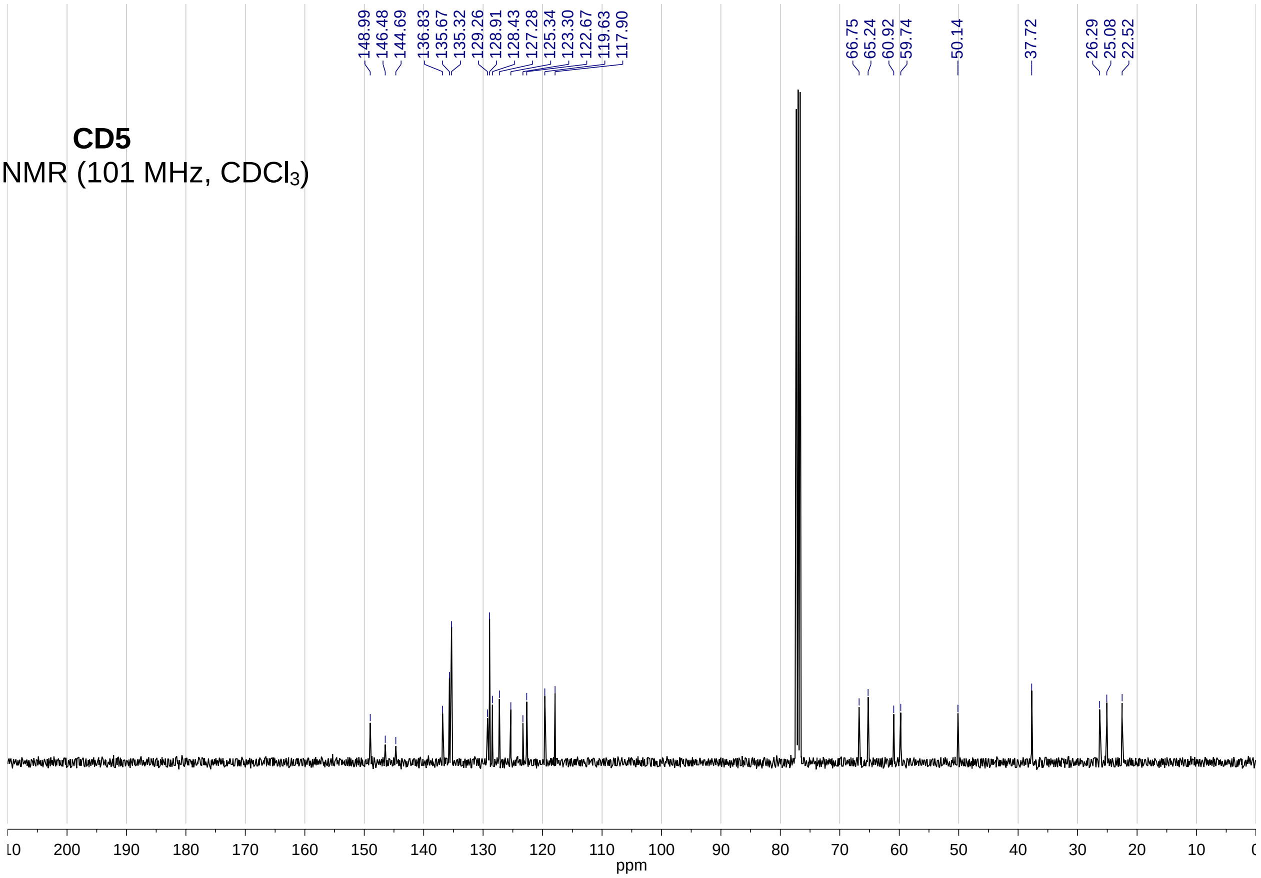

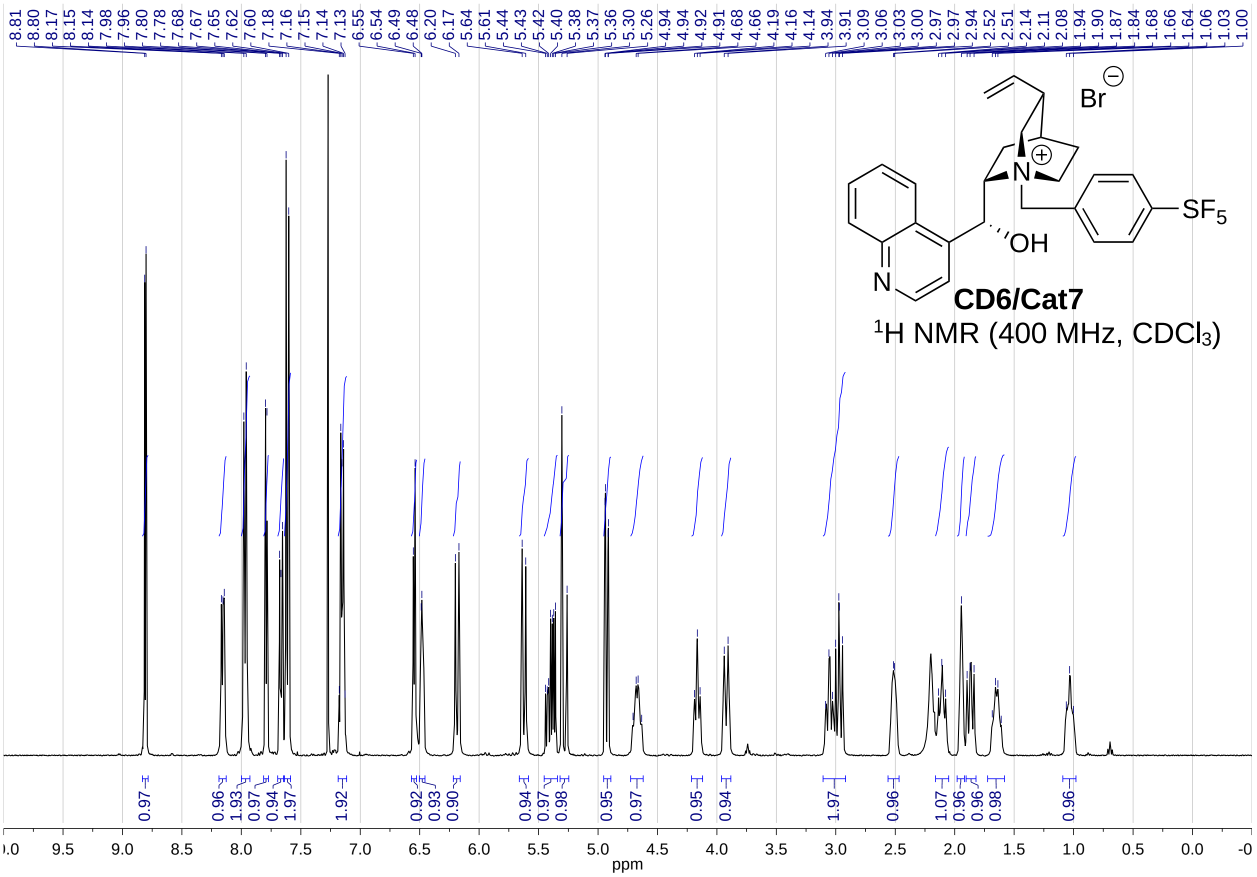
Br
SF5
OH
N
CD6/Cat7
1H NMR (400 MHz, CDCl3)
ppm

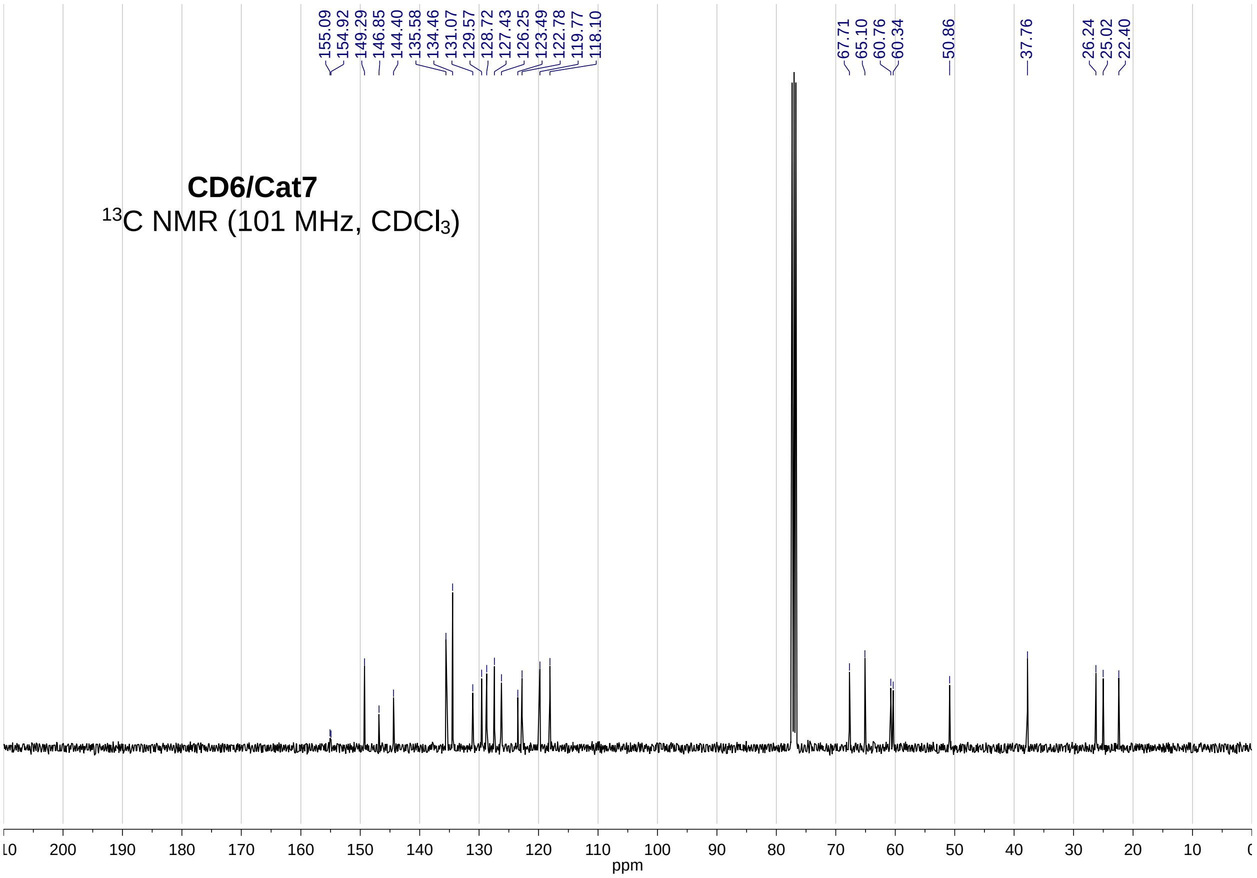
CD6/Cat7
13C NMR (101 MHz, CDCl3)
ppm

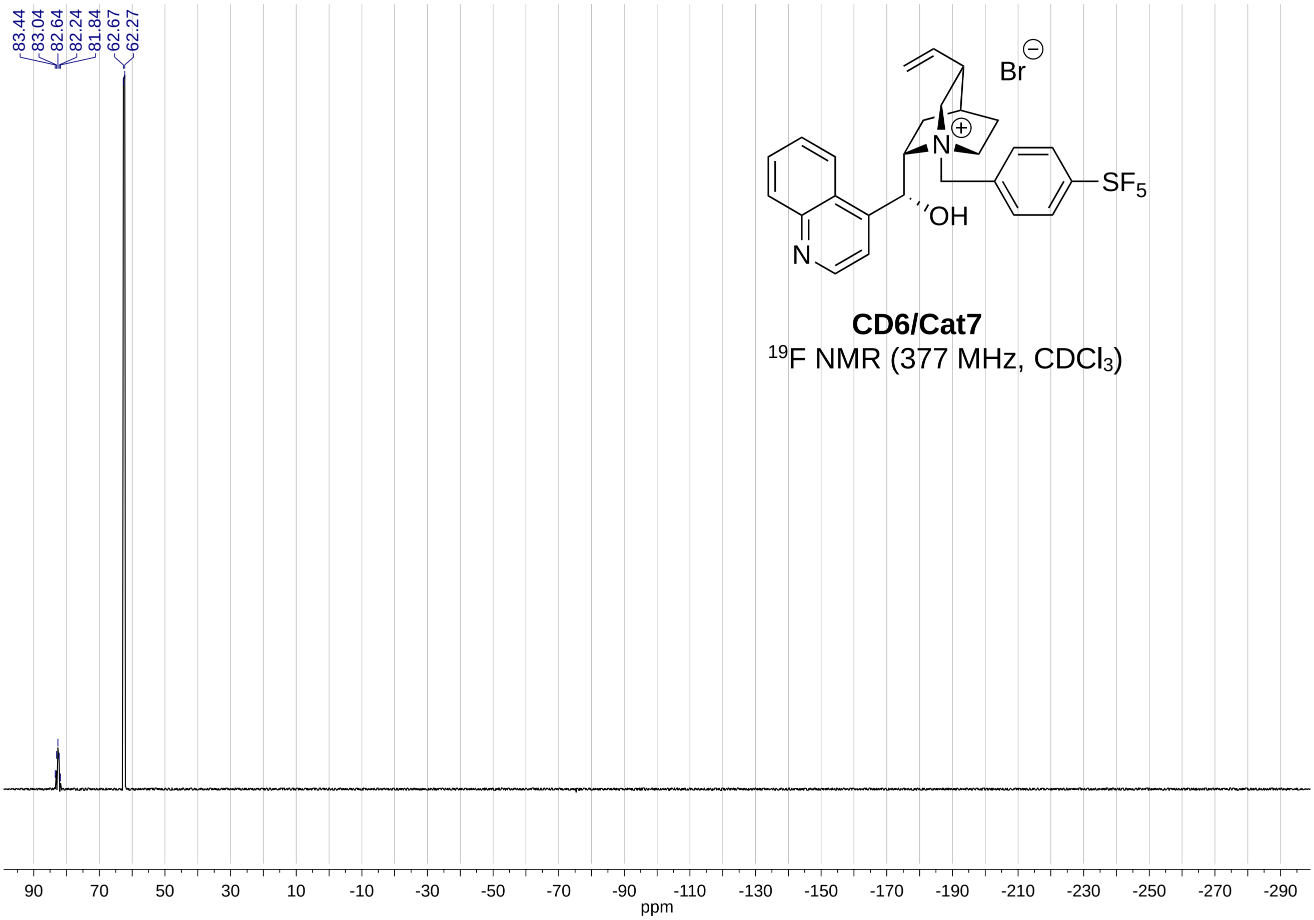

CD6/Cat7

^{19}F NMR (377 MHz, $CDCl_3$)

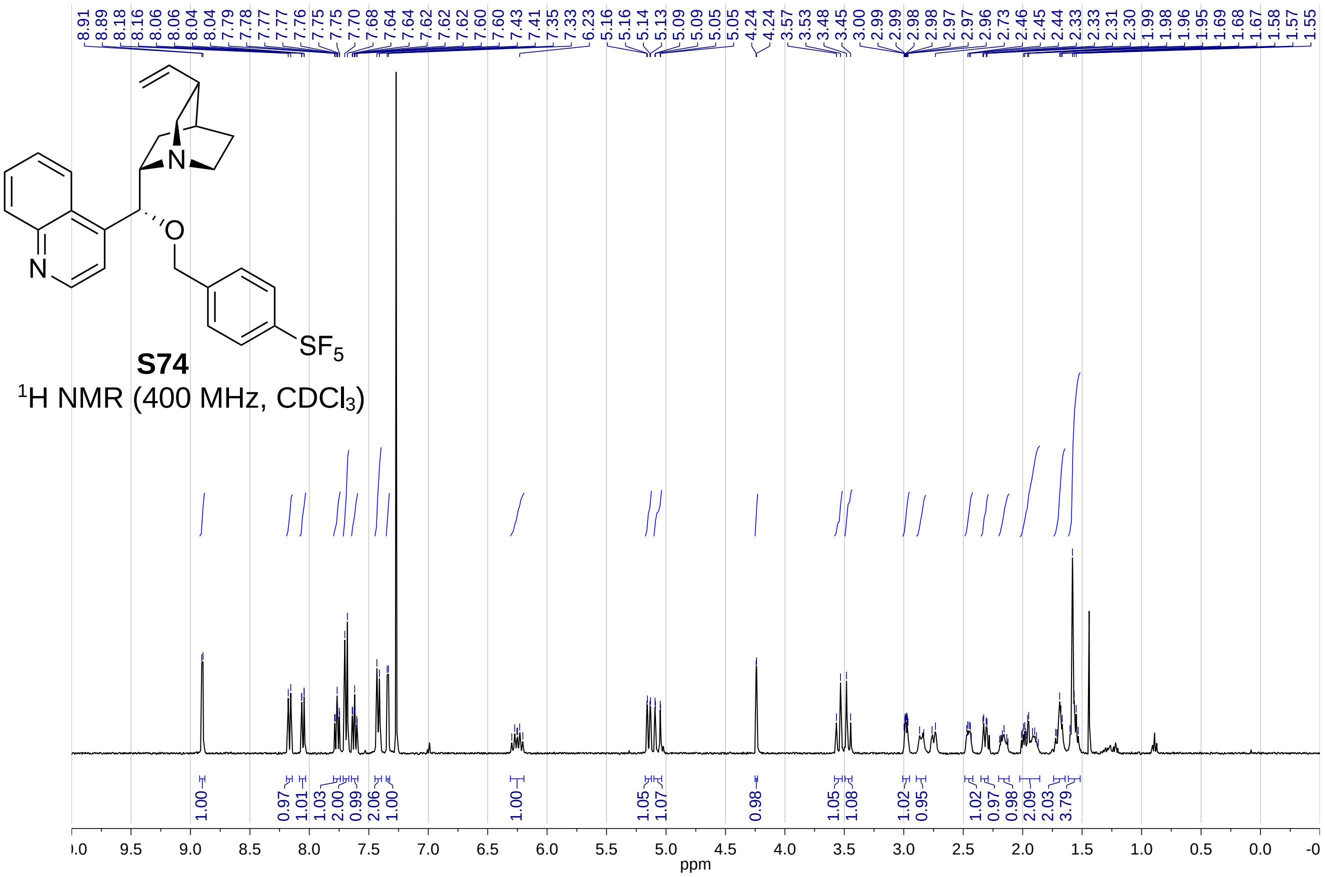

S74

^{1}H NMR (400 MHz, $CDCl_3$)

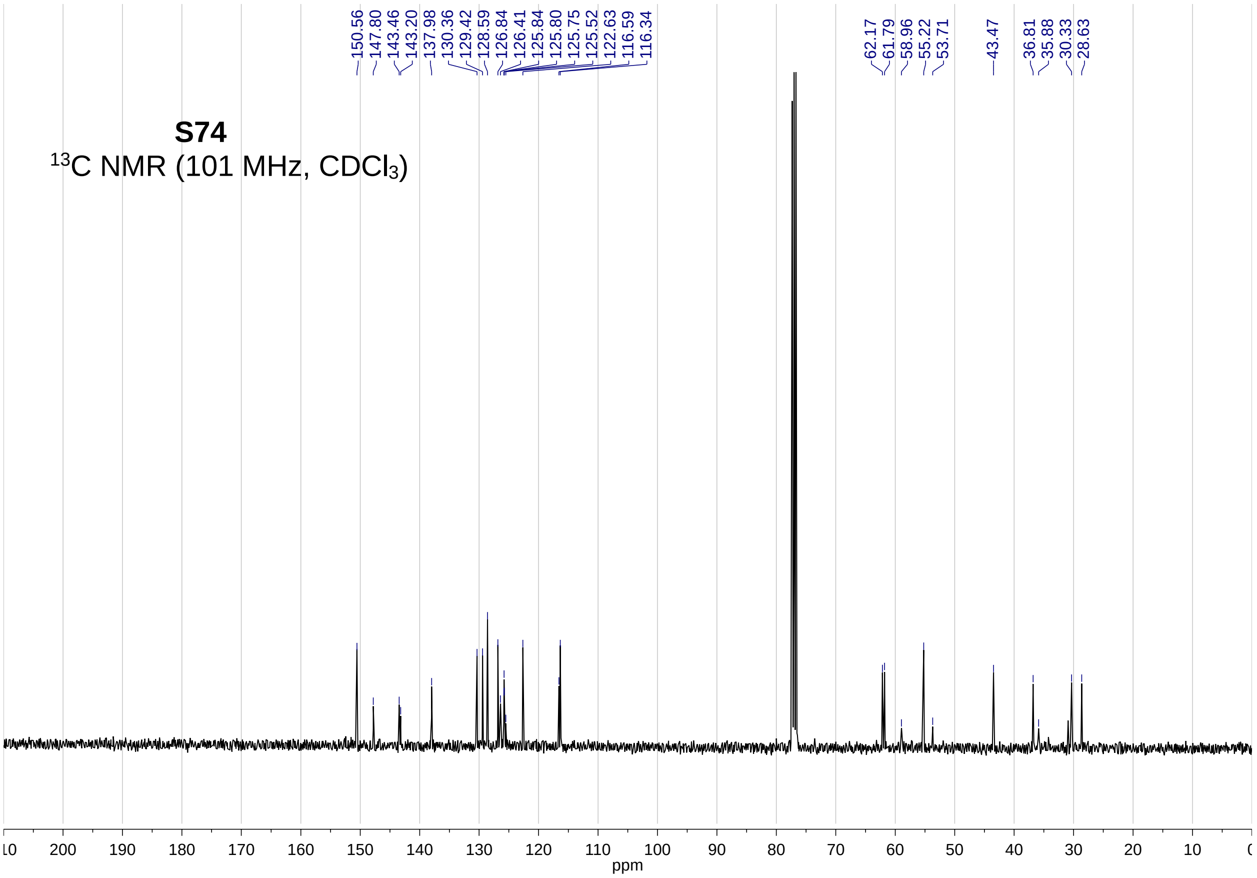

S74

^{13}C NMR (101 MHz, $CDCl_3$)

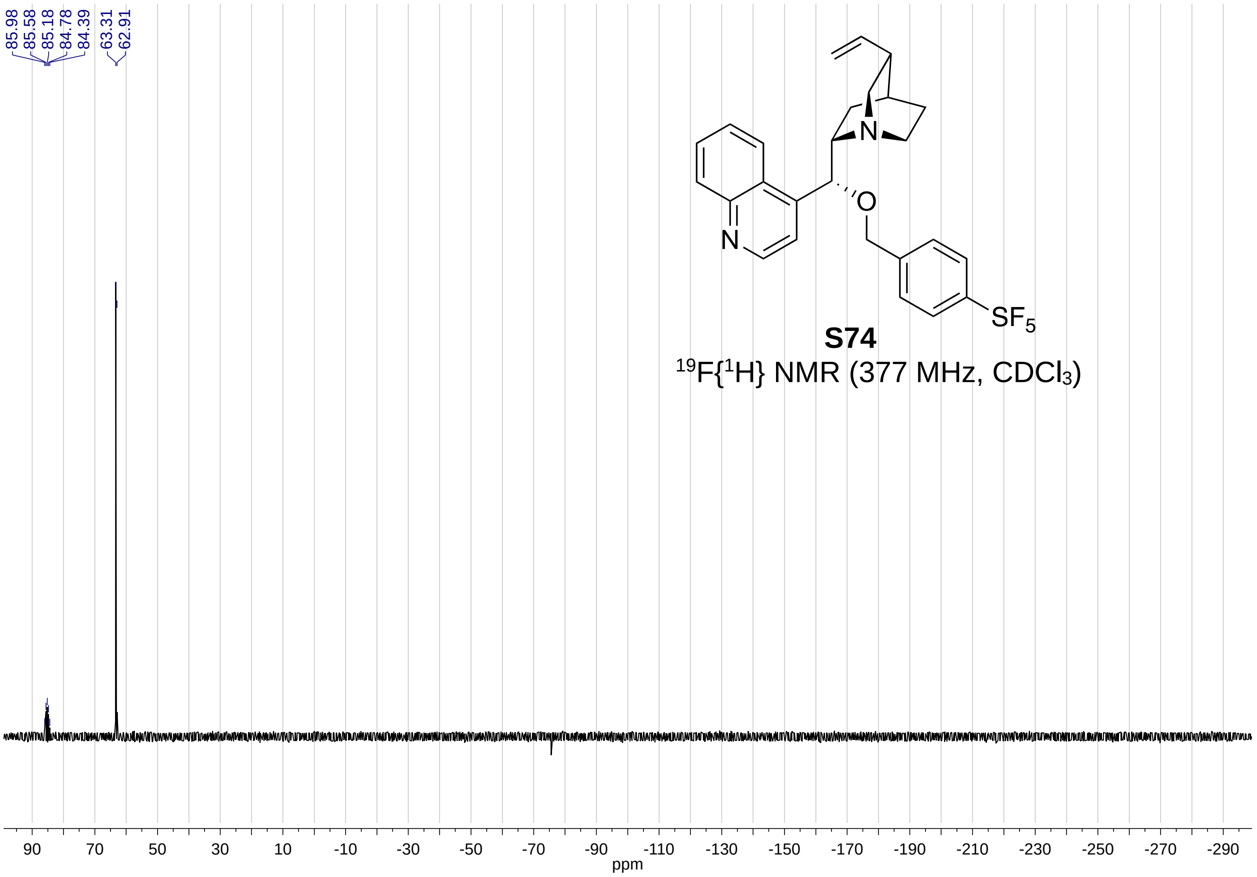
85.98
85.58
85.18
84.78
84.39
63.31
62.91
N
N
O
SF5
S74
19F{1H} NMR (377 MHz, CDCl3)
90
70
50
30
10
-10
-30
-50
-70
-90
-110
-130
-150
-170
-190
-210
-230
-250
-270
-290
ppm

Chiral HPLC Data

tert-Butyl 1'-benzyl-2'-oxospiro[azetidine-2,3'-indoline]-1-carboxylate (3)

Conditions: Chiralpak ID column, 90:10 *n*-hexane:*i*-PrOH, flow rate: 1 mL min^{-1}, 35 ºC, UV detection wavelength: 254 nm

(±)-3

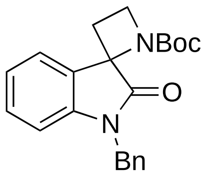

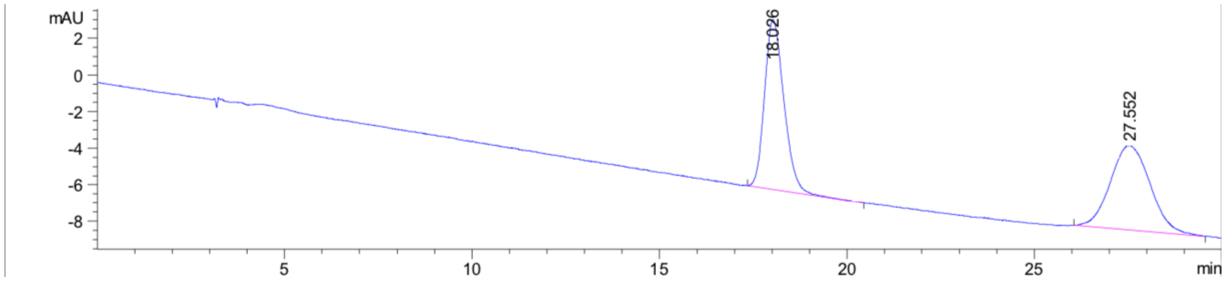

Peak #	RetTime [min]	Type	Width [min]	Area [mAU*s]	Height [mAU]	Area %
1	18.026	BB	0.5456	333.15030	9.26355	49.3461
2	27.552	BB	0.8746	341.97998	4.63588	50.6539
Totals :				675.13028	13.89943	

(-)-(*S*)-3

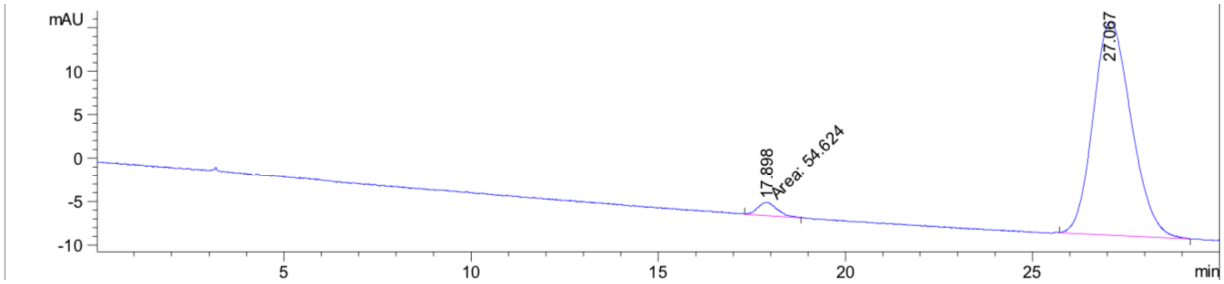

Peak #	RetTime [min]	Type	Width [min]	Area [mAU*s]	Height [mAU]	Area %
1	17.898	MM	0.6028	54.62404	1.51019	3.0559
2	27.067	BB	0.8755	1732.89771	24.55142	96.9441
Totals :				1787.52175	26.06161	

(+)-(*R*)-3

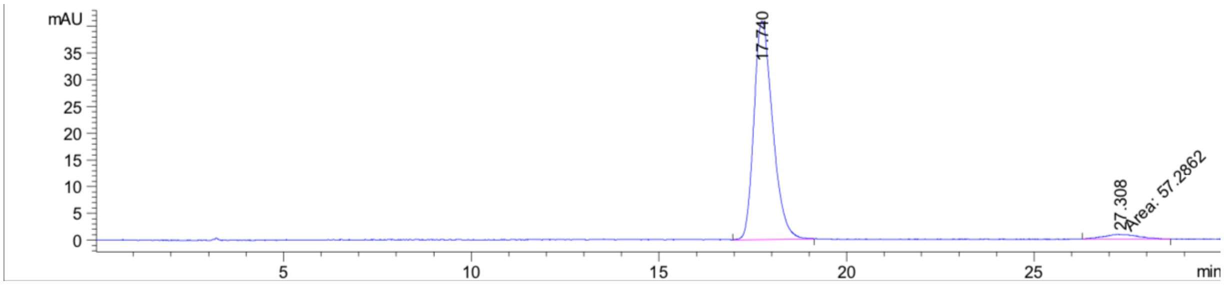

Peak #	Ret Time [min]	Type	Width [min]	Area [mAU*s]	Height [mAU]	Area %
1	17.740	BB	0.5269	1417.22314	40.83775	96.1149
2	27.308	PM	1.0938	57.28624	8.72885e-1	3.8851

Totals : 1474.50938 41.71063

tert-Butyl 1'-benzyl-5'-methoxy-2'-oxospiro[azetidine-2,3'-indoline]-1-carboxylate (4)

Conditions: Chiralpak IA column, 80:20 _n_-hexane:_i_-PrOH, flow rate: 1 mL min^{-1}, 35 ºC, UV detection wavelength: 254 nm

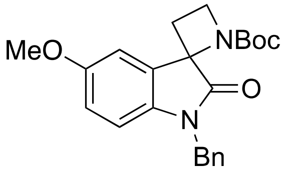

(±)–4

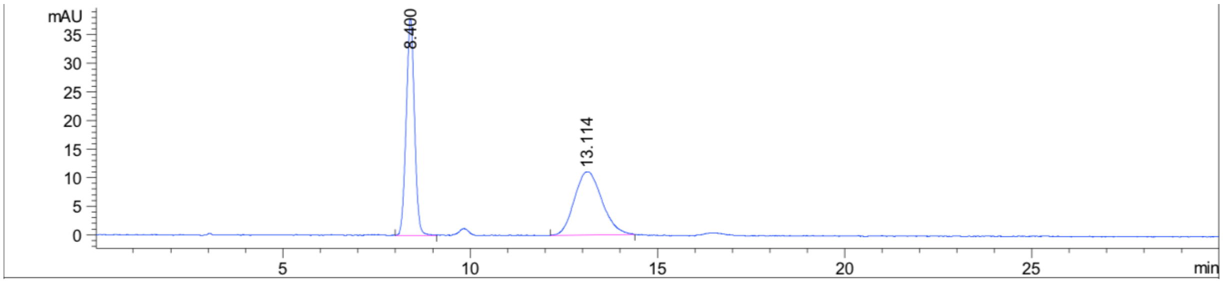

Peak #	Ret Time [min]	Type	Width [min]	Area [mAU*s]	Height [mAU]	Area %
1	8.400	BB	0.2396	583.41754	37.90751	50.8066
2	13.114	BB	0.6100	564.89215	11.05798	49.1934
Totals :				1148.30969	48.96549	

(–)–(_S_)–4

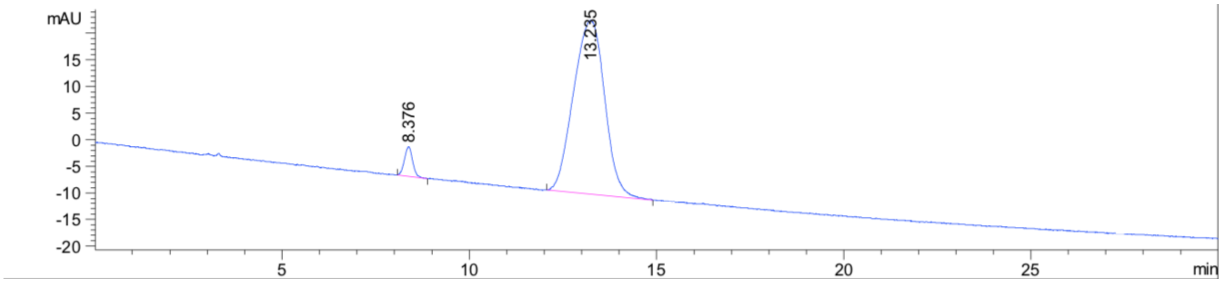

Peak #	Ret Time [min]	Type	Width [min]	Area [mAU*s]	Height [mAU]	Area %
1	8.376	BB	0.2253	84.58729	5.56446	4.3018
2	13.235	BB	0.6867	1881.75085	32.63501	95.6982
Totals :				1966.33814	38.19947	

tert-butyl 1'-benzyl-4'-methyl-2'-oxospiro[azetidine-2,3'-indoline]-1-carboxylate (5)

Conditions: Chiralpak IA column, 90:10 *n*-hexane:*i*-PrOH, flow rate: 1 mL min^{-1}, 35 ºC, UV detection wavelength: 254 nm

(±)–5

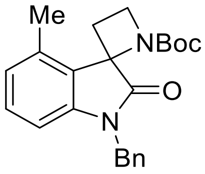

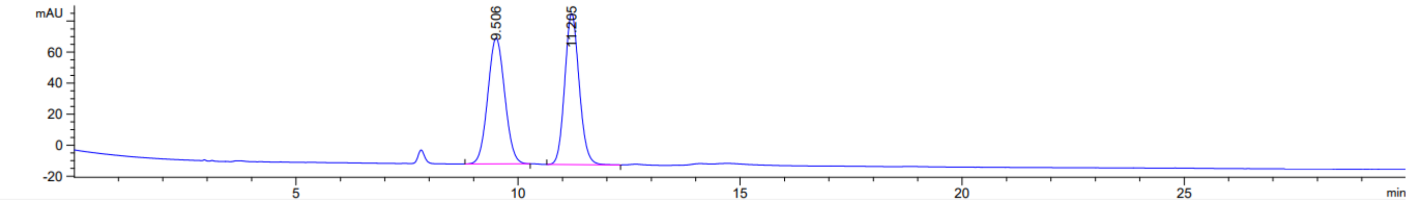

Signal 2: DAD1 B, Sig=254,10 Ref=off

Peak #	RetTime [min]	Type	Width [min]	Area [mAU*s]	Height [mAU]	Area %
1	9.506	BB	0.4146	2159.07593	81.08774	49.5236
2	11.205	BB	0.3515	2200.61523	97.42702	50.4764

Totals : 4359.69116 178.51476

(–)–(*S*)–5

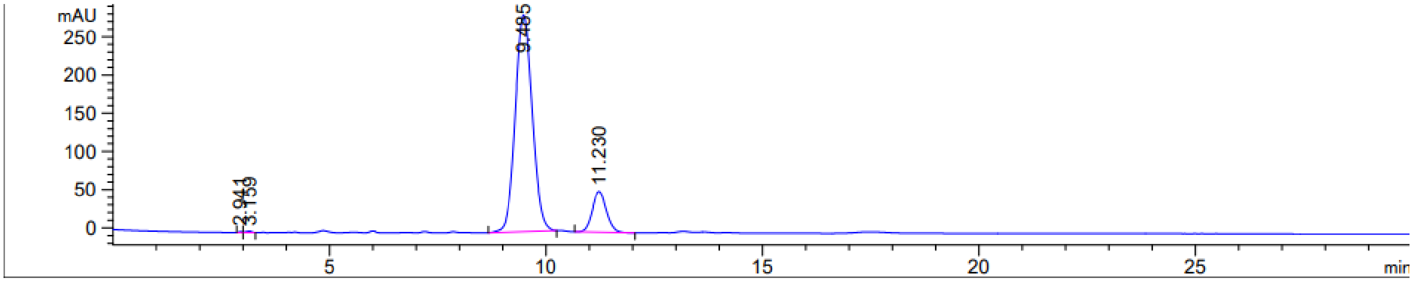

Peak #	RetTime [min]	Type	Width [min]	Area [mAU*s]	Height [mAU]	Area %
1	2.941	BV	0.0787	6.40875	1.31904	0.0717
2	3.159	VB	0.1319	18.37371	1.91571	0.2055
3	9.485	BB	0.4228	7697.79102	283.49500	86.0814
4	11.230	BB	0.3548	1219.88574	53.34302	13.6415

Totals : 8942.45922 340.07277

tert-Butyl 1'-benzyl-5'-methyl-2'-oxospiro[azetidine-2,3'-indoline]-1-carboxylate (6)

Conditions: Chiralpak ID column, 90:10 *n*-hexane:*i*-PrOH, flow rate: 1 mL min^{-1}, 35 ºC, UV detection wavelength: 254 nm

(±)–7

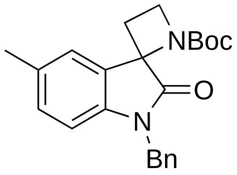

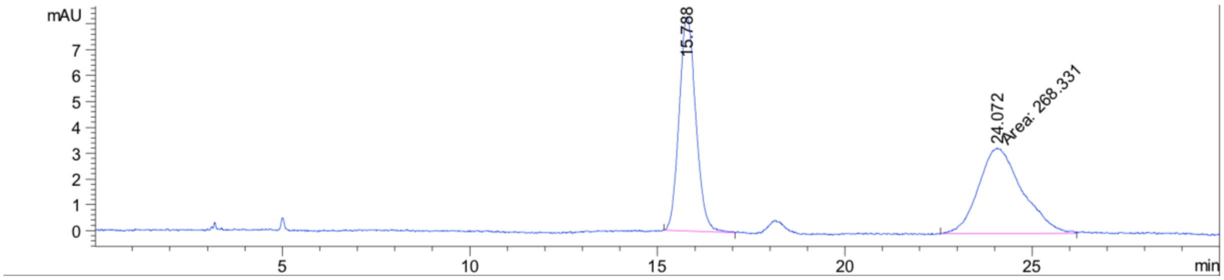

Peak #	Ret Time [min]	Type	Width [min]	Area [mAU*s]	Height [mAU]	Area %
1	15.788	BB	0.4610	256.26868	8.28246	48.8503
2	24.072	PM	1.3500	268.33084	3.31263	51.1497
Totals :				524.59952	11.59509	

(–)–(*S*)–6

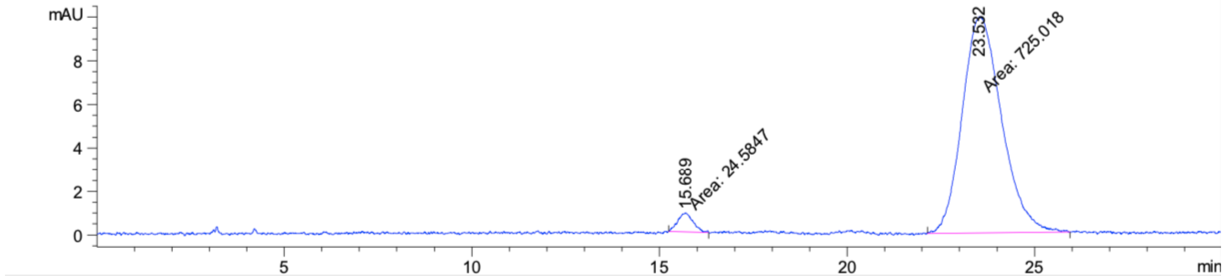

Peak #	Ret Time [min]	Type	Width [min]	Area [mAU*s]	Height [mAU]	Area %
1	15.689	PP	0.4787	24.58469	8.55947e-1	3.2797
2	23.532	MP	1.2158	725.01837	9.93894	96.7203
Totals :				749.60307	10.79488	

***tert*-Butyl 1'-benzyl-5'-fluoro-2'-oxospiro[azetidine-2,3'-indoline]-1-carboxylate (7)**

Conditions: Chiralpak ID column, 90:10 *n*-hexane:*i*-PrOH, flow rate: 1 mL min^{-1}, 35 ºC, UV detection wavelength: 254 nm

(±)–7

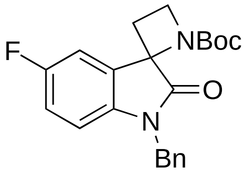

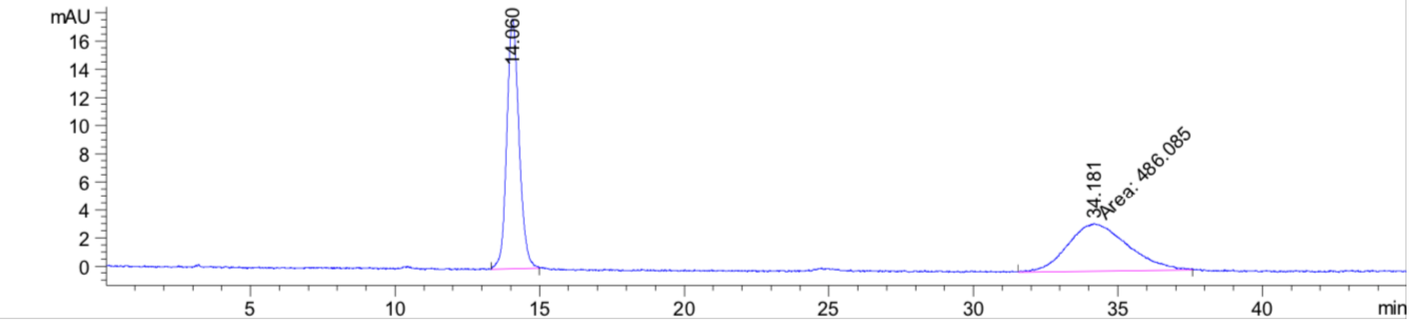

Peak #	Ret Time [min]	Type	Width [min]	Area [mAU*s]	Height [mAU]	Area %
1	14.060	BB	0.4468	520.76111	17.72309	51.7220
2	34.181	MM	2.3896	486.08478	3.39033	48.2780
Totals :				1006.84589	21.11343	

(–)–(*S*)-7

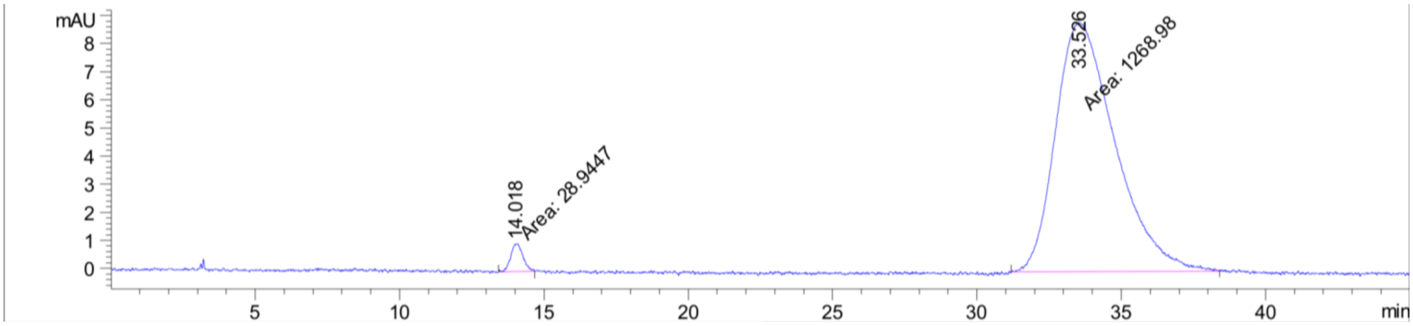

Peak #	Ret Time [min]	Type	Width [min]	Area [mAU*s]	Height [mAU]	Area %
1	14.018	MP	0.4917	28.94473	9.81207e-1	2.2301
2	33.526	PM	2.3872	1268.98315	8.85965	97.7699
Totals :				1297.92788	9.84086	

tert-Butyl 1'-benzyl-7'-fluoro-2'-oxospiro[azetidine-2,3'-indoline]-1-carboxylate (8)

Conditions: Chiralpak ID column, 90:10 *n*-hexane:*i*-PrOH, flow rate: 1 mL min^{-1}, 35 ºC, UV detection wavelength: 254 nm

(±)–8

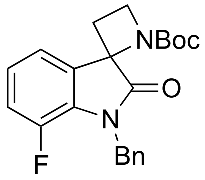

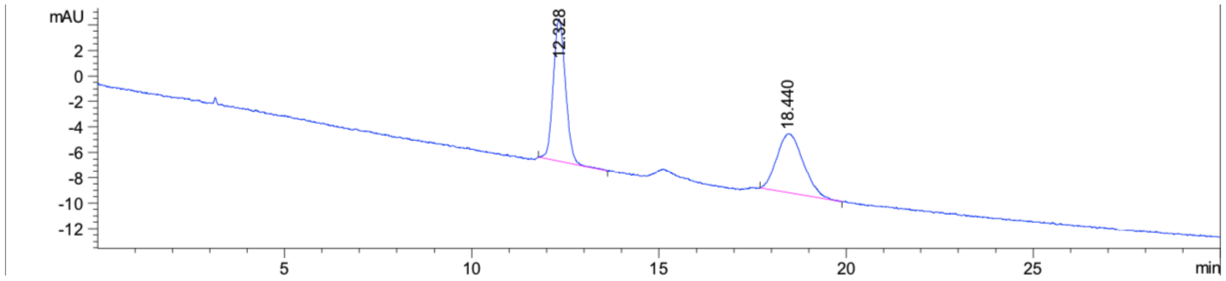

Peak #	Ret Time [min]	Type	Width [min]	Area [mAU*s]	Height [mAU]	Area %
1	12.328	BB	0.3419	254.60260	11.17275	52.4086
2	18.440	BB	0.6037	231.20050	4.63665	47.5914
Totals :				485.80310	15.80941	

(–)–(*S*)–8

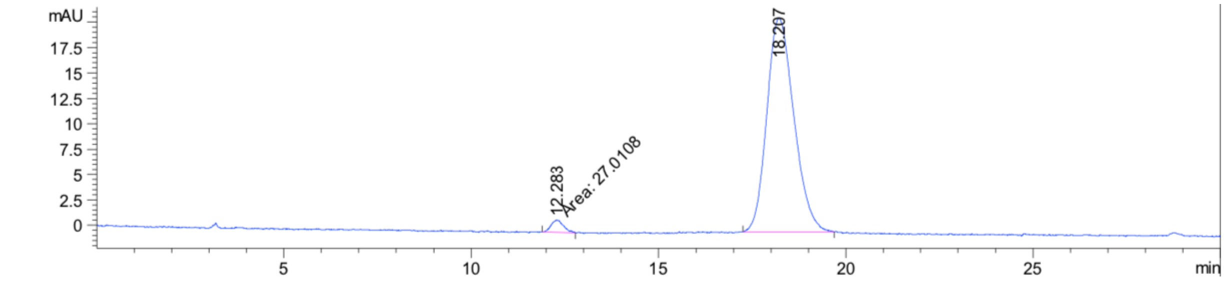

Peak #	Ret Time [min]	Type	Width [min]	Area [mAU*s]	Height [mAU]	Area %
1	12.283	MP	0.3790	27.01079	1.18786	2.4858
2	18.207	BB	0.7005	1059.60339	21.07650	97.5142
Totals :				1086.61419	22.26437	

tert-butyl 1'-benzyl-4'-bromo-2'-oxospiro[azetidine-2,3'-indoline]-1-carboxylate (9)

Conditions: Chiralpak IA column, 90:10 _n_-hexane:_i_-PrOH, flow rate: 1 mL min^{-1}, 35 ºC, UV detection wavelength: 254 nm

(±)–9

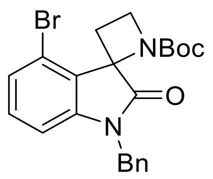

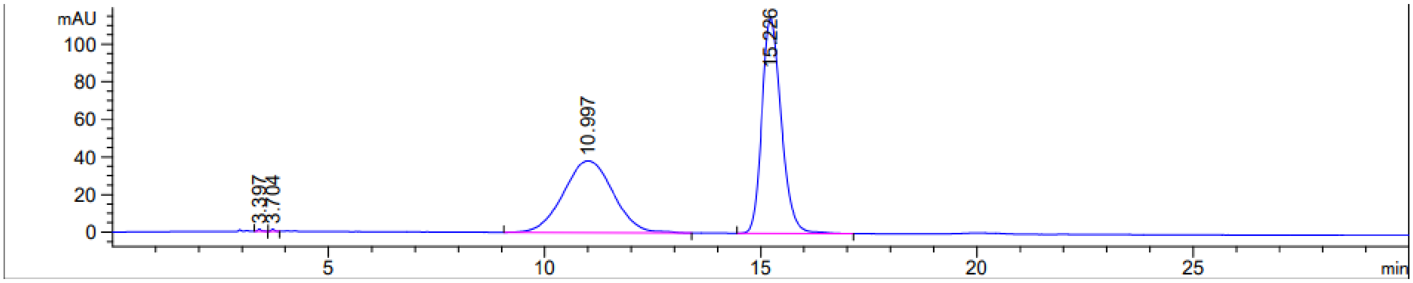

Peak #	RetTime [min]	Type	Width [min]	Area [mAU*s]	Height [mAU]	Area %
1	3.397	BB	0.0803	6.65112	1.24620	0.1001
2	3.704	BB	0.0696	5.80204	1.26374	0.0873
3	10.997	BB	1.1737	3065.92651	38.30988	46.1449
4	15.226	BB	0.4797	3565.74878	114.39133	53.6677

Totals : 6644.12845 155.21114

(±)–9

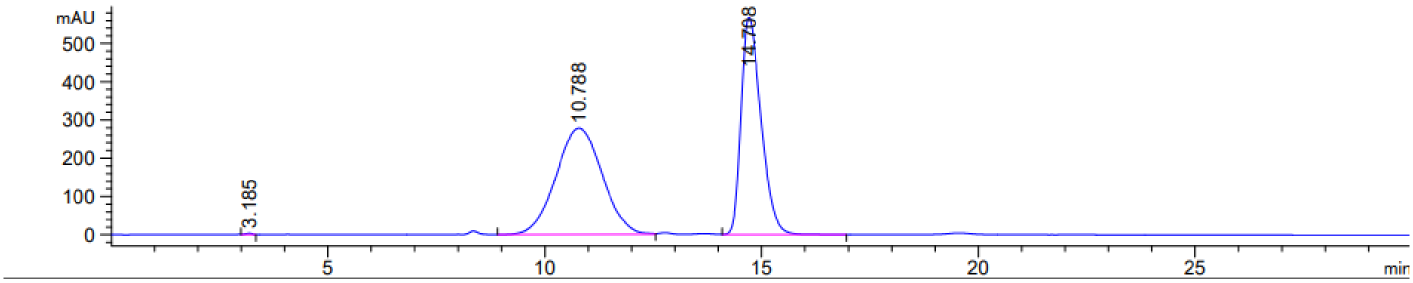

Peak #	RetTime [min]	Type	Width [min]	Area [mAU*s]	Height [mAU]	Area %
1	3.185	VB	0.1410	36.02195	3.59232	0.0922
2	10.788	BV	1.1586	2.07285e4	278.42987	53.0466
3	14.708	VB	0.4983	1.83115e4	567.72827	46.8612

Totals : 3.90760e4 849.75046

tert-Butyl 1'-benzyl-5'-bromo-2'-oxospiro[azetidine-2,3'-indoline]-1-carboxylate (10)

Conditions: Chiralpak IC column, 90:10 *n*-hexane:*i*-PrOH, flow rate: 1 mL min^{-1}, 35 ºC, UV detection wavelength: 254 nm

(±)–10

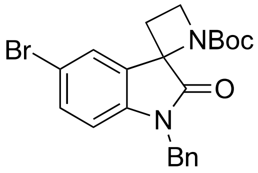

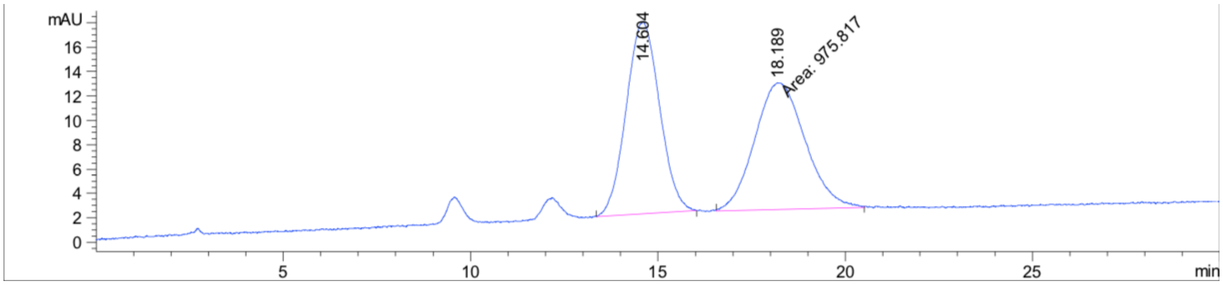

Peak #	Ret Time [min]	Type	Width [min]	Area [mAU*s]	Height [mAU]	Area %
1	14.604	BB	0.7431	978.13226	15.78033	50.0593
2	18.189	MM	1.5618	975.81659	10.41315	49.9407
Totals :				1953.94885	26.19347	

(–)–(*S*)–10

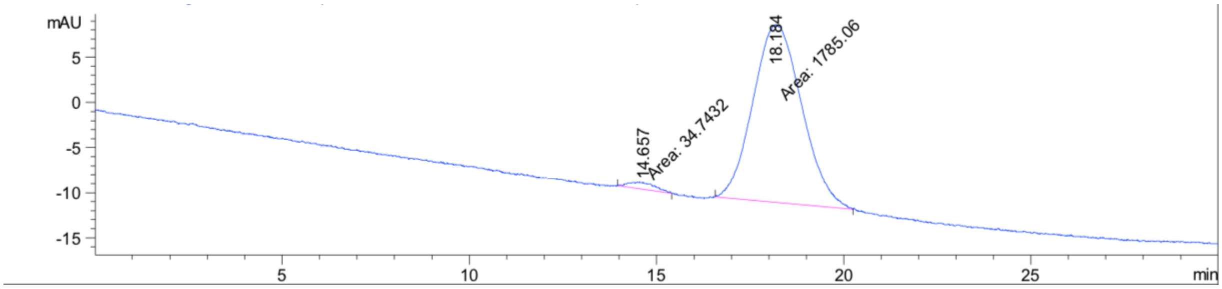

Peak #	Ret Time [min]	Type	Width [min]	Area [mAU*s]	Height [mAU]	Area %
1	14.657	PP	0.8110	34.74319	7.14008e-1	1.9092
2	18.184	MM	1.5118	1785.05896	19.67957	98.0908
Totals :				1819.80215	20.39358	

tert-butyl 1'-benzyl-6'-bromo-2'-oxospiro[azetidine-2,3'-indoline]-1-carboxylate (11)

Conditions: Chiralpak IC column, 90:10 *n*-hexane:*i*-PrOH, flow rate: 1 mL min^{-1}, 35 ºC, UV detection wavelength: 254 nm

(±)–11

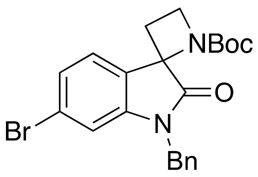

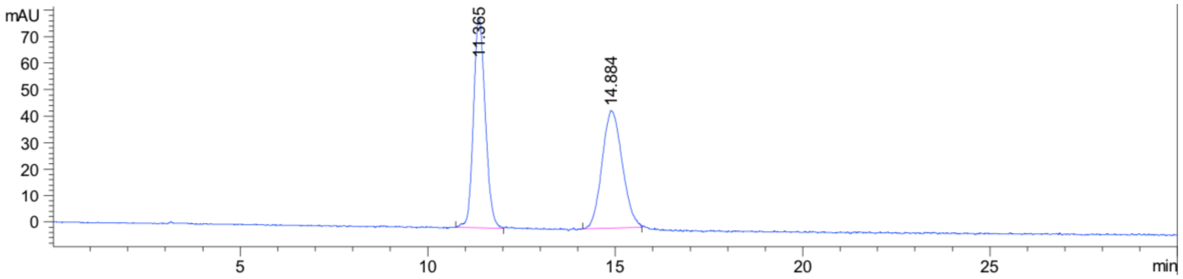

Peak #	Ret Time [min]	Type	Width [min]	Area [mAU*s]	Height [mAU]	Area %
1	11.365	BV	0.3298	1744.56006	79.56335	51.0347
2	14.884	BV	0.4514	1673.82007	44.64238	48.9653
Totals :				3418.38013	124.20573	

(–)–(*S*)–11

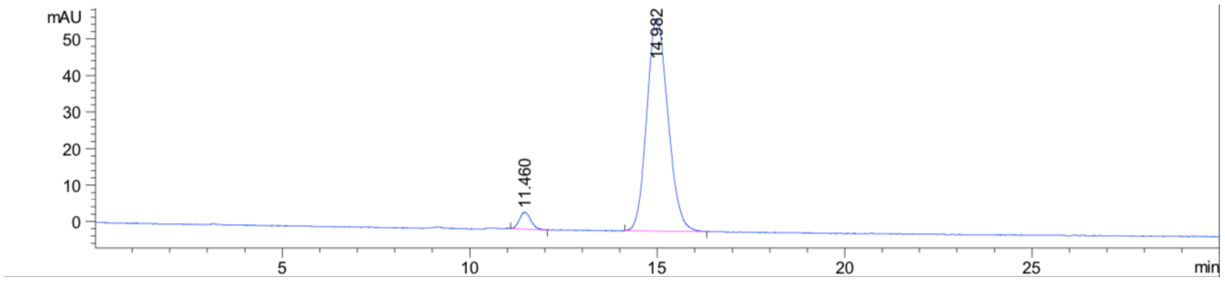

Peak #	Ret Time [min]	Type	Width [min]	Area [mAU*s]	Height [mAU]	Area %
1	11.460	BB	0.2978	96.84792	4.55978	4.0686
2	14.982	BB	0.6121	2283.53540	58.15580	95.9314
Totals :				2380.38332	62.71559	

tert-Butyl 1'-benzyl-7'-bromo-2'-oxospiro[azetidine-2,3'-indoline]-1-carboxylate (12)

Conditions: Chiralpak IC column, 90:10 _n_-hexane:_i_-PrOH, flow rate: 1 mL min^{-1}, 35 ºC, UV detection wavelength: 254 nm

(±)–12

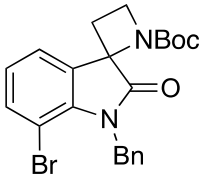

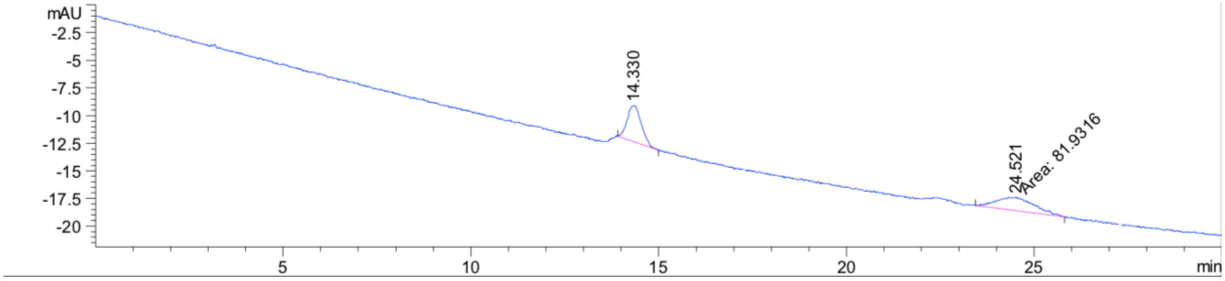

Peak #	Ret Time [min]	Type	Width [min]	Area [mAU*s]	Height [mAU]	Area %
1	14.330	BB	0.3275	84.98497	3.24541	50.9146
2	24.521	PP	1.1325	81.93165	1.20573	49.0854
Totals :				166.91662	4.45114	

(+)–(_R_)–12

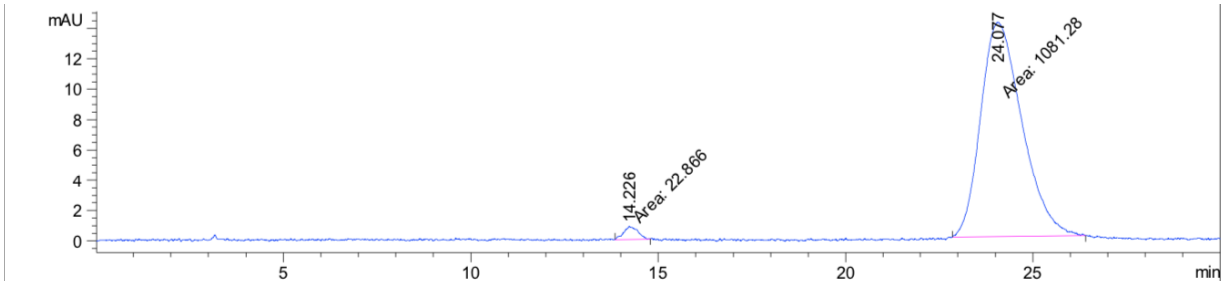

Peak #	Ret Time [min]	Type	Width [min]	Area [mAU*s]	Height [mAU]	Area %
1	14.226	MM	0.4404	22.86604	8.65271e-1	2.0709
2	24.077	PM	1.2770	1081.27954	14.11210	97.9291
Totals :				1104.14558	14.97737	

tert-Butyl 1'-benzyl-5'-chloro-2'-oxospiro[azetidine-2,3'-indoline]-1-carboxylate (13)

Conditions: Chiralpak IE column, 90:10 _n_-hexane:_i_-PrOH, flow rate: 1 mL min^{-1}, 35 ºC, UV detection wavelength: 254 nm

(±)–13

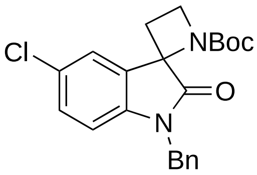

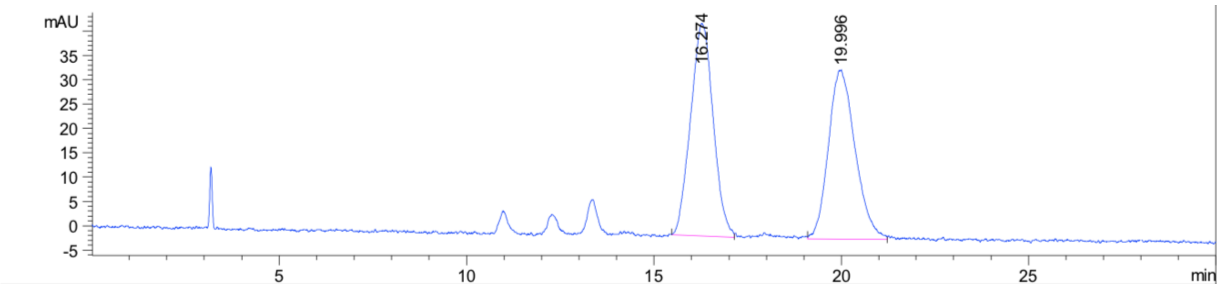

Peak #	Ret Time [min]	Type	Width [min]	Area [mAU*s]	Height [mAU]	Area %
1	16.274	BV	0.5034	1831.85645	43.67122	51.5426
2	19.996	VV	0.5968	1722.20837	34.71099	48.4574
Totals :				3554.06482	78.38221	

(–)–(_S_)–13

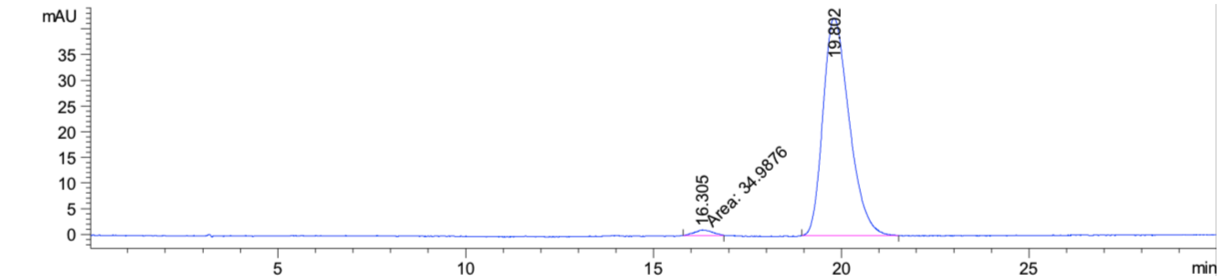

Peak #	Ret Time [min]	Type	Width [min]	Area [mAU*s]	Height [mAU]	Area %
1	16.305	MM	0.5448	34.98760	1.07041	1.6718
2	19.802	BB	0.7035	2057.87939	42.30371	98.3282
Totals :				2092.86699	43.37411	

tert-Butyl 1'-benzyl-5'-nitro-2'-oxospiro[azetidine-2,3'-indoline]-1-carboxylate (14)

Conditions: Chiralpak IF column, 90:10 *n*-hexane:*i*-PrOH, flow rate: 1 mL min^{-1}, 35 ºC, UV detection wavelength: 254 nm

(±)–14

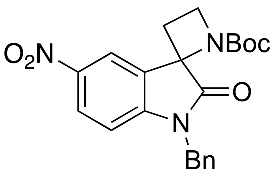

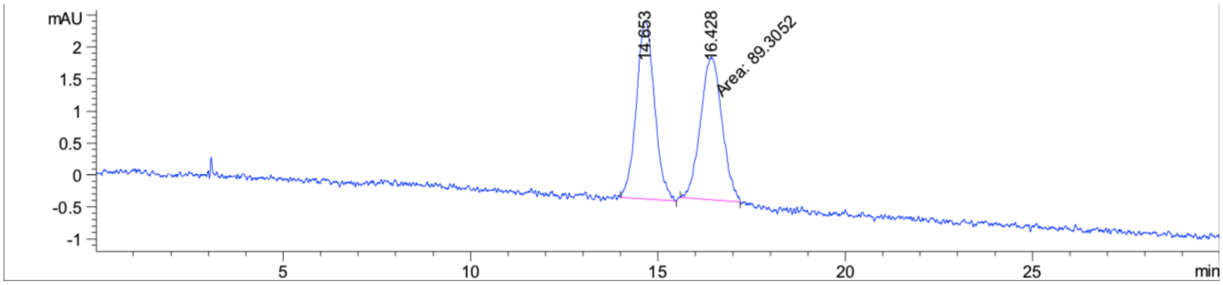

Peak #	Ret Time [min]	Type	Width [min]	Area [mAU*s]	Height [mAU]	Area %
1	14.653	BB	0.4144	94.33975	2.79078	51.3707
2	16.428	PP	0.6676	89.30518	2.22955	48.6293
Totals :				183.64493	5.02033	

(–)–(*S*)–14

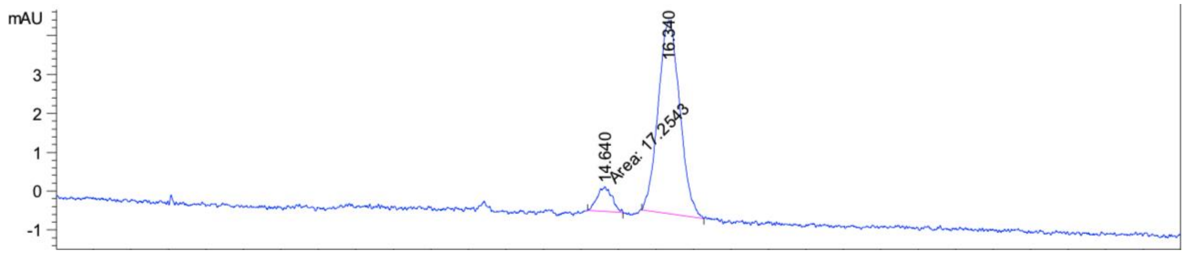

Peak #	Ret Time [min]	Type	Width [min]	Area [mAU*s]	Height [mAU]	Area %
1	14.640	PP	0.4519	17.25432	6.36379e-1	7.9902
2	16.340	BB	0.4907	198.68871	4.96717	92.0098
Totals :				215.94303	5.60355	

tert-butyl 1'-benzyl-2'-oxo-1',2'-dihydrospiro[azetidine-2,3'-pyrrolo[2,3-b]pyridine]-1-carboxylate (15)

Conditions: Chiralpak ID column, 90:10 *n*-hexane:*i*-PrOH, flow rate: 1 mL min^{-1}, 35 ºC, UV detection wavelength: 254 nm

(±)–15

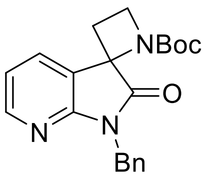

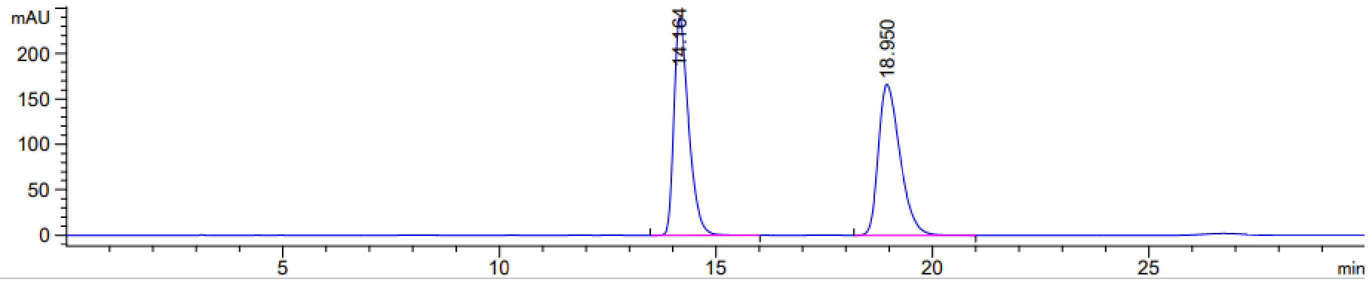

Signal 2: DAD1 B, Sig=254,10 Ref=off

Peak #	RetTime [min]	Type	Width [min]	Area [mAU*s]	Height [mAU]	Area %
1	14.164	BB	0.3671	5732.60596	239.64929	49.9450
2	18.950	BB	0.5337	5745.22656	166.03394	50.0550

Totals : 1.14778e4 405.68323

(–)–(*S*)–15

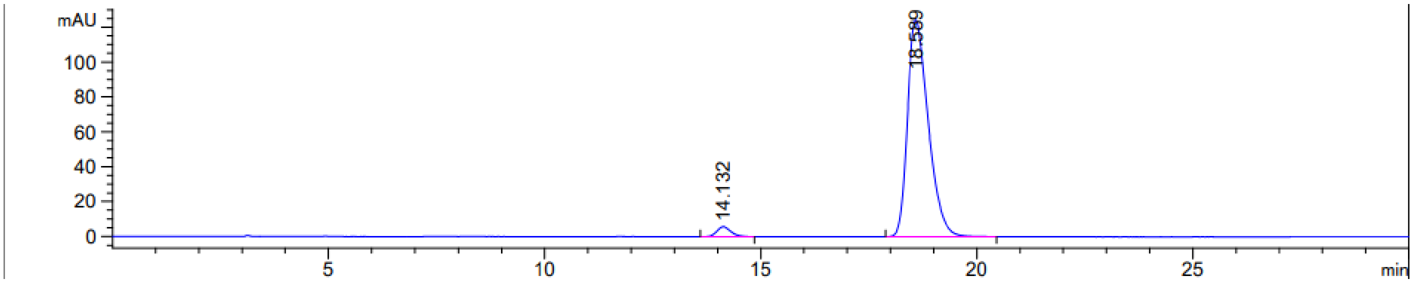

Peak #	RetTime [min]	Type	Width [min]	Area [mAU*s]	Height [mAU]	Area %
1	14.132	BB	0.3397	130.32875	5.72284	3.0406
2	18.589	BB	0.5161	4156.00146	124.29221	96.9594

Totals : 4286.33022 130.01506

tert-Butyl 1'-methyl-2'-oxospiro[azetidine-2,3'-indoline]-1-carboxylate (16)

Conditions: Chiralpak IA column, 95:5 *n*-hexane:*i*-PrOH, flow rate: 1 mL min^{-1}, 35 ºC, UV detection wavelength: 254 nm

(±)–16

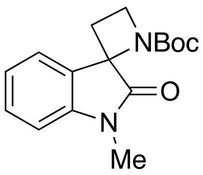

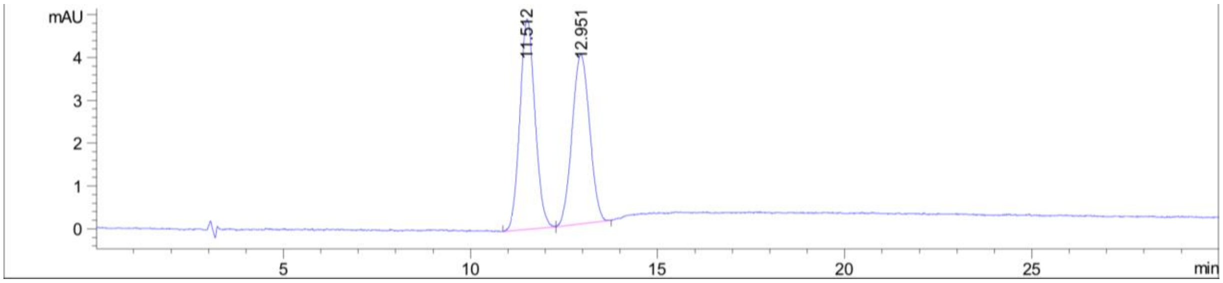

Peak #	Ret Time [min]	Type	Width [min]	Area [mAU*s]	Height [mAU]	Area %
1	11.512	BB	0.4287	138.43642	4.91338	51.7541
2	12.951	BB	0.4895	129.05222	3.96786	48.2459
Totals :				267.48863	8.88124	

(–)–(*S*)–16

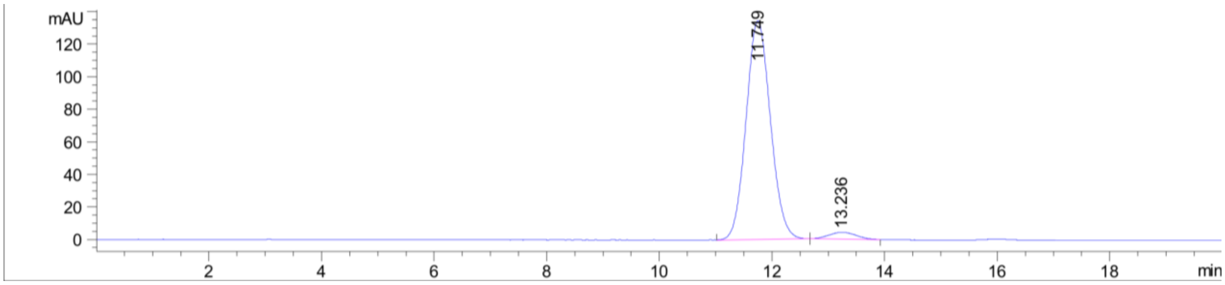

Peak #	Ret Time [min]	Type	Width [min]	Area [mAU*s]	Height [mAU]	Area %
1	11.749	BB	0.4663	4034.90308	134.37186	96.6522
2	13.236	BB	0.4017	139.76117	4.16239	3.3478
Totals :				4174.66425	138.53424	

tert-Butyl 1'-(4-methoxybenzyl)-2'-oxospiro[azetidine-2,3'-indoline]-1-carboxylate (17)

Conditions: Chiralpak IA column, 90:10 *n*-hexane:*i*-PrOH, flow rate: 1 mL min^{-1}, 35 ºC, UV detection wavelength: 254 nm

(±)–17

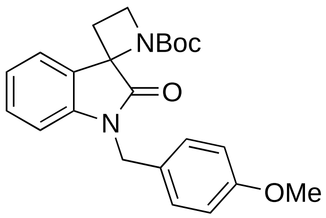

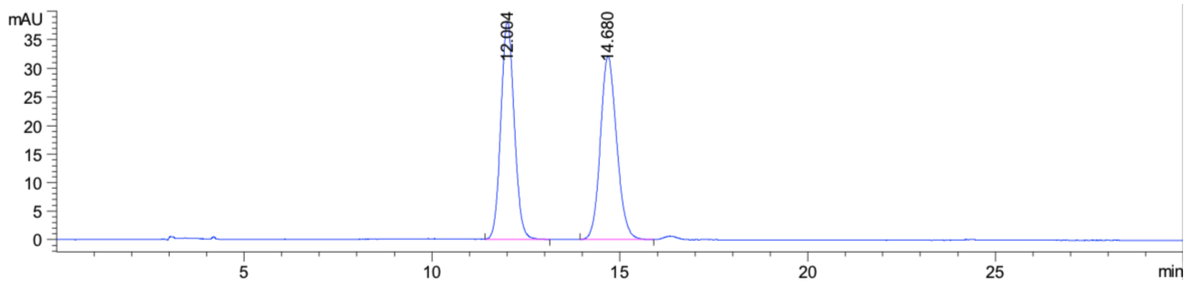

Peak #	Ret Time [min]	Type	Width [min]	Area [mAU*s]	Height [mAU]	Area %
1	12.004	BB	0.3849	944.28143	38.13831	49.3758
2	14.680	BB	0.4599	968.15479	32.09934	50.6242
Totals :				1912.43622	70.23765	

(–)–(*S*)–17

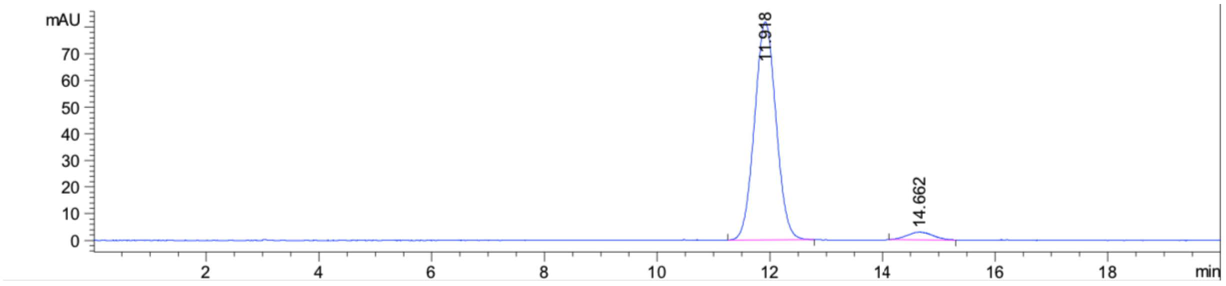

Peak #	Ret Time [min]	Type	Width [min]	Area [mAU*s]	Height [mAU]	Area %
1	11.918	BB	0.4158	2170.44214	81.73095	95.9089
2	14.662	BB	0.4039	92.58223	2.88870	4.0911
Totals :				2263.02437	84.61965	

tert-Butyl 1'-(4-bromobenzyl)-2'-oxospiro[azetidine-2,3'-indoline]-1-carboxylate (18)

Conditions: Chiralpak ID column, 90:10 *n*-hexane:*i*-PrOH, flow rate: 1 mL min^{-1}, 35 ºC, UV detection wavelength: 254 nm

(±)–18

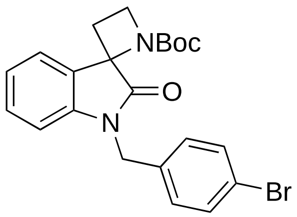

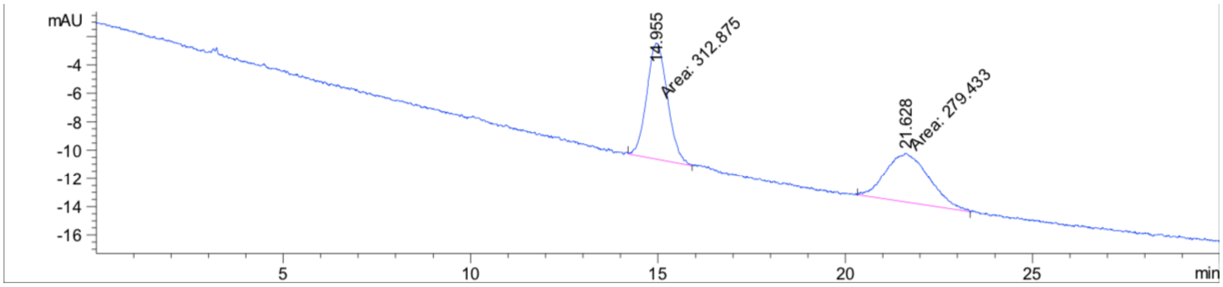

Peak #	Ret Time [min]	Type	Width [min]	Area [mAU*s]	Height [mAU]	Area %
1	14.955	MM	0.6401	312.87549	8.14698	52.8231
2	21.628	PP	1.3424	279.43311	3.46921	47.1769

Totals : 592.30859 11.61620

(–)–(*S*)–18

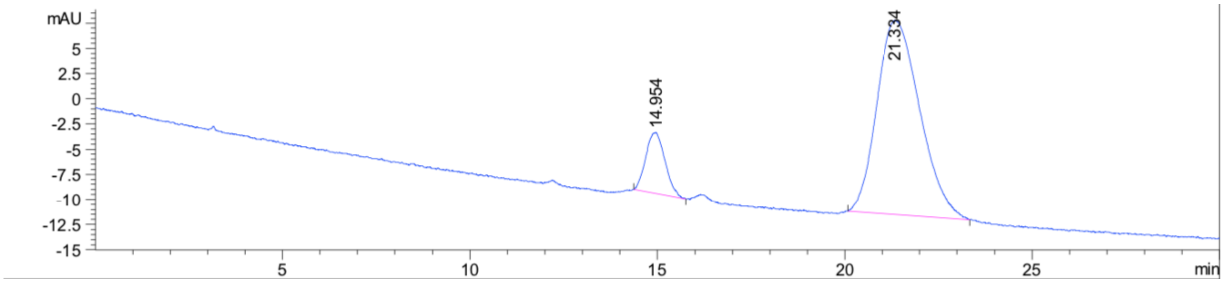

Peak #	Ret Time [min]	Type	Width [min]	Area [mAU*s]	Height [mAU]	Area %
1	14.954	BB	0.4303	215.12451	6.02916	12.2580
2	21.334	BB	0.9431	1539.85327	19.29395	87.7420

Totals : 1754.97778 25.32312

tert-Butyl 1'-(3,5-bis(trifluoromethyl)benzyl)-2'-oxospiro[azetidine-2,3'-indoline]-1-carboxylate (19)

Conditions: Chiralpak IC column, 98.5:1.5 _n_-hexane:_i_-PrOH, flow rate: 1 mL min^{-1}, 35 ºC, UV detection wavelength: 254 nm

(±)–19

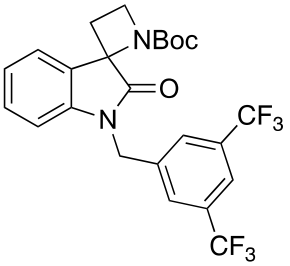

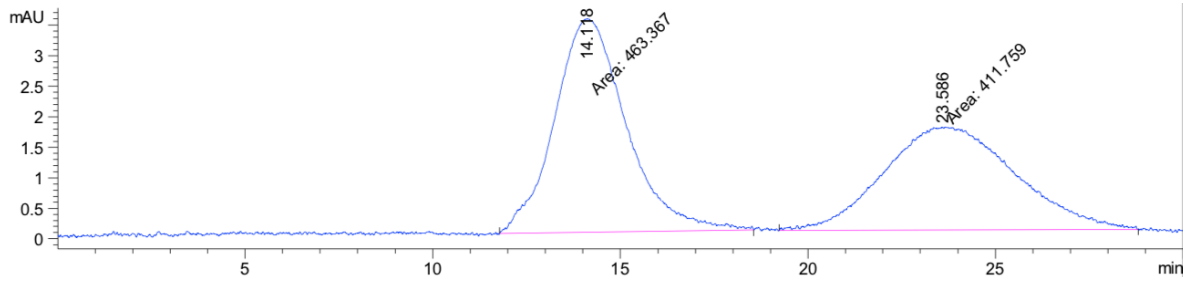

Peak #	Ret Time [min]	Type	Width [min]	Area [mAU*s]	Height [mAU]	Area %
1	14.118	PP	2.2116	463.36749	3.49198	52.9486
2	23.586	PM	4.0581	411.75876	1.69109	47.0514
Totals :				875.12625	5.18307	

(–)–(_S_)–19

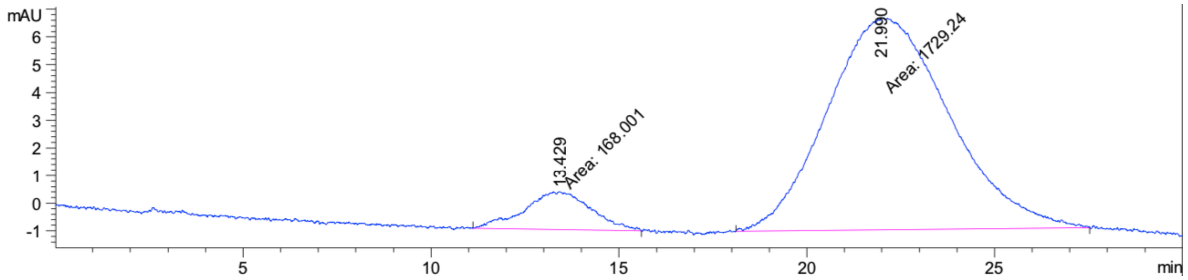

Peak #	Ret Time [min]	Type	Width [min]	Area [mAU*s]	Height [mAU]	Area %
1	13.429	MP	2.0608	168.00090	1.35869	8.8550
2	21.990	PP	3.7638	1729.23853	7.65734	91.1450
Totals :				1897.23943	9.01602	

tert-Butyl 1'-(naphthalen-2-ylmethyl)-2'-oxospiro[azetidine-2,3'-indoline]-1-carboxylate (20)

Conditions: Chiralpak ID column, 90:10 *n*-hexane:*i*-PrOH, flow rate: 1 mL min^{-1}, 35 ºC, UV detection wavelength: 254 nm

(±)–20

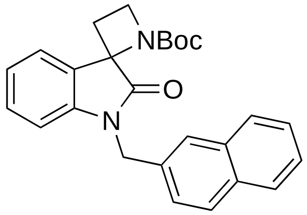

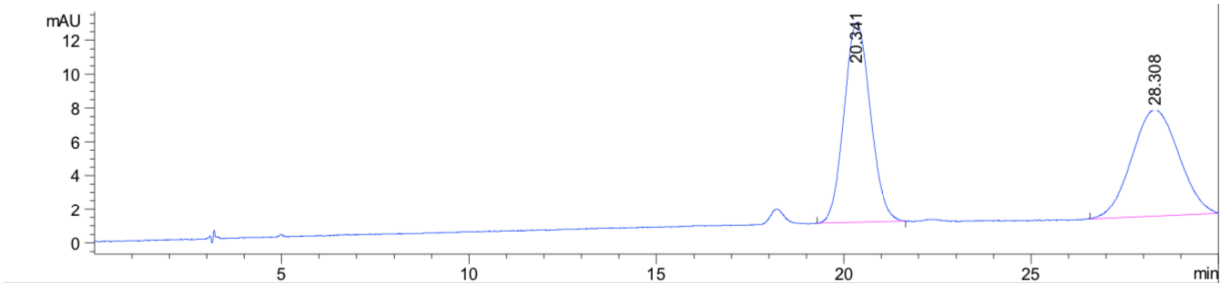

Peak #	Ret Time [min]	Type	Width [min]	Area [mAU*s]	Height [mAU]	Area %
1	20.341	BB	0.6875	545.33740	11.09324	51.2982
2	28.307	BBA	1.0254	517.73627	5.92523	48.7018
Totals :				1063.07367	17.01847	

(–)–(*S*)–20

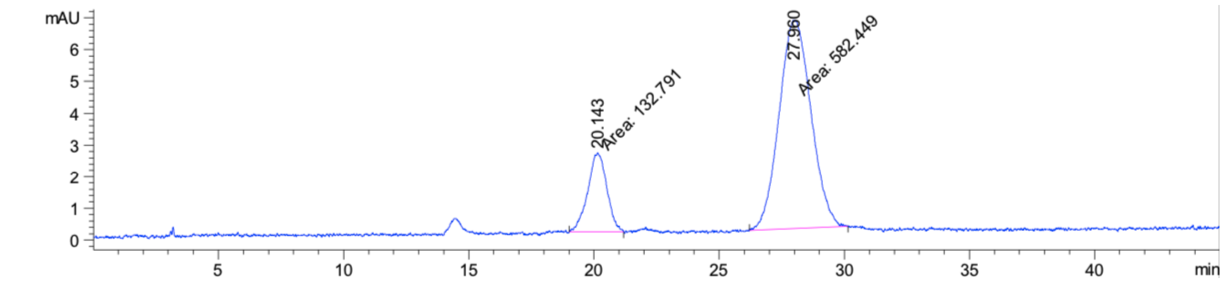

Peak #	Ret Time [min]	Type	Width [min]	Area [mAU*s]	Height [mAU]	Area %
1	20.143	PP	0.8829	132.79059	2.50682	18.5659
2	27.960	PP	1.4783	582.44928	6.56684	81.4341
Totals :				715.23987	9.07366	

Benzyl 1'-benzyl-2'-oxospiro[azetidine-2,3'-indoline]-1-carboxylate (21)

Conditions: Chiralpak IA column, 80:20 *n*-hexane:*i*-PrOH, flow rate: 1 mL min^{-1}, 35 ºC, UV detection wavelength: 254 nm

(±)–21

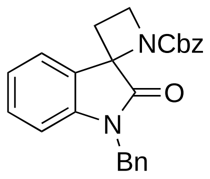

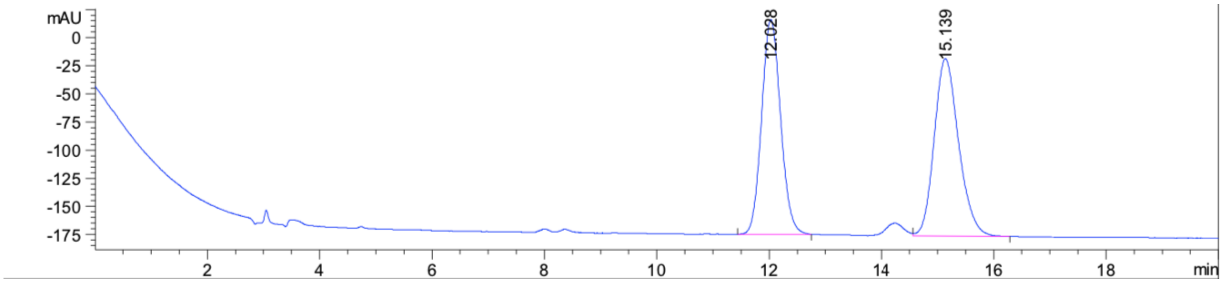

Peak #	Ret Time [min]	Type	Width [min]	Area [mAU*s]	Height [mAU]	Area %
1	12.028	BB	0.3675	4479.76172	191.11395	48.3737
2	15.139	VB	0.4574	4780.98584	157.85286	51.6263
Totals :				9260.74756	348.96681	

(–)–(*S*)–21

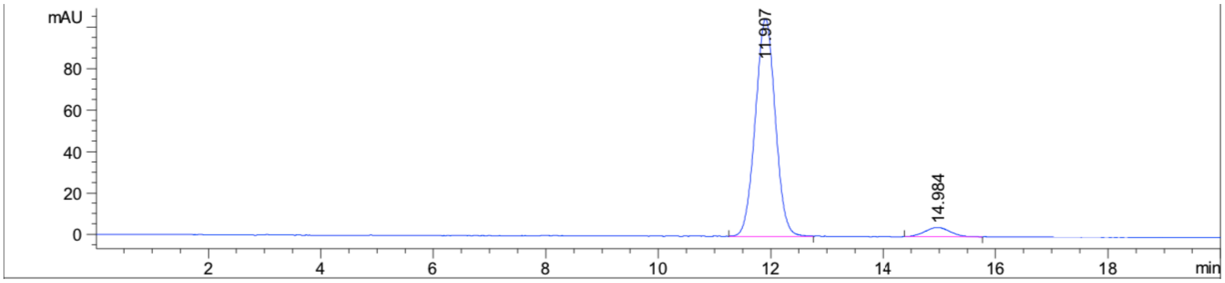

Peak #	Ret Time [min]	Type	Width [min]	Area [mAU*s]	Height [mAU]	Area %
1	11.907	BB	0.3812	2581.89502	104.88249	94.8984
2	14.984	BB	0.3936	138.79991	4.42879	5.1016
Totals :				2720.69493	109.31128	

Methyl 1'-benzyl-2'-oxospiro[azetidine-2,3'-indoline]-1-carboxylate (22)

Conditions: Chiralpak IA column, 80:20 *n*-hexane:*i*-PrOH, flow rate: 1 mL min^{-1}, 35 ºC, UV detection wavelength: 254 nm

(±)–22

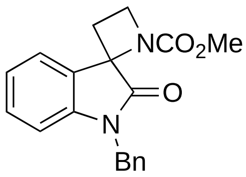

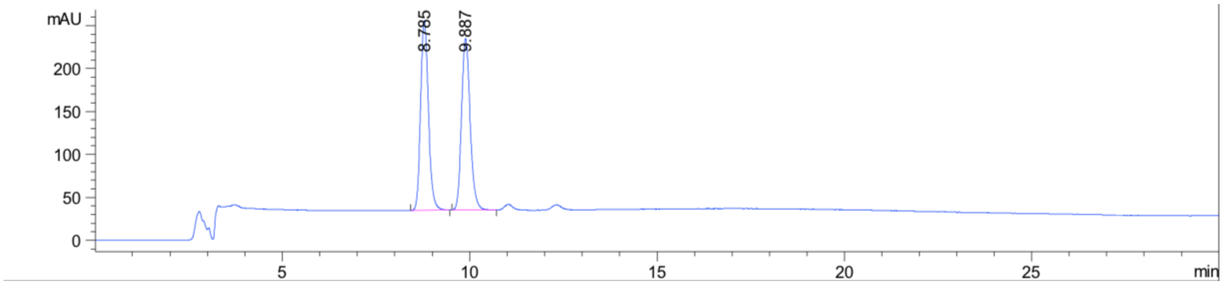

Peak #	RetTime [min]	Type	Width [min]	Area [mAU*s]	Height [mAU]	Area %
1	8.785	BB	0.2223	3190.69800	221.16893	50.0000
2	9.887	BB	0.2462	3190.69702	199.92406	50.0000
Totals :				6381.39502	421.09299	

(–)–(*S*)–22

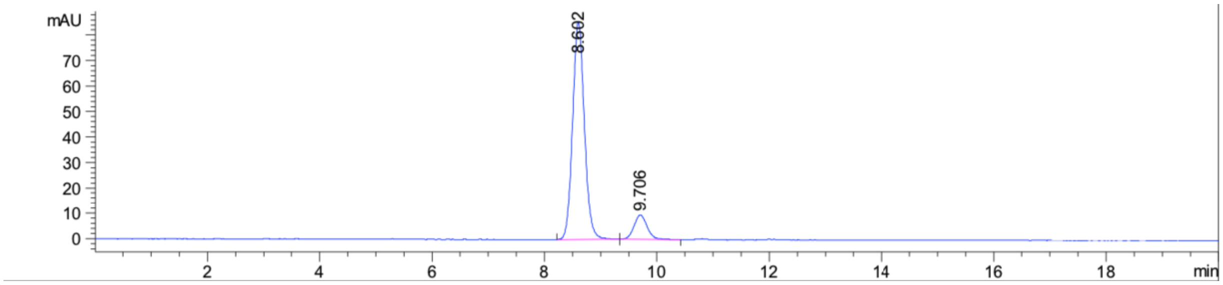

Peak #	RetTime [min]	Type	Width [min]	Area [mAU*s]	Height [mAU]	Area %
1	8.602	BB	0.2240	1245.56458	85.49292	88.6550
2	9.706	BB	0.2507	159.39255	9.65243	11.3450
Totals :				1404.95712	95.14535	

tert-Butyl 1'-benzyl-5',7'-dimethyl-2'-oxospiro[azetidine-2,3'-indoline]-1-carboxylate (23)

Conditions: Chiralpak IE column, 90:10 *n*-hexane:*i*-PrOH, flow rate: 1 mL min^{-1}, 35 ºC, UV detection wavelength: 254 nm

(±)–23

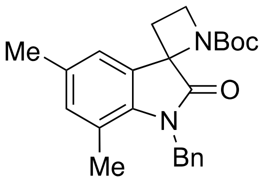

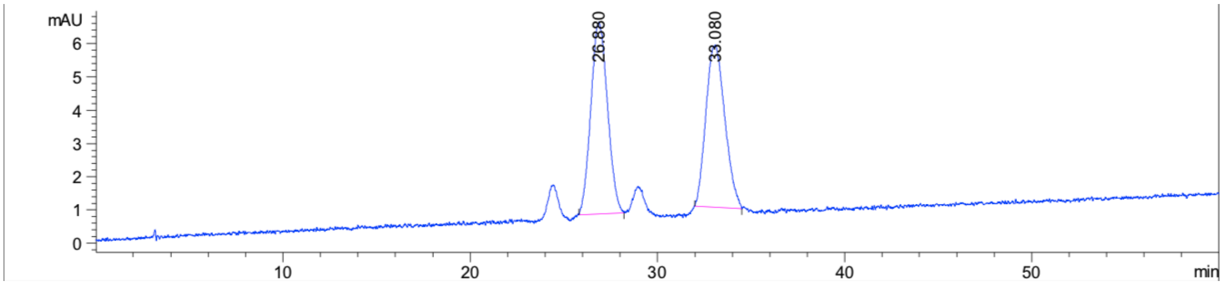

Peak #	Ret Time [min]	Type	Width [min]	Area [mAU*s]	Height [mAU]	Area %
1	26.880	BB	0.7374	357.44885	5.78069	51.0496
2	33.080	BB	0.8312	342.75040	4.88152	48.9504
Totals :				700.19925	10.66221	

(–)–(*S*)–23

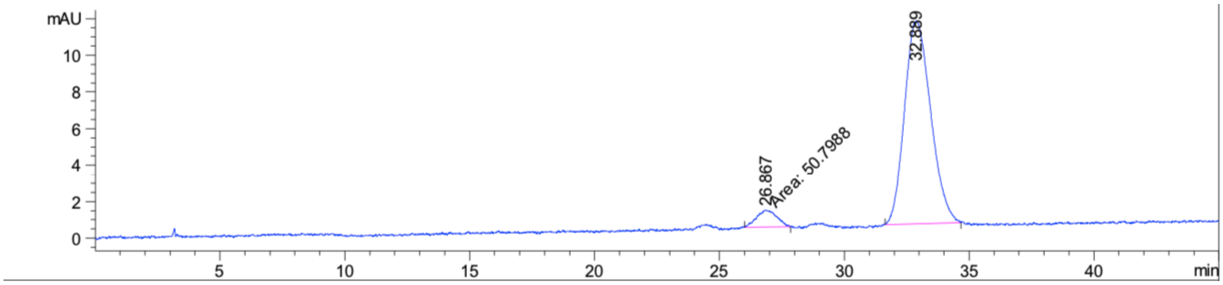

Peak #	Ret Time [min]	Type	Width [min]	Area [mAU*s]	Height [mAU]	Area %
1	26.867	PP	0.9263	50.79882	9.14001e-1	5.9034
2	32.889	BB	0.8649	809.70593	11.10130	94.0966
Totals :				860.50476	12.01530	

***tert*-Butyl 1'-benzyl-4',6'-difluoro-2'-oxospiro[azetidine-2,3'-indoline]-1-carboxylate (24)**

Conditions: Chiralpak ID column, 90:10 *n*-hexane:*i*-PrOH, flow rate: 1 mL min^{-1}, 35 ºC, UV detection wavelength: 254 nm

(±)–24

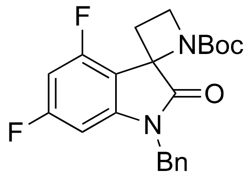

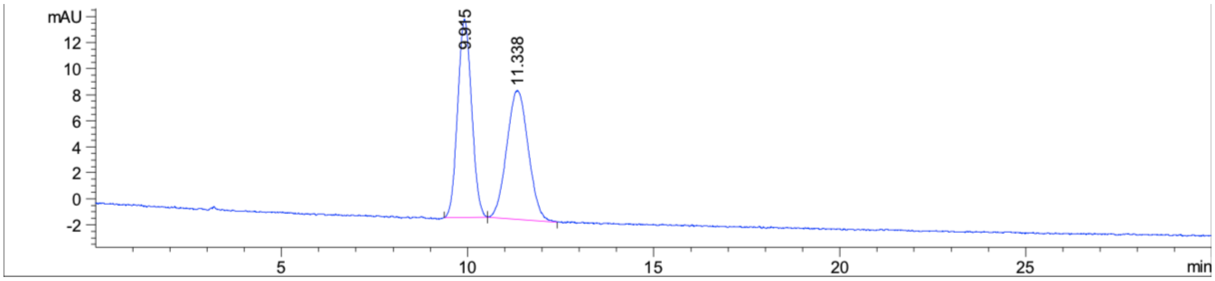

Peak #	Ret Time [min]	Type	Width [min]	Area [mAU*s]	Height [mAU]	Area %
1	9.915	BB	0.4001	397.16812	15.23955	50.0326
2	11.338	BB	0.4841	396.65027	9.88595	49.9674
Totals :				793.81839	25.12550	

(–)–(*S*)–24

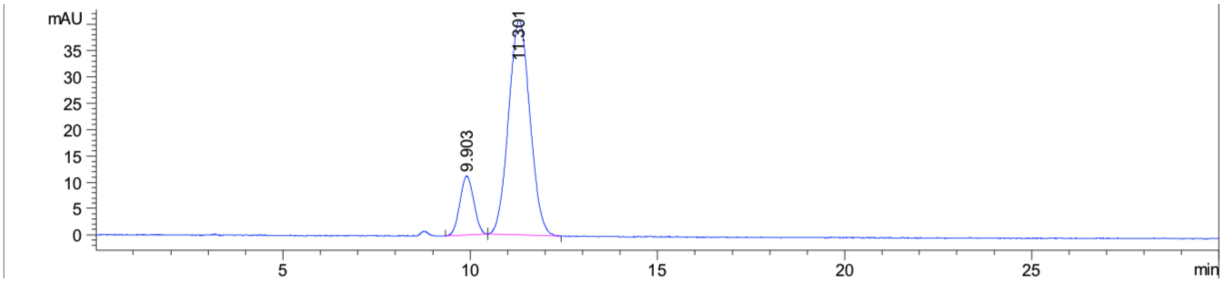

Peak #	Ret Time [min]	Type	Width [min]	Area [mAU*s]	Height [mAU]	Area %
1	9.903	BB	0.3777	289.13007	11.25432	15.1140
2	11.301	BB	0.5982	1623.87109	40.81146	84.8860
Totals :				1913.00116	52.06577	

tert-Butyl 2'-oxospiro[azetidine-2,3'-indoline]-1-carboxylate (25)

Conditions: Chiralpak IE column, 90:10 _n_-hexane:_i_-PrOH, flow rate: 1 mL min^{-1}, 35 ºC, UV detection wavelength: 254 nm

(±)–25

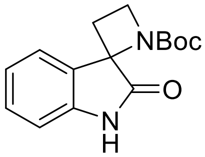

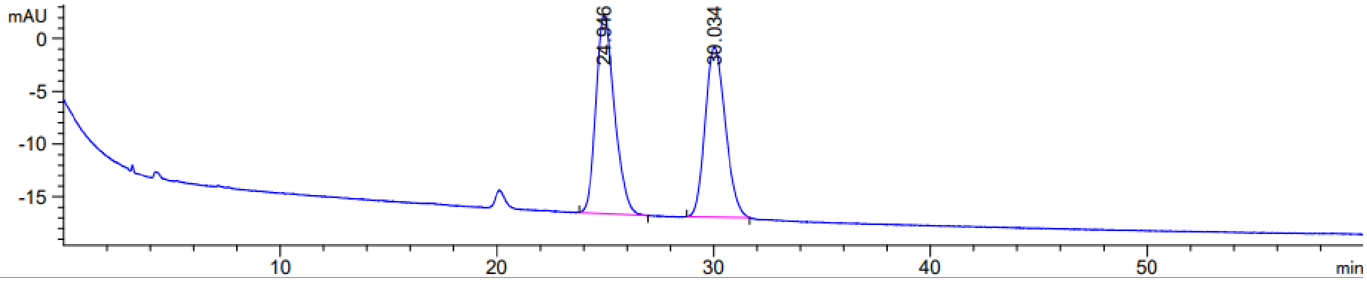

Peak #	RetTime [min]	Type	Width [min]	Area [mAU*s]	Height [mAU]	Area %
1	24.946	BB	0.7488	1112.07813	18.78098	51.6347
2	30.034	BB	0.7869	1041.66382	16.14024	48.3653
Totals :				2153.74194	34.92122	

(–)–(_S_)–25

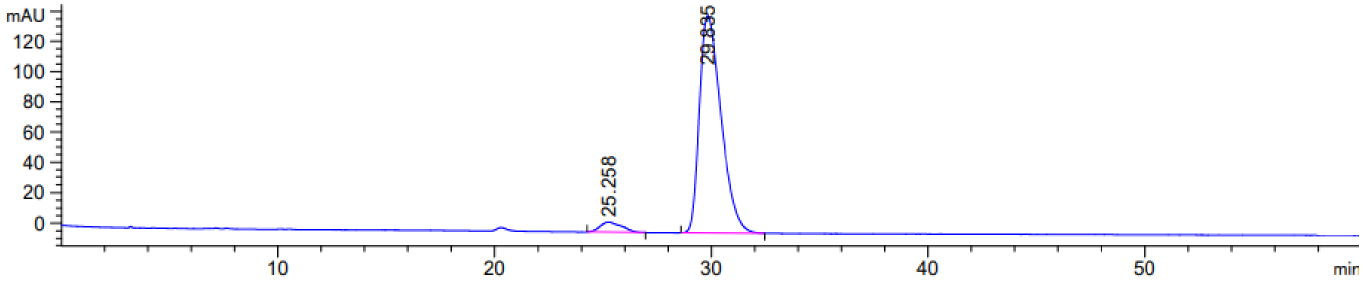

Peak #	RetTime [min]	Type	Width [min]	Area [mAU*s]	Height [mAU]	Area %
1	25.258	BB	0.8107	451.92432	6.60151	4.3667
2	29.835	BB	1.0383	9897.31836	143.57204	95.6333
Totals :				1.03492e4	150.17355	

1'-Benzylspiro[azetidine-2,3'-indolin]-2'-one (26)

Conditions: Chiralpak IE column, 90:10 *n*-hexane:*i*-PrOH, flow rate: 1 mL min^{-1}, 35 ºC, UV detection wavelength: 254 nm

(±)–26

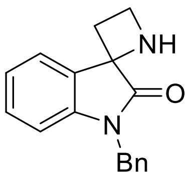

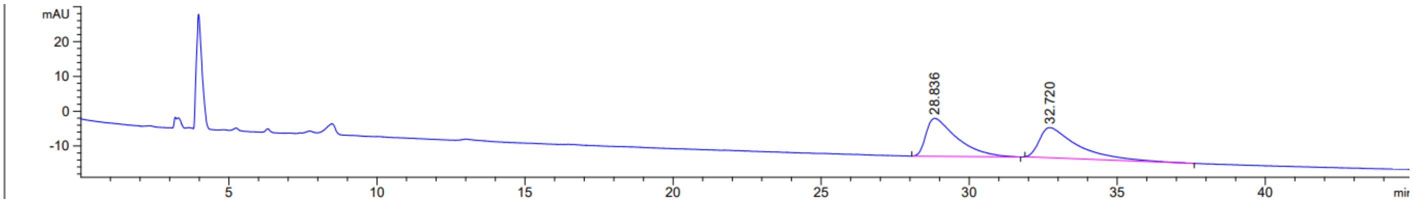

Peak #	RetTime [min]	Type	Width [min]	Area [mAU*s]	Height [mAU]	Area %
1	28.836	BB	0.8828	789.51941	10.90375	50.1963
2	32.720	BB	1.0679	783.34332	8.67170	49.8037
Totals :				1572.86273	19.57546	

(–)–(*S*)–26

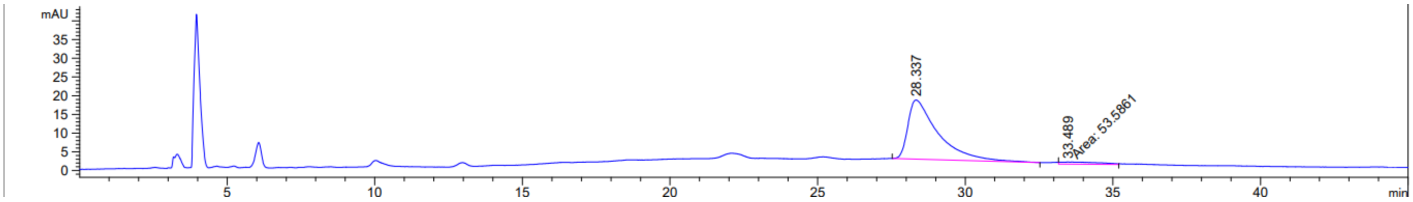

Peak #	RetTime [min]	Type	Width [min]	Area [mAU*s]	Height [mAU]	Area %
1	28.337	BB	0.9234	1117.16504	15.79121	95.4229
2	33.489	MM	1.6151	53.58606	5.52958e-1	4.5771
Totals :				1170.75110	16.34416	

1-Benzylspiro[indoline-3,6'-[1,3]oxazinane]-2,2'-dione (27)

Conditions: Chiralpak IF column, 80:20 *n*-hexane:*i*-PrOH, flow rate: 1 mL min^{-1}, 35 ºC, UV detection wavelength: 254 nm

(±)–27

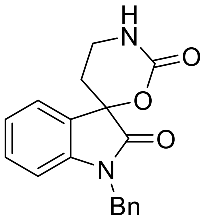

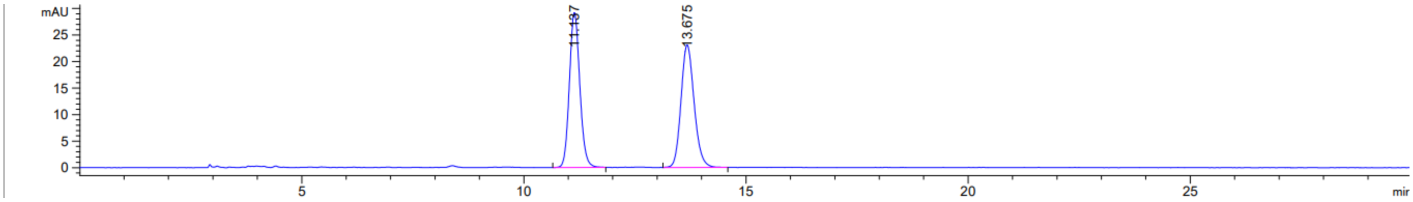

Peak #	RetTime [min]	Type	Width [min]	Area [mAU*s]	Height [mAU]	Area %
1	11.137	BB	0.2498	474.83978	29.19341	50.0278
2	13.675	BB	0.3136	474.31161	23.09693	49.9722
Totals :				949.15140	52.29035	

(–)–(*S*)–27

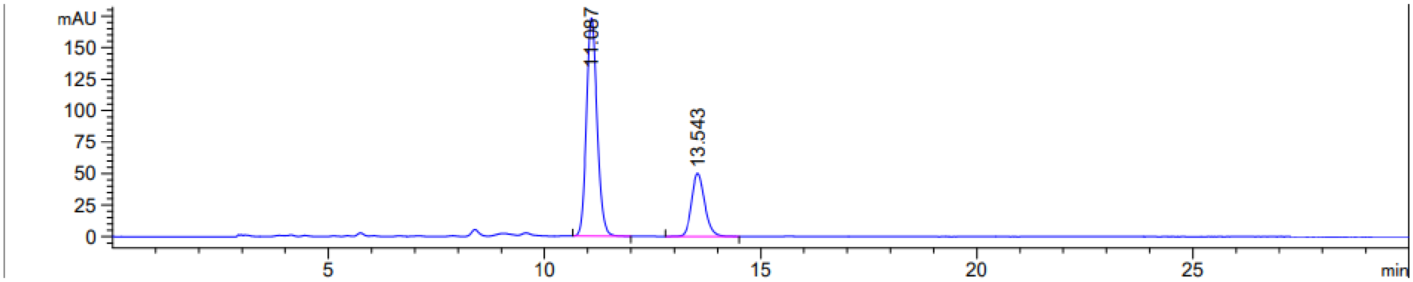

Peak #	RetTime [min]	Type	Width [min]	Area [mAU*s]	Height [mAU]	Area %
1	11.087	BB	0.2604	2912.89502	173.07283	73.5387
2	13.543	BB	0.3225	1048.14050	50.01987	26.4613
Totals :				3961.03552	223.09270	

tert-Butyl 1'-benzyl-2'-oxo-6'-(m-tolyl)spiro[azetidine-2,3'-indoline]-1-carboxylate (28)

Conditions: Chiralpak ID column, 90:10 *n*-hexane:*i*-PrOH, flow rate: 1 mL min^{-1}, 35 ºC, UV detection wavelength: 254 nm

(±)–28

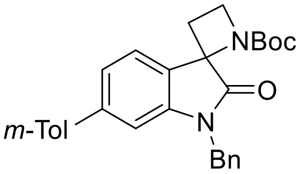

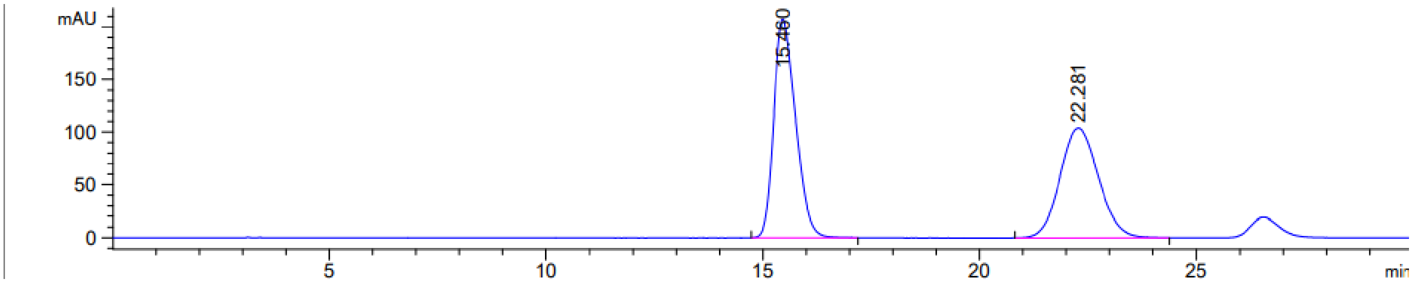

Peak #	RetTime [min]	Type	Width [min]	Area [mAU*s]	Height [mAU]	Area %
1	15.460	BB	0.5268	7245.66260	207.77954	53.1884
2	22.281	BB	0.9258	6376.98291	103.65095	46.8116

Totals : 1.36226e4 311.43049

(–)–(*S*)–28

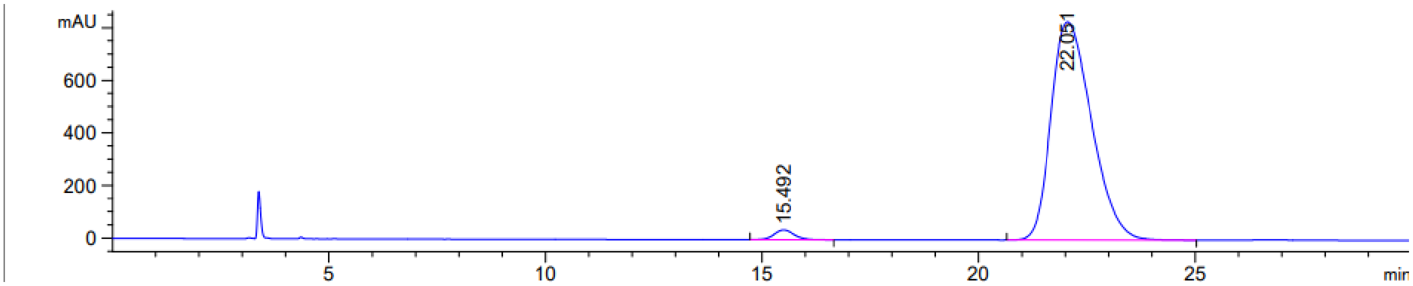

Peak #	RetTime [min]	Type	Width [min]	Area [mAU*s]	Height [mAU]	Area %
1	15.492	BB	0.4938	1177.29590	36.94494	2.1360
2	22.051	BB	0.9657	5.39392e4	830.47626	97.8640

Totals : 5.51165e4 867.42120

tert-Butyl 1-benzyl-2-oxospiro[indoline-3,2'-pyrrolidine]-1'-carboxylate (30)

Conditions: Chiralpak ID column, 80:20 *n*-hexane:*i*-PrOH, flow rate: 1 mL min^{-1}, 35 ºC, UV detection wavelength: 254 nm

(±)–30

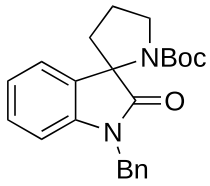

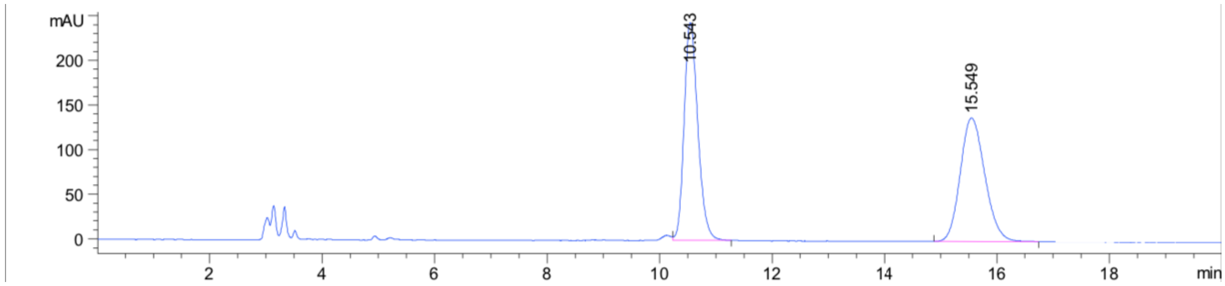

Peak #	Ret Time [min]	Type	Width [min]	Area [mAU*s]	Height [mAU]	Area %
1	10.543	VV	0.2634	4129.20264	244.09178	49.4999
2	15.549	BB	0.4605	4212.64063	138.63885	50.5001
Totals :				8341.84326	382.73064	

(–)–(*S*)–30

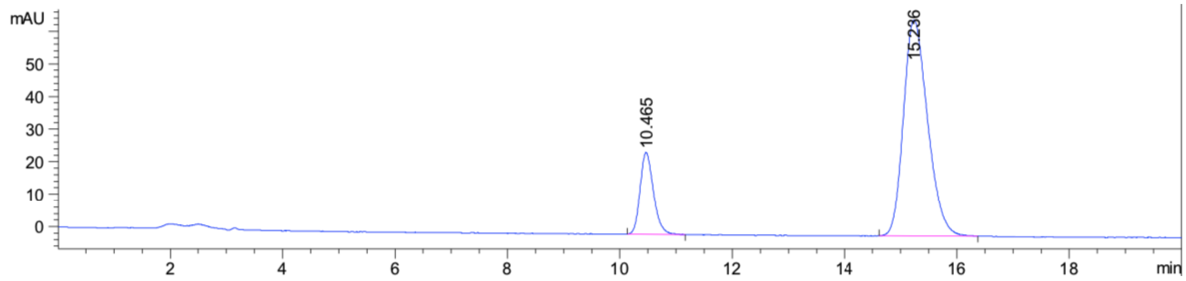

Peak #	Ret Time [min]	Type	Width [min]	Area [mAU*s]	Height [mAU]	Area %
1	10.465	BB	0.2553	416.95822	25.16654	17.3319
2	15.236	BB	0.4576	1988.76379	66.36789	82.6681
Totals :				2405.72202	91.53443	

X-Ray Discussion

Supporting Information — X-Ray Crystallography

Table S1. Crystal Data, Data Collection and Refinement Parameters for the structures of **Cat7**, **2a**, **3**, **4**, **8**, **11**, **12**, **24** and **30**.

data	Cat7	2a	3	4
formula	$[C_{26}H_{28}F_5N_2OS]Br$	$C_{22}H_{25}ClN_2O_3$	$C_{22}H_{24}N_2O_3$	$C_{23}H_{26}N_2O_4$
solvent	$0.5(H_2O)$	—	—	—
formula weight	600.48	400.89	364.43	394.46
colour, habit	colourless plates	colourless blocks	colourless blocks	colourless hexagonal tablets
temperature / K	173	173	173	173
crystal system	monoclinic	monoclinic	orthorhombic	monoclinic
space group	$P2_1$ (no. 4)	$P2_1/c$ (no. 14)	$P2_12_12_1$ (no. 19)	$P2_1/c$ (no. 14)
a / Å	11.56618(15)	9.4777(4)	12.9504(3)	13.9967(7)
b / Å	8.95899(14)	9.8446(4)	15.1729(4)	7.7675(3)
c / Å	25.7749(4)	22.6335(9)	20.2983(5)	18.8888(7)
α / deg	90	90	90	90
β / deg	97.8264(14)	95.696(4)	90	95.354(4)
γ / deg	90	90	90	90
V / Å^3	2645.95(7)	2101.37(14)	3988.51(18)	2044.62(16)
Z	4 [c]	4	8 [c]	4
D_c / g cm^{-3}	1.507	1.267	1.214	1.281
radiation used	Cu-Kα	Mo-Kα	Cu-Kα	Mo-Kα
μ / mm^{-1}	3.389	0.206	0.652	0.088
no. of unique reflns				
measured (R_{int})	10288 (0.0648)	4203 (0.0218)	7615 (0.0590)	4112 (0.0360)
obs, $\|F_o\| > 4\sigma(\|F_o\|)$	9057	2959	5585	3010
completeness (%) [a]	100	98.8	99.1	98.9
no. of variables	666	271	494	267
R_1(obs), wR_2(all) [b]	0.0375, 0.0889	0.0455, 0.1102	0.0533, 0.1445	0.0502, 0.1174
CCDC code	2271852	2271853	2271854	2271855

[a] Completeness to 0.84 Å resolution. [b] $R_1 = \Sigma||F_o| - |F_c||/\Sigma|F_o|$; $wR_2 = \{\Sigma[w(F_o^2 - F_c^2)^2] / \Sigma[w(F_o^2)^2]\}^{1/2}$; $w^{-1} = \sigma^2(F_o^2) + (aP)^2 + bP$.
[c] There are two crystallographically independent molecules.

Table S1. …part 2

data	8	11	12	24
**formula**	$C_{22}H_{23}FN_2O_3$	$C_{22}H_{23}BrN_2O_3$	$C_{22}H_{23}BrN_2O_3$	$C_{22}H_{22}FN_2O_3$
**solvent**	—	—	—	—
**formula weight**	382.42	443.33	443.33	400.41
**colour, habit**	colourless [illegible]	colourless [illegible]	colourless blocks	colourless blocks
**temperature / K**	173	173	173	173
**crystal system**	triclinic	orthorhombic	monoclinic	orthorhombic
**space group**	P-1 (no. 2)	$P2_12_12_1$ (no. 19)	$P2_1$ (no. 4)	$P2_12_12_1$ (no. 19)
a **/ Å**	9.0794(11)	9.3712(3)	8.8565(3)	9.3708(2)
b **/ Å**	10.1910(11)	13.2604(3)	11.1338(3)	12.1170(3)
c **/ Å**	10.6587(13)	16.8619(5)	10.0946(3)	18.0973(4)
**α / deg**	107.981(10)	90	90	90
**β / deg**	93.178(10)	90	90.987(3)	90
**γ / deg**	95.593(9)	90	90	90
V **/** $Å^3$	929.78(19)	2095.35(10)	995.25(5)	2054.87(9)
Z	2	4	2	4
D_c **/ g** cm^{-3}	1.366	1.405	1.479	1.294
**radiation used**	Mo-Kα	Cu-Kα	Cu-Kα	Cu-Kα
**μ /** mm^{-1}	0.098	2.873	3.025	0.827
**no. of unique reflns**				
**measured (**R_{int}**)**	3664 (0.0296)	3993 (0.0489)	3779 (0.0381)	4118 (0.0631)
**obs,** $\|F_o\| > 4\sigma(\|F_o\|)$	2675	3433	3453	3405
**completeness (%)** [a]	98.6	98.9	99.5	100
**no. of variables**	257	254	257	266
R_1**(obs),** wR_2**(all)** [b]	0.0539, 0.1408	0.0448, 0.1182	0.0424, 0.1162	0.0582, 0.1643
**CCDC code**	2271856	2271857	2271858	2271859

Table S1. …part 3

data	30
**formula**	$C_{23}H_{26}N_2O_3$
**solvent**	—
**formula weight**	378.46
**colour, habit**	colourless
**temperature / K**	173
**crystal system**	monoclinic
**space group**	$P2_1$ (no. 4)
a **/ Å**	8.7487(9)
b **/ Å**	13.4090(11)
c **/ Å**	9.3797(6)
**α / deg**	90
**β / deg**	110.630(10)
**γ / deg**	90
V **/ Å³**	1029.78(16)
Z	2
D_c **/ g cm⁻³**	1.221
**radiation used**	Cu-Kα
**μ / mm⁻¹**	0.649
**no. of unique reflns**	
**measured** (R_{int})	2654 (0.0601)
**obs,** $\|F_o\| > 4\sigma(\|F_o\|)$	1878
**completeness (%)** [a]	97.8
**no. of variables**	257
R_1**(obs),** wR_2**(all)** [b]	0.0506, 0.1208
**CCDC code**	2271860

Table S1 provides a summary of the crystallographic data for the structures of of **Cat7**, **2a**, **3**, **4**, **8**, **11**, **12**, **24** and **30**. Data were collected using Agilent Xcalibur PX Ultra A (**Cat7**, **3**, **11**, **12**, **24** and **30**) and Xcalibur 3 E (**2a**, **4** and **8**) diffractometers, and the structures were solved and refined using the OLEX2,[X1] SHELXTL[X2] and SHELX-2013[X3] program systems. Whilst the absolute structures of **Cat7**, **11** and **12** could be determined by use of the Flack parameter [*x* = –0.057(8), –0.040(2) and 0.00(3) respectively], those of **3**, **24** and **30** could not [Flack parameter *x* = 0.1(2), –0.21(10) and 0.1(5) respectively]. CCDC 2271852 to 2271860.

X-ray crystallography

The structure of **Cat7** was found to contain two crystallographically independent molecules (**Cat7-A** and **Cat7-B**) in the asymmetric unit. The O23–H hydrogen atoms of both independent molecules were located from Δ*F* maps and refined freely subject to an O–H distance constraint of 0.90 Å. The hydrogen atoms of the O40-based presumed water molecule could not be located from Δ*F* maps and so were omitted, and as a result the atom list for the asymmetric unit is low by 2H (and that for the unit cell low by 4H) compared to what is actually presumed to be present. The absolute structure of **Cat7** was unambiguously determined by use of the Flack parameter [*x* = –0.057(8)]. The O19-bound *t*-butyl group in the structure of **2a** was found to be disordered. Two orientations were identified of *ca*. 87 and 13% occupancy, their geometries were optimised, the thermal parameters of adjacent atoms were restrained to be similar, and only the non-hydrogen atoms of the major occupancy orientation were refined anisotropically (those of the minor occupancy orientation were refined isotropically). The structure of **3** was found to contain two crystallographically independent molecules (**3-A** and **3-B**) in the asymmetric unit. The absolute structure of **3** could not be determined [Flack parameter *x* = 0.1(2)]. The absolute structure of **11** was unambiguously determined by use of the Flack parameter [*x* = –

0.04(2)]. The absolute structure of **12** was unambiguously determined by use of the Flack parameter [*x* = 0.00(3)]. The absolute structure of **24** could not be determined [Flack parameter *x* = –0.21(10)]. The absolute structure of **30** could not be determined [Flack parameter *x* = 0.1(5)].

[X1] O.V. Dolomanov, L.J. Bourhis, R.J. Gildea, J.A.K. Howard, H. Puschmann, *J. Appl. Cryst.*, 2009, **42**, 339-341.
[X2] SHELXTL v5.1, Bruker AXS, Madison, WI, 1998.
[X3] SHELX-2013, G.M. Sheldrick, *Acta Cryst.*, 2015, **C71**, 3-8.

Figures

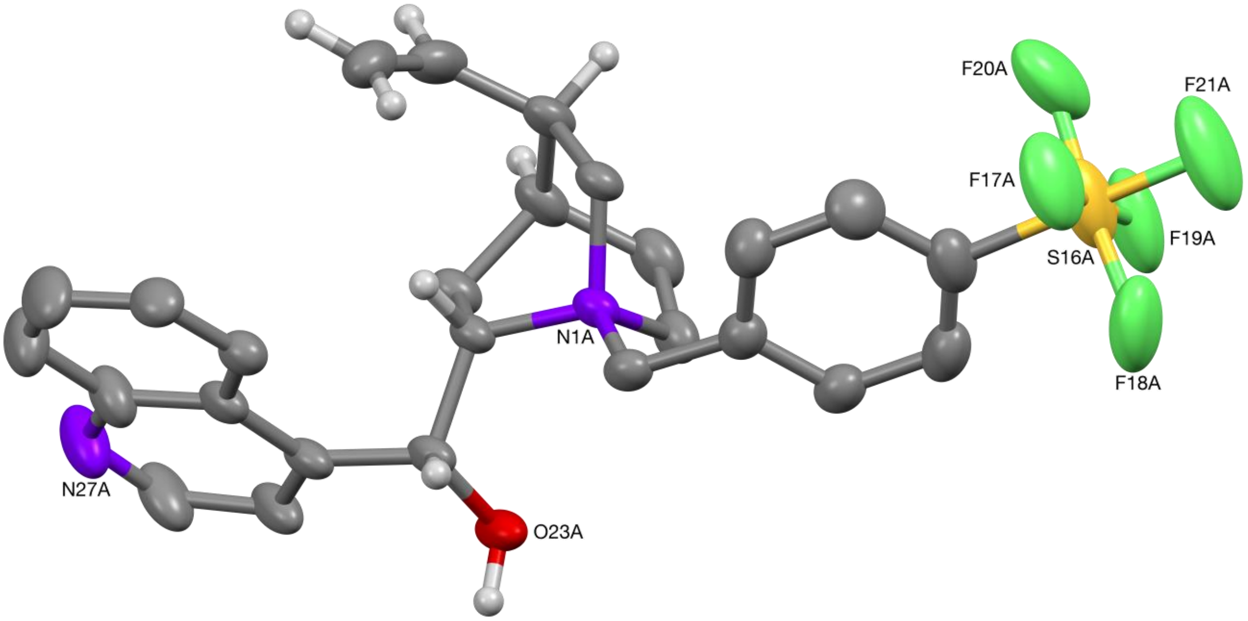

Fig. S1 The structure of cation **Cat7-A**, one of the two independent cations present in the crystal of **Cat7** (50% probability ellipsoids).

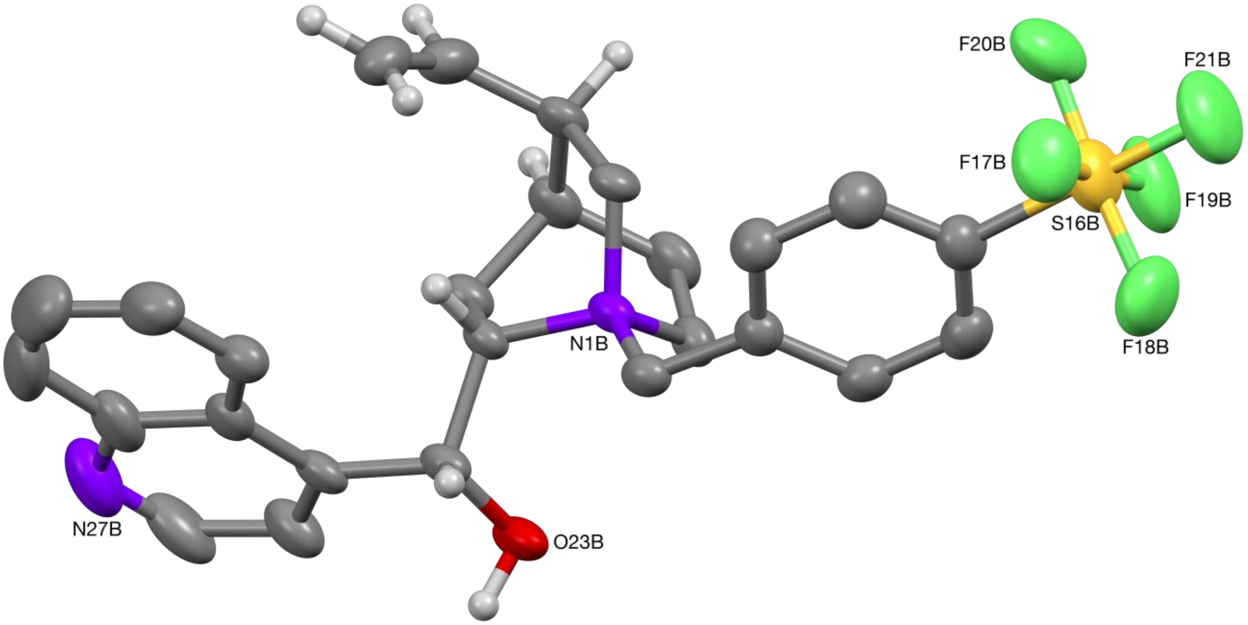

Fig. S2 The structure of cation **Cat7-B**, one of the two independent cations present in the crystal of **Cat7** (50% probability ellipsoids).

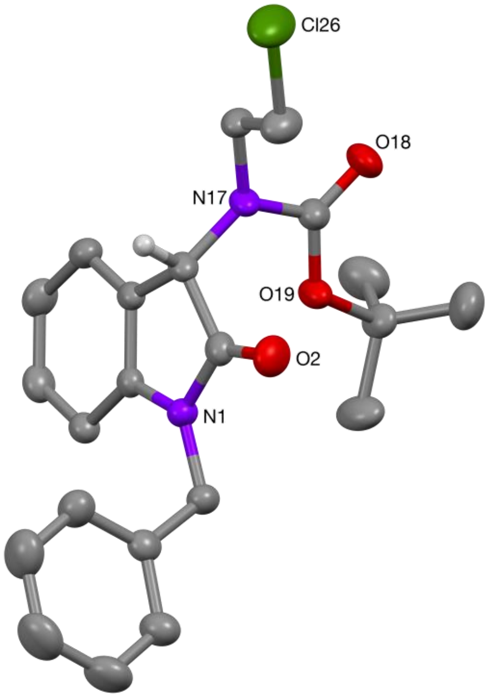

Fig. S3 The crystal structure of **2a** (50% probability ellipsoids).

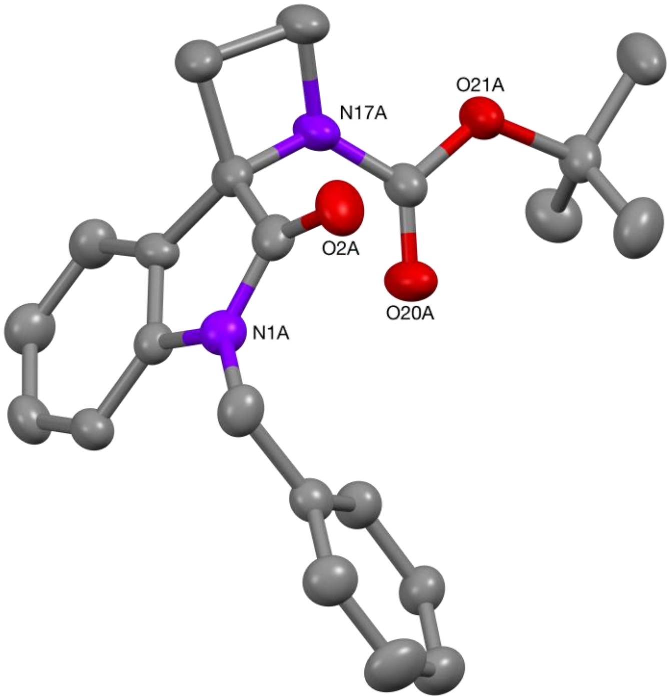

Fig. S4 The structure of **3-A**, one of the two independent molecules present in the crystal of **3** (50% probability ellipsoids).

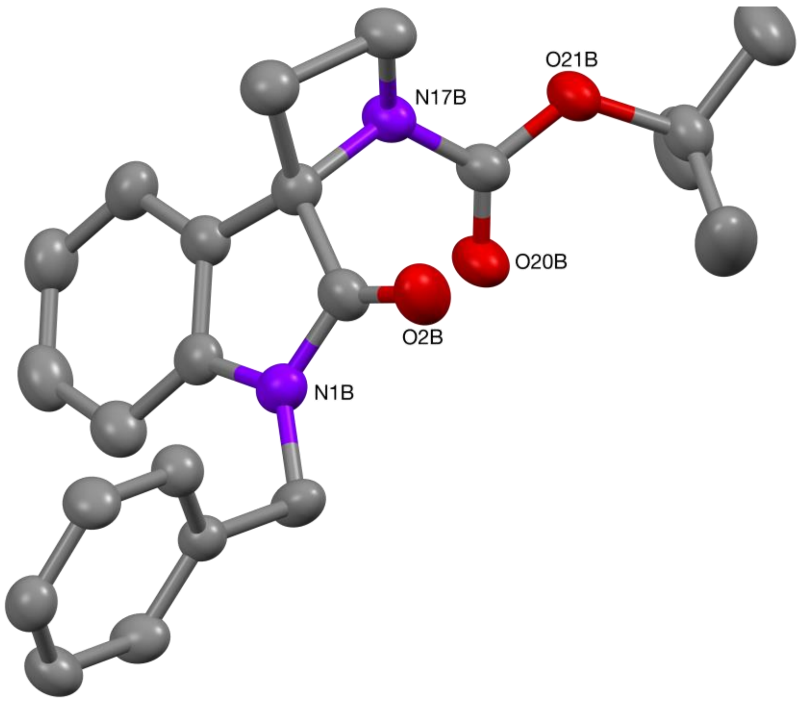

Fig. S5 The structure of **3-B**, one of the two independent molecules present in the crystal of **3** (50% probability ellipsoids).

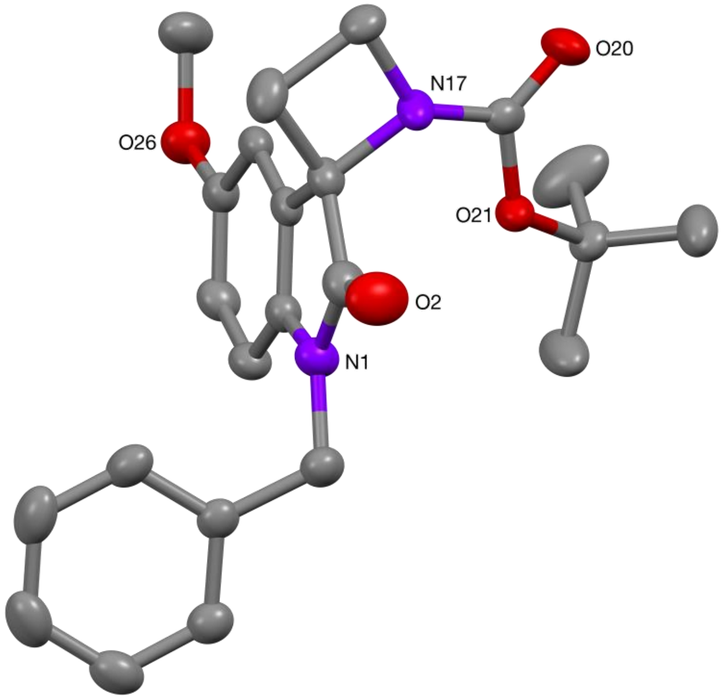

Fig. S6 The crystal structure of **4** (50% probability ellipsoids).

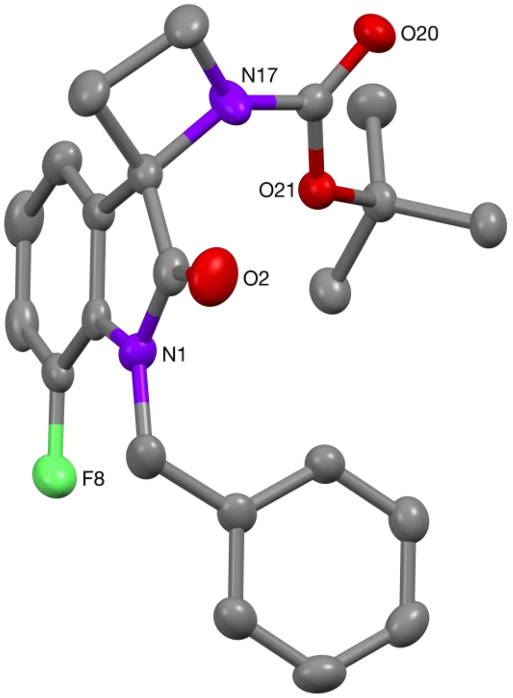

Fig. S7 The crystal structure of **8** (50% probability ellipsoids).

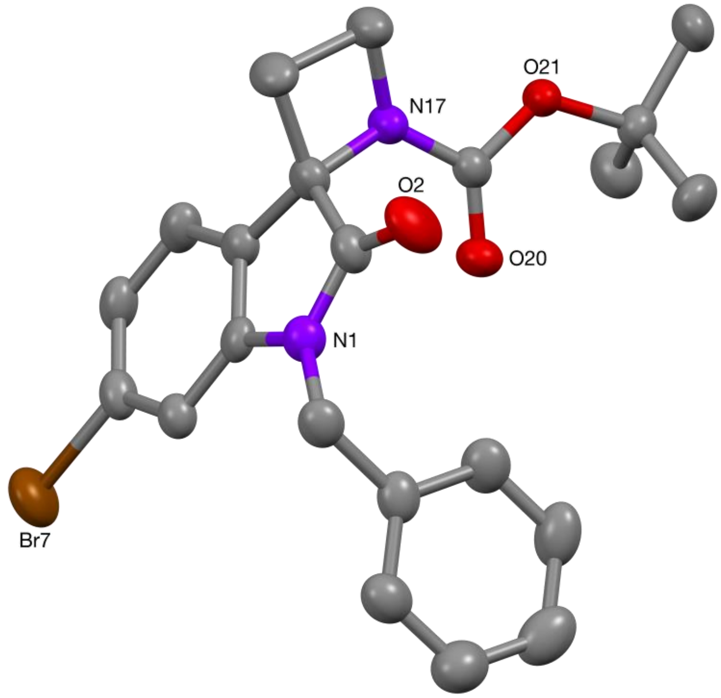

Fig. S8 The crystal structure of **11** (50% probability ellipsoids).

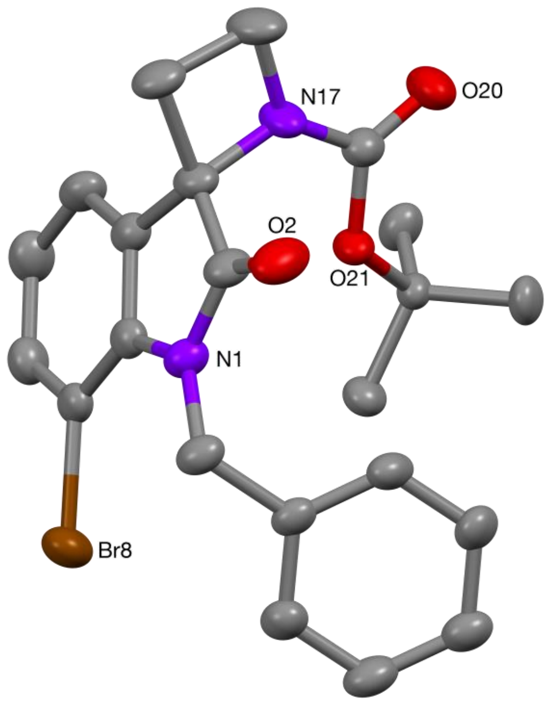

Fig. S9 The crystal structure of **12** (50% probability ellipsoids).

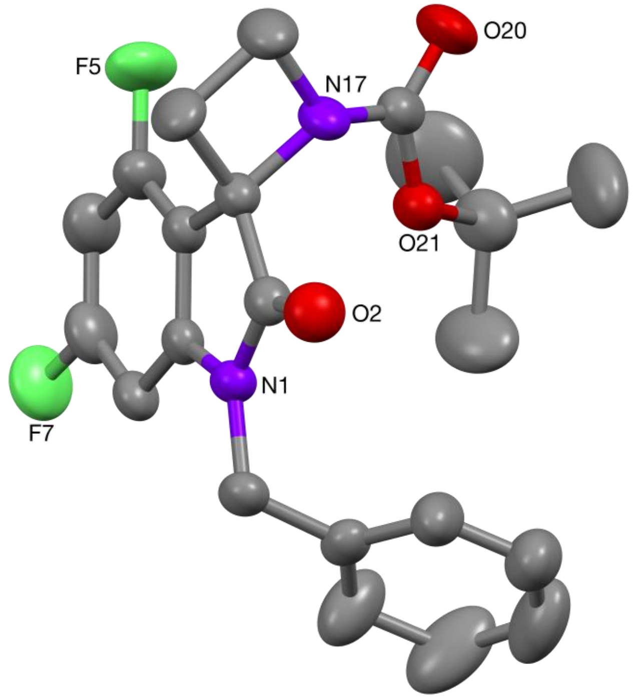

Fig. S10 The crystal structure of **24** (50% probability ellipsoids).

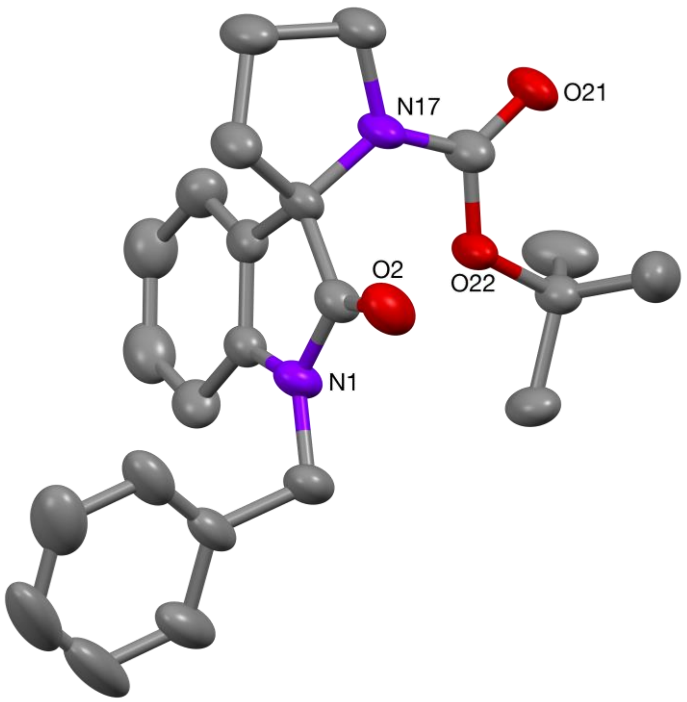

Fig. S11 The crystal structure of **30** (50% probability ellipsoids).

References

1) S. P. Green, K. M. Wheelhouse, A. D. Payne, J. P. Hallett, P. W. Miller, J. A. Bull, *Org. Process Res. Dev.* **2020**, *24*, 67–84.

2) K. M. Johnson, M. S. Rattley, F. Sladojevich, D. M. Barber, M. G. Nuñez, A. M. Goldys, D. J. Dixon, *Org. Lett.* **2012**, *14*, 2492–2495.

3) Y. Wang, H. Yin, X. Tang, Y. Wu, Q. Meng, Z. Gao, *J. Org. Chem.* **2016**, *81*, 7042–7050.

4) a) L. Pitzer, F. Schäfers, F. Glorius, *Angew. Chem. Int. Ed.* **2019**, *58*, 8572–8576. b) S. Cembellín, T. Dalton, T. Pinkert, F. Schäfers, F. Glorius, *ACS Catal.* **2020**, *10*, 197–202.

5) An assessment of some parameters for the sensitivity of an enantioselective reaction has been reported recently: Y. Liu, G. Luo, X. Yang, S. Jiang, W. Xue, Y. R. Chi, Z. Jin, *Angew. Chem.* **2020**, *132*, 450–456.

6) Marti, C.; Carreira, E. M. *J. Am. Chem. Soc.* **2005**, *127*, 11505–11515.

7) V. M. Muzalevskiy, E. S. Balenkova, A. V. Shastin, A. M. Magerramov, N. G. Shikhaliev, V. G. Nenajdenko, *Russ. Chem. Bull.* **2011**, *60*, 2343–2346.

8) L.-Y. Mei, X.-Y. Tang, M. Shi, *Chem. Eur. J.* **2014**, *20*, 13136–13142.

9) X. Yao, T. Wang, Z. Zhang, *Eur. J. Org. Chem.* **2018**, *2018*, 4475–4478.

10) Muthusamy, S.; Gunanathan, C.; Nethaji, M. *J. Org. Chem.* **2004**, *69*, 5631–5637.

11) B. M. Trost, J. Xie, J. D. Sieber, *J. Am. Chem. Soc.* **2011**, *133*, 20611–20622.

12) K. E. Eckert, A. J. Lepore, B. L. Ashfeld, *Helv. Chim. Acta* **2019**, *102*, e1900192.

13) L. I. Manasevia, B. U. Maria, A. Prandi, L. Brasili, S. Franchini, *Synthesis* **2015**, *47*, 3767–3775.

14) A. J. Brouwer, S. J. E. Mulders, R. M. J. Liskamp, *Eur. J. Org. Chem.* **2001**, *2001*, 1903–1915.

15) S. R. Chhabra, A. Mahajan, W. C. Chan, *J. Org. Chem.* **2002**, *67*, 4017–4029.

16) C. Hunter, R. F. W. Jackson, H. K. Rami, *J. Chem. Soc. Perkin Trans. 1* **2000**, 219–223.

17) G. Povie, A.-T. Tran, D. Bonnaffé, J. Habegger, Z. Hu, C. Le Narvor, P. Renaud, *Angew. Chem. Int. Ed.* **2014**, *53*, 3894–3898.

18) P. H. Huy, Method of Converting Alcohol to Halide, WO2016202894A1, 2016.

19) M. F. G. Stevens, J. A. Hickman, R. Stone, N. W. Gibson, G. U. Baig, E. Lunt, C. G. Newton, *J. Med. Chem.* **1984**, *27*, 196–201.

20) M. J. Kohl, R. G. Lejeune, *Steroids* **2002**, *67*, 71–75

21) S. Wu, S. Lee, P. Beak, *J. Am. Chem. Soc.* **1996**, *118*, 715–721.

22) King, H; Wright, J. *J. Chem. Soc.*, **1948**, 2314–2318.

23) A. Banerji, S. Maiti, *Tetrahedron* **1994**, *50*, 9079–9096.

24) S. Arai, T. Ishida, T. Shiori, *Tetrahedron Lett.* **1998**, *39*, 8299–8302.

25) R. Li, Z. Liu, L. Chen, J. Pan, W. Zhou, *Beilstein J. Org. Chem.* **2018**, *14*, 1421–1427.

26) G. Ma, W. Wan, J. Li, Q. Hu, H. Jiang, S. Zhu, J. Wang, J. Hao, *Chem. Commun.* **2014**, *50*, 9749–9752.

27) D. Belmessieri, A. De La Houpliere, E. D. D. Calder, J. E. Taylor, A. D. Smith, *Chem. Eur. J.* **2014**, *20*, 9762–9769.

28) L. Roiser, M. Waser, *Org. Lett.* **2017**, *19*, 2338–2341.

29) M. A. C. Oudemans, *Recueil des Travaux Chimiques des Pays-Bas*, **1889**, *8*, 147–172.

30) O. Hesse, *Justus Liebigs Annalen der Chemie*, **1885**, *227*, 153–161.

31) M. S. Manna, V. Kumar, S. Mukherjee, *Chem. Commun.* **2012**, *48*, 5193–5195.

32) K. C. Nicolaou, G. Liu, K. Beabout, M. D. McCurry, Y. Shamoo, *J. Am. Chem. Soc.* **2017**, *139*, 3736–3746.

33) M. H. Franz, S. Röper, R. Wartchow, H. M. R. Hoffmann, *J. Org. Chem.* **2004**, *69*, 2983–2991.

34) M. Zielińska-Błajet, M. Kucharska, J. Skarżewski, *Synthesis* **2006**, 1176–1182.

35) P. Rabe, *Justus Liebig's Ann. der Chemie* **1932**, *492*, 242–266.

36) J. M. Tsangaris, T. A. Kabanos, *Monatshefte für Chemie Chem. Mon.* **1982**, *113*, 1393–1398.

37) A. P. Gorka, K. S. Sherlach, A. C. De Dios, P. D. Roepea, *Antimicrob. Agents Chemother.* **2013**, *57*, 365–374.

38) R. Craig, M. Litvajova, S. A. Cronin, S. J. Connon, *Chem. Commun.* **2018**, *54*, 10108–10111.

39) G. Kobayashi, *Yakugaku Zasshi*, **1950**, *70*, 381–383.

40) L. Hintermann, M. Schmitz, U. Englert, *Angew. Chem. Int. Ed.* **2007**, *46*, 5164–5167.

41) Y. Wang, H. Yin, X. Tang, Y. Wu, Q. Meng, Z. Gao, *J. Org. Chem.* **2016**, *81*, 7042–7050.

42) S. E. Wengryniuk, A. Weickgenannt, C. Reiher, N. A. Strotman, K. Chen, M. D. Eastgate, P. S. Baran, *Org. Lett.* **2013**, *15*, 792–795.

43) S. Benard, F. Guibbal, E. Jestin, O. Meilhac, Int. Pat. App. WO2019063634A1, 2019.

44) M. D. Chappell, S. E. Conner, V. I. C. Gonzalez, J. E. Lamar, J. Li, J. S. Moyers, R. A. Owens, A. E. Allie, G. Zhu, Int. Pat. App. WO2005118542 (A1), 2005.

45) J. Shen, Y. Wang, K. Wang, Int. Pat. App. US2014171431 (A1), 2014.

46) T. Korenaga, T. Kosaki, R. Fukumura, T. Ema, T. Sakai, *Org. Lett.* **2005**, *7*, 4915–4917.

47) J. M. Birchall, R. Hazard, R. N. Haszeldine, A. W. Wakalski, *J. Chem. Soc. C Org.* **1967**, 47–50.

48) X. Mu, J. C. Axtell, N. A. Bernier, K. O. Kirlikovali, D. Jung, A. Umanzor, K. Qian, X. Chen, K. L. Bay, M. Kirollos, et al., *Chem* **2019**, *5*, 2461–2469.

49 L. Hintermann, M. Schmitz, U. Englert, *Angew. Chem. Int. Ed.* **2007**, *46*, 5164–5167.

50) C. Del Fiandra, M. Moccia, V. Cerulli, M. F. A. Adamo, *Chem. Commun.* **2016**, *52*, 1697–1700.

51) M. Lian, Z. Li, J. Du, Q. Meng, Z. Gao, *Eur. J. Org. Chem.* **2010**, *2010*, 6525–6530.

52) R. Li, Z. Liu, L. Chen, J. Pan, W. Zhou, *Beilstein J. Org. Chem.* **2018**, *14*, 1421–1427.
